# Supplementary material for: Systematic Analysis of Mouse Genome Reveals Distinct Evolutionary and Functional Properties Among Circadian and Ultradian Genes
Source: Front Physiol. 2018 Aug 23;9:1178. doi: 10.3389/fphys.2018.01178 (PMC6115496; doi:10.3389/fphys.2018.01178)
Supplement: TABLE S5 — Top five enriched pathways for the three oscillating gene subsets. [file Table_5.DOC]

| **Supplementary Table 5.** Top five enriched pathways for the three oscillating gene subsets | | | | |
| --- | --- | --- | --- | --- |
| **Class** | **Canonical Pathways** | **Ratio** | **-log(p-value)** | **Genes** |
| *8 h* | Acute Phase Response Signaling | 2.96E-02 | 4.05E00 | TTR,APOA1,APOA2,SOCS6,HMOX2 |
|  | LXR/RXR Activation | 3.31E-02 | 3.51E00 | PON1,TTR,APOA1,APOA2 |
|  | FXR/RXR Activation | 3.15E-02 | 3.43E00 | PON1,TTR,APOA1,APOA2 |
|  | NRF2-mediated Oxidative Stress Response | 2.22E-02 | 2.86E00 | PRDX1,CAT,EIF2AK3,DNAJC11 |
|  | Production of Nitric Oxide and Reactive Oxygen Species in Macrophages | 2.22E-02 | 2.86E00 | PON1,APOA1,APOA2,CAT |
| *12 h* | Unfolded protein response | 7.22E00 | 1.48E-01 | HSPA8,HSPA4,UBXN4,DDIT3,INSIG1,  Hspa1b,DNAJB9,HSPA5 |
|  | Protein Ubiquitination Pathway | 5.73E00 | 5.1E-02 | USP28,ANAPC10,DNAJB9,HSPA5,PSMC5,  UBE2G2,HSPA8,HSPA4,ANAPC4,HSPA13,  USP46,DNAJB1,VHL |
|  | Glucocorticoid Receptor Signaling | 2.18E00 | 2.91E-02 | HSPA8,POLR2I,HSPA4,GTF2E1,CDK7,  Hspa1b,IKBKE,HSPA5 |
|  | Aldosterone Signaling in Epithelial Cells | 2.37E00 | 3.95E-02 | HSPA8,HSPA4,HSPA13,DNAJB1,DNAJB9,  HSPA5 |
|  | Androgen Signaling | 2.29E00 | 4.5E-02 | POLR2I,HSPA4,GTF2E1,CDK7,DNAJB1 |
| *24 h* | Leukocyte Extravasation Signaling | 1,37E-01 | 2,56E00 | RAP1B,MMP20,ICAM1,CLDN20,MMP14,  CTNNA1,BCAR1,MMP8,VCL,PRKD1,ACTA1,  TIMP2,CLDN10,CRKL,ITGA2,MAPK8,GNAI1,  RDX,MMP10,PIK3R3,ARHGAP5,GNAI3,  ITGB2,MMP23B,ARHGAP9,WAS,RASGRP1,CLDN22 |
|  | Bladder Cancer Signaling | 1,72E-01 | 2,51E00 | DAPK1,MMP20,TFDP1,FGF2,MMP14,EGF,  MMP10,FGF13,VEGFA,FGF17,FGF4,  MMP23B,MMP8,FGF20,FGF19 |
|  | D-myo-inositol-5-phosphate Metabolism | 1,45E-01 | 2,4E00 | DUSP8,MTMR9,EPHX2,PPFIBP2,DOT1L,  PP2D1,PPP1R14A,NUDT14,IGBP1,NUDT2,  STYX,PTPRM,DUSP14,SACM1L,PLCD1,PLD4,  PPP1R13B,DUSP10,PGAM5,SSH3,PLCB3,PALD1 |
|  | Actin Cytoskeleton Signaling | 1,32E-01 | 2,37E00 | MYH10,PFN1,FGF2,EGF,BCAR1,FGF13,  FGF4,CFL2,Ppp1r12a,PFN4,PFN3,VCL,  ACTA1,FGF19,ARHGEF12,CSK,CRKL,  ITGA2,RDX,Ins1,MYL7,PIK3R3,FGF17,  DOCK1,WAS,SSH3,FGF20,NCKAP1L,PDGFD |
|  | HIF1α Signaling | 1,54E-01 | 2,14E00 | SLC2A5,MMP20,MMP14,MAPK8,MAPK6,  MMP10,ARNT,PIK3R3,VEGFA,MMP23B,  AKT1,EGLN1,SLC2A2,MMP8,NCOA1,VHL |
