# Supplementary material for: Systematic Analysis of Mouse Genome Reveals Distinct Evolutionary and Functional Properties Among Circadian and Ultradian Genes
Source: Front Physiol. 2018 Aug 23;9:1178. doi: 10.3389/fphys.2018.01178 (PMC6115496; doi:10.3389/fphys.2018.01178)
Supplement: TABLE S6 — Comparative analysis based on −log(p-value) of enriched pathways among the three oscillating gene subsets. [file Table_6.DOC]

| **Supplementary Table 6.** Comparative analysis based on -log(p-value) of enriched pathways among the three oscillating gene subsets | | | |
| --- | --- | --- | --- |
| *IPA Canonical Pathway* | *24h* | *12h* | *8h* |
| Protein Ubiquitination Pathway | 0.52 | **5.64** | 0.33 |
| Acute Phase Response Signaling | 0.43 | 0.37 | **5.49** |
| Unfolded protein response | 0.00 | **5.20** | 0.92 |
| NRF2-mediated Oxidative Stress Response | 1.58 | 1.17 | **3.05** |
| Production of Nitric Oxide and Reactive Oxygen Species in Macrophages | 0.84 | 0.68 | **2.99** |
| Complement System | **2.08** | 0.00 | **2.43** |
| FXR/RXR Activation | 0.72 | 0.00 | **3.49** |
| LXR/RXR Activation | 0.00 | 0.00 | **3.60** |
| Endoplasmic Reticulum Stress Pathway | 0.30 | **1.87** | 1.31 |
| Atherosclerosis Signaling | 0.94 | 0.00 | **2.48** |
