# Supplementary material for: Systematic Analysis of Mouse Genome Reveals Distinct Evolutionary and Functional Properties Among Circadian and Ultradian Genes
Source: Front Physiol. 2018 Aug 23;9:1178. doi: 10.3389/fphys.2018.01178 (PMC6115496; doi:10.3389/fphys.2018.01178)
Supplement: TABLE S7 — Summary of the top ranking over-represented Transcription Factor Binding Sites, as estimated by AME (MEME suite) comparative analyses. [file Table_7.DOC]

| **Supplementary Table 7.** Summary of the top ranking over-represented Transcription Factor Binding Sites, as estimated by AME (MEME suite) comparative analyses | | | | |
| --- | --- | --- | --- | --- |
| **Motif name** | **motif sequence** | **Source** | **corrected p-value** | **found significant vs.** |
| UP00065_1 Zfp161_primary | KGGCGCGCGCRCHYRD | Uniprobe_mouse | 4.074e-07 | 12h: 5 x 1500, 5 x 500, 24h |
| XBP1_MOUSE.H10MO.C | GACGTGKCMTWW | HOCOMOCOv10_MOUSE | 7.798e-06 | 12h: 5 x 1500, 5 x 500, 24h |
| UP00065_2 Zfp161_secondary | GYCGCGCARNGCRN | Uniprobe_mouse | 1.439e-05 | 12h: 5 x 1500, 5 x 500, 24h |
| MBD2_MOUSE.H10MO.B | SSGKCCGGMGR | HOCOMOCOv10_MOUSE | 8.358e-05 | 12h: 5 x 1500, 5 x 500, 24h |
| UP00013_1 Gabpa_primary | MNWWACCGGAAGTDNNN | Uniprobe_mouse | 0.004514 | 12h: 5 x 1500, 54 x 500, 24h |
| ERR3_MOUSE.H10MO.B | TCAAGGTCA | HOCOMOCOv10_MOUSE | 0.003423 | 24h: 1 x 1500 |
| E4F1_MOUSE.H10MO.D | YGTKACGTC | HOCOMOCOv10_MOUSE | 0.03171 | 24h: 1 x 1500 |
| For each significant motif name, degenerate sequence and queried data source are given; Fisher’s exact test corrected p-value for the first comparison; number and typology of comparison (considered oscillating gene subset vs. control set; controls can be: 1500 or 500 random mouse sequences (5 set each) or another oscillating gene subset. Only comparisons with statistically significant results are shown. | | | | |
