# Supplementary material for: Systematic Analysis of Mouse Genome Reveals Distinct Evolutionary and Functional Properties Among Circadian and Ultradian Genes
Source: Front Physiol. 2018 Aug 23;9:1178. doi: 10.3389/fphys.2018.01178 (PMC6115496; doi:10.3389/fphys.2018.01178)
Supplement: FILE S1 — Original output of CodonW. [file Data_Sheet_1.doc]

title Transcript_length T3s C3s A3s G3s CAI CBI Fop Nc GC3s GC L_sym L_aa Gravy Aromo

ENSMUST00000000161 2217 0.2267 0.4667 0.2139 0.3805 0.293 0.231 0.559 45.91 0.653 0.541 721 738 -0.310705 0.071816

ENSMUST00000000001 1065 0.3616 0.3247 0.3320 0.3074 0.257 0.030 0.450 52.16 0.465 0.438 342 354 -0.367514 0.098870

ENSMUST00000000096 1659 0.1861 0.5247 0.1335 0.4000 0.253 0.114 0.491 43.16 0.740 0.591 530 552 -0.535688 0.086957

ENSMUST00000000137 1185 0.3730 0.2701 0.3154 0.3114 0.236 0.034 0.427 51.09 0.446 0.452 379 394 -0.184772 0.088832

ENSMUST00000000122 1284 0.1733 0.5028 0.1692 0.3832 0.291 0.172 0.523 43.45 0.719 0.619 417 427 -0.258548 0.044496

ENSMUST00000000087 1995 0.2915 0.4059 0.2401 0.2919 0.213 0.040 0.434 53.33 0.564 0.528 640 664 -0.470181 0.072289

ENSMUST00000000175 480 0.2727 0.4056 0.1694 0.2975 0.219 0.151 0.487 46.73 0.610 0.579 154 159 0.420126 0.075472

ENSMUST00000000028 1701 0.2870 0.4009 0.1970 0.4199 0.252 0.037 0.440 53.52 0.621 0.509 541 566 -0.323145 0.102473

ENSMUST00000000109 3063 0.2884 0.3707 0.2923 0.3051 0.209 0.017 0.430 54.27 0.529 0.493 983 1020 -0.310490 0.097059

ENSMUST00000000095 2136 0.1378 0.4958 0.1353 0.4130 0.245 0.182 0.524 41.22 0.767 0.669 691 711 -0.418987 0.064698

ENSMUST00000000090 441 0.3902 0.3415 0.2389 0.2381 0.213 -0.037 0.387 61.00 0.472 0.523 142 146 -0.158904 0.068493

ENSMUST00000000058 489 0.2687 0.4701 0.1429 0.3725 0.297 0.097 0.471 48.71 0.660 0.523 153 162 0.270988 0.111111

ENSMUST00000000127 1068 0.1581 0.5808 0.1315 0.3924 0.290 0.186 0.534 41.36 0.768 0.609 341 355 -0.362254 0.087324

ENSMUST00000000080 957 0.3320 0.3633 0.2616 0.3013 0.230 0.071 0.469 59.69 0.524 0.510 309 318 -0.770755 0.078616

ENSMUST00000000153 1140 0.0970 0.5853 0.0777 0.5405 0.299 0.276 0.574 35.94 0.861 0.598 366 379 -0.357256 0.092348

ENSMUST00000000049 1038 0.2975 0.3548 0.2951 0.3391 0.234 0.006 0.425 58.75 0.533 0.496 332 345 -0.258551 0.110145

ENSMUST00000000003 525 0.2880 0.3280 0.3651 0.3540 0.184 -0.092 0.362 60.41 0.497 0.410 163 174 -0.235058 0.097701

ENSMUST00000000129 2472 0.3415 0.2735 0.3354 0.3793 0.214 -0.026 0.406 54.44 0.482 0.442 789 823 -0.512637 0.071689

ENSMUST00000000033 543 0.1830 0.5359 0.1429 0.3333 0.228 0.179 0.514 44.17 0.726 0.615 175 180 -0.253889 0.083333

ENSMUST00000000163 1113 0.2984 0.3836 0.2241 0.3172 0.242 0.082 0.458 54.58 0.564 0.504 358 370 -0.011892 0.067568

ENSMUST00000000186 756 0.1991 0.4602 0.1361 0.3784 0.239 0.192 0.518 47.18 0.710 0.622 245 251 -0.360956 0.079681

ENSMUST00000000010 753 0.1526 0.4789 0.1218 0.5052 0.245 0.207 0.544 46.94 0.780 0.621 241 250 -0.849600 0.088000

ENSMUST00000000171 948 0.3648 0.2790 0.3478 0.2838 0.214 0.036 0.429 61.00 0.429 0.438 303 315 -0.258730 0.047619

ENSMUST00000000275 1443 0.3273 0.3144 0.3313 0.3016 0.192 -0.056 0.381 58.09 0.473 0.450 452 480 -0.102708 0.129167

ENSMUST00000000260 1038 0.2186 0.4301 0.2261 0.3770 0.240 0.017 0.437 48.41 0.639 0.548 332 345 -0.085507 0.072464

ENSMUST00000000201 5220 0.3230 0.3252 0.2990 0.3188 0.208 0.019 0.417 55.33 0.497 0.459 1643 1739 0.078493 0.115584

ENSMUST00000000193 447 0.2667 0.3917 0.2689 0.2609 0.253 0.104 0.475 54.13 0.546 0.491 141 148 -0.078378 0.067568

ENSMUST00000000299 2313 0.2536 0.4588 0.1618 0.4305 0.270 0.090 0.479 47.49 0.673 0.548 753 770 -0.378052 0.068831

ENSMUST00000000285 1695 0.2796 0.3848 0.2825 0.3414 0.252 0.132 0.492 55.64 0.557 0.505 537 564 -0.248227 0.086879

ENSMUST00000000304 600 0.2797 0.3357 0.3377 0.3265 0.217 0.058 0.452 54.96 0.511 0.506 188 199 -0.600502 0.060302

ENSMUST00000000287 1359 0.2778 0.4327 0.2128 0.3452 0.271 0.110 0.481 54.58 0.607 0.514 420 452 -0.078982 0.121681

ENSMUST00000000206 1437 0.1646 0.4552 0.0822 0.4809 0.297 0.300 0.581 40.61 0.786 0.659 463 478 -0.047908 0.085774

ENSMUST00000000266 1338 0.4182 0.2515 0.4356 0.2290 0.235 -0.058 0.406 49.68 0.350 0.378 431 445 -0.591910 0.092135

ENSMUST00000000291 1776 0.1979 0.4042 0.1714 0.4071 0.214 0.047 0.418 46.22 0.684 0.637 582 591 -0.463452 0.013536

ENSMUST00000000221 1968 0.2224 0.5029 0.2158 0.3403 0.273 0.172 0.522 49.06 0.653 0.518 623 655 -0.266565 0.111450

ENSMUST00000000188 870 0.2166 0.4378 0.1422 0.5097 0.381 0.250 0.554 41.13 0.719 0.565 278 289 -0.125952 0.062284

ENSMUST00000000194 315 0.2976 0.3810 0.1928 0.3973 0.201 0.027 0.422 51.74 0.598 0.487 102 104 0.039423 0.048077

ENSMUST00000000253 1221 0.1647 0.5147 0.1500 0.4041 0.268 0.134 0.505 44.65 0.744 0.597 394 406 -0.469951 0.066502

ENSMUST00000000208 1809 0.3312 0.3009 0.3415 0.3230 0.223 -0.006 0.418 55.41 0.472 0.450 581 602 -0.219601 0.098007

ENSMUST00000000199 573 0.1942 0.4964 0.1544 0.5197 0.300 0.124 0.503 42.77 0.738 0.526 183 190 -0.516842 0.126316

ENSMUST00000000254 810 0.3380 0.3803 0.2833 0.2788 0.223 0.043 0.456 50.79 0.508 0.467 250 269 -0.351301 0.126394

ENSMUST00000000187 627 0.1500 0.4778 0.2108 0.3397 0.191 0.131 0.483 46.99 0.692 0.579 201 208 -0.032212 0.091346

ENSMUST00000000305 615 0.2466 0.3493 0.3086 0.3718 0.255 0.039 0.467 56.90 0.559 0.500 195 204 -0.702451 0.049020

ENSMUST00000000219 1497 0.2351 0.4084 0.1897 0.4213 0.259 0.065 0.455 47.50 0.657 0.577 492 498 -0.412651 0.094378

ENSMUST00000000544 1518 0.2694 0.4612 0.1662 0.3533 0.252 0.114 0.479 50.87 0.642 0.543 480 505 -0.063960 0.087129

ENSMUST00000000365 546 0.4962 0.1805 0.4586 0.1810 0.221 -0.090 0.360 47.17 0.262 0.361 172 181 -0.126519 0.082873

ENSMUST00000000395 1473 0.2543 0.3827 0.2663 0.3364 0.214 0.007 0.428 53.45 0.575 0.525 463 490 -0.202245 0.095918

ENSMUST00000000356 507 0.4257 0.2838 0.2727 0.1563 0.212 -0.061 0.373 50.66 0.385 0.542 161 168 -0.098214 0.107143

ENSMUST00000000574 960 0.2308 0.4615 0.1754 0.3415 0.252 0.177 0.502 49.89 0.656 0.495 299 319 0.753291 0.131661

ENSMUST00000000421 807 0.2512 0.4089 0.2426 0.3850 0.239 0.039 0.435 50.28 0.608 0.526 255 268 -0.172388 0.085821

ENSMUST00000000449 1251 0.3129 0.3896 0.2642 0.3298 0.248 0.040 0.455 56.49 0.550 0.517 402 416 -0.602885 0.084135

ENSMUST00000000430 1680 0.3676 0.2694 0.3775 0.2717 0.189 -0.019 0.402 52.67 0.411 0.441 530 559 -0.348122 0.093023

ENSMUST00000000412 1653 0.3096 0.3349 0.3410 0.3166 0.209 -0.027 0.407 55.88 0.497 0.491 535 550 -0.590182 0.072727

ENSMUST00000000329 1992 0.2640 0.3873 0.2238 0.3800 0.227 0.096 0.460 53.19 0.605 0.548 628 663 -0.229110 0.090498

ENSMUST00000000326 1425 0.2525 0.4118 0.2713 0.2658 0.226 0.113 0.483 53.27 0.564 0.573 470 474 -0.428481 0.067511

ENSMUST00000000542 1509 0.1966 0.4272 0.1497 0.4746 0.228 0.111 0.472 48.03 0.715 0.578 481 502 -0.216135 0.075697

ENSMUST00000000310 600 0.1765 0.4824 0.1831 0.3582 0.260 0.225 0.538 38.81 0.699 0.549 186 199 0.423116 0.160804

ENSMUST00000000514 1125 0.3381 0.3452 0.3127 0.2970 0.239 0.040 0.451 52.66 0.493 0.465 357 374 -0.250000 0.096257

ENSMUST00000000348 1101 0.2814 0.3356 0.2559 0.3542 0.217 0.058 0.452 59.49 0.551 0.527 354 366 -0.000546 0.051913

ENSMUST00000000327 918 0.2121 0.4502 0.1894 0.4358 0.267 0.164 0.515 48.73 0.684 0.548 291 305 -0.535410 0.081967

ENSMUST00000000573 2166 0.4109 0.3039 0.2074 0.2873 0.242 0.030 0.448 50.35 0.480 0.474 690 721 -0.265603 0.090153

ENSMUST00000000335 798 0.1737 0.4648 0.1782 0.4188 0.258 0.146 0.484 46.63 0.710 0.575 252 265 0.110943 0.086792

ENSMUST00000000369 894 0.1633 0.4735 0.2024 0.3527 0.253 0.190 0.522 42.18 0.691 0.629 291 297 -0.540404 0.047138

ENSMUST00000000342 294 0.1333 0.5600 0.2468 0.3239 0.297 0.255 0.564 37.95 0.691 0.509 94 97 -0.171134 0.061856

ENSMUST00000000312 2655 0.2589 0.4370 0.2057 0.3549 0.246 0.091 0.468 51.10 0.623 0.541 863 884 -0.417308 0.073529

ENSMUST00000000349 1449 0.3125 0.3281 0.3037 0.3029 0.255 0.106 0.474 55.16 0.496 0.486 468 482 -0.145850 0.074689

ENSMUST00000000641 2334 0.2744 0.3598 0.1765 0.3786 0.210 0.055 0.436 51.74 0.614 0.592 754 777 -0.118533 0.073359

ENSMUST00000000619 2244 0.2653 0.3907 0.1738 0.3933 0.213 0.049 0.435 50.74 0.629 0.536 706 747 0.208568 0.113788

ENSMUST00000000445 768 0.2233 0.5194 0.1780 0.3279 0.293 0.159 0.522 46.93 0.676 0.569 247 255 -0.667059 0.062745

ENSMUST00000000590 1752 0.3784 0.2649 0.3691 0.3462 0.237 -0.008 0.423 59.81 0.445 0.449 562 583 -0.999657 0.068611

ENSMUST00000000388 1362 0.2520 0.4209 0.1785 0.3963 0.259 0.099 0.477 50.74 0.646 0.550 444 453 -0.364238 0.061810

ENSMUST00000000476 3270 0.2587 0.4304 0.2093 0.3760 0.249 0.081 0.463 52.06 0.624 0.509 1047 1089 -0.284022 0.082645

ENSMUST00000000314 2742 0.1971 0.4941 0.1866 0.3580 0.250 0.133 0.494 46.81 0.684 0.566 881 913 -0.316868 0.069003

ENSMUST00000000466 1278 0.2719 0.4063 0.1716 0.4118 0.295 0.084 0.473 50.33 0.645 0.530 408 425 -0.279294 0.056471

ENSMUST00000000594 426 0.3061 0.2755 0.4037 0.2925 0.252 0.074 0.462 44.97 0.439 0.437 132 141 -0.568794 0.056738

ENSMUST00000000543 1179 0.1574 0.4784 0.1377 0.4123 0.233 0.167 0.508 46.17 0.749 0.662 386 392 -0.435714 0.066327

ENSMUST00000000451 1947 0.2967 0.3661 0.2427 0.3503 0.255 0.114 0.486 52.96 0.562 0.509 619 648 -0.405864 0.080247

ENSMUST00000000608 582 0.2298 0.4969 0.1656 0.3151 0.259 0.173 0.497 45.35 0.667 0.577 189 193 0.079275 0.062176

ENSMUST00000000317 1257 0.2957 0.3449 0.2422 0.3432 0.209 0.051 0.437 49.72 0.553 0.540 403 418 -0.405981 0.071770

ENSMUST00000000450 1518 0.3000 0.3897 0.2555 0.3769 0.257 0.056 0.453 53.29 0.568 0.481 486 505 -0.292673 0.091089

ENSMUST00000000500 726 0.1846 0.4410 0.1632 0.4556 0.201 0.008 0.413 46.11 0.715 0.595 235 241 -0.519917 0.041494

ENSMUST00000000384 3777 0.2655 0.4043 0.2143 0.3813 0.255 0.121 0.483 50.63 0.616 0.527 1212 1258 -0.250874 0.081876

ENSMUST00000000505 2160 0.3084 0.3512 0.2138 0.3864 0.226 0.055 0.445 51.99 0.577 0.531 695 719 -0.355911 0.068150

ENSMUST00000000579 1524 0.1192 0.5933 0.1050 0.4496 0.305 0.278 0.587 37.59 0.821 0.641 491 507 -1.021.499 0.069034

ENSMUST00000000704 675 0.1543 0.5314 0.0765 0.5060 0.283 0.227 0.562 42.27 0.816 0.644 217 224 -0.870536 0.125000

ENSMUST00000000769 1254 0.2047 0.5117 0.1467 0.3524 0.215 0.106 0.467 46.28 0.706 0.562 405 417 -0.138130 0.076739

ENSMUST00000000707 2265 0.2757 0.4192 0.1938 0.3635 0.241 0.082 0.474 49.03 0.619 0.568 721 754 -0.394032 0.084881

ENSMUST00000001081 1182 0.1914 0.4455 0.1572 0.5000 0.259 0.150 0.496 47.31 0.723 0.567 379 393 -0.128753 0.061069

ENSMUST00000000717 366 0.2900 0.3200 0.2921 0.3293 0.188 0.013 0.412 42.43 0.518 0.499 114 121 -0.385951 0.049587

ENSMUST00000000746 1266 0.2530 0.3988 0.1935 0.4262 0.238 0.107 0.475 50.26 0.643 0.546 406 421 -0.397862 0.097387

ENSMUST00000000958 1782 0.1939 0.4346 0.1844 0.4989 0.243 0.059 0.453 48.83 0.708 0.554 569 593 -0.682293 0.086003

ENSMUST00000001043 675 0.2787 0.2459 0.3316 0.2819 0.120 0.020 0.421 55.51 0.458 0.568 214 224 -0.763839 0.013393

ENSMUST00000001040 789 0.2043 0.3913 0.1767 0.3702 0.187 0.083 0.437 51.47 0.663 0.594 252 262 0.248473 0.072519

ENSMUST00000000985 1302 0.2521 0.4221 0.2122 0.3199 0.214 0.075 0.440 53.25 0.609 0.557 414 433 0.046189 0.076212

ENSMUST00000000804 1989 0.4077 0.2358 0.3725 0.2273 0.204 -0.024 0.418 51.16 0.365 0.463 641 662 -0.613142 0.093656

ENSMUST00000001027 4002 0.3227 0.3321 0.2794 0.3226 0.213 -0.008 0.413 54.11 0.510 0.500 1286 1333 -0.141710 0.072018

ENSMUST00000000767 1935 0.2854 0.3866 0.2366 0.3850 0.241 0.075 0.472 55.32 0.588 0.506 621 644 -0.455124 0.080745

ENSMUST00000000727 648 0.3110 0.3354 0.2575 0.3711 0.230 -0.019 0.423 57.39 0.548 0.507 208 215 -0.377209 0.079070

ENSMUST00000000793 1197 0.2386 0.3719 0.2364 0.4159 0.221 0.044 0.436 49.16 0.619 0.560 383 398 -0.808543 0.025126

ENSMUST00000000674 1527 0.3039 0.3088 0.3656 0.2829 0.191 -0.009 0.410 55.87 0.464 0.470 485 508 -0.171260 0.110236

ENSMUST00000001130 573 0.3025 0.3210 0.2308 0.3243 0.193 -0.008 0.405 49.87 0.541 0.561 185 190 -0.384211 0.057895

ENSMUST00000000895 1062 0.2581 0.3835 0.1863 0.4414 0.241 0.120 0.493 52.23 0.645 0.573 341 353 -0.485269 0.084986

ENSMUST00000000828 1848 0.2766 0.3586 0.2653 0.3705 0.245 0.050 0.447 55.52 0.564 0.507 599 614 -0.174104 0.076547

ENSMUST00000000924 1479 0.2758 0.4005 0.1698 0.3565 0.213 0.053 0.433 49.14 0.624 0.593 473 492 -0.310569 0.115854

ENSMUST00000000896 639 0.1818 0.5341 0.0974 0.4306 0.250 0.173 0.507 43.49 0.768 0.577 203 212 0.078302 0.122642

ENSMUST00000000808 2217 0.1962 0.4437 0.1722 0.4000 0.220 0.109 0.477 46.63 0.693 0.601 698 738 -0.440244 0.075881

ENSMUST00000001042 1173 0.3419 0.2806 0.2889 0.3242 0.191 -0.016 0.398 55.81 0.480 0.496 379 390 -0.234872 0.064103

ENSMUST00000001148 1116 0.2177 0.4898 0.2186 0.2982 0.251 0.128 0.494 52.09 0.631 0.556 358 371 -0.167385 0.035040

ENSMUST00000000925 1158 0.2150 0.4471 0.1901 0.4427 0.241 0.067 0.459 44.57 0.679 0.538 364 385 -0.576104 0.077922

ENSMUST00000000964 1011 0.2734 0.4676 0.1760 0.3264 0.268 0.173 0.538 53.86 0.635 0.566 329 336 -0.824405 0.080357

ENSMUST00000000844 621 0.3376 0.3057 0.2249 0.3697 0.249 0.110 0.485 55.92 0.545 0.524 200 206 -0.647573 0.038835

ENSMUST00000000811 2199 0.2292 0.4890 0.1511 0.3733 0.243 0.146 0.498 47.68 0.687 0.564 705 732 -0.252323 0.065574

ENSMUST00000000809 1857 0.1771 0.4871 0.1189 0.3605 0.248 0.177 0.505 45.38 0.737 0.615 586 618 0.533657 0.098705

ENSMUST00000000646 2934 0.2332 0.4627 0.1902 0.3208 0.236 0.080 0.460 49.09 0.646 0.591 954 977 -0.278608 0.085977

ENSMUST00000001036 3624 0.2189 0.4532 0.1611 0.4162 0.243 0.133 0.487 46.25 0.690 0.584 1166 1207 -0.342751 0.083679

ENSMUST00000001008 279 0.2632 0.5526 0.2090 0.2097 0.346 0.316 0.607 45.75 0.618 0.518 89 92 0.078261 0.108696

ENSMUST00000000718 363 0.3333 0.3125 0.3500 0.2692 0.217 -0.021 0.405 60.13 0.459 0.475 111 120 -0.431667 0.125000

ENSMUST00000001080 1614 0.2327 0.4158 0.1543 0.4238 0.226 0.130 0.490 44.55 0.686 0.624 525 537 -0.859777 0.052142

ENSMUST00000001002 3555 0.2429 0.3817 0.2574 0.3408 0.226 0.097 0.465 54.66 0.585 0.535 1143 1184 -0.063682 0.055743

ENSMUST00000001092 1845 0.2305 0.4465 0.1900 0.3790 0.233 0.087 0.476 48.02 0.660 0.597 597 614 -0.696091 0.052117

ENSMUST00000001125 951 0.1633 0.5388 0.1369 0.4554 0.267 0.140 0.505 44.26 0.762 0.575 307 316 -0.525317 0.079114

ENSMUST00000001127 1107 0.2649 0.3510 0.2491 0.3616 0.203 0.020 0.427 57.72 0.576 0.557 354 368 -0.525543 0.103261

ENSMUST00000001147 3078 0.2420 0.3950 0.2438 0.3605 0.215 0.018 0.434 50.68 0.602 0.579 1008 1025 -0.507024 0.063415

ENSMUST00000000901 2454 0.2985 0.3788 0.2188 0.3671 0.218 0.003 0.422 50.34 0.579 0.533 794 817 -0.514321 0.073439

ENSMUST00000000755 876 0.2115 0.5551 0.1422 0.3737 0.294 0.238 0.558 45.39 0.719 0.537 274 291 -0.237457 0.144330

ENSMUST00000001112 1725 0.3613 0.2774 0.4248 0.2617 0.191 -0.049 0.391 50.89 0.398 0.416 553 574 -0.501220 0.106272

ENSMUST00000001051 270 0.2813 0.4531 0.1176 0.5161 0.388 0.188 0.529 35.82 0.701 0.517 87 89 -0.252809 0.067416

ENSMUST00000000984 1533 0.2573 0.5011 0.1550 0.2811 0.265 0.138 0.486 48.83 0.646 0.528 494 510 0.711569 0.127451

ENSMUST00000001122 1761 0.2058 0.4740 0.1824 0.3454 0.248 0.126 0.474 49.54 0.671 0.546 553 586 0.550171 0.104096

ENSMUST00000000642 2754 0.2242 0.4387 0.1447 0.4694 0.260 0.088 0.468 46.93 0.703 0.563 874 917 -0.191167 0.064340

ENSMUST00000000756 636 0.1887 0.4654 0.1685 0.4277 0.218 0.158 0.507 42.09 0.707 0.585 205 211 -0.872038 0.056872

ENSMUST00000001009 225 0.4262 0.3279 0.2941 0.1600 0.254 0.026 0.449 48.95 0.406 0.500 69 74 -0.043243 0.067568

ENSMUST00000000940 1398 0.2474 0.3842 0.1768 0.4043 0.229 0.078 0.453 50.10 0.648 0.589 457 465 -0.235269 0.068817

ENSMUST00000000939 3207 0.1715 0.4524 0.1419 0.5042 0.258 0.096 0.473 43.56 0.750 0.597 1040 1068 -0.537547 0.042135

ENSMUST00000001047 306 0.2162 0.5135 0.1286 0.5147 0.279 0.058 0.459 41.89 0.745 0.564 98 101 -0.487129 0.118812

ENSMUST00000001115 1770 0.2483 0.4139 0.1780 0.4437 0.243 0.079 0.461 48.20 0.663 0.565 570 589 -0.500000 0.089983

ENSMUST00000000724 2442 0.2597 0.3887 0.2785 0.3590 0.221 0.045 0.447 53.47 0.572 0.499 788 813 -0.498524 0.087331

ENSMUST00000001046 306 0.1806 0.4861 0.2143 0.5000 0.280 0.077 0.474 52.38 0.711 0.508 97 101 -0.601980 0.099010

ENSMUST00000001055 834 0.2182 0.4227 0.2066 0.4112 0.222 0.073 0.455 52.05 0.654 0.538 266 277 -0.036462 0.108303

ENSMUST00000000687 861 0.2123 0.3726 0.2136 0.4737 0.197 0.000 0.411 47.80 0.659 0.564 270 286 -0.551399 0.090909

ENSMUST00000000776 2718 0.2694 0.3611 0.2449 0.3920 0.235 0.142 0.495 54.43 0.587 0.513 876 905 -0.365967 0.087293

ENSMUST00000000889 423 0.2252 0.4144 0.2718 0.3684 0.192 -0.002 0.410 61.00 0.604 0.476 134 140 -0.058571 0.057143

ENSMUST00000000910 1869 0.2171 0.4841 0.1696 0.3948 0.246 0.107 0.477 47.32 0.688 0.565 596 622 -0.285370 0.096463

ENSMUST00000001109 1371 0.1809 0.4447 0.1635 0.3592 0.236 0.189 0.507 48.92 0.696 0.595 434 456 0.570395 0.111842

ENSMUST00000000881 2322 0.2343 0.4205 0.2233 0.3554 0.221 0.072 0.462 53.58 0.625 0.579 753 773 -0.734282 0.049159

ENSMUST00000000759 591 0.2132 0.4044 0.1111 0.5409 0.284 0.056 0.457 44.25 0.750 0.577 188 196 -0.458674 0.025510

ENSMUST00000000926 372 0.2661 0.4954 0.1977 0.2658 0.274 0.098 0.479 49.41 0.620 0.572 121 123 0.038211 0.113821

ENSMUST00000001126 1380 0.2170 0.4414 0.1328 0.3817 0.240 0.140 0.477 45.80 0.695 0.583 440 459 0.637037 0.115468

ENSMUST00000001063 1788 0.2791 0.3679 0.2525 0.3013 0.251 0.093 0.479 51.89 0.554 0.550 574 595 -0.554622 0.055462

ENSMUST00000000894 1158 0.2458 0.3854 0.1948 0.4247 0.267 0.136 0.489 47.88 0.642 0.566 374 385 -0.336364 0.051948

ENSMUST00000001108 1992 0.3321 0.3090 0.2451 0.3722 0.227 0.010 0.424 52.90 0.534 0.513 642 663 -0.504072 0.063348

ENSMUST00000001059 2934 0.2526 0.4503 0.1860 0.3668 0.254 0.111 0.476 49.50 0.644 0.543 945 977 -0.348823 0.089048

ENSMUST00000000834 840 0.2780 0.3184 0.3221 0.3350 0.218 0.059 0.438 52.09 0.517 0.491 267 279 -0.351613 0.111111

ENSMUST00000001079 3756 0.3165 0.3203 0.2916 0.2778 0.206 0.009 0.417 56.63 0.493 0.516 1216 1251 -0.349400 0.075939

ENSMUST00000001155 1815 0.2679 0.4397 0.1537 0.3727 0.231 0.076 0.458 49.20 0.652 0.563 581 604 -0.326987 0.084437

ENSMUST00000000696 225 0.2500 0.5469 0.2034 0.1698 0.241 0.155 0.514 51.85 0.611 0.482 72 74 0.705406 0.094595

ENSMUST00000001701 915 0.1667 0.5528 0.1404 0.3946 0.230 0.140 0.512 42.99 0.754 0.607 297 304 -0.767434 0.101974

ENSMUST00000001836 1506 0.3500 0.3200 0.2892 0.3153 0.259 0.032 0.442 57.59 0.492 0.496 486 501 -0.543114 0.075848

ENSMUST00000001675 1395 0.2853 0.3112 0.3647 0.3449 0.221 0.039 0.445 55.79 0.493 0.458 440 464 -0.548491 0.101293

ENSMUST00000001451 3054 0.2727 0.4318 0.2005 0.3595 0.245 0.093 0.468 49.65 0.621 0.546 986 1017 -0.406883 0.067847

ENSMUST00000001920 453 0.1942 0.4466 0.1770 0.4907 0.208 -0.028 0.403 48.49 0.712 0.536 139 150 -0.535333 0.066667

ENSMUST00000001834 1518 0.3383 0.3259 0.2642 0.3362 0.230 -0.028 0.405 53.92 0.514 0.504 481 505 -0.241386 0.065347

ENSMUST00000001561 690 0.1788 0.3966 0.1927 0.4628 0.227 0.184 0.511 45.54 0.696 0.607 227 229 -0.514847 0.021834

ENSMUST00000001326 2346 0.3657 0.3386 0.2489 0.2569 0.235 0.046 0.462 54.45 0.486 0.524 764 781 -0.445839 0.029449

ENSMUST00000001802 2220 0.2088 0.4812 0.1296 0.3894 0.250 0.154 0.497 45.59 0.716 0.602 700 739 -0.037348 0.121786

ENSMUST00000001716 357 0.1667 0.4896 0.1596 0.4023 0.284 0.331 0.602 44.87 0.726 0.573 113 118 0.090678 0.067797

ENSMUST00000001520 2370 0.3124 0.3366 0.2109 0.3792 0.217 0.032 0.435 52.29 0.572 0.543 761 789 -0.318885 0.087452

ENSMUST00000001592 2238 0.1824 0.5191 0.1098 0.4241 0.286 0.164 0.505 41.45 0.758 0.595 715 745 -0.161208 0.045638

ENSMUST00000001507 1512 0.3333 0.2811 0.3474 0.2983 0.211 -0.028 0.395 53.98 0.450 0.469 484 503 -0.160239 0.107356

ENSMUST00000002084 2211 0.2348 0.4088 0.2124 0.3528 0.230 0.076 0.448 46.92 0.624 0.572 707 736 -0.008560 0.081522

ENSMUST00000002090 519 0.2986 0.3819 0.2197 0.3197 0.272 0.097 0.476 54.37 0.566 0.521 166 172 0.140116 0.104651

ENSMUST00000001319 1011 0.2482 0.3849 0.2937 0.3261 0.207 0.058 0.452 55.51 0.560 0.496 325 336 -0.352976 0.083333

ENSMUST00000001900 1032 0.2424 0.4478 0.1660 0.3894 0.259 0.173 0.502 51.64 0.664 0.527 333 343 0.433528 0.139942

ENSMUST00000001927 5139 0.3052 0.4006 0.2392 0.3511 0.252 0.039 0.454 54.23 0.575 0.543 1670 1712 -0.503037 0.063084

ENSMUST00000001964 2199 0.1958 0.4783 0.2042 0.4082 0.253 0.103 0.479 46.08 0.684 0.541 693 732 -0.413661 0.117486

ENSMUST00000001652 1179 0.0846 0.5674 0.0979 0.4794 0.278 0.232 0.536 38.27 0.849 0.588 364 392 0.456122 0.114796

ENSMUST00000001166 2142 0.1098 0.4592 0.1128 0.4974 0.288 0.249 0.549 39.51 0.809 0.667 701 713 0.033520 0.060309

ENSMUST00000002080 2319 0.2658 0.4055 0.1683 0.4239 0.242 0.095 0.471 45.49 0.652 0.573 745 772 -0.816839 0.042746

ENSMUST00000001975 1545 0.2285 0.4619 0.2020 0.3511 0.277 0.132 0.500 45.39 0.650 0.564 492 514 -0.391051 0.066148

ENSMUST00000002048 885 0.3026 0.3728 0.2311 0.3667 0.271 0.115 0.488 55.29 0.572 0.522 283 294 -0.328912 0.054422

ENSMUST00000001878 807 0.2064 0.4633 0.1376 0.4038 0.246 0.180 0.523 47.58 0.714 0.607 262 268 -0.531343 0.085821

ENSMUST00000001186 468 0.2586 0.2931 0.4186 0.2581 0.145 0.081 0.473 52.80 0.440 0.484 150 155 -1.984.516 0.045161

ENSMUST00000001722 1158 0.1567 0.4514 0.1940 0.4172 0.194 0.163 0.509 49.20 0.710 0.615 373 385 -0.660519 0.085714

ENSMUST00000001565 1392 0.2087 0.4255 0.1425 0.4499 0.221 0.139 0.483 48.64 0.709 0.582 443 463 -0.062635 0.095032

ENSMUST00000001812 2382 0.2093 0.4729 0.1221 0.4042 0.231 0.091 0.461 45.32 0.719 0.602 759 793 -0.035687 0.094578

ENSMUST00000001497 660 0.2034 0.4237 0.2189 0.3675 0.196 0.075 0.445 51.00 0.651 0.565 209 219 -0.311415 0.082192

ENSMUST00000001183 1626 0.2414 0.3540 0.1845 0.4479 0.263 0.068 0.448 46.95 0.648 0.586 529 541 -0.075416 0.060998

ENSMUST00000001456 1176 0.2226 0.4116 0.1709 0.3849 0.193 0.054 0.425 49.94 0.665 0.601 379 391 -0.103581 0.099744

ENSMUST00000001672 1350 0.4350 0.2345 0.3099 0.2870 0.241 0.017 0.436 55.43 0.404 0.456 436 449 -0.350557 0.073497

ENSMUST00000001485 789 0.2440 0.4115 0.1810 0.3930 0.195 0.092 0.441 51.64 0.650 0.575 254 262 -0.170229 0.053435

ENSMUST00000001620 2034 0.4485 0.1966 0.4064 0.2071 0.211 0.021 0.438 52.58 0.315 0.440 660 677 -0.870753 0.057607

ENSMUST00000001699 1029 0.2045 0.4735 0.2205 0.3658 0.216 0.068 0.468 54.39 0.662 0.563 331 342 -0.938889 0.076023

ENSMUST00000001569 1287 0.2020 0.4205 0.1828 0.4718 0.262 0.039 0.448 48.74 0.692 0.573 413 428 -0.355140 0.042056

ENSMUST00000001242 801 0.2156 0.4358 0.1751 0.3971 0.235 0.032 0.437 50.43 0.674 0.593 261 266 -0.022180 0.037594

ENSMUST00000001202 2703 0.3661 0.3357 0.3063 0.3007 0.211 0.031 0.430 55.43 0.477 0.457 866 900 -0.450333 0.090000

ENSMUST00000001984 705 0.2283 0.4348 0.2753 0.2931 0.232 0.072 0.459 59.58 0.590 0.519 222 234 -0.227350 0.085470

ENSMUST00000001825 996 0.4286 0.2227 0.4382 0.2278 0.246 -0.039 0.411 54.39 0.335 0.417 319 331 -0.705740 0.069486

ENSMUST00000001416 1530 0.2794 0.3473 0.2255 0.4354 0.245 0.041 0.433 51.66 0.600 0.525 497 509 -0.287033 0.068762

ENSMUST00000002008 987 0.2714 0.4036 0.1698 0.3282 0.245 0.121 0.480 50.90 0.621 0.569 319 328 0.007317 0.060976

ENSMUST00000001806 1692 0.2658 0.3769 0.1394 0.4341 0.253 0.138 0.480 47.18 0.662 0.573 550 563 -0.132860 0.062167

ENSMUST00000001700 987 0.1292 0.5609 0.0920 0.4235 0.314 0.317 0.605 37.47 0.815 0.666 319 328 -0.579573 0.088415

ENSMUST00000001921 1254 0.3882 0.3416 0.3345 0.2423 0.254 0.046 0.452 54.01 0.437 0.416 396 417 -0.435492 0.122302

ENSMUST00000001599 1827 0.3091 0.4303 0.2029 0.3368 0.240 0.000 0.427 53.96 0.590 0.500 578 608 -0.214145 0.120066

ENSMUST00000001882 768 0.2228 0.5181 0.1768 0.3885 0.282 0.202 0.549 48.77 0.691 0.553 233 255 -0.476863 0.117647

ENSMUST00000001412 2895 0.3885 0.2679 0.3183 0.3416 0.208 -0.054 0.385 55.18 0.454 0.435 934 964 -0.435788 0.086100

ENSMUST00000001544 2382 0.3429 0.3157 0.2745 0.3252 0.237 -0.001 0.418 51.75 0.501 0.471 766 793 -0.164187 0.073140

ENSMUST00000001460 2733 0.2493 0.4244 0.1994 0.3929 0.231 0.027 0.432 50.08 0.639 0.555 888 910 -0.441319 0.074725

ENSMUST00000001179 8751 0.2331 0.3478 0.2272 0.4786 0.245 0.078 0.465 52.35 0.643 0.556 2846 2916 -0.813957 0.028464

ENSMUST00000001713 723 0.2500 0.3936 0.1638 0.4639 0.267 0.113 0.463 52.36 0.665 0.561 227 240 0.047500 0.087500

ENSMUST00000001703 729 0.1966 0.5225 0.2316 0.3793 0.244 0.139 0.519 49.63 0.677 0.551 235 242 -1.124.380 0.111570

ENSMUST00000001172 552 0.1975 0.4586 0.1374 0.4409 0.305 0.223 0.554 52.81 0.723 0.623 177 183 -0.231147 0.060109

ENSMUST00000001963 3939 0.1893 0.4816 0.1899 0.4237 0.251 0.103 0.477 47.36 0.700 0.557 1240 1312 -0.422866 0.124238

ENSMUST00000001631 2223 0.2835 0.3752 0.2184 0.3599 0.236 0.101 0.473 51.99 0.590 0.563 719 740 -0.368919 0.056757

ENSMUST00000001562 1383 0.2323 0.4363 0.2135 0.3947 0.250 0.085 0.470 50.82 0.647 0.557 447 460 -0.721522 0.060870

ENSMUST00000001187 960 0.2581 0.3952 0.2245 0.4063 0.238 0.099 0.484 48.82 0.614 0.513 308 319 -0.434483 0.075235

ENSMUST00000001720 1365 0.2814 0.4153 0.1647 0.4065 0.222 0.047 0.437 50.75 0.636 0.532 437 454 -0.131498 0.077093

ENSMUST00000002044 1881 0.2763 0.3817 0.2542 0.3385 0.194 0.017 0.430 53.28 0.569 0.522 605 626 -0.698882 0.068690

ENSMUST00000001402 1314 0.2746 0.3612 0.2862 0.3571 0.249 0.130 0.495 52.91 0.555 0.511 416 437 -0.499771 0.107551

ENSMUST00000001790 684 0.1759 0.5578 0.1205 0.3117 0.290 0.218 0.542 44.00 0.743 0.574 214 227 0.561674 0.114537

ENSMUST00000001254 2274 0.2848 0.3560 0.3110 0.2982 0.214 0.036 0.430 56.22 0.512 0.458 739 757 0.361162 0.107001

ENSMUST00000001181 3105 0.2244 0.4501 0.2123 0.3485 0.212 0.062 0.459 46.67 0.642 0.597 1014 1034 -0.647195 0.060928

ENSMUST00000001480 2763 0.4097 0.2938 0.2773 0.2711 0.227 0.005 0.415 55.35 0.443 0.478 880 920 -0.208696 0.094565

ENSMUST00000001339 846 0.3189 0.3243 0.2731 0.3846 0.246 -0.034 0.425 53.72 0.545 0.516 266 281 -1.082.562 0.024911

ENSMUST00000002043 1452 0.3988 0.2560 0.3190 0.3848 0.239 -0.071 0.391 52.71 0.465 0.446 458 483 -0.772257 0.078675

ENSMUST00000001566 1335 0.2365 0.5442 0.1379 0.3488 0.314 0.211 0.548 44.46 0.700 0.565 423 444 -0.347973 0.096847

ENSMUST00000002081 1698 0.2980 0.4018 0.1851 0.3558 0.232 0.049 0.446 50.68 0.603 0.550 547 565 -0.429735 0.072566

ENSMUST00000001279 1785 0.2647 0.4275 0.1942 0.3202 0.189 0.043 0.438 50.70 0.616 0.588 578 594 -0.627441 0.082492

ENSMUST00000001455 1524 0.2162 0.4513 0.1885 0.3350 0.214 0.093 0.460 46.72 0.657 0.601 496 507 -0.557002 0.047337

ENSMUST00000002079 5709 0.2863 0.3912 0.1954 0.3524 0.241 0.085 0.455 49.95 0.601 0.563 1851 1902 -0.122871 0.069401

ENSMUST00000001253 2343 0.3015 0.3318 0.2410 0.3540 0.218 0.041 0.426 55.24 0.544 0.494 759 780 0.327949 0.085897

ENSMUST00000002029 780 0.3099 0.4413 0.1969 0.2935 0.265 0.118 0.492 53.59 0.587 0.516 252 259 -0.571815 0.127413

ENSMUST00000001845 807 0.2727 0.4545 0.1869 0.3115 0.277 0.201 0.547 47.44 0.618 0.557 254 268 -0.195896 0.089552

ENSMUST00000001513 1344 0.1457 0.5829 0.1160 0.4400 0.311 0.171 0.526 40.03 0.792 0.588 424 447 -0.347875 0.096197

ENSMUST00000001626 2001 0.2342 0.3604 0.2518 0.3251 0.181 0.033 0.412 51.57 0.582 0.595 638 666 -0.336336 0.055556

ENSMUST00000001327 2421 0.2533 0.4119 0.2036 0.3814 0.242 0.091 0.474 52.12 0.630 0.581 794 806 -0.270968 0.062035

ENSMUST00000002087 1032 0.2704 0.4667 0.1477 0.3796 0.253 0.064 0.453 48.61 0.662 0.559 331 343 -0.284548 0.087464

ENSMUST00000001715 726 0.2418 0.4066 0.1576 0.4576 0.254 0.101 0.461 45.95 0.680 0.555 228 241 -0.046473 0.087137

ENSMUST00000001559 1332 0.2535 0.3955 0.1799 0.4290 0.278 0.106 0.472 49.26 0.650 0.566 428 443 -0.197065 0.069977

ENSMUST00000001792 3969 0.2637 0.3729 0.2697 0.3083 0.235 0.100 0.480 49.57 0.555 0.542 1299 1322 -0.549924 0.041604

ENSMUST00000001711 708 0.2143 0.4890 0.2299 0.3576 0.233 0.148 0.524 50.18 0.652 0.543 227 235 -0.966808 0.106383

ENSMUST00000001809 1911 0.3946 0.2893 0.3057 0.2633 0.250 0.056 0.455 57.40 0.436 0.493 606 636 -0.511006 0.075472

ENSMUST00000001965 2853 0.1940 0.5137 0.1419 0.3761 0.269 0.187 0.518 44.62 0.720 0.581 923 950 -0.071053 0.091579

ENSMUST00000002011 1185 0.2774 0.3323 0.2149 0.3385 0.202 0.069 0.441 51.31 0.572 0.556 381 394 -0.000762 0.050761

ENSMUST00000001611 2565 0.2769 0.3554 0.2404 0.3864 0.226 0.094 0.471 54.93 0.584 0.523 824 854 -0.531967 0.055035

ENSMUST00000001280 2169 0.1993 0.4897 0.1545 0.3864 0.254 0.169 0.511 47.42 0.708 0.586 701 722 -0.464127 0.074792

ENSMUST00000002007 1626 0.3661 0.3272 0.2391 0.3077 0.231 0.033 0.437 54.18 0.502 0.490 508 541 -0.161368 0.110906

ENSMUST00000001384 894 0.2377 0.4709 0.1751 0.4363 0.306 0.175 0.533 46.74 0.681 0.539 285 297 -0.790236 0.077441

ENSMUST00000001724 1983 0.3158 0.3482 0.2683 0.3607 0.255 0.088 0.468 51.55 0.541 0.480 643 660 -0.499545 0.068182

ENSMUST00000002091 738 0.3314 0.3486 0.2154 0.4086 0.281 0.075 0.456 58.09 0.578 0.480 237 245 -0.216326 0.077551

ENSMUST00000001185 1575 0.2921 0.3515 0.2974 0.3425 0.230 0.095 0.476 55.42 0.532 0.485 500 524 -0.369466 0.099237

ENSMUST00000001548 3162 0.2137 0.4583 0.1549 0.4003 0.247 0.161 0.503 47.70 0.694 0.577 1014 1053 -0.200000 0.081671

ENSMUST00000001950 825 0.2837 0.4375 0.1675 0.3538 0.298 0.196 0.528 48.77 0.630 0.566 254 274 -0.289781 0.069343

ENSMUST00000001989 3177 0.3207 0.3765 0.2218 0.3467 0.262 0.053 0.447 51.61 0.565 0.521 1022 1058 -0.263894 0.087902

ENSMUST00000002063 3369 0.3916 0.2374 0.3397 0.2862 0.218 -0.004 0.408 53.49 0.409 0.443 1089 1122 -0.096613 0.072193

ENSMUST00000002064 888 0.2955 0.3409 0.2627 0.3818 0.236 0.096 0.469 53.66 0.556 0.495 286 295 -0.292203 0.074576

ENSMUST00000002053 1785 0.2080 0.4240 0.1670 0.3763 0.214 0.113 0.469 49.73 0.678 0.626 580 594 -0.271212 0.065657

ENSMUST00000001258 2349 0.2484 0.4490 0.1573 0.4255 0.252 0.050 0.452 48.25 0.679 0.565 756 782 -0.741688 0.078005

ENSMUST00000001484 1593 0.2023 0.4667 0.1621 0.3862 0.224 0.134 0.495 48.53 0.698 0.614 507 530 -0.579811 0.107547

ENSMUST00000001479 2631 0.3200 0.2993 0.2779 0.3824 0.238 0.021 0.425 55.05 0.523 0.493 844 876 -0.097374 0.061644

ENSMUST00000001534 636 0.1098 0.5434 0.0932 0.4968 0.241 0.190 0.524 37.46 0.835 0.679 206 211 -0.723697 0.071090

ENSMUST00000001256 2661 0.1843 0.4394 0.1586 0.3886 0.217 0.103 0.464 45.80 0.705 0.634 860 886 -0.188939 0.075621

ENSMUST00000002025 1884 0.3172 0.3714 0.2758 0.2748 0.247 0.055 0.455 56.58 0.513 0.509 606 627 -0.028868 0.074960

ENSMUST00000001536 1119 0.1024 0.5290 0.1133 0.4576 0.305 0.315 0.613 39.12 0.819 0.685 354 372 -0.498925 0.053763

ENSMUST00000002013 450 0.3232 0.2828 0.4224 0.3333 0.228 -0.053 0.411 61.00 0.445 0.461 146 149 -1.080.537 0.087248

ENSMUST00000001547 4362 0.4848 0.3565 0.1808 0.1187 0.234 0.185 0.529 45.83 0.415 0.630 1433 1453 -0.798968 0.031659

ENSMUST00000001583 1920 0.3512 0.2975 0.3367 0.3092 0.204 -0.008 0.419 57.63 0.462 0.465 626 639 -0.860407 0.061033

ENSMUST00000001184 684 0.1705 0.4318 0.2090 0.4425 0.221 0.154 0.509 45.65 0.695 0.573 220 227 -0.914097 0.030837

ENSMUST00000001757 525 0.3015 0.3529 0.2273 0.4065 0.254 0.123 0.485 54.79 0.580 0.494 169 174 -0.333333 0.080460

ENSMUST00000001415 1461 0.3546 0.3342 0.2064 0.3118 0.245 0.075 0.459 52.00 0.528 0.562 458 486 -0.189506 0.069959

ENSMUST00000001712 6564 0.2378 0.4185 0.1985 0.3971 0.242 0.085 0.465 48.71 0.647 0.563 2116 2187 -0.472748 0.065386

ENSMUST00000001884 2034 0.2268 0.4310 0.2102 0.3931 0.233 0.072 0.457 49.33 0.647 0.557 654 677 -0.480502 0.064993

ENSMUST00000001304 1146 0.1770 0.5246 0.0982 0.4808 0.327 0.241 0.557 40.73 0.779 0.599 366 381 -0.457218 0.078740

ENSMUST00000001709 669 0.2047 0.4854 0.1796 0.3962 0.264 0.207 0.550 51.47 0.692 0.580 211 222 -0.868469 0.085586

ENSMUST00000001801 2505 0.2020 0.4700 0.1301 0.4272 0.276 0.183 0.514 44.03 0.724 0.584 800 834 0.109113 0.105516

ENSMUST00000001872 1020 0.1625 0.4346 0.1805 0.4264 0.268 0.159 0.529 49.08 0.713 0.618 327 339 -0.411209 0.094395

ENSMUST00000001824 2259 0.4075 0.2422 0.3876 0.2288 0.207 -0.062 0.385 52.39 0.364 0.432 725 752 -0.334840 0.121011

ENSMUST00000001419 600 0.3089 0.3496 0.2282 0.5172 0.285 -0.022 0.432 54.09 0.621 0.481 190 199 -1.344.221 0.075377

ENSMUST00000001595 1746 0.2229 0.4785 0.1358 0.4091 0.247 0.159 0.503 45.85 0.703 0.576 563 581 -0.297763 0.087780

ENSMUST00000001818 2073 0.3248 0.3333 0.3594 0.3460 0.228 -0.017 0.418 55.10 0.488 0.455 658 690 -0.857101 0.130435

ENSMUST00000001854 1593 0.1710 0.5325 0.1415 0.3271 0.241 0.179 0.505 45.59 0.729 0.581 505 530 0.596038 0.118868

ENSMUST00000001347 684 0.2826 0.3913 0.1429 0.4201 0.221 0.016 0.427 46.89 0.650 0.573 220 227 -0.489868 0.066079

ENSMUST00000001522 1383 0.1909 0.5242 0.1227 0.4968 0.285 0.193 0.527 44.02 0.759 0.571 444 460 -0.473696 0.080435

ENSMUST00000001538 1029 0.1757 0.4831 0.1491 0.3843 0.242 0.147 0.500 42.59 0.722 0.617 334 342 -0.271053 0.070175

ENSMUST00000001706 783 0.1408 0.5728 0.0785 0.4385 0.243 0.188 0.530 42.44 0.819 0.636 249 260 -0.781154 0.103846

ENSMUST00000001247 1107 0.2941 0.3391 0.3885 0.2773 0.195 -0.044 0.387 57.15 0.467 0.466 362 368 -0.441304 0.067935

ENSMUST00000001780 1443 0.1760 0.5196 0.1232 0.5015 0.277 0.065 0.462 40.99 0.768 0.572 457 480 -0.576458 0.108333

ENSMUST00000001452 1638 0.3477 0.3213 0.2471 0.3464 0.235 0.021 0.428 55.21 0.514 0.490 519 545 -0.259633 0.033028

ENSMUST00000001156 1395 0.3019 0.3684 0.2874 0.2786 0.224 0.017 0.435 54.10 0.522 0.578 437 464 -0.562716 0.064655

ENSMUST00000003717 3831 0.2924 0.3480 0.2997 0.3139 0.216 0.002 0.422 54.05 0.516 0.484 1234 1276 0.035188 0.092476

ENSMUST00000003569 795 0.2010 0.4498 0.1724 0.4508 0.294 0.156 0.508 46.48 0.702 0.567 258 264 -0.165152 0.098485

ENSMUST00000003444 1482 0.2000 0.4939 0.1875 0.4902 0.262 0.111 0.479 48.08 0.710 0.510 476 493 -0.689250 0.060852

ENSMUST00000003529 1608 0.3514 0.4031 0.2313 0.3534 0.260 0.012 0.452 55.21 0.560 0.517 520 535 -1.177.757 0.069159

ENSMUST00000002289 693 0.3509 0.2573 0.4269 0.2767 0.225 -0.110 0.357 54.70 0.398 0.436 221 230 -0.386087 0.082609

ENSMUST00000002099 906 0.3362 0.3966 0.1713 0.4343 0.292 0.034 0.444 53.73 0.608 0.512 293 301 -0.694352 0.079734

ENSMUST00000002599 1644 0.2500 0.3811 0.2176 0.3906 0.241 0.009 0.427 50.73 0.615 0.566 525 547 -0.172578 0.053016

ENSMUST00000002625 852 0.2146 0.4506 0.1455 0.4550 0.268 0.164 0.509 45.30 0.708 0.572 277 283 -0.412368 0.070671

ENSMUST00000004428 2241 0.3307 0.3738 0.2269 0.2874 0.238 0.076 0.453 55.09 0.531 0.496 708 746 0.279759 0.111260

ENSMUST00000002436 1788 0.3056 0.3910 0.2575 0.3455 0.277 0.105 0.495 54.09 0.560 0.511 564 595 -0.640168 0.097479

ENSMUST00000003369 1500 0.3282 0.3511 0.2943 0.3109 0.247 0.055 0.461 58.90 0.512 0.481 486 499 -0.631463 0.068136

ENSMUST00000003659 2268 0.2488 0.4789 0.1466 0.4125 0.284 0.159 0.522 45.28 0.685 0.597 736 755 -0.696159 0.062252

ENSMUST00000003575 747 0.2876 0.3399 0.2727 0.4500 0.340 0.126 0.502 41.46 0.584 0.524 243 248 -1.019.355 0.016129

ENSMUST00000002837 690 0.3240 0.3240 0.3333 0.2968 0.228 0.067 0.454 55.44 0.477 0.450 218 229 -0.161572 0.104803

ENSMUST00000003599 2793 0.2718 0.3756 0.2160 0.3610 0.230 0.117 0.480 52.83 0.595 0.545 916 930 -0.321075 0.060215

ENSMUST00000002400 495 0.2815 0.3704 0.2602 0.3391 0.175 -0.041 0.384 53.54 0.560 0.518 159 164 -0.235975 0.097561

ENSMUST00000002350 1431 0.2005 0.4305 0.2386 0.3994 0.239 0.092 0.472 50.97 0.650 0.556 468 476 -0.374160 0.084034

ENSMUST00000004094 1086 0.3267 0.2600 0.3992 0.1946 0.144 -0.117 0.347 50.98 0.387 0.516 331 361 -0.602493 0.074792

ENSMUST00000003156 507 0.1915 0.4043 0.1831 0.3913 0.246 0.079 0.457 46.11 0.677 0.619 164 168 0.169048 0.047619

ENSMUST00000002640 2148 0.3178 0.3494 0.2707 0.3531 0.264 0.100 0.485 54.26 0.536 0.501 685 715 -0.508811 0.096503

ENSMUST00000002379 783 0.3246 0.3553 0.1777 0.3548 0.228 0.040 0.441 53.41 0.574 0.562 256 260 -0.170385 0.038462

ENSMUST00000003912 1251 0.3368 0.4192 0.2448 0.4120 0.311 -0.020 0.428 53.55 0.580 0.502 400 416 -1.089.423 0.103365

ENSMUST00000003416 1575 0.2682 0.4118 0.1606 0.4197 0.239 0.115 0.470 48.99 0.648 0.542 500 524 -0.137595 0.101145

ENSMUST00000003029 1359 0.2104 0.4207 0.2556 0.4102 0.233 0.046 0.453 53.88 0.632 0.522 435 452 -0.531859 0.075221

ENSMUST00000004145 2382 0.1791 0.5101 0.1000 0.5214 0.321 0.236 0.552 41.23 0.782 0.585 761 793 -0.460530 0.083228

ENSMUST00000003238 1698 0.2088 0.5253 0.2246 0.2822 0.265 0.143 0.510 44.12 0.650 0.589 543 565 -0.902655 0.074336

ENSMUST00000003705 1029 0.2776 0.3042 0.3125 0.3227 0.185 0.031 0.443 56.37 0.512 0.570 334 342 -1.055.848 0.038012

ENSMUST00000002360 1233 0.2500 0.4304 0.1899 0.3869 0.237 0.148 0.506 52.55 0.646 0.576 393 410 -0.614634 0.073171

ENSMUST00000003370 1575 0.2136 0.4447 0.2015 0.4255 0.260 0.099 0.481 43.76 0.669 0.550 499 524 -0.493511 0.097328

ENSMUST00000002292 1176 0.3344 0.3046 0.3495 0.3120 0.215 0.013 0.422 57.17 0.464 0.463 377 391 -0.175959 0.071611

ENSMUST00000002198 2376 0.2958 0.3838 0.2243 0.3828 0.227 -0.048 0.380 51.91 0.589 0.548 764 791 -0.719469 0.058154

ENSMUST00000002176 1455 0.3483 0.3245 0.2640 0.3070 0.258 0.101 0.490 55.40 0.501 0.506 463 484 -0.225620 0.064050

ENSMUST00000002765 2757 0.3212 0.3522 0.2504 0.3318 0.238 0.068 0.453 56.10 0.535 0.505 886 918 -0.273312 0.071895

ENSMUST00000003620 1800 0.2284 0.3922 0.1616 0.4773 0.254 0.101 0.474 46.69 0.685 0.575 572 599 -0.358097 0.088481

ENSMUST00000004137 657 0.2381 0.4405 0.1575 0.4963 0.280 0.124 0.490 43.98 0.691 0.526 204 218 -0.446789 0.137615

ENSMUST00000004057 468 0.2586 0.3362 0.2520 0.4138 0.216 0.036 0.439 56.05 0.588 0.501 148 155 -0.528387 0.070968

ENSMUST00000003310 9162 0.4807 0.1856 0.4176 0.2138 0.222 -0.085 0.384 48.43 0.302 0.408 2970 3053 -0.620210 0.086472

ENSMUST00000004281 1800 0.2080 0.4601 0.1620 0.4197 0.211 0.105 0.477 50.32 0.698 0.565 576 599 -0.494324 0.065109

ENSMUST00000003183 1500 0.2120 0.5236 0.1493 0.4428 0.284 0.098 0.484 44.06 0.721 0.552 481 499 -0.506814 0.104208

ENSMUST00000003554 1218 0.2754 0.4131 0.2305 0.4466 0.281 0.007 0.426 51.54 0.620 0.513 392 405 -0.527161 0.098765

ENSMUST00000002757 321 0.4615 0.1667 0.3218 0.3125 0.177 -0.027 0.382 52.36 0.373 0.425 102 106 -0.564151 0.075472

ENSMUST00000002677 2667 0.2423 0.3955 0.2153 0.3613 0.201 0.033 0.422 49.69 0.619 0.580 848 888 -0.277703 0.074324

ENSMUST00000002825 4440 0.3234 0.3100 0.2916 0.3857 0.220 -0.015 0.411 54.37 0.527 0.490 1432 1479 -0.916362 0.058824

ENSMUST00000002655 348 0.2111 0.3778 0.1875 0.4773 0.192 -0.085 0.363 52.52 0.673 0.583 113 115 -0.104348 0.069565

ENSMUST00000002172 1272 0.3123 0.3664 0.2083 0.3838 0.218 0.062 0.442 55.40 0.583 0.528 405 423 -0.246809 0.104019

ENSMUST00000003961 3585 0.2132 0.3912 0.1794 0.4471 0.214 0.070 0.453 47.69 0.680 0.598 1147 1194 -0.745645 0.036013

ENSMUST00000003049 2238 0.2211 0.4158 0.1675 0.3824 0.232 0.130 0.475 47.59 0.669 0.630 726 745 -0.196779 0.052349

ENSMUST00000002964 2175 0.2003 0.4805 0.1992 0.3737 0.284 0.172 0.515 47.71 0.677 0.534 691 724 0.122928 0.096685

ENSMUST00000002275 1362 0.2283 0.4375 0.1890 0.3899 0.233 0.087 0.463 45.79 0.662 0.555 441 453 -0.505519 0.081678

ENSMUST00000004072 774 0.2642 0.4340 0.1429 0.3866 0.245 0.121 0.490 44.67 0.660 0.588 253 257 -0.527626 0.054475

ENSMUST00000003574 1575 0.2402 0.4388 0.1546 0.4028 0.259 0.193 0.515 48.65 0.671 0.564 499 524 -0.079008 0.097328

ENSMUST00000003754 816 0.2674 0.4278 0.1753 0.5054 0.259 0.029 0.447 47.44 0.673 0.502 257 271 -0.655351 0.110701

ENSMUST00000003038 2817 0.2500 0.4136 0.1882 0.4071 0.267 0.129 0.486 51.69 0.643 0.530 918 938 -0.093177 0.074627

ENSMUST00000004208 1482 0.2216 0.4617 0.1695 0.4405 0.281 0.154 0.507 45.04 0.692 0.558 467 493 -0.695943 0.103448

ENSMUST00000002529 2826 0.3214 0.3000 0.2670 0.3824 0.221 -0.015 0.410 52.80 0.530 0.522 896 941 -0.616047 0.065887

ENSMUST00000003720 1839 0.4025 0.2331 0.4022 0.2459 0.199 -0.054 0.384 53.45 0.367 0.438 583 612 -0.363072 0.101307

ENSMUST00000003451 699 0.2174 0.4239 0.2308 0.3721 0.232 0.053 0.442 52.12 0.634 0.533 224 232 -0.205172 0.068966

ENSMUST00000003642 1860 0.2137 0.3910 0.1499 0.5030 0.254 0.108 0.483 40.88 0.710 0.603 607 619 -0.696931 0.045234

ENSMUST00000002881 669 0.2346 0.4134 0.1930 0.3926 0.256 0.105 0.474 52.46 0.648 0.580 213 222 -0.105405 0.085586

ENSMUST00000003284 1260 0.2086 0.4018 0.1911 0.4525 0.239 0.122 0.481 47.45 0.678 0.582 397 419 -0.395465 0.095465

ENSMUST00000002855 639 0.2077 0.5246 0.1293 0.3817 0.320 0.248 0.552 49.81 0.719 0.508 203 212 0.691509 0.183962

ENSMUST00000003345 2061 0.3366 0.3033 0.2714 0.3517 0.253 -0.013 0.416 54.16 0.516 0.533 670 686 -0.636589 0.061224

ENSMUST00000002790 2916 0.4099 0.2598 0.3375 0.2842 0.224 -0.017 0.400 53.79 0.408 0.416 942 971 -0.035839 0.094748

ENSMUST00000002808 942 0.3185 0.3427 0.2776 0.3304 0.228 0.026 0.441 57.10 0.520 0.488 306 313 -0.422045 0.057508

ENSMUST00000002284 984 0.1971 0.5018 0.1777 0.3333 0.255 0.182 0.524 45.29 0.691 0.596 317 327 -0.188073 0.055046

ENSMUST00000004156 2553 0.2049 0.3997 0.2176 0.3584 0.197 0.089 0.456 48.14 0.638 0.624 815 850 -0.598000 0.063529

ENSMUST00000003971 624 0.2160 0.3827 0.1193 0.5000 0.278 0.174 0.512 40.73 0.724 0.625 203 207 -0.435749 0.043478

ENSMUST00000003964 2217 0.2209 0.4668 0.1769 0.3918 0.272 0.132 0.496 47.89 0.679 0.565 710 738 -0.418970 0.113821

ENSMUST00000002101 549 0.3099 0.3521 0.3000 0.3256 0.221 0.069 0.461 58.56 0.517 0.476 178 182 -0.351648 0.076923

ENSMUST00000004140 657 0.2182 0.5152 0.1655 0.4394 0.273 0.101 0.478 42.59 0.704 0.518 203 218 -0.532569 0.137615

ENSMUST00000002457 1200 0.2351 0.4435 0.1843 0.3646 0.244 0.187 0.514 52.69 0.653 0.582 383 399 -0.336090 0.072682

ENSMUST00000003501 1104 0.1722 0.5563 0.1158 0.3902 0.273 0.186 0.531 41.92 0.761 0.586 356 367 -0.173025 0.081744

ENSMUST00000002310 1035 0.4409 0.2509 0.3265 0.1620 0.210 -0.019 0.379 49.58 0.346 0.513 335 344 -0.180814 0.069767

ENSMUST00000002678 1173 0.1531 0.4938 0.1809 0.4203 0.245 0.209 0.527 46.40 0.729 0.585 376 390 -0.411539 0.082051

ENSMUST00000003115 594 0.2162 0.3986 0.1854 0.4733 0.191 0.046 0.453 55.05 0.684 0.587 190 197 -1.254.822 0.045685

ENSMUST00000002397 1047 0.3659 0.3261 0.2727 0.2975 0.237 -0.016 0.421 54.73 0.484 0.487 335 348 -0.139081 0.080460

ENSMUST00000002925 858 0.3142 0.3186 0.2620 0.3255 0.191 0.012 0.426 57.64 0.518 0.539 272 285 -0.358947 0.101754

ENSMUST00000002518 594 0.1806 0.4722 0.1226 0.5270 0.308 0.216 0.545 48.34 0.764 0.587 191 197 -0.567513 0.045685

ENSMUST00000002976 2595 0.2168 0.4602 0.1817 0.3961 0.244 0.146 0.487 48.23 0.677 0.569 824 864 -0.217361 0.077546

ENSMUST00000004143 2361 0.1824 0.4854 0.1354 0.5145 0.307 0.195 0.531 43.53 0.752 0.570 751 786 -0.482061 0.085242

ENSMUST00000003207 2409 0.1855 0.4303 0.2292 0.3512 0.228 0.110 0.471 47.96 0.651 0.588 777 802 -0.155362 0.073566

ENSMUST00000003312 1347 0.2325 0.4454 0.2059 0.3665 0.262 0.082 0.472 48.94 0.644 0.558 430 448 -0.281920 0.062500

ENSMUST00000004326 5619 0.2802 0.4223 0.1615 0.3699 0.255 0.098 0.468 48.20 0.634 0.553 1824 1872 -0.153846 0.078526

ENSMUST00000003628 2736 0.2155 0.4468 0.1317 0.4109 0.203 0.077 0.446 45.24 0.706 0.611 867 911 -0.251262 0.087816

ENSMUST00000004379 735 0.2806 0.3622 0.2228 0.3626 0.213 0.037 0.426 52.94 0.583 0.527 235 244 -0.104098 0.061475

ENSMUST00000003137 1473 0.3460 0.3359 0.3221 0.2773 0.208 -0.004 0.407 52.81 0.468 0.444 474 490 -0.120204 0.100000

ENSMUST00000003527 3249 0.2660 0.4365 0.1902 0.3906 0.266 0.089 0.476 48.15 0.638 0.555 1047 1082 -0.741035 0.072089

ENSMUST00000002809 657 0.3457 0.3333 0.4091 0.2676 0.227 0.024 0.445 57.64 0.436 0.437 211 218 -0.662385 0.096330

ENSMUST00000002883 1056 0.2519 0.4407 0.2443 0.3375 0.246 0.127 0.482 47.64 0.602 0.523 332 351 -0.414815 0.056980

ENSMUST00000003438 654 0.1404 0.5731 0.1565 0.4526 0.335 0.264 0.570 41.99 0.773 0.562 207 217 -0.430876 0.129032

ENSMUST00000003843 1968 0.2763 0.3677 0.2495 0.3704 0.214 0.035 0.438 57.01 0.576 0.526 628 655 -0.297252 0.106870

ENSMUST00000002805 567 0.3576 0.3642 0.3043 0.2231 0.213 0.018 0.428 60.64 0.467 0.466 180 188 -0.260106 0.127660

ENSMUST00000002735 819 0.1818 0.4455 0.1697 0.4171 0.241 0.166 0.504 48.91 0.702 0.589 258 272 -0.076103 0.055147

ENSMUST00000003268 1107 0.1842 0.4436 0.1517 0.5288 0.258 0.053 0.447 43.28 0.740 0.576 358 368 -0.690761 0.062500

ENSMUST00000002845 525 0.2698 0.3095 0.3403 0.3165 0.221 -0.045 0.398 50.30 0.500 0.580 166 174 -0.731609 0.040230

ENSMUST00000002502 3012 0.4302 0.1982 0.3963 0.2553 0.193 -0.079 0.375 50.28 0.344 0.408 963 1003 -0.460718 0.071785

ENSMUST00000003274 687 0.1907 0.5206 0.1749 0.2898 0.207 0.141 0.489 43.48 0.688 0.614 221 228 -0.295176 0.092105

ENSMUST00000004378 1305 0.2550 0.4384 0.1511 0.4243 0.257 0.064 0.458 50.64 0.670 0.558 421 434 -0.195622 0.071429

ENSMUST00000002737 666 0.2376 0.3536 0.1977 0.4201 0.213 0.109 0.465 49.11 0.634 0.597 213 221 -0.407240 0.072398

ENSMUST00000004343 1083 0.2500 0.3841 0.2371 0.3838 0.238 0.057 0.443 55.20 0.603 0.520 348 360 -0.130833 0.063889

ENSMUST00000003621 1443 0.2486 0.3852 0.2247 0.3731 0.202 0.008 0.426 52.33 0.613 0.580 470 480 -0.768333 0.062500

ENSMUST00000003319 2130 0.2834 0.3556 0.2190 0.4053 0.236 0.112 0.477 53.90 0.596 0.534 690 709 -0.391678 0.070522

ENSMUST00000003413 1599 0.2178 0.4473 0.1788 0.4290 0.274 0.169 0.503 46.59 0.680 0.546 507 532 -0.160526 0.112782

ENSMUST00000004396 360 0.2041 0.4898 0.1573 0.4474 0.285 0.204 0.534 46.07 0.707 0.546 116 119 -0.257983 0.058824

ENSMUST00000002127 723 0.1684 0.4847 0.1117 0.5210 0.221 0.121 0.489 49.04 0.774 0.599 235 240 -0.641250 0.104167

ENSMUST00000003450 2460 0.2730 0.3771 0.2133 0.4477 0.231 0.049 0.451 49.08 0.622 0.542 796 819 -1.138.950 0.052503

ENSMUST00000002469 660 0.1954 0.4138 0.1620 0.4602 0.184 0.036 0.426 48.22 0.708 0.638 216 219 -1.128.311 0.059361

ENSMUST00000002926 1371 0.2715 0.4047 0.2145 0.3438 0.228 0.053 0.440 54.44 0.598 0.531 443 456 0.070614 0.092105

ENSMUST00000003313 1773 0.2701 0.4085 0.2587 0.3842 0.250 0.098 0.477 52.02 0.593 0.502 572 590 -0.597627 0.094915

ENSMUST00000003035 4566 0.1877 0.4980 0.1872 0.3833 0.255 0.124 0.492 46.55 0.699 0.556 1460 1521 -0.152663 0.100592

ENSMUST00000003017 1602 0.2419 0.3825 0.2402 0.3740 0.211 0.056 0.433 50.72 0.604 0.532 513 533 -0.054972 0.090056

ENSMUST00000003121 624 0.2062 0.4375 0.1933 0.5115 0.234 0.030 0.452 44.21 0.688 0.501 199 207 -0.361836 0.101449

ENSMUST00000002495 1137 0.1961 0.4373 0.2171 0.4060 0.213 0.035 0.440 48.37 0.667 0.578 366 378 -0.607407 0.066138

ENSMUST00000003469 663 0.1989 0.4365 0.2547 0.3642 0.224 0.113 0.469 45.23 0.635 0.548 211 220 -0.272273 0.090909

ENSMUST00000003645 897 0.1814 0.4262 0.2172 0.4068 0.221 0.064 0.444 46.68 0.672 0.582 293 298 -0.339597 0.070470

ENSMUST00000003910 1062 0.2361 0.4340 0.2405 0.3203 0.253 0.130 0.497 54.84 0.612 0.557 338 353 -0.298584 0.121813

ENSMUST00000003152 1311 0.1746 0.5118 0.1201 0.4870 0.275 0.167 0.509 43.08 0.765 0.583 422 436 -0.449541 0.073394

ENSMUST00000002839 1785 0.2403 0.4554 0.1776 0.4460 0.222 -0.015 0.402 50.48 0.677 0.527 575 594 -0.501852 0.097643

ENSMUST00000002663 1068 0.3612 0.2709 0.3320 0.3038 0.210 -0.043 0.383 61.00 0.441 0.438 347 355 -0.001409 0.104225

ENSMUST00000003459 3075 0.2382 0.4301 0.1770 0.4176 0.233 0.093 0.465 45.90 0.664 0.564 991 1024 -0.418457 0.087891

ENSMUST00000003493 1566 0.2391 0.4511 0.2122 0.4125 0.268 0.058 0.462 49.99 0.654 0.575 506 521 -0.925336 0.067179

ENSMUST00000003857 2115 0.2542 0.4103 0.1687 0.3413 0.219 0.084 0.456 50.32 0.635 0.592 682 704 -0.202415 0.071023

ENSMUST00000003826 1470 0.2063 0.4466 0.1589 0.4255 0.219 0.139 0.475 45.18 0.694 0.549 467 489 0.096115 0.108384

ENSMUST00000002923 1089 0.2160 0.4599 0.2016 0.3714 0.210 0.052 0.457 49.61 0.662 0.576 337 362 -0.311326 0.113260

ENSMUST00000002413 1443 0.1559 0.4480 0.1365 0.4484 0.275 0.234 0.527 43.73 0.751 0.610 461 480 0.451875 0.110417

ENSMUST00000003714 3258 0.3932 0.2969 0.3820 0.2367 0.220 -0.055 0.399 54.96 0.403 0.428 1038 1085 -0.450046 0.128111

ENSMUST00000002327 1893 0.1655 0.4775 0.1143 0.5478 0.280 0.165 0.513 41.17 0.787 0.612 606 630 -0.886984 0.058730

ENSMUST00000003643 1146 0.1633 0.5782 0.0803 0.4846 0.372 0.257 0.568 35.10 0.809 0.590 366 381 -0.595801 0.078740

ENSMUST00000003850 2037 0.2556 0.3993 0.1908 0.3933 0.219 0.062 0.457 50.57 0.637 0.585 652 678 -0.712389 0.060472

ENSMUST00000003290 768 0.2047 0.4093 0.1946 0.3470 0.203 0.102 0.450 48.62 0.653 0.629 251 255 -0.257647 0.047059

ENSMUST00000003860 1602 0.2372 0.4047 0.1749 0.4074 0.211 0.078 0.454 48.18 0.658 0.595 515 533 -0.190432 0.076923

ENSMUST00000002280 1563 0.1854 0.4610 0.1760 0.4051 0.229 0.105 0.471 45.94 0.701 0.596 495 520 -0.496731 0.071154

ENSMUST00000003434 1572 0.1734 0.5000 0.0794 0.4714 0.285 0.179 0.521 39.66 0.787 0.630 512 523 -0.123518 0.082218

ENSMUST00000002710 1095 0.3275 0.3415 0.2014 0.3686 0.245 0.062 0.453 57.54 0.570 0.560 349 364 -0.292582 0.087912

ENSMUST00000002708 1314 0.1192 0.4878 0.1345 0.4740 0.264 0.218 0.541 40.39 0.788 0.638 425 437 -0.224256 0.077803

ENSMUST00000003513 906 0.1345 0.4496 0.1694 0.4871 0.244 0.112 0.481 43.44 0.751 0.615 293 301 -0.497342 0.049834

ENSMUST00000002145 1017 0.2602 0.3618 0.2741 0.3760 0.232 0.097 0.466 59.74 0.574 0.499 324 338 -0.172485 0.053254

ENSMUST00000003726 4206 0.2724 0.3618 0.2581 0.3726 0.220 0.004 0.411 52.60 0.577 0.562 1359 1401 -1.065.525 0.034261

ENSMUST00000002880 1620 0.2177 0.4580 0.1527 0.4015 0.247 0.114 0.480 48.13 0.694 0.585 523 539 -0.048609 0.089054

ENSMUST00000004054 1617 0.4063 0.2506 0.3398 0.2865 0.219 -0.007 0.413 48.47 0.407 0.445 518 538 -0.246283 0.068773

ENSMUST00000003710 2637 0.3468 0.3512 0.2583 0.2861 0.239 0.088 0.469 54.15 0.507 0.520 866 878 -0.470615 0.051253

ENSMUST00000002588 4776 0.2153 0.4546 0.1844 0.4096 0.237 0.090 0.477 49.36 0.679 0.560 1550 1591 -0.603897 0.068510

ENSMUST00000002318 1707 0.2058 0.4712 0.1806 0.3982 0.304 0.152 0.521 44.14 0.689 0.596 562 568 -0.506338 0.054577

ENSMUST00000002551 360 0.3505 0.1753 0.4200 0.2316 0.125 -0.111 0.330 45.93 0.339 0.445 115 119 -0.579832 0.025210

ENSMUST00000003027 1407 0.2403 0.4530 0.1685 0.4012 0.247 0.087 0.463 48.73 0.668 0.558 449 468 -0.467308 0.064103

ENSMUST00000003681 1212 0.2248 0.4631 0.2100 0.4164 0.253 0.085 0.465 45.26 0.662 0.533 385 403 -0.479405 0.099256

ENSMUST00000002914 2736 0.2432 0.3769 0.2190 0.4433 0.226 -0.003 0.419 49.69 0.636 0.558 887 911 -0.824040 0.041712

ENSMUST00000004294 2379 0.2184 0.3513 0.2253 0.3858 0.200 0.103 0.466 51.10 0.622 0.609 775 792 -0.413763 0.037879

ENSMUST00000003071 375 0.1584 0.4356 0.1979 0.3750 0.222 0.259 0.548 49.60 0.696 0.591 115 124 -0.359677 0.088710

ENSMUST00000003677 1140 0.2138 0.3553 0.1683 0.4651 0.226 0.129 0.468 50.92 0.679 0.607 365 379 -0.114248 0.052770

ENSMUST00000003117 1272 0.2284 0.4969 0.1352 0.4379 0.260 0.044 0.440 45.33 0.711 0.530 405 423 -0.277541 0.104019

ENSMUST00000002572 1014 0.2052 0.4366 0.1550 0.4394 0.210 0.039 0.436 45.21 0.706 0.630 330 337 -0.863798 0.041543

ENSMUST00000002699 2064 0.3075 0.3660 0.2868 0.3239 0.215 -0.017 0.429 53.90 0.532 0.528 665 687 -0.940320 0.077147

ENSMUST00000004375 900 0.2112 0.4267 0.1633 0.4522 0.237 0.118 0.481 44.19 0.693 0.570 293 299 -0.257525 0.063545

ENSMUST00000002403 942 0.2946 0.3721 0.2259 0.3231 0.247 0.047 0.437 55.22 0.567 0.539 300 313 -0.010224 0.079872

ENSMUST00000003386 885 0.2163 0.4408 0.1489 0.3822 0.192 0.081 0.443 51.14 0.688 0.600 282 294 -0.243878 0.081633

ENSMUST00000003907 1344 0.2888 0.3651 0.1715 0.3981 0.209 0.061 0.448 52.53 0.613 0.549 426 447 -0.207830 0.071588

ENSMUST00000004232 1128 0.2788 0.4231 0.2517 0.2667 0.266 0.109 0.479 54.11 0.559 0.523 365 375 0.202667 0.061333

ENSMUST00000003318 2151 0.2043 0.4992 0.1664 0.3307 0.240 0.124 0.496 45.77 0.688 0.597 686 716 -0.643575 0.071229

ENSMUST00000003818 678 0.3229 0.2969 0.3353 0.2614 0.233 0.035 0.428 56.79 0.451 0.471 215 225 0.410667 0.128889

ENSMUST00000003509 1197 0.2516 0.4151 0.3021 0.3037 0.237 0.083 0.467 53.52 0.562 0.482 381 398 -0.325879 0.105528

ENSMUST00000003597 1431 0.2962 0.3494 0.3251 0.2522 0.195 0.016 0.419 53.83 0.487 0.473 458 476 -0.089916 0.096639

ENSMUST00000003759 1020 0.3419 0.4118 0.1694 0.3219 0.305 0.114 0.495 54.66 0.583 0.559 321 339 -0.394100 0.094395

ENSMUST00000002274 1260 0.2672 0.4022 0.1610 0.3742 0.206 0.091 0.458 52.53 0.635 0.560 408 419 0.089260 0.105012

ENSMUST00000002445 1476 0.2434 0.3968 0.2088 0.3844 0.249 0.032 0.452 49.27 0.631 0.582 480 491 -0.720978 0.038697

ENSMUST00000004050 717 0.2843 0.4020 0.2763 0.2868 0.211 -0.039 0.394 53.62 0.548 0.493 221 238 0.367227 0.176471

ENSMUST00000003219 1518 0.2160 0.4053 0.2132 0.3789 0.232 0.176 0.509 51.94 0.639 0.567 487 505 -0.130495 0.065347

ENSMUST00000002889 3816 0.2004 0.4714 0.1794 0.4367 0.282 0.150 0.500 45.49 0.700 0.560 1225 1271 -0.426436 0.086546

ENSMUST00000004076 2640 0.2747 0.4176 0.2671 0.3023 0.239 0.084 0.467 53.59 0.564 0.484 844 879 0.011832 0.111490

ENSMUST00000003442 1455 0.2063 0.4656 0.1618 0.4330 0.246 0.137 0.496 46.06 0.704 0.589 470 484 -0.731612 0.064050

ENSMUST00000003360 987 0.2318 0.4983 0.1417 0.3004 0.203 0.164 0.497 45.35 0.678 0.602 320 328 -0.235061 0.088415

ENSMUST00000003461 3072 0.2899 0.3980 0.2068 0.3878 0.263 0.081 0.464 50.08 0.603 0.526 981 1023 -0.346921 0.094819

ENSMUST00000002456 1722 0.3363 0.3274 0.3041 0.2976 0.220 0.035 0.444 58.19 0.487 0.497 556 573 -0.811169 0.080279

ENSMUST00000003741 2175 0.2134 0.4727 0.1387 0.4502 0.255 0.081 0.461 47.01 0.718 0.562 701 724 -0.365470 0.084254

ENSMUST00000003536 600 0.2903 0.3935 0.1656 0.4013 0.327 0.181 0.526 44.89 0.633 0.578 196 199 -0.344724 0.035176

ENSMUST00000003687 1239 0.1840 0.4816 0.1869 0.4371 0.236 0.140 0.494 46.81 0.707 0.549 399 412 -0.483738 0.072816

ENSMUST00000004206 963 0.2387 0.4609 0.1554 0.4322 0.229 0.011 0.434 50.03 0.688 0.565 311 320 -0.819062 0.065625

ENSMUST00000003044 1569 0.2222 0.3759 0.2095 0.4134 0.232 0.066 0.445 50.63 0.642 0.579 508 522 -0.278736 0.080460

ENSMUST00000002846 882 0.1700 0.4696 0.1635 0.4545 0.287 0.165 0.514 47.29 0.730 0.578 282 293 -0.249488 0.102389

ENSMUST00000002395 1776 0.2562 0.3537 0.2421 0.3642 0.177 0.003 0.387 52.77 0.585 0.569 569 591 -0.441624 0.055838

ENSMUST00000002532 651 0.1890 0.4878 0.2609 0.3312 0.230 0.066 0.459 54.94 0.644 0.576 205 216 -0.737500 0.078704

ENSMUST00000004222 3048 0.3144 0.3403 0.2250 0.3447 0.230 0.033 0.433 53.95 0.553 0.521 971 1015 -0.260197 0.059113

ENSMUST00000003550 2127 0.2898 0.3983 0.2135 0.3248 0.240 0.098 0.472 53.41 0.583 0.523 684 708 -0.132910 0.097458

ENSMUST00000003154 630 0.1530 0.5355 0.0987 0.4218 0.308 0.335 0.601 36.52 0.788 0.636 203 209 -0.293780 0.124402

ENSMUST00000003572 2190 0.3028 0.3468 0.2667 0.3619 0.232 0.017 0.427 55.27 0.547 0.504 707 729 -0.313855 0.085048

ENSMUST00000003640 1017 0.1913 0.4657 0.2103 0.3112 0.229 0.136 0.494 45.69 0.657 0.622 332 338 -0.581065 0.059172

ENSMUST00000003521 477 0.2881 0.4492 0.1393 0.4545 0.256 0.122 0.494 48.17 0.669 0.506 154 158 -0.606962 0.094937

ENSMUST00000002979 5505 0.3075 0.4014 0.2510 0.3441 0.279 0.115 0.496 54.04 0.566 0.525 1793 1834 -0.451145 0.069793

ENSMUST00000004201 5220 0.2940 0.3092 0.2554 0.3045 0.175 0.002 0.415 52.65 0.524 0.626 1715 1739 -0.671075 0.037378

ENSMUST00000003635 483 0.2391 0.4493 0.1053 0.3810 0.209 0.170 0.497 47.62 0.701 0.619 157 160 -0.224375 0.056250

ENSMUST00000002298 1524 0.2464 0.4052 0.1746 0.3808 0.207 0.068 0.449 49.79 0.646 0.592 492 507 -0.414004 0.065089

ENSMUST00000002473 1002 0.2258 0.4516 0.1846 0.4160 0.279 0.048 0.453 45.69 0.675 0.573 320 333 -0.456156 0.069069

ENSMUST00000003397 1272 0.2500 0.4634 0.1223 0.4482 0.281 0.100 0.468 43.77 0.701 0.532 408 423 -0.153428 0.104019

ENSMUST00000003445 606 0.2236 0.3230 0.2312 0.4313 0.197 0.050 0.416 48.59 0.614 0.542 197 201 0.025871 0.044776

ENSMUST00000004173 627 0.2431 0.3923 0.2968 0.2653 0.222 -0.028 0.385 51.26 0.550 0.580 200 208 -0.085577 0.091346

ENSMUST00000002850 4497 0.1905 0.4325 0.1480 0.4232 0.230 0.129 0.476 44.68 0.712 0.598 1433 1498 0.201469 0.086115

ENSMUST00000003906 1527 0.2306 0.4301 0.1525 0.4476 0.270 0.118 0.483 45.20 0.692 0.570 487 508 -0.395669 0.088583

ENSMUST00000002128 780 0.2573 0.4806 0.1546 0.3901 0.264 0.095 0.474 45.42 0.672 0.539 253 259 -0.122780 0.088803

ENSMUST00000004036 1023 0.2483 0.3537 0.2059 0.3636 0.155 0.001 0.407 49.18 0.608 0.624 329 340 -0.378529 0.076471

ENSMUST00000002902 1212 0.2090 0.4269 0.1645 0.4152 0.239 0.123 0.483 46.99 0.687 0.584 383 403 -0.043424 0.066998

ENSMUST00000004134 675 0.2970 0.4303 0.2222 0.4318 0.353 0.221 0.550 50.81 0.612 0.475 209 224 -0.377232 0.133929

ENSMUST00000002740 471 0.3106 0.3485 0.1818 0.3445 0.216 0.035 0.427 44.61 0.580 0.600 150 156 -0.101923 0.064103

ENSMUST00000004327 1548 0.2786 0.4428 0.2038 0.3765 0.254 0.043 0.444 47.97 0.621 0.524 493 515 -0.425049 0.108738

ENSMUST00000002487 2415 0.3344 0.3078 0.3376 0.2707 0.206 -0.038 0.398 54.83 0.455 0.488 778 804 -0.427736 0.079602

ENSMUST00000003203 3777 0.3584 0.2595 0.3327 0.2877 0.195 -0.024 0.402 54.88 0.437 0.485 1231 1258 -0.959141 0.043720

ENSMUST00000004203 1413 0.1699 0.4540 0.1789 0.4523 0.228 0.097 0.472 47.12 0.718 0.599 458 470 -0.809149 0.044681

ENSMUST00000003946 1212 0.2233 0.4057 0.1780 0.4742 0.239 0.109 0.478 45.83 0.679 0.561 393 403 -0.532258 0.066998

ENSMUST00000002121 5181 0.3098 0.4083 0.2467 0.3750 0.254 0.033 0.449 50.80 0.577 0.516 1670 1726 -0.861182 0.087486

ENSMUST00000002444 2154 0.1696 0.5156 0.1447 0.3992 0.281 0.174 0.516 43.32 0.741 0.606 688 717 -0.313389 0.073919

ENSMUST00000004381 1464 0.2094 0.4901 0.2090 0.3409 0.260 0.209 0.532 51.55 0.662 0.512 459 487 0.304928 0.166324

ENSMUST00000003568 1293 0.1648 0.5385 0.1338 0.4238 0.263 0.177 0.510 43.68 0.756 0.553 410 430 0.271628 0.148837

ENSMUST00000002603 4998 0.2660 0.3551 0.1770 0.4009 0.214 0.062 0.438 47.97 0.628 0.605 1637 1665 -0.444444 0.034234

ENSMUST00000002924 1461 0.3066 0.3797 0.2232 0.3115 0.214 0.060 0.432 57.97 0.558 0.495 468 486 0.238066 0.135802

ENSMUST00000002368 1989 0.4085 0.2254 0.3868 0.2779 0.208 -0.053 0.394 50.80 0.382 0.433 652 662 -0.737311 0.069486

ENSMUST00000002095 11892 0.3127 0.3432 0.2686 0.3065 0.208 0.009 0.427 54.71 0.522 0.525 3881 3963 -0.727025 0.043401

ENSMUST00000002152 582 0.1258 0.5409 0.1463 0.3313 0.155 0.044 0.418 41.65 0.761 0.722 184 193 -0.655440 0.041451

ENSMUST00000003512 1098 0.2749 0.3540 0.1873 0.3813 0.232 0.088 0.456 51.22 0.611 0.572 342 365 -0.229041 0.093151

ENSMUST00000003911 1089 0.2679 0.3849 0.1954 0.4240 0.270 0.105 0.480 53.18 0.631 0.556 352 362 -0.512707 0.052486

ENSMUST00000002100 1098 0.2724 0.3846 0.1960 0.3162 0.211 0.106 0.461 49.21 0.596 0.575 356 365 -0.054521 0.049315

ENSMUST00000004097 4281 0.2131 0.4184 0.2494 0.3616 0.241 0.073 0.463 50.48 0.620 0.566 1391 1426 -0.733520 0.037868

ENSMUST00000004172 948 0.2696 0.3652 0.2704 0.4159 0.247 -0.027 0.416 54.97 0.587 0.508 303 315 -0.515556 0.101587

ENSMUST00000002533 720 0.2703 0.4270 0.3182 0.2470 0.227 0.115 0.496 53.72 0.531 0.523 226 239 -0.603766 0.066946

ENSMUST00000004133 2352 0.2308 0.4553 0.1784 0.3827 0.239 0.163 0.505 49.72 0.667 0.550 753 783 -0.320051 0.095785

ENSMUST00000003468 2940 0.1420 0.5383 0.1012 0.4350 0.260 0.218 0.535 40.69 0.795 0.617 936 979 -0.129520 0.081716

ENSMUST00000003870 888 0.1504 0.4690 0.2075 0.4304 0.221 0.159 0.516 49.82 0.709 0.588 289 295 -0.877288 0.033898

ENSMUST00000004330 1293 0.3015 0.3382 0.1623 0.5463 0.249 0.042 0.446 48.87 0.666 0.550 413 430 -0.918837 0.039535

ENSMUST00000002389 2448 0.2661 0.4254 0.2066 0.3212 0.232 0.069 0.459 51.59 0.608 0.563 785 815 -0.444049 0.084663

ENSMUST00000002840 2946 0.1962 0.3522 0.1692 0.4367 0.182 0.049 0.418 48.95 0.681 0.634 965 981 0.013252 0.041794

ENSMUST00000002180 819 0.1809 0.5427 0.1340 0.4555 0.331 0.240 0.564 42.55 0.759 0.585 257 272 -0.888235 0.099265

ENSMUST00000002885 675 0.1930 0.4386 0.1850 0.4417 0.262 0.183 0.519 47.94 0.693 0.577 212 224 -0.374554 0.102679

ENSMUST00000004392 1494 0.2296 0.4591 0.1907 0.3827 0.220 0.073 0.442 50.01 0.662 0.582 477 497 -0.498189 0.086519

ENSMUST00000004051 888 0.1747 0.4978 0.1826 0.4083 0.229 0.145 0.491 50.97 0.712 0.581 285 295 -0.823051 0.057627

ENSMUST00000002418 363 0.1860 0.5116 0.1753 0.4043 0.299 0.175 0.522 38.98 0.713 0.575 115 120 -0.559167 0.066667

ENSMUST00000003561 993 0.1852 0.5963 0.0609 0.4372 0.341 0.264 0.577 37.21 0.799 0.582 319 330 -0.382121 0.109091

ENSMUST00000002177 1338 0.1958 0.4688 0.1989 0.4012 0.231 0.070 0.451 48.23 0.682 0.574 428 445 -0.416629 0.065169

ENSMUST00000002305 2823 0.3764 0.2624 0.3727 0.2751 0.214 -0.019 0.413 54.11 0.411 0.452 905 940 -0.621702 0.078723

ENSMUST00000003612 558 0.1974 0.5724 0.0882 0.3953 0.287 0.233 0.556 42.73 0.767 0.573 180 185 -0.255676 0.075676

ENSMUST00000004136 657 0.2738 0.4643 0.1523 0.4184 0.232 0.021 0.427 47.27 0.665 0.517 206 218 -0.466055 0.133028

ENSMUST00000002112 480 0.0938 0.5234 0.0984 0.5041 0.267 0.193 0.520 35.30 0.842 0.642 152 159 0.122013 0.094340

ENSMUST00000002398 3234 0.1969 0.4284 0.1946 0.4056 0.245 0.144 0.488 48.16 0.675 0.561 1035 1077 0.153203 0.100279

ENSMUST00000003061 1869 0.2109 0.4355 0.2012 0.3561 0.251 0.121 0.485 48.64 0.656 0.599 602 622 -0.343730 0.072347

ENSMUST00000002412 3807 0.2459 0.4071 0.2180 0.3299 0.218 0.064 0.454 50.62 0.611 0.598 1217 1268 -0.480521 0.063091

ENSMUST00000002133 660 0.2873 0.3370 0.2545 0.3354 0.207 0.064 0.457 53.49 0.548 0.528 208 219 -0.334703 0.068493

ENSMUST00000002452 432 0.2033 0.5203 0.1743 0.2843 0.276 0.138 0.511 51.60 0.679 0.587 137 143 0.210489 0.104895

ENSMUST00000002291 3171 0.2773 0.3659 0.2257 0.4343 0.248 0.059 0.448 54.52 0.609 0.538 1031 1056 -0.543561 0.066288

ENSMUST00000003395 1833 0.2004 0.4515 0.2044 0.4111 0.272 0.130 0.491 48.36 0.676 0.563 580 610 -0.332623 0.065574

ENSMUST00000002844 921 0.2669 0.3267 0.1952 0.4043 0.203 0.025 0.423 53.59 0.604 0.576 293 306 -0.325490 0.049020

ENSMUST00000002818 597 0.2500 0.4079 0.2745 0.3425 0.284 0.154 0.510 56.16 0.583 0.500 192 198 -0.311616 0.090909

ENSMUST00000003981 1380 0.3531 0.2803 0.3924 0.2377 0.188 -0.024 0.396 52.49 0.405 0.441 447 459 -0.371460 0.080610

ENSMUST00000002683 1023 0.2374 0.4241 0.1509 0.4825 0.272 0.074 0.467 44.26 0.698 0.608 334 340 -0.929412 0.076471

ENSMUST00000004389 381 0.2245 0.4184 0.1553 0.4242 0.252 0.015 0.430 37.48 0.686 0.603 121 126 0.088095 0.055556

ENSMUST00000002911 2010 0.2286 0.3469 0.2260 0.4531 0.194 -0.107 0.370 47.81 0.638 0.580 660 669 -1.433.782 0.029895

ENSMUST00000003947 825 0.2864 0.3932 0.2573 0.3508 0.300 0.116 0.488 48.62 0.569 0.499 260 274 -0.231387 0.116788

ENSMUST00000002466 1173 0.1433 0.4909 0.0654 0.5084 0.293 0.213 0.538 37.19 0.823 0.678 379 390 -0.143077 0.076923

ENSMUST00000002756 588 0.4000 0.2444 0.3836 0.2828 0.220 0.023 0.429 51.52 0.392 0.438 189 195 -0.700513 0.076923

ENSMUST00000003622 936 0.1462 0.4731 0.2181 0.3894 0.230 0.149 0.500 49.05 0.699 0.580 302 311 -0.108360 0.080386

ENSMUST00000002891 3012 0.2861 0.3823 0.2533 0.3467 0.231 0.055 0.453 53.09 0.569 0.532 970 1003 -0.447757 0.081755

ENSMUST00000003655 1131 0.3612 0.3579 0.2421 0.3221 0.250 0.041 0.443 58.36 0.522 0.503 370 376 -0.284309 0.079787

ENSMUST00000002320 1323 0.1261 0.5916 0.1090 0.4950 0.297 0.177 0.525 39.37 0.818 0.588 423 440 -0.336818 0.081818

ENSMUST00000003808 912 0.1521 0.4068 0.1452 0.4531 0.241 0.285 0.558 45.73 0.741 0.666 294 303 -0.279538 0.036304

ENSMUST00000002336 1740 0.2517 0.3811 0.2938 0.4071 0.237 -0.021 0.430 52.87 0.585 0.500 574 579 -1.052.504 0.060449

ENSMUST00000003876 2856 0.3626 0.2679 0.3064 0.2921 0.221 -0.018 0.407 56.23 0.449 0.508 913 951 -0.495268 0.036803

ENSMUST00000004202 4863 0.2398 0.4354 0.2191 0.3945 0.245 0.083 0.472 49.71 0.639 0.543 1573 1620 -0.745802 0.077160

ENSMUST00000003436 933 0.1970 0.5130 0.0847 0.4240 0.317 0.233 0.551 37.70 0.759 0.618 303 310 -0.080323 0.100000

ENSMUST00000002549 1236 0.2595 0.3819 0.2432 0.3584 0.224 0.085 0.462 54.33 0.589 0.514 392 411 -0.007299 0.114355

ENSMUST00000004120 1128 0.4089 0.2405 0.3018 0.3359 0.207 -0.085 0.355 51.73 0.436 0.447 358 375 -0.144267 0.082667

ENSMUST00000003123 339 0.0625 0.5313 0.2198 0.5393 0.274 -0.011 0.434 38.50 0.774 0.548 106 112 -1.359.821 0.044643

ENSMUST00000004200 690 0.3681 0.2883 0.3955 0.2824 0.209 -0.064 0.400 56.45 0.422 0.463 225 229 -1.431.004 0.052402

ENSMUST00000002848 3972 0.1449 0.4939 0.1138 0.4158 0.256 0.217 0.532 41.43 0.776 0.673 1271 1323 -0.202646 0.088435

ENSMUST00000002733 1527 0.2464 0.4527 0.1845 0.4303 0.250 -0.039 0.422 45.75 0.671 0.559 493 508 -1.300.591 0.055118

ENSMUST00000003762 1752 0.1956 0.4274 0.1889 0.3653 0.240 0.146 0.490 48.96 0.673 0.602 547 583 0.116981 0.126930

ENSMUST00000003100 1476 0.1976 0.4902 0.1801 0.3776 0.259 0.207 0.525 46.90 0.691 0.548 472 491 -0.134827 0.101833

ENSMUST00000002989 1224 0.1719 0.4613 0.1335 0.4056 0.254 0.180 0.509 43.74 0.736 0.646 397 407 -0.129238 0.071253

ENSMUST00000003135 1176 0.2533 0.5000 0.1541 0.3640 0.245 0.106 0.489 45.52 0.676 0.565 374 391 -0.808184 0.097187

ENSMUST00000003898 2292 0.2795 0.4060 0.1873 0.3993 0.251 0.111 0.482 51.69 0.626 0.536 732 763 -0.355570 0.099607

ENSMUST00000003320 2154 0.2517 0.4231 0.2505 0.3359 0.246 0.133 0.490 53.93 0.596 0.532 698 717 -0.279358 0.071130

ENSMUST00000007803 702 0.2500 0.3859 0.2011 0.4107 0.246 0.036 0.448 48.66 0.633 0.559 221 233 -0.385837 0.111588

ENSMUST00000009143 1293 0.1343 0.5857 0.1213 0.4321 0.263 0.195 0.533 42.58 0.797 0.593 413 430 -0.402326 0.104651

ENSMUST00000009877 1509 0.2617 0.3808 0.2362 0.4245 0.232 0.054 0.450 53.87 0.608 0.518 487 502 -0.536454 0.085657

ENSMUST00000011934 80661 0.3443 0.3156 0.3449 0.2445 0.231 0.024 0.433 54.68 0.440 0.472 26138 26886 -0.469278 0.070743

ENSMUST00000010239 1581 0.2482 0.4177 0.1550 0.4133 0.252 0.171 0.507 48.50 0.667 0.560 505 526 -0.209316 0.079848

ENSMUST00000009220 513 0.1360 0.4880 0.2160 0.4472 0.222 0.126 0.488 45.61 0.725 0.590 160 170 -0.837059 0.100000

ENSMUST00000012580 3009 0.3579 0.2754 0.3086 0.3333 0.220 0.017 0.415 56.94 0.468 0.460 968 1002 -0.031936 0.080838

ENSMUST00000006462 1308 0.2746 0.4075 0.1867 0.3684 0.309 0.133 0.499 50.21 0.624 0.577 417 435 -0.266667 0.066667

ENSMUST00000005651 2037 0.1856 0.5114 0.1325 0.4628 0.274 0.125 0.495 43.95 0.748 0.575 652 678 -0.449558 0.104720

ENSMUST00000007318 1251 0.1719 0.5031 0.1115 0.5267 0.282 0.158 0.506 37.96 0.780 0.593 409 416 -0.512260 0.043269

ENSMUST00000006217 777 0.2688 0.3817 0.2184 0.4249 0.262 0.090 0.472 49.66 0.617 0.540 248 258 -0.257752 0.081395

ENSMUST00000005238 1251 0.1880 0.4672 0.1840 0.3780 0.236 0.096 0.470 46.07 0.685 0.547 400 416 0.414183 0.117788

ENSMUST00000005509 867 0.1401 0.5700 0.1106 0.5306 0.288 0.126 0.504 37.01 0.804 0.558 276 288 -0.594097 0.045139

ENSMUST00000005057 2064 0.1459 0.4933 0.1354 0.5160 0.295 0.135 0.496 40.83 0.779 0.598 661 687 -0.424163 0.088792

ENSMUST00000010941 1083 0.2603 0.4658 0.1984 0.3222 0.255 0.074 0.473 49.23 0.630 0.553 338 360 -0.418611 0.091667

ENSMUST00000010502 861 0.2770 0.3615 0.1833 0.4391 0.228 0.065 0.438 46.69 0.633 0.549 281 286 -0.238462 0.048951

ENSMUST00000012440 1620 0.2353 0.4259 0.1648 0.3964 0.217 0.073 0.450 48.64 0.671 0.607 529 539 -0.770872 0.035250

ENSMUST00000004920 3114 0.3565 0.2812 0.3091 0.2755 0.218 0.016 0.426 55.25 0.449 0.491 1006 1037 -0.347252 0.060752

ENSMUST00000008052 1032 0.3796 0.2774 0.3333 0.2348 0.221 0.041 0.443 51.91 0.410 0.476 327 343 0.021283 0.061224

ENSMUST00000011733 1491 0.1592 0.5172 0.1821 0.4060 0.287 0.151 0.512 43.40 0.727 0.577 473 496 -0.431048 0.076613

ENSMUST00000004673 1116 0.2953 0.3893 0.2183 0.3396 0.256 0.126 0.492 51.06 0.579 0.536 356 371 -0.274393 0.080863

ENSMUST00000008094 1320 0.1737 0.4398 0.1715 0.4424 0.265 0.106 0.467 44.61 0.715 0.616 424 439 -0.177677 0.075171

ENSMUST00000012587 3159 0.4095 0.2298 0.3695 0.2753 0.237 0.014 0.432 52.12 0.386 0.421 1029 1052 -0.530038 0.044677

ENSMUST00000008684 468 0.2803 0.4318 0.1786 0.3173 0.223 0.117 0.476 50.12 0.612 0.505 147 155 0.150968 0.116129

ENSMUST00000006254 735 0.2421 0.4368 0.2258 0.3687 0.247 0.014 0.435 59.52 0.629 0.549 237 244 -0.530328 0.098361

ENSMUST00000004576 1659 0.3356 0.3333 0.2394 0.3300 0.213 0.038 0.433 55.95 0.529 0.505 529 552 -0.242391 0.096014

ENSMUST00000005103 1983 0.3164 0.3239 0.3260 0.2869 0.211 0.019 0.431 58.63 0.483 0.505 642 660 -0.562879 0.056061

ENSMUST00000013807 1212 0.4051 0.2690 0.4340 0.2612 0.219 -0.119 0.362 53.55 0.380 0.400 392 403 -0.689826 0.124069

ENSMUST00000006392 1134 0.3036 0.3714 0.3007 0.3282 0.258 0.041 0.443 49.03 0.529 0.460 357 377 -0.036339 0.100796

ENSMUST00000008605 1134 0.2902 0.4006 0.1873 0.3451 0.237 0.086 0.459 50.69 0.602 0.549 357 377 -0.044828 0.122016

ENSMUST00000013842 393 0.2447 0.5532 0.1635 0.3804 0.316 0.176 0.520 43.12 0.685 0.515 127 130 -0.643846 0.061538

ENSMUST00000011315 1314 0.2070 0.4597 0.2547 0.3077 0.252 0.194 0.520 50.24 0.621 0.517 417 437 0.292906 0.118993

ENSMUST00000005678 996 0.2328 0.4224 0.2857 0.3761 0.233 0.062 0.461 58.85 0.600 0.517 310 331 -0.625378 0.075529

ENSMUST00000010211 813 0.2829 0.3902 0.1916 0.4078 0.239 0.095 0.471 52.12 0.624 0.548 263 270 -0.778889 0.074074

ENSMUST00000005950 1422 0.3763 0.3184 0.3715 0.2181 0.216 -0.019 0.412 55.32 0.414 0.435 449 473 -0.314165 0.158562

ENSMUST00000007130 2346 0.3720 0.3092 0.2388 0.3105 0.248 0.081 0.460 55.33 0.496 0.520 744 781 -0.170679 0.047375

ENSMUST00000006557 840 0.2434 0.4867 0.1737 0.3313 0.203 0.091 0.455 47.64 0.652 0.507 253 279 0.380286 0.168459

ENSMUST00000007754 1479 0.1769 0.4222 0.1594 0.4119 0.236 0.165 0.498 43.91 0.710 0.640 486 492 0.013618 0.038618

ENSMUST00000010974 645 0.1768 0.5193 0.1391 0.4222 0.277 0.212 0.525 44.28 0.740 0.500 204 214 0.630374 0.182243

ENSMUST00000010248 600 0.2697 0.3876 0.1484 0.3421 0.211 0.190 0.505 52.60 0.630 0.580 192 199 0.280904 0.100503

ENSMUST00000009790 588 0.1923 0.5128 0.1955 0.3798 0.302 0.200 0.541 40.50 0.697 0.568 185 195 -0.211282 0.107692

ENSMUST00000011178 1998 0.2369 0.4448 0.1932 0.3560 0.254 0.108 0.474 51.06 0.637 0.518 625 665 0.553985 0.114286

ENSMUST00000009344 3375 0.2841 0.3951 0.1950 0.3954 0.244 0.110 0.469 53.02 0.613 0.506 1089 1124 0.001512 0.096085

ENSMUST00000005487 501 0.2406 0.4060 0.1860 0.4098 0.220 0.001 0.406 52.10 0.650 0.552 160 166 -0.064458 0.060241

ENSMUST00000008582 2190 0.4307 0.2771 0.3241 0.2384 0.213 -0.079 0.375 52.37 0.398 0.448 701 729 -0.098217 0.089163

ENSMUST00000006854 4083 0.2831 0.3636 0.1985 0.3905 0.232 0.049 0.438 51.29 0.607 0.571 1327 1360 -0.471912 0.069118

ENSMUST00000012921 1278 0.2240 0.3825 0.1657 0.3930 0.193 0.082 0.440 45.55 0.662 0.620 414 425 -0.129647 0.070588

ENSMUST00000004990 1083 0.2822 0.3624 0.1818 0.4805 0.241 0.070 0.453 49.99 0.629 0.519 342 360 -0.323333 0.094444

ENSMUST00000004829 1194 0.3407 0.3470 0.2423 0.3466 0.218 0.025 0.439 49.53 0.535 0.479 385 397 -0.508816 0.095718

ENSMUST00000012679 2772 0.3442 0.2898 0.3194 0.3092 0.205 0.000 0.403 55.32 0.466 0.469 888 923 0.037811 0.073673

ENSMUST00000007042 1674 0.3525 0.3159 0.3199 0.3614 0.231 -0.021 0.425 54.60 0.497 0.479 537 557 -1.427.828 0.071813

ENSMUST00000007272 1455 0.2507 0.5041 0.0833 0.4247 0.352 0.261 0.582 34.57 0.733 0.587 464 484 -0.538017 0.059917

ENSMUST00000011776 639 0.2143 0.4890 0.2067 0.3241 0.253 0.083 0.481 49.12 0.660 0.569 206 212 -0.096698 0.080189

ENSMUST00000010795 1275 0.2402 0.3939 0.1416 0.4169 0.229 0.072 0.458 45.97 0.672 0.601 415 424 0.049292 0.037736

ENSMUST00000004774 810 0.2143 0.5000 0.0952 0.3743 0.296 0.245 0.550 46.20 0.727 0.581 260 269 0.496283 0.078067

ENSMUST00000009885 1515 0.3299 0.3477 0.2992 0.2986 0.239 0.046 0.454 57.29 0.499 0.485 487 504 -0.531548 0.079365

ENSMUST00000009774 930 0.3425 0.3110 0.3206 0.3299 0.242 -0.003 0.416 54.90 0.483 0.485 298 309 -0.445631 0.113269

ENSMUST00000006301 1947 0.2046 0.4493 0.1660 0.4107 0.232 0.066 0.446 47.05 0.695 0.588 626 648 -0.179012 0.067901

ENSMUST00000013294 1158 0.2006 0.4498 0.1390 0.4291 0.278 0.147 0.496 45.53 0.716 0.587 363 385 0.025455 0.075325

ENSMUST00000009058 3831 0.3291 0.3350 0.2781 0.3135 0.218 0.013 0.426 54.74 0.504 0.470 1235 1276 0.048119 0.088558

ENSMUST00000009036 852 0.4052 0.2716 0.3186 0.2746 0.240 -0.011 0.436 56.88 0.422 0.441 275 283 -0.315194 0.113074

ENSMUST00000006587 816 0.1222 0.5249 0.1538 0.4637 0.301 0.256 0.559 44.31 0.777 0.611 256 271 -0.079336 0.118081

ENSMUST00000007738 1203 0.2098 0.4397 0.1266 0.3902 0.263 0.198 0.529 45.68 0.708 0.667 384 400 -0.186750 0.097500

ENSMUST00000005643 1542 0.3575 0.3350 0.2481 0.3029 0.231 0.047 0.456 54.75 0.507 0.495 487 513 -0.224756 0.064327

ENSMUST00000010682 375 0.3404 0.3617 0.2784 0.3118 0.238 0.063 0.459 61.00 0.516 0.489 122 124 -0.356452 0.048387

ENSMUST00000009705 1962 0.1785 0.5228 0.1906 0.2966 0.273 0.191 0.517 47.10 0.686 0.574 634 653 0.167228 0.059724

ENSMUST00000005019 417 0.2165 0.4124 0.2190 0.4536 0.248 0.042 0.453 45.79 0.656 0.512 128 138 -0.467391 0.072464

ENSMUST00000012279 645 0.1154 0.4945 0.1141 0.4302 0.196 0.227 0.536 41.95 0.799 0.699 209 214 -0.692991 0.046729

ENSMUST00000008088 720 0.2172 0.4495 0.0941 0.4271 0.282 0.263 0.564 45.16 0.737 0.665 236 239 -0.597908 0.058577

ENSMUST00000007977 2409 0.2242 0.3363 0.2391 0.3636 0.200 0.014 0.410 49.58 0.599 0.614 770 802 0.057855 0.063591

ENSMUST00000012734 786 0.3455 0.3141 0.3041 0.3862 0.238 -0.069 0.395 54.81 0.516 0.502 258 261 -0.732950 0.114943

ENSMUST00000010804 2271 0.2099 0.4812 0.1380 0.4676 0.286 0.177 0.523 45.96 0.725 0.567 727 756 -0.503836 0.085979

ENSMUST00000012355 570 0.2426 0.4338 0.1688 0.4133 0.207 0.057 0.450 49.38 0.672 0.600 180 189 -1.078.836 0.042328

ENSMUST00000005751 2571 0.2358 0.3962 0.2410 0.3613 0.219 0.062 0.452 50.84 0.610 0.565 830 856 -0.698598 0.032710

ENSMUST00000013773 6678 0.2516 0.4071 0.1858 0.3773 0.242 0.078 0.453 49.19 0.635 0.574 2153 2225 -0.069483 0.069213

ENSMUST00000007533 447 0.2308 0.3750 0.2091 0.4717 0.275 0.210 0.537 52.71 0.654 0.520 136 148 -0.464189 0.128378

ENSMUST00000008004 1443 0.1868 0.4176 0.1637 0.5111 0.266 0.132 0.487 46.45 0.718 0.572 468 480 -0.194167 0.056250

ENSMUST00000004827 1635 0.2783 0.3939 0.2782 0.3015 0.210 0.001 0.424 54.07 0.551 0.507 521 544 -0.558824 0.075368

ENSMUST00000011152 1701 0.2642 0.4345 0.1965 0.4113 0.264 0.170 0.515 52.30 0.634 0.485 544 566 0.048940 0.127208

ENSMUST00000005829 2301 0.2462 0.4536 0.1739 0.4264 0.256 0.090 0.469 47.38 0.670 0.536 734 766 -0.539295 0.105744

ENSMUST00000006667 7284 0.3756 0.3019 0.3991 0.2506 0.224 0.007 0.429 51.73 0.407 0.414 2338 2427 -0.505604 0.088999

ENSMUST00000005532 3738 0.2621 0.4314 0.2205 0.3302 0.245 0.088 0.475 48.84 0.607 0.556 1214 1245 -0.364819 0.089960

ENSMUST00000009219 648 0.1411 0.5460 0.0828 0.5603 0.316 0.168 0.515 41.15 0.824 0.581 204 215 -0.118140 0.079070

ENSMUST00000010969 3075 0.1897 0.4928 0.1504 0.3689 0.242 0.186 0.515 44.83 0.714 0.614 994 1024 -0.479785 0.062500

ENSMUST00000009174 906 0.3333 0.3474 0.2620 0.3972 0.246 0.031 0.448 53.72 0.548 0.489 290 301 -0.672757 0.073090

ENSMUST00000007949 483 0.0847 0.6780 0.0305 0.4683 0.317 0.314 0.608 31.44 0.908 0.646 153 160 -0.836250 0.075000

ENSMUST00000006476 774 0.1881 0.4954 0.1751 0.3608 0.293 0.156 0.506 47.62 0.696 0.558 237 257 0.482490 0.147860

ENSMUST00000005493 1632 0.2197 0.4371 0.1916 0.3789 0.218 0.060 0.439 49.66 0.653 0.525 513 543 0.381216 0.073665

ENSMUST00000007800 339 0.3600 0.2400 0.3152 0.3736 0.253 -0.016 0.426 56.54 0.481 0.500 108 112 -1.060.714 0.044643

ENSMUST00000009727 705 0.1429 0.6327 0.0671 0.4000 0.328 0.264 0.582 39.85 0.827 0.615 225 234 0.003419 0.162393

ENSMUST00000012540 918 0.3529 0.4027 0.1688 0.3480 0.281 0.110 0.495 50.94 0.589 0.527 285 305 -0.772131 0.098361

ENSMUST00000008445 1158 0.3261 0.3478 0.3064 0.3404 0.222 0.035 0.450 57.88 0.515 0.490 373 385 -0.928571 0.051948

ENSMUST00000011492 1878 0.2820 0.3700 0.2130 0.3877 0.244 0.101 0.476 54.70 0.595 0.519 607 625 -0.057760 0.073600

ENSMUST00000008594 384 0.3229 0.4271 0.2588 0.3425 0.306 0.155 0.521 57.13 0.555 0.475 119 127 -0.200787 0.110236

ENSMUST00000005279 1341 0.2350 0.4235 0.2107 0.3684 0.262 0.190 0.522 49.94 0.636 0.568 431 446 -0.610090 0.069507

ENSMUST00000006893 3246 0.2627 0.3783 0.2273 0.3514 0.226 0.097 0.472 50.70 0.593 0.544 1045 1081 -0.344588 0.064755

ENSMUST00000004968 2226 0.2309 0.4302 0.1664 0.4347 0.251 0.148 0.494 47.71 0.679 0.572 710 741 -0.415924 0.112011

ENSMUST00000011055 726 0.3611 0.3167 0.3198 0.3121 0.250 0.013 0.438 57.01 0.469 0.481 226 241 -0.352697 0.082988

ENSMUST00000005220 1677 0.2418 0.4292 0.1733 0.3887 0.245 0.081 0.450 50.49 0.649 0.518 527 558 0.495341 0.062724

ENSMUST00000006716 1095 0.1608 0.4695 0.1679 0.3949 0.240 0.174 0.511 44.74 0.724 0.659 352 364 -0.281044 0.054945

ENSMUST00000010249 660 0.2775 0.4188 0.1834 0.2994 0.241 0.191 0.526 51.58 0.602 0.536 211 219 0.536986 0.105023

ENSMUST00000006525 1680 0.1796 0.5033 0.1734 0.3666 0.239 0.158 0.508 42.81 0.709 0.628 543 559 -0.776565 0.041145

ENSMUST00000011029 474 0.3385 0.2923 0.3902 0.1930 0.229 -0.017 0.408 61.00 0.395 0.473 152 157 -0.109554 0.057325

ENSMUST00000006293 912 0.3213 0.3614 0.2412 0.3224 0.216 0.035 0.435 56.26 0.541 0.519 294 303 -0.586139 0.089109

ENSMUST00000005820 1077 0.2527 0.3682 0.2415 0.4297 0.211 0.028 0.429 54.27 0.609 0.528 343 358 -0.149162 0.083799

ENSMUST00000013458 1134 0.3259 0.3639 0.2941 0.2529 0.241 0.046 0.447 52.88 0.491 0.486 369 377 0.156764 0.066313

ENSMUST00000006614 2934 0.1884 0.5084 0.1480 0.4034 0.287 0.150 0.506 43.99 0.725 0.582 932 977 -0.236643 0.090072

ENSMUST00000005064 1524 0.1601 0.4532 0.1538 0.4715 0.259 0.125 0.479 41.67 0.742 0.600 493 507 -0.292702 0.059172

ENSMUST00000007482 501 0.3281 0.3594 0.2137 0.3468 0.208 0.058 0.434 48.61 0.560 0.538 159 166 -0.628313 0.096386

ENSMUST00000007171 2847 0.2484 0.3513 0.1969 0.3952 0.199 0.049 0.432 50.98 0.623 0.617 922 948 -0.226793 0.048523

ENSMUST00000006646 1593 0.1860 0.5193 0.1576 0.3979 0.250 0.158 0.512 44.98 0.722 0.587 508 530 -0.688868 0.075472

ENSMUST00000008991 7167 0.2247 0.3969 0.2056 0.4394 0.244 0.070 0.460 47.90 0.658 0.583 2295 2388 -0.647613 0.056951

ENSMUST00000006760 1674 0.2354 0.4305 0.1290 0.3966 0.226 0.111 0.463 47.26 0.694 0.625 542 557 -0.458887 0.041293

ENSMUST00000007884 1758 0.4742 0.2584 0.4253 0.2202 0.252 -0.047 0.425 45.33 0.343 0.390 577 585 -1.006.325 0.092308

ENSMUST00000007747 1914 0.1945 0.4597 0.1626 0.4280 0.248 0.139 0.493 44.69 0.709 0.608 615 637 -0.406436 0.070644

ENSMUST00000004910 1056 0.2297 0.4664 0.1365 0.4211 0.255 0.118 0.485 47.89 0.698 0.563 338 351 -0.131909 0.062678

ENSMUST00000010597 2634 0.2914 0.3611 0.2062 0.3132 0.209 0.049 0.437 53.20 0.570 0.560 854 877 -0.255872 0.030787

ENSMUST00000008451 633 0.3718 0.2885 0.4101 0.3053 0.232 -0.046 0.400 52.44 0.425 0.400 200 210 -0.192381 0.133333

ENSMUST00000010044 1059 0.1903 0.5398 0.1429 0.3750 0.293 0.213 0.552 46.21 0.730 0.605 337 352 -0.366761 0.093750

ENSMUST00000004686 726 0.2239 0.4030 0.1640 0.3812 0.199 0.135 0.473 46.02 0.664 0.584 226 241 0.117012 0.082988

ENSMUST00000008036 345 0.3478 0.4457 0.1398 0.2874 0.300 0.046 0.459 44.70 0.595 0.582 111 114 0.163158 0.052632

ENSMUST00000012104 2175 0.3516 0.3145 0.2682 0.3154 0.248 0.048 0.451 57.55 0.499 0.499 698 724 -0.655249 0.059392

ENSMUST00000005488 774 0.2597 0.4033 0.3030 0.3492 0.233 -0.003 0.419 53.26 0.565 0.502 246 257 -0.668482 0.066148

ENSMUST00000006911 912 0.3004 0.3202 0.2489 0.3468 0.196 0.001 0.401 52.93 0.541 0.543 292 303 -0.113202 0.082508

ENSMUST00000013227 333 0.2000 0.4105 0.1868 0.3827 0.199 0.082 0.443 52.10 0.660 0.542 106 110 0.634545 0.063636

ENSMUST00000006718 1254 0.1420 0.5455 0.1346 0.3967 0.285 0.308 0.593 40.31 0.772 0.654 403 417 -0.453237 0.067146

ENSMUST00000008016 360 0.1616 0.4444 0.1122 0.5000 0.298 0.357 0.607 40.74 0.769 0.636 117 119 -0.156303 0.033613

ENSMUST00000007257 726 0.2011 0.4656 0.2404 0.3829 0.260 0.114 0.481 49.53 0.654 0.535 237 241 -0.305394 0.087137

ENSMUST00000006444 7890 0.2974 0.3349 0.2294 0.3645 0.215 0.047 0.431 53.26 0.565 0.539 2539 2629 -0.183073 0.068467

ENSMUST00000006626 2703 0.2033 0.4714 0.1760 0.4551 0.273 0.146 0.505 46.55 0.702 0.569 857 900 -0.534445 0.075556

ENSMUST00000004480 351 0.1705 0.4659 0.1413 0.4725 0.287 0.218 0.545 39.64 0.750 0.612 112 116 -0.289655 0.060345

ENSMUST00000012426 1065 0.2070 0.4737 0.1667 0.4082 0.239 0.108 0.496 44.20 0.697 0.582 337 354 -0.417797 0.087571

ENSMUST00000010451 726 0.2300 0.4272 0.1366 0.3605 0.254 0.161 0.489 42.60 0.674 0.585 227 241 0.915768 0.145228

ENSMUST00000009102 1107 0.2743 0.3681 0.2104 0.3859 0.196 -0.010 0.414 54.44 0.605 0.583 365 368 -0.934783 0.054348

ENSMUST00000006178 1293 0.2343 0.4029 0.1756 0.4406 0.239 0.096 0.461 50.89 0.667 0.572 423 430 -0.176047 0.076744

ENSMUST00000005503 4077 0.3609 0.2904 0.2860 0.3234 0.211 -0.025 0.411 57.25 0.481 0.489 1317 1358 -0.472680 0.081738

ENSMUST00000009679 1398 0.4196 0.2560 0.3400 0.3202 0.239 0.009 0.434 57.74 0.425 0.437 452 465 -0.712903 0.090323

ENSMUST00000010673 603 0.0491 0.5153 0.0482 0.5875 0.280 0.223 0.531 28.77 0.918 0.692 194 200 0.008500 0.065000

ENSMUST00000010736 897 0.4713 0.2008 0.3243 0.2548 0.226 -0.007 0.415 49.68 0.353 0.440 289 298 -0.509396 0.097315

ENSMUST00000009522 1461 0.3040 0.3705 0.2493 0.2655 0.225 0.043 0.428 57.43 0.529 0.504 465 486 0.583333 0.117284

ENSMUST00000006380 822 0.2081 0.5068 0.1463 0.3788 0.260 0.161 0.517 46.72 0.711 0.614 263 273 -0.584615 0.069597

ENSMUST00000006470 8166 0.2920 0.3461 0.2383 0.3250 0.187 -0.017 0.395 52.20 0.556 0.593 2660 2721 -0.680007 0.048144

ENSMUST00000004507 1641 0.2854 0.3503 0.1968 0.4127 0.217 0.042 0.424 53.91 0.608 0.549 536 546 -0.261355 0.060440

ENSMUST00000006181 888 0.1963 0.5421 0.1878 0.4315 0.348 0.165 0.537 41.61 0.710 0.540 283 295 -0.355254 0.098305

ENSMUST00000004913 477 0.3040 0.3120 0.1966 0.4727 0.226 0.028 0.428 48.94 0.599 0.513 152 158 -0.262658 0.069620

ENSMUST00000010520 246 0.3214 0.2857 0.2394 0.4444 0.185 -0.054 0.380 57.93 0.557 0.506 79 81 -0.471605 0.024691

ENSMUST00000009798 1641 0.2030 0.5248 0.1811 0.3631 0.281 0.156 0.511 47.28 0.696 0.567 536 546 -0.246154 0.073260

ENSMUST00000006659 798 0.4140 0.2837 0.3560 0.2151 0.211 0.022 0.424 57.17 0.384 0.406 255 265 -0.044528 0.079245

ENSMUST00000004784 918 0.1810 0.5603 0.1384 0.4038 0.304 0.215 0.557 45.23 0.747 0.578 289 305 -0.520656 0.072131

ENSMUST00000007139 342 0.3253 0.4217 0.1765 0.4605 0.311 0.168 0.527 47.24 0.625 0.493 112 113 -0.527434 0.079646

ENSMUST00000007266 396 0.2115 0.4135 0.2680 0.3978 0.227 0.010 0.414 60.12 0.625 0.519 128 131 -0.248092 0.091603

ENSMUST00000008297 2871 0.2724 0.4450 0.1399 0.3985 0.263 0.084 0.467 48.54 0.663 0.565 928 956 -0.302301 0.080544

ENSMUST00000012281 1365 0.3485 0.3110 0.3135 0.3043 0.192 -0.069 0.380 56.16 0.474 0.472 437 454 -0.482379 0.099119

ENSMUST00000007959 582 0.3333 0.2925 0.2639 0.4135 0.220 -0.036 0.395 60.57 0.530 0.487 185 193 -0.366321 0.072539

ENSMUST00000006792 978 0.3020 0.3412 0.3249 0.2941 0.195 -0.004 0.418 52.11 0.497 0.470 306 325 -0.324308 0.120000

ENSMUST00000013130 2391 0.3968 0.2111 0.3868 0.2681 0.204 -0.079 0.378 53.52 0.371 0.458 769 796 -0.531533 0.065327

ENSMUST00000006397 1524 0.1995 0.4200 0.1728 0.3858 0.243 0.146 0.487 46.90 0.681 0.615 489 507 -0.194477 0.080868

ENSMUST00000006391 1125 0.3286 0.3464 0.3040 0.3269 0.254 0.007 0.426 47.63 0.510 0.461 357 374 -0.200802 0.093583

ENSMUST00000006136 855 0.3054 0.4142 0.2153 0.3005 0.275 0.165 0.509 52.70 0.571 0.513 275 284 -0.001408 0.119718

ENSMUST00000008573 1215 0.3781 0.2719 0.3400 0.2517 0.213 -0.020 0.410 57.26 0.418 0.488 383 404 -0.476485 0.084158

ENSMUST00000006853 1512 0.2060 0.4467 0.1702 0.4266 0.251 0.129 0.484 48.03 0.694 0.590 481 503 -0.468986 0.079523

ENSMUST00000005051 1086 0.2776 0.4235 0.1685 0.3961 0.271 0.083 0.461 53.72 0.638 0.516 345 361 0.008587 0.069252

ENSMUST00000007245 2676 0.2373 0.4068 0.1717 0.3499 0.216 0.110 0.463 47.75 0.645 0.609 866 891 -0.043098 0.072952

ENSMUST00000013886 2349 0.2500 0.3661 0.1555 0.4277 0.199 0.037 0.437 49.47 0.662 0.638 767 782 -0.765985 0.026854

ENSMUST00000010085 2076 0.3410 0.3276 0.3467 0.3356 0.215 0.006 0.427 52.57 0.482 0.431 660 691 -0.609551 0.105644

ENSMUST00000009396 771 0.2186 0.4512 0.1602 0.3976 0.262 0.167 0.510 46.50 0.682 0.546 239 256 0.386719 0.132813

ENSMUST00000006435 1536 0.3253 0.3373 0.2563 0.3287 0.237 0.083 0.462 52.55 0.521 0.504 493 511 -0.176125 0.076321

ENSMUST00000007131 3276 0.2383 0.4743 0.1884 0.3519 0.244 0.061 0.456 48.69 0.651 0.547 1046 1091 -0.097892 0.087993

ENSMUST00000006128 978 0.2739 0.4191 0.1614 0.5189 0.318 0.150 0.514 48.84 0.674 0.539 313 325 -0.996615 0.098462

ENSMUST00000009234 2832 0.2547 0.4213 0.1696 0.4156 0.275 0.058 0.441 46.84 0.655 0.539 914 943 -0.038176 0.064687

ENSMUST00000006353 1737 0.3274 0.3073 0.3486 0.3052 0.222 -0.005 0.414 52.41 0.466 0.467 560 578 -0.432353 0.081315

ENSMUST00000010506 846 0.2603 0.3744 0.1858 0.4332 0.249 0.124 0.487 52.79 0.640 0.553 275 281 -0.161210 0.092527

ENSMUST00000010195 1389 0.2737 0.4228 0.2404 0.2923 0.247 0.077 0.460 53.21 0.580 0.547 433 462 -0.230520 0.114719

ENSMUST00000011450 1932 0.2495 0.4070 0.2255 0.4149 0.220 -0.045 0.400 46.87 0.630 0.554 613 643 -0.911042 0.074650

ENSMUST00000008745 642 0.2267 0.4942 0.1325 0.3861 0.286 0.107 0.488 43.42 0.705 0.570 207 213 -0.160094 0.084507

ENSMUST00000010421 6705 0.3314 0.3366 0.2381 0.3522 0.236 0.078 0.452 53.66 0.539 0.505 2141 2234 -0.275157 0.076992

ENSMUST00000005889 2538 0.2677 0.4157 0.1817 0.4643 0.248 0.032 0.441 48.22 0.654 0.532 812 845 -0.583195 0.102959

ENSMUST00000005841 2211 0.3817 0.3073 0.3171 0.3283 0.254 -0.004 0.430 55.18 0.472 0.490 712 736 -0.989538 0.048913

ENSMUST00000006037 4389 0.2701 0.3846 0.2548 0.3315 0.209 0.043 0.447 52.85 0.572 0.537 1384 1462 -0.668126 0.041724

ENSMUST00000005173 1509 0.1848 0.5071 0.1616 0.3905 0.279 0.215 0.537 45.95 0.718 0.585 482 502 -0.264940 0.111554

ENSMUST00000006235 1020 0.3039 0.4205 0.2759 0.2535 0.308 0.202 0.548 52.44 0.536 0.514 323 339 -0.305605 0.117994

ENSMUST00000006838 2328 0.3312 0.3344 0.2312 0.3755 0.242 0.031 0.431 53.76 0.550 0.521 751 775 -0.332774 0.091613

ENSMUST00000007207 2727 0.2172 0.4424 0.2008 0.3377 0.226 0.105 0.470 48.99 0.645 0.576 870 908 0.132269 0.085903

ENSMUST00000008633 1083 0.3208 0.3276 0.3148 0.3008 0.216 0.036 0.447 53.04 0.487 0.468 349 360 -0.383889 0.080556

ENSMUST00000009693 333 0.1410 0.4615 0.1556 0.5287 0.232 0.030 0.439 38.50 0.766 0.527 107 110 -0.780000 0.054545

ENSMUST00000013949 1809 0.3216 0.3320 0.2995 0.3094 0.217 -0.025 0.409 55.66 0.503 0.492 574 602 -0.296013 0.094684

ENSMUST00000010536 753 0.2475 0.3317 0.3730 0.3118 0.199 0.066 0.460 52.52 0.502 0.473 239 250 -0.527600 0.060000

ENSMUST00000005504 2793 0.4161 0.2354 0.3905 0.2573 0.199 -0.035 0.409 52.39 0.365 0.444 906 930 -0.613548 0.083871

ENSMUST00000006762 864 0.2276 0.4878 0.1336 0.3555 0.256 0.223 0.543 43.78 0.696 0.613 280 287 -0.418467 0.059233

ENSMUST00000005352 3342 0.3002 0.4203 0.2261 0.3243 0.260 0.106 0.485 53.55 0.581 0.534 1075 1113 -0.339533 0.077269

ENSMUST00000011981 1080 0.2711 0.3627 0.2115 0.3311 0.210 0.050 0.435 53.01 0.588 0.609 347 359 -0.493315 0.036212

ENSMUST00000009390 3477 0.2306 0.4332 0.1720 0.4118 0.252 0.109 0.469 46.13 0.671 0.562 1108 1158 -0.053022 0.096718

ENSMUST00000007865 654 0.1511 0.3957 0.1568 0.5738 0.240 0.016 0.429 37.29 0.762 0.621 210 217 -1.302.304 0.032258

ENSMUST00000007275 1314 0.2605 0.4551 0.1637 0.3944 0.314 0.218 0.559 44.24 0.661 0.557 422 437 -0.548513 0.070938

ENSMUST00000006053 2472 0.2530 0.4464 0.2013 0.3289 0.241 0.119 0.472 49.87 0.627 0.548 793 823 -0.071324 0.106926

ENSMUST00000004728 1212 0.2647 0.3412 0.2539 0.3344 0.197 0.051 0.428 47.47 0.559 0.522 388 403 0.045906 0.071960

ENSMUST00000004456 666 0.3275 0.3918 0.2611 0.3288 0.246 0.055 0.443 56.54 0.542 0.471 212 221 -0.168326 0.131222

ENSMUST00000013497 1104 0.2791 0.3887 0.2772 0.2576 0.190 0.059 0.434 51.08 0.532 0.490 348 367 0.117711 0.073569

ENSMUST00000010049 999 0.2586 0.3536 0.2519 0.3796 0.238 0.039 0.436 53.41 0.579 0.509 321 332 0.163253 0.069277

ENSMUST00000007156 786 0.2796 0.4313 0.2888 0.2500 0.229 0.065 0.449 51.81 0.543 0.512 247 261 -0.165134 0.076628

ENSMUST00000005592 2067 0.2312 0.4409 0.2318 0.3204 0.233 0.125 0.480 47.99 0.619 0.547 664 688 -0.257122 0.091570

ENSMUST00000010251 1614 0.2013 0.4698 0.2193 0.3168 0.241 0.148 0.492 50.32 0.644 0.535 514 537 0.346927 0.113594

ENSMUST00000006112 2982 0.2375 0.4676 0.1541 0.3657 0.240 0.084 0.463 49.18 0.674 0.577 952 993 -0.181672 0.086606

ENSMUST00000005364 1068 0.3098 0.3872 0.2449 0.2831 0.211 0.046 0.432 58.70 0.538 0.482 329 355 0.403099 0.152113

ENSMUST00000006005 1776 0.1976 0.4745 0.1560 0.3985 0.265 0.147 0.497 47.57 0.706 0.574 561 591 0.024704 0.118443

ENSMUST00000005791 522 0.3613 0.2773 0.2422 0.4417 0.226 -0.071 0.381 47.48 0.537 0.503 160 173 -0.424278 0.075145

ENSMUST00000010280 741 0.3936 0.2394 0.3667 0.2841 0.205 -0.093 0.374 51.83 0.404 0.457 235 246 -0.815854 0.113821

ENSMUST00000006704 2736 0.2260 0.4168 0.2225 0.4006 0.228 0.059 0.450 49.39 0.639 0.530 889 911 -0.282547 0.081229

ENSMUST00000005671 4110 0.2223 0.4968 0.1651 0.3965 0.246 0.107 0.480 48.43 0.690 0.543 1308 1369 -0.410884 0.095690

ENSMUST00000006703 2829 0.2668 0.4090 0.2239 0.3570 0.224 0.055 0.443 51.91 0.603 0.526 912 942 -0.298726 0.077495

ENSMUST00000005188 1866 0.2113 0.4321 0.1602 0.3830 0.255 0.128 0.494 43.51 0.684 0.632 605 621 -0.364251 0.064412

ENSMUST00000005003 1881 0.2672 0.3890 0.2328 0.3429 0.228 0.129 0.477 54.86 0.588 0.520 602 626 -0.110064 0.115016

ENSMUST00000006478 675 0.2128 0.5372 0.1818 0.2695 0.271 0.174 0.512 45.55 0.671 0.528 207 224 0.745089 0.169643

ENSMUST00000004868 501 0.1773 0.4965 0.1203 0.3730 0.276 0.218 0.532 41.90 0.741 0.612 158 166 0.022892 0.096386

ENSMUST00000012849 345 0.2258 0.4946 0.2840 0.2568 0.268 0.101 0.477 42.27 0.596 0.515 109 114 0.214035 0.078947

ENSMUST00000009831 540 0.2583 0.3709 0.2740 0.2941 0.221 0.044 0.440 49.81 0.549 0.527 175 179 -0.417877 0.050279

ENSMUST00000006764 543 0.1579 0.4342 0.1486 0.4783 0.231 0.185 0.506 44.18 0.742 0.596 178 180 0.091667 0.072222

ENSMUST00000006669 1305 0.2725 0.3708 0.2477 0.3574 0.204 0.069 0.452 60.71 0.577 0.515 418 434 -0.295852 0.099078

ENSMUST00000009329 294 0.2958 0.4085 0.1711 0.4203 0.268 0.112 0.457 47.52 0.630 0.509 92 97 0.245361 0.061856

ENSMUST00000013759 3234 0.3502 0.2252 0.3630 0.2770 0.221 -0.022 0.409 57.41 0.410 0.517 1039 1077 -0.760167 0.040854

ENSMUST00000005815 2940 0.2657 0.4010 0.2532 0.3438 0.237 0.035 0.440 53.97 0.583 0.500 941 979 -0.221553 0.096016

ENSMUST00000006027 570 0.1800 0.5333 0.1579 0.4065 0.290 0.150 0.506 44.51 0.730 0.538 178 189 0.123281 0.137566

ENSMUST00000009789 1605 0.3440 0.2629 0.3325 0.3604 0.225 -0.016 0.413 53.67 0.470 0.470 511 534 -0.497191 0.099251

ENSMUST00000005077 630 0.1420 0.4911 0.1600 0.3964 0.235 0.164 0.510 45.20 0.743 0.648 202 209 -0.441627 0.090909

ENSMUST00000006523 234 0.3276 0.4138 0.1923 0.4038 0.250 -0.012 0.446 38.15 0.608 0.554 74 77 -0.842857 0.103896

ENSMUST00000013299 1041 0.2148 0.4185 0.1945 0.3936 0.205 0.141 0.484 51.68 0.661 0.611 339 346 -0.889017 0.034682

ENSMUST00000007255 858 0.2379 0.3548 0.2050 0.3547 0.215 0.099 0.459 54.17 0.613 0.621 279 285 -0.010877 0.035088

ENSMUST00000012314 2922 0.3918 0.2726 0.4657 0.2441 0.220 -0.178 0.341 42.12 0.370 0.403 960 973 -0.648407 0.098664

ENSMUST00000006625 2010 0.3363 0.3783 0.1724 0.2818 0.291 0.132 0.515 51.86 0.563 0.599 656 669 -0.422720 0.094170

ENSMUST00000013766 672 0.3871 0.2849 0.2048 0.3590 0.215 0.057 0.451 55.62 0.507 0.519 215 223 -0.001794 0.058296

ENSMUST00000009777 312 0.1299 0.3896 0.1429 0.5222 0.262 0.135 0.480 39.63 0.770 0.644 100 103 0.177670 0.029126

ENSMUST00000008517 1500 0.2650 0.3880 0.1798 0.4497 0.246 -0.021 0.413 47.46 0.647 0.542 482 499 -0.491182 0.056112

ENSMUST00000006749 2790 0.2031 0.4479 0.1774 0.3806 0.237 0.158 0.485 48.17 0.678 0.568 895 929 0.250377 0.090420

ENSMUST00000005907 495 0.1890 0.4646 0.2381 0.3833 0.213 0.081 0.465 50.47 0.660 0.553 159 164 -0.612195 0.085366

ENSMUST00000006660 762 0.4384 0.2562 0.3820 0.1939 0.214 -0.069 0.369 50.74 0.349 0.385 241 253 0.013834 0.106719

ENSMUST00000013559 1734 0.2233 0.4605 0.2038 0.3986 0.254 0.090 0.475 51.35 0.658 0.551 564 577 -0.459619 0.048527

ENSMUST00000007340 3108 0.2802 0.4070 0.2178 0.3629 0.254 0.068 0.456 53.48 0.593 0.497 997 1035 0.045314 0.081159

ENSMUST00000008542 1230 0.1701 0.4985 0.1722 0.3662 0.266 0.200 0.525 45.12 0.713 0.570 400 409 -0.214670 0.066015

ENSMUST00000006286 1407 0.2572 0.4698 0.1812 0.3702 0.267 0.159 0.502 52.49 0.647 0.504 442 468 -0.218376 0.128205

ENSMUST00000006137 2121 0.2626 0.3855 0.2180 0.4155 0.259 0.070 0.457 51.96 0.616 0.525 683 706 -0.313739 0.080737

ENSMUST00000005041 1428 0.2747 0.3973 0.1607 0.4273 0.217 0.018 0.425 50.59 0.648 0.553 457 475 -0.577684 0.071579

ENSMUST00000004681 3984 0.2489 0.3971 0.2056 0.3531 0.219 0.063 0.442 51.95 0.617 0.571 1284 1327 -0.202185 0.065561

ENSMUST00000010899 2496 0.2572 0.4019 0.2332 0.4060 0.227 0.030 0.439 52.05 0.617 0.525 798 831 -0.589531 0.089049

ENSMUST00000013220 2907 0.2232 0.4209 0.2035 0.4155 0.226 0.062 0.461 48.83 0.659 0.573 941 968 -0.885021 0.046488

ENSMUST00000006559 1281 0.1903 0.4718 0.1442 0.3826 0.236 0.203 0.519 50.46 0.716 0.598 412 426 0.009390 0.068075

ENSMUST00000005749 3522 0.3237 0.3527 0.3096 0.3436 0.244 0.015 0.442 54.88 0.518 0.486 1148 1173 -0.895141 0.075021

ENSMUST00000013771 1101 0.1450 0.4944 0.1219 0.5589 0.300 0.164 0.513 42.01 0.793 0.596 353 366 -0.434699 0.046448

ENSMUST00000004565 621 0.1678 0.4899 0.2250 0.4342 0.285 0.112 0.495 43.57 0.695 0.537 200 206 -0.599029 0.082524

ENSMUST00000004473 729 0.2995 0.4011 0.3684 0.2405 0.203 0.066 0.470 48.18 0.487 0.463 232 242 -0.676859 0.128099

ENSMUST00000011493 1113 0.2767 0.3711 0.2208 0.2760 0.179 0.042 0.410 47.63 0.562 0.619 361 370 -0.273784 0.027027

ENSMUST00000005705 2505 0.3558 0.3483 0.1765 0.3473 0.272 0.068 0.462 50.65 0.563 0.572 812 834 -0.408753 0.044365

ENSMUST00000008999 3366 0.2085 0.4405 0.1362 0.4553 0.252 0.128 0.489 45.04 0.721 0.604 1092 1121 -0.437199 0.042819

ENSMUST00000007993 2253 0.3075 0.3230 0.2894 0.3873 0.242 -0.009 0.425 56.02 0.542 0.502 736 750 -0.912667 0.050667

ENSMUST00000009667 2733 0.2219 0.4438 0.1779 0.4220 0.235 0.063 0.446 46.47 0.678 0.571 883 910 -0.629670 0.064835

ENSMUST00000004959 654 0.1796 0.4970 0.1625 0.4605 0.303 0.214 0.545 44.61 0.732 0.578 209 217 -0.590323 0.129032

ENSMUST00000005336 1707 0.2911 0.3711 0.2442 0.3325 0.195 0.047 0.438 52.76 0.564 0.550 543 568 -0.675000 0.082746

ENSMUST00000012028 630 0.1049 0.5494 0.1169 0.5461 0.340 0.194 0.527 37.06 0.826 0.582 201 209 -0.070813 0.110048

ENSMUST00000007449 951 0.2468 0.4468 0.2134 0.4144 0.274 0.081 0.464 43.48 0.644 0.513 306 316 -0.331646 0.094937

ENSMUST00000009728 576 0.1056 0.6273 0.1168 0.3953 0.339 0.251 0.568 41.50 0.822 0.599 185 191 0.221990 0.167539

ENSMUST00000009713 3276 0.2713 0.3637 0.2557 0.3345 0.244 0.081 0.457 52.57 0.565 0.537 1067 1091 -0.552521 0.033914

ENSMUST00000006750 1341 0.1383 0.5216 0.1591 0.4324 0.264 0.185 0.527 41.87 0.758 0.592 429 446 -0.474664 0.076233

ENSMUST00000011058 1506 0.3684 0.2506 0.2947 0.2969 0.220 0.019 0.420 56.25 0.443 0.508 483 501 -0.235329 0.045908

ENSMUST00000010250 1638 0.1934 0.4857 0.1551 0.3676 0.255 0.220 0.534 45.17 0.704 0.575 517 545 0.339083 0.099083

ENSMUST00000006544 1251 0.2479 0.4387 0.1615 0.3682 0.273 0.173 0.517 45.44 0.654 0.586 402 416 -0.006731 0.064904

ENSMUST00000012873 3138 0.4213 0.2398 0.4715 0.2394 0.216 -0.195 0.330 41.06 0.342 0.389 1033 1045 -0.672153 0.101435

ENSMUST00000012262 1002 0.2530 0.4177 0.1937 0.4310 0.252 0.091 0.467 52.01 0.649 0.548 319 333 -0.385285 0.087087

ENSMUST00000009772 8358 0.4790 0.1937 0.4502 0.1774 0.220 -0.060 0.399 46.48 0.279 0.375 2727 2785 -0.654650 0.066068

ENSMUST00000007046 1485 0.2391 0.3419 0.1763 0.4536 0.189 0.057 0.421 52.34 0.654 0.575 480 494 0.142915 0.048583

ENSMUST00000012664 945 0.1828 0.5187 0.1550 0.3135 0.266 0.220 0.567 47.26 0.710 0.649 307 314 -0.430573 0.070064

ENSMUST00000004786 633 0.1325 0.4702 0.1446 0.5686 0.278 0.137 0.490 42.22 0.782 0.584 202 210 -0.512381 0.076190

ENSMUST00000005365 681 0.4600 0.2333 0.3274 0.3806 0.209 -0.080 0.390 50.14 0.431 0.391 218 226 -0.831416 0.079646

ENSMUST00000004936 360 0.2577 0.4845 0.1667 0.3412 0.289 0.176 0.521 55.42 0.650 0.529 117 119 0.098319 0.050420

ENSMUST00000006035 1152 0.2126 0.4651 0.1250 0.5136 0.258 0.053 0.451 47.39 0.735 0.552 370 383 -0.254830 0.093995

ENSMUST00000006742 4389 0.2340 0.4443 0.1990 0.3461 0.236 0.080 0.460 49.94 0.637 0.544 1407 1462 0.088919 0.050616

ENSMUST00000010201 1143 0.2148 0.5000 0.1603 0.4402 0.292 0.174 0.512 45.39 0.705 0.534 373 380 -0.225000 0.084211

ENSMUST00000010007 849 0.2294 0.4679 0.2010 0.3989 0.283 0.167 0.522 50.77 0.660 0.535 268 282 -0.381206 0.088652

ENSMUST00000009321 2322 0.3672 0.3121 0.2738 0.3310 0.219 -0.042 0.398 57.01 0.497 0.501 749 773 -0.720440 0.064683

ENSMUST00000008579 1005 0.2463 0.3860 0.1894 0.3984 0.218 0.028 0.429 55.91 0.637 0.559 322 334 -0.084731 0.068862

ENSMUST00000004729 768 0.2147 0.3717 0.1689 0.4878 0.264 0.069 0.446 43.75 0.687 0.575 249 255 -0.083137 0.023529

ENSMUST00000009538 1761 0.2051 0.4588 0.1840 0.3773 0.247 0.106 0.483 49.13 0.678 0.586 565 586 -0.444881 0.073379

ENSMUST00000007216 1308 0.2387 0.4743 0.1536 0.4407 0.268 0.084 0.468 46.90 0.688 0.522 417 435 -0.272644 0.091954

ENSMUST00000009039 348 0.3488 0.3023 0.2556 0.3827 0.265 0.099 0.482 49.80 0.518 0.449 110 115 -0.294783 0.060870

ENSMUST00000010348 525 0.1689 0.4257 0.1304 0.4701 0.198 0.075 0.450 49.78 0.746 0.644 169 174 -0.213218 0.045977

ENSMUST00000005234 1821 0.2976 0.4286 0.2046 0.3317 0.251 0.018 0.440 50.07 0.594 0.516 588 606 -0.271122 0.095710

ENSMUST00000005256 1185 0.2414 0.5172 0.1379 0.3261 0.270 0.129 0.495 45.77 0.685 0.574 372 394 -0.315990 0.055838

ENSMUST00000005548 870 0.2275 0.4360 0.1667 0.4450 0.265 0.060 0.451 46.97 0.687 0.564 275 289 -0.422145 0.086505

ENSMUST00000007584 2895 0.3193 0.3243 0.2655 0.3197 0.237 0.057 0.448 54.79 0.517 0.514 943 964 -0.242116 0.076763

ENSMUST00000006508 1707 0.2447 0.4620 0.1618 0.3529 0.284 0.155 0.506 49.16 0.659 0.562 551 568 0.011091 0.077465

ENSMUST00000006915 807 0.2417 0.4028 0.2412 0.3915 0.230 0.025 0.422 55.40 0.616 0.551 258 268 -0.432463 0.100746

ENSMUST00000007280 1410 0.2639 0.4722 0.1173 0.4108 0.298 0.232 0.564 40.05 0.694 0.564 454 469 -0.569083 0.063966

ENSMUST00000007248 1926 0.2127 0.4652 0.1752 0.4397 0.277 0.104 0.487 47.04 0.691 0.543 624 641 -0.402652 0.063963

ENSMUST00000006991 2733 0.2622 0.4140 0.2284 0.3663 0.247 0.129 0.494 54.31 0.606 0.523 868 910 -0.383956 0.085714

ENSMUST00000009340 1278 0.3526 0.2596 0.4214 0.2595 0.243 0.008 0.443 44.88 0.393 0.429 415 425 -0.600941 0.058824

ENSMUST00000009018 591 0.2450 0.4967 0.2727 0.2721 0.252 0.147 0.505 49.98 0.596 0.519 188 196 -0.478571 0.076531

ENSMUST00000010807 1344 0.1944 0.5139 0.1500 0.4235 0.270 0.160 0.504 47.69 0.726 0.557 419 447 -0.084340 0.152125

ENSMUST00000013787 768 0.3033 0.4692 0.2283 0.2601 0.348 0.205 0.560 49.82 0.576 0.498 250 255 -0.110196 0.086275

ENSMUST00000011302 2031 0.2222 0.4068 0.2280 0.3996 0.217 0.025 0.436 52.91 0.636 0.561 659 676 -0.538314 0.041420

ENSMUST00000011607 1248 0.3867 0.3565 0.2793 0.2647 0.282 0.028 0.444 53.42 0.476 0.470 399 415 -0.385783 0.130120

ENSMUST00000007814 2247 0.3003 0.3944 0.1984 0.3234 0.220 0.061 0.459 49.13 0.583 0.616 727 748 -0.740642 0.057487

ENSMUST00000004554 615 0.3168 0.4099 0.1509 0.3846 0.285 0.057 0.454 47.45 0.617 0.542 196 204 -0.380392 0.058824

ENSMUST00000007251 1677 0.1753 0.4740 0.1408 0.4350 0.216 0.082 0.456 44.81 0.737 0.599 533 558 -0.201613 0.107527

ENSMUST00000006900 423 0.2170 0.3019 0.4144 0.2913 0.183 -0.037 0.397 53.35 0.473 0.464 131 140 0.009286 0.028571

ENSMUST00000009236 687 0.1231 0.5538 0.1227 0.4063 0.270 0.319 0.581 39.17 0.797 0.596 217 228 0.593421 0.171053

ENSMUST00000013845 630 0.3390 0.2881 0.3438 0.2013 0.207 0.049 0.452 61.00 0.416 0.496 197 209 0.053588 0.086124

ENSMUST00000006105 1431 0.2481 0.3887 0.1973 0.3649 0.233 0.060 0.447 50.83 0.623 0.576 454 476 0.053781 0.077731

ENSMUST00000008090 843 0.1234 0.5532 0.0913 0.4185 0.247 0.212 0.545 42.84 0.818 0.692 275 280 -0.490357 0.085714

ENSMUST00000005053 423 0.3491 0.2642 0.3009 0.2752 0.198 -0.063 0.388 61.00 0.450 0.517 129 140 -0.036429 0.100000

ENSMUST00000005218 2343 0.3349 0.3333 0.3259 0.2559 0.241 0.004 0.441 54.46 0.467 0.490 752 780 -0.788333 0.066667

ENSMUST00000005452 2400 0.2087 0.4444 0.1717 0.3809 0.229 0.113 0.468 48.74 0.680 0.587 769 799 -0.175720 0.078849

ENSMUST00000007733 1245 0.2773 0.2991 0.2805 0.3832 0.218 0.033 0.428 57.75 0.547 0.537 400 414 -0.498792 0.065217

ENSMUST00000006956 369 0.3085 0.4255 0.2791 0.2500 0.217 0.126 0.513 53.59 0.531 0.530 113 122 -0.789344 0.114754

ENSMUST00000004494 3297 0.2274 0.4380 0.1400 0.4950 0.300 0.168 0.517 45.12 0.713 0.574 1065 1098 -0.638616 0.090164

ENSMUST00000006496 585 0.2848 0.3907 0.1132 0.4730 0.230 0.098 0.458 47.10 0.679 0.569 190 194 -0.644330 0.067010

ENSMUST00000006851 2334 0.3222 0.3222 0.2804 0.3483 0.270 0.099 0.475 52.83 0.520 0.514 756 777 -0.451351 0.063063

ENSMUST00000008830 1638 0.4638 0.2569 0.4079 0.2528 0.253 -0.051 0.422 49.01 0.363 0.412 535 545 -1.082.202 0.088073

ENSMUST00000004684 3066 0.2027 0.4376 0.2084 0.4167 0.244 0.075 0.462 47.68 0.669 0.577 995 1021 -0.646131 0.052889

ENSMUST00000006403 1212 0.2676 0.4190 0.1698 0.4477 0.269 0.119 0.495 48.42 0.663 0.554 386 403 -0.794293 0.054591

ENSMUST00000005108 5073 0.3841 0.2400 0.3785 0.2850 0.212 -0.070 0.375 54.72 0.403 0.455 1615 1690 -0.523254 0.070414

ENSMUST00000013302 924 0.2227 0.3013 0.2381 0.4424 0.199 -0.074 0.359 47.80 0.618 0.594 304 307 -0.488925 0.019544

ENSMUST00000008748 1791 0.2286 0.4615 0.1476 0.3807 0.219 0.101 0.476 45.50 0.687 0.593 559 596 -0.381879 0.033557

ENSMUST00000008528 711 0.1771 0.4323 0.1701 0.4149 0.237 0.083 0.443 45.60 0.706 0.627 228 236 -0.307203 0.025424

ENSMUST00000004749 300 0.2838 0.5000 0.1772 0.2692 0.255 0.218 0.538 39.46 0.624 0.609 93 99 -0.989899 0.020202

ENSMUST00000004646 1425 0.2091 0.4906 0.1852 0.4063 0.230 0.043 0.445 51.11 0.685 0.542 454 474 -0.352110 0.071730

ENSMUST00000010208 1518 0.1991 0.4871 0.1361 0.4088 0.273 0.172 0.506 45.75 0.717 0.547 484 505 0.563960 0.112871

ENSMUST00000013970 1266 0.2733 0.4414 0.1774 0.4014 0.268 0.072 0.462 47.44 0.643 0.533 409 421 -0.384561 0.099762

ENSMUST00000010267 1704 0.2521 0.4063 0.1828 0.3696 0.241 0.124 0.471 55.07 0.628 0.536 543 567 0.662610 0.086420

ENSMUST00000013737 1392 0.2849 0.3425 0.2105 0.4313 0.197 -0.024 0.397 51.44 0.596 0.538 436 463 -0.283369 0.095032

ENSMUST00000011526 1002 0.2642 0.3962 0.2205 0.3548 0.231 0.014 0.421 56.92 0.601 0.551 321 333 0.000000 0.078078

ENSMUST00000005017 714 0.2372 0.4167 0.2240 0.4628 0.247 -0.097 0.392 51.64 0.655 0.564 232 237 -1.267.089 0.063291

ENSMUST00000013235 501 0.2388 0.3433 0.3223 0.3276 0.204 0.034 0.439 55.43 0.542 0.548 155 166 -0.254217 0.084337

ENSMUST00000008893 1455 0.2628 0.4235 0.1880 0.3642 0.225 0.045 0.440 48.77 0.629 0.563 464 484 -0.344421 0.074380

ENSMUST00000005976 720 0.1951 0.3805 0.2251 0.3784 0.195 0.083 0.446 51.96 0.641 0.596 231 239 -0.113389 0.083682

ENSMUST00000009120 1365 0.3731 0.2418 0.3583 0.3169 0.224 -0.026 0.402 56.40 0.420 0.459 438 454 -0.364978 0.059471

ENSMUST00000004965 642 0.3358 0.3285 0.3353 0.3185 0.271 0.045 0.465 59.57 0.480 0.448 198 213 -0.687324 0.018779

ENSMUST00000004986 1101 0.1832 0.5018 0.1476 0.4764 0.284 0.169 0.517 44.59 0.741 0.562 348 366 -0.395902 0.101093

ENSMUST00000010904 435 0.1284 0.6147 0.0748 0.4900 0.390 0.342 0.623 34.41 0.841 0.586 138 144 -0.431945 0.111111

ENSMUST00000006687 621 0.2867 0.3733 0.2895 0.3929 0.228 0.054 0.444 55.10 0.561 0.474 198 206 -0.494660 0.082524

ENSMUST00000013882 1335 0.3634 0.2492 0.4116 0.2809 0.226 -0.028 0.402 53.53 0.395 0.441 423 444 -0.394820 0.081081

ENSMUST00000005952 1293 0.2806 0.3444 0.3006 0.3080 0.212 0.056 0.440 58.31 0.521 0.474 409 430 0.145814 0.076744

ENSMUST00000006825 3771 0.2214 0.4094 0.1903 0.3802 0.220 0.093 0.461 48.09 0.653 0.588 1221 1256 -0.250637 0.066083

ENSMUST00000013755 1317 0.2702 0.3758 0.2236 0.4430 0.281 0.192 0.526 49.51 0.618 0.511 416 438 -0.387671 0.100457

ENSMUST00000005606 1056 0.2366 0.5153 0.1181 0.4764 0.291 0.118 0.494 45.18 0.728 0.529 338 351 -0.407692 0.128205

ENSMUST00000005714 552 0.2029 0.5072 0.1353 0.5164 0.289 0.133 0.497 48.72 0.743 0.532 179 183 -0.516940 0.098361

ENSMUST00000008734 1314 0.2888 0.4087 0.1824 0.3596 0.232 0.097 0.456 51.79 0.609 0.509 419 437 0.088101 0.112128

ENSMUST00000006632 855 0.2292 0.3636 0.1461 0.4047 0.234 0.160 0.480 48.57 0.665 0.631 269 284 0.660915 0.088028

ENSMUST00000006786 1344 0.2709 0.3722 0.2257 0.3133 0.206 0.076 0.452 55.26 0.564 0.505 427 447 0.703356 0.102908

ENSMUST00000006774 1644 0.3585 0.2561 0.3623 0.3222 0.223 -0.050 0.398 53.41 0.436 0.441 527 547 -0.521024 0.076782

ENSMUST00000007790 1155 0.4000 0.1903 0.4067 0.2500 0.169 -0.156 0.318 53.80 0.342 0.438 374 384 -0.067969 0.072917

ENSMUST00000013931 3792 0.2099 0.4493 0.1450 0.4383 0.216 0.051 0.448 44.60 0.709 0.613 1224 1263 -0.560174 0.048298

ENSMUST00000008812 459 0.2101 0.4118 0.1818 0.4727 0.222 0.098 0.466 49.79 0.682 0.546 148 152 -0.709869 0.059211

ENSMUST00000004949 1593 0.3228 0.3714 0.2902 0.3248 0.242 0.044 0.447 56.05 0.524 0.496 510 530 -0.395094 0.064151

ENSMUST00000008477 678 0.4424 0.2061 0.3720 0.3046 0.247 -0.043 0.411 49.11 0.374 0.410 214 225 -0.566222 0.093333

ENSMUST00000006828 1965 0.2460 0.3889 0.1806 0.4111 0.243 0.170 0.506 46.58 0.650 0.611 632 654 -0.636392 0.045872

ENSMUST00000007735 879 0.2402 0.4891 0.1586 0.3714 0.255 0.102 0.477 52.55 0.676 0.533 281 292 -0.183562 0.102740

ENSMUST00000004756 1245 0.2130 0.4467 0.1857 0.3939 0.267 0.122 0.491 47.77 0.675 0.568 397 414 -0.399517 0.091787

ENSMUST00000006061 3735 0.3327 0.3001 0.2981 0.3109 0.221 0.063 0.450 56.08 0.485 0.499 1216 1244 -0.287540 0.059486

ENSMUST00000006754 2259 0.2289 0.4103 0.1706 0.5115 0.244 0.063 0.461 49.21 0.699 0.536 700 752 -1.135.505 0.065160

ENSMUST00000007250 2502 0.2442 0.4321 0.1731 0.3889 0.229 0.128 0.477 50.80 0.654 0.551 811 833 0.020048 0.073229

ENSMUST00000005954 519 0.1818 0.3058 0.3056 0.4714 0.214 0.033 0.426 56.35 0.609 0.525 169 172 -0.828488 0.034884

ENSMUST00000004683 1743 0.2164 0.5030 0.1283 0.3916 0.273 0.178 0.513 45.88 0.712 0.556 563 580 0.143276 0.117241

ENSMUST00000006969 1269 0.2233 0.4207 0.2312 0.4239 0.228 0.083 0.472 51.89 0.641 0.531 407 422 -0.738152 0.042654

ENSMUST00000011398 897 0.2684 0.3636 0.2133 0.4577 0.199 -0.041 0.396 48.45 0.622 0.515 283 298 -0.400000 0.127517

ENSMUST00000005830 678 0.2975 0.2975 0.2917 0.4459 0.226 -0.030 0.408 61.00 0.549 0.493 213 225 -0.703556 0.080000

ENSMUST00000005620 1023 0.2841 0.4945 0.1790 0.3149 0.262 0.154 0.514 51.25 0.628 0.540 331 340 -0.718235 0.088235

ENSMUST00000012798 1710 0.2341 0.4267 0.2210 0.3280 0.239 0.127 0.491 51.03 0.619 0.552 546 569 -0.163093 0.070299

ENSMUST00000007444 1296 0.2578 0.4391 0.2069 0.3446 0.237 0.097 0.471 53.26 0.621 0.527 414 431 -0.245940 0.088167

ENSMUST00000004732 522 0.1879 0.5503 0.1176 0.3000 0.296 0.232 0.554 42.61 0.732 0.582 157 173 0.650867 0.184971

ENSMUST00000007259 408 0.1525 0.5085 0.1942 0.3535 0.249 0.131 0.496 43.85 0.714 0.602 133 135 0.161481 0.044444

ENSMUST00000006618 2409 0.1750 0.4562 0.1371 0.4380 0.224 0.118 0.474 43.40 0.739 0.628 782 802 -0.468579 0.054863

ENSMUST00000006123 1173 0.2575 0.3619 0.1938 0.4901 0.204 -0.007 0.415 53.02 0.652 0.540 376 390 -0.802308 0.028205

ENSMUST00000004505 4002 0.2383 0.4757 0.1768 0.3243 0.271 0.181 0.515 48.19 0.653 0.548 1295 1333 0.218755 0.110278

ENSMUST00000006377 2385 0.1984 0.4878 0.1702 0.4381 0.292 0.108 0.496 46.82 0.712 0.591 780 794 -0.610453 0.050378

ENSMUST00000013667 1902 0.2025 0.3776 0.2873 0.3748 0.223 -0.025 0.419 53.36 0.602 0.560 623 633 -0.998262 0.028436

ENSMUST00000007012 669 0.3029 0.3771 0.1768 0.4286 0.271 0.111 0.491 50.53 0.617 0.541 214 222 -0.441892 0.094595

ENSMUST00000011298 912 0.2532 0.3734 0.2613 0.3971 0.223 0.079 0.460 55.61 0.592 0.505 287 303 -0.401320 0.069307

ENSMUST00000006611 909 0.1624 0.5812 0.1131 0.4369 0.297 0.206 0.536 38.86 0.782 0.588 289 302 -0.234106 0.092715

ENSMUST00000008273 198 0.1639 0.3443 0.1667 0.5000 0.168 0.183 0.484 44.59 0.710 0.626 62 65 0.584615 0.076923

ENSMUST00000006701 867 0.1726 0.4558 0.2385 0.3812 0.211 0.079 0.450 54.00 0.664 0.547 271 288 0.088542 0.104167

ENSMUST00000005810 1053 0.2740 0.3381 0.2686 0.3475 0.213 0.003 0.405 52.93 0.547 0.518 338 350 -0.087714 0.031429

ENSMUST00000004943 648 0.4790 0.1916 0.4710 0.1778 0.205 -0.105 0.368 49.96 0.268 0.358 209 215 -0.049302 0.125581

ENSMUST00000009732 1581 0.2609 0.3841 0.2388 0.3786 0.215 0.053 0.445 52.29 0.598 0.520 508 526 -0.524525 0.102662

ENSMUST00000005185 297 0.2639 0.4583 0.2192 0.3857 0.247 0.039 0.453 51.84 0.632 0.503 95 98 -0.478571 0.081633

ENSMUST00000008021 504 0.1513 0.4286 0.1448 0.5035 0.268 0.228 0.540 41.41 0.758 0.639 161 167 -0.700599 0.047904

ENSMUST00000011391 954 0.2209 0.4574 0.1783 0.3942 0.226 0.114 0.473 51.72 0.671 0.551 298 317 -0.059306 0.107256

ENSMUST00000012161 2502 0.1662 0.4972 0.1337 0.4066 0.255 0.180 0.532 43.82 0.750 0.682 816 833 -0.584994 0.040816

ENSMUST00000005891 756 0.2452 0.4375 0.1872 0.3371 0.233 0.159 0.498 47.38 0.637 0.584 237 251 -0.155378 0.083665

ENSMUST00000008987 636 0.2737 0.3743 0.2338 0.3188 0.242 0.114 0.459 54.20 0.566 0.509 196 211 0.573934 0.094787

ENSMUST00000004715 1557 0.3681 0.3029 0.3708 0.2655 0.233 0.036 0.442 55.41 0.426 0.418 493 518 -0.500965 0.104247

ENSMUST00000005923 795 0.2930 0.3814 0.1910 0.3369 0.254 0.168 0.516 52.77 0.589 0.561 246 264 -0.173106 0.102273

ENSMUST00000006662 726 0.3936 0.2606 0.3829 0.2454 0.223 -0.028 0.396 47.67 0.387 0.423 230 241 -0.134440 0.107884

ENSMUST00000008032 1278 0.1740 0.5083 0.1185 0.3868 0.237 0.170 0.509 44.20 0.751 0.635 409 425 -0.414353 0.082353

ENSMUST00000005607 609 0.2515 0.4737 0.1655 0.3684 0.259 0.097 0.462 46.75 0.660 0.548 197 202 -0.152970 0.089109

ENSMUST00000006046 771 0.2623 0.3552 0.2767 0.3532 0.192 0.044 0.436 54.54 0.564 0.579 241 256 -0.937109 0.066406

ENSMUST00000004470 8370 0.3107 0.3144 0.2974 0.3583 0.213 0.007 0.409 55.37 0.516 0.466 2707 2789 -0.128935 0.076013

ENSMUST00000009875 1956 0.2594 0.4544 0.1773 0.3074 0.244 0.080 0.462 49.96 0.631 0.566 634 651 -0.105837 0.096774

ENSMUST00000005825 3603 0.2936 0.4160 0.1948 0.3664 0.255 0.067 0.452 51.04 0.607 0.526 1159 1200 -0.223250 0.093333

ENSMUST00000012627 366 0.3889 0.2889 0.3370 0.2892 0.213 -0.051 0.379 52.17 0.431 0.446 116 121 -0.083471 0.074380

ENSMUST00000006664 723 0.3651 0.3228 0.3750 0.2483 0.215 0.025 0.426 53.30 0.426 0.400 230 240 -0.150000 0.100000

ENSMUST00000011895 7686 0.1662 0.3917 0.1384 0.5394 0.253 0.143 0.495 43.68 0.754 0.640 2471 2561 -0.597579 0.051933

ENSMUST00000005616 2841 0.2089 0.3876 0.1804 0.4406 0.211 0.057 0.440 47.04 0.676 0.595 924 946 -0.346723 0.062368

ENSMUST00000010298 2157 0.1831 0.4452 0.1517 0.4520 0.227 0.078 0.455 43.64 0.725 0.618 692 718 -0.490529 0.057103

ENSMUST00000006963 1389 0.2351 0.4448 0.1806 0.4103 0.276 0.168 0.525 49.66 0.667 0.556 451 462 -0.498052 0.045455

ENSMUST00000008826 645 0.3735 0.3916 0.1859 0.3451 0.257 0.011 0.429 52.71 0.556 0.511 205 214 -0.553271 0.088785

ENSMUST00000009259 1029 0.2366 0.4158 0.1460 0.4368 0.230 0.098 0.461 48.26 0.685 0.567 336 342 -0.384503 0.067251

ENSMUST00000013693 552 0.4031 0.2791 0.1986 0.4478 0.258 0.054 0.443 54.14 0.545 0.481 176 183 -0.366667 0.065574

ENSMUST00000007797 1425 0.3257 0.3791 0.2575 0.2767 0.204 -0.013 0.401 55.46 0.520 0.465 446 474 -0.068355 0.120253

ENSMUST00000008995 3231 0.2324 0.4007 0.1868 0.4256 0.241 0.092 0.466 48.91 0.661 0.584 1050 1076 -0.536152 0.044610

ENSMUST00000009435 525 0.2045 0.4015 0.1769 0.4880 0.225 0.134 0.488 52.05 0.695 0.557 164 174 -0.382184 0.068966

ENSMUST00000006451 1323 0.2927 0.3537 0.1753 0.4402 0.250 0.014 0.425 49.52 0.630 0.551 424 440 -0.431364 0.065909

ENSMUST00000006378 2064 0.1913 0.4609 0.1553 0.3909 0.280 0.180 0.511 45.46 0.704 0.573 656 687 0.401455 0.110626

ENSMUST00000005014 1026 0.1204 0.5485 0.1111 0.4160 0.240 0.229 0.544 41.23 0.805 0.670 333 341 -0.319062 0.111437

ENSMUST00000009699 1119 0.2000 0.4737 0.1236 0.5078 0.271 0.142 0.494 45.70 0.744 0.554 356 372 -0.362097 0.088710

ENSMUST00000009550 1290 0.2238 0.3683 0.2715 0.3073 0.216 0.051 0.436 50.07 0.571 0.580 420 429 -0.361072 0.051282

ENSMUST00000004574 1077 0.2888 0.3394 0.2985 0.3911 0.226 0.079 0.464 53.05 0.544 0.499 351 358 -0.570112 0.083799

ENSMUST00000005431 2217 0.2148 0.4000 0.1852 0.4708 0.261 0.084 0.458 46.53 0.680 0.555 721 738 -0.421816 0.043360

ENSMUST00000004770 1602 0.3575 0.3326 0.3333 0.2470 0.199 -0.047 0.394 54.17 0.453 0.463 508 533 -0.347842 0.129456

ENSMUST00000005072 1857 0.2114 0.4834 0.1573 0.3485 0.247 0.136 0.495 44.70 0.689 0.601 592 618 -0.301780 0.076052

ENSMUST00000006103 1104 0.2701 0.4759 0.1731 0.3231 0.229 0.103 0.464 53.89 0.632 0.479 351 367 0.452316 0.163488

ENSMUST00000009138 1398 0.2824 0.3471 0.3599 0.3248 0.230 0.028 0.441 54.56 0.502 0.457 438 465 -0.538495 0.101075

ENSMUST00000007253 1230 0.1816 0.4986 0.1290 0.4055 0.250 0.139 0.497 44.60 0.739 0.606 394 409 -0.215648 0.088020

ENSMUST00000007980 918 0.0423 0.6500 0.0335 0.4850 0.324 0.306 0.621 29.67 0.937 0.719 301 305 -0.526230 0.098361

ENSMUST00000010198 333 0.2198 0.4396 0.1818 0.3735 0.233 0.061 0.449 39.62 0.664 0.603 107 110 -0.420000 0.090909

ENSMUST00000007161 768 0.2010 0.4545 0.2487 0.3371 0.215 0.096 0.473 50.68 0.634 0.552 243 255 -0.011373 0.070588

ENSMUST00000012847 717 0.3164 0.4294 0.2515 0.3043 0.281 0.132 0.507 51.51 0.561 0.487 223 238 -0.413866 0.113445

ENSMUST00000005507 2595 0.2105 0.4474 0.1819 0.3487 0.192 0.090 0.453 48.85 0.666 0.599 836 864 -0.429630 0.067130

ENSMUST00000005975 1710 0.1670 0.4989 0.1755 0.4221 0.273 0.165 0.506 46.61 0.722 0.552 551 569 -0.040246 0.103691

ENSMUST00000010985 495 0.2677 0.3622 0.4138 0.2857 0.158 -0.034 0.399 50.88 0.481 0.417 158 164 -1.024.390 0.091463

ENSMUST00000006949 1467 0.2559 0.3984 0.2715 0.3721 0.247 0.104 0.481 53.80 0.589 0.501 474 488 -0.442418 0.104508

ENSMUST00000009358 666 0.1902 0.5435 0.1146 0.3581 0.294 0.191 0.524 43.25 0.743 0.564 206 221 0.501357 0.149321

ENSMUST00000005769 1038 0.2976 0.3333 0.2809 0.3896 0.239 0.013 0.423 53.90 0.547 0.507 331 345 -0.675072 0.046377

ENSMUST00000007602 837 0.2768 0.3348 0.3085 0.3651 0.230 0.060 0.451 56.64 0.537 0.498 268 278 -0.168345 0.093525

ENSMUST00000005798 1221 0.3693 0.2451 0.4324 0.2898 0.223 0.002 0.427 49.73 0.394 0.420 398 406 -0.604187 0.086207

ENSMUST00000009157 270 0.2656 0.4688 0.1525 0.5577 0.383 0.167 0.541 47.07 0.694 0.491 85 89 -0.448315 0.123596

ENSMUST00000009411 1479 0.2583 0.3611 0.1864 0.4627 0.243 0.094 0.476 48.69 0.650 0.568 477 492 -0.803862 0.063008

ENSMUST00000006638 1806 0.2189 0.4340 0.1667 0.3400 0.250 0.185 0.503 49.53 0.661 0.567 581 601 0.649751 0.076539

ENSMUST00000005647 792 0.3102 0.3472 0.2071 0.3883 0.206 -0.024 0.395 56.09 0.578 0.536 256 263 -0.349810 0.110266

ENSMUST00000013851 555 0.2215 0.4295 0.1389 0.4638 0.275 0.143 0.492 45.57 0.707 0.583 181 184 -0.113587 0.070652

ENSMUST00000012259 2370 0.2401 0.3899 0.2227 0.4082 0.264 0.114 0.479 48.27 0.630 0.580 756 789 -0.674651 0.032953

ENSMUST00000012152 1650 0.2835 0.4177 0.2026 0.3413 0.263 0.109 0.481 49.43 0.605 0.576 532 549 -0.459927 0.096539

ENSMUST00000007007 1191 0.3777 0.2626 0.3467 0.3035 0.236 0.017 0.444 59.46 0.436 0.468 385 396 -0.967424 0.042929

ENSMUST00000005334 1212 0.2741 0.3735 0.2387 0.3108 0.198 0.088 0.446 50.45 0.567 0.554 381 403 -0.175682 0.069479

ENSMUST00000009740 1416 0.4507 0.2716 0.3065 0.2869 0.253 0.024 0.442 54.46 0.420 0.467 457 471 -0.877919 0.050955

ENSMUST00000005015 1476 0.3185 0.3368 0.1990 0.3682 0.238 0.084 0.462 52.65 0.576 0.580 481 491 -0.692464 0.065173

ENSMUST00000007317 1212 0.2300 0.4952 0.0997 0.4365 0.289 0.161 0.518 38.30 0.734 0.582 394 403 -0.491067 0.057072

ENSMUST00000007559 801 0.2911 0.3944 0.2140 0.3367 0.231 0.081 0.483 54.30 0.583 0.541 259 266 -0.515414 0.086466

ENSMUST00000011400 2763 0.2659 0.4272 0.1912 0.3617 0.215 0.067 0.461 49.84 0.628 0.574 893 920 -0.574783 0.067391

ENSMUST00000010038 1242 0.2557 0.3770 0.2119 0.4217 0.281 0.157 0.503 51.05 0.624 0.529 396 413 -0.289104 0.062954

ENSMUST00000006692 1206 0.2000 0.4646 0.1519 0.4195 0.254 0.128 0.491 45.61 0.710 0.597 389 401 -0.227431 0.069825

ENSMUST00000004622 2001 0.2771 0.4440 0.2281 0.2738 0.235 0.077 0.470 49.24 0.584 0.542 645 666 -0.819369 0.067568

ENSMUST00000006303 753 0.1770 0.4833 0.1515 0.3947 0.218 0.101 0.465 47.50 0.724 0.596 243 250 -0.139200 0.068000

ENSMUST00000005601 1872 0.2475 0.3631 0.2169 0.3685 0.213 0.091 0.450 50.45 0.608 0.575 587 623 -0.224880 0.088283

ENSMUST00000006818 630 0.2514 0.3600 0.2267 0.3254 0.180 -0.028 0.398 51.66 0.587 0.593 201 209 -0.636364 0.052632

ENSMUST00000007212 2727 0.3404 0.3235 0.2294 0.3621 0.245 0.005 0.415 52.68 0.539 0.513 870 908 -0.173458 0.062775

ENSMUST00000006341 1530 0.2248 0.3653 0.2198 0.3679 0.218 0.112 0.472 54.19 0.620 0.592 492 509 0.068762 0.082515

ENSMUST00000006029 1278 0.1739 0.4754 0.1975 0.4156 0.282 0.224 0.534 44.09 0.702 0.562 416 425 -0.370118 0.098824

ENSMUST00000013910 405 0.2054 0.5268 0.1748 0.2871 0.187 0.049 0.434 49.17 0.682 0.619 129 134 -0.088806 0.067164

ENSMUST00000005233 4947 0.2705 0.3905 0.2567 0.3694 0.249 0.104 0.476 53.37 0.582 0.510 1612 1648 -0.418932 0.075243

ENSMUST00000006697 2670 0.2553 0.4502 0.1924 0.3855 0.265 0.080 0.469 48.98 0.642 0.514 864 889 -0.288302 0.091114

ENSMUST00000006745 216 0.3548 0.3710 0.2857 0.2174 0.205 0.058 0.449 55.90 0.478 0.488 69 71 0.084507 0.084507

ENSMUST00000011285 594 0.1887 0.5094 0.0976 0.4051 0.214 0.147 0.492 42.01 0.759 0.631 191 197 -0.434010 0.055838

ENSMUST00000008284 807 0.2287 0.3901 0.1126 0.4384 0.235 0.119 0.483 47.63 0.707 0.643 259 268 -0.125000 0.055970

ENSMUST00000010020 519 0.1736 0.4876 0.2093 0.4127 0.226 0.065 0.465 46.71 0.698 0.564 159 172 -0.651744 0.122093

ENSMUST00000005067 948 0.1784 0.5021 0.1371 0.4596 0.277 0.123 0.497 41.15 0.748 0.600 306 315 -0.500952 0.057143

ENSMUST00000005262 948 0.3745 0.2809 0.3938 0.2692 0.235 -0.003 0.428 49.60 0.408 0.425 299 315 -0.803492 0.111111

ENSMUST00000005669 1476 0.3208 0.3709 0.2376 0.3403 0.216 0.020 0.420 54.94 0.550 0.492 476 491 -0.075967 0.101833

ENSMUST00000005849 396 0.2455 0.3364 0.2079 0.4300 0.315 0.249 0.547 52.82 0.625 0.578 128 131 0.038168 0.053435

ENSMUST00000006467 903 0.3054 0.3682 0.3333 0.3081 0.254 0.012 0.433 56.13 0.509 0.459 293 300 -0.441667 0.103333

ENSMUST00000012186 978 0.2293 0.3045 0.3702 0.3162 0.197 0.017 0.429 50.43 0.505 0.509 319 325 -0.786154 0.043077

ENSMUST00000006094 1503 0.2127 0.4328 0.2342 0.3765 0.222 0.051 0.432 46.65 0.639 0.545 477 500 -0.103400 0.112000

ENSMUST00000007296 6852 0.2916 0.4055 0.2365 0.3633 0.247 0.057 0.450 52.32 0.583 0.505 2189 2283 -0.360578 0.095488

ENSMUST00000012348 657 0.2699 0.4601 0.1757 0.4380 0.271 0.059 0.454 47.26 0.659 0.508 205 218 -0.503211 0.137615

ENSMUST00000006565 1500 0.2804 0.4045 0.2377 0.2916 0.236 0.078 0.465 54.45 0.568 0.556 475 499 -0.466333 0.070140

ENSMUST00000010434 699 0.2961 0.2849 0.2667 0.4162 0.171 -0.066 0.371 53.36 0.549 0.496 224 232 -0.812069 0.034483

ENSMUST00000004587 987 0.2101 0.4514 0.1595 0.4118 0.225 0.106 0.478 48.14 0.699 0.628 316 328 -0.589634 0.076220

ENSMUST00000006311 1284 0.2018 0.5241 0.1829 0.3675 0.267 0.105 0.485 48.12 0.692 0.559 412 427 -0.495550 0.098361

ENSMUST00000006221 2934 0.4197 0.2066 0.3847 0.2693 0.207 -0.042 0.388 51.39 0.361 0.412 948 977 -0.230911 0.065507

ENSMUST00000005839 381 0.2857 0.3048 0.3100 0.3263 0.229 0.018 0.427 55.32 0.508 0.511 124 126 -0.373016 0.095238

ENSMUST00000005394 924 0.3208 0.3458 0.2800 0.3527 0.222 0.026 0.436 54.16 0.527 0.481 296 307 -0.453420 0.091205

ENSMUST00000009617 1887 0.2256 0.4662 0.1185 0.4027 0.245 0.107 0.478 47.04 0.708 0.601 607 628 -0.158917 0.090764

ENSMUST00000005711 669 0.2583 0.3444 0.2931 0.3841 0.242 0.056 0.454 51.67 0.561 0.512 205 222 -0.629279 0.018018

ENSMUST00000008051 1176 0.4318 0.2792 0.3799 0.1701 0.277 0.044 0.460 53.28 0.351 0.427 385 391 -0.630691 0.040921

ENSMUST00000004657 1428 0.2817 0.3655 0.3060 0.2682 0.191 0.004 0.412 54.11 0.514 0.487 459 475 -0.106526 0.090526

ENSMUST00000005413 2640 0.3407 0.3437 0.2936 0.3344 0.217 -0.040 0.416 51.92 0.513 0.516 859 879 -0.895791 0.076223

ENSMUST00000005826 1395 0.2745 0.3750 0.2225 0.3636 0.218 0.014 0.420 50.34 0.592 0.531 436 464 -0.186423 0.088362

ENSMUST00000009392 792 0.1659 0.4350 0.1500 0.4110 0.259 0.214 0.541 47.91 0.728 0.669 257 263 -0.428897 0.038023

ENSMUST00000004560 588 0.1867 0.4933 0.2465 0.3684 0.267 0.146 0.505 47.32 0.661 0.535 186 195 -0.416923 0.056410

ENSMUST00000007249 2124 0.2248 0.4826 0.1431 0.3790 0.250 0.145 0.487 48.07 0.691 0.541 679 707 0.482744 0.124470

ENSMUST00000013338 1479 0.3269 0.3929 0.2766 0.3758 0.241 -0.000 0.433 57.32 0.554 0.494 471 492 -0.738008 0.103659

ENSMUST00000007236 690 0.1823 0.4631 0.1695 0.3567 0.266 0.218 0.545 48.39 0.698 0.620 222 229 0.279913 0.139738

ENSMUST00000005073 1275 0.2562 0.4242 0.1728 0.3567 0.246 0.129 0.484 51.60 0.641 0.563 415 424 -0.054953 0.075472

ENSMUST00000005583 699 0.2211 0.4000 0.2330 0.3892 0.241 0.083 0.451 48.39 0.629 0.575 224 232 -0.316379 0.060345

ENSMUST00000009695 489 0.3740 0.2824 0.4000 0.1765 0.191 -0.036 0.401 61.00 0.369 0.479 157 162 -0.737037 0.037037

ENSMUST00000006973 2490 0.2016 0.4822 0.1708 0.4055 0.237 0.095 0.467 46.64 0.699 0.573 801 829 -0.417009 0.084439

ENSMUST00000011262 1179 0.2538 0.4159 0.2153 0.3532 0.231 0.109 0.463 50.28 0.614 0.511 376 392 0.185969 0.114796

ENSMUST00000010753 1347 0.1579 0.5180 0.1357 0.4263 0.268 0.145 0.489 44.01 0.757 0.576 423 448 0.324107 0.116071

ENSMUST00000007981 597 0.2335 0.4132 0.1656 0.3816 0.182 0.037 0.417 52.23 0.661 0.599 192 198 -0.413131 0.090909

ENSMUST00000010192 1326 0.2927 0.4444 0.1191 0.3487 0.270 0.197 0.528 45.89 0.654 0.596 436 441 -0.098640 0.052154

ENSMUST00000006761 1755 0.2660 0.3638 0.2923 0.3107 0.212 0.053 0.451 52.07 0.544 0.542 566 584 -0.867808 0.047945

ENSMUST00000005692 3105 0.2396 0.4470 0.1465 0.4244 0.293 0.142 0.498 46.28 0.682 0.561 994 1034 0.075919 0.087041

ENSMUST00000006362 684 0.3436 0.3129 0.2896 0.3315 0.236 0.127 0.498 48.37 0.502 0.552 219 227 -1.115.859 0.048458

ENSMUST00000006020 1995 0.3447 0.3769 0.2186 0.3432 0.278 0.103 0.475 51.93 0.549 0.485 638 664 -0.264157 0.106928

ENSMUST00000010940 1428 0.4126 0.2131 0.3441 0.3026 0.209 -0.010 0.407 51.47 0.396 0.429 462 475 -0.256842 0.054737

ENSMUST00000005817 927 0.2558 0.3605 0.1965 0.4063 0.230 0.093 0.464 49.46 0.624 0.564 295 308 -0.066234 0.094156

ENSMUST00000006104 1368 0.2376 0.4503 0.1727 0.4503 0.289 0.141 0.505 50.00 0.676 0.529 442 455 -0.179780 0.109890

ENSMUST00000008626 720 0.2368 0.4474 0.1771 0.4235 0.261 0.155 0.502 50.74 0.674 0.572 233 239 -0.471548 0.041841

ENSMUST00000009003 621 0.2848 0.3576 0.3399 0.3472 0.259 0.099 0.487 59.67 0.523 0.456 199 206 -0.620874 0.082524

ENSMUST00000010241 1857 0.3515 0.3414 0.3026 0.2763 0.190 -0.038 0.396 55.10 0.479 0.476 599 618 -0.658738 0.082524

ENSMUST00000006856 4398 0.4274 0.2196 0.3630 0.3152 0.229 -0.041 0.394 51.70 0.391 0.419 1418 1465 -0.436655 0.083959

ENSMUST00000006912 1218 0.2952 0.3810 0.2535 0.4091 0.240 0.021 0.437 57.20 0.579 0.477 394 405 -0.517778 0.088889

ENSMUST00000006424 651 0.4024 0.2561 0.3667 0.3139 0.242 -0.061 0.388 51.18 0.413 0.418 206 216 -0.337963 0.125000

ENSMUST00000007921 423 0.3423 0.4144 0.2857 0.2949 0.294 0.201 0.542 58.84 0.527 0.443 131 140 -0.252143 0.150000

ENSMUST00000010205 1053 0.2444 0.5185 0.1406 0.4299 0.334 0.191 0.539 45.23 0.699 0.524 336 350 -0.288571 0.091429

ENSMUST00000011896 765 0.2228 0.4974 0.1487 0.4056 0.276 0.164 0.510 45.13 0.701 0.573 241 254 -0.488189 0.082677

ENSMUST00000005292 597 0.2590 0.4759 0.1517 0.3806 0.263 0.132 0.497 54.63 0.667 0.544 195 198 -0.150505 0.106061

ENSMUST00000011877 7092 0.3103 0.3799 0.2371 0.4046 0.263 0.064 0.463 52.41 0.582 0.506 2249 2363 -0.755100 0.065595

ENSMUST00000006914 1602 0.1926 0.4289 0.2033 0.3790 0.229 0.157 0.491 50.22 0.669 0.590 525 533 -0.097561 0.088180

ENSMUST00000006952 393 0.2718 0.4757 0.2609 0.2644 0.199 0.101 0.492 53.84 0.581 0.518 124 130 -0.626154 0.169231

ENSMUST00000008907 1926 0.3294 0.2959 0.3565 0.2809 0.222 0.034 0.435 56.98 0.450 0.476 611 641 -0.382995 0.106084

ENSMUST00000009814 2844 0.4193 0.2125 0.4165 0.2532 0.217 -0.021 0.401 48.89 0.346 0.387 892 947 -0.139916 0.101373

ENSMUST00000005016 1428 0.2324 0.3864 0.2016 0.3952 0.207 0.077 0.438 50.31 0.640 0.579 461 475 -0.153895 0.061053

ENSMUST00000004750 1356 0.3361 0.2889 0.3183 0.3172 0.203 0.007 0.415 60.27 0.471 0.472 429 451 -0.285144 0.101996

ENSMUST00000010189 1053 0.1694 0.4319 0.1725 0.3919 0.212 0.103 0.445 50.09 0.703 0.598 337 350 0.553143 0.085714

ENSMUST00000004985 3615 0.2250 0.4543 0.1775 0.4119 0.237 0.107 0.477 47.40 0.678 0.576 1174 1204 -0.699086 0.058140

ENSMUST00000005862 1017 0.1715 0.4895 0.1596 0.4771 0.232 0.108 0.479 45.47 0.738 0.589 328 338 -0.872485 0.026627

ENSMUST00000011623 2361 0.2571 0.4075 0.2284 0.3328 0.202 0.072 0.446 51.95 0.601 0.561 769 786 -0.360942 0.063613

ENSMUST00000005069 588 0.1961 0.4837 0.1786 0.4179 0.239 0.096 0.465 50.06 0.703 0.554 185 195 -0.347693 0.102564

ENSMUST00000005964 1125 0.3411 0.3478 0.2644 0.2852 0.256 0.037 0.449 59.36 0.501 0.504 361 374 0.133957 0.069519

ENSMUST00000011445 714 0.2961 0.3966 0.2690 0.3292 0.242 0.036 0.448 54.54 0.556 0.488 223 237 -0.362447 0.101266

ENSMUST00000012331 1008 0.3045 0.3233 0.2863 0.3814 0.227 0.020 0.421 57.85 0.532 0.477 316 335 0.056119 0.164179

ENSMUST00000006785 1398 0.3174 0.3627 0.2735 0.2483 0.199 0.044 0.425 54.60 0.500 0.462 438 465 0.699355 0.131183

ENSMUST00000008280 840 0.2476 0.5333 0.2011 0.4260 0.333 0.151 0.533 43.67 0.676 0.528 272 279 -0.613620 0.111111

ENSMUST00000005066 1182 0.2328 0.4066 0.1922 0.4448 0.224 0.032 0.430 48.26 0.657 0.541 379 393 -0.299237 0.063613

ENSMUST00000006101 3504 0.2672 0.4203 0.2174 0.3615 0.246 0.054 0.452 51.42 0.610 0.519 1136 1167 -0.182434 0.083119

ENSMUST00000006367 1443 0.2120 0.4539 0.1718 0.3950 0.260 0.127 0.486 48.13 0.680 0.577 475 480 -0.104375 0.035417

ENSMUST00000006071 1068 0.1921 0.5066 0.1519 0.3346 0.295 0.219 0.560 45.83 0.710 0.630 341 355 -0.544507 0.073239

ENSMUST00000006431 1542 0.2337 0.4434 0.1513 0.4419 0.247 0.074 0.464 47.85 0.685 0.559 496 513 -0.219493 0.079922

ENSMUST00000004955 1110 0.2955 0.3540 0.2828 0.3320 0.253 0.026 0.427 52.39 0.525 0.473 354 369 0.029539 0.051491

ENSMUST00000004430 2601 0.3905 0.2580 0.3266 0.2591 0.211 -0.025 0.399 52.08 0.406 0.450 825 866 0.084411 0.112009

ENSMUST00000010279 630 0.2898 0.4205 0.2387 0.2794 0.205 0.040 0.445 52.99 0.560 0.488 200 209 -0.166029 0.076555

ENSMUST00000009256 1305 0.2827 0.3252 0.2326 0.3757 0.217 0.018 0.423 54.79 0.574 0.551 423 434 -0.247005 0.052995

ENSMUST00000005406 2088 0.2279 0.4695 0.2042 0.4072 0.288 0.084 0.473 48.17 0.665 0.555 666 695 -0.566475 0.058993

ENSMUST00000005120 597 0.2260 0.5342 0.2180 0.3680 0.268 0.138 0.511 45.59 0.667 0.525 186 198 -0.989899 0.095960

ENSMUST00000006721 594 0.1739 0.5155 0.2057 0.3525 0.241 0.156 0.519 47.48 0.698 0.596 189 197 -0.571066 0.126904

ENSMUST00000007799 537 0.1944 0.5903 0.1774 0.3529 0.358 0.227 0.556 44.70 0.708 0.506 171 178 0.042135 0.140449

ENSMUST00000013633 1590 0.2330 0.3665 0.2113 0.3759 0.213 0.007 0.413 47.97 0.620 0.585 508 529 -0.349906 0.054820

ENSMUST00000013304 1056 0.2518 0.4539 0.1680 0.4640 0.304 0.127 0.493 47.79 0.673 0.519 343 351 -0.092593 0.125356

ENSMUST00000010210 3447 0.2112 0.4709 0.1667 0.4253 0.268 0.140 0.495 47.74 0.698 0.559 1108 1148 -0.372300 0.099303

ENSMUST00000004614 2499 0.3810 0.2841 0.3006 0.3333 0.221 -0.013 0.423 55.08 0.470 0.472 802 832 -0.845673 0.061298

ENSMUST00000010127 1788 0.3758 0.3374 0.3056 0.2272 0.254 0.014 0.435 55.56 0.444 0.469 572 595 -0.005714 0.078992

ENSMUST00000010278 1029 0.3780 0.3643 0.1846 0.2569 0.252 0.103 0.471 55.07 0.520 0.543 329 342 -0.086842 0.070175

ENSMUST00000004453 666 0.3333 0.4035 0.2208 0.3514 0.238 -0.038 0.401 58.49 0.571 0.492 212 221 -0.264706 0.131222

ENSMUST00000005164 1197 0.2772 0.3993 0.2397 0.3971 0.251 0.059 0.460 50.86 0.598 0.502 383 398 -0.474874 0.123116

ENSMUST00000004508 684 0.2456 0.4737 0.1322 0.4321 0.322 0.248 0.560 44.62 0.699 0.564 216 227 -0.311894 0.088106

ENSMUST00000007005 1194 0.2879 0.3220 0.2795 0.3023 0.224 0.023 0.431 54.38 0.516 0.540 378 397 0.153652 0.037783

ENSMUST00000008966 300 0.2192 0.4247 0.2703 0.4000 0.257 0.060 0.463 49.59 0.621 0.505 95 99 -0.505051 0.111111

ENSMUST00000009689 2007 0.2098 0.4756 0.1528 0.3783 0.250 0.148 0.495 48.52 0.695 0.574 645 668 -0.107336 0.094311

ENSMUST00000013797 708 0.3586 0.2879 0.2818 0.2743 0.234 0.009 0.423 61.00 0.463 0.491 227 235 0.323404 0.055319

ENSMUST00000008878 1233 0.1487 0.4665 0.1581 0.4306 0.225 0.108 0.458 42.62 0.740 0.581 384 410 0.428780 0.104878

ENSMUST00000005933 786 0.2995 0.4106 0.2872 0.2571 0.243 0.086 0.459 47.09 0.528 0.503 246 261 -0.005747 0.084291

ENSMUST00000005630 2877 0.3045 0.2968 0.3243 0.3240 0.184 -0.050 0.391 56.04 0.486 0.463 930 958 -0.199478 0.080376

ENSMUST00000013262 1758 0.1968 0.4577 0.2039 0.4215 0.247 0.129 0.489 47.54 0.683 0.597 568 585 -0.824615 0.063248

ENSMUST00000004994 1455 0.2500 0.4337 0.2047 0.3370 0.225 0.092 0.472 49.67 0.623 0.565 470 484 -0.691322 0.066116

ENSMUST00000007757 1512 0.3833 0.2359 0.3893 0.2412 0.212 -0.032 0.398 53.18 0.371 0.448 480 503 -0.112724 0.071571

ENSMUST00000005255 1104 0.2025 0.4778 0.2308 0.3361 0.235 0.067 0.457 45.91 0.653 0.582 357 367 -0.223706 0.081744

ENSMUST00000005611 444 0.2476 0.4952 0.1881 0.4731 0.326 0.126 0.511 47.35 0.681 0.508 141 147 -0.465986 0.115646

ENSMUST00000008179 549 0.1250 0.5764 0.0977 0.4720 0.314 0.252 0.566 37.44 0.821 0.604 173 182 -0.489011 0.076923

ENSMUST00000006679 765 0.1574 0.4861 0.1218 0.4545 0.274 0.224 0.536 40.07 0.766 0.621 248 254 0.110630 0.074803

ENSMUST00000006578 1527 0.2217 0.3855 0.2122 0.3875 0.233 0.115 0.474 47.14 0.638 0.589 494 508 -0.409055 0.055118

ENSMUST00000004497 2271 0.2811 0.4242 0.2056 0.3706 0.254 0.110 0.478 50.15 0.613 0.516 719 756 -0.324074 0.117725

ENSMUST00000007708 1770 0.2787 0.3936 0.1761 0.4033 0.234 0.015 0.411 48.34 0.628 0.539 570 589 0.075042 0.056027

ENSMUST00000010188 1323 0.2094 0.4219 0.2035 0.4644 0.248 0.104 0.468 48.74 0.677 0.556 421 440 -0.334773 0.070455

ENSMUST00000005685 1485 0.2196 0.4729 0.1989 0.3905 0.239 0.084 0.464 48.14 0.667 0.526 472 494 -0.207692 0.123482

ENSMUST00000005600 2892 0.1804 0.4761 0.1429 0.4150 0.291 0.164 0.512 41.02 0.731 0.621 938 963 -0.313292 0.064382

ENSMUST00000005953 1353 0.3249 0.3361 0.2500 0.3450 0.209 -0.042 0.392 52.30 0.531 0.499 429 450 -0.171556 0.097778

ENSMUST00000008537 447 0.2381 0.4286 0.1597 0.3717 0.269 0.188 0.517 43.04 0.662 0.599 145 148 -0.482432 0.054054

ENSMUST00000009241 1467 0.1694 0.4894 0.1188 0.4225 0.287 0.224 0.548 41.41 0.757 0.661 473 488 -0.487500 0.090164

ENSMUST00000008733 1098 0.3218 0.2976 0.3746 0.2691 0.197 -0.074 0.396 56.36 0.446 0.505 359 365 -0.916712 0.101370

ENSMUST00000009356 1248 0.3408 0.3631 0.3269 0.2695 0.264 0.048 0.457 50.28 0.477 0.447 398 415 -0.245301 0.098795

ENSMUST00000010191 1422 0.2194 0.4770 0.1884 0.3373 0.252 0.130 0.490 48.01 0.665 0.588 451 473 -0.285412 0.122622

ENSMUST00000006151 1614 0.3725 0.3181 0.2992 0.2542 0.209 0.017 0.427 52.03 0.456 0.484 518 537 -0.381192 0.113594

ENSMUST00000008462 1311 0.3056 0.3278 0.2000 0.3584 0.220 0.029 0.425 49.40 0.571 0.589 424 436 -0.366284 0.043578

ENSMUST00000009707 966 0.1870 0.5229 0.1033 0.4181 0.292 0.232 0.542 43.16 0.760 0.609 308 321 -0.012773 0.099688

ENSMUST00000010579 549 0.2786 0.2786 0.4324 0.2794 0.211 -0.002 0.428 57.39 0.428 0.454 180 182 -0.637912 0.043956

ENSMUST00000004655 1428 0.2982 0.3383 0.3075 0.2614 0.188 0.002 0.403 55.05 0.492 0.487 461 475 0.014947 0.086316

ENSMUST00000010550 366 0.2386 0.3068 0.3107 0.3333 0.156 0.022 0.412 61.00 0.535 0.565 114 121 -0.733058 0.049587

ENSMUST00000005490 1686 0.2468 0.4030 0.1762 0.3535 0.238 0.133 0.479 48.66 0.631 0.549 534 561 0.403208 0.069519

ENSMUST00000018918 981 0.2877 0.4140 0.2490 0.2177 0.197 0.088 0.461 54.06 0.539 0.544 319 326 -0.110736 0.061350

ENSMUST00000016143 1506 0.2594 0.3915 0.2487 0.3351 0.220 0.054 0.446 53.62 0.583 0.559 487 501 -0.851098 0.055888

ENSMUST00000021853 954 0.3000 0.3280 0.3156 0.3158 0.219 -0.041 0.402 57.04 0.503 0.473 306 317 -0.241325 0.091483

ENSMUST00000020546 891 0.2574 0.4051 0.1636 0.4641 0.265 0.123 0.500 47.07 0.666 0.572 290 296 -0.505405 0.054054

ENSMUST00000020721 2766 0.2201 0.4239 0.2121 0.3376 0.214 0.072 0.462 49.19 0.635 0.598 903 921 -0.823996 0.032573

ENSMUST00000015749 1515 0.1568 0.4893 0.1050 0.4005 0.261 0.156 0.515 41.10 0.771 0.651 489 504 -0.245040 0.035714

ENSMUST00000020308 690 0.1789 0.4579 0.0847 0.4624 0.294 0.288 0.574 42.75 0.776 0.630 223 229 -0.240611 0.052402

ENSMUST00000018005 2115 0.2195 0.4466 0.1474 0.4499 0.247 0.098 0.471 46.93 0.707 0.578 680 704 -0.728409 0.055398

ENSMUST00000018549 963 0.2322 0.4232 0.1037 0.4122 0.201 0.112 0.457 47.54 0.711 0.635 311 320 -0.029687 0.043750

ENSMUST00000021843 696 0.3390 0.3277 0.3353 0.2813 0.229 0.014 0.432 54.60 0.468 0.470 220 231 -0.196537 0.138528

ENSMUST00000021670 6420 0.3676 0.2226 0.3838 0.2700 0.180 -0.048 0.383 54.01 0.392 0.508 2047 2139 -1.170.594 0.066854

ENSMUST00000019169 813 0.1823 0.3646 0.2018 0.4861 0.255 0.118 0.478 49.35 0.686 0.567 255 270 -0.383704 0.044444

ENSMUST00000018805 2943 0.1932 0.4796 0.1714 0.3973 0.253 0.143 0.496 47.92 0.702 0.575 942 980 -0.205306 0.061224

ENSMUST00000018066 639 0.3742 0.2699 0.3907 0.2571 0.224 0.036 0.435 54.40 0.400 0.399 200 212 -0.285377 0.089623

ENSMUST00000018544 1437 0.2255 0.4293 0.2225 0.4294 0.234 0.061 0.454 48.96 0.650 0.543 463 478 -0.737029 0.079498

ENSMUST00000014476 801 0.4184 0.2704 0.4022 0.2560 0.230 0.016 0.440 54.54 0.381 0.393 252 266 -0.579323 0.116541

ENSMUST00000016106 438 0.3280 0.2400 0.4196 0.2330 0.131 -0.146 0.317 57.43 0.380 0.453 142 145 -0.753793 0.110345

ENSMUST00000021595 1323 0.2752 0.3394 0.2991 0.3746 0.221 -0.003 0.412 58.58 0.543 0.489 427 440 -0.542500 0.045455

ENSMUST00000020964 327 0.2262 0.4762 0.2471 0.3205 0.222 0.072 0.467 48.16 0.619 0.522 105 108 -0.357407 0.074074

ENSMUST00000020930 1134 0.2123 0.4589 0.1927 0.4585 0.273 0.199 0.529 50.34 0.685 0.525 365 377 -0.415915 0.095491

ENSMUST00000015645 7293 0.3091 0.3412 0.2940 0.2974 0.202 0.013 0.429 55.80 0.510 0.515 2354 2430 -0.766379 0.058436

ENSMUST00000019386 2361 0.2416 0.3984 0.1787 0.4172 0.237 0.072 0.453 47.21 0.654 0.574 758 786 -0.293003 0.049618

ENSMUST00000020158 1911 0.2831 0.3678 0.2898 0.3406 0.243 0.064 0.459 55.30 0.546 0.512 614 636 -0.753931 0.061321

ENSMUST00000018431 684 0.2733 0.4000 0.2376 0.4335 0.237 -0.022 0.425 51.02 0.616 0.535 219 227 -1.029.075 0.057269

ENSMUST00000021817 1470 0.3245 0.3456 0.2486 0.3941 0.230 0.008 0.419 55.27 0.556 0.487 477 489 -0.399387 0.083845

ENSMUST00000017572 1269 0.2835 0.3489 0.2853 0.3556 0.252 0.061 0.449 56.78 0.544 0.487 412 422 -0.175829 0.066351

ENSMUST00000016668 531 0.2676 0.4225 0.2188 0.3739 0.223 0.115 0.479 42.28 0.609 0.483 169 176 -0.146591 0.056818

ENSMUST00000015017 774 0.2316 0.4421 0.2474 0.4063 0.230 0.065 0.461 49.44 0.638 0.528 254 257 -1.265.370 0.081712

ENSMUST00000014981 1404 0.2207 0.4106 0.1704 0.4564 0.268 0.184 0.523 45.13 0.685 0.578 444 467 -0.562527 0.087794

ENSMUST00000017799 870 0.2902 0.3973 0.2254 0.3861 0.221 -0.022 0.411 55.08 0.596 0.537 280 289 -0.349135 0.048443

ENSMUST00000014080 501 0.2269 0.4790 0.2689 0.3981 0.260 0.012 0.447 50.29 0.629 0.494 159 166 -0.569879 0.096386

ENSMUST00000021790 345 0.3400 0.2600 0.2247 0.3214 0.213 0.046 0.439 43.33 0.495 0.526 107 114 0.621930 0.114035

ENSMUST00000020756 2352 0.3905 0.2000 0.4334 0.2106 0.190 -0.062 0.380 52.27 0.322 0.427 761 783 -0.128480 0.057471

ENSMUST00000021659 1770 0.2734 0.3995 0.2061 0.3816 0.211 0.049 0.452 52.56 0.618 0.553 571 589 -0.968761 0.057725

ENSMUST00000020238 2409 0.3805 0.2850 0.3693 0.3012 0.246 -0.001 0.429 53.84 0.430 0.432 774 802 -0.719950 0.081047

ENSMUST00000015620 921 0.1720 0.4731 0.1406 0.3457 0.238 0.173 0.500 43.39 0.720 0.682 300 306 -0.089869 0.058824

ENSMUST00000018610 3435 0.2176 0.4855 0.1745 0.3973 0.240 0.106 0.477 47.45 0.687 0.552 1100 1144 -0.389948 0.098776

ENSMUST00000018389 723 0.4011 0.2513 0.3933 0.2375 0.199 -0.034 0.391 53.86 0.370 0.401 230 240 -0.120000 0.116667

ENSMUST00000021181 471 0.2500 0.4516 0.1493 0.3465 0.210 0.022 0.430 48.41 0.662 0.581 151 156 -0.244872 0.032051

ENSMUST00000021420 456 0.1532 0.4435 0.1667 0.4274 0.224 0.123 0.472 44.20 0.729 0.600 144 151 -0.124503 0.092715

ENSMUST00000020118 1146 0.1830 0.5142 0.1014 0.4690 0.282 0.249 0.559 44.81 0.768 0.569 370 381 -0.255906 0.083990

ENSMUST00000021065 672 0.1685 0.5163 0.1603 0.4196 0.287 0.124 0.493 44.11 0.735 0.562 211 223 0.292377 0.130045

ENSMUST00000015107 2157 0.3528 0.2509 0.3898 0.3236 0.190 -0.056 0.394 56.50 0.427 0.439 700 718 -0.826045 0.066852

ENSMUST00000018710 1530 0.2477 0.4014 0.1818 0.3385 0.202 0.078 0.440 51.10 0.624 0.563 489 509 0.558153 0.094303

ENSMUST00000016094 1215 0.1943 0.4713 0.1614 0.4339 0.265 0.171 0.515 44.17 0.711 0.590 388 404 -0.693564 0.089109

ENSMUST00000021062 1848 0.4718 0.1976 0.3510 0.2452 0.200 -0.054 0.396 48.75 0.338 0.438 594 615 -0.640976 0.087805

ENSMUST00000020681 1758 0.3291 0.3342 0.3105 0.3957 0.250 0.002 0.442 55.51 0.528 0.483 561 585 -1.237.607 0.082051

ENSMUST00000021933 1005 0.3200 0.3840 0.2348 0.3773 0.251 0.078 0.482 51.48 0.572 0.501 313 334 -0.520659 0.110778

ENSMUST00000021005 2745 0.2530 0.4040 0.2412 0.3411 0.232 0.071 0.457 51.41 0.596 0.542 879 914 -0.264770 0.083151

ENSMUST00000020350 2724 0.2719 0.3939 0.2411 0.3188 0.208 0.085 0.450 57.50 0.575 0.499 884 907 0.303749 0.081588

ENSMUST00000019911 1467 0.4572 0.2299 0.3869 0.2628 0.215 -0.098 0.382 54.41 0.358 0.423 469 488 -0.714549 0.100410

ENSMUST00000018143 2283 0.2179 0.4209 0.1758 0.4889 0.250 0.024 0.439 45.50 0.694 0.556 738 760 -0.679605 0.052632

ENSMUST00000020223 1554 0.3646 0.3595 0.2469 0.3038 0.241 0.075 0.455 50.94 0.513 0.480 497 517 -0.259188 0.056093

ENSMUST00000020775 270 0.3077 0.4923 0.1930 0.4200 0.308 -0.065 0.417 38.98 0.631 0.468 84 89 -0.456180 0.134831

ENSMUST00000020692 477 0.1969 0.5433 0.0909 0.3982 0.272 0.197 0.527 41.63 0.760 0.586 150 158 -0.236709 0.075949

ENSMUST00000017945 897 0.2687 0.4758 0.1810 0.3761 0.269 0.099 0.491 56.36 0.648 0.547 293 298 -0.765436 0.070470

ENSMUST00000017590 846 0.1916 0.5187 0.1587 0.4381 0.294 0.190 0.533 42.91 0.726 0.567 270 281 -0.514591 0.113879

ENSMUST00000020573 855 0.3056 0.3373 0.1822 0.3269 0.213 0.044 0.435 52.51 0.569 0.590 269 284 0.046127 0.077465

ENSMUST00000019422 1233 0.2283 0.4306 0.1843 0.3905 0.261 0.184 0.514 47.06 0.658 0.542 389 410 0.003658 0.078049

ENSMUST00000020153 2049 0.3962 0.2667 0.2987 0.2992 0.235 -0.005 0.418 55.39 0.442 0.476 661 682 -0.387097 0.064516

ENSMUST00000021681 1128 0.2014 0.4437 0.1716 0.4201 0.234 0.086 0.467 41.94 0.693 0.597 362 375 -0.664533 0.069333

ENSMUST00000020754 1032 0.3241 0.2222 0.4225 0.3408 0.225 -0.029 0.407 53.89 0.422 0.437 329 343 -0.802624 0.046647

ENSMUST00000021691 972 0.2362 0.4686 0.1488 0.3970 0.273 0.154 0.497 48.06 0.682 0.551 302 323 0.027554 0.157895

ENSMUST00000019354 681 0.3245 0.3113 0.2978 0.4136 0.263 0.016 0.426 52.77 0.528 0.465 216 226 -0.522566 0.053097

ENSMUST00000020315 3693 0.3624 0.2803 0.2866 0.3234 0.218 0.019 0.416 55.91 0.470 0.462 1196 1230 -0.019512 0.056098

ENSMUST00000016511 1356 0.2514 0.4364 0.1486 0.4578 0.275 0.137 0.494 47.46 0.684 0.553 427 451 -0.390687 0.104213

ENSMUST00000020650 396 0.2281 0.4825 0.1650 0.3021 0.252 0.149 0.488 55.13 0.661 0.565 127 131 0.333588 0.061069

ENSMUST00000020366 1323 0.2332 0.4531 0.2444 0.3147 0.228 0.091 0.466 53.91 0.612 0.480 423 440 0.078864 0.136364

ENSMUST00000020947 1005 0.2590 0.3309 0.2642 0.3597 0.204 0.067 0.446 58.35 0.563 0.546 325 334 -0.021856 0.068862

ENSMUST00000020513 2220 0.4240 0.1886 0.4268 0.2081 0.192 -0.089 0.358 50.37 0.308 0.410 717 739 -0.392422 0.069012

ENSMUST00000020984 3438 0.1817 0.5143 0.1304 0.4355 0.274 0.147 0.498 43.26 0.747 0.562 1082 1145 -0.015633 0.096943

ENSMUST00000021514 1149 0.3256 0.3189 0.3333 0.3140 0.214 -0.042 0.401 57.14 0.482 0.478 367 382 -0.468063 0.065445

ENSMUST00000020434 999 0.3580 0.2490 0.3730 0.2900 0.213 -0.030 0.398 57.96 0.417 0.475 314 332 -0.232229 0.105422

ENSMUST00000015346 1668 0.2410 0.3874 0.1982 0.4111 0.223 0.088 0.460 46.91 0.639 0.545 537 555 -0.508829 0.061261

ENSMUST00000015841 3927 0.3779 0.3285 0.2686 0.2871 0.237 0.019 0.436 54.43 0.481 0.498 1267 1308 -0.611239 0.074924

ENSMUST00000015481 885 0.1484 0.4648 0.0819 0.4955 0.226 0.175 0.509 41.27 0.801 0.660 287 294 -0.275850 0.081633

ENSMUST00000020792 828 0.2314 0.4236 0.2156 0.3447 0.241 0.072 0.459 47.18 0.627 0.550 268 275 -0.111273 0.072727

ENSMUST00000021550 1065 0.3663 0.2739 0.3309 0.2520 0.183 -0.044 0.379 52.18 0.420 0.479 348 354 -0.109040 0.067797

ENSMUST00000021438 1524 0.3333 0.2671 0.3394 0.2408 0.201 -0.011 0.421 52.93 0.421 0.502 501 507 -0.081657 0.041420

ENSMUST00000020920 2028 0.2070 0.4590 0.2257 0.3838 0.222 0.093 0.474 49.36 0.654 0.542 642 675 -0.649037 0.093333

ENSMUST00000021793 879 0.2511 0.4632 0.1768 0.3978 0.272 0.162 0.504 51.69 0.658 0.487 272 292 0.203767 0.167808

ENSMUST00000020878 399 0.3469 0.2755 0.3708 0.3095 0.206 -0.005 0.408 59.06 0.442 0.429 120 132 -0.228030 0.098485

ENSMUST00000020253 1197 0.3363 0.2883 0.3194 0.2902 0.195 -0.019 0.393 58.76 0.455 0.457 374 398 0.565075 0.130653

ENSMUST00000019896 858 0.2381 0.4000 0.2658 0.3768 0.216 -0.013 0.395 53.73 0.598 0.509 271 285 -0.272281 0.073684

ENSMUST00000021077 1068 0.1593 0.4889 0.1468 0.4664 0.245 0.084 0.469 44.56 0.754 0.611 350 355 -0.742254 0.030986

ENSMUST00000020958 2628 0.2154 0.4609 0.1846 0.3389 0.234 0.087 0.463 49.97 0.663 0.581 842 875 -0.128457 0.074286

ENSMUST00000020501 333 0.2000 0.3750 0.2410 0.5128 0.215 -0.046 0.406 44.69 0.660 0.542 106 110 -0.887273 0.054545

ENSMUST00000020049 723 0.2953 0.2550 0.3416 0.4141 0.247 0.109 0.498 53.97 0.515 0.492 233 240 -1.411.250 0.079167

ENSMUST00000021938 681 0.2292 0.3490 0.2833 0.3571 0.176 0.043 0.428 60.33 0.572 0.541 222 226 -0.107965 0.070796

ENSMUST00000021447 1404 0.3439 0.2832 0.3866 0.2936 0.210 0.012 0.415 57.13 0.430 0.420 451 467 -0.300428 0.107066

ENSMUST00000018744 1437 0.2842 0.3747 0.2194 0.3858 0.255 0.061 0.459 52.08 0.593 0.526 464 478 -0.261088 0.081590

ENSMUST00000017151 1548 0.1651 0.4953 0.1439 0.4145 0.248 0.172 0.512 43.85 0.743 0.608 502 515 -0.205049 0.085437

ENSMUST00000021164 696 0.2529 0.3736 0.2040 0.3655 0.177 0.088 0.464 46.64 0.617 0.595 222 231 -0.933333 0.017316

ENSMUST00000019135 2157 0.2667 0.4035 0.2034 0.3524 0.216 0.042 0.428 52.42 0.612 0.554 698 718 -0.377994 0.051532

ENSMUST00000021806 723 0.4011 0.2582 0.3842 0.2606 0.231 -0.042 0.394 58.62 0.390 0.439 231 240 -0.275000 0.112500

ENSMUST00000021611 3111 0.2807 0.3863 0.2705 0.3559 0.234 0.054 0.452 54.01 0.567 0.499 1003 1036 -0.442181 0.092664

ENSMUST00000021197 1368 0.3441 0.3412 0.2582 0.4000 0.240 0.018 0.435 52.72 0.542 0.464 428 455 -0.405934 0.112088

ENSMUST00000020377 1959 0.2047 0.4811 0.1468 0.3358 0.214 0.070 0.462 44.52 0.698 0.633 639 652 -0.557976 0.050613

ENSMUST00000020586 1662 0.1926 0.4372 0.2053 0.3914 0.244 0.185 0.510 49.05 0.667 0.532 522 553 0.496383 0.132007

ENSMUST00000018851 13935 0.2283 0.4317 0.1927 0.4366 0.262 0.122 0.483 49.92 0.666 0.535 4446 4644 -0.339212 0.082472

ENSMUST00000018186 906 0.2082 0.4816 0.1435 0.4335 0.232 0.091 0.457 49.10 0.713 0.555 289 301 -0.196013 0.096346

ENSMUST00000021787 1296 0.1758 0.5000 0.2097 0.3481 0.245 0.207 0.532 52.02 0.687 0.579 425 431 -0.573782 0.053364

ENSMUST00000021287 1863 0.2903 0.3996 0.2234 0.3487 0.240 0.086 0.473 51.95 0.581 0.508 594 620 -0.071935 0.080645

ENSMUST00000019362 2211 0.2640 0.4400 0.1495 0.3196 0.205 0.076 0.459 48.95 0.643 0.599 706 736 -0.540353 0.063859

ENSMUST00000021297 588 0.2971 0.3478 0.3272 0.3245 0.261 0.082 0.471 61.00 0.508 0.491 191 195 -0.474359 0.051282

ENSMUST00000018184 843 0.2100 0.4250 0.1674 0.4773 0.234 0.025 0.430 48.19 0.704 0.582 270 280 -0.803928 0.082143

ENSMUST00000015267 825 0.2387 0.4685 0.1814 0.3568 0.272 0.148 0.496 47.84 0.654 0.536 260 274 -0.049635 0.087591

ENSMUST00000020298 315 0.2222 0.4444 0.2500 0.3971 0.277 0.134 0.505 45.85 0.636 0.529 99 104 -0.358654 0.105769

ENSMUST00000021757 1725 0.3297 0.3537 0.3039 0.3000 0.232 0.045 0.445 54.72 0.501 0.476 551 574 -0.387805 0.097561

ENSMUST00000020381 1527 0.3532 0.2673 0.3185 0.3108 0.171 -0.071 0.374 55.63 0.457 0.490 497 508 -0.889764 0.055118

ENSMUST00000020411 2697 0.2193 0.4545 0.1591 0.4254 0.245 0.120 0.486 45.98 0.696 0.577 867 898 -0.636748 0.076837

ENSMUST00000019118 2889 0.2251 0.4225 0.1514 0.4912 0.255 0.082 0.470 47.12 0.707 0.581 920 962 -0.658420 0.075884

ENSMUST00000015664 990 0.3108 0.3865 0.2331 0.3571 0.264 0.063 0.468 51.11 0.571 0.504 310 329 -0.487538 0.106383

ENSMUST00000015903 435 0.2541 0.4672 0.1685 0.3671 0.280 0.174 0.508 51.32 0.652 0.505 132 144 0.800694 0.194444

ENSMUST00000021243 969 0.2741 0.4296 0.1240 0.3919 0.271 0.159 0.498 47.26 0.661 0.513 307 322 0.582919 0.133540

ENSMUST00000020768 762 0.2500 0.4415 0.1487 0.4389 0.252 0.029 0.437 49.41 0.681 0.567 238 253 -0.543478 0.075099

ENSMUST00000019907 1266 0.3333 0.3393 0.3038 0.3090 0.229 0.060 0.453 55.95 0.499 0.467 413 421 -0.587173 0.054632

ENSMUST00000015611 882 0.2241 0.3693 0.2155 0.3921 0.208 0.047 0.433 49.91 0.631 0.606 282 293 -0.177474 0.061433

ENSMUST00000020102 1806 0.2901 0.3712 0.2539 0.3325 0.220 0.000 0.423 53.76 0.552 0.502 572 601 0.185857 0.118136

ENSMUST00000021471 837 0.3868 0.2170 0.3726 0.2736 0.205 -0.046 0.389 53.65 0.385 0.472 262 278 -0.209352 0.104317

ENSMUST00000020440 288 0.1127 0.5634 0.0909 0.5323 0.274 0.175 0.540 42.36 0.839 0.614 87 95 -0.526316 0.063158

ENSMUST00000018311 1341 0.2310 0.4864 0.1268 0.3889 0.253 0.160 0.502 47.67 0.703 0.565 434 446 0.006951 0.118834

ENSMUST00000021864 861 0.3750 0.2545 0.3364 0.3107 0.221 0.039 0.434 55.10 0.434 0.472 279 286 -0.358042 0.083916

ENSMUST00000020851 828 0.2646 0.3049 0.2464 0.4221 0.171 -0.080 0.366 55.14 0.580 0.561 262 275 -0.370545 0.116364

ENSMUST00000018449 7008 0.2941 0.4063 0.1915 0.4118 0.251 0.082 0.460 50.84 0.616 0.510 2214 2335 -0.468351 0.114347

ENSMUST00000020504 381 0.3663 0.3663 0.2151 0.3375 0.248 -0.011 0.413 53.28 0.529 0.519 121 126 -0.153968 0.055556

ENSMUST00000020484 1500 0.2668 0.3666 0.2883 0.3438 0.242 0.075 0.465 56.95 0.551 0.507 486 498 -0.168675 0.082329

ENSMUST00000020145 1296 0.3072 0.4174 0.1847 0.3759 0.242 0.056 0.446 52.53 0.607 0.503 417 431 -0.281671 0.102088

ENSMUST00000021959 1035 0.3066 0.3540 0.1954 0.3880 0.241 0.053 0.444 53.89 0.590 0.552 329 344 -0.194186 0.087209

ENSMUST00000020980 1173 0.2847 0.4203 0.2140 0.3893 0.237 0.055 0.453 57.13 0.609 0.502 371 390 -0.247436 0.112821

ENSMUST00000019649 918 0.1778 0.5911 0.0664 0.4868 0.380 0.304 0.591 31.58 0.811 0.572 301 305 -0.492131 0.042623

ENSMUST00000015049 669 0.4368 0.2931 0.4236 0.1985 0.231 -0.074 0.419 53.75 0.363 0.398 215 222 -0.837387 0.135135

ENSMUST00000020382 684 0.2875 0.3312 0.3699 0.3459 0.238 0.046 0.454 50.72 0.495 0.436 218 227 -0.616300 0.105727

ENSMUST00000014673 909 0.3038 0.3154 0.3243 0.2917 0.209 -0.010 0.391 51.55 0.478 0.453 289 302 0.634437 0.129139

ENSMUST00000014447 1566 0.2533 0.4449 0.1582 0.3317 0.234 0.126 0.472 48.46 0.650 0.601 515 521 -0.406526 0.055662

ENSMUST00000019726 1314 0.1976 0.4225 0.1622 0.4345 0.251 0.097 0.476 47.12 0.702 0.600 420 437 -0.363844 0.036613

ENSMUST00000020948 4863 0.3196 0.3072 0.3174 0.3156 0.199 0.017 0.417 53.67 0.483 0.455 1554 1620 0.065556 0.099383

ENSMUST00000020941 792 0.2857 0.3622 0.2879 0.3455 0.212 0.060 0.460 59.41 0.548 0.515 250 263 -1.036.882 0.060837

ENSMUST00000020317 747 0.2537 0.2985 0.3417 0.3497 0.172 -0.019 0.407 59.86 0.510 0.507 243 248 -0.403226 0.060484

ENSMUST00000019128 921 0.3014 0.2877 0.3306 0.3617 0.234 0.030 0.471 61.00 0.502 0.521 295 306 -0.789216 0.078431

ENSMUST00000019517 1272 0.3684 0.2941 0.3191 0.3322 0.225 -0.021 0.407 56.91 0.467 0.441 405 423 -0.193380 0.085106

ENSMUST00000021715 1050 0.1536 0.4437 0.1825 0.4222 0.217 0.190 0.510 44.54 0.716 0.602 341 349 0.009456 0.051576

ENSMUST00000021040 1596 0.3484 0.2840 0.3209 0.2982 0.237 0.072 0.451 56.61 0.453 0.469 519 531 0.016949 0.037665

ENSMUST00000019701 1359 0.2327 0.4155 0.1549 0.4234 0.262 0.146 0.501 44.72 0.682 0.604 443 452 -0.342478 0.064159

ENSMUST00000014830 1281 0.2135 0.4663 0.1347 0.3707 0.257 0.123 0.480 46.99 0.702 0.599 406 426 -0.065962 0.079812

ENSMUST00000021416 1026 0.3640 0.2797 0.4218 0.2016 0.200 -0.024 0.402 51.87 0.372 0.446 336 341 -0.584164 0.046921

ENSMUST00000020485 1056 0.2883 0.3808 0.2811 0.3304 0.232 0.084 0.456 52.43 0.547 0.491 333 351 -0.203134 0.096866

ENSMUST00000021141 1200 0.2677 0.4400 0.1661 0.4060 0.276 0.116 0.484 51.51 0.650 0.533 386 399 -0.104261 0.112782

ENSMUST00000015889 1227 0.1947 0.4356 0.1917 0.4329 0.233 0.111 0.482 45.97 0.688 0.586 398 408 -0.941176 0.046569

ENSMUST00000019257 1656 0.2229 0.4177 0.1902 0.3727 0.216 0.064 0.445 49.30 0.653 0.611 542 551 -0.388022 0.041742

ENSMUST00000020608 930 0.3690 0.3175 0.3066 0.3015 0.255 0.054 0.450 54.17 0.470 0.478 298 309 -0.428479 0.113269

ENSMUST00000019876 1143 0.2873 0.3927 0.2360 0.4498 0.309 0.087 0.486 48.84 0.608 0.496 362 380 -0.755263 0.126316

ENSMUST00000019611 1857 0.2899 0.3214 0.2063 0.4153 0.206 0.054 0.440 50.87 0.594 0.563 596 618 -0.486569 0.059871

ENSMUST00000019043 990 0.2520 0.4268 0.2427 0.3641 0.244 0.101 0.484 49.33 0.605 0.522 304 329 -0.694529 0.069909

ENSMUST00000018630 1080 0.2020 0.4646 0.1000 0.4409 0.290 0.209 0.542 40.08 0.748 0.643 349 359 -0.223677 0.072423

ENSMUST00000021259 3327 0.2225 0.3797 0.1913 0.3855 0.214 0.101 0.457 49.78 0.644 0.598 1064 1108 -0.024729 0.063177

ENSMUST00000016086 2811 0.1750 0.4600 0.1821 0.4481 0.240 0.070 0.457 45.77 0.712 0.564 900 936 -0.236966 0.080128

ENSMUST00000021313 696 0.1534 0.5238 0.1130 0.4601 0.267 0.188 0.507 42.66 0.780 0.589 223 231 -0.082251 0.077922

ENSMUST00000019050 1614 0.2500 0.3892 0.2153 0.4005 0.225 0.052 0.445 51.37 0.625 0.547 515 537 -0.403538 0.100559

ENSMUST00000020990 708 0.1180 0.5506 0.0884 0.5085 0.273 0.234 0.556 38.77 0.836 0.633 225 235 -0.799149 0.093617

ENSMUST00000020008 1368 0.3169 0.3169 0.2630 0.3793 0.234 0.072 0.462 56.16 0.540 0.527 446 455 -0.549011 0.035165

ENSMUST00000019999 6513 0.2385 0.4157 0.2176 0.3755 0.250 0.114 0.480 50.48 0.628 0.544 2090 2170 -0.221014 0.061751

ENSMUST00000021866 1704 0.3477 0.3261 0.3621 0.3045 0.236 0.012 0.439 52.49 0.463 0.439 544 567 -0.813757 0.065256

ENSMUST00000015797 1470 0.2721 0.3627 0.2541 0.3547 0.211 0.076 0.444 54.83 0.564 0.481 468 489 0.249080 0.122699

ENSMUST00000021950 2121 0.2803 0.3902 0.2027 0.3862 0.265 0.052 0.455 48.07 0.614 0.591 692 706 -0.747450 0.050992

ENSMUST00000018482 1944 0.1982 0.4305 0.1985 0.4637 0.247 0.051 0.451 47.67 0.691 0.581 625 647 -0.972334 0.041731

ENSMUST00000020886 1476 0.3594 0.2891 0.3641 0.2895 0.236 0.004 0.425 53.58 0.436 0.430 482 491 -0.451120 0.103870

ENSMUST00000017454 480 0.2955 0.3409 0.2906 0.3391 0.202 0.069 0.446 56.27 0.535 0.486 157 159 -0.569182 0.081761

ENSMUST00000021028 2364 0.2563 0.4748 0.1525 0.4045 0.299 0.132 0.503 46.78 0.675 0.551 765 787 -0.275858 0.073698

ENSMUST00000020630 2529 0.4033 0.2446 0.3394 0.3237 0.246 -0.060 0.393 53.89 0.428 0.448 817 842 -0.567815 0.068884

ENSMUST00000020478 2169 0.3819 0.2647 0.3525 0.2266 0.207 -0.027 0.405 55.29 0.396 0.471 692 722 -0.386981 0.091413

ENSMUST00000018755 993 0.2286 0.4429 0.1705 0.3614 0.220 0.101 0.472 48.79 0.665 0.607 322 330 -0.515455 0.048485

ENSMUST00000014747 1032 0.1860 0.4912 0.1434 0.3731 0.228 0.156 0.503 45.64 0.723 0.647 332 343 -0.651021 0.078717

ENSMUST00000014777 315 0.2500 0.3929 0.2532 0.3289 0.188 -0.046 0.384 61.00 0.586 0.545 99 104 -0.382692 0.076923

ENSMUST00000016338 879 0.2641 0.4199 0.2613 0.2950 0.222 0.054 0.447 55.64 0.567 0.482 275 292 0.206507 0.089041

ENSMUST00000021333 1446 0.0943 0.6030 0.0637 0.4615 0.277 0.256 0.573 37.32 0.869 0.658 466 481 -0.727235 0.066528

ENSMUST00000014927 3546 0.2527 0.3652 0.2115 0.3917 0.216 0.076 0.451 52.38 0.617 0.582 1141 1181 -0.385436 0.066892

ENSMUST00000020997 1023 0.3659 0.2355 0.3911 0.2561 0.179 -0.045 0.394 55.53 0.382 0.449 335 340 -0.311765 0.082353

ENSMUST00000021362 1221 0.3855 0.2952 0.3564 0.2252 0.221 0.022 0.439 53.16 0.410 0.466 383 406 -0.417241 0.123153

ENSMUST00000015486 1341 0.2174 0.4810 0.1843 0.3579 0.263 0.186 0.511 50.03 0.668 0.515 425 446 0.515695 0.134529

ENSMUST00000019859 2100 0.2450 0.4036 0.2106 0.4238 0.258 0.080 0.466 51.21 0.640 0.544 670 699 -0.622032 0.068670

ENSMUST00000015950 726 0.2408 0.4136 0.1935 0.3520 0.245 0.056 0.460 51.96 0.634 0.574 224 241 -0.057676 0.070539

ENSMUST00000017144 300 0.4308 0.2154 0.3500 0.3247 0.194 -0.185 0.337 50.00 0.411 0.438 95 99 -0.923232 0.080808

ENSMUST00000016491 2280 0.2075 0.4635 0.1550 0.3972 0.226 0.098 0.471 49.00 0.698 0.580 731 759 -0.349802 0.092227

ENSMUST00000021888 1032 0.4457 0.2246 0.3766 0.2108 0.208 -0.002 0.422 50.75 0.339 0.424 322 343 -0.329446 0.125364

ENSMUST00000015605 2121 0.2197 0.4200 0.1492 0.4063 0.200 0.063 0.436 46.72 0.689 0.618 684 706 -0.484419 0.041076

ENSMUST00000021851 1533 0.3367 0.3063 0.3805 0.2460 0.240 0.085 0.476 52.06 0.431 0.452 494 510 -0.774314 0.062745

ENSMUST00000019721 1239 0.2896 0.3910 0.2458 0.3333 0.245 0.040 0.434 54.16 0.568 0.496 396 412 -0.222816 0.092233

ENSMUST00000020208 4200 0.3327 0.3206 0.2941 0.3257 0.228 0.011 0.427 54.99 0.502 0.489 1364 1399 -0.703860 0.053610

ENSMUST00000020699 996 0.2509 0.4509 0.1379 0.3924 0.290 0.198 0.522 48.16 0.674 0.570 322 331 0.117523 0.084592

ENSMUST00000021331 1716 0.4151 0.2839 0.3211 0.2395 0.238 0.030 0.433 56.49 0.408 0.452 547 571 -0.054291 0.098074

ENSMUST00000020843 7038 0.3376 0.3333 0.2810 0.3133 0.240 0.065 0.451 53.84 0.501 0.484 2243 2345 -0.239403 0.086567

ENSMUST00000020375 1287 0.3009 0.3363 0.2404 0.3801 0.236 0.051 0.451 53.42 0.563 0.515 419 428 -0.386215 0.065421

ENSMUST00000019986 9369 0.4275 0.2251 0.3859 0.2330 0.203 -0.048 0.394 52.17 0.353 0.422 3040 3122 -0.602146 0.067265

ENSMUST00000015578 747 0.2500 0.3724 0.2690 0.3631 0.219 0.037 0.433 53.39 0.575 0.511 240 248 -0.188710 0.064516

ENSMUST00000019697 1860 0.2505 0.3137 0.2063 0.4500 0.225 0.130 0.472 51.92 0.626 0.576 604 619 -0.407755 0.027464

ENSMUST00000015394 1419 0.4010 0.3393 0.2898 0.2432 0.249 0.038 0.444 50.14 0.451 0.459 450 472 -0.422246 0.144068

ENSMUST00000021046 2790 0.4282 0.2575 0.3050 0.2826 0.202 -0.099 0.380 51.16 0.415 0.477 902 929 -0.826265 0.069968

ENSMUST00000020531 429 0.2364 0.5364 0.0588 0.4362 0.341 0.257 0.568 43.78 0.758 0.556 132 142 -0.053521 0.098592

ENSMUST00000016400 921 0.2369 0.5020 0.1232 0.3979 0.283 0.192 0.542 47.90 0.703 0.568 286 306 -0.381373 0.101307

ENSMUST00000018353 1464 0.3052 0.3256 0.2973 0.3917 0.232 0.009 0.431 56.32 0.532 0.482 459 487 -0.603696 0.067762

ENSMUST00000018739 1371 0.3081 0.4054 0.2669 0.2591 0.210 0.050 0.436 53.47 0.531 0.480 429 456 -0.060746 0.120614

ENSMUST00000018871 573 0.1921 0.4636 0.2595 0.3846 0.228 0.093 0.464 53.38 0.656 0.558 183 190 -0.314211 0.105263

ENSMUST00000014698 834 0.2632 0.3971 0.2233 0.4000 0.249 0.155 0.504 49.70 0.615 0.531 262 277 -0.398556 0.104693

ENSMUST00000021653 1299 0.2107 0.4579 0.1381 0.4309 0.245 0.148 0.495 47.29 0.711 0.599 418 432 -0.214352 0.078704

ENSMUST00000020970 1089 0.2831 0.3971 0.2090 0.4246 0.265 0.167 0.514 49.46 0.618 0.514 348 362 -0.377348 0.104972

ENSMUST00000014445 378 0.2667 0.4111 0.2075 0.3922 0.245 0.033 0.447 47.40 0.626 0.560 123 125 -0.513600 0.056000

ENSMUST00000017488 1437 0.2706 0.4721 0.2035 0.3364 0.239 0.072 0.473 53.41 0.627 0.552 461 478 -0.778870 0.133891

ENSMUST00000021285 711 0.2816 0.2931 0.2835 0.4148 0.201 0.006 0.408 52.71 0.544 0.494 228 236 -0.457627 0.029661

ENSMUST00000020263 1041 0.4141 0.1953 0.4051 0.2242 0.157 -0.125 0.379 50.43 0.330 0.483 327 346 -0.719364 0.118497

ENSMUST00000019068 1992 0.2649 0.4223 0.1814 0.4000 0.248 0.152 0.498 51.55 0.641 0.542 630 663 -0.211463 0.104072

ENSMUST00000021116 2433 0.2231 0.4754 0.1625 0.3864 0.254 0.123 0.497 46.52 0.688 0.600 795 810 -0.609630 0.065432

ENSMUST00000020982 1509 0.2795 0.4072 0.1965 0.3101 0.223 0.105 0.473 51.55 0.597 0.568 484 502 -0.374900 0.043825

ENSMUST00000018429 2802 0.2468 0.4078 0.2017 0.3873 0.221 0.086 0.459 50.51 0.631 0.546 895 933 -0.259593 0.100750

ENSMUST00000018988 966 0.2460 0.4206 0.2033 0.3793 0.273 0.074 0.461 54.92 0.634 0.525 306 321 -0.330218 0.068536

ENSMUST00000018156 579 0.1875 0.4500 0.1875 0.4296 0.274 0.101 0.465 40.56 0.695 0.571 187 192 -0.102604 0.088542

ENSMUST00000016897 1272 0.1657 0.5392 0.1767 0.4286 0.270 0.163 0.512 47.57 0.733 0.531 404 423 -0.509693 0.113475

ENSMUST00000021506 1257 0.2291 0.4303 0.2188 0.4020 0.247 0.084 0.465 45.55 0.642 0.517 402 418 -0.055742 0.090909

ENSMUST00000016977 432 0.3964 0.2432 0.3178 0.3333 0.198 -0.050 0.391 61.00 0.435 0.450 138 143 -0.328671 0.090909

ENSMUST00000021060 1380 0.2803 0.3288 0.2345 0.3826 0.186 0.055 0.440 59.18 0.576 0.533 441 459 -0.267320 0.084967

ENSMUST00000019226 972 0.2368 0.4398 0.1188 0.4280 0.271 0.138 0.492 45.99 0.702 0.591 315 323 0.111765 0.071207

ENSMUST00000021609 2190 0.3211 0.3172 0.3122 0.3287 0.220 0.049 0.455 54.90 0.501 0.486 707 729 -0.869685 0.039781

ENSMUST00000017086 2436 0.1876 0.5008 0.1529 0.4042 0.280 0.200 0.531 44.94 0.724 0.594 778 811 -0.282491 0.101110

ENSMUST00000020986 822 0.2857 0.4143 0.2836 0.3207 0.230 -0.007 0.426 58.37 0.555 0.487 263 273 -0.390476 0.091575

ENSMUST00000021166 573 0.1608 0.4825 0.0946 0.5282 0.228 -0.006 0.409 42.50 0.796 0.602 181 190 -0.281579 0.084211

ENSMUST00000020449 3225 0.2841 0.3671 0.2868 0.3553 0.243 0.071 0.464 56.26 0.552 0.503 1043 1074 -0.539106 0.079143

ENSMUST00000020258 3150 0.4274 0.2255 0.3721 0.2607 0.197 -0.060 0.381 51.47 0.366 0.422 1021 1049 -0.112298 0.103908

ENSMUST00000017841 1059 0.1955 0.5113 0.2000 0.4016 0.276 0.062 0.456 49.12 0.692 0.543 338 352 -0.419034 0.090909

ENSMUST00000020034 969 0.3760 0.2149 0.3808 0.2645 0.183 -0.040 0.389 55.47 0.379 0.436 306 322 -0.127640 0.055901

ENSMUST00000020012 1578 0.3249 0.2857 0.3195 0.2949 0.220 -0.009 0.411 54.11 0.468 0.491 511 525 -0.090095 0.074286

ENSMUST00000015435 1344 0.3201 0.4164 0.2025 0.3702 0.288 0.086 0.472 47.96 0.588 0.493 432 447 -0.305593 0.100671

ENSMUST00000020702 879 0.1535 0.5311 0.1384 0.4118 0.283 0.269 0.578 40.90 0.763 0.644 287 292 -0.594178 0.058219

ENSMUST00000021784 1353 0.2507 0.4113 0.2400 0.3896 0.236 0.091 0.471 49.59 0.614 0.544 433 450 -0.675333 0.102222

ENSMUST00000019992 5451 0.3030 0.3784 0.2815 0.3160 0.227 0.024 0.439 54.08 0.537 0.494 1772 1816 -0.412831 0.080947

ENSMUST00000019638 975 0.2852 0.4063 0.1818 0.4000 0.248 0.036 0.443 51.44 0.621 0.529 309 324 -0.043519 0.080247

ENSMUST00000016569 4341 0.3937 0.2498 0.3713 0.2836 0.216 -0.029 0.399 53.27 0.402 0.430 1397 1446 -0.560650 0.059474

ENSMUST00000016680 2280 0.1996 0.4278 0.2513 0.4436 0.241 0.128 0.490 52.28 0.650 0.507 726 759 -0.433465 0.081686

ENSMUST00000016463 897 0.3004 0.4280 0.1982 0.3168 0.313 0.158 0.519 50.37 0.589 0.522 285 298 0.019463 0.127517

ENSMUST00000019290 972 0.1583 0.6151 0.1139 0.3241 0.309 0.297 0.599 40.69 0.772 0.574 312 323 -0.162539 0.108359

ENSMUST00000014063 744 0.2788 0.3942 0.1913 0.3543 0.206 0.043 0.435 55.61 0.608 0.559 237 247 -0.175709 0.076923

ENSMUST00000021346 2295 0.2543 0.4631 0.1964 0.3478 0.248 0.167 0.507 52.77 0.635 0.500 742 764 0.050785 0.108639

ENSMUST00000021467 1161 0.3616 0.2769 0.3042 0.3213 0.185 -0.030 0.401 51.73 0.468 0.490 372 386 -0.854404 0.108808

ENSMUST00000020322 3189 0.2259 0.4531 0.2354 0.3848 0.232 0.081 0.469 49.37 0.638 0.523 1028 1062 -0.733522 0.064972

ENSMUST00000021016 1677 0.1698 0.5401 0.1628 0.3830 0.241 0.124 0.494 46.52 0.732 0.581 534 558 -0.664516 0.050179

ENSMUST00000020749 2184 0.4096 0.1974 0.4418 0.2146 0.216 -0.006 0.412 50.84 0.316 0.421 702 727 -0.371940 0.053645

ENSMUST00000020002 246 0.4242 0.1818 0.3607 0.3103 0.207 -0.038 0.375 61.00 0.375 0.432 80 81 0.002469 0.049383

ENSMUST00000019917 732 0.3494 0.3072 0.3913 0.3218 0.262 0.001 0.435 58.29 0.451 0.431 237 243 -0.834568 0.074074

ENSMUST00000019281 333 0.3293 0.2683 0.3000 0.3690 0.179 0.039 0.430 57.56 0.495 0.530 107 110 -1.003.636 0.063636

ENSMUST00000013995 6933 0.2613 0.4145 0.2236 0.3503 0.234 0.095 0.465 51.58 0.605 0.521 2206 2310 -0.069654 0.103030

ENSMUST00000020255 1836 0.3222 0.3352 0.2566 0.2791 0.206 0.022 0.421 57.65 0.504 0.477 585 611 0.536497 0.127660

ENSMUST00000019183 1617 0.2505 0.3758 0.2075 0.3539 0.239 0.127 0.472 54.58 0.612 0.589 523 538 0.023048 0.065056

ENSMUST00000020899 1446 0.2826 0.4029 0.1861 0.3717 0.260 0.074 0.468 52.55 0.614 0.552 472 481 -0.129730 0.054054

ENSMUST00000017867 525 0.2128 0.4752 0.2426 0.3083 0.236 0.113 0.491 50.41 0.632 0.580 171 174 -0.293678 0.011494

ENSMUST00000016781 561 0.2207 0.3931 0.2246 0.4436 0.299 0.089 0.469 51.20 0.648 0.523 179 186 0.001075 0.102151

ENSMUST00000019924 1020 0.2749 0.3368 0.3210 0.2357 0.200 -0.026 0.410 55.75 0.489 0.544 327 339 -0.208555 0.047198

ENSMUST00000018470 741 0.2765 0.4235 0.2553 0.3876 0.333 0.181 0.538 46.42 0.597 0.496 236 246 -0.693496 0.077236

ENSMUST00000015866 3336 0.2855 0.3572 0.2993 0.3529 0.234 0.070 0.453 52.58 0.541 0.473 1063 1111 -0.227723 0.064806

ENSMUST00000018698 1080 0.2086 0.3477 0.2045 0.4066 0.141 -0.048 0.385 49.06 0.643 0.661 356 359 -0.940669 0.052925

ENSMUST00000020085 444 0.2645 0.3636 0.3786 0.2796 0.216 0.043 0.440 61.00 0.496 0.476 141 147 -0.357143 0.102041

ENSMUST00000019684 681 0.2649 0.3946 0.1813 0.3729 0.186 -0.001 0.412 45.35 0.629 0.600 221 226 -0.881858 0.039823

ENSMUST00000020340 1968 0.1737 0.4954 0.1275 0.4209 0.281 0.229 0.548 41.72 0.748 0.616 626 655 -0.366565 0.093130

ENSMUST00000020185 1641 0.3263 0.3474 0.2783 0.3228 0.243 0.047 0.442 53.94 0.518 0.490 523 546 -0.419963 0.093407

ENSMUST00000016309 930 0.2664 0.4562 0.1991 0.2828 0.263 0.148 0.492 53.79 0.605 0.519 299 309 0.401294 0.142395

ENSMUST00000020361 675 0.2299 0.4332 0.1130 0.3964 0.299 0.250 0.550 46.41 0.701 0.616 211 224 -0.055357 0.075893

ENSMUST00000020999 2391 0.2405 0.4256 0.1690 0.4521 0.253 0.080 0.468 46.89 0.678 0.559 767 796 -0.759799 0.051508

ENSMUST00000020113 1431 0.3223 0.3427 0.2943 0.2918 0.241 0.080 0.464 56.58 0.501 0.480 459 476 -0.324790 0.079832

ENSMUST00000018466 930 0.2375 0.4208 0.2317 0.3755 0.270 0.119 0.490 49.79 0.625 0.534 304 309 -0.554369 0.061489

ENSMUST00000021273 351 0.2088 0.4945 0.1170 0.4167 0.264 0.096 0.464 54.64 0.727 0.580 110 116 0.004310 0.051724

ENSMUST00000016088 990 0.2138 0.4964 0.1627 0.3565 0.261 0.099 0.470 47.06 0.687 0.546 319 329 0.213374 0.094225

ENSMUST00000016081 1119 0.2353 0.4221 0.2000 0.3937 0.232 0.031 0.441 50.14 0.644 0.536 365 372 -0.262366 0.051075

ENSMUST00000015893 747 0.3512 0.3333 0.3090 0.4353 0.239 -0.065 0.398 54.85 0.533 0.477 244 248 -1.169.355 0.040323

ENSMUST00000021425 1017 0.2702 0.4194 0.2414 0.3592 0.266 0.037 0.450 52.83 0.596 0.517 322 338 -0.512426 0.094675

ENSMUST00000021329 639 0.2115 0.4551 0.1863 0.4662 0.225 0.101 0.473 47.27 0.690 0.520 203 212 -0.564151 0.051887

ENSMUST00000021082 603 0.1447 0.4843 0.1351 0.5108 0.249 0.191 0.513 43.28 0.775 0.595 191 200 -0.353000 0.085000

ENSMUST00000021937 885 0.1991 0.4213 0.2489 0.4439 0.258 0.098 0.493 52.29 0.655 0.550 284 294 -0.845918 0.051020

ENSMUST00000021283 2616 0.1818 0.4943 0.1750 0.3735 0.238 0.166 0.505 46.17 0.705 0.584 850 871 -0.313433 0.066590

ENSMUST00000020071 2298 0.2923 0.4107 0.2522 0.2921 0.238 0.094 0.481 53.43 0.559 0.516 744 765 -0.587582 0.084967

ENSMUST00000020004 615 0.3232 0.2683 0.4345 0.2647 0.259 0.049 0.444 52.07 0.408 0.436 196 204 -0.296569 0.093137

ENSMUST00000021227 975 0.2590 0.3028 0.3182 0.3665 0.191 -0.038 0.379 54.57 0.530 0.521 317 324 -0.509259 0.061728

ENSMUST00000015234 570 0.1575 0.5616 0.1736 0.3571 0.290 0.191 0.533 46.80 0.733 0.573 180 189 -0.297884 0.105820

ENSMUST00000020885 732 0.4078 0.3204 0.2299 0.2625 0.268 0.033 0.440 55.91 0.466 0.458 232 243 0.327160 0.139918

ENSMUST00000021201 4134 0.3237 0.3298 0.2832 0.3141 0.248 0.081 0.466 56.25 0.507 0.500 1333 1377 -0.398039 0.083515

ENSMUST00000020578 474 0.2931 0.3448 0.2787 0.3534 0.262 0.088 0.477 59.05 0.544 0.529 149 157 -0.538854 0.095541

ENSMUST00000021168 1719 0.2126 0.4337 0.1542 0.4272 0.196 0.052 0.442 43.87 0.697 0.592 552 572 -0.476923 0.101399

ENSMUST00000020190 1503 0.3453 0.3094 0.2617 0.3422 0.221 -0.020 0.407 53.37 0.506 0.491 484 500 -0.186000 0.124000

ENSMUST00000021641 3246 0.2005 0.4951 0.1779 0.3951 0.280 0.137 0.509 48.60 0.698 0.553 1042 1081 -0.694265 0.053654

ENSMUST00000019229 807 0.3050 0.2900 0.3028 0.3493 0.220 0.052 0.446 53.78 0.508 0.515 258 268 -0.600746 0.041045

ENSMUST00000017604 579 0.2157 0.4052 0.1718 0.4040 0.208 0.024 0.418 46.45 0.668 0.599 184 192 0.031250 0.083333

ENSMUST00000021284 1815 0.1356 0.5789 0.0926 0.4763 0.332 0.288 0.595 37.73 0.818 0.626 583 604 -0.617550 0.079470

ENSMUST00000021519 741 0.1728 0.4293 0.2010 0.4427 0.220 0.088 0.467 50.19 0.696 0.598 240 246 -0.619512 0.077236

ENSMUST00000021684 1503 0.2108 0.4447 0.1289 0.4611 0.265 0.158 0.497 46.92 0.723 0.581 477 500 -0.212600 0.096000

ENSMUST00000020640 954 0.2644 0.4789 0.1581 0.3163 0.308 0.222 0.552 51.17 0.645 0.540 299 317 -0.251420 0.085174

ENSMUST00000020552 912 0.1195 0.4263 0.1230 0.5142 0.254 0.155 0.495 39.92 0.793 0.674 295 303 -0.133333 0.052805

ENSMUST00000021666 1821 0.2052 0.4225 0.1720 0.4411 0.217 0.096 0.460 47.21 0.688 0.546 583 606 0.015676 0.105611

ENSMUST00000021134 1578 0.2195 0.4122 0.1583 0.4644 0.220 0.101 0.471 50.33 0.696 0.596 514 525 -0.626095 0.078095

ENSMUST00000020577 489 0.1678 0.4406 0.1440 0.4083 0.199 0.204 0.519 48.81 0.727 0.636 154 162 -0.474691 0.117284

ENSMUST00000018653 759 0.1542 0.4826 0.1232 0.4721 0.235 0.122 0.504 44.76 0.772 0.646 246 252 -0.714683 0.071429

ENSMUST00000017920 915 0.2642 0.3577 0.2008 0.4248 0.175 -0.016 0.401 55.87 0.620 0.563 297 304 -0.654605 0.085526

ENSMUST00000021605 5931 0.3672 0.2455 0.3751 0.3389 0.230 0.014 0.433 54.61 0.437 0.437 1927 1976 -0.880213 0.028846

ENSMUST00000020341 330 0.2222 0.3827 0.1596 0.4778 0.261 0.202 0.533 42.02 0.692 0.624 107 109 -0.855046 0.036697

ENSMUST00000018614 1215 0.2893 0.3585 0.2266 0.3365 0.215 0.051 0.447 58.20 0.568 0.555 387 404 -0.315594 0.064356

ENSMUST00000020182 957 0.3545 0.3246 0.2304 0.3211 0.251 0.053 0.449 54.61 0.515 0.515 305 318 -0.151887 0.110063

ENSMUST00000020444 393 0.2152 0.3038 0.3173 0.4554 0.180 -0.079 0.383 44.18 0.583 0.508 120 130 -1.370.000 0.030769

ENSMUST00000020064 474 0.3033 0.3525 0.2656 0.2906 0.215 0.133 0.486 61.00 0.520 0.527 148 157 -0.232484 0.082803

ENSMUST00000015509 3564 0.2972 0.3351 0.2797 0.3510 0.217 0.034 0.432 51.95 0.532 0.487 1157 1187 -0.293345 0.070767

ENSMUST00000021607 1308 0.2593 0.4644 0.1717 0.3927 0.288 0.132 0.499 48.72 0.656 0.526 413 435 -0.367586 0.094253

ENSMUST00000021009 3558 0.2992 0.3937 0.2800 0.3429 0.232 0.002 0.438 53.88 0.555 0.511 1145 1185 -1.065.823 0.041350

ENSMUST00000019063 591 0.2151 0.4012 0.2207 0.3603 0.227 0.087 0.455 53.23 0.631 0.573 187 196 0.611224 0.071429

ENSMUST00000018767 804 0.3287 0.3287 0.3172 0.2931 0.253 0.074 0.464 50.81 0.484 0.483 252 267 -0.300375 0.116105

ENSMUST00000020450 1971 0.2585 0.4039 0.2281 0.3181 0.241 0.101 0.465 52.77 0.587 0.505 620 656 0.679726 0.111280

ENSMUST00000019266 369 0.3438 0.3542 0.3043 0.2805 0.198 -0.062 0.398 59.75 0.483 0.470 118 122 -0.328688 0.098361

ENSMUST00000018699 855 0.2411 0.4196 0.1990 0.4162 0.212 0.080 0.463 49.86 0.652 0.545 270 284 -0.659507 0.084507

ENSMUST00000014499 5835 0.3681 0.2809 0.2831 0.3035 0.209 0.016 0.417 54.83 0.464 0.477 1863 1944 -0.101492 0.078189

ENSMUST00000021226 1476 0.3671 0.2110 0.4442 0.2571 0.159 -0.038 0.405 51.19 0.361 0.435 479 491 -1.475.356 0.038697

ENSMUST00000015000 522 0.2015 0.4552 0.2362 0.3333 0.206 0.061 0.449 53.40 0.639 0.539 158 173 0.094798 0.132948

ENSMUST00000019660 1686 0.2902 0.3549 0.3011 0.3565 0.215 0.009 0.435 53.52 0.541 0.515 549 561 -0.917825 0.057041

ENSMUST00000017783 1908 0.1617 0.4745 0.1575 0.5116 0.279 0.152 0.510 40.71 0.752 0.584 618 635 -0.775276 0.045669

ENSMUST00000021290 609 0.1971 0.5109 0.1753 0.5035 0.315 0.101 0.492 47.27 0.723 0.521 195 202 -0.657426 0.108911

ENSMUST00000020957 540 0.2519 0.4427 0.1917 0.4679 0.256 0.028 0.436 48.94 0.661 0.525 165 179 -0.717877 0.128492

ENSMUST00000015498 1245 0.2899 0.3942 0.2336 0.3262 0.220 0.056 0.454 55.21 0.571 0.514 399 414 -0.211594 0.089372

ENSMUST00000021405 453 0.3171 0.3740 0.2039 0.4105 0.276 0.121 0.490 55.86 0.586 0.489 145 150 -0.336000 0.113333

ENSMUST00000017908 1368 0.1833 0.5722 0.1228 0.3704 0.276 0.224 0.541 43.11 0.751 0.582 434 455 -0.139560 0.090110

ENSMUST00000017561 1503 0.2080 0.5154 0.1538 0.3547 0.277 0.205 0.541 47.01 0.702 0.579 484 500 -0.374800 0.096000

ENSMUST00000021661 1431 0.2707 0.4185 0.2011 0.2957 0.232 0.062 0.453 54.49 0.598 0.555 450 476 0.019118 0.073529

ENSMUST00000015011 810 0.2064 0.4587 0.1606 0.4101 0.299 0.215 0.530 49.15 0.695 0.529 249 269 0.573978 0.141264

ENSMUST00000017430 897 0.3728 0.2632 0.3318 0.3317 0.240 -0.013 0.420 56.81 0.451 0.472 286 298 -0.419463 0.097315

ENSMUST00000021794 2502 0.2641 0.4198 0.2520 0.3344 0.233 0.038 0.441 52.36 0.589 0.528 807 833 -0.606843 0.070828

ENSMUST00000019443 3201 0.2688 0.4085 0.2005 0.3742 0.222 0.057 0.445 51.43 0.622 0.582 1035 1066 -0.450751 0.073171

ENSMUST00000015920 603 0.2857 0.4610 0.1355 0.4069 0.325 0.211 0.544 50.87 0.667 0.563 195 200 -0.574000 0.050000

ENSMUST00000020094 969 0.3903 0.2900 0.3363 0.2871 0.234 0.020 0.420 54.65 0.429 0.432 317 322 -0.462733 0.071429

ENSMUST00000020759 1482 0.3432 0.3309 0.3871 0.2364 0.226 -0.006 0.430 54.24 0.434 0.467 479 493 -0.354767 0.081136

ENSMUST00000020801 2976 0.3128 0.3492 0.2931 0.2952 0.195 0.011 0.420 56.93 0.511 0.516 962 991 -0.475681 0.078708

ENSMUST00000021231 4569 0.2038 0.4530 0.1691 0.4058 0.245 0.128 0.478 48.36 0.689 0.549 1461 1522 0.228186 0.092641

ENSMUST00000021548 951 0.2471 0.3688 0.2551 0.3593 0.216 0.074 0.455 51.52 0.584 0.518 308 316 0.042721 0.091772

ENSMUST00000020523 1218 0.4243 0.2611 0.3613 0.1588 0.188 -0.045 0.395 51.02 0.346 0.455 390 405 -0.403951 0.106173

ENSMUST00000015433 447 0.2891 0.3672 0.3025 0.2124 0.221 0.067 0.444 55.82 0.493 0.541 144 148 -0.019595 0.040541

ENSMUST00000019012 1299 0.2186 0.4454 0.1615 0.3934 0.206 0.091 0.458 46.69 0.682 0.573 415 432 -0.165046 0.106481

ENSMUST00000021161 1719 0.2462 0.4066 0.2067 0.3698 0.230 0.049 0.430 52.60 0.623 0.509 525 572 0.514860 0.106643

ENSMUST00000015171 1563 0.2370 0.4550 0.2058 0.3444 0.224 0.096 0.474 49.24 0.640 0.560 494 520 -0.306346 0.101923

ENSMUST00000021770 831 0.3881 0.3483 0.2746 0.3389 0.297 0.158 0.515 51.25 0.500 0.453 262 276 -0.479348 0.094203

ENSMUST00000020243 696 0.1000 0.5333 0.0741 0.5187 0.348 0.378 0.644 32.94 0.858 0.678 225 231 -0.595238 0.064935

ENSMUST00000016498 1524 0.2225 0.4621 0.1855 0.3581 0.222 0.098 0.460 49.13 0.663 0.577 489 507 -0.181854 0.100592

ENSMUST00000016033 1836 0.2944 0.3674 0.2820 0.3249 0.215 0.037 0.435 57.89 0.539 0.497 588 611 -0.249591 0.098200

ENSMUST00000021797 882 0.2675 0.3991 0.2430 0.3850 0.231 0.056 0.441 57.59 0.598 0.499 281 293 -0.187372 0.102389

ENSMUST00000020463 1692 0.1558 0.4545 0.1366 0.4879 0.267 0.131 0.484 42.36 0.759 0.604 543 563 -0.017051 0.087034

ENSMUST00000018748 2226 0.1922 0.4528 0.1901 0.3677 0.234 0.126 0.497 51.69 0.679 0.597 714 741 -0.325371 0.070175

ENSMUST00000018896 723 0.3177 0.2500 0.2887 0.3387 0.174 -0.067 0.364 56.67 0.487 0.531 228 240 -0.301667 0.070833

ENSMUST00000020311 1452 0.2694 0.4028 0.2584 0.3746 0.284 0.131 0.505 52.09 0.590 0.507 461 483 -0.523395 0.103520

ENSMUST00000020016 618 0.3118 0.3588 0.2908 0.3496 0.231 0.063 0.439 51.04 0.525 0.437 198 205 0.636098 0.165854

ENSMUST00000021453 1728 0.3122 0.3049 0.3181 0.3632 0.185 -0.039 0.395 55.22 0.508 0.473 557 575 -0.809391 0.036522

ENSMUST00000019439 1089 0.2226 0.4452 0.2148 0.3308 0.238 0.052 0.438 49.08 0.638 0.575 345 362 0.046133 0.104972

ENSMUST00000019994 876 0.2805 0.3943 0.2585 0.3226 0.193 0.003 0.412 56.64 0.563 0.478 279 291 0.125430 0.147766

ENSMUST00000019722 1329 0.2060 0.4090 0.1813 0.4713 0.219 0.039 0.438 46.08 0.690 0.583 436 442 -0.568100 0.065611

ENSMUST00000020677 936 0.2520 0.4488 0.1867 0.3835 0.310 0.177 0.525 51.17 0.645 0.539 299 311 -0.045016 0.070740

ENSMUST00000018993 3315 0.1945 0.4346 0.1907 0.4330 0.244 0.095 0.474 46.52 0.688 0.573 1075 1104 -0.680797 0.057971

ENSMUST00000016530 885 0.2582 0.4139 0.3171 0.2865 0.242 0.096 0.472 60.06 0.546 0.493 282 294 -0.388775 0.119048

ENSMUST00000017881 2193 0.2330 0.4871 0.1718 0.3360 0.291 0.186 0.531 47.87 0.669 0.584 706 730 -0.400548 0.128767

ENSMUST00000018315 1221 0.3754 0.2362 0.3259 0.3404 0.238 0.032 0.434 56.20 0.437 0.462 387 406 -0.000493 0.108374

ENSMUST00000020665 702 0.4671 0.1976 0.4128 0.2516 0.170 -0.082 0.357 54.75 0.326 0.412 221 233 -0.729614 0.042918

ENSMUST00000019908 1122 0.2982 0.3018 0.2626 0.3943 0.220 0.091 0.471 54.61 0.546 0.521 359 373 -0.576408 0.058981

ENSMUST00000021885 375 0.4048 0.2738 0.3830 0.2727 0.276 0.091 0.479 53.43 0.402 0.441 117 124 -0.431452 0.064516

ENSMUST00000017961 2091 0.1429 0.5027 0.1224 0.4330 0.243 0.109 0.485 41.24 0.777 0.671 678 696 -0.792241 0.064655

ENSMUST00000021004 1620 0.2602 0.3688 0.2748 0.3411 0.222 0.031 0.436 53.69 0.566 0.521 521 539 -0.192022 0.087199

ENSMUST00000020461 1320 0.1201 0.4972 0.1221 0.4970 0.290 0.218 0.541 37.95 0.801 0.629 427 439 -0.540775 0.070615

ENSMUST00000017288 735 0.2798 0.3627 0.3111 0.3314 0.223 0.006 0.428 54.39 0.534 0.470 236 244 -0.450820 0.065574

ENSMUST00000021802 1431 0.2636 0.3940 0.2661 0.3410 0.226 0.034 0.436 53.35 0.573 0.520 459 476 -0.458403 0.067227

ENSMUST00000018795 1332 0.3120 0.3440 0.3166 0.3356 0.204 -0.034 0.401 57.25 0.512 0.481 426 443 -0.585327 0.092551

ENSMUST00000018561 3411 0.3189 0.3528 0.2727 0.3813 0.234 0.050 0.449 55.57 0.543 0.464 1103 1136 -0.489349 0.101232

ENSMUST00000018965 429 0.2124 0.4248 0.1513 0.4211 0.221 0.198 0.536 47.27 0.696 0.629 138 142 -0.999296 0.042254

ENSMUST00000021706 1704 0.2429 0.4381 0.1758 0.4700 0.291 0.127 0.496 48.69 0.679 0.534 548 567 -0.500529 0.063492

ENSMUST00000015800 1941 0.3785 0.3865 0.2112 0.3079 0.279 0.058 0.463 53.07 0.531 0.491 631 646 -0.451858 0.063467

ENSMUST00000017458 1659 0.1955 0.4273 0.1618 0.4492 0.240 0.097 0.465 45.68 0.705 0.593 536 552 -0.367210 0.065217

ENSMUST00000021246 816 0.2560 0.3961 0.1349 0.4476 0.203 0.024 0.426 52.11 0.682 0.581 258 271 -0.518450 0.077491

ENSMUST00000020508 411 0.2000 0.3400 0.2736 0.4706 0.194 0.025 0.420 56.85 0.626 0.529 131 136 -0.565441 0.073529

ENSMUST00000015858 1143 0.2730 0.4671 0.2222 0.3051 0.228 0.092 0.465 50.90 0.603 0.502 355 380 0.007368 0.171053

ENSMUST00000021208 426 0.1468 0.4312 0.1273 0.5686 0.234 0.246 0.548 37.71 0.778 0.582 135 141 -0.182979 0.056738

ENSMUST00000021961 1188 0.2539 0.4357 0.1469 0.4528 0.236 0.054 0.440 49.05 0.674 0.484 377 395 0.305569 0.121519

ENSMUST00000019037 465 0.2569 0.4495 0.1500 0.4865 0.266 -0.061 0.396 42.28 0.691 0.539 149 154 -0.369480 0.071429

ENSMUST00000018521 1557 0.2652 0.3771 0.2871 0.3008 0.216 0.007 0.429 55.39 0.545 0.512 499 518 -0.430309 0.042471

ENSMUST00000017188 1200 0.3100 0.3800 0.2561 0.3491 0.246 0.032 0.448 55.27 0.557 0.481 377 399 -0.229073 0.100251

ENSMUST00000021631 2628 0.3609 0.2840 0.3477 0.2814 0.210 0.001 0.411 56.51 0.436 0.441 847 875 -0.266057 0.067429

ENSMUST00000021413 945 0.2058 0.4609 0.1441 0.4802 0.277 0.146 0.498 51.18 0.725 0.590 305 314 -0.428025 0.057325

ENSMUST00000020649 3939 0.3429 0.2754 0.3652 0.3707 0.220 0.045 0.451 56.04 0.467 0.433 1273 1312 -0.915777 0.039634

ENSMUST00000015511 5778 0.1538 0.5144 0.1303 0.4370 0.292 0.221 0.542 42.20 0.765 0.607 1872 1925 -0.173454 0.072208

ENSMUST00000021011 294 0.2535 0.4507 0.2500 0.3188 0.200 -0.022 0.407 51.77 0.593 0.512 91 97 -0.195876 0.082474

ENSMUST00000021494 2469 0.3002 0.2928 0.4162 0.3361 0.206 -0.003 0.421 56.35 0.451 0.406 794 822 -0.690998 0.060827

ENSMUST00000017851 1419 0.3359 0.4217 0.1958 0.2558 0.260 0.117 0.481 53.84 0.551 0.480 443 472 0.522881 0.135593

ENSMUST00000021495 1218 0.2406 0.4781 0.1815 0.3665 0.256 0.125 0.492 46.96 0.660 0.511 388 405 -0.192840 0.096296

ENSMUST00000020149 1122 0.2539 0.3477 0.3389 0.3497 0.227 0.077 0.469 59.11 0.531 0.479 356 373 -0.693298 0.048257

ENSMUST00000021179 606 0.2635 0.3772 0.2517 0.3456 0.226 0.056 0.453 56.38 0.579 0.557 190 201 -0.242786 0.099502

ENSMUST00000021728 528 0.2378 0.3986 0.2256 0.3769 0.228 0.037 0.447 51.59 0.624 0.592 170 175 -0.118286 0.057143

ENSMUST00000021063 1371 0.3077 0.3385 0.2691 0.3981 0.254 0.107 0.477 56.71 0.551 0.464 434 456 -0.396930 0.065789

ENSMUST00000016294 8196 0.3729 0.3082 0.3250 0.2564 0.208 -0.017 0.413 54.08 0.439 0.462 2634 2731 -0.353936 0.091175

ENSMUST00000020979 723 0.3882 0.2294 0.3757 0.3158 0.189 -0.058 0.378 56.37 0.404 0.394 230 240 -0.197917 0.095833

ENSMUST00000015829 1299 0.3102 0.3253 0.3494 0.2852 0.228 0.050 0.457 55.11 0.471 0.471 414 432 -0.119213 0.094907

ENSMUST00000015622 543 0.2617 0.4027 0.2463 0.3308 0.220 0.058 0.457 48.57 0.589 0.587 175 180 -0.304445 0.133333

ENSMUST00000020078 1014 0.3462 0.4115 0.2581 0.3561 0.260 0.001 0.436 55.99 0.552 0.492 326 337 -0.312760 0.100890

ENSMUST00000018623 711 0.2381 0.3988 0.2394 0.4066 0.181 -0.055 0.381 51.98 0.624 0.523 226 236 -0.714830 0.067797

ENSMUST00000014891 1494 0.2329 0.4253 0.2356 0.3646 0.211 0.056 0.442 51.29 0.622 0.535 482 497 -0.625755 0.072435

ENSMUST00000019975 1680 0.3618 0.2544 0.3039 0.2851 0.192 -0.034 0.370 57.18 0.442 0.530 548 559 -0.623614 0.042934

ENSMUST00000021676 423 0.1667 0.4250 0.3048 0.2990 0.194 0.018 0.402 50.79 0.606 0.517 132 140 0.540000 0.114286

ENSMUST00000021048 2517 0.3440 0.2867 0.2753 0.3942 0.259 0.029 0.438 52.08 0.523 0.507 821 838 -0.820883 0.062053

ENSMUST00000014686 1647 0.3000 0.3450 0.1958 0.4348 0.238 0.079 0.473 51.15 0.609 0.529 522 548 -0.620438 0.054745

ENSMUST00000021523 930 0.3302 0.2744 0.3195 0.4113 0.221 0.027 0.430 57.19 0.510 0.467 302 309 -0.714239 0.064725

ENSMUST00000018304 4683 0.3839 0.3081 0.2567 0.2725 0.225 -0.016 0.421 54.59 0.469 0.488 1509 1560 -0.650385 0.044872

ENSMUST00000020045 7023 0.3701 0.2869 0.3349 0.2610 0.216 -0.009 0.412 53.25 0.429 0.452 2240 2340 -0.163162 0.107265

ENSMUST00000020022 1338 0.3388 0.3306 0.2935 0.3038 0.247 0.041 0.438 57.24 0.494 0.467 427 445 -0.142023 0.119101

ENSMUST00000021512 1017 0.2632 0.3271 0.2218 0.4435 0.212 0.062 0.438 56.21 0.603 0.505 320 338 -0.011835 0.082840

ENSMUST00000020741 1104 0.4150 0.2721 0.2621 0.3230 0.242 0.002 0.413 51.94 0.452 0.451 361 367 -0.154223 0.065395

ENSMUST00000017348 1341 0.1965 0.3900 0.2591 0.4000 0.222 0.059 0.436 51.66 0.630 0.545 433 446 -0.231166 0.060538

ENSMUST00000018572 2574 0.2719 0.3538 0.2271 0.3692 0.233 0.037 0.435 53.57 0.586 0.553 834 857 -0.348425 0.052509

ENSMUST00000020528 1002 0.2751 0.3457 0.2374 0.3333 0.199 0.031 0.424 55.22 0.567 0.586 323 333 -0.417718 0.060060

ENSMUST00000021963 885 0.2350 0.3632 0.2532 0.3901 0.214 0.118 0.486 57.74 0.601 0.543 286 294 -0.356123 0.081633

ENSMUST00000021941 621 0.1373 0.3922 0.1714 0.5318 0.204 0.133 0.488 44.06 0.749 0.623 203 206 -0.893689 0.033981

ENSMUST00000021903 480 0.2109 0.4531 0.1333 0.4865 0.302 0.215 0.542 47.23 0.723 0.591 155 159 -0.113837 0.050314

ENSMUST00000019931 1452 0.1971 0.4136 0.1851 0.4016 0.201 0.108 0.473 49.57 0.677 0.619 474 483 -0.379710 0.047619

ENSMUST00000021335 1920 0.3733 0.2615 0.3811 0.2624 0.217 -0.007 0.410 52.17 0.402 0.428 615 639 -0.303287 0.081377

ENSMUST00000015846 1038 0.3094 0.3201 0.2403 0.3670 0.239 0.057 0.446 54.69 0.548 0.528 341 345 -0.158261 0.057971

ENSMUST00000020161 972 0.2594 0.3233 0.2996 0.3542 0.235 0.077 0.457 53.29 0.539 0.514 317 323 -0.186997 0.068111

ENSMUST00000018571 360 0.2473 0.4624 0.3704 0.2400 0.263 0.123 0.509 57.72 0.535 0.473 114 119 -0.400840 0.109244

ENSMUST00000021033 828 0.2489 0.3575 0.2121 0.3801 0.209 0.065 0.446 54.31 0.610 0.564 267 275 -0.097818 0.043636

ENSMUST00000017339 981 0.3516 0.3004 0.3836 0.2624 0.218 -0.020 0.406 58.55 0.429 0.439 315 326 -0.089264 0.110429

ENSMUST00000020339 2016 0.3525 0.2693 0.3862 0.2913 0.205 -0.072 0.378 52.90 0.423 0.441 638 671 -0.389568 0.092399

ENSMUST00000021177 2160 0.2334 0.4443 0.2243 0.3686 0.236 0.095 0.470 50.37 0.632 0.531 690 719 -0.325869 0.094576

ENSMUST00000020420 3600 0.2151 0.4786 0.1419 0.4709 0.307 0.143 0.500 44.45 0.721 0.553 1163 1199 -0.462886 0.054212

ENSMUST00000020288 363 0.2308 0.4519 0.1778 0.3614 0.239 0.170 0.521 56.03 0.658 0.575 117 120 -0.548333 0.041667

ENSMUST00000020550 708 0.1189 0.5189 0.0983 0.5671 0.274 0.119 0.487 36.47 0.829 0.610 228 235 -0.600000 0.110638

ENSMUST00000017148 369 0.2917 0.4063 0.2900 0.1979 0.190 0.075 0.487 52.89 0.504 0.503 115 122 -0.472951 0.073770

ENSMUST00000019512 1212 0.2567 0.4067 0.1528 0.4842 0.231 0.027 0.426 49.03 0.679 0.544 383 403 -0.350372 0.096774

ENSMUST00000020938 1626 0.1596 0.5188 0.1975 0.3714 0.210 0.146 0.491 48.53 0.712 0.579 525 541 -0.259704 0.092421

ENSMUST00000021076 672 0.2582 0.4505 0.1706 0.3648 0.258 0.097 0.481 48.34 0.648 0.550 216 223 -0.261884 0.098655

ENSMUST00000021614 2355 0.2846 0.3927 0.2353 0.3445 0.237 0.011 0.430 52.57 0.575 0.517 751 784 -0.111480 0.070153

ENSMUST00000015227 507 0.2500 0.3382 0.2538 0.3889 0.207 0.042 0.432 61.00 0.586 0.556 162 168 -0.100000 0.101190

ENSMUST00000015449 3693 0.2450 0.4013 0.2308 0.3690 0.228 0.081 0.466 50.62 0.614 0.552 1192 1230 -0.808862 0.047154

ENSMUST00000020106 3567 0.2432 0.4567 0.2581 0.3247 0.240 0.097 0.481 52.17 0.604 0.525 1165 1188 -0.762205 0.063131

ENSMUST00000019456 1608 0.2483 0.4328 0.1659 0.3914 0.252 0.150 0.498 46.48 0.661 0.581 522 535 -0.418879 0.085981

ENSMUST00000015138 2583 0.4273 0.1860 0.2962 0.1493 0.191 0.042 0.448 46.65 0.314 0.626 859 860 0.501977 0.043023

ENSMUST00000021837 1164 0.3267 0.3500 0.3187 0.3189 0.233 0.006 0.423 47.73 0.501 0.444 371 387 -0.188114 0.111111

ENSMUST00000021407 1515 0.4294 0.2050 0.4213 0.2391 0.227 -0.062 0.397 48.27 0.333 0.407 469 504 -0.244246 0.065476

ENSMUST00000020101 2088 0.2062 0.4836 0.1667 0.4194 0.251 0.140 0.495 47.41 0.703 0.564 666 695 -0.315683 0.084892

ENSMUST00000020057 702 0.2798 0.2679 0.3198 0.3810 0.246 0.060 0.449 58.51 0.515 0.522 227 233 -0.487554 0.038627

ENSMUST00000017544 1227 0.1779 0.4785 0.2232 0.3459 0.250 0.126 0.486 47.45 0.670 0.585 397 408 -0.642647 0.061275

ENSMUST00000021824 3498 0.4386 0.2527 0.3998 0.2467 0.237 -0.053 0.411 51.78 0.368 0.414 1134 1165 -1.004.635 0.054077

ENSMUST00000019117 894 0.2114 0.4390 0.1941 0.3590 0.200 0.032 0.448 47.80 0.662 0.607 290 297 -0.718519 0.097643

ENSMUST00000020927 1188 0.2086 0.4139 0.1927 0.4322 0.223 0.033 0.425 46.43 0.675 0.585 388 395 -0.613924 0.050633

ENSMUST00000020241 1362 0.3251 0.3664 0.2301 0.3778 0.237 0.044 0.444 52.74 0.563 0.494 448 453 -0.381678 0.114790

ENSMUST00000020977 975 0.3702 0.2137 0.4372 0.2440 0.179 -0.034 0.397 47.50 0.351 0.426 305 324 -0.148457 0.077160

ENSMUST00000020399 1332 0.2534 0.3994 0.2410 0.3986 0.256 0.135 0.492 51.88 0.609 0.505 425 443 -0.305869 0.117381

ENSMUST00000020679 1221 0.2155 0.5201 0.1677 0.2950 0.247 0.132 0.481 47.75 0.673 0.529 391 406 0.642364 0.098522

ENSMUST00000018568 1095 0.2085 0.4735 0.1958 0.3977 0.299 0.139 0.497 46.26 0.673 0.532 352 364 -0.139011 0.074176

ENSMUST00000021372 603 0.3439 0.2994 0.4203 0.2460 0.177 -0.076 0.363 56.02 0.411 0.420 190 200 -0.225500 0.095000

ENSMUST00000020776 834 0.2977 0.3349 0.2511 0.3288 0.217 0.073 0.455 51.05 0.545 0.572 266 277 -0.664621 0.039711

ENSMUST00000019662 1350 0.3018 0.3727 0.2062 0.3294 0.196 0.035 0.420 53.40 0.574 0.537 441 449 -0.104900 0.080178

ENSMUST00000020040 510 0.2868 0.3876 0.3033 0.3243 0.230 0.147 0.494 52.37 0.537 0.458 160 169 -0.371006 0.076923

ENSMUST00000021707 1377 0.1461 0.4660 0.1008 0.4770 0.266 0.253 0.555 41.31 0.789 0.659 445 458 0.068122 0.063319

ENSMUST00000021854 1077 0.3022 0.3489 0.2894 0.3142 0.234 0.010 0.433 55.70 0.523 0.491 342 358 -0.297207 0.089385

ENSMUST00000019470 765 0.3177 0.3333 0.2755 0.3908 0.204 -0.021 0.393 52.09 0.534 0.450 247 254 -0.371260 0.066929

ENSMUST00000017276 1194 0.1966 0.4881 0.2125 0.4598 0.261 0.139 0.504 47.65 0.689 0.524 383 397 -0.673048 0.090680

ENSMUST00000021323 1149 0.1881 0.4356 0.1867 0.3981 0.209 0.155 0.499 46.86 0.687 0.607 371 382 -0.664922 0.049738

ENSMUST00000019962 594 0.3046 0.3793 0.2338 0.2568 0.234 0.019 0.430 61.00 0.539 0.518 193 197 0.029441 0.065990

ENSMUST00000016907 3057 0.1928 0.5401 0.1943 0.3761 0.287 0.136 0.516 44.87 0.702 0.577 997 1018 -0.536444 0.069745

ENSMUST00000015712 1425 0.2663 0.4230 0.2435 0.3427 0.231 0.007 0.428 50.48 0.594 0.508 458 474 -0.307806 0.103376

ENSMUST00000021559 315 0.3718 0.3590 0.1944 0.4242 0.307 0.140 0.505 56.89 0.566 0.474 99 104 -0.666346 0.105769

ENSMUST00000021779 684 0.2857 0.3600 0.3091 0.3248 0.250 0.152 0.502 52.54 0.530 0.463 215 227 -0.323789 0.096916

ENSMUST00000016406 1134 0.2926 0.4373 0.2292 0.2694 0.225 0.078 0.473 54.21 0.571 0.527 366 377 -0.494695 0.079576

ENSMUST00000017741 1464 0.2970 0.3706 0.2100 0.3653 0.252 0.147 0.513 49.84 0.589 0.551 470 487 -0.514784 0.049281

ENSMUST00000015236 447 0.1748 0.4466 0.1563 0.5169 0.254 0.054 0.462 47.13 0.738 0.581 145 148 -0.930405 0.020270

ENSMUST00000021813 765 0.1257 0.4450 0.1500 0.4884 0.234 0.120 0.486 47.04 0.769 0.659 247 254 -0.584646 0.051181

ENSMUST00000020488 558 0.3235 0.3382 0.2078 0.3889 0.237 0.071 0.472 58.98 0.573 0.528 178 185 -0.660541 0.027027

ENSMUST00000018816 618 0.2357 0.4076 0.1875 0.4333 0.240 0.084 0.464 53.26 0.658 0.538 196 205 -0.248293 0.082927

ENSMUST00000019991 1428 0.3069 0.3529 0.2393 0.3600 0.229 0.068 0.451 53.89 0.556 0.491 459 475 -0.064210 0.073684

ENSMUST00000020856 1260 0.2633 0.3967 0.3000 0.3583 0.285 0.127 0.495 54.28 0.567 0.485 404 419 -0.384725 0.095465

ENSMUST00000019220 2283 0.2576 0.4187 0.1843 0.3659 0.255 0.070 0.461 49.94 0.635 0.589 740 760 -0.469211 0.052632

ENSMUST00000018798 1287 0.2202 0.4940 0.1749 0.4061 0.273 0.116 0.498 48.33 0.692 0.564 412 428 -0.810514 0.058411

ENSMUST00000015796 1020 0.2985 0.3358 0.2778 0.3333 0.216 0.054 0.426 52.85 0.527 0.468 317 339 0.183776 0.123894

ENSMUST00000015124 507 0.3308 0.3008 0.3154 0.2773 0.185 -0.013 0.399 61.00 0.462 0.494 158 168 -0.197619 0.065476

ENSMUST00000019939 489 0.3023 0.3566 0.2602 0.3717 0.187 0.049 0.440 59.63 0.553 0.496 159 162 -0.629630 0.092593

ENSMUST00000021347 2373 0.3116 0.3589 0.2684 0.3459 0.249 0.068 0.466 56.09 0.543 0.512 761 790 -0.406329 0.102532

ENSMUST00000019441 1911 0.3166 0.2915 0.1977 0.3988 0.195 0.015 0.408 54.78 0.571 0.567 627 636 -0.109434 0.056604

ENSMUST00000020617 4092 0.1916 0.4830 0.1792 0.4008 0.245 0.121 0.487 45.98 0.700 0.577 1307 1363 -0.389949 0.083639

ENSMUST00000020286 870 0.3467 0.3778 0.2879 0.3184 0.285 -0.002 0.430 54.90 0.513 0.475 277 289 -0.506574 0.107266

ENSMUST00000015576 735 0.3927 0.2618 0.3459 0.2663 0.198 -0.094 0.372 61.00 0.406 0.447 234 244 -0.148361 0.090164

ENSMUST00000016231 1359 0.2402 0.4693 0.2120 0.3465 0.234 0.055 0.460 49.31 0.641 0.537 426 452 -0.796903 0.099558

ENSMUST00000021544 1062 0.2473 0.4364 0.2190 0.3608 0.243 0.085 0.468 52.45 0.624 0.525 340 353 -0.428612 0.090652

ENSMUST00000019944 576 0.3453 0.3453 0.2378 0.3723 0.279 0.120 0.492 53.76 0.547 0.511 181 191 -0.368063 0.057592

ENSMUST00000021148 513 0.5000 0.1923 0.3680 0.2281 0.235 -0.018 0.401 60.64 0.315 0.429 162 170 -0.553529 0.088235

ENSMUST00000017732 1347 0.1983 0.4810 0.1841 0.4137 0.277 0.148 0.513 47.05 0.696 0.567 437 448 -0.488616 0.044643

ENSMUST00000021552 1017 0.1906 0.5953 0.0769 0.3095 0.274 0.282 0.586 40.76 0.769 0.628 333 338 -0.502663 0.076923

ENSMUST00000021087 669 0.2222 0.4444 0.1296 0.5033 0.301 0.151 0.500 49.93 0.722 0.566 212 222 -0.177027 0.090090

ENSMUST00000019576 1284 0.3096 0.3839 0.1714 0.4542 0.260 0.035 0.432 51.01 0.626 0.521 412 427 -0.299297 0.079625

ENSMUST00000018246 381 0.0430 0.6559 0.0093 0.5644 0.328 0.166 0.528 27.29 0.959 0.640 123 126 -0.738095 0.055556

ENSMUST00000016703 411 0.1981 0.4245 0.1597 0.4414 0.210 0.142 0.500 45.29 0.701 0.586 134 136 -0.618382 0.051471

ENSMUST00000014221 588 0.2632 0.4013 0.2361 0.4198 0.233 0.088 0.474 57.49 0.611 0.501 190 195 -0.626154 0.076923

ENSMUST00000019268 1245 0.3006 0.3773 0.2825 0.3194 0.237 0.024 0.438 58.14 0.537 0.521 400 414 -0.419565 0.084541

ENSMUST00000018382 1326 0.2722 0.4194 0.1864 0.3466 0.221 0.107 0.471 50.51 0.621 0.541 425 441 -0.357143 0.088435

ENSMUST00000021411 1755 0.3673 0.3252 0.3024 0.3154 0.224 0.009 0.426 53.47 0.480 0.455 563 584 -0.471918 0.104452

ENSMUST00000015771 1215 0.1547 0.5215 0.1821 0.2969 0.253 0.196 0.538 46.70 0.710 0.634 390 404 -0.337376 0.071782

ENSMUST00000019977 1026 0.2817 0.3169 0.2271 0.3711 0.203 0.062 0.443 52.73 0.566 0.555 327 341 -0.056012 0.079179

ENSMUST00000020537 516 0.2374 0.4604 0.1250 0.4331 0.250 0.070 0.456 50.90 0.704 0.561 169 171 -0.185380 0.099415

ENSMUST00000021241 723 0.1917 0.4715 0.2051 0.3438 0.289 0.261 0.560 41.16 0.671 0.600 234 240 -0.590000 0.083333

ENSMUST00000019927 1383 0.4006 0.2238 0.3904 0.2866 0.210 -0.072 0.378 52.47 0.381 0.417 444 460 -0.471304 0.115217

ENSMUST00000020922 1032 0.2650 0.4028 0.1621 0.4530 0.257 0.136 0.497 51.37 0.655 0.535 336 343 -0.337609 0.093294

ENSMUST00000018430 717 0.2500 0.4050 0.2216 0.3216 0.229 0.064 0.463 58.73 0.599 0.570 227 238 -0.006723 0.079832

ENSMUST00000019503 1620 0.2292 0.4090 0.2418 0.3378 0.207 0.084 0.439 49.91 0.609 0.534 506 539 0.263636 0.100186

ENSMUST00000019074 279 0.2821 0.4744 0.1515 0.2769 0.241 0.083 0.460 41.98 0.632 0.536 87 92 0.183696 0.108696

ENSMUST00000020705 1755 0.2776 0.4141 0.1956 0.4444 0.249 0.008 0.425 50.03 0.639 0.528 570 584 -0.630479 0.092466

ENSMUST00000021091 1233 0.4581 0.2194 0.3591 0.2411 0.250 -0.033 0.410 54.33 0.353 0.437 385 410 -0.513659 0.087805

ENSMUST00000019920 984 0.3398 0.3398 0.2913 0.3255 0.231 0.056 0.442 55.07 0.503 0.467 312 327 -0.377676 0.103976

ENSMUST00000019861 3171 0.3983 0.2873 0.3248 0.3238 0.228 -0.064 0.398 57.00 0.449 0.458 1023 1056 -0.564867 0.077652

ENSMUST00000017549 2097 0.2216 0.4278 0.1596 0.3955 0.232 0.117 0.473 45.59 0.677 0.592 678 698 -0.026218 0.053009

ENSMUST00000020706 3357 0.1813 0.4560 0.1513 0.4333 0.235 0.142 0.488 46.81 0.722 0.584 1082 1118 0.153757 0.084079

ENSMUST00000020252 765 0.5130 0.1494 0.4337 0.2912 0.241 0.027 0.454 48.25 0.317 0.382 240 254 -0.943701 0.059055

ENSMUST00000018485 2625 0.3296 0.3338 0.3187 0.2645 0.208 0.006 0.417 55.95 0.471 0.474 840 874 -0.321053 0.085812

ENSMUST00000015587 747 0.3116 0.3367 0.2500 0.3636 0.237 0.058 0.444 59.32 0.544 0.495 241 248 -0.067339 0.056452

ENSMUST00000021204 1308 0.1643 0.4409 0.1554 0.4969 0.231 0.112 0.474 45.55 0.739 0.604 422 435 -0.210805 0.087356

ENSMUST00000019749 870 0.1957 0.4511 0.1972 0.4104 0.242 0.146 0.514 50.94 0.684 0.614 282 289 -0.833564 0.096886

ENSMUST00000018337 1596 0.3951 0.2222 0.4000 0.2354 0.193 -0.073 0.368 51.34 0.357 0.443 513 531 -0.576836 0.071563

ENSMUST00000019426 3126 0.3002 0.3345 0.3474 0.2625 0.193 -0.051 0.392 53.98 0.472 0.477 998 1041 -0.187512 0.072046

ENSMUST00000017751 2091 0.2230 0.4774 0.1986 0.2962 0.224 0.126 0.490 47.21 0.643 0.580 678 696 -0.382328 0.053161

ENSMUST00000021620 705 0.2139 0.4920 0.2378 0.3893 0.274 0.168 0.520 52.59 0.655 0.493 229 234 -0.285470 0.115385

ENSMUST00000021097 2949 0.2826 0.4102 0.2026 0.3616 0.233 0.039 0.448 52.12 0.611 0.556 963 982 -0.576884 0.070265

ENSMUST00000021898 1425 0.2120 0.4503 0.2225 0.3399 0.211 0.080 0.462 48.81 0.641 0.572 457 474 -0.529958 0.063291

ENSMUST00000016678 1248 0.4253 0.2816 0.3056 0.2428 0.257 0.032 0.444 56.57 0.407 0.427 405 415 -0.038313 0.103614

ENSMUST00000020827 1188 0.4177 0.2317 0.4000 0.2087 0.190 -0.052 0.381 52.15 0.341 0.410 378 395 0.124810 0.139241

ENSMUST00000014691 1203 0.2355 0.4129 0.2696 0.3553 0.262 0.083 0.472 53.28 0.597 0.530 377 400 -0.351750 0.082500

ENSMUST00000020794 363 0.4000 0.1750 0.4255 0.3187 0.266 -0.019 0.417 48.66 0.374 0.411 115 120 -0.560000 0.050000

ENSMUST00000021458 6990 0.1935 0.4423 0.1736 0.5100 0.261 0.081 0.468 46.29 0.717 0.569 2235 2329 -0.679863 0.069128

ENSMUST00000018437 423 0.2348 0.4783 0.1698 0.3168 0.261 0.157 0.515 41.94 0.659 0.564 132 140 0.031429 0.078571

ENSMUST00000020379 1950 0.2090 0.4613 0.1402 0.3567 0.216 0.083 0.469 44.33 0.699 0.632 635 649 -0.546071 0.050847

ENSMUST00000020804 945 0.2698 0.3849 0.2783 0.3602 0.232 0.062 0.443 59.83 0.567 0.470 305 314 -0.037898 0.098726

ENSMUST00000019374 993 0.2337 0.4866 0.1867 0.3739 0.267 0.173 0.527 50.16 0.668 0.554 319 330 -0.614546 0.075758

ENSMUST00000015333 2394 0.3638 0.3080 0.3030 0.3056 0.242 0.031 0.428 57.12 0.464 0.430 761 797 0.256462 0.136763

ENSMUST00000020197 1251 0.3294 0.3412 0.2773 0.2973 0.233 0.032 0.444 53.53 0.504 0.486 405 416 -0.299519 0.074519

ENSMUST00000017743 1296 0.2492 0.4131 0.2486 0.3927 0.271 0.141 0.504 55.54 0.611 0.513 419 431 -0.661717 0.051044

ENSMUST00000021726 1374 0.1836 0.4932 0.1525 0.4286 0.278 0.171 0.518 44.55 0.726 0.583 442 457 -0.265208 0.080963

ENSMUST00000018313 966 0.1691 0.5000 0.1555 0.4009 0.250 0.218 0.529 47.71 0.732 0.594 310 321 -0.048598 0.093458

ENSMUST00000020543 2190 0.4248 0.2727 0.3073 0.2455 0.221 0.009 0.433 54.42 0.406 0.489 711 729 -0.638409 0.094650

ENSMUST00000017090 705 0.1803 0.4481 0.2303 0.4118 0.268 0.194 0.531 56.23 0.673 0.557 226 234 -0.458547 0.076923

ENSMUST00000016279 717 0.2128 0.4734 0.2749 0.3252 0.206 0.170 0.520 49.34 0.620 0.536 229 238 -1.136.975 0.109244

ENSMUST00000021564 1359 0.2327 0.4515 0.2081 0.3675 0.240 0.071 0.463 51.03 0.646 0.567 441 452 -0.661283 0.057522

ENSMUST00000015581 744 0.2953 0.3316 0.2670 0.3750 0.194 -0.003 0.416 58.94 0.546 0.493 238 247 -0.364777 0.068826

ENSMUST00000021490 1236 0.2266 0.4350 0.2881 0.3296 0.228 0.084 0.462 54.55 0.592 0.463 392 411 -0.051582 0.082725

ENSMUST00000021482 459 0.2212 0.4248 0.2627 0.3565 0.158 -0.048 0.366 47.22 0.614 0.550 145 152 -0.346711 0.078947

ENSMUST00000018841 1581 0.3333 0.2946 0.3051 0.3636 0.210 -0.010 0.415 55.80 0.503 0.497 513 526 -0.909696 0.055133

ENSMUST00000019026 921 0.3333 0.2857 0.2827 0.3600 0.186 -0.044 0.381 54.53 0.505 0.498 291 306 -0.533007 0.094771

ENSMUST00000020392 456 0.2764 0.4065 0.3211 0.2551 0.206 0.065 0.458 61.00 0.521 0.468 144 151 -0.115232 0.105960

ENSMUST00000018990 1113 0.4158 0.2244 0.3700 0.2500 0.212 -0.042 0.396 54.67 0.368 0.425 359 370 -0.125946 0.108108

ENSMUST00000017975 648 0.3728 0.2012 0.5185 0.1765 0.186 -0.120 0.365 50.26 0.293 0.423 208 215 -0.404651 0.079070

ENSMUST00000021957 2337 0.2532 0.4194 0.1866 0.3497 0.220 0.055 0.454 48.43 0.635 0.592 758 778 -0.823136 0.038560

ENSMUST00000020413 1227 0.3225 0.2988 0.3028 0.3056 0.197 -0.005 0.410 55.94 0.485 0.508 398 408 -0.115196 0.080882

ENSMUST00000020270 2205 0.4173 0.1972 0.4147 0.2214 0.189 -0.051 0.398 51.27 0.328 0.437 714 734 -0.732289 0.062670

ENSMUST00000017864 717 0.2378 0.3598 0.3125 0.3799 0.169 -0.088 0.367 55.90 0.562 0.462 226 238 -0.846639 0.054622

ENSMUST00000021891 1002 0.4196 0.2863 0.3263 0.2624 0.233 0.036 0.454 51.84 0.416 0.438 315 333 -0.570270 0.114114

ENSMUST00000020062 2787 0.3468 0.3690 0.2797 0.2686 0.243 0.031 0.438 53.35 0.494 0.448 894 928 0.168965 0.106681

ENSMUST00000020974 405 0.1376 0.5413 0.1089 0.4783 0.333 0.388 0.636 44.23 0.798 0.550 129 134 -0.311194 0.037313

ENSMUST00000019405 2922 0.2241 0.3703 0.1465 0.4083 0.233 0.129 0.470 46.12 0.676 0.651 959 973 -0.267215 0.038027

ENSMUST00000014578 2439 0.2632 0.4195 0.2830 0.3159 0.247 0.096 0.485 53.22 0.571 0.530 781 812 -0.695936 0.092365

ENSMUST00000021921 4305 0.2648 0.4263 0.1937 0.3363 0.221 0.091 0.462 52.47 0.618 0.547 1384 1434 -0.109763 0.096234

ENSMUST00000020123 2082 0.3763 0.2455 0.2756 0.3273 0.216 -0.020 0.409 53.14 0.463 0.501 687 693 -0.594084 0.047619

ENSMUST00000021807 1143 0.3948 0.2318 0.3793 0.3430 0.257 -0.020 0.429 52.25 0.429 0.418 373 380 -1.324.737 0.036842

ENSMUST00000021050 1146 0.2264 0.4493 0.2234 0.3918 0.239 0.115 0.484 52.12 0.647 0.542 368 381 -0.549081 0.112861

ENSMUST00000021497 627 0.2303 0.4667 0.1118 0.4371 0.339 0.297 0.578 43.11 0.719 0.543 199 208 0.405289 0.110577

ENSMUST00000021918 2835 0.1556 0.5229 0.1358 0.4346 0.266 0.196 0.529 43.14 0.762 0.608 904 944 -0.335487 0.080508

ENSMUST00000019231 1392 0.3079 0.3740 0.1777 0.3323 0.258 0.100 0.466 49.09 0.584 0.531 440 463 0.211879 0.107991

ENSMUST00000016471 1071 0.3606 0.3717 0.2520 0.3447 0.274 0.127 0.497 52.84 0.529 0.483 342 356 -0.557865 0.126404

ENSMUST00000021424 1683 0.3163 0.3096 0.3043 0.3368 0.214 0.035 0.439 52.61 0.499 0.481 535 560 -0.240536 0.094643

ENSMUST00000015197 1443 0.2063 0.4879 0.1667 0.3158 0.237 0.127 0.499 48.45 0.683 0.626 461 480 -0.506042 0.062500

ENSMUST00000021056 5526 0.1832 0.5689 0.1245 0.4091 0.301 0.188 0.524 43.24 0.753 0.546 1734 1841 0.065942 0.116784

ENSMUST00000017208 693 0.3667 0.3056 0.3210 0.3108 0.217 0.036 0.429 54.93 0.461 0.420 219 230 -0.410870 0.069565

ENSMUST00000020490 942 0.3206 0.4389 0.2196 0.2784 0.258 0.050 0.457 57.01 0.563 0.475 300 313 -0.196486 0.115016

ENSMUST00000021634 972 0.3780 0.2846 0.2894 0.3767 0.237 -0.027 0.394 52.50 0.489 0.459 315 323 -0.371517 0.095975

ENSMUST00000017759 951 0.2087 0.5118 0.1695 0.3945 0.263 0.129 0.495 46.47 0.699 0.544 309 316 -0.524051 0.085443

ENSMUST00000021262 2034 0.2318 0.4307 0.2090 0.3682 0.230 0.095 0.463 51.70 0.638 0.554 646 677 -0.191581 0.097489

ENSMUST00000016771 5883 0.1715 0.4800 0.1164 0.5887 0.298 0.093 0.481 42.12 0.788 0.573 1899 1960 -0.843622 0.047449

ENSMUST00000019067 354 0.1910 0.4607 0.1563 0.4556 0.303 0.176 0.526 44.69 0.719 0.590 114 117 -0.476068 0.042735

ENSMUST00000021090 654 0.2424 0.4606 0.2587 0.3955 0.237 0.070 0.471 52.53 0.626 0.508 206 217 -0.678341 0.133641

ENSMUST00000020668 846 0.3333 0.2944 0.3102 0.3100 0.207 0.001 0.416 51.31 0.474 0.456 274 281 -0.161922 0.071174

ENSMUST00000014321 618 0.2934 0.3473 0.2500 0.3577 0.203 -0.068 0.373 53.23 0.554 0.486 193 205 0.137561 0.121951

ENSMUST00000016452 459 0.4000 0.2667 0.2589 0.3462 0.198 -0.033 0.400 61.00 0.469 0.482 145 152 -0.628289 0.098684

ENSMUST00000015239 1722 0.2336 0.4631 0.1443 0.4026 0.267 0.130 0.487 45.35 0.688 0.575 554 573 -0.269459 0.097731

ENSMUST00000015003 1233 0.2614 0.4316 0.1839 0.3364 0.241 0.110 0.469 45.41 0.628 0.572 403 410 -0.321707 0.034146

ENSMUST00000021785 2775 0.3366 0.3083 0.3305 0.3017 0.207 0.005 0.418 56.64 0.469 0.461 892 924 -0.293398 0.072511

ENSMUST00000017904 1479 0.2308 0.4814 0.1686 0.3908 0.270 0.159 0.507 47.86 0.682 0.546 471 492 -0.367683 0.119919

ENSMUST00000014917 2169 0.2266 0.5123 0.1737 0.3527 0.278 0.143 0.514 45.85 0.683 0.589 706 722 -0.448477 0.081717

ENSMUST00000014290 2013 0.3490 0.3000 0.3467 0.2774 0.219 0.003 0.412 57.03 0.449 0.490 651 670 -0.724925 0.064179

ENSMUST00000015723 957 0.1090 0.5414 0.0895 0.4585 0.238 0.186 0.522 36.82 0.833 0.690 312 318 -0.467610 0.084906

ENSMUST00000020039 1437 0.4191 0.2467 0.4646 0.1911 0.190 -0.069 0.382 53.07 0.325 0.370 458 478 -0.312552 0.138075

ENSMUST00000021306 2919 0.2636 0.4393 0.1840 0.4009 0.273 0.087 0.464 48.58 0.642 0.527 942 972 -0.245473 0.083333

ENSMUST00000020985 1611 0.2589 0.4584 0.2250 0.3587 0.269 0.176 0.523 49.27 0.620 0.504 524 536 -0.526119 0.082090

ENSMUST00000016553 1248 0.3467 0.3167 0.3333 0.2760 0.222 0.021 0.455 52.76 0.462 0.490 407 415 -1.626.988 0.053012

ENSMUST00000020898 528 0.2794 0.3750 0.3285 0.2769 0.236 0.095 0.465 49.98 0.512 0.469 170 175 -0.393714 0.080000

ENSMUST00000021572 1491 0.3728 0.2988 0.3111 0.2465 0.215 0.050 0.450 58.28 0.436 0.478 482 496 -0.438105 0.052419

ENSMUST00000014220 435 0.3125 0.3393 0.3200 0.3368 0.259 0.049 0.453 61.00 0.511 0.470 137 144 -0.257639 0.118056

ENSMUST00000021078 1485 0.2107 0.3777 0.1916 0.4077 0.191 0.022 0.410 47.88 0.656 0.607 480 494 -0.247166 0.066802

ENSMUST00000020248 585 0.3113 0.2781 0.3376 0.3154 0.227 -0.050 0.386 52.16 0.471 0.491 189 194 -0.346392 0.036082

ENSMUST00000020928 1578 0.2838 0.3874 0.2480 0.3016 0.233 0.066 0.454 53.24 0.562 0.547 504 525 -0.216191 0.099048

ENSMUST00000021822 897 0.4120 0.2232 0.4529 0.2195 0.217 -0.001 0.408 54.31 0.330 0.385 294 298 -0.389262 0.077181

ENSMUST00000020309 1131 0.1672 0.5452 0.2028 0.3643 0.281 0.150 0.519 47.30 0.709 0.574 368 376 -0.770479 0.085106

ENSMUST00000021800 1023 0.1741 0.3889 0.2076 0.4291 0.223 0.172 0.503 49.49 0.676 0.575 330 340 -0.203235 0.041176

ENSMUST00000014614 714 0.1340 0.5670 0.1163 0.4545 0.317 0.272 0.576 37.81 0.801 0.623 231 237 -0.232490 0.071730

ENSMUST00000018651 2271 0.2250 0.4417 0.1769 0.4049 0.252 0.159 0.496 49.45 0.671 0.549 724 756 -0.043386 0.105820

ENSMUST00000021119 939 0.5020 0.2253 0.3486 0.2071 0.231 0.009 0.422 48.07 0.326 0.407 301 312 -0.288462 0.089744

ENSMUST00000020767 1491 0.2157 0.3858 0.2214 0.3924 0.206 0.070 0.449 51.41 0.638 0.589 481 496 -0.385484 0.076613

ENSMUST00000021277 1038 0.2214 0.4389 0.1481 0.4762 0.242 0.111 0.477 49.09 0.706 0.556 333 345 -0.522609 0.084058

ENSMUST00000020103 927 0.2533 0.3867 0.2954 0.3796 0.251 0.066 0.459 57.07 0.571 0.476 296 308 -0.310390 0.084416

ENSMUST00000020374 330 0.3133 0.2169 0.4667 0.3333 0.208 -0.139 0.369 38.58 0.408 0.413 103 109 -0.766055 0.119266

ENSMUST00000018727 1398 0.3159 0.3132 0.2335 0.3977 0.222 0.038 0.434 52.73 0.559 0.548 454 465 -0.840215 0.062366

ENSMUST00000021083 465 0.3095 0.3254 0.2419 0.3171 0.217 0.016 0.436 50.51 0.537 0.550 149 154 -0.868182 0.045455

ENSMUST00000020502 1434 0.1716 0.5074 0.1302 0.4031 0.308 0.307 0.582 43.21 0.743 0.543 455 477 0.621174 0.109015

ENSMUST00000020568 2169 0.3316 0.3175 0.3100 0.2929 0.224 0.072 0.447 52.55 0.479 0.475 695 722 -0.182826 0.078947

ENSMUST00000016110 1500 0.2654 0.4005 0.2243 0.3524 0.225 0.035 0.432 53.93 0.600 0.516 477 499 -0.055711 0.082164

ENSMUST00000020551 1578 0.4537 0.2019 0.3568 0.2500 0.202 -0.078 0.372 50.17 0.348 0.428 503 525 -0.061143 0.083810

ENSMUST00000014743 1659 0.2812 0.3946 0.2046 0.3630 0.211 0.022 0.429 51.39 0.604 0.553 538 552 -0.617572 0.050725

ENSMUST00000018094 1425 0.1900 0.5092 0.1421 0.4212 0.253 0.145 0.499 41.36 0.730 0.575 455 474 -0.280169 0.059072

ENSMUST00000018966 945 0.1152 0.5638 0.1200 0.4793 0.296 0.174 0.514 40.31 0.814 0.614 296 314 -0.171975 0.095541

ENSMUST00000019232 945 0.3487 0.3218 0.2383 0.3318 0.235 0.033 0.420 53.94 0.518 0.496 305 314 0.003822 0.098726

ENSMUST00000020163 1983 0.3561 0.2768 0.3405 0.2651 0.213 -0.008 0.422 55.15 0.429 0.456 645 660 -0.324091 0.059091

ENSMUST00000020846 3405 0.2234 0.3899 0.1944 0.3556 0.242 0.142 0.488 46.77 0.638 0.609 1100 1134 -0.092416 0.053792

ENSMUST00000019514 450 0.3173 0.3750 0.1619 0.5155 0.270 -0.064 0.410 44.14 0.640 0.521 139 149 -0.653691 0.067114

ENSMUST00000019982 684 0.3841 0.2384 0.2706 0.4658 0.257 -0.070 0.400 60.63 0.516 0.485 215 227 -0.754626 0.057269

ENSMUST00000020703 1332 0.3520 0.3492 0.3065 0.2157 0.219 0.039 0.436 56.51 0.455 0.446 420 443 0.070429 0.112867

ENSMUST00000021410 1362 0.3411 0.3178 0.3937 0.2925 0.206 -0.012 0.411 54.90 0.447 0.416 436 453 -0.491612 0.128035

ENSMUST00000019942 789 0.2318 0.4182 0.2500 0.3333 0.208 0.104 0.467 51.37 0.602 0.508 246 262 0.381680 0.129771

ENSMUST00000020203 261 0.2581 0.3548 0.3226 0.3559 0.173 -0.002 0.405 38.20 0.544 0.488 79 86 -0.305814 0.081395

ENSMUST00000016172 9105 0.2108 0.4654 0.1359 0.4030 0.261 0.157 0.505 46.21 0.709 0.598 2972 3034 -0.233355 0.069216

ENSMUST00000020991 2727 0.2373 0.4498 0.1872 0.4012 0.241 0.049 0.453 46.85 0.662 0.576 865 908 -0.670044 0.082599

ENSMUST00000017981 576 0.1538 0.5664 0.0861 0.4861 0.286 0.109 0.484 42.93 0.812 0.597 186 191 -0.524607 0.068063

ENSMUST00000018295 1251 0.2212 0.4268 0.1949 0.4674 0.291 0.061 0.459 45.26 0.674 0.544 405 416 -0.355529 0.079327

ENSMUST00000021816 774 0.2318 0.4773 0.1566 0.3141 0.241 0.149 0.506 46.53 0.668 0.598 247 257 -0.180545 0.054475

ENSMUST00000016168 1446 0.2544 0.4414 0.1507 0.3923 0.251 0.136 0.484 49.79 0.664 0.536 467 481 0.049064 0.087318

ENSMUST00000015157 420 0.1875 0.4732 0.1183 0.5233 0.302 0.128 0.492 45.37 0.754 0.530 130 139 -0.124461 0.107914

ENSMUST00000020454 954 0.1563 0.4375 0.1509 0.5273 0.261 0.096 0.477 45.90 0.756 0.612 308 317 -0.843218 0.059937

ENSMUST00000015719 246 0.2985 0.3881 0.1607 0.3673 0.259 0.122 0.452 43.97 0.603 0.502 73 81 0.888889 0.172840

ENSMUST00000016427 915 0.3080 0.3544 0.2336 0.3873 0.262 0.070 0.465 48.01 0.570 0.526 286 304 -0.490132 0.134868

ENSMUST00000020530 1710 0.2754 0.4068 0.1712 0.3744 0.253 0.150 0.491 49.57 0.629 0.550 552 569 -0.151845 0.065026

ENSMUST00000016115 1875 0.3025 0.3277 0.2723 0.3844 0.221 0.018 0.428 55.05 0.544 0.507 596 624 -0.456410 0.070513

ENSMUST00000016401 588 0.1961 0.3399 0.3419 0.3288 0.231 0.021 0.426 49.09 0.546 0.513 183 195 -0.205128 0.051282

ENSMUST00000014339 1485 0.3599 0.3324 0.3099 0.3678 0.240 -0.059 0.411 55.25 0.505 0.476 479 494 -0.702024 0.107287

ENSMUST00000018875 2856 0.3245 0.3483 0.2493 0.3526 0.242 0.035 0.427 55.55 0.539 0.482 921 951 -0.091062 0.066246

ENSMUST00000015594 744 0.3265 0.3622 0.2819 0.2759 0.206 -0.001 0.411 52.36 0.504 0.484 236 247 -0.073279 0.060729

ENSMUST00000021377 1059 0.2500 0.3714 0.2763 0.4135 0.204 0.043 0.431 55.98 0.589 0.497 343 352 -0.432955 0.099432

ENSMUST00000018700 2175 0.2200 0.4975 0.1720 0.3805 0.240 0.089 0.474 48.94 0.682 0.564 711 724 -0.463122 0.074586

ENSMUST00000020365 2049 0.2296 0.3738 0.2139 0.4275 0.209 0.017 0.427 49.54 0.639 0.565 668 682 -0.657918 0.057185

ENSMUST00000018880 1038 0.3205 0.2201 0.3958 0.3188 0.192 -0.072 0.376 55.55 0.426 0.478 340 345 -0.708406 0.055072

ENSMUST00000020864 645 0.2062 0.4437 0.2092 0.4690 0.302 0.166 0.520 48.10 0.681 0.540 204 214 -0.446729 0.135514

ENSMUST00000019906 516 0.3050 0.3546 0.3077 0.2787 0.220 0.059 0.449 52.34 0.503 0.481 167 171 -0.418129 0.093567

ENSMUST00000014058 837 0.2291 0.4581 0.1748 0.3618 0.272 0.166 0.504 49.95 0.667 0.578 264 278 -0.150360 0.079137

ENSMUST00000020448 1830 0.2609 0.3665 0.3050 0.3341 0.225 0.117 0.478 56.42 0.548 0.505 588 609 -0.341872 0.077176

ENSMUST00000015663 1014 0.4312 0.2464 0.3799 0.1843 0.190 -0.069 0.376 48.39 0.344 0.433 314 337 -0.171810 0.139466

ENSMUST00000020717 543 0.2246 0.4420 0.0889 0.5238 0.282 0.160 0.506 40.23 0.747 0.569 170 180 -0.204445 0.088889

ENSMUST00000021617 1905 0.1541 0.5145 0.1370 0.4384 0.282 0.201 0.528 42.05 0.762 0.609 617 634 -0.285174 0.066246

ENSMUST00000020967 390 0.2885 0.2596 0.3229 0.3895 0.209 -0.098 0.352 50.52 0.512 0.463 125 129 -0.159690 0.085271

ENSMUST00000018333 987 0.4009 0.2026 0.4229 0.2931 0.181 -0.191 0.314 50.71 0.365 0.412 315 328 -0.404268 0.082317

ENSMUST00000019060 426 0.2110 0.4954 0.2830 0.2900 0.270 0.147 0.500 52.44 0.610 0.499 136 141 -0.176596 0.106383

ENSMUST00000019109 741 0.2938 0.3898 0.2198 0.4393 0.273 0.061 0.464 47.30 0.612 0.505 237 246 -0.606504 0.085366

ENSMUST00000021468 6108 0.2541 0.3341 0.2728 0.4521 0.235 0.056 0.454 54.22 0.597 0.520 1976 2035 -0.874889 0.033907

ENSMUST00000015585 747 0.2980 0.2980 0.3194 0.3427 0.212 -0.014 0.408 52.27 0.500 0.495 240 248 -0.272984 0.072581

ENSMUST00000020285 597 0.3439 0.3376 0.3172 0.2632 0.239 0.110 0.479 58.68 0.468 0.458 188 198 -0.091414 0.101010

ENSMUST00000018478 2622 0.1535 0.4821 0.1703 0.4279 0.225 0.106 0.477 43.70 0.733 0.602 839 873 -0.517526 0.060710

ENSMUST00000014848 1119 0.2780 0.4271 0.1979 0.3633 0.236 0.085 0.475 51.41 0.620 0.543 366 372 -0.657796 0.069892

ENSMUST00000020830 1050 0.3172 0.3862 0.2472 0.2794 0.233 0.024 0.429 55.43 0.532 0.501 340 349 -0.031805 0.074499

ENSMUST00000021190 1416 0.2222 0.4548 0.2022 0.3593 0.238 0.091 0.462 46.25 0.651 0.567 455 471 -0.322293 0.070064

ENSMUST00000018392 720 0.3822 0.2670 0.4104 0.2215 0.194 -0.074 0.365 51.36 0.374 0.400 230 239 0.004602 0.104603

ENSMUST00000019317 705 0.2447 0.4202 0.2123 0.3765 0.228 -0.020 0.419 49.38 0.630 0.557 227 234 -0.232906 0.076923

ENSMUST00000021596 3519 0.2683 0.3950 0.1875 0.4227 0.236 0.077 0.463 51.10 0.639 0.544 1124 1172 -0.557765 0.088737

ENSMUST00000019464 1050 0.2491 0.3183 0.2792 0.3443 0.186 0.011 0.404 53.18 0.552 0.567 337 349 -0.314327 0.065903

ENSMUST00000019379 1635 0.2023 0.4000 0.2215 0.3877 0.215 0.102 0.470 48.04 0.646 0.593 526 544 -0.400184 0.064338

ENSMUST00000021930 969 0.2689 0.3902 0.2204 0.3378 0.222 0.098 0.470 58.85 0.589 0.520 304 322 0.012422 0.086957

ENSMUST00000020277 2748 0.1828 0.4599 0.1582 0.4767 0.251 0.106 0.479 45.16 0.726 0.560 873 915 -0.191803 0.068852

ENSMUST00000021370 1395 0.3532 0.3169 0.2936 0.2796 0.205 -0.001 0.412 58.97 0.469 0.488 456 464 -0.078664 0.090517

ENSMUST00000018765 1398 0.3342 0.3469 0.3873 0.2007 0.195 -0.004 0.422 54.70 0.435 0.456 448 465 -0.399140 0.144086

ENSMUST00000020822 879 0.3000 0.3773 0.1980 0.4757 0.298 0.118 0.491 50.33 0.617 0.494 277 292 -0.202740 0.113014

ENSMUST00000020971 879 0.2009 0.4821 0.2488 0.3854 0.282 0.138 0.500 50.81 0.655 0.557 278 292 -0.035617 0.082192

ENSMUST00000020024 399 0.3636 0.3131 0.2917 0.3636 0.296 0.022 0.449 57.97 0.496 0.457 127 132 -0.341667 0.083333

ENSMUST00000020527 591 0.3056 0.3611 0.3750 0.2879 0.225 -0.043 0.404 55.86 0.479 0.442 188 196 -0.425510 0.081633

ENSMUST00000015146 2460 0.3966 0.2496 0.3134 0.3119 0.226 0.011 0.416 53.89 0.427 0.449 791 819 -0.158852 0.069597

ENSMUST00000017552 1794 0.1841 0.4874 0.1795 0.3855 0.233 0.113 0.485 47.52 0.701 0.594 582 597 -0.705025 0.058626

ENSMUST00000021773 2529 0.2849 0.3897 0.2092 0.3432 0.240 0.121 0.490 50.53 0.590 0.523 808 842 -0.257363 0.112827

ENSMUST00000021922 804 0.1442 0.4791 0.1973 0.3733 0.205 0.147 0.494 46.65 0.710 0.617 259 267 -0.486142 0.071161

ENSMUST00000014647 1866 0.2970 0.4061 0.3000 0.3103 0.230 0.082 0.467 55.42 0.538 0.439 591 621 -0.008213 0.180354

ENSMUST00000015628 507 0.2276 0.4828 0.1938 0.3058 0.234 0.061 0.461 52.70 0.648 0.587 165 168 -0.141667 0.065476

ENSMUST00000019051 1989 0.2361 0.3916 0.1784 0.4421 0.201 0.045 0.424 50.06 0.661 0.556 632 662 -0.210423 0.086103

ENSMUST00000015583 786 0.2383 0.3738 0.2696 0.3421 0.189 0.092 0.462 52.51 0.578 0.536 251 261 -0.291954 0.068966

ENSMUST00000019199 2187 0.2120 0.4855 0.1404 0.4503 0.274 0.150 0.502 46.12 0.720 0.565 703 728 -0.353709 0.118132

ENSMUST00000019075 333 0.1609 0.5287 0.1310 0.4625 0.340 0.223 0.537 38.04 0.769 0.627 108 110 -0.565455 0.081818

ENSMUST00000021486 270 0.2623 0.2951 0.4412 0.3594 0.270 0.127 0.506 50.01 0.471 0.442 87 89 -0.556180 0.089888

ENSMUST00000021889 1005 0.4431 0.2667 0.3235 0.2615 0.254 0.011 0.441 52.40 0.397 0.441 315 334 -0.433234 0.110778

ENSMUST00000021155 1257 0.2107 0.4147 0.1835 0.5367 0.268 0.078 0.463 44.86 0.699 0.527 408 418 -0.733254 0.047847

ENSMUST00000018877 1080 0.3599 0.3114 0.2836 0.2727 0.240 0.090 0.459 53.53 0.471 0.494 344 359 -0.075209 0.114206

ENSMUST00000019044 1674 0.2260 0.4264 0.1938 0.3740 0.245 0.163 0.496 50.65 0.646 0.527 528 557 0.430700 0.131059

ENSMUST00000018531 2283 0.2590 0.3697 0.2016 0.3596 0.226 0.132 0.489 52.58 0.612 0.604 744 760 -0.723158 0.039474

ENSMUST00000020212 2079 0.3364 0.2126 0.4674 0.3025 0.206 0.016 0.427 54.44 0.386 0.420 677 692 -1.091.040 0.034682

ENSMUST00000020575 771 0.1560 0.5596 0.1534 0.3370 0.329 0.300 0.591 40.15 0.745 0.647 247 256 -0.109375 0.050781

ENSMUST00000021603 1347 0.2929 0.4301 0.2551 0.3209 0.279 0.127 0.501 51.37 0.572 0.521 435 448 -0.388170 0.093750

ENSMUST00000019058 501 0.3000 0.3714 0.2578 0.2893 0.222 0.138 0.481 52.24 0.537 0.498 162 166 -0.253012 0.048193

ENSMUST00000020343 669 0.2486 0.3526 0.3099 0.3797 0.233 0.026 0.452 61.00 0.558 0.508 217 222 -0.342342 0.067568

ENSMUST00000018985 990 0.2377 0.3472 0.2064 0.3941 0.213 0.060 0.439 54.30 0.621 0.572 319 329 0.048632 0.036474

ENSMUST00000017530 1413 0.2293 0.5147 0.1320 0.4085 0.277 0.146 0.500 42.35 0.714 0.588 458 470 -0.482979 0.089362

ENSMUST00000021234 6819 0.2018 0.4855 0.1350 0.3951 0.242 0.160 0.502 45.23 0.717 0.588 2184 2272 -0.132659 0.082306

ENSMUST00000015271 720 0.2623 0.3989 0.1900 0.3854 0.230 0.126 0.481 52.57 0.631 0.580 233 239 -0.500418 0.037657

ENSMUST00000019677 1248 0.2648 0.3894 0.2253 0.3961 0.242 0.067 0.457 51.73 0.610 0.544 405 415 -0.475422 0.069880

ENSMUST00000015277 6045 0.1920 0.4647 0.1902 0.4128 0.254 0.144 0.495 47.19 0.691 0.557 1935 2014 -0.192701 0.078947

ENSMUST00000020535 438 0.2927 0.4228 0.1667 0.3465 0.287 0.166 0.514 50.07 0.621 0.552 140 145 0.042069 0.089655

ENSMUST00000016901 1920 0.1815 0.5286 0.1203 0.4623 0.248 0.117 0.479 42.86 0.761 0.588 610 639 -0.376369 0.114241

ENSMUST00000019143 996 0.2436 0.4073 0.2137 0.3894 0.254 0.090 0.461 53.58 0.623 0.484 310 331 0.652870 0.151057

ENSMUST00000021900 2586 0.1922 0.4595 0.2166 0.3946 0.255 0.087 0.470 48.99 0.673 0.546 839 861 -0.285366 0.094077

ENSMUST00000017147 798 0.2593 0.3598 0.4085 0.2745 0.244 0.063 0.465 54.35 0.477 0.488 260 265 -0.678868 0.090566

ENSMUST00000021324 2829 0.1762 0.4699 0.2339 0.3589 0.233 0.117 0.480 45.52 0.664 0.591 913 942 -0.494268 0.056263

ENSMUST00000019291 1419 0.2853 0.3548 0.3361 0.2463 0.199 0.046 0.437 51.57 0.488 0.470 455 472 -0.131568 0.095339

ENSMUST00000017453 864 0.2366 0.4063 0.1754 0.3935 0.223 0.067 0.442 51.07 0.654 0.566 269 287 -0.188502 0.066202

ENSMUST00000019615 1140 0.1950 0.5145 0.1484 0.5311 0.308 0.080 0.478 40.68 0.751 0.568 358 379 -0.978100 0.073879

ENSMUST00000020023 765 0.2565 0.3194 0.3198 0.3622 0.202 -0.098 0.363 54.48 0.533 0.492 240 254 -0.313780 0.125984

ENSMUST00000020188 1434 0.3210 0.3077 0.2903 0.3192 0.221 -0.034 0.397 54.39 0.500 0.489 458 477 -0.285325 0.069182

ENSMUST00000021527 2052 0.2384 0.4418 0.2199 0.4081 0.238 0.065 0.459 51.64 0.642 0.525 656 683 -0.283602 0.098097

ENSMUST00000020931 3294 0.4396 0.1928 0.4605 0.2406 0.200 -0.038 0.405 50.42 0.315 0.388 1062 1097 -0.778578 0.060164

ENSMUST00000020278 1155 0.1824 0.5471 0.1338 0.3415 0.274 0.168 0.511 43.70 0.733 0.566 360 384 0.425781 0.138021

ENSMUST00000021036 1008 0.2201 0.4556 0.1753 0.4280 0.258 0.105 0.480 44.77 0.687 0.575 323 335 -0.430149 0.062687

ENSMUST00000021682 2004 0.2720 0.3927 0.2074 0.3902 0.237 0.095 0.464 51.79 0.615 0.561 631 667 -0.258921 0.095952

ENSMUST00000019633 588 0.2195 0.3963 0.1879 0.4056 0.267 0.212 0.524 44.77 0.658 0.600 187 195 -0.076410 0.087179

ENSMUST00000017946 1401 0.2835 0.3608 0.1983 0.3576 0.219 0.093 0.457 51.27 0.592 0.564 444 466 -0.179185 0.077253

ENSMUST00000019878 786 0.2022 0.3539 0.2952 0.4063 0.178 -0.028 0.397 46.84 0.599 0.590 257 261 -1.522.222 0.030651

ENSMUST00000021052 1596 0.2615 0.3874 0.1710 0.4203 0.223 0.096 0.464 48.76 0.644 0.582 506 531 -0.508851 0.064030

ENSMUST00000020734 2880 0.3435 0.2838 0.3291 0.2581 0.178 -0.021 0.398 56.11 0.443 0.507 931 959 -0.688738 0.045881

ENSMUST00000020497 2772 0.2573 0.3665 0.2727 0.3700 0.230 0.026 0.437 49.37 0.573 0.516 885 923 -0.194041 0.087757

ENSMUST00000020755 2463 0.4373 0.2186 0.4293 0.2248 0.224 -0.010 0.416 52.52 0.330 0.392 796 820 -0.415854 0.084146

ENSMUST00000020174 786 0.2989 0.3370 0.3302 0.3455 0.250 0.121 0.496 61.00 0.504 0.475 254 261 -0.604215 0.049808

ENSMUST00000021170 537 0.1393 0.4016 0.1812 0.5135 0.255 0.132 0.497 48.95 0.740 0.646 169 178 -0.784832 0.044944

ENSMUST00000018122 3915 0.2986 0.3364 0.2982 0.3566 0.218 0.015 0.425 56.98 0.531 0.491 1255 1304 -0.411043 0.085890

ENSMUST00000021147 2094 0.1851 0.5172 0.1344 0.4799 0.284 0.189 0.526 42.56 0.750 0.550 680 697 -0.546915 0.073171

ENSMUST00000018681 1029 0.2092 0.5071 0.1901 0.3187 0.223 0.064 0.455 48.13 0.672 0.569 332 342 -0.539181 0.064327

ENSMUST00000019950 1413 0.3522 0.3224 0.3305 0.3422 0.230 -0.036 0.407 55.43 0.488 0.467 459 470 -0.994894 0.070213

ENSMUST00000016125 1467 0.1789 0.4526 0.1519 0.4912 0.225 0.080 0.464 44.30 0.734 0.581 463 488 -0.528894 0.081967

ENSMUST00000020877 993 0.3665 0.2988 0.2734 0.3678 0.258 0.000 0.429 56.50 0.503 0.489 326 330 -0.650606 0.057576

ENSMUST00000021332 675 0.4375 0.2500 0.3276 0.3129 0.266 0.003 0.431 59.06 0.417 0.430 218 224 -0.750446 0.071429

ENSMUST00000015545 669 0.3422 0.2995 0.3602 0.2448 0.198 -0.025 0.390 57.63 0.427 0.455 213 222 0.055856 0.090090

ENSMUST00000015725 687 0.2179 0.4359 0.2434 0.4068 0.215 0.037 0.436 51.22 0.636 0.503 220 228 -0.457456 0.043860

ENSMUST00000018711 354 0.3034 0.4157 0.2706 0.3590 0.267 0.119 0.487 56.33 0.565 0.479 115 117 -0.534188 0.145299

ENSMUST00000019987 1668 0.2664 0.3634 0.2665 0.3397 0.185 0.025 0.413 53.81 0.563 0.547 538 555 -0.637117 0.068468

ENSMUST00000021832 1983 0.2237 0.4104 0.1634 0.4426 0.237 0.089 0.471 49.53 0.681 0.594 643 660 -0.439546 0.057576

ENSMUST00000018821 1080 0.2100 0.4533 0.1649 0.3345 0.233 0.163 0.501 44.31 0.674 0.591 341 359 0.100279 0.097493

ENSMUST00000021380 477 0.3051 0.2373 0.3590 0.4206 0.194 -0.033 0.404 56.70 0.483 0.432 151 158 -0.075950 0.094937

ENSMUST00000015934 921 0.2874 0.3158 0.2747 0.3348 0.172 0.036 0.429 58.15 0.530 0.532 287 306 -0.235948 0.098039

ENSMUST00000016124 1572 0.2630 0.3620 0.2819 0.3782 0.199 -0.021 0.395 53.39 0.569 0.504 506 523 -0.708987 0.059273

ENSMUST00000020753 1791 0.4332 0.2442 0.4169 0.2409 0.207 -0.031 0.404 50.04 0.349 0.385 570 596 -0.373154 0.083893

ENSMUST00000016396 159 0.1892 0.4054 0.2093 0.4872 0.254 0.066 0.480 45.31 0.680 0.519 50 52 -0.282692 0.096154

ENSMUST00000014694 2352 0.3121 0.3690 0.2447 0.3690 0.223 0.027 0.434 54.71 0.565 0.524 763 783 -0.771520 0.051086

ENSMUST00000020629 2049 0.2674 0.4176 0.2278 0.3675 0.244 0.105 0.477 53.13 0.602 0.503 664 682 -0.186657 0.074780

ENSMUST00000021045 1692 0.1567 0.4790 0.1553 0.4640 0.271 0.211 0.538 45.31 0.747 0.599 541 563 -0.308526 0.094139

ENSMUST00000021479 1254 0.3550 0.2426 0.3456 0.3010 0.204 0.004 0.402 57.11 0.425 0.456 405 417 -0.000480 0.074341

ENSMUST00000015667 1026 0.3660 0.3623 0.2522 0.3209 0.265 0.091 0.488 52.36 0.516 0.485 320 341 -0.519648 0.114369

ENSMUST00000021761 1542 0.2317 0.4181 0.1854 0.4556 0.224 0.066 0.454 51.27 0.669 0.558 493 513 -0.403704 0.083821

ENSMUST00000021466 1677 0.2998 0.3607 0.3077 0.3325 0.255 0.110 0.486 56.57 0.523 0.472 537 558 -0.294265 0.103943

ENSMUST00000017839 1254 0.2675 0.3769 0.1807 0.4123 0.219 0.090 0.463 52.16 0.632 0.563 397 417 -0.416547 0.069544

ENSMUST00000019192 591 0.2346 0.5556 0.0870 0.3740 0.307 0.229 0.550 40.90 0.735 0.571 189 196 -0.493878 0.117347

ENSMUST00000016571 213 0.2727 0.4909 0.1569 0.3333 0.303 0.297 0.585 52.23 0.646 0.524 65 70 -0.111428 0.128571

ENSMUST00000020257 2214 0.3727 0.2363 0.3462 0.3081 0.209 -0.025 0.405 55.92 0.420 0.491 726 737 -0.452917 0.055631

ENSMUST00000021939 1041 0.2473 0.4099 0.1538 0.4370 0.260 0.122 0.469 47.34 0.670 0.592 339 346 -0.083526 0.080925

ENSMUST00000015100 984 0.4286 0.2068 0.3810 0.2830 0.212 -0.027 0.397 48.22 0.363 0.426 317 327 -0.242813 0.103976

ENSMUST00000020397 381 0.2188 0.3438 0.3535 0.3371 0.205 0.038 0.445 55.49 0.529 0.500 119 126 -0.434127 0.039683

ENSMUST00000014457 231 0.4310 0.2586 0.1864 0.3750 0.277 0.030 0.444 46.76 0.500 0.504 72 76 -0.136842 0.118421

ENSMUST00000020372 171 0.1556 0.4444 0.1282 0.4722 0.234 0.155 0.490 29.08 0.755 0.607 49 56 -0.110714 0.160714

ENSMUST00000020262 867 0.2479 0.3950 0.2332 0.3602 0.228 0.076 0.459 54.94 0.605 0.547 281 288 -0.230555 0.090278

ENSMUST00000020853 321 0.3291 0.2911 0.3684 0.3099 0.209 -0.149 0.343 59.23 0.455 0.437 99 106 -0.553774 0.094340

ENSMUST00000021738 1158 0.2047 0.4955 0.1648 0.3636 0.237 0.104 0.462 50.79 0.696 0.530 372 385 0.345455 0.171429

ENSMUST00000017891 1599 0.2037 0.3611 0.2050 0.4101 0.176 0.024 0.405 48.30 0.651 0.602 513 532 -0.015414 0.062030

ENSMUST00000021714 705 0.2366 0.4140 0.1724 0.4294 0.270 0.095 0.478 49.35 0.670 0.594 224 234 -0.323504 0.064103

ENSMUST00000018743 1365 0.2249 0.3677 0.1326 0.4590 0.224 0.125 0.464 44.75 0.695 0.614 442 454 0.073568 0.070485

ENSMUST00000021606 1068 0.3668 0.2510 0.4073 0.2703 0.198 0.004 0.424 58.14 0.395 0.452 342 355 -0.676901 0.067606

ENSMUST00000018989 2790 0.2102 0.4219 0.1425 0.5066 0.276 0.109 0.476 45.95 0.718 0.565 904 929 -0.224866 0.044133

ENSMUST00000020283 1119 0.1312 0.5106 0.2190 0.3912 0.241 0.106 0.488 47.69 0.710 0.560 365 372 -0.420699 0.048387

ENSMUST00000017694 5481 0.4080 0.2175 0.4015 0.2554 0.208 -0.050 0.396 50.77 0.363 0.419 1795 1826 -0.758324 0.049836

ENSMUST00000018491 1671 0.2896 0.3342 0.2814 0.3952 0.218 0.018 0.434 52.29 0.558 0.526 539 556 -0.757734 0.071942

ENSMUST00000019065 3372 0.3341 0.2945 0.2386 0.3177 0.199 0.011 0.402 52.60 0.515 0.574 1102 1123 -0.260908 0.032947

ENSMUST00000019862 1065 0.2161 0.3742 0.2390 0.3855 0.236 0.039 0.436 50.73 0.622 0.582 349 354 0.031073 0.073446

ENSMUST00000020820 621 0.2830 0.3333 0.2875 0.3681 0.218 0.068 0.447 61.00 0.538 0.508 197 206 -0.468932 0.072816

ENSMUST00000017622 2919 0.2476 0.3666 0.2700 0.3527 0.178 -0.048 0.402 54.59 0.578 0.581 958 972 -1.500.309 0.038066

ENSMUST00000020323 1272 0.1798 0.4972 0.1474 0.3806 0.306 0.242 0.557 45.45 0.723 0.577 397 423 0.093617 0.118203

ENSMUST00000017332 327 0.2976 0.3095 0.2022 0.4118 0.207 0.110 0.481 39.89 0.587 0.586 104 108 -0.502778 0.074074

ENSMUST00000017153 597 0.1925 0.5031 0.1656 0.3836 0.240 0.125 0.474 49.25 0.706 0.577 194 198 -0.149495 0.065657

ENSMUST00000017821 1266 0.4375 0.2358 0.3390 0.2179 0.201 -0.028 0.397 56.80 0.362 0.458 398 421 -0.158432 0.099762

ENSMUST00000021130 486 0.2756 0.3386 0.1890 0.4194 0.174 -0.025 0.377 50.14 0.617 0.557 154 161 -0.236646 0.068323

ENSMUST00000018403 723 0.3743 0.2727 0.4615 0.1806 0.203 -0.039 0.388 49.48 0.348 0.408 227 240 -0.171667 0.108333

ENSMUST00000014118 552 0.2857 0.3759 0.2329 0.3796 0.199 -0.045 0.391 61.00 0.586 0.494 174 183 -0.509836 0.054645

ENSMUST00000014996 3114 0.2714 0.3972 0.1967 0.3533 0.243 0.103 0.477 49.49 0.611 0.581 997 1037 -0.284474 0.068467

ENSMUST00000020687 600 0.4013 0.2930 0.2147 0.3228 0.221 0.019 0.410 50.29 0.497 0.503 195 199 -0.417086 0.060302

ENSMUST00000020835 1797 0.2851 0.3459 0.2638 0.3259 0.202 0.020 0.427 57.36 0.545 0.535 571 598 -0.459532 0.053512

ENSMUST00000019683 987 0.2095 0.4466 0.1485 0.4978 0.250 0.062 0.460 43.54 0.720 0.605 311 328 -0.897561 0.094512

ENSMUST00000021345 1137 0.4007 0.2172 0.3696 0.3193 0.232 -0.051 0.394 47.04 0.405 0.481 368 378 -0.620899 0.037037

ENSMUST00000020383 4008 0.1889 0.4899 0.1574 0.4438 0.295 0.176 0.517 45.87 0.723 0.545 1270 1335 -0.117378 0.098127

ENSMUST00000020017 5076 0.2838 0.3851 0.2728 0.3096 0.225 0.066 0.461 55.65 0.550 0.521 1644 1691 -0.592904 0.062685

ENSMUST00000020165 1341 0.2918 0.3824 0.2540 0.3627 0.243 0.125 0.485 56.74 0.569 0.502 425 446 -0.337892 0.078475

ENSMUST00000021792 1869 0.2854 0.3229 0.3262 0.3424 0.202 -0.018 0.410 54.82 0.514 0.482 595 622 -0.652733 0.088424

ENSMUST00000014545 999 0.3727 0.3137 0.2269 0.3136 0.259 0.095 0.461 58.47 0.498 0.482 319 332 0.163253 0.054217

ENSMUST00000020965 1245 0.3578 0.3021 0.2590 0.3297 0.208 -0.005 0.409 58.61 0.492 0.496 396 414 -0.254831 0.086957

ENSMUST00000021645 1560 0.2447 0.4323 0.1922 0.3846 0.233 0.061 0.457 48.55 0.645 0.543 499 519 -0.432177 0.080925

ENSMUST00000020547 2598 0.4105 0.2566 0.3481 0.2407 0.196 -0.056 0.375 50.14 0.382 0.402 822 865 0.240231 0.129480

ENSMUST00000020769 1311 0.1975 0.4734 0.1966 0.4142 0.262 0.082 0.480 45.64 0.688 0.593 423 436 -0.871330 0.075688

ENSMUST00000019974 672 0.2529 0.4655 0.2303 0.3377 0.257 0.120 0.493 55.49 0.619 0.529 215 223 -0.376682 0.089686

ENSMUST00000015358 2010 0.4004 0.2309 0.3306 0.3154 0.199 -0.090 0.371 52.73 0.413 0.451 642 669 -0.324066 0.095665

ENSMUST00000019038 1731 0.1644 0.5320 0.1299 0.4573 0.246 0.110 0.480 42.80 0.763 0.580 558 576 -0.580382 0.052083

ENSMUST00000019224 1239 0.2322 0.3142 0.1914 0.3736 0.199 0.111 0.441 49.99 0.617 0.596 397 412 0.736165 0.070388

ENSMUST00000021030 1170 0.3505 0.3505 0.2226 0.3603 0.241 0.023 0.433 58.00 0.546 0.515 379 389 -0.435990 0.100257

ENSMUST00000020439 1140 0.2913 0.4013 0.2970 0.2920 0.210 0.017 0.434 51.37 0.538 0.536 366 379 -0.285488 0.084433

ENSMUST00000021554 2679 0.2152 0.4970 0.1628 0.4644 0.268 0.076 0.469 46.21 0.708 0.546 850 892 -0.600448 0.079596

ENSMUST00000018909 2025 0.3053 0.3565 0.2285 0.3321 0.196 0.020 0.431 54.09 0.558 0.555 661 674 -0.832789 0.057864

ENSMUST00000018963 993 0.2965 0.3673 0.2580 0.3626 0.260 0.079 0.481 54.50 0.565 0.546 322 330 -1.205.454 0.036364

ENSMUST00000020099 894 0.2532 0.3906 0.2780 0.3650 0.224 0.121 0.477 55.89 0.575 0.484 285 297 -0.243434 0.094276

ENSMUST00000021803 4389 0.4119 0.2284 0.3604 0.2000 0.225 -0.058 0.408 51.26 0.353 0.461 1446 1462 -0.470930 0.075239

ENSMUST00000021828 471 0.0873 0.4762 0.1008 0.5965 0.246 0.170 0.510 36.03 0.848 0.632 151 156 -0.109615 0.102564

ENSMUST00000020456 621 0.1566 0.5000 0.1104 0.4467 0.240 0.274 0.570 44.77 0.777 0.657 193 206 -0.748058 0.131068

ENSMUST00000020634 1272 0.3580 0.2901 0.3718 0.2711 0.240 0.009 0.424 56.90 0.424 0.444 403 423 -0.324113 0.082742

ENSMUST00000020346 654 0.3713 0.2695 0.4207 0.2222 0.197 0.000 0.414 51.50 0.376 0.415 210 217 -0.513825 0.069124

ENSMUST00000017548 591 0.2296 0.4667 0.1056 0.5034 0.194 0.037 0.435 43.57 0.742 0.549 186 196 -1.111.225 0.030612

ENSMUST00000020647 1008 0.3359 0.3047 0.2574 0.3457 0.217 0.030 0.440 50.03 0.523 0.555 327 335 -0.903582 0.047761

ENSMUST00000016023 2829 0.2295 0.3724 0.2401 0.4575 0.224 0.021 0.438 52.41 0.637 0.554 913 942 -1.006.157 0.033970

ENSMUST00000019516 777 0.1921 0.4236 0.2124 0.4309 0.193 -0.001 0.401 51.01 0.676 0.578 247 258 -0.329070 0.073643

ENSMUST00000020316 2190 0.3188 0.3207 0.3162 0.3445 0.226 0.004 0.420 58.08 0.503 0.467 700 729 -0.371193 0.085048

ENSMUST00000021428 1611 0.4042 0.2441 0.3381 0.3158 0.207 -0.002 0.426 53.63 0.424 0.478 516 536 -1.131.716 0.059701

ENSMUST00000016673 537 0.2016 0.4729 0.2500 0.4224 0.259 0.134 0.497 45.18 0.659 0.494 167 178 -0.287079 0.084270

ENSMUST00000021574 1905 0.4199 0.2186 0.3074 0.3795 0.199 -0.063 0.380 53.21 0.441 0.434 608 634 -0.778864 0.063091

ENSMUST00000017443 987 0.2204 0.3878 0.2374 0.4156 0.221 0.118 0.489 52.64 0.630 0.573 311 328 -0.722866 0.057927

ENSMUST00000020159 4113 0.3024 0.3443 0.2515 0.3765 0.228 0.028 0.420 53.62 0.554 0.489 1307 1370 -0.002409 0.097810

ENSMUST00000020000 618 0.2733 0.4000 0.2704 0.3581 0.233 -0.001 0.426 55.53 0.574 0.504 197 205 -0.660488 0.097561

ENSMUST00000015891 1713 0.3484 0.2896 0.2886 0.3578 0.204 0.007 0.414 53.60 0.494 0.463 555 570 -0.304386 0.080702

ENSMUST00000016344 1725 0.3326 0.2930 0.3473 0.2963 0.208 -0.014 0.416 51.13 0.458 0.463 553 574 -0.487456 0.085366

ENSMUST00000019854 651 0.2814 0.3473 0.3412 0.2562 0.177 -0.009 0.402 61.00 0.485 0.511 204 216 -0.656019 0.083333

ENSMUST00000020403 582 0.3245 0.3642 0.2409 0.3893 0.252 0.015 0.463 58.06 0.564 0.530 188 193 -0.626943 0.098446

ENSMUST00000021314 1491 0.2022 0.4528 0.2161 0.4344 0.260 0.088 0.466 48.75 0.674 0.530 470 496 -0.511895 0.090726

ENSMUST00000021734 228 0.0909 0.6727 0.1404 0.3962 0.271 0.141 0.507 34.45 0.817 0.582 71 75 -0.273333 0.053333

ENSMUST00000021639 1341 0.3471 0.3251 0.2818 0.3146 0.243 0.048 0.444 54.44 0.493 0.484 432 446 -0.076233 0.096413

ENSMUST00000014421 7812 0.3956 0.2134 0.3469 0.2474 0.208 -0.021 0.409 53.52 0.378 0.486 2537 2603 -0.395736 0.030734

ENSMUST00000015460 1032 0.2509 0.3970 0.2857 0.3333 0.246 0.079 0.464 53.90 0.569 0.497 332 343 -0.334111 0.069971

ENSMUST00000019198 459 0.2385 0.3853 0.1855 0.4706 0.300 0.152 0.503 42.13 0.667 0.539 147 152 -0.286184 0.078947

ENSMUST00000021089 957 0.1762 0.5287 0.2058 0.3392 0.261 0.190 0.527 49.28 0.691 0.559 311 318 -0.035849 0.122642

ENSMUST00000018685 1251 0.1942 0.4137 0.1808 0.5030 0.178 0.002 0.429 49.92 0.709 0.550 399 416 -1.489.423 0.040865

ENSMUST00000017365 1593 0.1854 0.4732 0.1691 0.4560 0.269 0.156 0.507 45.17 0.718 0.574 515 530 -0.537736 0.083019

ENSMUST00000018361 483 0.1571 0.5571 0.1513 0.3178 0.272 0.247 0.546 50.17 0.737 0.550 152 160 0.999375 0.150000

ENSMUST00000021249 1278 0.2252 0.3994 0.1642 0.4431 0.235 0.078 0.463 45.65 0.679 0.609 408 425 -0.273176 0.070588

ENSMUST00000018593 660 0.2651 0.3313 0.2545 0.4331 0.220 0.046 0.435 54.21 0.589 0.514 209 219 -0.317352 0.050228

ENSMUST00000015791 11157 0.2413 0.4568 0.1623 0.3681 0.252 0.126 0.490 46.77 0.668 0.602 3634 3718 -0.288973 0.069661

ENSMUST00000019276 690 0.3772 0.3353 0.2788 0.3595 0.257 0.074 0.459 60.31 0.505 0.448 220 229 -0.557642 0.104803

ENSMUST00000018522 1167 0.1967 0.4689 0.2000 0.3780 0.232 0.161 0.515 47.53 0.678 0.592 373 388 -0.846650 0.064433

ENSMUST00000021674 1143 0.2127 0.4571 0.1776 0.3547 0.243 0.097 0.478 47.91 0.673 0.586 370 380 -0.416579 0.065789

ENSMUST00000014892 591 0.1687 0.5120 0.1714 0.3952 0.296 0.234 0.543 46.36 0.720 0.522 186 196 0.835714 0.204082

ENSMUST00000016323 1434 0.2400 0.4667 0.2017 0.3650 0.254 0.100 0.477 49.00 0.646 0.532 461 477 -0.344235 0.079665

ENSMUST00000019998 582 0.1637 0.5906 0.1136 0.3421 0.284 0.268 0.568 41.90 0.765 0.584 183 193 0.691192 0.165803

ENSMUST00000021880 414 0.3608 0.2887 0.3617 0.3407 0.224 -0.027 0.414 44.81 0.461 0.445 128 137 -0.783942 0.109489

ENSMUST00000021630 972 0.4286 0.2612 0.3077 0.3241 0.234 -0.013 0.412 55.70 0.431 0.440 311 323 -0.418885 0.099071

ENSMUST00000020268 3441 0.4218 0.1861 0.4270 0.2743 0.213 -0.047 0.393 52.27 0.349 0.435 1116 1146 -1.003.927 0.058464

ENSMUST00000016138 1134 0.3239 0.3134 0.2826 0.3969 0.221 0.067 0.452 57.18 0.529 0.500 361 377 -0.692042 0.098143

ENSMUST00000020150 1038 0.2656 0.2852 0.3484 0.3545 0.200 -0.007 0.414 53.62 0.500 0.479 336 345 -0.470145 0.040580

ENSMUST00000020959 858 0.2294 0.3716 0.2398 0.4048 0.204 0.003 0.431 51.50 0.617 0.565 269 285 -0.634035 0.080702

ENSMUST00000019378 4236 0.4057 0.2445 0.3645 0.2632 0.213 -0.020 0.409 53.73 0.390 0.434 1373 1411 -0.542169 0.060950

ENSMUST00000020446 717 0.2689 0.3821 0.2126 0.3478 0.253 0.094 0.455 53.17 0.593 0.492 231 238 0.831512 0.147059

ENSMUST00000021907 1020 0.2868 0.3750 0.2060 0.3800 0.243 0.014 0.427 49.82 0.597 0.526 330 339 -0.164307 0.076696

ENSMUST00000018625 1758 0.3581 0.3122 0.3128 0.3342 0.266 0.047 0.450 57.88 0.482 0.460 571 585 -0.228718 0.107692

ENSMUST00000016087 414 0.1622 0.3874 0.2017 0.4052 0.194 0.027 0.432 53.84 0.682 0.640 132 137 -0.219708 0.021898

ENSMUST00000021692 1245 0.1708 0.5155 0.1715 0.4498 0.309 0.165 0.532 43.96 0.733 0.599 404 414 -0.763043 0.045894

ENSMUST00000021142 3117 0.2327 0.4042 0.1638 0.4143 0.258 0.075 0.452 46.25 0.666 0.577 996 1038 0.032948 0.066474

ENSMUST00000021443 2808 0.2614 0.3529 0.2470 0.3779 0.214 0.004 0.414 52.26 0.578 0.526 911 935 -0.071016 0.054545

ENSMUST00000018637 5829 0.2234 0.4648 0.1801 0.4962 0.308 0.107 0.493 45.14 0.699 0.530 1879 1942 -0.788980 0.055613

ENSMUST00000016640 873 0.2393 0.3504 0.2818 0.4039 0.204 -0.007 0.408 54.85 0.582 0.507 282 290 -0.180345 0.082759

ENSMUST00000020287 1299 0.1138 0.5664 0.0650 0.4408 0.277 0.263 0.554 37.70 0.845 0.640 406 432 0.260417 0.104167

ENSMUST00000021610 1392 0.2185 0.4172 0.2205 0.4468 0.245 0.015 0.444 47.70 0.662 0.593 450 463 -1.253.564 0.032397

ENSMUST00000020653 597 0.3567 0.2484 0.3469 0.3088 0.193 -0.020 0.399 53.61 0.431 0.451 188 198 -0.076263 0.101010

ENSMUST00000019572 1344 0.4759 0.2590 0.3935 0.2500 0.263 -0.050 0.418 48.14 0.364 0.416 440 447 -0.934228 0.107383

ENSMUST00000021217 459 0.2348 0.4435 0.1429 0.4950 0.304 0.107 0.479 40.56 0.701 0.553 144 152 -0.288158 0.098684

ENSMUST00000014684 720 0.2461 0.3979 0.2882 0.3681 0.199 0.062 0.470 53.74 0.586 0.536 232 239 -1.066.527 0.092050

ENSMUST00000021390 2055 0.3542 0.3148 0.2811 0.2936 0.220 0.029 0.439 57.86 0.479 0.486 645 684 -0.247368 0.135965

ENSMUST00000020909 927 0.2874 0.3793 0.2009 0.3561 0.230 0.086 0.452 56.43 0.589 0.523 292 308 0.225000 0.133117

ENSMUST00000021646 3843 0.2407 0.4312 0.1842 0.3611 0.215 0.100 0.479 48.63 0.647 0.619 1248 1280 -0.649141 0.064844

ENSMUST00000014389 759 0.2163 0.4135 0.1937 0.3934 0.233 0.128 0.475 52.49 0.658 0.561 240 252 0.157143 0.095238

ENSMUST00000015540 591 0.1938 0.4375 0.2037 0.3654 0.227 0.072 0.455 52.89 0.665 0.565 191 196 0.055612 0.081633

ENSMUST00000017142 342 0.4588 0.2235 0.2766 0.2667 0.181 -0.104 0.380 42.71 0.398 0.431 108 113 -0.716814 0.061947

ENSMUST00000015612 5895 0.2349 0.4510 0.2057 0.3284 0.230 0.104 0.481 47.36 0.639 0.624 1925 1964 -0.300153 0.045825

ENSMUST00000014913 723 0.3020 0.3465 0.2737 0.3152 0.233 0.073 0.466 52.52 0.526 0.501 232 240 -0.145417 0.091667

ENSMUST00000017309 306 0.2222 0.3750 0.2821 0.3896 0.249 0.196 0.537 40.03 0.600 0.574 95 101 -0.903960 0.079208

ENSMUST00000017455 297 0.0964 0.5542 0.1867 0.3514 0.232 0.217 0.521 41.65 0.766 0.633 94 98 -0.165306 0.102041

ENSMUST00000015467 975 0.2000 0.3491 0.2109 0.4046 0.229 0.123 0.457 53.12 0.641 0.595 315 324 0.743210 0.058642

ENSMUST00000017637 765 0.1626 0.5222 0.1198 0.4620 0.252 0.157 0.506 34.31 0.773 0.643 247 254 -0.535039 0.031496

ENSMUST00000014505 2985 0.2809 0.3787 0.2713 0.3188 0.220 0.044 0.437 54.64 0.549 0.502 956 994 -0.162475 0.078471

ENSMUST00000021187 1131 0.2391 0.3882 0.1694 0.3826 0.213 0.103 0.462 51.31 0.649 0.608 368 376 -0.148670 0.058511

ENSMUST00000021942 654 0.2050 0.4969 0.2024 0.3642 0.269 0.109 0.485 42.60 0.675 0.550 206 217 -0.467281 0.096774

ENSMUST00000019882 378 0.1753 0.5670 0.1375 0.5135 0.361 0.264 0.587 39.71 0.769 0.579 121 125 -0.733600 0.096000

ENSMUST00000021368 3195 0.4003 0.2047 0.4282 0.2824 0.225 -0.019 0.413 50.85 0.363 0.421 1031 1064 -0.713252 0.065789

ENSMUST00000016654 831 0.2732 0.3144 0.2864 0.4223 0.223 0.104 0.485 49.85 0.561 0.489 264 276 -0.899275 0.039855

ENSMUST00000020549 795 0.1972 0.4460 0.2255 0.3487 0.240 0.138 0.486 49.62 0.649 0.572 251 264 -0.189015 0.068182

ENSMUST00000021530 2511 0.4272 0.2291 0.3704 0.2462 0.222 -0.043 0.399 52.00 0.367 0.432 804 836 -0.586962 0.061005

ENSMUST00000018577 2172 0.3363 0.2785 0.3746 0.2769 0.198 -0.028 0.393 57.18 0.430 0.447 704 723 -0.097372 0.080221

ENSMUST00000020015 1173 0.2589 0.4196 0.1815 0.3623 0.220 0.076 0.443 54.53 0.629 0.499 377 390 0.384103 0.105128

ENSMUST00000018713 636 0.2235 0.4529 0.1772 0.3265 0.251 0.105 0.482 50.01 0.654 0.564 191 211 0.499526 0.094787

ENSMUST00000021343 738 0.2857 0.3619 0.2928 0.3232 0.199 0.096 0.463 56.42 0.533 0.464 242 245 -0.048163 0.097959

ENSMUST00000018506 1590 0.4048 0.2810 0.2912 0.2756 0.231 0.052 0.445 56.35 0.433 0.464 515 529 -0.173346 0.054820

ENSMUST00000016026 1803 0.3965 0.2423 0.4328 0.2009 0.202 -0.025 0.413 52.93 0.343 0.435 583 600 -0.682167 0.053333

ENSMUST00000016638 1149 0.3539 0.3149 0.2516 0.2881 0.231 0.079 0.473 53.66 0.492 0.503 370 382 -0.395812 0.052356

ENSMUST00000020707 1368 0.3154 0.3235 0.3609 0.2327 0.211 -0.008 0.409 50.23 0.448 0.457 433 455 -0.234286 0.114286

ENSMUST00000021652 1266 0.2147 0.4153 0.1858 0.4053 0.203 0.076 0.449 50.57 0.664 0.591 405 421 -0.114014 0.085511

ENSMUST00000015941 1092 0.2543 0.3368 0.2878 0.3598 0.218 0.017 0.429 49.89 0.556 0.545 347 363 -0.153444 0.093664

ENSMUST00000014640 3162 0.3934 0.2453 0.3765 0.2251 0.196 -0.105 0.360 52.28 0.374 0.455 1029 1053 -0.111681 0.050332

ENSMUST00000021669 597 0.3000 0.3533 0.3050 0.3798 0.227 0.040 0.437 61.00 0.537 0.463 190 198 -0.652525 0.080808

ENSMUST00000021860 489 0.3657 0.3881 0.2269 0.2752 0.250 0.105 0.481 47.29 0.519 0.486 158 162 -0.212346 0.086420

ENSMUST00000020672 387 0.2584 0.4831 0.1368 0.4773 0.336 0.117 0.512 51.54 0.702 0.516 121 128 -0.610937 0.109375

ENSMUST00000020779 2157 0.1993 0.4975 0.1595 0.3718 0.216 0.102 0.464 45.11 0.703 0.571 688 718 -0.310585 0.086351

ENSMUST00000019248 2607 0.2340 0.4419 0.2234 0.3657 0.235 0.070 0.462 50.77 0.632 0.533 838 868 -0.520392 0.082949

ENSMUST00000018800 582 0.2357 0.4500 0.1645 0.4483 0.276 0.042 0.452 46.33 0.688 0.566 186 193 -0.509844 0.067358

ENSMUST00000020580 3624 0.1561 0.4564 0.1458 0.4792 0.266 0.182 0.511 42.83 0.753 0.611 1160 1207 -0.333306 0.072908

ENSMUST00000020312 1053 0.1779 0.4164 0.2637 0.3822 0.199 0.039 0.429 52.87 0.639 0.562 338 350 -0.267429 0.085714

ENSMUST00000020157 3750 0.3730 0.3125 0.2862 0.3197 0.224 -0.010 0.414 55.64 0.479 0.462 1203 1249 -0.221457 0.087270

ENSMUST00000021207 651 0.1630 0.4837 0.1588 0.4024 0.251 0.164 0.505 45.99 0.731 0.611 212 216 -0.250463 0.074074

ENSMUST00000019071 351 0.3196 0.4227 0.2727 0.2368 0.210 0.082 0.474 56.98 0.518 0.483 114 116 -0.240517 0.094828

ENSMUST00000020538 3216 0.4716 0.1704 0.4165 0.2493 0.196 -0.100 0.353 48.99 0.307 0.378 1019 1071 -0.080579 0.089636

ENSMUST00000021567 7035 0.2882 0.3878 0.2178 0.3273 0.224 0.064 0.456 53.79 0.579 0.531 2285 2344 -0.294539 0.078925

ENSMUST00000015137 1944 0.1936 0.4962 0.1801 0.3941 0.250 0.131 0.488 46.51 0.699 0.573 625 647 -0.373107 0.074189

ENSMUST00000016951 1149 0.3414 0.3414 0.2887 0.3245 0.262 0.050 0.454 48.92 0.505 0.455 366 382 -0.153665 0.096859

ENSMUST00000019006 1791 0.1847 0.4900 0.1367 0.4093 0.266 0.200 0.519 44.83 0.730 0.574 563 596 0.360403 0.105705

ENSMUST00000020566 1128 0.2430 0.4085 0.1803 0.4118 0.220 0.055 0.440 52.98 0.655 0.555 359 375 -0.702133 0.072000

ENSMUST00000021001 603 0.2273 0.4156 0.3493 0.3281 0.224 0.025 0.448 55.22 0.552 0.460 192 200 -0.330000 0.090000

ENSMUST00000021693 921 0.1931 0.4402 0.1566 0.3908 0.257 0.140 0.490 44.76 0.699 0.614 296 306 0.188235 0.075163

ENSMUST00000020220 1977 0.2311 0.4754 0.1680 0.3797 0.234 0.096 0.473 49.87 0.674 0.556 639 658 -0.628268 0.063830

ENSMUST00000021857 1356 0.2885 0.4118 0.1900 0.4007 0.258 0.121 0.484 51.09 0.622 0.531 434 451 -0.352771 0.113082

ENSMUST00000021956 1869 0.2268 0.4622 0.1744 0.4292 0.273 0.100 0.474 48.07 0.681 0.562 599 622 -0.419293 0.054662

ENSMUST00000021522 459 0.1475 0.4344 0.0992 0.5603 0.330 0.321 0.601 40.70 0.797 0.640 148 152 -0.240132 0.059211

ENSMUST00000021970 300 0.0714 0.5857 0.0800 0.5694 0.288 0.233 0.548 33.78 0.882 0.596 93 99 -0.606061 0.080808

ENSMUST00000021632 972 0.3589 0.3024 0.3106 0.3527 0.231 -0.003 0.408 53.78 0.487 0.458 316 323 -0.400000 0.102167

ENSMUST00000019482 3714 0.3255 0.3606 0.2112 0.3041 0.222 0.019 0.429 51.08 0.550 0.572 1214 1237 -0.479143 0.040420

ENSMUST00000020234 636 0.1988 0.5542 0.1408 0.3926 0.270 0.173 0.525 43.13 0.732 0.555 198 211 -0.303318 0.137441

ENSMUST00000019723 501 0.1692 0.4462 0.2105 0.4000 0.230 -0.021 0.425 42.08 0.688 0.584 160 166 -0.153614 0.114458

ENSMUST00000020378 2010 0.2230 0.4281 0.2317 0.3454 0.218 0.126 0.484 51.28 0.627 0.534 638 669 -0.353214 0.116592

ENSMUST00000021647 1815 0.3299 0.3549 0.2854 0.2564 0.236 0.028 0.440 55.51 0.495 0.531 588 604 -0.544702 0.072848

ENSMUST00000021929 1239 0.2138 0.3994 0.2454 0.3875 0.195 0.020 0.441 50.74 0.629 0.600 399 412 -1.258.010 0.053398

ENSMUST00000019932 723 0.2686 0.3200 0.2984 0.3967 0.212 -0.020 0.403 55.01 0.554 0.472 233 240 -0.741667 0.087500

ENSMUST00000021157 396 0.3846 0.2967 0.2444 0.4773 0.237 -0.021 0.413 46.57 0.548 0.471 126 131 -0.606107 0.145038

ENSMUST00000021307 714 0.2486 0.3220 0.2396 0.4415 0.203 0.043 0.435 53.11 0.609 0.551 230 237 -0.507173 0.067511

ENSMUST00000014370 690 0.3613 0.2710 0.3687 0.3373 0.235 -0.033 0.407 50.71 0.448 0.410 221 229 -0.851965 0.061135

ENSMUST00000019734 753 0.2160 0.4366 0.1263 0.4333 0.293 0.227 0.535 48.75 0.710 0.564 241 250 0.617200 0.152000

ENSMUST00000021158 372 0.2727 0.4205 0.3034 0.3690 0.310 0.033 0.454 44.55 0.571 0.491 119 123 -0.397561 0.113821

ENSMUST00000020437 2127 0.3061 0.2913 0.2984 0.3536 0.198 0.021 0.433 54.44 0.514 0.513 691 708 -0.989407 0.057910

ENSMUST00000014920 663 0.1765 0.3660 0.2872 0.4118 0.218 0.024 0.421 49.96 0.621 0.635 214 220 -1.214.091 0.031818

ENSMUST00000020664 1860 0.2637 0.4346 0.2472 0.3610 0.259 0.100 0.478 52.59 0.603 0.506 594 619 -0.445557 0.117932

ENSMUST00000015484 1713 0.3246 0.3640 0.2512 0.3565 0.235 0.014 0.427 53.00 0.541 0.475 543 570 -0.000702 0.129825

ENSMUST00000020273 2340 0.2987 0.3459 0.2222 0.3899 0.193 -0.016 0.400 54.33 0.574 0.517 760 779 -0.232863 0.075738

ENSMUST00000020217 540 0.2701 0.3139 0.2643 0.4254 0.185 -0.023 0.402 59.26 0.575 0.527 174 179 -0.584358 0.083799

ENSMUST00000021920 1422 0.3208 0.3342 0.2400 0.3790 0.238 0.034 0.432 57.07 0.549 0.499 463 473 -0.045243 0.078224

ENSMUST00000019913 1539 0.3418 0.3010 0.3610 0.2968 0.231 -0.011 0.414 54.89 0.451 0.455 490 512 -0.416992 0.117188

ENSMUST00000019708 1806 0.2258 0.4344 0.1829 0.3642 0.229 0.067 0.460 47.13 0.658 0.608 576 601 -0.549584 0.041597

ENSMUST00000017576 1125 0.2799 0.3994 0.2310 0.2986 0.207 -0.022 0.410 54.54 0.574 0.584 366 374 -0.564706 0.114973

ENSMUST00000018914 426 0.4080 0.2560 0.2414 0.2476 0.226 0.045 0.445 54.74 0.423 0.494 137 141 0.567376 0.092199

ENSMUST00000017629 4839 0.4870 0.1788 0.3980 0.2516 0.241 -0.056 0.399 49.53 0.319 0.395 1559 1612 -0.651179 0.083747

ENSMUST00000018287 471 0.1810 0.4655 0.1232 0.4519 0.210 0.011 0.418 41.19 0.752 0.603 153 156 -0.669872 0.025641

ENSMUST00000021668 450 0.2373 0.5000 0.1404 0.4175 0.304 0.233 0.548 43.87 0.699 0.537 146 149 -0.093289 0.073826

ENSMUST00000018113 1506 0.2272 0.4148 0.1759 0.4016 0.223 0.098 0.456 49.26 0.666 0.572 476 501 -0.200599 0.095808

ENSMUST00000020077 1191 0.3454 0.4013 0.2819 0.3525 0.251 -0.023 0.422 57.65 0.539 0.492 386 396 -0.509344 0.101010

ENSMUST00000021818 861 0.3317 0.2780 0.4083 0.2892 0.212 0.006 0.425 55.64 0.425 0.431 273 286 -0.482168 0.087413

ENSMUST00000021628 972 0.4190 0.2767 0.3377 0.2701 0.221 -0.033 0.394 54.67 0.410 0.431 310 323 -0.255418 0.099071

ENSMUST00000015456 483 0.1532 0.4516 0.1557 0.5217 0.298 0.218 0.539 50.83 0.753 0.590 154 160 -0.042500 0.043750

ENSMUST00000021328 447 0.2623 0.4180 0.2717 0.3902 0.289 0.087 0.471 58.36 0.593 0.477 140 148 0.045946 0.121622

ENSMUST00000021892 996 0.3898 0.2638 0.3872 0.2466 0.232 0.026 0.442 52.95 0.391 0.451 312 331 -0.621450 0.111782

ENSMUST00000018691 1251 0.1957 0.5186 0.1577 0.4417 0.294 0.151 0.512 45.04 0.726 0.540 402 416 -0.483894 0.093750

ENSMUST00000018644 999 0.1959 0.4708 0.1331 0.4089 0.248 0.131 0.480 50.87 0.718 0.562 319 332 0.751506 0.102410

ENSMUST00000020507 624 0.2215 0.4747 0.1266 0.4737 0.244 0.063 0.460 44.75 0.728 0.565 202 207 -0.713044 0.096618

ENSMUST00000020969 1344 0.2327 0.3518 0.1872 0.4566 0.237 0.164 0.498 51.83 0.654 0.598 436 447 -0.346309 0.058166

ENSMUST00000020334 2946 0.3620 0.3203 0.3638 0.2857 0.223 0.013 0.435 56.19 0.449 0.439 945 981 -0.674618 0.100917

ENSMUST00000021173 1350 0.2864 0.4092 0.1988 0.3254 0.239 0.077 0.461 51.50 0.590 0.497 434 449 0.617817 0.142539

ENSMUST00000021311 2505 0.2309 0.4526 0.1869 0.3661 0.233 0.088 0.465 49.41 0.659 0.576 817 834 -0.582734 0.047962

ENSMUST00000017911 681 0.2688 0.3226 0.1875 0.3656 0.194 0.064 0.439 45.05 0.598 0.622 214 226 -0.122124 0.044248

ENSMUST00000014174 1176 0.2179 0.4239 0.2190 0.3356 0.205 0.093 0.470 52.62 0.629 0.575 383 391 -0.521739 0.069054

ENSMUST00000021423 1101 0.2973 0.3784 0.2527 0.3440 0.210 0.062 0.439 52.51 0.558 0.483 355 366 -0.018306 0.092896

ENSMUST00000020329 3633 0.2701 0.4241 0.2834 0.2993 0.234 0.069 0.458 53.83 0.561 0.508 1169 1210 -0.316116 0.072727

ENSMUST00000020493 1290 0.1922 0.4414 0.1625 0.4767 0.219 0.059 0.448 46.75 0.714 0.577 406 429 -0.379953 0.116550

ENSMUST00000021940 1077 0.1852 0.5051 0.1344 0.4167 0.269 0.204 0.534 47.48 0.737 0.590 339 358 -0.401117 0.111732

ENSMUST00000019333 1992 0.2716 0.4239 0.1700 0.3684 0.265 0.174 0.501 51.96 0.632 0.519 631 663 0.478130 0.116139

ENSMUST00000021834 1128 0.3415 0.3484 0.2688 0.3258 0.259 0.062 0.457 54.90 0.518 0.469 359 375 -0.157600 0.088000

ENSMUST00000016672 1161 0.2389 0.4403 0.1667 0.4407 0.262 0.119 0.482 49.50 0.676 0.545 367 386 -0.468135 0.080311

ENSMUST00000021381 2181 0.3676 0.2444 0.3349 0.3274 0.177 -0.041 0.407 51.03 0.450 0.487 713 726 -1.434.160 0.016529

ENSMUST00000021359 1584 0.3611 0.2894 0.3165 0.3005 0.227 0.012 0.422 57.55 0.455 0.457 516 527 -0.129981 0.111954

ENSMUST00000020496 2106 0.2115 0.4493 0.1953 0.3796 0.225 0.072 0.457 48.88 0.666 0.560 682 701 -0.439230 0.071327

ENSMUST00000021426 1419 0.2884 0.3827 0.2717 0.3514 0.245 0.078 0.464 54.38 0.559 0.493 463 472 -0.570763 0.091102

ENSMUST00000021071 1323 0.1769 0.5174 0.1463 0.3529 0.256 0.128 0.480 44.12 0.725 0.577 415 440 0.376363 0.111364

ENSMUST00000020112 4374 0.3829 0.2861 0.3059 0.2867 0.232 0.032 0.440 55.72 0.446 0.459 1409 1457 -0.446328 0.060398

ENSMUST00000014248 435 0.4775 0.2342 0.3883 0.1702 0.248 -0.015 0.407 56.85 0.311 0.387 135 144 0.145833 0.090278

ENSMUST00000021356 2445 0.2057 0.3994 0.2052 0.4218 0.237 0.116 0.484 51.36 0.663 0.605 804 814 -0.567076 0.054054

ENSMUST00000020524 2571 0.1969 0.4907 0.1318 0.4227 0.243 0.139 0.492 45.56 0.729 0.598 824 856 -0.254089 0.102804

ENSMUST00000019302 567 0.2236 0.3540 0.1623 0.3816 0.230 0.160 0.500 42.29 0.653 0.674 176 188 0.092021 0.090426

ENSMUST00000017126 2919 0.3146 0.3337 0.2400 0.2618 0.173 -0.001 0.410 51.52 0.513 0.540 946 972 -0.382099 0.048354

ENSMUST00000020662 1422 0.2506 0.4816 0.1686 0.3160 0.244 0.088 0.475 49.50 0.651 0.574 459 473 -0.263636 0.105708

ENSMUST00000017270 1359 0.2069 0.4828 0.1077 0.4511 0.318 0.220 0.550 38.56 0.745 0.602 436 452 -0.531416 0.053097

ENSMUST00000021338 435 0.4071 0.2920 0.2475 0.3933 0.243 0.047 0.439 55.82 0.489 0.412 139 144 0.073611 0.111111

ENSMUST00000018569 2340 0.3827 0.2052 0.4086 0.2699 0.175 -0.086 0.362 50.83 0.364 0.433 756 779 -0.337997 0.073171

ENSMUST00000019101 2718 0.4251 0.2133 0.4315 0.1726 0.186 -0.123 0.358 46.52 0.307 0.455 880 905 -0.862320 0.067403

ENSMUST00000019447 654 0.2381 0.4082 0.2209 0.4847 0.256 0.031 0.453 44.01 0.656 0.525 212 217 -0.827189 0.064516

ENSMUST00000021685 2376 0.1809 0.4838 0.1358 0.4173 0.253 0.185 0.520 44.06 0.736 0.636 769 791 -0.381416 0.093552

ENSMUST00000021302 297 0.2564 0.3077 0.1818 0.4857 0.229 -0.002 0.413 39.57 0.630 0.544 92 98 -0.090816 0.081633

ENSMUST00000020655 2490 0.2178 0.4006 0.2100 0.4216 0.208 0.013 0.422 51.10 0.654 0.577 797 829 -0.666707 0.050663

ENSMUST00000021667 465 0.1953 0.4375 0.2734 0.2951 0.176 0.047 0.441 54.53 0.605 0.552 152 154 -0.050000 0.071429

ENSMUST00000019614 2568 0.2412 0.4455 0.1719 0.4383 0.279 0.113 0.484 47.06 0.675 0.563 819 855 -0.547018 0.105263

ENSMUST00000021271 3876 0.2691 0.4175 0.1890 0.3067 0.223 0.083 0.462 48.76 0.610 0.605 1266 1291 -0.535476 0.048025

ENSMUST00000019469 1074 0.2384 0.4603 0.1892 0.3376 0.215 0.099 0.459 47.89 0.644 0.519 340 357 0.464986 0.151261

ENSMUST00000020027 1362 0.3394 0.3133 0.3022 0.2637 0.215 0.010 0.413 59.70 0.465 0.458 424 453 0.476600 0.128035

ENSMUST00000020908 819 0.3000 0.2810 0.3364 0.3706 0.211 0.043 0.434 56.16 0.494 0.480 267 272 -0.638971 0.040441

ENSMUST00000020554 1218 0.1946 0.4491 0.1935 0.3354 0.253 0.165 0.496 45.62 0.666 0.607 389 405 -0.029136 0.061728

ENSMUST00000021018 1674 0.4076 0.2248 0.4433 0.1851 0.152 -0.164 0.383 43.69 0.326 0.497 549 557 -1.539.138 0.116697

ENSMUST00000021719 177 0.3810 0.3571 0.3409 0.1750 0.215 0.011 0.415 47.24 0.415 0.437 53 58 -0.132759 0.120690

ENSMUST00000016399 1356 0.2274 0.4548 0.1969 0.3967 0.266 0.129 0.498 49.78 0.660 0.546 432 451 -0.230155 0.088692

ENSMUST00000019901 2151 0.2988 0.3361 0.2625 0.4113 0.232 0.040 0.446 55.85 0.571 0.506 690 716 -0.745112 0.027933

ENSMUST00000020576 885 0.3451 0.2389 0.3363 0.3697 0.208 0.005 0.413 54.96 0.462 0.434 286 294 -0.120408 0.085034

ENSMUST00000020884 1314 0.2537 0.4060 0.2360 0.3832 0.248 0.051 0.443 51.50 0.611 0.506 424 437 -0.283753 0.086957

ENSMUST00000021135 1848 0.3408 0.2873 0.2951 0.3570 0.204 -0.044 0.399 58.95 0.498 0.493 592 615 -1.038.699 0.047154

ENSMUST00000021375 2298 0.3914 0.2516 0.3310 0.2825 0.195 -0.041 0.390 56.12 0.415 0.465 731 765 -0.235294 0.095425

ENSMUST00000021459 639 0.2179 0.4359 0.2112 0.4694 0.298 0.176 0.532 50.33 0.668 0.533 205 212 -0.571226 0.089623

ENSMUST00000021558 1677 0.2135 0.4472 0.2103 0.3813 0.218 0.040 0.439 49.31 0.654 0.554 535 558 -0.376882 0.086022

ENSMUST00000019382 927 0.1969 0.5354 0.1055 0.4229 0.281 0.185 0.517 42.13 0.752 0.555 294 308 0.011039 0.162338

ENSMUST00000021239 924 0.2136 0.4000 0.1360 0.5363 0.269 0.078 0.450 49.25 0.732 0.583 302 307 -0.748209 0.026059

ENSMUST00000016664 2064 0.1655 0.4965 0.1764 0.4036 0.240 0.147 0.498 46.60 0.720 0.588 671 687 -0.317176 0.046579

ENSMUST00000020821 1461 0.3438 0.3333 0.2966 0.2952 0.224 0.028 0.440 54.72 0.488 0.477 461 486 -0.229424 0.102881

ENSMUST00000021820 1122 0.3838 0.2222 0.4525 0.2583 0.189 -0.067 0.368 52.69 0.355 0.384 361 373 -0.301877 0.080429

ENSMUST00000019937 2283 0.3858 0.2397 0.3952 0.3019 0.211 -0.023 0.407 54.34 0.400 0.428 727 760 -0.654342 0.089474

ENSMUST00000018737 909 0.2247 0.5374 0.1280 0.4646 0.333 0.139 0.503 40.90 0.733 0.562 292 302 -0.425166 0.082781

ENSMUST00000019323 1017 0.2607 0.4500 0.1715 0.3360 0.276 0.107 0.477 50.55 0.637 0.555 331 338 0.117752 0.050296

ENSMUST00000020289 2580 0.1971 0.4691 0.1218 0.4658 0.240 0.094 0.462 42.09 0.741 0.592 835 859 -0.257276 0.081490

ENSMUST00000016072 4395 0.2138 0.3925 0.2321 0.4764 0.242 -0.076 0.407 50.38 0.660 0.561 1442 1464 -1.192.418 0.013661

ENSMUST00000021114 1179 0.2025 0.4112 0.1667 0.4103 0.207 0.031 0.425 51.53 0.686 0.614 379 392 -0.008674 0.063776

ENSMUST00000019954 519 0.3385 0.3308 0.4046 0.2276 0.206 -0.014 0.411 60.20 0.423 0.461 168 172 -0.754651 0.069767

ENSMUST00000021539 1323 0.3223 0.3253 0.3293 0.3165 0.214 0.051 0.454 55.09 0.489 0.483 425 440 -0.708636 0.077273

ENSMUST00000021513 771 0.0773 0.5411 0.0882 0.5124 0.277 0.257 0.566 36.75 0.863 0.674 249 256 -0.564844 0.074219

ENSMUST00000021547 7590 0.2428 0.4165 0.2251 0.3601 0.229 0.087 0.460 50.91 0.620 0.554 2452 2529 -0.285963 0.063266

ENSMUST00000021776 735 0.3895 0.2947 0.3218 0.2893 0.219 0.002 0.409 57.94 0.440 0.413 232 244 0.111475 0.102459

ENSMUST00000017193 672 0.2656 0.4219 0.3234 0.3836 0.240 0.079 0.478 52.59 0.567 0.498 203 223 -1.045.292 0.156951

ENSMUST00000018992 1983 0.3207 0.3267 0.2945 0.3792 0.235 0.058 0.451 54.83 0.521 0.463 637 660 -0.301060 0.087879

ENSMUST00000014065 1644 0.2500 0.4085 0.2079 0.3527 0.246 0.099 0.470 51.06 0.621 0.583 530 547 -0.374589 0.065814

ENSMUST00000015998 1059 0.2698 0.3489 0.2702 0.4100 0.237 -0.005 0.421 55.58 0.579 0.545 337 352 -0.407671 0.065341

ENSMUST00000014750 945 0.3156 0.3954 0.1457 0.3291 0.255 0.180 0.520 49.73 0.603 0.551 300 314 0.077707 0.098726

ENSMUST00000020227 1821 0.3491 0.3470 0.2596 0.2940 0.205 -0.008 0.413 54.69 0.505 0.507 576 606 -0.392574 0.103960

ENSMUST00000021379 810 0.4106 0.1691 0.3615 0.3024 0.179 -0.079 0.355 53.30 0.375 0.461 259 269 -0.410781 0.074349

ENSMUST00000019608 1218 0.1897 0.4011 0.1538 0.3578 0.234 0.190 0.501 45.66 0.685 0.646 387 405 0.479753 0.059259

ENSMUST00000019938 1158 0.2808 0.3785 0.2584 0.3179 0.214 0.095 0.472 57.10 0.557 0.518 375 385 -0.181039 0.075325

ENSMUST00000020362 576 0.2027 0.4797 0.1901 0.4030 0.239 0.061 0.445 51.16 0.687 0.534 182 191 -0.004712 0.109948

ENSMUST00000016383 2262 0.2710 0.3465 0.2857 0.3712 0.212 0.003 0.419 58.47 0.559 0.535 735 753 -0.421116 0.061089

ENSMUST00000018212 3597 0.3683 0.2665 0.2776 0.3193 0.195 0.003 0.401 55.32 0.465 0.474 1150 1198 0.097579 0.059265

ENSMUST00000021592 1317 0.3138 0.3226 0.2584 0.4000 0.203 -0.009 0.425 53.56 0.551 0.507 428 438 -0.918493 0.073059

ENSMUST00000021282 4014 0.2877 0.3562 0.2095 0.3590 0.223 0.047 0.437 51.61 0.585 0.579 1289 1337 -0.194166 0.070307

ENSMUST00000020107 3663 0.3427 0.2769 0.3379 0.3097 0.219 0.003 0.417 56.70 0.450 0.458 1181 1220 -0.167295 0.068033

ENSMUST00000021913 945 0.2539 0.3242 0.2687 0.3468 0.189 0.018 0.418 61.00 0.552 0.548 306 314 0.047771 0.035032

ENSMUST00000019283 1674 0.1783 0.4650 0.1316 0.4361 0.256 0.146 0.490 44.19 0.739 0.607 541 557 -0.152962 0.064632

ENSMUST00000021536 744 0.3068 0.2898 0.3382 0.3568 0.241 0.082 0.467 56.62 0.487 0.459 240 247 -0.438057 0.060729

ENSMUST00000014990 531 0.1832 0.5267 0.1533 0.4609 0.311 0.057 0.486 43.01 0.740 0.566 173 176 -0.634659 0.073864

ENSMUST00000020896 615 0.3149 0.3646 0.1600 0.3881 0.253 0.167 0.503 51.99 0.593 0.511 199 204 0.728921 0.112745

ENSMUST00000014438 300 0.2564 0.3846 0.1341 0.4487 0.259 0.137 0.490 45.42 0.677 0.589 96 99 -0.212121 0.050505

ENSMUST00000016696 1998 0.1818 0.5134 0.1656 0.3879 0.275 0.208 0.535 45.55 0.719 0.571 641 665 -0.286617 0.117293

ENSMUST00000018012 1104 0.2178 0.4686 0.2061 0.3911 0.249 0.103 0.474 49.47 0.666 0.543 359 367 -0.336240 0.106267

ENSMUST00000021029 1299 0.2938 0.3442 0.3516 0.3217 0.188 -0.045 0.401 59.72 0.500 0.438 416 432 -0.576389 0.101852

ENSMUST00000021242 387 0.2157 0.3039 0.2544 0.3945 0.151 -0.011 0.392 46.02 0.592 0.570 125 128 -0.192969 0.039063

ENSMUST00000021958 411 0.2075 0.3302 0.2095 0.4851 0.276 0.158 0.523 55.42 0.656 0.578 128 136 -0.122794 0.095588

ENSMUST00000015160 1752 0.2277 0.4021 0.1875 0.3959 0.235 0.105 0.466 48.69 0.652 0.577 558 583 -0.194854 0.087479

ENSMUST00000015981 1215 0.2539 0.4025 0.1922 0.4000 0.245 0.085 0.473 47.55 0.638 0.583 389 404 -0.634654 0.069307

ENSMUST00000020704 819 0.2765 0.4194 0.2222 0.3216 0.248 0.075 0.464 53.77 0.594 0.580 261 272 -0.217279 0.077206

ENSMUST00000020391 804 0.1943 0.4882 0.1659 0.4293 0.235 0.057 0.458 46.18 0.712 0.572 260 267 -0.262547 0.089888

ENSMUST00000021948 1794 0.2306 0.4571 0.1859 0.3850 0.292 0.214 0.548 47.46 0.666 0.589 575 597 -0.369514 0.087102

ENSMUST00000017836 1215 0.1717 0.4669 0.1316 0.4549 0.277 0.192 0.514 43.32 0.747 0.595 383 404 0.190841 0.108911

ENSMUST00000021209 1239 0.1712 0.5495 0.1424 0.4103 0.260 0.122 0.489 41.71 0.749 0.591 403 412 -0.681553 0.067961

ENSMUST00000017567 1395 0.4366 0.1239 0.4514 0.1886 0.182 -0.150 0.317 45.97 0.255 0.484 432 464 -0.676724 0.047414

ENSMUST00000019631 1644 0.2578 0.4271 0.1875 0.4429 0.246 0.072 0.470 48.57 0.660 0.559 530 547 -0.995612 0.071298

ENSMUST00000019808 1392 0.2271 0.3490 0.1694 0.4634 0.245 0.056 0.441 43.34 0.672 0.616 442 463 -0.320086 0.038877

ENSMUST00000021251 972 0.2531 0.4066 0.2287 0.3959 0.259 0.124 0.492 50.29 0.619 0.528 315 323 -0.611765 0.037152

ENSMUST00000014683 801 0.3827 0.2908 0.4348 0.2398 0.227 -0.003 0.431 47.40 0.387 0.398 253 266 -0.574060 0.109023

ENSMUST00000021020 1533 0.2019 0.4513 0.1974 0.3564 0.255 0.173 0.513 47.96 0.668 0.612 485 510 -0.410196 0.119608

ENSMUST00000021412 741 0.4000 0.2842 0.3579 0.2312 0.278 0.063 0.466 53.69 0.395 0.444 238 246 -0.166667 0.089431

ENSMUST00000021796 609 0.2403 0.4286 0.2885 0.3200 0.206 0.056 0.454 46.01 0.582 0.507 196 202 -0.575248 0.074257

ENSMUST00000019076 1044 0.2022 0.4607 0.1654 0.4603 0.286 0.151 0.503 45.13 0.706 0.544 330 347 -0.323631 0.077810

ENSMUST00000020408 1470 0.3577 0.3089 0.2834 0.3511 0.253 0.024 0.447 54.01 0.501 0.481 477 489 -0.827198 0.055215

ENSMUST00000020831 1473 0.3370 0.2597 0.3269 0.3182 0.214 -0.090 0.374 53.86 0.460 0.488 478 490 -0.419184 0.044898

ENSMUST00000014022 861 0.3632 0.2778 0.3824 0.2418 0.159 -0.063 0.364 53.41 0.401 0.435 272 286 0.166784 0.132867

ENSMUST00000019577 1002 0.1955 0.4662 0.1624 0.4078 0.243 0.096 0.469 43.52 0.704 0.609 324 333 -0.303904 0.063063

ENSMUST00000018803 786 0.2500 0.4400 0.1885 0.3925 0.257 0.122 0.490 51.84 0.652 0.568 247 261 -0.648659 0.114943

ENSMUST00000021772 1305 0.2350 0.3754 0.2128 0.3920 0.195 0.108 0.460 52.11 0.625 0.535 413 434 -0.070968 0.069124

ENSMUST00000020962 777 0.4398 0.2513 0.3959 0.2270 0.219 -0.007 0.421 49.19 0.357 0.408 252 258 -0.603876 0.081395

ENSMUST00000015278 1539 0.2074 0.4074 0.2323 0.4297 0.245 0.060 0.456 47.97 0.648 0.548 500 512 -0.196485 0.085938

ENSMUST00000021203 585 0.3063 0.3688 0.2013 0.3333 0.244 0.036 0.455 50.60 0.572 0.565 187 194 0.092784 0.092784

ENSMUST00000021450 1293 0.1989 0.4462 0.1532 0.4082 0.242 0.215 0.523 50.63 0.696 0.567 411 430 0.375814 0.106977

ENSMUST00000019803 501 0.1770 0.3540 0.2177 0.5000 0.178 -0.088 0.360 39.83 0.683 0.610 164 166 -1.151.205 0.012048

ENSMUST00000014812 1992 0.3212 0.3308 0.2851 0.3347 0.247 0.084 0.468 52.84 0.518 0.508 643 663 -0.534389 0.058824

ENSMUST00000016144 855 0.3182 0.3802 0.2424 0.3073 0.205 0.026 0.434 58.22 0.540 0.469 272 284 0.097887 0.133803

ENSMUST00000020911 936 0.2724 0.4024 0.1977 0.3295 0.228 0.045 0.446 47.43 0.607 0.597 303 311 -0.459486 0.051447

ENSMUST00000019246 1362 0.2632 0.4321 0.1632 0.4230 0.256 0.069 0.457 50.16 0.655 0.536 435 453 -0.279470 0.075055

ENSMUST00000021890 1005 0.4086 0.2724 0.3843 0.2381 0.233 0.022 0.444 48.61 0.383 0.427 313 334 -0.520060 0.116766

ENSMUST00000018383 537 0.2879 0.4015 0.2745 0.2819 0.214 0.020 0.474 61.00 0.543 0.474 175 178 -1.258.989 0.022472

ENSMUST00000015987 1392 0.2322 0.4723 0.2006 0.3243 0.240 0.118 0.484 51.10 0.643 0.550 446 463 -0.285961 0.066955

ENSMUST00000021296 774 0.2074 0.4424 0.1082 0.4365 0.239 0.173 0.502 47.87 0.726 0.584 241 257 0.460700 0.136187

ENSMUST00000019506 285 0.1905 0.3651 0.2022 0.4588 0.239 0.094 0.478 50.58 0.674 0.582 92 94 -0.831915 0.010638

ENSMUST00000021278 3636 0.2877 0.3551 0.2270 0.3323 0.195 0.051 0.424 54.22 0.566 0.547 1178 1211 -0.061189 0.070190

ENSMUST00000014562 3381 0.3459 0.2948 0.2836 0.3170 0.207 0.011 0.414 55.41 0.486 0.490 1093 1126 -0.223091 0.074600

ENSMUST00000020271 459 0.3468 0.4194 0.2642 0.2245 0.217 0.079 0.476 60.22 0.510 0.498 145 152 -0.503947 0.125000

ENSMUST00000020949 1005 0.2902 0.3294 0.2767 0.3983 0.251 0.053 0.447 53.12 0.550 0.493 320 334 -0.266766 0.077844

ENSMUST00000020624 1659 0.3759 0.2483 0.3791 0.2754 0.190 -0.040 0.386 52.74 0.401 0.422 529 552 -0.272283 0.088768

ENSMUST00000021049 1221 0.2857 0.3389 0.2136 0.4426 0.239 0.054 0.438 53.51 0.601 0.512 388 406 -0.321675 0.041872

ENSMUST00000018274 1248 0.2743 0.4218 0.2058 0.3402 0.224 0.109 0.481 51.96 0.607 0.535 399 415 -0.581446 0.089157

ENSMUST00000020171 1047 0.1684 0.5754 0.1687 0.3462 0.292 0.195 0.539 43.04 0.734 0.599 334 348 -0.152586 0.063218

ENSMUST00000020553 501 0.3712 0.2879 0.4390 0.1709 0.242 0.017 0.429 52.81 0.360 0.436 161 166 -0.453614 0.144578

ENSMUST00000021968 948 0.0906 0.5827 0.1004 0.4407 0.270 0.261 0.579 36.52 0.843 0.653 299 315 -0.746667 0.082540

ENSMUST00000016034 1281 0.2294 0.4118 0.2055 0.4013 0.277 0.192 0.531 51.70 0.644 0.556 407 426 -0.025117 0.056338

ENSMUST00000017900 3252 0.2073 0.4647 0.1601 0.4019 0.282 0.142 0.496 47.29 0.695 0.560 1023 1083 0.103693 0.090489

ENSMUST00000021750 14901 0.2944 0.3760 0.2630 0.3583 0.239 0.062 0.453 53.50 0.560 0.494 4750 4966 -0.309666 0.091422

ENSMUST00000019833 342 0.2796 0.3548 0.1505 0.4130 0.208 0.071 0.450 61.00 0.640 0.611 111 113 -0.444248 0.079646

ENSMUST00000020657 459 0.3950 0.2353 0.3750 0.2692 0.195 -0.066 0.379 61.00 0.386 0.445 145 152 -0.649342 0.098684

ENSMUST00000021635 972 0.3874 0.3202 0.2556 0.3744 0.225 -0.054 0.385 52.73 0.503 0.445 312 323 -0.342105 0.095975

ENSMUST00000018842 1221 0.2455 0.4671 0.1892 0.3519 0.244 0.125 0.496 52.11 0.651 0.572 395 406 -0.646798 0.078818

ENSMUST00000017610 663 0.1734 0.5202 0.1563 0.4577 0.266 0.088 0.476 48.48 0.738 0.571 210 220 -0.161818 0.081818

ENSMUST00000021532 1170 0.3050 0.3262 0.2852 0.3776 0.236 0.079 0.472 58.24 0.539 0.489 371 389 -0.778149 0.074550

ENSMUST00000021689 1245 0.2400 0.3815 0.2286 0.3582 0.194 0.003 0.423 50.98 0.607 0.576 402 414 -0.784541 0.033816

ENSMUST00000020169 2625 0.3556 0.3152 0.3289 0.2822 0.199 -0.045 0.394 54.66 0.459 0.465 841 874 -0.351259 0.104119

ENSMUST00000015391 744 0.3618 0.2864 0.3115 0.2841 0.206 -0.027 0.403 56.29 0.453 0.487 236 247 -0.349393 0.133603

ENSMUST00000019069 1710 0.2353 0.3505 0.3066 0.3990 0.199 -0.001 0.406 51.81 0.573 0.482 539 569 -0.459754 0.082601

ENSMUST00000014957 744 0.2228 0.4752 0.2391 0.3353 0.247 0.150 0.512 48.30 0.632 0.526 242 247 -0.305263 0.056680

ENSMUST00000017290 5439 0.3825 0.2636 0.3532 0.2688 0.211 -0.023 0.417 52.97 0.414 0.467 1771 1812 -0.726766 0.039183

ENSMUST00000014922 3594 0.2284 0.3893 0.2060 0.3665 0.227 0.096 0.460 48.41 0.632 0.593 1173 1197 -0.308438 0.051796

ENSMUST00000020251 3708 0.2941 0.3730 0.2835 0.3461 0.233 0.073 0.460 55.90 0.547 0.485 1194 1235 -0.466964 0.093117

ENSMUST00000018632 5820 0.2302 0.4650 0.1748 0.4965 0.315 0.123 0.503 44.66 0.700 0.531 1873 1939 -0.798504 0.055183

ENSMUST00000021810 486 0.1135 0.4752 0.0602 0.5313 0.329 0.381 0.629 35.27 0.849 0.714 159 161 -0.061491 0.012422

ENSMUST00000015855 1365 0.2610 0.3929 0.2343 0.3792 0.254 0.058 0.444 51.56 0.601 0.527 444 454 -0.156167 0.055066

ENSMUST00000020500 1989 0.2763 0.3579 0.2907 0.3563 0.253 0.074 0.466 55.54 0.548 0.498 640 662 -0.324018 0.067976

ENSMUST00000021649 1362 0.1860 0.4663 0.1250 0.4344 0.248 0.198 0.523 43.22 0.739 0.622 436 453 -0.122958 0.088300

ENSMUST00000017974 2037 0.2197 0.3969 0.1969 0.4633 0.235 0.084 0.460 49.57 0.666 0.551 650 678 -0.276401 0.063422

ENSMUST00000020215 597 0.2839 0.2774 0.3182 0.3767 0.204 0.074 0.461 58.14 0.513 0.498 191 198 -0.495960 0.090909

ENSMUST00000015894 744 0.2844 0.3733 0.1947 0.3064 0.233 0.114 0.466 52.06 0.576 0.534 238 247 0.899190 0.145749

ENSMUST00000020683 846 0.2857 0.3871 0.2877 0.3299 0.222 0.034 0.424 43.35 0.546 0.453 271 281 -0.062278 0.053381

ENSMUST00000018716 1206 0.3462 0.3045 0.2477 0.3230 0.179 -0.067 0.378 56.19 0.512 0.557 389 401 -0.932419 0.044888

ENSMUST00000015596 1209 0.2638 0.3466 0.2238 0.3383 0.159 -0.049 0.370 49.90 0.579 0.598 392 402 -0.308458 0.039801

ENSMUST00000018002 1281 0.3404 0.3495 0.2658 0.3525 0.251 0.034 0.436 55.82 0.528 0.476 415 426 -0.288967 0.096244

ENSMUST00000016105 1371 0.3945 0.2438 0.3553 0.2679 0.202 0.005 0.422 55.21 0.395 0.450 443 456 -0.212938 0.083333

ENSMUST00000020719 783 0.1905 0.4815 0.2057 0.4087 0.273 0.193 0.545 45.17 0.690 0.591 255 260 -1.491.154 0.042308

ENSMUST00000015877 861 0.4346 0.2103 0.4115 0.2842 0.239 -0.083 0.385 55.58 0.356 0.406 278 286 -0.586364 0.101399

ENSMUST00000015501 1905 0.3876 0.2149 0.3594 0.2946 0.199 -0.060 0.385 52.95 0.398 0.462 626 634 -0.411356 0.052050

ENSMUST00000019130 1554 0.3291 0.3013 0.3520 0.3149 0.213 -0.036 0.395 54.99 0.465 0.437 501 517 -0.130948 0.096712

ENSMUST00000020284 1707 0.1389 0.4365 0.1074 0.4549 0.246 0.210 0.523 41.94 0.781 0.692 553 568 0.181514 0.058099

ENSMUST00000021085 1971 0.2935 0.3718 0.2190 0.3872 0.237 0.063 0.444 52.34 0.588 0.518 621 656 -0.117683 0.082317

ENSMUST00000019616 2754 0.1980 0.3959 0.1932 0.3753 0.217 0.100 0.467 51.01 0.661 0.635 892 917 -0.199891 0.051254

ENSMUST00000020926 879 0.1174 0.5783 0.0987 0.4883 0.311 0.251 0.563 39.04 0.829 0.645 286 292 -0.506507 0.085616

ENSMUST00000015901 1479 0.3803 0.2563 0.4500 0.2840 0.217 -0.024 0.421 53.79 0.384 0.409 482 492 -1.025.813 0.085366

ENSMUST00000016315 3507 0.2686 0.4148 0.2122 0.3625 0.220 0.040 0.448 49.37 0.614 0.575 1125 1168 -0.518493 0.052226

ENSMUST00000018407 1557 0.2518 0.4866 0.1641 0.3531 0.261 0.145 0.510 48.21 0.665 0.562 496 518 -0.694788 0.086873

ENSMUST00000021339 507 0.2117 0.3942 0.1742 0.4603 0.264 0.167 0.512 55.38 0.683 0.573 164 168 -0.135714 0.059524

ENSMUST00000017831 1992 0.3910 0.2632 0.3416 0.2928 0.208 -0.020 0.403 55.61 0.420 0.438 643 663 -0.308597 0.093514

ENSMUST00000021183 1314 0.2686 0.3800 0.1833 0.3900 0.207 0.055 0.427 54.26 0.624 0.548 426 437 -0.117163 0.045767

ENSMUST00000017408 2664 0.2541 0.3916 0.2478 0.4015 0.239 0.068 0.458 52.42 0.607 0.532 861 887 -0.647689 0.080045

ENSMUST00000020522 2343 0.2299 0.4394 0.1321 0.4358 0.279 0.115 0.486 43.74 0.698 0.576 745 780 -0.083333 0.073077

ENSMUST00000020579 2385 0.3940 0.2160 0.3976 0.3344 0.246 0.039 0.452 52.02 0.411 0.418 779 794 -0.986020 0.036524

ENSMUST00000021120 1929 0.1667 0.5000 0.1296 0.4130 0.235 0.155 0.500 44.34 0.753 0.654 628 642 -0.330841 0.062305

ENSMUST00000021791 1515 0.2857 0.3971 0.2507 0.3201 0.219 0.009 0.427 50.10 0.569 0.540 487 504 -0.612897 0.097222

ENSMUST00000020939 3771 0.3416 0.3218 0.3141 0.2758 0.217 0.000 0.418 57.32 0.468 0.480 1215 1256 -0.403822 0.079618

ENSMUST00000014597 1500 0.2228 0.4404 0.1532 0.4472 0.238 0.090 0.462 46.12 0.695 0.554 476 499 -0.296393 0.098196

ENSMUST00000020003 3432 0.3022 0.3184 0.3035 0.4128 0.228 0.024 0.438 57.68 0.544 0.482 1106 1143 -0.931671 0.035871

ENSMUST00000021406 906 0.3636 0.2893 0.3151 0.3168 0.197 -0.069 0.368 59.35 0.460 0.455 291 301 -0.011296 0.086379

ENSMUST00000017597 1173 0.2790 0.3793 0.2491 0.3585 0.205 0.005 0.414 56.53 0.573 0.517 377 390 -0.177436 0.089744

ENSMUST00000020081 759 0.3312 0.2930 0.2066 0.4952 0.294 0.096 0.482 49.96 0.608 0.520 245 252 -0.804365 0.035714

ENSMUST00000019445 1035 0.2396 0.3750 0.1855 0.4023 0.243 0.094 0.466 55.19 0.642 0.588 335 344 0.056686 0.061047

ENSMUST00000019679 1407 0.2727 0.4171 0.1573 0.3983 0.266 0.101 0.469 47.78 0.648 0.584 458 468 0.012393 0.029915

ENSMUST00000018971 1962 0.2015 0.4203 0.1869 0.4511 0.234 0.075 0.451 45.71 0.683 0.565 638 653 -0.491118 0.061256

ENSMUST00000021729 1149 0.1860 0.4909 0.1831 0.3688 0.284 0.203 0.526 47.31 0.695 0.542 371 382 0.363351 0.107330

ENSMUST00000017692 2226 0.3925 0.2294 0.4135 0.2561 0.195 -0.043 0.408 52.87 0.370 0.437 716 741 -0.732254 0.070175

ENSMUST00000017534 1092 0.3131 0.4007 0.1707 0.3521 0.244 0.023 0.431 51.55 0.600 0.560 355 363 -0.232507 0.066116

ENSMUST00000019945 1119 0.3507 0.2674 0.3472 0.2992 0.196 0.031 0.426 54.32 0.437 0.436 357 372 -0.152419 0.067204

ENSMUST00000021133 1878 0.2102 0.4437 0.2141 0.4200 0.244 0.114 0.488 50.49 0.664 0.545 611 625 -0.575200 0.075200

ENSMUST00000016902 453 0.2114 0.4146 0.1345 0.4196 0.176 0.117 0.450 43.93 0.700 0.587 140 150 0.061333 0.060000

ENSMUST00000020954 2394 0.2672 0.3984 0.2320 0.3524 0.248 0.092 0.475 51.76 0.597 0.532 767 797 -0.475157 0.069009

ENSMUST00000021195 1893 0.2268 0.4726 0.1703 0.3672 0.260 0.130 0.489 50.97 0.667 0.529 597 630 0.461746 0.139683

ENSMUST00000019967 3147 0.2350 0.3875 0.2110 0.4150 0.226 0.033 0.432 50.61 0.639 0.574 1012 1048 -0.422710 0.066794

ENSMUST00000016631 2910 0.3273 0.2790 0.2833 0.3786 0.227 0.027 0.435 54.10 0.514 0.496 930 969 -0.647368 0.052632

ENSMUST00000021778 669 0.3600 0.3486 0.3624 0.2273 0.209 0.040 0.438 59.15 0.438 0.410 208 222 -0.175225 0.085586

ENSMUST00000021288 3399 0.2283 0.4165 0.1824 0.3650 0.205 0.112 0.475 51.92 0.652 0.591 1099 1132 -0.492668 0.067138

ENSMUST00000020043 2445 0.3769 0.2192 0.4642 0.2848 0.223 -0.034 0.413 50.82 0.367 0.411 791 814 -1.106.265 0.040541

ENSMUST00000015368 1515 0.3441 0.3218 0.3123 0.3082 0.217 0.021 0.423 53.98 0.478 0.446 485 504 -0.111310 0.109127

ENSMUST00000020249 717 0.2609 0.4348 0.2059 0.3200 0.216 0.032 0.432 52.25 0.608 0.499 227 238 0.777311 0.142857

ENSMUST00000021399 930 0.3882 0.2194 0.4420 0.2488 0.216 -0.079 0.378 49.67 0.355 0.420 296 309 -0.471197 0.077670

ENSMUST00000021066 984 0.1678 0.5524 0.1213 0.3704 0.238 0.203 0.537 44.46 0.756 0.580 315 327 -0.183180 0.103976

ENSMUST00000021884 342 0.3750 0.2375 0.4286 0.2895 0.191 -0.155 0.356 44.29 0.394 0.448 104 113 -1.123.894 0.106195

ENSMUST00000020904 4167 0.3964 0.2264 0.4083 0.3149 0.220 -0.018 0.415 54.35 0.395 0.417 1334 1388 -0.843588 0.056916

ENSMUST00000020695 4323 0.2812 0.4000 0.2106 0.3318 0.235 0.059 0.452 51.35 0.593 0.551 1400 1440 -0.434722 0.063194

ENSMUST00000020712 507 0.2381 0.4444 0.1667 0.4215 0.227 0.162 0.506 51.09 0.677 0.520 158 168 -0.866667 0.071429

ENSMUST00000020643 2139 0.2202 0.3786 0.2461 0.4510 0.238 0.066 0.459 54.91 0.636 0.544 687 712 -0.660253 0.033708

ENSMUST00000020529 996 0.2926 0.3013 0.3410 0.3648 0.249 0.065 0.462 53.51 0.503 0.472 314 331 -0.484894 0.078550

ENSMUST00000017255 1539 0.2725 0.3925 0.2302 0.3497 0.256 0.143 0.522 51.29 0.591 0.548 494 512 -0.420313 0.058594

ENSMUST00000019064 741 0.3232 0.3283 0.2629 0.3122 0.250 0.099 0.481 60.59 0.519 0.539 239 246 -0.423984 0.081301

ENSMUST00000017354 2964 0.1927 0.4922 0.1415 0.4284 0.264 0.141 0.491 45.23 0.727 0.562 935 987 0.026140 0.066869

ENSMUST00000020349 6825 0.2287 0.3977 0.1756 0.3685 0.228 0.100 0.470 46.39 0.652 0.623 2220 2274 -0.531310 0.029903

ENSMUST00000018476 1494 0.3736 0.2299 0.4048 0.3129 0.204 -0.077 0.385 53.34 0.400 0.432 468 497 -0.656137 0.068410

ENSMUST00000014546 1176 0.3799 0.2760 0.3400 0.2572 0.223 0.012 0.416 56.38 0.416 0.457 375 391 -0.437340 0.084399

ENSMUST00000019965 1260 0.2536 0.4140 0.1993 0.4078 0.253 0.128 0.486 50.85 0.638 0.543 403 419 -0.066110 0.105012

ENSMUST00000020109 1191 0.3987 0.3023 0.3759 0.2573 0.223 -0.013 0.415 57.21 0.407 0.405 383 396 -0.287879 0.121212

ENSMUST00000021932 1341 0.2876 0.4327 0.1692 0.3391 0.245 0.049 0.444 49.95 0.615 0.502 426 446 0.281166 0.109865

ENSMUST00000018516 2484 0.3458 0.2695 0.3837 0.3023 0.206 0.026 0.442 56.61 0.434 0.464 800 827 -1.033.011 0.061669

ENSMUST00000020204 1887 0.2085 0.4749 0.2806 0.3308 0.246 0.110 0.494 51.67 0.627 0.544 603 628 -0.416401 0.090764

ENSMUST00000016670 1761 0.2430 0.4215 0.2115 0.3910 0.249 0.168 0.514 53.36 0.633 0.535 570 586 -0.438055 0.081911

ENSMUST00000018399 1215 0.2013 0.4968 0.0915 0.5326 0.296 0.188 0.525 37.23 0.774 0.584 398 404 -0.568317 0.044554

ENSMUST00000018905 744 0.2049 0.4488 0.1960 0.3403 0.235 0.117 0.462 51.72 0.660 0.557 238 247 0.716599 0.089069

ENSMUST00000015816 564 0.3406 0.2609 0.3673 0.3015 0.181 -0.013 0.399 61.00 0.433 0.472 178 187 -0.667380 0.074866

ENSMUST00000019625 5814 0.2170 0.4526 0.1737 0.5168 0.302 0.092 0.484 44.75 0.709 0.535 1885 1937 -0.788797 0.055240

ENSMUST00000021665 1086 0.1455 0.4478 0.1921 0.4403 0.207 0.070 0.462 49.41 0.720 0.608 346 361 -0.687812 0.044321

ENSMUST00000020826 549 0.1338 0.5704 0.1691 0.4453 0.243 0.161 0.522 41.45 0.767 0.571 180 182 -0.905494 0.076923

ENSMUST00000020301 930 0.1699 0.5019 0.2075 0.3362 0.232 0.164 0.508 49.29 0.688 0.591 301 309 -0.295793 0.064725

ENSMUST00000021870 1242 0.2639 0.3578 0.2871 0.3287 0.241 0.093 0.466 48.18 0.547 0.473 395 413 0.415739 0.123487

ENSMUST00000019997 2328 0.3091 0.4033 0.2756 0.2987 0.224 0.076 0.469 54.55 0.541 0.508 748 775 -0.576258 0.076129

ENSMUST00000021384 1554 0.3867 0.2640 0.4340 0.2308 0.231 -0.010 0.424 52.57 0.371 0.444 502 517 -0.701354 0.088975

ENSMUST00000021268 2136 0.2245 0.4575 0.1744 0.4004 0.259 0.104 0.471 48.89 0.675 0.556 684 711 -0.148383 0.105485

ENSMUST00000014642 3231 0.2830 0.3880 0.1802 0.3615 0.262 0.106 0.477 49.50 0.611 0.569 1054 1076 -0.045074 0.048327

ENSMUST00000019791 3096 0.2649 0.4271 0.2142 0.3375 0.235 0.073 0.462 50.64 0.609 0.529 998 1031 -0.437051 0.075655

ENSMUST00000025853 618 0.2338 0.3896 0.2073 0.4522 0.235 -0.001 0.428 45.35 0.652 0.572 201 205 -0.955122 0.043902

ENSMUST00000027579 1257 0.3754 0.2553 0.3706 0.2776 0.208 -0.080 0.371 57.50 0.403 0.451 404 418 -0.266986 0.105263

ENSMUST00000027337 4521 0.4296 0.2305 0.3922 0.2143 0.218 -0.025 0.407 51.46 0.345 0.416 1472 1506 -0.317198 0.072377

ENSMUST00000026474 3336 0.2773 0.3983 0.2037 0.3145 0.199 0.032 0.437 51.16 0.596 0.591 1081 1111 -0.705311 0.054905

ENSMUST00000026973 1146 0.2416 0.4430 0.1831 0.4176 0.238 0.072 0.457 49.56 0.661 0.549 372 381 -0.463517 0.078740

ENSMUST00000029268 390 0.3107 0.3592 0.3368 0.2366 0.177 0.008 0.415 50.05 0.480 0.512 123 129 -0.770543 0.069767

ENSMUST00000029208 1803 0.1542 0.5233 0.1293 0.3920 0.250 0.159 0.492 44.11 0.758 0.588 561 600 0.529833 0.111667

ENSMUST00000027507 867 0.2338 0.4199 0.2377 0.3113 0.181 -0.045 0.385 51.72 0.604 0.569 270 288 -0.240278 0.090278

ENSMUST00000024839 2340 0.1919 0.4758 0.1613 0.3869 0.219 0.154 0.497 46.70 0.705 0.596 760 779 -0.248524 0.060334

ENSMUST00000028118 2124 0.2514 0.4171 0.3210 0.3452 0.234 0.051 0.454 52.42 0.566 0.484 670 707 -0.398869 0.104668

ENSMUST00000023958 2199 0.2038 0.3869 0.1706 0.4508 0.206 0.095 0.461 50.32 0.690 0.636 710 732 -0.521721 0.066940

ENSMUST00000025786 2886 0.2424 0.4318 0.2053 0.3453 0.251 0.116 0.484 50.54 0.629 0.548 931 961 -0.467326 0.055151

ENSMUST00000025236 675 0.4034 0.2955 0.2822 0.2763 0.258 0.083 0.464 61.00 0.445 0.473 211 224 -0.371875 0.102679

ENSMUST00000028593 681 0.3386 0.2857 0.3943 0.2061 0.195 -0.070 0.367 49.72 0.398 0.456 221 226 -0.224779 0.101770

ENSMUST00000027690 1266 0.2458 0.4721 0.1098 0.3409 0.274 0.235 0.540 44.94 0.688 0.579 398 421 0.232541 0.090261

ENSMUST00000025506 1263 0.2716 0.3940 0.3077 0.2891 0.213 0.051 0.448 52.34 0.537 0.525 404 420 -0.674286 0.080952

ENSMUST00000026550 1269 0.2091 0.4939 0.2226 0.3978 0.266 0.114 0.493 52.82 0.672 0.515 408 422 -0.469668 0.125592

ENSMUST00000025705 3399 0.3637 0.2851 0.3649 0.3046 0.200 -0.012 0.413 54.53 0.436 0.431 1092 1132 -0.412456 0.103357

ENSMUST00000025636 873 0.2688 0.3913 0.2643 0.2562 0.233 0.031 0.419 46.37 0.541 0.515 279 290 0.320690 0.089655

ENSMUST00000026520 1203 0.4539 0.1776 0.4192 0.2662 0.234 -0.033 0.407 50.30 0.330 0.421 388 400 -0.669000 0.097500

ENSMUST00000022051 1386 0.2527 0.4231 0.2213 0.3668 0.205 0.017 0.433 53.96 0.623 0.585 453 461 -1.106.508 0.047722

ENSMUST00000024783 1311 0.2147 0.4202 0.1695 0.4521 0.231 0.056 0.438 47.84 0.689 0.585 418 436 -0.405505 0.068807

ENSMUST00000024742 1095 0.2458 0.3654 0.1993 0.4199 0.258 0.148 0.492 53.42 0.633 0.595 360 364 -0.283791 0.030220

ENSMUST00000024721 1317 0.3995 0.2962 0.3087 0.2171 0.233 0.014 0.429 55.60 0.412 0.452 413 438 0.389498 0.121005

ENSMUST00000024826 390 0.2844 0.4220 0.2184 0.3452 0.260 0.099 0.472 52.71 0.600 0.558 125 129 -0.077519 0.093023

ENSMUST00000026577 2190 0.1734 0.4557 0.1966 0.4480 0.261 0.129 0.487 44.90 0.704 0.578 706 729 -0.628532 0.063100

ENSMUST00000029877 1008 0.3764 0.2583 0.3244 0.2731 0.222 0.017 0.435 53.60 0.419 0.470 322 335 -0.056418 0.086567

ENSMUST00000028584 597 0.2102 0.4204 0.2166 0.3946 0.314 0.283 0.576 53.72 0.649 0.545 191 198 -0.017677 0.040404

ENSMUST00000025192 753 0.1810 0.5143 0.1543 0.3678 0.258 0.196 0.515 46.49 0.720 0.575 239 250 0.055200 0.096000

ENSMUST00000028360 471 0.2500 0.4194 0.2288 0.3514 0.252 0.109 0.477 51.48 0.611 0.545 149 156 -0.003846 0.089744

ENSMUST00000027286 225 0.3036 0.2321 0.3273 0.4364 0.207 -0.051 0.403 36.91 0.514 0.514 72 74 -0.710811 0.094595

ENSMUST00000025993 4596 0.2070 0.5078 0.1328 0.4183 0.273 0.172 0.519 44.28 0.725 0.587 1500 1531 -0.268648 0.063357

ENSMUST00000028105 1335 0.3815 0.2630 0.3563 0.2635 0.201 -0.060 0.381 57.86 0.409 0.448 425 444 -0.132432 0.101351

ENSMUST00000028898 396 0.1810 0.3879 0.2315 0.3301 0.198 0.205 0.504 50.71 0.632 0.603 125 131 0.769465 0.076336

ENSMUST00000025864 501 0.1594 0.4130 0.1591 0.4844 0.260 0.171 0.512 48.70 0.735 0.657 162 166 -0.332530 0.066265

ENSMUST00000025058 3570 0.1897 0.4623 0.1571 0.4267 0.259 0.149 0.504 46.32 0.715 0.595 1162 1189 -0.538604 0.047098

ENSMUST00000025749 1458 0.2312 0.4422 0.2214 0.3352 0.215 0.035 0.430 49.17 0.627 0.565 474 485 -0.359381 0.080412

ENSMUST00000022646 714 0.2105 0.4211 0.1809 0.4262 0.215 0.081 0.461 52.76 0.681 0.592 232 237 -0.921941 0.080169

ENSMUST00000022834 783 0.2619 0.3762 0.2778 0.3297 0.178 -0.029 0.392 53.75 0.560 0.508 250 260 -0.224615 0.069231

ENSMUST00000025053 1644 0.1995 0.5134 0.1244 0.4403 0.248 0.054 0.458 41.54 0.746 0.591 535 547 -1.021.389 0.053016

ENSMUST00000027826 453 0.1600 0.4400 0.1695 0.4554 0.208 0.117 0.473 41.89 0.726 0.631 146 150 -0.189333 0.080000

ENSMUST00000025617 468 0.3967 0.3802 0.2613 0.2600 0.300 0.091 0.477 54.43 0.483 0.452 149 155 -0.307097 0.109677

ENSMUST00000022163 615 0.2930 0.3121 0.2874 0.3438 0.228 0.046 0.455 49.92 0.525 0.529 198 204 -0.703431 0.029412

ENSMUST00000028137 801 0.1972 0.4404 0.1690 0.3911 0.254 0.212 0.516 49.65 0.689 0.604 254 266 -0.153759 0.063910

ENSMUST00000027237 1845 0.3486 0.3111 0.3015 0.3086 0.191 -0.091 0.357 52.55 0.480 0.464 588 614 -0.224919 0.100977

ENSMUST00000023083 1503 0.2279 0.4240 0.1806 0.4011 0.258 0.155 0.493 48.01 0.663 0.575 475 500 -0.053800 0.098000

ENSMUST00000022587 432 0.2500 0.2885 0.2750 0.4261 0.274 0.138 0.500 52.11 0.572 0.524 138 143 -0.389511 0.041958

ENSMUST00000027682 1446 0.2095 0.4688 0.1227 0.4028 0.234 0.111 0.466 44.34 0.718 0.589 461 481 0.248649 0.087318

ENSMUST00000027921 3039 0.3199 0.3338 0.2487 0.3430 0.220 0.001 0.417 55.99 0.535 0.511 963 1012 -0.224407 0.089921

ENSMUST00000027429 861 0.1387 0.3866 0.1452 0.4878 0.224 0.182 0.516 43.95 0.754 0.704 281 286 -0.428671 0.041958

ENSMUST00000029521 309 0.1957 0.6630 0.1447 0.1447 0.315 0.439 0.703 35.34 0.713 0.676 101 102 -0.535294 0.019608

ENSMUST00000026318 516 0.3333 0.3810 0.3504 0.2710 0.281 0.103 0.488 61.00 0.481 0.448 160 171 -0.296491 0.146199

ENSMUST00000023994 1515 0.2488 0.4458 0.2338 0.3027 0.274 0.139 0.496 49.59 0.605 0.526 484 504 -0.166270 0.085317

ENSMUST00000023805 1482 0.2302 0.4399 0.1079 0.4737 0.232 0.064 0.445 43.37 0.724 0.590 474 493 -0.126775 0.083164

ENSMUST00000026710 2478 0.4027 0.2529 0.3714 0.3013 0.229 -0.014 0.419 55.52 0.412 0.437 804 825 -0.748243 0.055758

ENSMUST00000029850 996 0.3197 0.3569 0.2622 0.2927 0.231 0.081 0.466 55.87 0.519 0.515 324 331 -0.009366 0.063444

ENSMUST00000025477 1143 0.3409 0.3312 0.2992 0.3184 0.197 -0.017 0.409 59.04 0.495 0.447 364 380 -0.205790 0.131579

ENSMUST00000028689 5718 0.2631 0.4556 0.1814 0.3498 0.256 0.120 0.494 48.93 0.637 0.562 1821 1905 -0.485669 0.072966

ENSMUST00000027554 867 0.1983 0.5148 0.1134 0.3978 0.273 0.189 0.525 47.77 0.740 0.567 265 288 0.314583 0.163194

ENSMUST00000028355 1374 0.2437 0.4548 0.1801 0.3090 0.210 0.044 0.445 48.75 0.639 0.578 449 457 -0.470460 0.070022

ENSMUST00000027269 1131 0.3311 0.3174 0.3860 0.2602 0.204 -0.020 0.407 54.41 0.437 0.452 359 376 -0.400798 0.093085

ENSMUST00000025511 456 0.3306 0.3636 0.1746 0.3475 0.209 0.007 0.429 53.07 0.578 0.565 147 151 -0.502649 0.033113

ENSMUST00000028972 402 0.2198 0.3736 0.2358 0.4851 0.231 0.102 0.461 51.94 0.648 0.516 128 133 -0.565414 0.037594

ENSMUST00000023265 372 0.1759 0.4907 0.1412 0.4444 0.333 0.260 0.567 45.11 0.742 0.556 120 123 0.185366 0.073171

ENSMUST00000028300 1761 0.2381 0.4555 0.1535 0.3616 0.273 0.102 0.475 44.74 0.671 0.593 569 586 -0.235836 0.064846

ENSMUST00000025064 3717 0.3659 0.2911 0.3726 0.2500 0.209 -0.018 0.417 53.23 0.417 0.452 1205 1238 -0.698061 0.062197

ENSMUST00000029567 1554 0.3012 0.3529 0.2266 0.3501 0.203 0.088 0.456 53.31 0.562 0.524 502 517 -0.413153 0.071567

ENSMUST00000022344 1926 0.2994 0.3293 0.3077 0.3451 0.221 0.012 0.425 56.08 0.519 0.507 619 641 -0.444150 0.082683

ENSMUST00000028408 1251 0.4227 0.2366 0.3411 0.3345 0.233 -0.022 0.408 51.38 0.416 0.421 404 416 -0.442789 0.134615

ENSMUST00000025729 3501 0.4004 0.2417 0.3596 0.2536 0.226 -0.013 0.413 53.37 0.388 0.466 1146 1166 -0.321527 0.055746

ENSMUST00000029386 1851 0.3910 0.2383 0.3693 0.2651 0.193 -0.071 0.377 55.23 0.389 0.466 594 616 -0.311201 0.087662

ENSMUST00000028209 717 0.1716 0.4853 0.1921 0.3476 0.193 0.109 0.462 51.73 0.693 0.555 225 238 0.365966 0.147059

ENSMUST00000029311 2925 0.2640 0.3732 0.2832 0.3649 0.209 0.044 0.437 54.53 0.564 0.481 940 974 -0.189220 0.099589

ENSMUST00000029508 1401 0.3066 0.3867 0.2575 0.3304 0.220 0.033 0.431 56.69 0.551 0.499 457 466 -0.230043 0.098712

ENSMUST00000028597 1530 0.2853 0.3676 0.2360 0.3669 0.241 0.066 0.446 53.14 0.578 0.518 493 509 -0.056385 0.062868

ENSMUST00000023147 3237 0.2150 0.4194 0.2037 0.3948 0.231 0.092 0.459 47.22 0.657 0.579 1052 1078 -0.251484 0.062152

ENSMUST00000029422 996 0.1818 0.4053 0.2169 0.4067 0.215 0.117 0.495 48.79 0.669 0.626 323 331 -0.516314 0.048338

ENSMUST00000023219 1608 0.2316 0.4232 0.1276 0.3915 0.237 0.232 0.530 49.18 0.691 0.614 515 535 -0.148037 0.056075

ENSMUST00000028761 1620 0.2948 0.3900 0.2191 0.3081 0.231 0.078 0.458 51.04 0.571 0.537 522 539 -0.231911 0.053803

ENSMUST00000025718 960 0.2435 0.3435 0.3347 0.3750 0.236 0.052 0.447 56.65 0.547 0.493 309 319 -0.505643 0.050157

ENSMUST00000022856 843 0.3867 0.3022 0.2985 0.3155 0.226 -0.002 0.420 55.17 0.464 0.443 274 280 -0.120714 0.096429

ENSMUST00000025406 1326 0.3272 0.2840 0.3159 0.3418 0.230 -0.013 0.426 56.13 0.491 0.503 434 441 -1.071.882 0.043084

ENSMUST00000029123 396 0.1273 0.5727 0.1633 0.3646 0.260 0.168 0.516 43.44 0.766 0.593 128 131 -0.274046 0.045802

ENSMUST00000022616 1347 0.1945 0.5106 0.1651 0.4569 0.300 0.183 0.527 46.99 0.725 0.545 429 448 -0.553571 0.082589

ENSMUST00000023814 345 0.2558 0.3488 0.2273 0.3953 0.234 0.154 0.500 42.36 0.604 0.582 106 114 -0.520175 0.122807

ENSMUST00000025713 1257 0.2366 0.3944 0.1313 0.4230 0.233 0.171 0.489 51.33 0.681 0.580 395 418 0.484450 0.131579

ENSMUST00000029911 2445 0.3664 0.2534 0.3707 0.2764 0.181 -0.041 0.413 55.34 0.415 0.487 785 814 -1.635.749 0.045455

ENSMUST00000022115 981 0.3808 0.2552 0.3594 0.2992 0.216 -0.008 0.420 55.79 0.423 0.447 317 326 -0.744479 0.064417

ENSMUST00000029419 2502 0.2792 0.3754 0.3132 0.3254 0.244 0.087 0.468 52.62 0.532 0.474 809 833 -0.356423 0.069628

ENSMUST00000026560 1131 0.3134 0.3239 0.2070 0.4440 0.263 0.083 0.457 50.61 0.588 0.507 359 376 -0.139362 0.077128

ENSMUST00000022629 1719 0.2597 0.4485 0.1532 0.3846 0.278 0.141 0.505 47.78 0.658 0.557 553 572 -0.266783 0.068182

ENSMUST00000025641 1872 0.2795 0.4090 0.2064 0.3206 0.231 0.079 0.456 51.82 0.595 0.556 607 623 -0.082504 0.096308

ENSMUST00000022450 4497 0.4630 0.1836 0.3889 0.2619 0.211 -0.070 0.381 50.22 0.335 0.403 1466 1498 -0.489786 0.080107

ENSMUST00000024884 1548 0.3686 0.2835 0.4130 0.2528 0.211 -0.035 0.416 54.38 0.400 0.414 503 515 -0.656116 0.089320

ENSMUST00000027533 1746 0.2229 0.4480 0.1494 0.4141 0.288 0.156 0.503 45.76 0.695 0.601 557 581 -0.214458 0.094664

ENSMUST00000024761 2106 0.1768 0.4870 0.1550 0.4103 0.227 0.143 0.496 47.86 0.725 0.615 684 701 -0.197718 0.051355

ENSMUST00000025229 2292 0.2753 0.4179 0.2277 0.3550 0.210 -0.016 0.411 51.82 0.599 0.529 739 763 -0.462516 0.082569

ENSMUST00000029643 696 0.2800 0.3550 0.3681 0.1899 0.158 0.048 0.469 51.91 0.461 0.567 228 231 -0.825541 0.108225

ENSMUST00000024049 1179 0.3910 0.3333 0.2465 0.2704 0.188 -0.060 0.370 56.27 0.478 0.487 370 392 -0.361480 0.084184

ENSMUST00000022980 540 0.3145 0.3145 0.2080 0.4959 0.236 0.087 0.470 51.91 0.604 0.534 164 179 -1.047.486 0.139665

ENSMUST00000025762 270 0.2388 0.3881 0.2344 0.4444 0.212 -0.041 0.400 47.76 0.635 0.551 85 89 -0.377528 0.101124

ENSMUST00000029625 888 0.0893 0.6295 0.1038 0.5051 0.360 0.337 0.610 35.17 0.851 0.594 282 295 -0.274576 0.064407

ENSMUST00000022916 2577 0.3201 0.3365 0.3010 0.2909 0.201 -0.004 0.424 58.18 0.497 0.515 835 858 -0.450932 0.081585

ENSMUST00000025377 1341 0.2750 0.4278 0.2704 0.3394 0.254 0.132 0.502 55.10 0.577 0.475 430 446 -0.569731 0.107623

ENSMUST00000022573 849 0.3947 0.3377 0.3096 0.2431 0.252 0.039 0.456 52.47 0.445 0.462 272 282 -0.254255 0.124113

ENSMUST00000022696 3264 0.2977 0.3580 0.2701 0.3437 0.228 0.062 0.446 54.13 0.544 0.476 1046 1087 -0.086660 0.090156

ENSMUST00000027440 1218 0.1971 0.4435 0.1433 0.4247 0.239 0.129 0.470 46.21 0.712 0.573 389 405 0.255803 0.091358

ENSMUST00000027602 1506 0.4289 0.2377 0.3351 0.2943 0.229 0.044 0.443 50.67 0.400 0.436 488 501 -0.403194 0.081836

ENSMUST00000028987 1425 0.1943 0.4948 0.1667 0.3724 0.249 0.136 0.477 44.55 0.699 0.542 455 474 0.168987 0.059072

ENSMUST00000024956 618 0.2317 0.3659 0.3487 0.3380 0.195 -0.033 0.392 58.93 0.543 0.488 199 205 0.048781 0.082927

ENSMUST00000027434 2454 0.2319 0.4101 0.2219 0.4047 0.235 0.087 0.463 51.77 0.636 0.535 789 817 -0.411383 0.069767

ENSMUST00000023617 1614 0.2373 0.4378 0.1941 0.3520 0.221 0.148 0.492 50.83 0.643 0.585 510 537 -0.632030 0.070764

ENSMUST00000029909 1113 0.3636 0.2626 0.2724 0.3285 0.202 0.002 0.419 56.18 0.475 0.498 356 370 -0.289730 0.083784

ENSMUST00000026659 2433 0.2039 0.4539 0.1258 0.4164 0.234 0.179 0.504 46.08 0.720 0.593 776 810 0.080988 0.096296

ENSMUST00000022528 999 0.2939 0.3465 0.3552 0.3333 0.254 -0.004 0.444 53.90 0.506 0.478 322 332 -1.259.036 0.039157

ENSMUST00000026128 255 0.2759 0.5172 0.1304 0.5116 0.343 0.136 0.514 39.45 0.703 0.548 74 84 -0.241667 0.107143

ENSMUST00000023670 1791 0.2376 0.3643 0.2256 0.4449 0.213 -0.043 0.414 56.85 0.632 0.594 587 596 -0.845134 0.040268

ENSMUST00000027064 438 0.3438 0.2422 0.2821 0.3009 0.147 -0.065 0.359 51.91 0.458 0.503 142 145 0.065517 0.089655

ENSMUST00000026297 1734 0.3443 0.3396 0.2349 0.3732 0.253 0.040 0.439 54.30 0.547 0.498 556 577 -0.573137 0.050260

ENSMUST00000028475 1278 0.3634 0.3169 0.2141 0.3471 0.256 0.032 0.429 49.87 0.528 0.504 413 425 -0.222118 0.087059

ENSMUST00000029046 408 0.2632 0.4000 0.2222 0.4362 0.243 -0.093 0.389 50.42 0.627 0.511 126 135 -0.447407 0.059259

ENSMUST00000022767 1743 0.3589 0.3129 0.2584 0.3278 0.229 0.018 0.428 57.19 0.501 0.505 561 580 -0.503793 0.055172

ENSMUST00000022518 675 0.3103 0.3621 0.2422 0.4094 0.256 0.097 0.484 59.91 0.571 0.510 217 224 -0.441964 0.111607

ENSMUST00000025497 8724 0.2745 0.4767 0.2299 0.3263 0.278 0.118 0.507 50.91 0.611 0.543 2836 2907 -0.371655 0.068111

ENSMUST00000022986 1068 0.1875 0.4632 0.2162 0.4440 0.261 0.179 0.518 49.78 0.685 0.510 340 355 -0.395775 0.104225

ENSMUST00000028252 1617 0.2894 0.3459 0.2857 0.3482 0.227 0.073 0.462 53.87 0.542 0.508 517 538 -0.481970 0.085502

ENSMUST00000025739 2412 0.4176 0.2606 0.3243 0.2740 0.228 0.015 0.433 53.62 0.411 0.465 778 803 -0.661519 0.078456

ENSMUST00000029451 666 0.3081 0.3081 0.3043 0.3333 0.223 -0.022 0.408 53.91 0.498 0.489 211 221 0.521267 0.122172

ENSMUST00000023150 828 0.3146 0.3286 0.2557 0.3493 0.199 -0.043 0.398 55.71 0.538 0.538 266 275 -0.778182 0.054545

ENSMUST00000028834 912 0.2736 0.3184 0.3651 0.3812 0.198 0.022 0.428 59.56 0.510 0.436 292 303 -0.654125 0.066007

ENSMUST00000026045 4413 0.2399 0.4049 0.2741 0.2269 0.198 0.094 0.474 49.35 0.549 0.598 1431 1470 -0.608639 0.049660

ENSMUST00000023509 1803 0.3870 0.3096 0.3140 0.2569 0.224 0.019 0.427 55.50 0.439 0.456 579 600 -0.177833 0.101667

ENSMUST00000027830 858 0.2650 0.4188 0.2257 0.3155 0.219 0.098 0.464 54.91 0.591 0.506 276 285 -0.209123 0.066667

ENSMUST00000023061 609 0.2667 0.4267 0.2381 0.3500 0.230 0.089 0.463 56.89 0.601 0.525 188 202 -0.399010 0.089109

ENSMUST00000029674 621 0.1833 0.4111 0.1911 0.4013 0.209 0.145 0.480 50.05 0.682 0.605 198 206 -0.020388 0.116505

ENSMUST00000028024 597 0.3312 0.3187 0.2585 0.3806 0.214 0.012 0.415 50.14 0.528 0.478 193 198 -0.112121 0.085859

ENSMUST00000027137 1713 0.3404 0.3298 0.2668 0.2711 0.240 0.074 0.448 53.53 0.487 0.475 536 570 0.440000 0.089474

ENSMUST00000029149 1575 0.4265 0.1831 0.4333 0.1827 0.165 -0.099 0.367 48.66 0.292 0.431 507 524 -0.680534 0.059160

ENSMUST00000025004 2070 0.3779 0.3040 0.3051 0.2673 0.220 0.034 0.434 52.87 0.445 0.436 659 689 0.151959 0.094340

ENSMUST00000022226 1941 0.3932 0.2455 0.3809 0.2744 0.205 -0.061 0.392 55.89 0.391 0.433 617 646 -0.437616 0.092879

ENSMUST00000024756 1434 0.3876 0.3230 0.3079 0.2667 0.227 -0.029 0.399 54.29 0.450 0.449 456 477 -0.220126 0.117400

ENSMUST00000025570 495 0.2602 0.3496 0.3171 0.3652 0.236 0.095 0.462 54.72 0.545 0.480 156 164 -0.652439 0.097561

ENSMUST00000026539 462 0.2667 0.3667 0.2033 0.4000 0.271 0.178 0.503 50.31 0.612 0.547 147 153 0.152287 0.065359

ENSMUST00000024930 1311 0.2586 0.3506 0.2582 0.3324 0.202 0.070 0.449 52.83 0.567 0.573 425 436 -0.433257 0.032110

ENSMUST00000026381 2001 0.1717 0.4887 0.1574 0.4475 0.267 0.147 0.498 42.16 0.735 0.577 642 666 -0.311111 0.091592

ENSMUST00000025823 990 0.2049 0.4063 0.1654 0.3893 0.254 0.216 0.518 49.37 0.677 0.596 313 329 0.639514 0.100304

ENSMUST00000022757 747 0.2736 0.3632 0.2723 0.3448 0.201 0.037 0.433 52.36 0.554 0.500 240 248 -0.263710 0.072581

ENSMUST00000022954 1041 0.2409 0.3321 0.3210 0.3640 0.187 -0.050 0.392 56.99 0.549 0.523 339 346 -0.844220 0.092486

ENSMUST00000025856 4845 0.2054 0.4989 0.1337 0.3993 0.269 0.173 0.516 43.34 0.718 0.590 1550 1614 -0.296592 0.082404

ENSMUST00000028314 945 0.3229 0.3408 0.3347 0.3363 0.249 0.012 0.436 56.72 0.498 0.455 305 314 -0.522293 0.054140

ENSMUST00000026188 2328 0.4387 0.2152 0.3827 0.2150 0.221 -0.075 0.389 51.89 0.339 0.452 756 775 -0.590968 0.045161

ENSMUST00000022150 390 0.1800 0.4300 0.1481 0.4757 0.192 0.087 0.444 49.48 0.730 0.605 126 129 -0.148837 0.031008

ENSMUST00000022218 564 0.3162 0.3529 0.3706 0.2500 0.184 -0.029 0.390 53.17 0.458 0.444 177 187 -0.440642 0.096257

ENSMUST00000023370 3333 0.2448 0.4164 0.2246 0.3174 0.203 0.043 0.436 51.43 0.605 0.570 1070 1110 -0.352252 0.065766

ENSMUST00000022330 984 0.1932 0.4962 0.1661 0.3398 0.252 0.189 0.527 45.88 0.695 0.599 315 327 -0.479511 0.055046

ENSMUST00000028129 1434 0.1535 0.5520 0.1198 0.3196 0.232 0.192 0.508 44.79 0.758 0.629 447 477 0.661426 0.106918

ENSMUST00000023686 477 0.3258 0.3561 0.2286 0.3298 0.234 0.046 0.448 56.86 0.538 0.498 145 158 0.518354 0.183544

ENSMUST00000026062 912 0.2181 0.4979 0.1914 0.4115 0.286 0.166 0.526 49.73 0.683 0.535 293 303 -0.525082 0.079208

ENSMUST00000024118 630 0.3846 0.3462 0.2826 0.2969 0.230 0.025 0.445 49.71 0.482 0.456 191 209 -0.301435 0.133971

ENSMUST00000029331 1122 0.2752 0.4159 0.1711 0.3571 0.238 0.123 0.480 52.21 0.621 0.484 356 373 0.431635 0.147453

ENSMUST00000025484 1269 0.2378 0.4116 0.2462 0.3604 0.213 0.050 0.442 53.51 0.607 0.532 405 422 -0.370379 0.075829

ENSMUST00000023494 1116 0.2403 0.4318 0.1527 0.4053 0.228 0.103 0.461 49.18 0.674 0.570 356 371 -0.004852 0.113208

ENSMUST00000028841 3243 0.3570 0.2749 0.3353 0.3193 0.226 0.019 0.434 55.35 0.456 0.472 1050 1080 -0.817685 0.068519

ENSMUST00000029666 1875 0.2940 0.3644 0.1969 0.4309 0.248 0.046 0.442 53.65 0.610 0.537 595 624 -0.393590 0.088141

ENSMUST00000026661 702 0.1882 0.4731 0.1299 0.4759 0.257 0.075 0.462 45.60 0.742 0.561 225 233 -0.035193 0.081545

ENSMUST00000023212 777 0.2537 0.4390 0.2340 0.3596 0.251 0.012 0.440 50.19 0.616 0.528 250 258 -0.499612 0.100775

ENSMUST00000028238 648 0.3669 0.2663 0.4172 0.2297 0.221 0.001 0.435 51.60 0.378 0.440 209 215 -0.399070 0.093023

ENSMUST00000027931 1332 0.2456 0.3787 0.2164 0.4458 0.215 0.075 0.455 52.03 0.634 0.521 429 443 -0.635215 0.065463

ENSMUST00000025931 1410 0.3919 0.2243 0.3518 0.2950 0.189 -0.054 0.387 52.89 0.402 0.453 455 469 -0.492537 0.055437

ENSMUST00000029795 1551 0.2395 0.4469 0.2525 0.3386 0.223 0.050 0.449 51.06 0.611 0.549 506 516 -0.456783 0.071705

ENSMUST00000025295 903 0.2000 0.5294 0.1176 0.3692 0.256 0.176 0.515 45.09 0.735 0.618 291 300 -0.346667 0.043333

ENSMUST00000022097 351 0.2447 0.3617 0.2069 0.4938 0.247 0.108 0.487 59.42 0.643 0.549 115 116 -0.488793 0.077586

ENSMUST00000023104 1983 0.3265 0.2936 0.3173 0.3629 0.232 0.012 0.436 52.82 0.499 0.481 647 660 -0.790303 0.069697

ENSMUST00000028970 1842 0.1979 0.4442 0.2361 0.3801 0.242 0.032 0.441 45.26 0.649 0.562 596 613 -0.454649 0.052202

ENSMUST00000024963 438 0.2456 0.4737 0.1881 0.4043 0.289 0.110 0.482 40.42 0.662 0.552 139 145 -0.303448 0.055172

ENSMUST00000024984 681 0.2211 0.4158 0.1734 0.3742 0.267 0.169 0.505 48.81 0.660 0.580 212 226 0.344690 0.088496

ENSMUST00000022925 1059 0.2964 0.4466 0.2052 0.3745 0.310 0.182 0.531 55.31 0.614 0.514 337 352 -0.527841 0.073864

ENSMUST00000025212 1818 0.2591 0.4127 0.1842 0.3349 0.242 0.126 0.477 49.79 0.616 0.544 576 605 0.444463 0.099174

ENSMUST00000029866 1212 0.4219 0.2359 0.4013 0.2519 0.212 -0.051 0.381 47.74 0.360 0.398 386 403 -0.257568 0.091811

ENSMUST00000027303 876 0.2446 0.4335 0.1883 0.3785 0.228 0.062 0.445 48.99 0.648 0.570 281 291 -0.580756 0.079038

ENSMUST00000023489 954 0.3532 0.1944 0.4093 0.2694 0.157 -0.013 0.403 52.52 0.371 0.442 310 317 -0.827760 0.053628

ENSMUST00000023758 1581 0.2145 0.4988 0.1405 0.4682 0.292 0.142 0.508 45.51 0.724 0.557 510 526 -0.274715 0.108365

ENSMUST00000024104 1311 0.2355 0.4680 0.2516 0.3333 0.251 0.144 0.506 49.94 0.621 0.531 417 436 -0.794037 0.103211

ENSMUST00000026036 1419 0.1894 0.4540 0.1459 0.4576 0.277 0.214 0.539 45.65 0.727 0.607 447 472 -0.216525 0.080508

ENSMUST00000023555 1452 0.3093 0.3280 0.2798 0.3545 0.236 0.034 0.441 54.49 0.531 0.515 463 483 -0.387578 0.099379

ENSMUST00000027876 2208 0.3230 0.3143 0.2991 0.3030 0.222 0.021 0.420 54.62 0.493 0.498 714 735 -0.242177 0.070748

ENSMUST00000022272 714 0.2769 0.4359 0.2229 0.3378 0.225 0.063 0.455 55.10 0.603 0.501 224 237 -0.447679 0.118143

ENSMUST00000022688 1470 0.2513 0.4824 0.1204 0.4139 0.285 0.151 0.506 50.38 0.697 0.545 472 489 0.146421 0.102249

ENSMUST00000029034 399 0.2245 0.3878 0.2816 0.3936 0.210 -0.024 0.413 46.13 0.595 0.470 126 132 -0.359849 0.075758

ENSMUST00000022650 2271 0.3854 0.2434 0.4086 0.3136 0.218 -0.025 0.407 51.96 0.407 0.408 738 756 -0.996958 0.044974

ENSMUST00000023361 2370 0.3257 0.2736 0.3254 0.3244 0.192 -0.031 0.390 55.87 0.472 0.491 767 789 -0.323194 0.051965

ENSMUST00000029043 399 0.2600 0.3800 0.3235 0.3158 0.254 0.049 0.457 48.75 0.535 0.465 127 132 -0.442424 0.060606

ENSMUST00000028672 423 0.1443 0.6186 0.1376 0.4167 0.268 0.058 0.485 36.71 0.784 0.593 134 140 -0.749286 0.078571

ENSMUST00000025968 1473 0.3532 0.3358 0.3400 0.2516 0.212 -0.002 0.406 53.96 0.451 0.437 475 490 -0.131633 0.091837

ENSMUST00000023220 1353 0.2170 0.3591 0.1830 0.3716 0.254 0.155 0.477 47.60 0.642 0.605 436 450 0.746222 0.073333

ENSMUST00000026743 1443 0.2718 0.4140 0.1557 0.3873 0.273 0.124 0.489 48.12 0.644 0.570 466 480 -0.196667 0.072917

ENSMUST00000023477 2100 0.3914 0.2280 0.3680 0.2584 0.192 -0.037 0.382 52.35 0.379 0.440 681 699 -0.238198 0.044349

ENSMUST00000025314 1533 0.2345 0.4184 0.2252 0.3147 0.206 0.095 0.471 52.18 0.613 0.582 499 510 -0.389608 0.076471

ENSMUST00000029405 2082 0.4412 0.2170 0.3635 0.2366 0.195 -0.074 0.370 52.36 0.348 0.423 676 693 -0.162482 0.064935

ENSMUST00000027886 1695 0.3529 0.2715 0.3395 0.2892 0.217 0.010 0.431 56.64 0.440 0.459 541 564 -0.358511 0.085106

ENSMUST00000026519 1920 0.3679 0.2720 0.3727 0.2484 0.202 -0.020 0.404 52.65 0.407 0.454 621 639 -0.425978 0.086072

ENSMUST00000026096 2973 0.2706 0.4240 0.2159 0.3497 0.244 0.110 0.484 50.37 0.609 0.539 962 990 -0.517980 0.058586

ENSMUST00000025806 1164 0.2336 0.3988 0.1927 0.3925 0.203 0.041 0.428 45.10 0.646 0.598 376 387 -0.491731 0.072351

ENSMUST00000025413 2898 0.2793 0.3597 0.3195 0.3076 0.229 0.047 0.453 55.66 0.521 0.502 942 965 -0.761865 0.038342

ENSMUST00000026541 681 0.1839 0.4540 0.2147 0.3905 0.303 0.163 0.521 44.06 0.674 0.588 215 226 -0.412389 0.066372

ENSMUST00000027394 774 0.3254 0.3541 0.2139 0.3557 0.275 0.118 0.492 54.18 0.563 0.565 254 257 -0.555642 0.031128

ENSMUST00000023846 1677 0.1872 0.3957 0.2038 0.4513 0.235 0.075 0.456 48.49 0.679 0.561 546 558 -0.491219 0.041219

ENSMUST00000023222 3867 0.2698 0.3887 0.1877 0.3505 0.248 0.101 0.470 49.68 0.611 0.591 1259 1288 -0.071662 0.065217

ENSMUST00000024999 936 0.2348 0.4280 0.1784 0.3262 0.241 0.126 0.483 49.84 0.643 0.613 294 311 0.119293 0.086817

ENSMUST00000022962 894 0.4333 0.2381 0.4085 0.2589 0.226 -0.031 0.416 50.78 0.362 0.416 279 297 -0.717845 0.101010

ENSMUST00000028167 2184 0.3139 0.3499 0.2679 0.3053 0.218 0.028 0.429 53.75 0.522 0.503 697 727 -0.209216 0.078404

ENSMUST00000023737 1191 0.1681 0.4145 0.1667 0.4202 0.226 0.179 0.503 50.02 0.712 0.647 382 396 -0.147980 0.073232

ENSMUST00000023217 2199 0.2327 0.4165 0.1757 0.4165 0.243 0.111 0.470 47.30 0.666 0.586 704 732 -0.485383 0.075137

ENSMUST00000028977 2244 0.3131 0.3359 0.2538 0.4085 0.222 -0.023 0.413 51.82 0.562 0.500 724 747 -0.898527 0.050870

ENSMUST00000029761 795 0.3077 0.3125 0.3600 0.2872 0.205 0.021 0.431 52.83 0.467 0.485 255 264 -0.742803 0.087121

ENSMUST00000025580 735 0.3385 0.3128 0.2649 0.3193 0.242 0.061 0.441 53.39 0.498 0.451 229 244 0.453279 0.081967

ENSMUST00000025246 648 0.2545 0.5212 0.2153 0.3308 0.292 0.150 0.507 52.85 0.640 0.524 203 215 -0.460930 0.120930

ENSMUST00000023070 864 0.1984 0.4980 0.1475 0.3333 0.239 0.150 0.498 43.71 0.703 0.585 273 287 0.065854 0.094077

ENSMUST00000025322 996 0.2634 0.3321 0.3020 0.3562 0.201 0.020 0.419 52.41 0.543 0.515 313 331 -0.293051 0.096677

ENSMUST00000026912 1146 0.4337 0.1406 0.5081 0.2305 0.183 -0.113 0.363 46.88 0.275 0.355 364 381 -1.081.627 0.036745

ENSMUST00000027491 1245 0.1952 0.4264 0.1656 0.4441 0.237 0.097 0.462 47.75 0.699 0.571 396 414 -0.071739 0.072464

ENSMUST00000022960 1338 0.4128 0.2529 0.3592 0.2982 0.233 0.004 0.419 49.96 0.405 0.413 425 445 -0.326966 0.114607

ENSMUST00000026859 1560 0.3889 0.2639 0.3167 0.2353 0.191 -0.026 0.387 53.11 0.402 0.451 493 519 0.453372 0.121387

ENSMUST00000023359 1035 0.2341 0.3056 0.3310 0.3768 0.187 -0.020 0.413 51.37 0.542 0.530 334 344 -0.946221 0.043605

ENSMUST00000025562 2631 0.2729 0.3424 0.2245 0.4336 0.200 -0.046 0.393 50.99 0.611 0.553 855 876 -1.162.899 0.027397

ENSMUST00000029183 1758 0.2206 0.4268 0.1545 0.4080 0.256 0.189 0.528 48.54 0.686 0.587 570 585 -0.397094 0.073504

ENSMUST00000027380 894 0.2304 0.4304 0.1630 0.4346 0.259 0.109 0.479 46.47 0.681 0.567 282 297 -0.319192 0.090909

ENSMUST00000027726 918 0.2760 0.3480 0.1807 0.4286 0.229 0.130 0.471 49.59 0.620 0.543 295 305 -0.137705 0.081967

ENSMUST00000026538 873 0.2978 0.3867 0.2795 0.2925 0.278 0.089 0.479 56.43 0.532 0.513 280 290 -0.095862 0.065517

ENSMUST00000024802 501 0.2313 0.4478 0.1750 0.4091 0.267 0.144 0.510 46.58 0.669 0.566 157 166 -0.309639 0.102410

ENSMUST00000028759 2667 0.2186 0.4116 0.1849 0.3699 0.202 0.073 0.454 50.25 0.655 0.616 852 888 -0.215878 0.070946

ENSMUST00000027634 264 0.2857 0.3929 0.2576 0.4219 0.296 0.038 0.463 61.00 0.598 0.487 82 87 -0.900000 0.091954

ENSMUST00000028554 1575 0.2317 0.4381 0.1791 0.3422 0.206 0.083 0.444 50.54 0.652 0.591 511 524 0.092939 0.083969

ENSMUST00000029369 1404 0.3368 0.3135 0.2386 0.3305 0.229 0.054 0.451 53.28 0.521 0.507 457 467 -0.271092 0.077088

ENSMUST00000027988 822 0.1667 0.4905 0.0976 0.5198 0.230 0.093 0.468 47.16 0.791 0.603 263 273 -0.411355 0.095238

ENSMUST00000025663 4152 0.2961 0.3729 0.2372 0.3560 0.222 0.033 0.443 54.70 0.568 0.511 1341 1383 -0.368474 0.098337

ENSMUST00000029677 1635 0.2090 0.4607 0.2165 0.3373 0.224 0.071 0.460 48.04 0.651 0.612 530 544 -0.595221 0.053309

ENSMUST00000026495 1662 0.3326 0.3458 0.1711 0.3708 0.256 0.130 0.490 48.96 0.577 0.530 541 553 -0.100181 0.056058

ENSMUST00000026818 540 0.3448 0.2414 0.3053 0.3846 0.153 -0.133 0.312 54.13 0.471 0.456 170 179 0.103911 0.100559

ENSMUST00000022597 2595 0.4196 0.2492 0.3762 0.2900 0.206 -0.018 0.410 53.76 0.396 0.409 830 864 -0.542708 0.096065

ENSMUST00000026507 2694 0.3807 0.2884 0.3198 0.3177 0.233 -0.001 0.422 55.59 0.454 0.456 867 897 -0.453400 0.080268

ENSMUST00000025844 1116 0.2195 0.4669 0.2169 0.3808 0.271 0.122 0.496 49.88 0.656 0.544 355 371 -0.529380 0.102426

ENSMUST00000023525 1323 0.3569 0.3127 0.2927 0.3280 0.234 -0.043 0.402 58.97 0.489 0.503 425 440 -0.644318 0.068182

ENSMUST00000024786 1605 0.2045 0.4888 0.1189 0.4461 0.265 0.162 0.509 42.24 0.741 0.606 505 534 -0.677153 0.056180

ENSMUST00000025755 1125 0.1755 0.5099 0.1481 0.3746 0.262 0.221 0.558 45.00 0.731 0.618 360 374 -0.620321 0.069519

ENSMUST00000025106 429 0.2800 0.4200 0.1696 0.4673 0.296 0.159 0.518 58.86 0.662 0.535 139 142 -0.630986 0.070423

ENSMUST00000026823 1746 0.2153 0.4493 0.1507 0.4034 0.235 0.169 0.489 46.52 0.692 0.566 552 581 0.296558 0.101549

ENSMUST00000025349 1491 0.2964 0.3247 0.3627 0.2834 0.234 0.040 0.445 54.00 0.474 0.487 485 496 -0.635282 0.074597

ENSMUST00000022678 729 0.2526 0.3368 0.3631 0.3059 0.224 0.091 0.467 58.29 0.507 0.501 229 242 -0.316529 0.095041

ENSMUST00000026217 2238 0.4139 0.2149 0.3517 0.3054 0.191 -0.086 0.360 52.11 0.392 0.433 709 745 -0.285906 0.072483

ENSMUST00000029641 1488 0.3358 0.3333 0.3221 0.3179 0.228 0.003 0.421 56.16 0.487 0.441 485 495 -0.167677 0.117172

ENSMUST00000023276 2883 0.2105 0.4506 0.1775 0.3986 0.241 0.125 0.480 48.97 0.682 0.565 936 960 -0.160417 0.073958

ENSMUST00000025684 1605 0.1516 0.5501 0.0848 0.5217 0.300 0.176 0.515 40.20 0.813 0.586 513 534 -0.362734 0.082397

ENSMUST00000029822 1359 0.2178 0.4829 0.1843 0.3378 0.249 0.125 0.488 52.45 0.664 0.526 428 452 0.245575 0.141593

ENSMUST00000028619 939 0.2811 0.3614 0.2656 0.3410 0.264 0.098 0.470 54.04 0.550 0.470 298 312 0.094551 0.099359

ENSMUST00000027975 1017 0.3446 0.3633 0.2833 0.3045 0.215 -0.008 0.410 56.17 0.506 0.485 324 338 -0.391124 0.091716

ENSMUST00000027267 2754 0.4267 0.2233 0.3962 0.2500 0.196 -0.099 0.366 51.55 0.355 0.405 889 917 -0.534787 0.068702

ENSMUST00000026122 1530 0.2703 0.4042 0.2342 0.4282 0.325 0.086 0.480 50.83 0.614 0.515 498 509 -0.373477 0.100196

ENSMUST00000023068 3747 0.3915 0.2252 0.4475 0.2879 0.220 -0.020 0.414 52.84 0.368 0.396 1220 1248 -0.679167 0.060897

ENSMUST00000023048 1392 0.2435 0.3912 0.1945 0.3757 0.207 0.054 0.430 50.30 0.630 0.569 446 463 -0.138013 0.069114

ENSMUST00000029771 885 0.2333 0.3708 0.3333 0.3158 0.203 -0.007 0.411 54.50 0.540 0.484 287 294 -0.185034 0.091837

ENSMUST00000027768 6732 0.4094 0.1941 0.3678 0.2563 0.190 -0.043 0.386 50.78 0.361 0.448 2196 2243 -0.439902 0.054391

ENSMUST00000024749 2085 0.3907 0.2722 0.2762 0.3000 0.229 0.003 0.421 56.55 0.457 0.500 670 694 -0.375648 0.061960

ENSMUST00000023179 2058 0.3081 0.3876 0.2431 0.3921 0.235 0.023 0.452 51.38 0.580 0.519 671 685 -0.867007 0.075912

ENSMUST00000025598 888 0.3527 0.3571 0.2476 0.3467 0.255 0.054 0.446 58.55 0.532 0.468 280 295 -0.247797 0.098305

ENSMUST00000029444 3423 0.3665 0.2559 0.3430 0.2921 0.202 -0.077 0.377 56.83 0.429 0.490 1116 1140 -0.538684 0.044737

ENSMUST00000025305 765 0.3448 0.3498 0.2513 0.2798 0.217 0.049 0.433 54.76 0.510 0.543 245 254 -0.658268 0.082677

ENSMUST00000029438 3633 0.3387 0.3337 0.2441 0.2964 0.239 0.060 0.454 54.52 0.513 0.515 1183 1210 -0.257934 0.066942

ENSMUST00000026470 1515 0.2177 0.4545 0.1582 0.3941 0.224 0.082 0.461 49.47 0.688 0.591 490 504 -0.265873 0.077381

ENSMUST00000023044 1488 0.1858 0.4707 0.1624 0.4187 0.231 0.076 0.463 47.86 0.714 0.593 479 495 -0.455354 0.074747

ENSMUST00000025254 1026 0.2127 0.5522 0.1198 0.4712 0.274 0.045 0.456 43.67 0.748 0.563 329 341 -0.326100 0.102639

ENSMUST00000027071 867 0.3478 0.3217 0.3443 0.2865 0.215 0.020 0.429 61.00 0.454 0.433 280 288 -0.302431 0.072917

ENSMUST00000024810 1968 0.1924 0.4569 0.1420 0.4747 0.256 0.118 0.479 43.57 0.733 0.601 632 655 -0.489771 0.077863

ENSMUST00000024470 1050 0.2203 0.4406 0.1103 0.4692 0.284 0.177 0.512 45.67 0.729 0.615 340 349 -0.277937 0.088825

ENSMUST00000022813 1683 0.2756 0.3549 0.2309 0.3060 0.206 0.030 0.414 50.66 0.564 0.613 553 560 -0.310893 0.044643

ENSMUST00000028995 573 0.1768 0.4390 0.1840 0.3419 0.205 0.098 0.451 45.77 0.679 0.591 184 190 0.310526 0.073684

ENSMUST00000026416 1041 0.3039 0.3675 0.2385 0.3443 0.220 0.030 0.423 52.33 0.560 0.520 336 346 -0.114740 0.089595

ENSMUST00000028636 1344 0.2801 0.3697 0.2676 0.3364 0.203 0.026 0.429 54.34 0.557 0.513 431 447 -0.074497 0.071588

ENSMUST00000026997 2184 0.3772 0.2669 0.3283 0.3165 0.222 0.012 0.437 52.92 0.451 0.476 709 727 -0.951719 0.042641

ENSMUST00000025393 1656 0.3698 0.2823 0.2729 0.3187 0.214 0.018 0.431 55.60 0.472 0.509 534 551 -0.373321 0.070780

ENSMUST00000025761 816 0.2414 0.3695 0.2150 0.4231 0.205 0.032 0.442 50.90 0.632 0.579 258 271 -0.799262 0.055351

ENSMUST00000029573 1014 0.2892 0.2972 0.3678 0.3264 0.178 0.039 0.424 53.36 0.477 0.452 321 337 -0.475371 0.047478

ENSMUST00000024005 639 0.3436 0.3190 0.2628 0.3600 0.188 -0.036 0.394 53.90 0.522 0.517 203 212 -0.691038 0.084906

ENSMUST00000026988 732 0.1968 0.4096 0.0938 0.5134 0.246 0.179 0.500 46.35 0.759 0.620 228 243 -0.128395 0.094650

ENSMUST00000028223 1398 0.3850 0.2914 0.3158 0.2748 0.203 -0.042 0.385 58.34 0.436 0.457 447 465 -0.154839 0.083871

ENSMUST00000025645 3057 0.2380 0.3906 0.1776 0.3750 0.225 0.063 0.437 47.22 0.645 0.625 987 1018 -0.236542 0.056974

ENSMUST00000022189 2136 0.3302 0.3097 0.2598 0.3922 0.239 0.029 0.450 54.86 0.539 0.517 700 711 -0.972011 0.060478

ENSMUST00000029416 1599 0.2929 0.3410 0.3039 0.2850 0.180 0.010 0.427 56.00 0.506 0.503 524 532 -0.846617 0.048872

ENSMUST00000022082 324 0.2716 0.3951 0.2471 0.3816 0.229 0.109 0.481 49.42 0.587 0.495 104 107 0.042991 0.065421

ENSMUST00000027476 366 0.2523 0.3738 0.1589 0.3431 0.245 0.119 0.462 42.59 0.630 0.631 119 121 0.474380 0.033058

ENSMUST00000022172 2559 0.3636 0.2712 0.3884 0.2747 0.198 -0.038 0.404 54.82 0.412 0.435 826 852 -0.634624 0.053991

ENSMUST00000028304 564 0.2662 0.3597 0.2357 0.4567 0.225 0.053 0.438 50.39 0.607 0.481 178 187 0.094118 0.085561

ENSMUST00000025706 1236 0.2478 0.4218 0.1746 0.3701 0.277 0.151 0.503 49.02 0.649 0.610 396 411 -0.108272 0.065693

ENSMUST00000026735 1221 0.2389 0.3376 0.2287 0.3982 0.207 0.081 0.457 54.61 0.610 0.568 392 406 -0.372906 0.046798

ENSMUST00000026499 726 0.4789 0.2053 0.4235 0.1747 0.235 -0.040 0.394 57.37 0.294 0.414 231 241 -0.311203 0.099585

ENSMUST00000029060 3144 0.1761 0.4859 0.1140 0.4743 0.268 0.165 0.504 45.25 0.760 0.570 1006 1047 0.061891 0.098376

ENSMUST00000025751 2982 0.2491 0.3982 0.1683 0.4233 0.255 0.120 0.485 46.56 0.659 0.575 980 993 -0.488721 0.041289

ENSMUST00000028162 1155 0.1700 0.4667 0.1119 0.4877 0.241 0.148 0.499 44.46 0.769 0.614 363 384 -0.282552 0.093750

ENSMUST00000026891 876 0.2594 0.3389 0.2212 0.4319 0.236 0.058 0.442 53.74 0.607 0.536 285 291 -0.031959 0.051546

ENSMUST00000024725 2562 0.3383 0.2653 0.4141 0.2500 0.188 -0.017 0.405 53.96 0.400 0.461 829 853 -0.651817 0.071512

ENSMUST00000022913 1413 0.3298 0.3325 0.2185 0.3524 0.204 0.049 0.420 55.11 0.545 0.483 448 470 0.322128 0.119149

ENSMUST00000029078 783 0.3382 0.3816 0.2228 0.3468 0.284 0.139 0.508 53.13 0.556 0.506 250 260 -0.555769 0.100000

ENSMUST00000025356 528 0.2579 0.3962 0.1935 0.3220 0.207 0.095 0.452 55.32 0.608 0.539 166 175 0.794857 0.148571

ENSMUST00000026144 735 0.2379 0.3932 0.1179 0.4278 0.254 0.124 0.476 42.27 0.691 0.600 233 244 0.206148 0.032787

ENSMUST00000027792 261 0.2667 0.3500 0.2742 0.4333 0.214 -0.021 0.400 61.00 0.588 0.484 80 86 -0.413954 0.081395

ENSMUST00000025646 1737 0.1835 0.4778 0.1637 0.3608 0.251 0.213 0.524 45.37 0.702 0.573 550 578 0.352768 0.114187

ENSMUST00000022078 1836 0.2955 0.3785 0.2717 0.3442 0.253 0.076 0.458 55.02 0.550 0.475 589 611 -0.006056 0.098200

ENSMUST00000025025 1104 0.1900 0.5452 0.1000 0.3626 0.300 0.251 0.565 41.14 0.752 0.608 359 367 0.076839 0.079019

ENSMUST00000025760 1362 0.3081 0.3430 0.2595 0.3723 0.211 0.047 0.440 57.52 0.551 0.508 434 453 -0.442826 0.112583

ENSMUST00000023113 615 0.2138 0.4340 0.1908 0.4539 0.247 -0.029 0.398 48.66 0.679 0.549 196 204 -0.139706 0.112745

ENSMUST00000023407 1398 0.3073 0.2961 0.2960 0.4049 0.219 -0.015 0.401 51.89 0.528 0.477 451 465 -0.470968 0.096774

ENSMUST00000028951 1587 0.1752 0.5023 0.1512 0.3947 0.265 0.136 0.498 40.77 0.730 0.616 518 528 -0.541667 0.035985

ENSMUST00000025137 1974 0.4394 0.2177 0.3951 0.2658 0.201 -0.065 0.386 50.48 0.360 0.405 634 657 -0.608219 0.089802

ENSMUST00000029170 3192 0.3594 0.2740 0.3562 0.2815 0.201 -0.036 0.394 57.18 0.427 0.450 1028 1063 -0.274882 0.077140

ENSMUST00000027754 1578 0.2467 0.3990 0.2274 0.4264 0.254 0.061 0.448 51.77 0.629 0.531 504 525 -0.451429 0.080000

ENSMUST00000024171 3057 0.4788 0.1812 0.3499 0.2964 0.211 -0.081 0.372 48.61 0.357 0.403 991 1018 -0.404715 0.075639

ENSMUST00000027425 810 0.1733 0.5067 0.1144 0.4699 0.289 0.229 0.546 45.44 0.763 0.586 262 269 -0.164684 0.096654

ENSMUST00000027438 2124 0.4513 0.2112 0.4204 0.2379 0.256 -0.074 0.408 49.93 0.338 0.447 696 707 -1.092.928 0.050919

ENSMUST00000023112 789 0.1854 0.5024 0.1414 0.4809 0.243 0.092 0.475 47.31 0.743 0.570 257 262 -0.379771 0.099237

ENSMUST00000029131 1989 0.2220 0.4388 0.1578 0.3617 0.235 0.104 0.467 48.04 0.674 0.606 644 662 -0.014653 0.057402

ENSMUST00000029433 2409 0.4079 0.2540 0.3033 0.3050 0.213 -0.033 0.405 52.52 0.432 0.458 778 802 -0.574564 0.079800

ENSMUST00000027518 612 0.2727 0.3515 0.2432 0.3537 0.215 0.080 0.466 55.59 0.576 0.527 191 203 -0.506404 0.088670

ENSMUST00000028515 1374 0.2195 0.4986 0.1761 0.3686 0.295 0.175 0.507 49.68 0.676 0.517 432 457 0.219694 0.105033

ENSMUST00000025833 1866 0.2959 0.3633 0.2842 0.3248 0.213 0.018 0.424 53.58 0.535 0.512 594 621 -0.397585 0.083736

ENSMUST00000025075 783 0.2598 0.4461 0.2120 0.3563 0.256 0.127 0.494 52.76 0.624 0.533 245 260 -0.196538 0.061538

ENSMUST00000028098 1302 0.4333 0.1697 0.4795 0.2287 0.179 -0.120 0.340 45.88 0.294 0.366 418 433 -0.177136 0.080831

ENSMUST00000025656 1506 0.3495 0.3010 0.2987 0.3162 0.231 -0.025 0.410 54.36 0.476 0.482 481 501 -0.134331 0.079840

ENSMUST00000027997 546 0.2846 0.4472 0.1857 0.4609 0.309 0.072 0.463 40.08 0.651 0.492 175 181 -0.517680 0.099448

ENSMUST00000027564 1170 0.2900 0.3633 0.3042 0.3509 0.261 0.102 0.481 52.77 0.537 0.464 376 389 -0.279692 0.105398

ENSMUST00000025427 1233 0.4198 0.2594 0.3183 0.3379 0.244 0.006 0.433 58.34 0.441 0.447 397 410 -0.769512 0.078049

ENSMUST00000026449 591 0.3046 0.3775 0.2649 0.2897 0.179 0.028 0.416 58.32 0.535 0.546 185 196 -0.301531 0.056122

ENSMUST00000028858 3180 0.4414 0.2282 0.4143 0.2238 0.218 -0.048 0.398 49.08 0.339 0.408 1012 1059 -0.451369 0.088763

ENSMUST00000029504 1140 0.2414 0.4639 0.1569 0.3920 0.290 0.190 0.527 44.35 0.672 0.537 366 379 -0.019525 0.121372

ENSMUST00000027052 1617 0.4101 0.2354 0.3830 0.2356 0.203 -0.028 0.410 57.65 0.367 0.446 520 538 -0.686989 0.059480

ENSMUST00000026009 549 0.2174 0.4275 0.2778 0.3615 0.247 0.070 0.455 52.69 0.602 0.500 176 182 -0.282418 0.065934

ENSMUST00000022494 621 0.2262 0.3810 0.1871 0.4122 0.247 0.134 0.469 55.92 0.651 0.545 192 206 0.545146 0.140777

ENSMUST00000029629 579 0.1582 0.5190 0.1857 0.4211 0.275 0.219 0.545 50.80 0.730 0.587 189 192 -0.410417 0.067708

ENSMUST00000022721 1026 0.2810 0.3321 0.3418 0.3500 0.192 -0.047 0.399 58.61 0.515 0.473 326 341 -0.275073 0.170088

ENSMUST00000027860 345 0.2584 0.3483 0.3093 0.2667 0.210 0.094 0.463 51.84 0.509 0.512 108 114 0.178947 0.052632

ENSMUST00000024570 1875 0.3048 0.3327 0.3013 0.3194 0.203 0.046 0.437 52.52 0.509 0.490 599 624 -0.207372 0.078526

ENSMUST00000025546 1428 0.2022 0.4399 0.1915 0.4606 0.260 0.076 0.464 46.65 0.688 0.548 455 475 -0.287790 0.088421

ENSMUST00000022348 3294 0.2999 0.3398 0.3060 0.3416 0.214 0.003 0.417 54.94 0.523 0.490 1066 1097 -0.371559 0.081130

ENSMUST00000027849 621 0.1635 0.4780 0.2805 0.3312 0.192 0.088 0.470 46.48 0.640 0.552 200 206 -0.692233 0.087379

ENSMUST00000027173 1272 0.3506 0.2866 0.3365 0.2819 0.227 -0.013 0.415 54.17 0.445 0.467 400 423 -0.317021 0.080378

ENSMUST00000023754 882 0.2588 0.3922 0.1333 0.3778 0.267 0.132 0.484 48.34 0.654 0.582 283 293 0.663822 0.088737

ENSMUST00000025666 1656 0.3305 0.3069 0.2834 0.2835 0.192 0.019 0.407 56.22 0.477 0.465 526 551 0.457532 0.085299

ENSMUST00000027991 618 0.2381 0.4354 0.2876 0.3862 0.324 0.145 0.523 54.04 0.603 0.488 199 205 -0.619512 0.078049

ENSMUST00000022119 636 0.3642 0.2160 0.3882 0.3427 0.200 -0.117 0.351 49.07 0.416 0.447 202 211 -0.354028 0.113744

ENSMUST00000023344 1314 0.3470 0.3333 0.2722 0.3085 0.207 -0.010 0.403 52.37 0.495 0.437 422 437 0.252632 0.123570

ENSMUST00000029199 873 0.2611 0.3850 0.1886 0.4133 0.214 0.015 0.429 51.04 0.638 0.569 282 290 -0.619310 0.065517

ENSMUST00000022665 2187 0.2100 0.4650 0.1654 0.4113 0.237 0.094 0.464 45.58 0.694 0.572 696 728 -0.288599 0.087912

ENSMUST00000022064 1533 0.2712 0.3939 0.2276 0.3431 0.242 0.109 0.464 51.21 0.592 0.545 500 510 -0.009412 0.064706

ENSMUST00000023693 1542 0.2810 0.3738 0.3067 0.2885 0.194 0.008 0.421 55.68 0.523 0.494 497 513 -0.376998 0.064327

ENSMUST00000022705 813 0.3434 0.2424 0.3302 0.3415 0.221 0.074 0.459 54.70 0.459 0.485 257 270 -0.605926 0.029630

ENSMUST00000023247 405 0.3363 0.3540 0.2653 0.2826 0.248 0.098 0.462 56.29 0.508 0.473 130 134 0.436567 0.052239

ENSMUST00000027233 2394 0.2689 0.4003 0.2383 0.3477 0.226 0.079 0.460 53.05 0.585 0.496 761 797 0.043287 0.126725

ENSMUST00000024697 363 0.2286 0.4476 0.1546 0.3298 0.158 0.065 0.419 50.66 0.667 0.619 117 120 -0.417500 0.066667

ENSMUST00000022419 621 0.1582 0.5198 0.1720 0.3600 0.293 0.232 0.557 45.99 0.726 0.591 201 206 -0.139320 0.072816

ENSMUST00000026420 348 0.2500 0.4688 0.2333 0.2738 0.175 -0.046 0.381 49.98 0.602 0.559 113 115 -0.660000 0.052174

ENSMUST00000023897 783 0.4259 0.2407 0.3487 0.2260 0.177 -0.063 0.377 50.85 0.365 0.447 252 260 -0.282308 0.065385

ENSMUST00000027302 1362 0.1957 0.4155 0.2083 0.4012 0.207 0.063 0.451 49.41 0.664 0.595 441 453 -0.425166 0.075055

ENSMUST00000023396 729 0.3011 0.3387 0.3371 0.3671 0.223 -0.020 0.415 54.58 0.513 0.466 236 242 -0.457438 0.107438

ENSMUST00000026303 1551 0.2957 0.3894 0.2337 0.3099 0.228 0.075 0.455 52.84 0.565 0.543 497 516 -0.354457 0.075581

ENSMUST00000028071 975 0.3674 0.2917 0.3574 0.2500 0.169 -0.090 0.372 53.85 0.420 0.439 312 324 -0.629938 0.070988

ENSMUST00000025956 2586 0.2895 0.3599 0.2841 0.3822 0.253 0.033 0.439 54.65 0.555 0.477 823 861 -0.295354 0.095238

ENSMUST00000025569 264 0.2222 0.4583 0.1538 0.3559 0.230 0.193 0.500 49.10 0.675 0.510 80 87 0.952874 0.114943

ENSMUST00000024782 1179 0.2334 0.5899 0.0887 0.3177 0.307 0.248 0.565 40.67 0.733 0.566 375 392 -0.016071 0.125000

ENSMUST00000027178 4956 0.3085 0.3223 0.2758 0.3333 0.218 0.003 0.419 53.78 0.524 0.507 1593 1651 -0.279285 0.086008

ENSMUST00000022593 5685 0.4551 0.2034 0.3787 0.2395 0.223 -0.085 0.383 51.05 0.341 0.426 1847 1894 -0.501690 0.063886

ENSMUST00000027379 2199 0.3780 0.3277 0.3011 0.3267 0.275 0.068 0.462 55.76 0.480 0.449 708 732 -0.361339 0.081967

ENSMUST00000025957 1074 0.3442 0.2935 0.3619 0.2713 0.255 0.073 0.456 53.59 0.435 0.452 340 357 0.005042 0.095238

ENSMUST00000028332 1521 0.2751 0.4115 0.2095 0.3538 0.245 0.072 0.464 49.58 0.607 0.551 483 506 -0.179249 0.126482

ENSMUST00000028306 579 0.2500 0.3487 0.2535 0.4211 0.263 0.013 0.432 53.54 0.596 0.498 183 192 0.184375 0.078125

ENSMUST00000022906 2130 0.3155 0.3517 0.3031 0.2754 0.220 0.044 0.443 56.41 0.498 0.490 677 709 -0.064316 0.098731

ENSMUST00000027941 546 0.2598 0.3622 0.2603 0.4275 0.189 -0.029 0.403 49.83 0.597 0.510 176 181 -0.748066 0.033149

ENSMUST00000025875 1572 0.2483 0.4115 0.1679 0.3907 0.254 0.131 0.481 49.63 0.645 0.522 499 523 0.524092 0.076482

ENSMUST00000023283 2109 0.1913 0.4383 0.1993 0.3569 0.243 0.197 0.510 46.59 0.668 0.586 665 702 0.049288 0.128205

ENSMUST00000026841 1428 0.4238 0.2403 0.2959 0.2522 0.250 0.048 0.449 49.02 0.398 0.465 452 475 -0.097053 0.069474

ENSMUST00000027012 1122 0.3250 0.3500 0.3903 0.2590 0.253 0.058 0.460 55.96 0.454 0.443 359 373 -0.480965 0.085791

ENSMUST00000023065 1023 0.3745 0.2819 0.3788 0.2489 0.228 -0.032 0.413 59.13 0.401 0.448 329 340 -0.299412 0.073529

ENSMUST00000025060 1023 0.2086 0.4676 0.1946 0.4000 0.274 0.228 0.536 50.41 0.675 0.543 332 340 -0.061471 0.061765

ENSMUST00000023934 444 0.3171 0.4309 0.0841 0.3981 0.322 0.147 0.507 43.04 0.662 0.565 142 147 0.091837 0.081633

ENSMUST00000028062 1401 0.2377 0.4725 0.1340 0.4469 0.318 0.231 0.554 42.64 0.710 0.565 455 466 -0.833476 0.051502

ENSMUST00000022831 2016 0.2547 0.3566 0.1825 0.4393 0.204 0.072 0.454 49.26 0.642 0.586 650 671 -0.572131 0.070045

ENSMUST00000028161 1800 0.2668 0.4847 0.1373 0.3580 0.260 0.088 0.474 48.78 0.667 0.552 574 599 -0.192487 0.105175

ENSMUST00000028005 462 0.2406 0.4812 0.1636 0.3077 0.235 0.089 0.466 47.79 0.658 0.560 146 153 0.196078 0.143791

ENSMUST00000024233 1689 0.2287 0.4215 0.2559 0.3447 0.199 0.040 0.440 55.02 0.610 0.563 541 562 -0.755872 0.064057

ENSMUST00000026243 2751 0.3626 0.2822 0.3285 0.3163 0.227 -0.011 0.416 56.52 0.456 0.468 871 916 -0.338865 0.094978

ENSMUST00000027956 1434 0.3211 0.3159 0.3314 0.3133 0.194 -0.027 0.413 60.51 0.484 0.488 465 477 -0.591824 0.096436

ENSMUST00000023610 2907 0.2639 0.4116 0.2454 0.3285 0.223 0.019 0.440 53.72 0.589 0.545 935 968 -0.485847 0.073347

ENSMUST00000028256 513 0.2000 0.5333 0.1667 0.4343 0.224 0.099 0.494 46.82 0.719 0.533 160 170 -0.869412 0.164706

ENSMUST00000028241 855 0.2819 0.3744 0.2140 0.3713 0.250 0.087 0.465 55.00 0.586 0.515 273 284 0.048591 0.059859

ENSMUST00000022634 657 0.2663 0.4083 0.2622 0.3269 0.232 0.119 0.495 56.53 0.577 0.523 208 218 -0.720642 0.055046

ENSMUST00000028737 1032 0.3308 0.2481 0.3592 0.2960 0.187 -0.063 0.385 51.00 0.438 0.480 338 343 -0.781633 0.040816

ENSMUST00000027370 1158 0.1830 0.4606 0.1894 0.3870 0.209 0.142 0.492 46.73 0.693 0.603 374 385 -0.539481 0.067532

ENSMUST00000022327 2172 0.2582 0.4624 0.1707 0.2984 0.269 0.115 0.491 44.28 0.636 0.597 704 723 -0.365975 0.076072

ENSMUST00000029325 1197 0.4348 0.2391 0.3345 0.2364 0.169 -0.119 0.334 54.02 0.374 0.433 380 398 -0.106282 0.110553

ENSMUST00000028831 2502 0.3748 0.2594 0.3524 0.2730 0.205 -0.008 0.415 54.57 0.413 0.456 814 833 -0.343217 0.068427

ENSMUST00000025867 1650 0.2533 0.4170 0.1972 0.3547 0.221 0.068 0.450 49.03 0.625 0.574 536 549 -0.483242 0.063752

ENSMUST00000025181 1110 0.2061 0.4020 0.1459 0.4745 0.256 0.169 0.513 46.07 0.709 0.602 351 369 -0.387805 0.092141

ENSMUST00000023237 1617 0.2556 0.3422 0.1779 0.4046 0.227 0.098 0.452 50.39 0.630 0.600 524 538 0.048513 0.074349

ENSMUST00000026698 1512 0.2751 0.3923 0.2236 0.3000 0.232 0.074 0.461 51.45 0.577 0.541 492 503 -0.415905 0.031809

ENSMUST00000027414 3219 0.2690 0.3540 0.1886 0.3991 0.230 0.090 0.450 50.23 0.619 0.580 1050 1072 -0.175373 0.061567

ENSMUST00000026480 1161 0.3204 0.3521 0.2420 0.4328 0.232 -0.019 0.416 48.05 0.576 0.504 375 386 -0.702850 0.095855

ENSMUST00000022725 1554 0.2540 0.3972 0.2624 0.3487 0.208 0.035 0.434 53.21 0.588 0.524 498 517 -0.311992 0.114120

ENSMUST00000022881 1443 0.2973 0.3324 0.2861 0.3287 0.206 0.014 0.423 54.88 0.525 0.517 461 480 -0.267917 0.072917

ENSMUST00000029891 990 0.4275 0.2268 0.3745 0.2512 0.198 -0.061 0.373 52.91 0.358 0.413 316 329 0.078419 0.136778

ENSMUST00000028329 1278 0.1910 0.4060 0.1595 0.4309 0.252 0.211 0.533 45.08 0.704 0.647 415 425 -0.523059 0.032941

ENSMUST00000029663 960 0.3982 0.2036 0.2932 0.3865 0.245 -0.065 0.383 52.76 0.461 0.484 308 319 -0.468025 0.031348

ENSMUST00000028838 1989 0.2500 0.4261 0.2072 0.3640 0.246 0.116 0.484 50.06 0.627 0.544 630 662 -0.201964 0.095166

ENSMUST00000022508 1281 0.4398 0.2410 0.3117 0.3110 0.209 -0.063 0.385 55.76 0.410 0.419 410 426 -0.500235 0.098592

ENSMUST00000028826 2034 0.2408 0.3709 0.3302 0.3792 0.201 -0.005 0.439 55.27 0.563 0.532 661 677 -1.551.699 0.064993

ENSMUST00000028342 360 0.1625 0.5000 0.1500 0.5263 0.277 0.134 0.508 56.67 0.763 0.566 118 119 -0.928571 0.025210

ENSMUST00000027760 2307 0.2392 0.4441 0.2085 0.3964 0.264 0.151 0.511 49.68 0.645 0.521 738 768 -0.499740 0.065104

ENSMUST00000026434 603 0.1085 0.4031 0.1775 0.6062 0.237 0.109 0.477 40.35 0.772 0.580 193 200 -0.903500 0.030000

ENSMUST00000023810 414 0.3846 0.3419 0.2524 0.2100 0.173 -0.093 0.348 44.26 0.462 0.569 132 137 -0.917518 0.058394

ENSMUST00000025983 1140 0.2967 0.3767 0.1738 0.4201 0.281 0.208 0.530 53.72 0.621 0.534 364 379 -0.159103 0.100264

ENSMUST00000029553 3318 0.3786 0.2801 0.2648 0.2782 0.223 -0.002 0.434 54.65 0.461 0.514 1081 1105 -0.707873 0.059729

ENSMUST00000022197 1017 0.2909 0.3709 0.2607 0.3484 0.263 0.051 0.453 52.12 0.559 0.505 320 338 -0.074260 0.136095

ENSMUST00000024764 615 0.3034 0.4438 0.1690 0.3037 0.216 0.091 0.470 48.18 0.606 0.554 198 204 -0.452941 0.107843

ENSMUST00000025057 636 0.2449 0.3741 0.1989 0.4788 0.234 -0.020 0.420 47.29 0.654 0.536 205 211 -0.742180 0.037915

ENSMUST00000024757 1371 0.3757 0.3439 0.2934 0.2343 0.238 0.060 0.447 54.73 0.456 0.444 432 456 -0.169298 0.100877

ENSMUST00000026156 999 0.2021 0.4251 0.1647 0.4125 0.270 0.295 0.575 47.35 0.691 0.599 320 332 -0.156325 0.075301

ENSMUST00000027503 639 0.2562 0.3625 0.2294 0.4172 0.285 0.195 0.524 60.24 0.612 0.536 206 212 -0.270283 0.075472

ENSMUST00000027824 675 0.3563 0.3276 0.3540 0.2434 0.202 0.007 0.413 53.28 0.441 0.440 213 224 -0.283036 0.147321

ENSMUST00000025409 1236 0.2210 0.4503 0.2021 0.3538 0.221 0.135 0.499 53.08 0.654 0.594 399 411 -0.787835 0.116788

ENSMUST00000023086 324 0.2396 0.4167 0.1395 0.3500 0.190 0.086 0.447 60.92 0.660 0.595 103 107 0.146729 0.084112

ENSMUST00000024774 606 0.1338 0.5141 0.1119 0.5970 0.283 0.107 0.484 38.11 0.814 0.575 188 201 -0.388557 0.109453

ENSMUST00000026274 900 0.3614 0.2525 0.4208 0.3040 0.208 -0.056 0.384 59.59 0.408 0.407 294 299 -0.728094 0.046823

ENSMUST00000029140 729 0.2147 0.4607 0.1639 0.4286 0.322 0.213 0.538 44.18 0.697 0.559 234 242 -0.111157 0.095041

ENSMUST00000027626 954 0.3537 0.2402 0.3110 0.3852 0.189 -0.067 0.382 56.46 0.482 0.463 309 317 -0.979811 0.066246

ENSMUST00000022567 3276 0.3008 0.3914 0.2450 0.3560 0.248 0.048 0.450 53.89 0.569 0.492 1053 1091 -0.220440 0.098992

ENSMUST00000024620 1644 0.3855 0.2651 0.3284 0.3133 0.225 0.017 0.436 54.45 0.440 0.458 523 547 -0.656124 0.076782

ENSMUST00000028157 1545 0.2621 0.4224 0.1984 0.4174 0.225 0.054 0.447 53.18 0.638 0.534 494 514 -0.739105 0.095331

ENSMUST00000028934 420 0.2157 0.3824 0.2778 0.4479 0.249 0.038 0.440 46.55 0.612 0.460 134 139 -0.046043 0.100719

ENSMUST00000027979 1260 0.3314 0.3284 0.2173 0.3737 0.236 0.057 0.446 56.16 0.552 0.525 404 419 -0.078520 0.097852

ENSMUST00000025396 1029 0.0961 0.4769 0.1649 0.4421 0.247 0.238 0.549 43.71 0.776 0.675 335 342 -0.575439 0.070175

ENSMUST00000028855 2508 0.2086 0.4426 0.1502 0.4295 0.247 0.139 0.481 47.11 0.704 0.577 811 835 0.040838 0.073054

ENSMUST00000028293 1149 0.2635 0.3547 0.3023 0.3436 0.217 0.065 0.460 56.02 0.548 0.513 374 382 -0.706806 0.054974

ENSMUST00000026322 4173 0.2711 0.4103 0.2164 0.3918 0.248 0.074 0.458 50.90 0.611 0.509 1346 1390 -0.239928 0.069784

ENSMUST00000025740 1170 0.2431 0.4554 0.1601 0.3711 0.272 0.110 0.477 48.23 0.667 0.574 384 389 -0.073522 0.066838

ENSMUST00000022998 2685 0.3519 0.2742 0.3305 0.3169 0.206 -0.009 0.413 54.59 0.458 0.469 871 894 -0.481208 0.071588

ENSMUST00000029297 1509 0.2797 0.3660 0.2774 0.2693 0.216 0.077 0.442 57.63 0.523 0.483 480 502 0.670120 0.121514

ENSMUST00000022791 1158 0.3257 0.2993 0.2734 0.3481 0.222 0.066 0.452 58.65 0.510 0.497 363 385 -0.316364 0.093506

ENSMUST00000027639 1557 0.2953 0.3176 0.3468 0.2580 0.200 0.017 0.442 55.41 0.468 0.557 498 518 -0.660039 0.042471

ENSMUST00000024981 573 0.3007 0.3007 0.3718 0.2467 0.188 0.007 0.433 59.63 0.444 0.514 187 190 -1.028.947 0.036842

ENSMUST00000024709 1914 0.3923 0.1834 0.4535 0.2394 0.205 -0.047 0.389 49.06 0.326 0.430 628 637 -0.791523 0.050235

ENSMUST00000026607 1989 0.4070 0.2192 0.4606 0.1937 0.216 -0.104 0.368 50.40 0.316 0.417 646 662 -0.360574 0.075529

ENSMUST00000025802 633 0.2171 0.3829 0.1637 0.4345 0.224 0.113 0.456 53.67 0.680 0.613 206 210 -0.107619 0.061905

ENSMUST00000029061 3204 0.2096 0.4731 0.1559 0.3842 0.224 0.076 0.463 49.12 0.698 0.598 1040 1067 -0.401125 0.035614

ENSMUST00000029385 201 0.2653 0.4898 0.1731 0.3333 0.186 -0.021 0.410 61.00 0.639 0.505 61 66 0.003030 0.060606

ENSMUST00000025595 1842 0.4179 0.2626 0.3870 0.2676 0.219 -0.058 0.401 50.15 0.387 0.403 594 613 -0.585318 0.089723

ENSMUST00000023339 480 0.3520 0.3440 0.3276 0.2566 0.185 -0.014 0.422 50.56 0.468 0.486 154 159 -0.942767 0.106918

ENSMUST00000029545 2079 0.2938 0.3656 0.2163 0.2855 0.192 0.051 0.433 53.31 0.558 0.576 674 692 -0.508381 0.046243

ENSMUST00000028049 2448 0.3946 0.2492 0.3588 0.2945 0.227 -0.035 0.402 51.41 0.411 0.438 786 815 -0.367853 0.098160

ENSMUST00000027797 2568 0.3920 0.2161 0.3712 0.2698 0.193 -0.023 0.397 54.55 0.381 0.458 837 855 -0.421637 0.043275

ENSMUST00000022345 780 0.1965 0.4220 0.3596 0.3698 0.261 0.095 0.486 52.72 0.574 0.497 251 259 -0.923166 0.073359

ENSMUST00000022287 1032 0.2776 0.4791 0.2149 0.3491 0.285 0.079 0.482 49.33 0.623 0.538 332 343 -0.482799 0.058309

ENSMUST00000025092 894 0.1724 0.4828 0.1518 0.3951 0.221 0.160 0.497 49.08 0.724 0.596 286 297 0.135017 0.097643

ENSMUST00000029803 654 0.4157 0.2530 0.3484 0.2847 0.231 0.053 0.456 57.72 0.403 0.444 206 217 -0.685254 0.101382

ENSMUST00000024704 420 0.2404 0.4135 0.1920 0.3577 0.219 0.000 0.419 46.25 0.640 0.607 136 139 -0.748201 0.007194

ENSMUST00000022641 1404 0.3696 0.2853 0.3275 0.3043 0.198 -0.040 0.396 52.77 0.449 0.455 452 467 -0.491221 0.079229

ENSMUST00000029201 3207 0.3329 0.3256 0.3400 0.3192 0.214 0.012 0.417 56.23 0.478 0.445 1012 1068 -0.311142 0.096442

ENSMUST00000026658 5703 0.2988 0.3938 0.2371 0.2640 0.229 0.084 0.480 53.06 0.548 0.562 1803 1900 -0.733053 0.048421

ENSMUST00000025110 1278 0.3183 0.3754 0.2644 0.3149 0.223 0.006 0.419 52.89 0.535 0.471 415 425 -0.316000 0.082353

ENSMUST00000024792 969 0.2206 0.3346 0.2519 0.3774 0.199 0.068 0.435 50.93 0.597 0.586 315 322 -0.119565 0.043478

ENSMUST00000026487 249 0.1912 0.3235 0.3077 0.3929 0.189 0.026 0.403 43.09 0.571 0.492 77 82 0.780488 0.073171

ENSMUST00000028259 3078 0.3381 0.3329 0.3477 0.2971 0.229 0.002 0.424 56.01 0.469 0.442 990 1025 -0.448683 0.067317

ENSMUST00000024627 5136 0.3844 0.2723 0.3554 0.3056 0.207 -0.022 0.421 53.96 0.431 0.448 1656 1711 -1.059.030 0.070134

ENSMUST00000022784 1092 0.2023 0.3852 0.1678 0.5155 0.237 0.088 0.453 52.34 0.709 0.562 351 363 -0.453719 0.057851

ENSMUST00000029030 645 0.2857 0.3257 0.2471 0.3353 0.207 0.044 0.444 49.78 0.551 0.589 207 214 -0.736916 0.051402

ENSMUST00000028111 807 0.2376 0.4158 0.3465 0.2760 0.202 0.012 0.431 54.43 0.537 0.496 255 268 -0.587687 0.074627

ENSMUST00000027103 2070 0.3917 0.2455 0.3327 0.3135 0.204 -0.037 0.383 53.13 0.421 0.418 660 689 -0.100435 0.076923

ENSMUST00000027706 2193 0.1948 0.4605 0.1693 0.3727 0.213 0.151 0.484 47.78 0.692 0.599 705 730 -0.100822 0.064384

ENSMUST00000023154 636 0.2443 0.4034 0.2025 0.3517 0.237 0.147 0.495 52.90 0.616 0.532 198 211 0.482938 0.104265

ENSMUST00000027202 3495 0.4386 0.2158 0.4009 0.2616 0.207 -0.106 0.376 50.36 0.355 0.399 1145 1164 -0.989175 0.036082

ENSMUST00000026677 873 0.3750 0.2857 0.3382 0.3109 0.228 0.005 0.421 52.69 0.446 0.424 278 290 -0.251379 0.127586

ENSMUST00000026259 909 0.1270 0.4960 0.1032 0.4480 0.243 0.121 0.488 36.75 0.803 0.689 295 302 -0.385430 0.079470

ENSMUST00000022461 249 0.3175 0.3810 0.3462 0.3830 0.256 -0.144 0.363 61.00 0.525 0.455 80 82 -0.350000 0.109756

ENSMUST00000028612 2163 0.2946 0.4108 0.2331 0.3222 0.244 0.094 0.476 54.59 0.574 0.521 695 720 -0.283195 0.087500

ENSMUST00000028177 1458 0.1883 0.5332 0.1437 0.4215 0.268 0.137 0.503 46.82 0.736 0.544 459 485 -0.466598 0.098969

ENSMUST00000025791 1944 0.2079 0.4740 0.1783 0.4706 0.282 0.131 0.494 46.44 0.703 0.546 620 647 -0.494590 0.098918

ENSMUST00000022316 420 0.3158 0.2737 0.3043 0.3909 0.249 0.028 0.448 50.19 0.515 0.516 134 139 -0.921583 0.057554

ENSMUST00000029805 2685 0.2925 0.3746 0.2514 0.3460 0.243 0.076 0.461 54.36 0.561 0.494 870 894 -0.161969 0.073826

ENSMUST00000029275 510 0.2602 0.3740 0.2901 0.3730 0.237 0.119 0.485 47.68 0.571 0.481 163 169 -0.343195 0.071006

ENSMUST00000027241 1731 0.3341 0.3057 0.3471 0.3306 0.202 -0.033 0.393 55.22 0.469 0.433 557 576 -0.222569 0.118056

ENSMUST00000023714 1617 0.2291 0.4654 0.1572 0.3942 0.243 0.107 0.494 47.96 0.685 0.571 524 538 -0.479926 0.066914

ENSMUST00000026398 735 0.1803 0.4590 0.2308 0.3977 0.257 0.243 0.546 43.87 0.672 0.549 229 244 -0.052869 0.110656

ENSMUST00000026387 753 0.1988 0.4152 0.2500 0.4780 0.273 0.091 0.471 48.88 0.653 0.499 242 250 -0.516800 0.052000

ENSMUST00000028667 2790 0.1992 0.4802 0.1608 0.4225 0.240 0.095 0.473 46.36 0.710 0.596 899 929 -0.584715 0.065662

ENSMUST00000027499 642 0.1813 0.3901 0.1453 0.4551 0.222 0.032 0.415 45.48 0.717 0.631 205 213 0.211737 0.084507

ENSMUST00000022820 849 0.2363 0.3629 0.1878 0.4131 0.236 0.108 0.469 47.57 0.637 0.552 273 282 0.210284 0.053191

ENSMUST00000024011 1509 0.2146 0.4419 0.2016 0.4067 0.246 0.065 0.459 48.72 0.663 0.541 484 502 -0.226295 0.111554

ENSMUST00000022121 816 0.3333 0.3134 0.3518 0.3109 0.232 0.002 0.435 57.57 0.473 0.485 260 271 -0.850923 0.055351

ENSMUST00000022311 1380 0.4077 0.2590 0.3137 0.2149 0.235 0.006 0.426 51.10 0.390 0.495 444 459 0.022876 0.071895

ENSMUST00000029164 780 0.2000 0.4233 0.2383 0.3155 0.180 0.056 0.432 51.44 0.624 0.584 250 259 -0.352124 0.069498

ENSMUST00000027057 1179 0.3322 0.2534 0.3600 0.3345 0.201 -0.007 0.422 55.38 0.452 0.462 374 392 -0.723214 0.061224

ENSMUST00000026360 2304 0.3746 0.3119 0.3610 0.2455 0.232 0.023 0.438 54.94 0.425 0.456 746 767 -0.204172 0.063885

ENSMUST00000029038 399 0.2526 0.3579 0.3689 0.3152 0.213 0.038 0.448 51.25 0.504 0.442 125 132 -0.284091 0.075758

ENSMUST00000023226 13632 0.1881 0.4349 0.1274 0.5220 0.280 0.126 0.491 42.41 0.752 0.615 4448 4543 -0.694101 0.047986

ENSMUST00000027198 1731 0.4315 0.2237 0.3643 0.2782 0.198 -0.022 0.404 48.10 0.379 0.407 564 576 -0.621007 0.071181

ENSMUST00000029172 312 0.0921 0.5658 0.1772 0.4306 0.212 0.188 0.516 37.57 0.779 0.566 95 103 -0.366990 0.077670

ENSMUST00000025253 6477 0.3163 0.3117 0.2648 0.2781 0.171 -0.029 0.388 53.05 0.503 0.606 2113 2158 -1.024.606 0.046803

ENSMUST00000029477 1428 0.2983 0.3591 0.2637 0.3463 0.248 0.103 0.480 55.65 0.547 0.498 450 475 -0.165474 0.098947

ENSMUST00000029213 1497 0.1962 0.4279 0.1699 0.3756 0.241 0.170 0.495 47.24 0.685 0.606 485 498 0.202008 0.080321

ENSMUST00000026479 1209 0.2804 0.3547 0.2222 0.4056 0.254 0.014 0.427 51.80 0.601 0.546 393 402 -0.475871 0.037313

ENSMUST00000024967 2808 0.3445 0.3053 0.2806 0.3608 0.244 0.046 0.448 57.88 0.506 0.472 907 935 -0.201604 0.077005

ENSMUST00000022380 1170 0.4007 0.1821 0.4267 0.2664 0.202 -0.030 0.390 49.84 0.340 0.418 377 389 -0.436504 0.056555

ENSMUST00000026011 969 0.2600 0.4200 0.1525 0.4099 0.219 0.021 0.421 49.61 0.660 0.546 297 322 0.133541 0.099379

ENSMUST00000028804 1731 0.2573 0.4228 0.1938 0.3906 0.255 0.139 0.496 50.32 0.637 0.553 557 576 -0.603993 0.072917

ENSMUST00000022437 1746 0.3427 0.3319 0.2624 0.3086 0.229 0.040 0.440 56.00 0.504 0.503 554 581 -0.046644 0.079174

ENSMUST00000022620 1539 0.1849 0.5304 0.1436 0.4043 0.280 0.167 0.503 45.02 0.732 0.555 477 512 0.134766 0.117188

ENSMUST00000027405 1836 0.2764 0.3247 0.1953 0.3388 0.196 0.117 0.454 51.19 0.578 0.575 588 611 0.447790 0.070376

ENSMUST00000025430 4749 0.2374 0.4048 0.2408 0.3610 0.222 0.048 0.452 50.76 0.610 0.540 1540 1582 -0.855752 0.045512

ENSMUST00000026601 2235 0.2911 0.2968 0.5471 0.1402 0.199 -0.108 0.373 45.56 0.336 0.457 687 744 -0.940726 0.083333

ENSMUST00000025250 3465 0.3266 0.3139 0.2096 0.3259 0.217 0.067 0.447 48.94 0.541 0.594 1117 1154 -0.361612 0.035529

ENSMUST00000026411 1584 0.3028 0.3638 0.2615 0.2989 0.219 0.023 0.431 54.19 0.537 0.532 499 527 -0.403985 0.117647

ENSMUST00000025932 1749 0.3174 0.3326 0.3072 0.3030 0.186 0.009 0.404 55.08 0.496 0.448 570 582 -0.343643 0.037801

ENSMUST00000022007 549 0.2030 0.4436 0.2621 0.4044 0.191 -0.015 0.413 54.48 0.637 0.542 179 182 -1.107.143 0.065934

ENSMUST00000024206 1023 0.2535 0.4542 0.1488 0.3867 0.287 0.121 0.500 48.14 0.667 0.559 324 340 -0.086765 0.082353

ENSMUST00000025208 1074 0.3345 0.3382 0.2896 0.3730 0.240 0.018 0.444 54.88 0.524 0.478 351 357 -0.871989 0.109244

ENSMUST00000029651 1905 0.3216 0.3278 0.3085 0.3203 0.234 0.036 0.433 53.31 0.498 0.476 616 634 -0.149685 0.069401

ENSMUST00000027601 2466 0.3364 0.3458 0.2808 0.3203 0.224 0.013 0.427 57.19 0.510 0.489 804 821 -0.414251 0.071864

ENSMUST00000028125 1404 0.3218 0.4109 0.2292 0.3583 0.270 0.051 0.468 53.98 0.577 0.508 447 467 -0.650321 0.083512

ENSMUST00000028248 2184 0.2169 0.4234 0.1717 0.4419 0.235 0.127 0.482 50.81 0.682 0.559 701 727 -0.395874 0.077029

ENSMUST00000029007 513 0.3095 0.3651 0.2114 0.4017 0.189 -0.029 0.399 56.48 0.589 0.500 158 170 -0.400000 0.100000

ENSMUST00000023614 2292 0.3766 0.3141 0.2857 0.2958 0.229 0.036 0.453 57.22 0.472 0.482 733 763 -0.698558 0.076016

ENSMUST00000023452 1626 0.2550 0.4094 0.2370 0.3139 0.222 0.106 0.471 49.99 0.593 0.555 526 541 -0.542514 0.055453

ENSMUST00000025681 4656 0.2188 0.3918 0.1707 0.4374 0.239 0.107 0.469 45.64 0.679 0.611 1515 1551 -0.439201 0.060606

ENSMUST00000023710 1575 0.2181 0.4951 0.1181 0.4410 0.289 0.178 0.531 40.91 0.730 0.573 512 524 -0.501336 0.059160

ENSMUST00000027032 6288 0.4494 0.2228 0.3896 0.2295 0.209 -0.052 0.400 50.96 0.344 0.405 2033 2095 -0.697136 0.062053

ENSMUST00000026292 13137 0.3731 0.3029 0.3076 0.2508 0.223 0.031 0.434 55.66 0.442 0.486 4242 4378 -0.384948 0.050480

ENSMUST00000028580 1104 0.3114 0.2982 0.3618 0.3581 0.256 0.074 0.476 59.34 0.490 0.484 355 367 -1.205.722 0.068120

ENSMUST00000022135 519 0.3858 0.2835 0.3710 0.3186 0.220 -0.039 0.395 55.48 0.431 0.440 167 172 -0.600000 0.098837

ENSMUST00000022185 1200 0.1994 0.5289 0.1448 0.3233 0.236 0.142 0.483 47.95 0.706 0.528 381 399 0.501002 0.117794

ENSMUST00000025045 411 0.1667 0.4896 0.1731 0.4706 0.242 0.100 0.481 45.95 0.736 0.581 129 136 -1.042.647 0.110294

ENSMUST00000023752 816 0.2101 0.4958 0.0734 0.3835 0.307 0.260 0.555 42.42 0.749 0.630 263 271 0.522140 0.088561

ENSMUST00000027185 1257 0.3545 0.2565 0.3103 0.3123 0.205 -0.006 0.407 54.76 0.452 0.472 405 418 -0.222010 0.090909

ENSMUST00000025840 1428 0.2237 0.4211 0.2279 0.3715 0.265 0.153 0.505 50.75 0.633 0.582 463 475 -0.207790 0.054737

ENSMUST00000029785 360 0.2222 0.3556 0.3258 0.4074 0.215 0.108 0.482 43.58 0.570 0.493 114 119 -0.615126 0.084034

ENSMUST00000024734 438 0.1870 0.5203 0.1478 0.3396 0.209 0.091 0.464 45.08 0.714 0.582 140 145 -0.206207 0.062069

ENSMUST00000024976 1170 0.2270 0.4785 0.1552 0.3558 0.270 0.151 0.514 47.29 0.682 0.578 368 389 -0.444216 0.087404

ENSMUST00000022921 1497 0.3306 0.3278 0.3686 0.3231 0.215 0.028 0.450 59.20 0.474 0.443 473 498 -0.671486 0.096386

ENSMUST00000027157 687 0.3296 0.3296 0.2577 0.3289 0.238 0.088 0.467 61.00 0.519 0.513 210 228 -0.112281 0.061404

ENSMUST00000029445 570 0.2905 0.3446 0.2826 0.3858 0.235 -0.041 0.401 46.99 0.549 0.489 182 189 -0.319048 0.079365

ENSMUST00000025675 714 0.2890 0.4046 0.2242 0.4177 0.249 0.008 0.444 52.55 0.610 0.519 223 237 -0.621097 0.101266

ENSMUST00000024988 4992 0.2002 0.4645 0.2177 0.4014 0.260 0.072 0.458 47.70 0.667 0.533 1606 1663 -0.257486 0.081780

ENSMUST00000023365 1353 0.2614 0.3977 0.2508 0.3560 0.211 0.095 0.458 51.83 0.590 0.502 424 450 -0.154445 0.133333

ENSMUST00000022682 2202 0.2571 0.4050 0.2364 0.3266 0.188 0.028 0.429 50.02 0.593 0.569 708 733 -0.933015 0.073670

ENSMUST00000029516 1917 0.3398 0.2316 0.4310 0.2831 0.204 -0.098 0.378 46.28 0.394 0.469 630 638 -1.070.376 0.034483

ENSMUST00000027783 585 0.4211 0.2697 0.3846 0.1970 0.235 -0.016 0.425 60.58 0.360 0.447 186 194 -0.160309 0.113402

ENSMUST00000023140 558 0.2800 0.3667 0.1957 0.4135 0.238 0.018 0.430 48.51 0.615 0.533 179 185 -0.272432 0.075676

ENSMUST00000023348 657 0.3256 0.2151 0.3988 0.3205 0.164 -0.095 0.352 52.91 0.414 0.422 210 218 0.027523 0.091743

ENSMUST00000023206 2754 0.2384 0.3712 0.2521 0.3924 0.238 0.114 0.474 52.66 0.602 0.532 898 917 -0.340240 0.071974

ENSMUST00000029815 408 0.2336 0.4393 0.2019 0.4021 0.207 -0.036 0.371 52.27 0.652 0.523 132 135 -0.062963 0.074074

ENSMUST00000029753 1677 0.2573 0.4676 0.1948 0.3407 0.230 0.131 0.487 49.49 0.638 0.588 544 558 -0.690323 0.062724

ENSMUST00000027817 7248 0.3335 0.3217 0.2912 0.4009 0.245 0.012 0.433 54.27 0.530 0.488 2336 2415 -0.767578 0.070807

ENSMUST00000027298 609 0.2431 0.4365 0.2157 0.2877 0.232 0.188 0.520 51.54 0.611 0.564 198 202 0.010396 0.064356

ENSMUST00000025196 831 0.2566 0.4292 0.1558 0.3770 0.229 0.110 0.492 51.84 0.655 0.570 258 276 -0.310870 0.101449

ENSMUST00000023672 597 0.2645 0.4387 0.2483 0.3529 0.214 0.001 0.425 52.09 0.601 0.500 193 198 -0.610606 0.111111

ENSMUST00000023343 945 0.3712 0.2314 0.4459 0.2864 0.253 -0.045 0.404 51.07 0.377 0.432 302 314 -0.498408 0.085987

ENSMUST00000026256 903 0.1412 0.4157 0.1551 0.4669 0.253 0.287 0.556 47.56 0.747 0.672 293 300 -0.035333 0.033333

ENSMUST00000029450 417 0.3577 0.3984 0.2222 0.2683 0.249 0.024 0.444 58.48 0.526 0.481 135 138 0.193478 0.130435

ENSMUST00000023362 933 0.3669 0.2782 0.3416 0.2667 0.228 0.028 0.426 52.33 0.426 0.475 303 310 -0.353871 0.061290

ENSMUST00000023144 156 0.0851 0.4681 0.3684 0.2703 0.072 0.196 0.520 NA 0.640 0.608 50 51 -2.250.980 0.058824

ENSMUST00000023203 1491 0.2308 0.4026 0.1675 0.4575 0.273 0.126 0.484 49.23 0.676 0.581 479 496 -0.154234 0.078629

ENSMUST00000024916 2103 0.2864 0.3881 0.2677 0.3025 0.232 0.077 0.455 54.33 0.547 0.480 682 700 0.211000 0.107143

ENSMUST00000024099 1845 0.2250 0.4212 0.1832 0.3697 0.211 0.083 0.453 46.90 0.656 0.594 587 614 -0.157980 0.114007

ENSMUST00000023353 2589 0.3504 0.3022 0.3049 0.2960 0.212 -0.006 0.411 56.20 0.467 0.480 839 862 -0.412297 0.060325

ENSMUST00000029848 5202 0.3247 0.2523 0.3933 0.2126 0.153 -0.067 0.380 52.40 0.388 0.538 1701 1733 -0.651125 0.048471

ENSMUST00000023328 318 0.3483 0.3933 0.1842 0.2676 0.212 0.023 0.434 52.65 0.545 0.540 99 105 0.295238 0.057143

ENSMUST00000029421 1146 0.2237 0.3586 0.1913 0.4437 0.220 0.070 0.453 51.77 0.653 0.590 360 381 -0.139370 0.068241

ENSMUST00000026573 1995 0.2441 0.4102 0.2173 0.3757 0.244 0.127 0.492 52.39 0.627 0.557 651 664 -0.630572 0.058735

ENSMUST00000025914 624 0.2367 0.4615 0.1919 0.2909 0.269 0.194 0.518 47.47 0.633 0.620 199 207 -0.150725 0.024155

ENSMUST00000027637 366 0.4082 0.2347 0.3778 0.2840 0.177 -0.134 0.325 61.00 0.383 0.405 120 121 -0.148760 0.099174

ENSMUST00000027215 678 0.4602 0.1648 0.4403 0.2361 0.230 -0.107 0.360 51.15 0.294 0.393 214 225 -0.164000 0.075556

ENSMUST00000022358 5499 0.2847 0.3666 0.2210 0.3240 0.222 0.028 0.432 51.45 0.574 0.582 1772 1832 -0.371998 0.066594

ENSMUST00000028783 1524 0.2086 0.5132 0.1940 0.3573 0.322 0.217 0.550 46.21 0.681 0.570 496 507 -0.340039 0.098619

ENSMUST00000022718 1329 0.2541 0.3867 0.2371 0.3691 0.208 0.071 0.448 54.19 0.595 0.496 420 442 0.246833 0.104072

ENSMUST00000028280 5517 0.3155 0.3538 0.2567 0.2601 0.194 0.039 0.440 52.36 0.514 0.613 1806 1838 -0.851850 0.048422

ENSMUST00000027263 1119 0.3771 0.2357 0.4021 0.2638 0.181 -0.070 0.376 55.47 0.378 0.422 362 372 -0.320699 0.075269

ENSMUST00000025541 1080 0.2158 0.4676 0.2461 0.3983 0.252 0.108 0.481 51.33 0.643 0.500 345 359 -0.447354 0.105850

ENSMUST00000022766 1860 0.3691 0.2680 0.2617 0.3176 0.228 -0.021 0.400 55.36 0.477 0.518 598 619 -0.387722 0.038772

ENSMUST00000025686 576 0.3247 0.3831 0.2687 0.3008 0.196 0.000 0.407 52.98 0.527 0.462 182 191 -0.021990 0.089005

ENSMUST00000022204 2118 0.3907 0.2206 0.4223 0.2515 0.197 -0.059 0.386 54.60 0.359 0.432 686 705 -0.632908 0.056738

ENSMUST00000029485 507 0.3088 0.4191 0.2339 0.3186 0.244 0.121 0.506 59.24 0.567 0.500 164 168 -0.800595 0.089286

ENSMUST00000028132 2184 0.1909 0.4314 0.1678 0.5070 0.244 0.125 0.479 47.97 0.720 0.570 701 727 -0.558184 0.037139

ENSMUST00000029483 1620 0.3610 0.2755 0.3249 0.3075 0.220 -0.030 0.398 50.74 0.451 0.477 512 539 -0.342301 0.094620

ENSMUST00000027650 1173 0.3404 0.2462 0.4214 0.2258 0.204 -0.009 0.406 57.48 0.377 0.456 382 390 -0.369231 0.071795

ENSMUST00000029465 1122 0.2669 0.3716 0.2772 0.3511 0.227 0.051 0.444 50.42 0.561 0.494 360 373 -0.167024 0.099196

ENSMUST00000022519 984 0.2634 0.4362 0.1992 0.3974 0.252 0.015 0.437 47.18 0.633 0.511 311 327 -0.320795 0.079511

ENSMUST00000029277 2679 0.3887 0.2169 0.3503 0.2745 0.181 -0.025 0.396 53.13 0.390 0.457 869 892 -0.252018 0.049327

ENSMUST00000024270 801 0.2899 0.3285 0.2987 0.2946 0.217 0.083 0.460 55.37 0.510 0.545 263 266 -0.847744 0.022556

ENSMUST00000028148 1764 0.2012 0.4295 0.1543 0.4348 0.253 0.196 0.522 46.83 0.703 0.599 565 587 -0.181431 0.071550

ENSMUST00000022228 1410 0.3593 0.2545 0.4016 0.3066 0.215 -0.056 0.401 53.88 0.418 0.451 459 469 -1.102.345 0.059701

ENSMUST00000027532 1299 0.2135 0.4017 0.1893 0.4286 0.205 0.045 0.438 44.35 0.665 0.574 418 432 -0.189120 0.050926

ENSMUST00000022331 1566 0.2067 0.4899 0.1858 0.2898 0.217 0.105 0.471 46.98 0.662 0.576 497 521 0.157390 0.105566

ENSMUST00000028309 1605 0.2027 0.4162 0.2251 0.4859 0.245 0.068 0.461 52.13 0.669 0.512 514 534 -0.710861 0.046816

ENSMUST00000025278 447 0.2520 0.3659 0.2101 0.3707 0.226 0.075 0.458 59.16 0.611 0.563 144 148 -0.200676 0.067568

ENSMUST00000022369 3201 0.3451 0.3035 0.2517 0.3501 0.234 0.031 0.437 54.86 0.516 0.524 1021 1066 -0.419231 0.030019

ENSMUST00000024866 4008 0.2791 0.4130 0.2049 0.3527 0.241 0.063 0.458 50.91 0.605 0.527 1287 1335 -0.166367 0.080150

ENSMUST00000028467 672 0.3575 0.3296 0.2882 0.2515 0.210 0.103 0.484 56.37 0.469 0.535 213 223 -0.394170 0.076233

ENSMUST00000028045 4371 0.3822 0.3058 0.3504 0.2596 0.244 -0.003 0.435 55.38 0.429 0.454 1367 1456 -0.495330 0.130495

ENSMUST00000026879 1077 0.3143 0.2893 0.3102 0.3514 0.181 -0.046 0.374 58.15 0.499 0.478 345 358 -0.388827 0.081006

ENSMUST00000029654 1491 0.3441 0.3441 0.2845 0.2943 0.216 -0.011 0.405 51.31 0.495 0.448 479 496 -0.051613 0.114919

ENSMUST00000029910 2763 0.3306 0.3127 0.3047 0.3409 0.219 -0.002 0.420 55.20 0.497 0.468 879 920 -0.324131 0.098913

ENSMUST00000027065 300 0.3596 0.2135 0.2593 0.2895 0.174 0.058 0.415 61.00 0.436 0.481 94 99 0.868687 0.090909

ENSMUST00000023036 327 0.3133 0.4217 0.1618 0.4286 0.315 0.091 0.495 61.00 0.626 0.537 99 108 -0.279630 0.101852

ENSMUST00000025421 1083 0.3818 0.2838 0.2980 0.2708 0.228 0.028 0.447 54.95 0.441 0.474 338 360 -0.366111 0.080556

ENSMUST00000026537 1515 0.2624 0.3639 0.2222 0.3826 0.246 0.087 0.465 52.56 0.601 0.559 486 504 -0.110119 0.091270

ENSMUST00000029632 696 0.2383 0.4249 0.2102 0.3602 0.239 0.107 0.466 51.07 0.628 0.517 223 231 0.131169 0.086580

ENSMUST00000027569 2121 0.4256 0.2145 0.4097 0.2205 0.193 -0.106 0.356 50.53 0.328 0.384 680 706 0.233428 0.114731

ENSMUST00000024518 555 0.2897 0.3310 0.2199 0.4361 0.241 0.039 0.436 53.63 0.592 0.514 179 184 -0.272826 0.097826

ENSMUST00000028694 1275 0.2343 0.4257 0.1497 0.4681 0.252 0.080 0.478 45.75 0.700 0.607 404 424 -0.975707 0.084906

ENSMUST00000026125 768 0.2186 0.4140 0.2365 0.3163 0.211 0.153 0.520 49.06 0.614 0.605 246 255 -0.853333 0.047059

ENSMUST00000026414 2193 0.2998 0.3968 0.2457 0.3536 0.252 0.088 0.470 51.43 0.569 0.503 692 730 -0.250822 0.091781

ENSMUST00000028836 1185 0.2375 0.4063 0.2383 0.3684 0.188 -0.000 0.408 54.22 0.615 0.541 382 394 -0.395178 0.076142

ENSMUST00000025836 579 0.3197 0.3469 0.1976 0.3882 0.234 -0.033 0.400 46.10 0.579 0.536 190 192 -0.134896 0.052083

ENSMUST00000024660 1344 0.2630 0.3931 0.2344 0.4037 0.226 0.006 0.432 49.69 0.612 0.546 438 447 -0.852349 0.060403

ENSMUST00000024957 660 0.3613 0.2565 0.3145 0.2778 0.211 0.020 0.404 46.86 0.428 0.423 208 219 0.709132 0.141553

ENSMUST00000028361 483 0.3115 0.3279 0.2951 0.3363 0.231 0.023 0.428 53.78 0.513 0.479 152 160 -0.156250 0.081250

ENSMUST00000023561 1767 0.3543 0.2848 0.3193 0.3140 0.187 -0.058 0.385 53.79 0.465 0.464 564 588 -0.695068 0.081633

ENSMUST00000029748 1215 0.2813 0.3456 0.2776 0.3256 0.197 0.002 0.414 51.61 0.540 0.507 391 404 -0.241832 0.096535

ENSMUST00000024575 2202 0.2216 0.4467 0.1779 0.4300 0.228 0.041 0.438 50.33 0.679 0.544 708 733 -0.324557 0.092769

ENSMUST00000024944 2058 0.2919 0.4036 0.2645 0.3160 0.220 0.059 0.457 56.61 0.557 0.490 654 685 -0.395036 0.116788

ENSMUST00000023906 522 0.3453 0.4101 0.2712 0.2368 0.327 0.178 0.537 54.18 0.512 0.487 164 173 -0.236416 0.138728

ENSMUST00000027066 1776 0.2857 0.3541 0.3333 0.2407 0.210 0.016 0.439 57.52 0.488 0.502 572 591 -0.481895 0.096447

ENSMUST00000023965 1032 0.4000 0.2321 0.4538 0.1911 0.177 -0.142 0.342 48.27 0.324 0.402 333 343 -0.323032 0.104956

ENSMUST00000027451 2961 0.2737 0.4175 0.2445 0.3204 0.250 0.109 0.486 53.81 0.580 0.514 945 986 -0.279412 0.087221

ENSMUST00000023448 1797 0.2484 0.3790 0.1945 0.4229 0.247 0.038 0.437 44.37 0.640 0.566 577 598 -0.240301 0.073579

ENSMUST00000029796 1677 0.3216 0.2770 0.3655 0.2916 0.223 0.008 0.412 51.71 0.443 0.461 539 558 0.101792 0.030466

ENSMUST00000023062 429 0.2079 0.3663 0.2437 0.3966 0.161 -0.093 0.353 52.81 0.624 0.580 133 142 -0.169718 0.063380

ENSMUST00000029908 1230 0.2603 0.4317 0.2042 0.3925 0.224 0.069 0.466 50.61 0.632 0.517 380 409 -0.373594 0.124694

ENSMUST00000028905 1914 0.2602 0.4136 0.2406 0.3473 0.243 0.057 0.463 49.98 0.598 0.534 605 637 -0.491680 0.094192

ENSMUST00000028984 720 0.2692 0.3681 0.2275 0.3736 0.226 0.112 0.464 56.50 0.589 0.531 224 239 -0.015063 0.054393

ENSMUST00000022612 993 0.4885 0.2023 0.3574 0.2454 0.243 -0.045 0.396 51.13 0.333 0.417 318 330 -0.350606 0.072727

ENSMUST00000029527 420 0.3577 0.5366 0.0971 0.1748 0.335 0.302 0.623 40.91 0.609 0.631 138 139 -0.446043 0.000000

ENSMUST00000029740 918 0.3164 0.3281 0.3594 0.2512 0.194 -0.031 0.414 57.35 0.461 0.505 295 305 -0.604918 0.091803

ENSMUST00000028683 1518 0.3769 0.3282 0.3127 0.2943 0.239 -0.046 0.403 57.21 0.468 0.457 494 505 -0.492475 0.100990

ENSMUST00000029240 1572 0.2989 0.3563 0.2655 0.2923 0.230 0.056 0.445 56.47 0.525 0.499 499 523 0.503250 0.120459

ENSMUST00000028769 2382 0.2565 0.4016 0.2787 0.3487 0.270 0.087 0.474 50.95 0.575 0.496 762 793 -0.366078 0.094578

ENSMUST00000024755 756 0.1872 0.4926 0.1758 0.4471 0.262 0.105 0.484 48.89 0.715 0.547 246 251 -0.545817 0.083665

ENSMUST00000025992 798 0.2258 0.4101 0.3131 0.3060 0.168 0.009 0.410 53.43 0.566 0.509 256 265 -0.420755 0.086792

ENSMUST00000029559 1383 0.1816 0.4421 0.2039 0.3818 0.249 0.147 0.490 51.49 0.679 0.571 445 460 -0.122609 0.078261

ENSMUST00000028128 1917 0.2222 0.3853 0.2520 0.4604 0.242 0.025 0.439 56.57 0.636 0.522 613 638 -0.881191 0.026646

ENSMUST00000023854 843 0.2374 0.5616 0.1243 0.4877 0.349 0.106 0.513 41.59 0.735 0.535 275 280 -0.480000 0.125000

ENSMUST00000022137 1668 0.2723 0.3683 0.2607 0.3737 0.223 0.046 0.443 55.86 0.574 0.511 530 555 -0.481261 0.113514

ENSMUST00000022707 1719 0.3239 0.3391 0.2536 0.3325 0.196 0.027 0.429 56.96 0.530 0.509 545 572 -0.284615 0.069930

ENSMUST00000023351 480 0.2197 0.3106 0.2143 0.4030 0.186 0.120 0.481 44.94 0.617 0.606 154 159 -0.129560 0.018868

ENSMUST00000022865 1740 0.3894 0.2644 0.3229 0.2922 0.232 0.011 0.439 57.76 0.437 0.484 561 579 -1.142.142 0.041451

ENSMUST00000027897 1302 0.1581 0.5228 0.1923 0.4505 0.277 0.154 0.512 45.64 0.731 0.556 416 433 -0.386836 0.090069

ENSMUST00000029257 3528 0.4278 0.2034 0.4075 0.2234 0.196 -0.061 0.378 49.23 0.326 0.403 1132 1175 0.019149 0.109787

ENSMUST00000024885 3159 0.3511 0.3085 0.3476 0.3410 0.242 -0.026 0.414 55.74 0.476 0.443 1015 1052 -0.698004 0.080798

ENSMUST00000026693 1926 0.3430 0.2975 0.3122 0.3438 0.226 -0.015 0.416 53.49 0.491 0.502 627 641 -0.842278 0.060842

ENSMUST00000027290 5634 0.2758 0.3656 0.3200 0.2610 0.197 0.001 0.420 56.28 0.508 0.495 1835 1877 -0.381300 0.075653

ENSMUST00000027981 1566 0.3359 0.3157 0.3010 0.3711 0.240 -0.017 0.415 55.73 0.508 0.477 504 521 -0.347025 0.086372

ENSMUST00000026236 1002 0.1649 0.5125 0.0996 0.4053 0.301 0.327 0.613 43.42 0.774 0.673 323 333 -0.430030 0.060060

ENSMUST00000023959 2619 0.2178 0.5232 0.0994 0.3558 0.264 0.114 0.480 41.63 0.728 0.607 846 872 0.081078 0.102064

ENSMUST00000025237 423 0.3063 0.3874 0.3776 0.2386 0.207 0.021 0.430 58.55 0.474 0.443 135 140 -0.270000 0.100000

ENSMUST00000023861 1164 0.3791 0.2960 0.3942 0.2720 0.246 -0.031 0.410 52.18 0.418 0.402 366 387 -0.457881 0.108527

ENSMUST00000028883 747 0.2500 0.4167 0.1481 0.4486 0.248 0.136 0.494 46.27 0.682 0.574 239 248 -0.589113 0.076613

ENSMUST00000026262 1587 0.2552 0.4269 0.1872 0.3927 0.261 0.130 0.491 53.49 0.642 0.532 503 528 -0.185227 0.142045

ENSMUST00000026552 1482 0.3241 0.3797 0.2500 0.3354 0.231 0.039 0.429 54.68 0.543 0.474 475 493 -0.155578 0.115619

ENSMUST00000022603 1005 0.2368 0.4323 0.2898 0.3080 0.239 0.132 0.497 47.54 0.579 0.518 318 334 -0.367665 0.089820

ENSMUST00000022469 4782 0.2369 0.4414 0.2114 0.3497 0.264 0.096 0.468 49.07 0.633 0.574 1565 1593 -0.231199 0.072191

ENSMUST00000027916 927 0.2632 0.3806 0.1967 0.4201 0.278 0.142 0.502 50.73 0.622 0.539 299 308 -0.028896 0.064935

ENSMUST00000028117 2148 0.4687 0.1485 0.4143 0.2239 0.183 -0.095 0.361 47.79 0.287 0.401 693 715 -0.316084 0.074126

ENSMUST00000022720 1287 0.3431 0.3138 0.2875 0.3299 0.236 0.055 0.440 58.36 0.496 0.476 411 428 -0.186449 0.081776

ENSMUST00000029888 918 0.4035 0.2368 0.3655 0.2748 0.229 -0.020 0.412 55.91 0.391 0.444 294 305 -0.344918 0.118033

ENSMUST00000027863 915 0.2456 0.4868 0.1818 0.4394 0.263 0.128 0.497 49.61 0.673 0.507 294 304 -0.549671 0.131579

ENSMUST00000028348 2364 0.3163 0.3969 0.2862 0.2863 0.254 0.076 0.469 54.46 0.526 0.495 768 787 -0.227446 0.060991

ENSMUST00000023074 996 0.1714 0.4571 0.1901 0.4840 0.275 0.127 0.477 44.16 0.717 0.542 325 331 -0.229003 0.066465

ENSMUST00000028730 1713 0.3576 0.2682 0.3533 0.2324 0.188 -0.060 0.372 54.45 0.408 0.450 559 570 0.143684 0.054386

ENSMUST00000027743 768 0.2032 0.4011 0.2537 0.4171 0.232 0.104 0.475 48.47 0.632 0.528 242 255 -0.318823 0.058824

ENSMUST00000026551 1719 0.2447 0.4404 0.1878 0.3619 0.260 0.111 0.485 49.51 0.642 0.565 553 572 -0.179021 0.064685

ENSMUST00000024572 1170 0.2544 0.3380 0.3045 0.3741 0.175 -0.010 0.400 54.15 0.552 0.521 375 389 -0.766581 0.056555

ENSMUST00000029005 924 0.2271 0.4148 0.1852 0.4675 0.252 0.052 0.463 48.80 0.677 0.556 300 307 -0.734853 0.048860

ENSMUST00000028592 1125 0.3688 0.2908 0.3262 0.3180 0.233 -0.024 0.400 53.71 0.458 0.442 360 374 -0.187166 0.072193

ENSMUST00000022550 2760 0.2453 0.4544 0.1407 0.4209 0.244 0.085 0.459 45.63 0.685 0.559 883 919 -0.215560 0.103373

ENSMUST00000025505 1383 0.3011 0.3722 0.2761 0.3314 0.241 0.066 0.450 54.07 0.544 0.509 447 460 -0.490435 0.067391

ENSMUST00000023554 1437 0.2477 0.3341 0.1909 0.3840 0.170 0.084 0.438 55.55 0.610 0.561 454 478 0.726360 0.121339

ENSMUST00000028282 2181 0.2361 0.4050 0.2268 0.4034 0.217 -0.067 0.387 47.60 0.631 0.551 705 726 -0.880441 0.044077

ENSMUST00000023486 2292 0.4481 0.2339 0.3602 0.2303 0.229 -0.022 0.412 49.60 0.356 0.417 735 763 -0.259633 0.110092

ENSMUST00000027266 462 0.3985 0.2406 0.3028 0.2755 0.226 0.059 0.434 59.30 0.407 0.436 145 153 0.329412 0.130719

ENSMUST00000023911 1554 0.1891 0.4624 0.1429 0.4328 0.252 0.172 0.520 44.20 0.725 0.625 502 517 -0.286074 0.067698

ENSMUST00000022538 309 0.1600 0.5600 0.1282 0.4306 0.263 0.136 0.505 46.49 0.768 0.608 95 102 -0.623529 0.098039

ENSMUST00000025020 1401 0.2179 0.4413 0.1565 0.4512 0.244 0.111 0.477 47.00 0.699 0.577 438 466 -0.483047 0.098712

ENSMUST00000025704 795 0.3095 0.3000 0.2434 0.3502 0.187 -0.011 0.398 54.02 0.537 0.528 259 264 -0.539015 0.045455

ENSMUST00000029380 1188 0.2104 0.4628 0.1860 0.3754 0.216 0.110 0.481 51.08 0.676 0.581 374 395 -0.713924 0.048101

ENSMUST00000024832 906 0.2350 0.4378 0.3028 0.3886 0.241 0.032 0.466 59.86 0.602 0.537 294 301 -1.279.402 0.109635

ENSMUST00000022694 1152 0.2027 0.4502 0.1906 0.3974 0.216 0.098 0.472 47.70 0.677 0.564 371 383 -0.829504 0.057441

ENSMUST00000029490 1593 0.3333 0.3478 0.2259 0.3733 0.261 0.020 0.437 56.74 0.553 0.504 508 530 -0.265283 0.077358

ENSMUST00000025007 561 0.1806 0.4194 0.1724 0.4265 0.183 0.084 0.455 46.90 0.699 0.604 176 186 -0.129032 0.080645

ENSMUST00000022586 1455 0.3661 0.2486 0.3333 0.3203 0.211 -0.010 0.416 54.07 0.444 0.491 464 484 -0.906612 0.074380

ENSMUST00000029865 720 0.4167 0.3500 0.3216 0.2086 0.275 0.068 0.476 54.74 0.427 0.471 227 239 -0.594142 0.083682

ENSMUST00000024778 639 0.2667 0.4000 0.1761 0.4183 0.258 -0.023 0.411 50.65 0.644 0.550 202 212 0.025000 0.094340

ENSMUST00000027285 780 0.2857 0.3304 0.2616 0.3836 0.203 -0.017 0.389 49.83 0.553 0.462 244 259 0.421236 0.193050

ENSMUST00000026839 957 0.3626 0.3473 0.2231 0.3190 0.275 0.095 0.472 52.59 0.515 0.485 307 318 0.062264 0.050314

ENSMUST00000023918 1929 0.4366 0.2281 0.3274 0.2864 0.211 -0.084 0.374 53.52 0.392 0.445 610 642 -0.352181 0.088785

ENSMUST00000024849 3225 0.2237 0.3905 0.2131 0.4187 0.222 0.051 0.445 50.82 0.645 0.570 1046 1074 -0.550186 0.054935

ENSMUST00000025891 2142 0.2111 0.4919 0.1445 0.4507 0.285 0.191 0.531 44.22 0.718 0.554 682 713 -0.385274 0.116410

ENSMUST00000024905 1947 0.2488 0.3920 0.2528 0.4414 0.232 0.026 0.435 53.71 0.617 0.517 630 648 -0.944290 0.044753

ENSMUST00000024816 2514 0.2791 0.4279 0.1842 0.4073 0.246 0.054 0.452 49.76 0.635 0.523 801 837 -0.465950 0.100358

ENSMUST00000022343 1059 0.2796 0.3154 0.2242 0.4222 0.231 0.073 0.458 52.17 0.589 0.533 343 352 -0.155398 0.088068

ENSMUST00000027139 2493 0.3531 0.2892 0.3671 0.2895 0.233 -0.000 0.424 54.40 0.438 0.441 806 830 -0.323253 0.098795

ENSMUST00000022857 1377 0.3145 0.3501 0.3175 0.3222 0.265 0.124 0.502 51.28 0.505 0.462 444 458 -0.350000 0.076419

ENSMUST00000027421 6078 0.3930 0.2201 0.3770 0.2533 0.186 -0.084 0.371 53.04 0.374 0.451 1966 2025 -0.476642 0.063210

ENSMUST00000025402 1347 0.2083 0.4306 0.2440 0.4153 0.249 0.150 0.505 50.70 0.642 0.535 436 448 -0.462277 0.087054

ENSMUST00000025065 1389 0.3549 0.2845 0.3380 0.2832 0.188 -0.049 0.386 55.30 0.445 0.483 443 462 -0.346537 0.077922

ENSMUST00000029769 825 0.2823 0.3493 0.2685 0.3582 0.258 0.164 0.508 58.14 0.553 0.504 262 274 -0.275548 0.054745

ENSMUST00000025243 2301 0.3978 0.2810 0.3524 0.2875 0.208 -0.106 0.382 52.92 0.428 0.477 746 766 -1.373.238 0.027415

ENSMUST00000022849 2169 0.2871 0.3846 0.2762 0.3873 0.231 0.031 0.445 57.13 0.569 0.494 692 722 -0.645706 0.102493

ENSMUST00000029565 666 0.2320 0.4845 0.1000 0.3648 0.252 0.179 0.500 48.26 0.710 0.560 214 221 0.449774 0.117647

ENSMUST00000026014 2253 0.3808 0.2815 0.4000 0.2747 0.196 -0.079 0.374 55.79 0.406 0.411 727 750 -0.466667 0.141333

ENSMUST00000028102 2871 0.2601 0.3961 0.2069 0.4753 0.254 0.035 0.446 51.48 0.645 0.512 933 956 -0.799791 0.040795

ENSMUST00000026506 726 0.3155 0.2888 0.3446 0.3353 0.211 -0.097 0.375 61.00 0.483 0.508 232 241 -0.641079 0.078838

ENSMUST00000022186 300 0.2273 0.4394 0.2308 0.5161 0.289 0.062 0.462 45.33 0.670 0.485 91 99 -0.444445 0.080808

ENSMUST00000022638 2547 0.1644 0.5203 0.2087 0.4652 0.317 0.095 0.496 44.06 0.714 0.561 836 848 -1.183.844 0.035377

ENSMUST00000028121 1419 0.4323 0.2161 0.3770 0.2514 0.200 -0.054 0.409 48.09 0.360 0.448 450 472 -0.833687 0.072034

ENSMUST00000028336 681 0.2775 0.3717 0.1919 0.3519 0.198 0.101 0.472 58.63 0.598 0.563 214 226 -0.003982 0.106195

ENSMUST00000023146 963 0.2299 0.4330 0.2065 0.3805 0.255 0.089 0.471 49.92 0.642 0.572 310 320 -0.142188 0.050000

ENSMUST00000025719 342 0.3226 0.3656 0.2584 0.2840 0.196 0.057 0.445 59.66 0.518 0.525 110 113 -0.700885 0.070796

ENSMUST00000029128 366 0.1333 0.6667 0.0889 0.4512 0.324 0.259 0.564 38.98 0.829 0.576 117 121 -0.522314 0.099174

ENSMUST00000027393 2298 0.3552 0.2881 0.2876 0.3209 0.212 -0.003 0.412 56.48 0.479 0.485 743 765 -0.448758 0.047059

ENSMUST00000025490 1332 0.2946 0.3324 0.1833 0.3551 0.241 0.124 0.482 50.18 0.584 0.581 425 443 -0.100903 0.058691

ENSMUST00000026018 570 0.3438 0.4125 0.2344 0.2613 0.299 0.126 0.494 54.05 0.528 0.460 180 189 0.011640 0.111111

ENSMUST00000028702 882 0.3913 0.2913 0.3226 0.2886 0.236 0.059 0.453 50.96 0.439 0.438 285 293 -0.291126 0.092150

ENSMUST00000027492 1041 0.3347 0.3145 0.2338 0.4151 0.186 -0.006 0.402 55.02 0.560 0.494 336 346 -0.545376 0.066474

ENSMUST00000027775 495 0.3750 0.2250 0.4309 0.2909 0.208 -0.076 0.382 56.90 0.376 0.429 157 164 -0.467683 0.079268

ENSMUST00000024939 699 0.3602 0.3710 0.2338 0.3490 0.267 0.032 0.455 54.98 0.540 0.497 224 232 -0.775862 0.086207

ENSMUST00000026740 8835 0.3169 0.2787 0.2557 0.2962 0.168 -0.006 0.405 53.30 0.498 0.617 2910 2944 -0.621366 0.032609

ENSMUST00000026218 1614 0.3548 0.3381 0.2366 0.3634 0.235 0.010 0.427 55.46 0.535 0.502 529 537 -0.415270 0.096834

ENSMUST00000024705 969 0.2480 0.3425 0.3320 0.2815 0.168 -0.016 0.395 61.00 0.512 0.517 301 322 -0.034472 0.090062

ENSMUST00000022019 435 0.2101 0.3950 0.2752 0.3654 0.236 0.130 0.486 52.02 0.607 0.498 140 144 -0.126389 0.062500

ENSMUST00000023694 873 0.2296 0.3418 0.2348 0.4977 0.225 0.032 0.436 51.96 0.640 0.533 275 290 -0.670690 0.058621

ENSMUST00000029139 1638 0.3158 0.3409 0.2883 0.3537 0.271 0.053 0.460 52.45 0.527 0.506 533 545 -0.535780 0.053211

ENSMUST00000028780 672 0.2692 0.4231 0.1977 0.3274 0.269 0.122 0.484 45.92 0.614 0.587 215 223 -0.334529 0.107623

ENSMUST00000025069 1038 0.4160 0.1794 0.4054 0.2130 0.225 -0.015 0.415 53.59 0.316 0.463 335 345 -0.486957 0.028986

ENSMUST00000023231 966 0.1992 0.4981 0.1853 0.3953 0.249 0.038 0.448 48.08 0.694 0.548 310 321 -0.257632 0.109034

ENSMUST00000027015 1209 0.3808 0.2417 0.3920 0.3004 0.226 -0.038 0.402 51.20 0.399 0.419 388 402 -0.341542 0.077114

ENSMUST00000027472 723 0.1808 0.4802 0.1295 0.4894 0.257 0.106 0.483 46.08 0.756 0.611 234 240 -0.606667 0.070833

ENSMUST00000025476 870 0.4241 0.2455 0.3544 0.2953 0.242 -0.039 0.407 57.70 0.400 0.438 280 289 -0.444291 0.089965

ENSMUST00000026826 846 0.1897 0.5603 0.1190 0.3646 0.279 0.223 0.546 42.66 0.743 0.574 269 281 -0.262278 0.085409

ENSMUST00000025563 549 0.3162 0.3897 0.2177 0.4407 0.348 0.174 0.531 47.31 0.600 0.502 175 182 -0.732418 0.087912

ENSMUST00000027367 786 0.1719 0.4887 0.1667 0.4286 0.207 0.039 0.426 45.35 0.727 0.591 256 261 -0.214176 0.080460

ENSMUST00000029692 660 0.2588 0.4118 0.2455 0.3636 0.231 0.055 0.455 51.13 0.597 0.525 211 219 -0.545662 0.091324

ENSMUST00000029400 2253 0.4128 0.2763 0.3686 0.2594 0.199 -0.075 0.386 54.69 0.396 0.415 718 750 -0.478000 0.105333

ENSMUST00000022124 1986 0.3254 0.3857 0.2833 0.2808 0.212 0.027 0.424 55.46 0.515 0.443 648 661 0.034947 0.083207

ENSMUST00000028059 834 0.2130 0.4491 0.2286 0.4061 0.278 0.218 0.531 56.21 0.653 0.499 271 277 -0.454874 0.064982

ENSMUST00000028910 1017 0.3047 0.3047 0.3027 0.3775 0.239 0.011 0.422 57.95 0.523 0.489 329 338 -0.468343 0.094675

ENSMUST00000023813 1098 0.3174 0.4027 0.1633 0.3230 0.261 0.106 0.480 52.48 0.599 0.580 354 365 -0.302740 0.041096

ENSMUST00000026750 3099 0.3575 0.2774 0.3911 0.2592 0.213 -0.015 0.413 53.92 0.410 0.446 997 1032 -0.734012 0.072674

ENSMUST00000025698 387 0.2143 0.4732 0.1368 0.3736 0.275 0.162 0.508 48.81 0.702 0.602 124 128 0.060156 0.062500

ENSMUST00000023750 954 0.2299 0.4781 0.1250 0.3756 0.275 0.171 0.510 46.97 0.699 0.559 306 317 0.411672 0.154574

ENSMUST00000029092 1245 0.3079 0.3810 0.1942 0.3816 0.256 0.022 0.463 53.68 0.601 0.548 393 414 -0.860870 0.101449

ENSMUST00000028243 960 0.3429 0.3347 0.4419 0.2030 0.210 0.039 0.442 52.93 0.405 0.401 301 319 -0.359248 0.159875

ENSMUST00000026865 2505 0.2704 0.3821 0.2179 0.4148 0.223 0.030 0.441 50.89 0.615 0.534 807 834 -0.716067 0.063549

ENSMUST00000025027 534 0.2276 0.3517 0.2333 0.3427 0.217 0.085 0.440 53.07 0.595 0.578 168 177 0.271187 0.062147

ENSMUST00000025582 744 0.3463 0.3171 0.2624 0.2717 0.248 0.069 0.448 53.30 0.481 0.460 239 247 0.567207 0.072874

ENSMUST00000022871 609 0.3418 0.3038 0.3171 0.2781 0.256 0.006 0.429 52.85 0.459 0.482 196 202 -0.294555 0.069307

ENSMUST00000026175 930 0.2438 0.3884 0.1659 0.4749 0.209 0.001 0.420 50.19 0.671 0.561 295 309 -0.367961 0.113269

ENSMUST00000023180 2304 0.2766 0.3351 0.3149 0.3463 0.206 0.004 0.425 54.06 0.530 0.512 744 767 -0.788527 0.052151

ENSMUST00000023718 1440 0.2160 0.4933 0.1117 0.4598 0.276 0.113 0.495 40.34 0.739 0.595 467 479 -0.409603 0.056367

ENSMUST00000022609 1701 0.3209 0.2967 0.2698 0.3722 0.194 0.004 0.415 53.33 0.518 0.500 544 566 -0.353887 0.088339

ENSMUST00000029772 1977 0.3067 0.2762 0.3062 0.2966 0.167 -0.061 0.371 53.39 0.478 0.554 647 658 -0.604863 0.031915

ENSMUST00000028758 1380 0.1989 0.4409 0.1432 0.4270 0.233 0.123 0.483 46.04 0.713 0.624 443 459 -0.545534 0.058824

ENSMUST00000028251 573 0.3623 0.3478 0.3154 0.2766 0.249 0.010 0.429 49.59 0.473 0.467 184 190 -0.645790 0.089474

ENSMUST00000028807 1275 0.2690 0.4181 0.1877 0.3721 0.222 0.058 0.449 52.20 0.625 0.546 408 424 -0.108019 0.073113

ENSMUST00000026922 1065 0.1673 0.4861 0.1886 0.4830 0.252 0.094 0.490 50.66 0.725 0.541 345 354 -0.935593 0.059322

ENSMUST00000023043 1455 0.2414 0.3846 0.2077 0.4441 0.271 0.161 0.510 49.46 0.639 0.549 463 484 -0.251653 0.072314

ENSMUST00000023760 1050 0.2065 0.4638 0.1287 0.4816 0.259 0.059 0.453 45.27 0.728 0.572 338 349 0.102292 0.060172

ENSMUST00000026270 1764 0.4046 0.2243 0.3844 0.2732 0.191 -0.113 0.353 52.82 0.374 0.416 561 587 -0.126576 0.110733

ENSMUST00000023390 1341 0.2145 0.4393 0.2018 0.3505 0.208 0.080 0.449 49.12 0.649 0.545 430 446 0.234080 0.091928

ENSMUST00000023269 474 0.2844 0.3486 0.2910 0.3543 0.196 -0.056 0.405 54.87 0.542 0.510 153 157 -0.926115 0.063694

ENSMUST00000023387 1038 0.2527 0.3553 0.3475 0.3320 0.231 0.024 0.429 53.85 0.527 0.494 336 345 -0.125217 0.072464

ENSMUST00000027943 357 0.1687 0.3614 0.1134 0.6250 0.208 0.083 0.452 34.31 0.783 0.607 115 118 -1.092.373 0.008475

ENSMUST00000026378 1989 0.1996 0.4828 0.1278 0.4322 0.256 0.107 0.471 43.11 0.731 0.580 643 662 -0.194411 0.095166

ENSMUST00000025391 1719 0.4048 0.2516 0.3373 0.2846 0.186 -0.061 0.379 55.59 0.408 0.426 549 572 -0.383566 0.101399

ENSMUST00000023769 810 0.3077 0.2933 0.2838 0.3395 0.231 0.089 0.479 58.20 0.510 0.502 263 269 -0.642008 0.026022

ENSMUST00000022915 1560 0.2458 0.3747 0.2853 0.3425 0.236 0.028 0.440 54.00 0.568 0.536 495 519 -0.223700 0.077071

ENSMUST00000023432 831 0.3677 0.2870 0.2870 0.3166 0.227 -0.027 0.406 50.97 0.469 0.486 271 276 -0.223913 0.094203

ENSMUST00000023602 687 0.3410 0.3468 0.3258 0.2575 0.243 0.069 0.450 52.18 0.468 0.452 220 228 -0.183772 0.057018

ENSMUST00000026148 735 0.2000 0.4390 0.1771 0.3736 0.250 0.110 0.472 44.60 0.678 0.564 233 244 0.179918 0.049180

ENSMUST00000023781 516 0.2713 0.4186 0.1357 0.4015 0.256 0.172 0.521 46.31 0.669 0.606 163 171 -0.281287 0.029240

ENSMUST00000027338 315 0.3553 0.2237 0.3373 0.3293 0.185 -0.100 0.354 59.76 0.444 0.500 99 104 -1.017.308 0.076923

ENSMUST00000026162 579 0.2250 0.4875 0.1831 0.3520 0.277 0.206 0.533 46.33 0.663 0.526 184 192 -0.050000 0.093750

ENSMUST00000029626 831 0.2018 0.4574 0.2368 0.4246 0.290 0.171 0.522 49.43 0.664 0.525 268 276 -0.351087 0.105072

ENSMUST00000026241 807 0.1038 0.5708 0.1327 0.4390 0.254 0.222 0.548 41.39 0.808 0.626 261 268 -0.814925 0.074627

ENSMUST00000025048 2118 0.1899 0.4792 0.1752 0.4383 0.247 0.139 0.497 50.86 0.705 0.537 668 705 -0.178723 0.086525

ENSMUST00000029549 747 0.3333 0.2933 0.2417 0.4824 0.311 0.113 0.494 53.98 0.581 0.522 241 248 -1.027.016 0.016129

ENSMUST00000023799 1596 0.2209 0.4563 0.1375 0.4331 0.270 0.152 0.519 46.22 0.709 0.575 516 531 -0.538230 0.058380

ENSMUST00000026558 1593 0.2243 0.4010 0.1990 0.4129 0.201 0.020 0.412 49.97 0.655 0.569 510 530 -0.269434 0.052830

ENSMUST00000029142 738 0.2153 0.4545 0.1344 0.4310 0.228 0.030 0.429 51.73 0.708 0.570 240 245 0.108572 0.053061

ENSMUST00000024119 1443 0.2995 0.3454 0.2032 0.3462 0.213 0.052 0.443 50.64 0.572 0.610 470 480 -0.382708 0.060417

ENSMUST00000022386 2136 0.2317 0.4528 0.2290 0.3184 0.243 0.154 0.507 51.62 0.622 0.554 682 711 -0.509142 0.052039

ENSMUST00000025266 609 0.2210 0.4917 0.1437 0.2968 0.221 0.160 0.490 49.95 0.682 0.587 198 202 0.261386 0.108911

ENSMUST00000025774 2637 0.2954 0.3135 0.2387 0.4251 0.219 -0.031 0.397 53.67 0.580 0.555 841 878 -0.960137 0.050114

ENSMUST00000023593 744 0.3317 0.3894 0.2458 0.2717 0.231 0.015 0.432 54.90 0.531 0.526 241 247 -0.381781 0.109312

ENSMUST00000023572 1098 0.2949 0.3356 0.2650 0.3550 0.228 0.079 0.463 56.09 0.542 0.508 354 365 -0.284658 0.068493

ENSMUST00000027725 1707 0.3758 0.2851 0.2637 0.3240 0.212 -0.064 0.379 53.63 0.476 0.494 544 568 -0.161268 0.079225

ENSMUST00000029459 1497 0.3275 0.3176 0.3079 0.3324 0.236 0.014 0.425 56.74 0.497 0.475 487 498 -0.307430 0.100402

ENSMUST00000025354 1134 0.2628 0.3750 0.2424 0.3959 0.223 0.095 0.478 55.81 0.594 0.516 360 377 -0.292042 0.095491

ENSMUST00000026196 1242 0.2760 0.4214 0.1830 0.3693 0.237 0.086 0.466 51.89 0.625 0.542 397 413 -0.254237 0.106538

ENSMUST00000028880 2046 0.3471 0.3399 0.2233 0.3027 0.257 0.092 0.466 56.64 0.519 0.496 644 681 0.228781 0.088106

ENSMUST00000027356 1602 0.2423 0.3777 0.1995 0.4322 0.226 0.076 0.448 50.08 0.639 0.546 513 533 -0.247092 0.097561

ENSMUST00000025215 1398 0.2353 0.3971 0.1947 0.4533 0.269 0.096 0.466 50.69 0.662 0.546 453 465 -0.322150 0.062366

ENSMUST00000024015 351 0.2796 0.3441 0.2083 0.3978 0.215 -0.031 0.374 58.60 0.600 0.569 115 116 0.209483 0.025862

ENSMUST00000027131 2502 0.3851 0.3194 0.3327 0.2838 0.248 0.011 0.433 58.25 0.448 0.459 811 833 -0.521369 0.066026

ENSMUST00000028340 444 0.3077 0.4154 0.1624 0.2523 0.197 0.064 0.418 52.16 0.582 0.599 141 147 0.387755 0.027211

ENSMUST00000022511 4131 0.4426 0.2363 0.4118 0.2184 0.224 -0.060 0.397 51.47 0.342 0.413 1329 1376 -0.610393 0.080669

ENSMUST00000028239 2343 0.2520 0.4569 0.1559 0.3817 0.277 0.145 0.507 44.85 0.668 0.581 751 780 -0.446667 0.089744

ENSMUST00000022147 867 0.3493 0.3188 0.3519 0.2233 0.241 0.073 0.469 52.33 0.433 0.495 275 288 -0.775347 0.076389

ENSMUST00000022553 1014 0.3133 0.3414 0.3202 0.3761 0.264 0.060 0.450 53.15 0.517 0.430 329 337 -0.288131 0.089021

ENSMUST00000029382 1113 0.4265 0.2079 0.4036 0.2778 0.225 -0.079 0.388 54.72 0.358 0.423 358 370 -0.432703 0.078378

ENSMUST00000027251 3750 0.3894 0.2345 0.3459 0.2799 0.211 -0.012 0.414 53.39 0.404 0.461 1212 1249 -0.485268 0.056045

ENSMUST00000023924 369 0.2828 0.3232 0.2917 0.3333 0.200 -0.027 0.387 61.00 0.529 0.481 119 122 0.239344 0.106557

ENSMUST00000023687 999 0.2193 0.3717 0.2795 0.3950 0.241 0.080 0.451 52.93 0.599 0.521 324 332 -0.265060 0.102410

ENSMUST00000027090 528 0.2286 0.5786 0.1869 0.2970 0.274 0.285 0.595 53.74 0.681 0.537 163 175 -0.714857 0.171429

ENSMUST00000026989 912 0.3191 0.3447 0.2823 0.2614 0.226 -0.014 0.412 54.90 0.498 0.548 289 303 -0.725083 0.049505

ENSMUST00000026409 462 0.2406 0.4586 0.1802 0.3300 0.230 0.133 0.473 50.83 0.644 0.510 146 153 0.462745 0.130719

ENSMUST00000023759 1548 0.2455 0.3850 0.1960 0.4536 0.241 0.129 0.492 52.00 0.648 0.561 494 515 -0.630680 0.071845

ENSMUST00000026172 987 0.2213 0.3648 0.2148 0.4553 0.214 0.022 0.421 46.85 0.648 0.580 318 328 -0.616768 0.024390

ENSMUST00000029667 2802 0.2674 0.3827 0.2620 0.3003 0.222 0.060 0.453 54.69 0.557 0.528 905 933 -0.246195 0.075027

ENSMUST00000028748 2130 0.2546 0.4705 0.2107 0.3973 0.272 0.095 0.483 50.90 0.643 0.519 673 709 -0.485755 0.118477

ENSMUST00000023467 1575 0.3570 0.2835 0.3309 0.3029 0.243 0.057 0.451 51.58 0.451 0.459 506 524 -0.446565 0.059160

ENSMUST00000022945 2007 0.4512 0.2285 0.3585 0.2907 0.215 -0.063 0.387 54.39 0.376 0.410 644 668 -0.298353 0.091317

ENSMUST00000028608 3075 0.3125 0.3463 0.1915 0.4189 0.234 0.057 0.439 51.96 0.595 0.515 995 1024 -0.207910 0.072266

ENSMUST00000027121 1503 0.2802 0.3393 0.3111 0.3253 0.206 0.012 0.432 52.77 0.525 0.520 484 500 -0.498000 0.076000

ENSMUST00000026681 666 0.3595 0.2484 0.3931 0.3272 0.211 0.002 0.425 54.80 0.425 0.434 214 221 -1.090.498 0.054299

ENSMUST00000028633 8622 0.3256 0.4269 0.2570 0.3043 0.264 0.067 0.478 53.35 0.551 0.517 2808 2873 -0.423042 0.066829

ENSMUST00000028915 561 0.2624 0.3972 0.2593 0.3790 0.279 0.048 0.457 53.70 0.589 0.525 175 186 -0.394624 0.107527

ENSMUST00000025202 1536 0.1355 0.5794 0.1335 0.3964 0.307 0.258 0.564 41.36 0.781 0.596 489 511 -0.112329 0.121331

ENSMUST00000025319 453 0.1709 0.4957 0.1880 0.4196 0.246 0.238 0.551 56.91 0.714 0.589 147 150 -0.718667 0.053333

ENSMUST00000023335 1533 0.3776 0.2424 0.3400 0.2615 0.193 -0.040 0.383 54.95 0.403 0.473 499 510 -0.155882 0.078431

ENSMUST00000025079 6513 0.2601 0.3606 0.2777 0.3524 0.205 0.020 0.431 52.53 0.564 0.524 2105 2170 -0.694378 0.059908

ENSMUST00000023845 990 0.2328 0.3740 0.2623 0.3879 0.225 0.074 0.450 52.12 0.601 0.532 313 329 -0.229179 0.109422

ENSMUST00000027165 657 0.2903 0.3978 0.2245 0.3571 0.197 0.009 0.412 56.91 0.588 0.489 211 218 -0.142202 0.133028

ENSMUST00000029783 1581 0.2829 0.3390 0.2961 0.3884 0.226 -0.008 0.422 56.61 0.549 0.505 510 526 -0.465779 0.102662

ENSMUST00000028814 981 0.1959 0.4980 0.1890 0.4025 0.265 0.157 0.502 46.84 0.693 0.548 313 326 -0.647853 0.070552

ENSMUST00000023024 906 0.1741 0.4777 0.1446 0.4625 0.261 0.049 0.447 41.59 0.744 0.597 293 301 -0.737209 0.053156

ENSMUST00000028386 3387 0.3594 0.2880 0.3459 0.2855 0.222 0.001 0.414 56.52 0.439 0.433 1073 1128 -0.100443 0.086879

ENSMUST00000023869 597 0.3551 0.2681 0.4387 0.2817 0.225 -0.130 0.366 50.60 0.397 0.434 194 198 -0.717172 0.070707

ENSMUST00000029727 1890 0.3181 0.2684 0.3319 0.3288 0.195 -0.003 0.411 57.21 0.470 0.475 598 629 -0.241017 0.073132

ENSMUST00000029271 2733 0.2483 0.4175 0.1896 0.4151 0.243 0.111 0.474 52.97 0.646 0.527 875 910 -0.109890 0.103297

ENSMUST00000028856 465 0.1643 0.5357 0.1215 0.3535 0.286 0.218 0.534 40.93 0.753 0.558 146 154 0.758442 0.207792

ENSMUST00000028764 378 0.1101 0.6422 0.0879 0.3750 0.325 0.364 0.634 29.63 0.837 0.688 123 125 0.047200 0.040000

ENSMUST00000027036 693 0.3925 0.2957 0.3235 0.2078 0.213 -0.010 0.409 60.03 0.405 0.471 215 230 0.114348 0.060870

ENSMUST00000028842 1173 0.2681 0.4416 0.2296 0.3735 0.312 0.160 0.518 52.90 0.613 0.505 380 390 -0.220000 0.115385

ENSMUST00000022271 2046 0.2527 0.3978 0.2628 0.3354 0.216 0.050 0.443 51.67 0.582 0.526 655 681 -0.262702 0.093979

ENSMUST00000025904 633 0.2299 0.3448 0.2312 0.3892 0.211 -0.010 0.405 58.35 0.610 0.570 205 210 0.176190 0.057143

ENSMUST00000023221 1866 0.2008 0.4261 0.1573 0.3896 0.255 0.175 0.495 47.83 0.691 0.600 596 621 0.400322 0.096618

ENSMUST00000023087 1053 0.3083 0.2932 0.3333 0.3811 0.226 0.007 0.427 55.04 0.500 0.466 342 350 -0.553429 0.088571

ENSMUST00000028926 897 0.2824 0.3796 0.2977 0.3970 0.276 -0.017 0.437 50.21 0.563 0.485 286 298 -0.456376 0.093960

ENSMUST00000022803 795 0.2489 0.4044 0.1667 0.3797 0.255 0.113 0.486 50.33 0.645 0.578 251 264 -0.073106 0.102273

ENSMUST00000029406 2739 0.4035 0.2676 0.3192 0.2753 0.226 -0.023 0.402 51.62 0.415 0.430 878 912 0.130702 0.114035

ENSMUST00000027885 1473 0.2861 0.3529 0.3276 0.3395 0.227 0.066 0.460 54.74 0.523 0.474 463 490 -0.634082 0.097959

ENSMUST00000022895 1878 0.2272 0.4841 0.2157 0.3925 0.272 0.120 0.500 48.64 0.658 0.511 602 625 -0.676640 0.089600

ENSMUST00000028966 540 0.3041 0.4122 0.2230 0.2308 0.266 0.007 0.459 41.88 0.547 0.547 172 179 -0.121788 0.022346

ENSMUST00000023285 1416 0.1934 0.3435 0.2327 0.3964 0.203 0.065 0.437 47.91 0.629 0.622 458 471 0.046709 0.025478

ENSMUST00000025521 807 0.1498 0.4758 0.1327 0.4054 0.219 0.185 0.511 41.52 0.756 0.685 262 268 -0.612313 0.085821

ENSMUST00000025217 2040 0.4489 0.2428 0.2888 0.2790 0.254 0.058 0.463 52.96 0.407 0.470 659 679 -0.388512 0.048601

ENSMUST00000023270 2571 0.3613 0.2819 0.2575 0.3626 0.218 0.017 0.427 56.24 0.504 0.499 832 856 -0.465187 0.047897

ENSMUST00000026540 450 0.3458 0.3364 0.2203 0.3739 0.280 0.057 0.437 53.39 0.556 0.562 142 149 -0.661074 0.040268

ENSMUST00000025679 816 0.1800 0.5550 0.1800 0.4462 0.330 0.222 0.556 41.91 0.729 0.549 266 271 -0.552768 0.107011

ENSMUST00000029116 1080 0.3521 0.3239 0.2436 0.3462 0.228 0.032 0.430 57.28 0.521 0.505 349 359 -0.438162 0.083565

ENSMUST00000026667 1236 0.2156 0.4313 0.1720 0.4662 0.250 0.137 0.495 50.13 0.686 0.538 392 411 -0.259368 0.072993

ENSMUST00000023453 666 0.1828 0.4247 0.1647 0.4364 0.255 0.136 0.502 51.10 0.709 0.624 213 221 -0.412217 0.058824

ENSMUST00000023123 4464 0.4119 0.3349 0.2219 0.1829 0.204 0.107 0.486 48.42 0.447 0.632 1460 1487 -0.797445 0.028245

ENSMUST00000022576 1269 0.3214 0.3869 0.3133 0.2714 0.236 0.078 0.469 54.69 0.501 0.450 405 422 -0.344550 0.125592

ENSMUST00000028114 1581 0.2458 0.4289 0.2222 0.3854 0.240 0.083 0.460 52.45 0.628 0.522 511 526 -0.464639 0.089354

ENSMUST00000022176 2664 0.3156 0.3268 0.2826 0.3065 0.219 0.006 0.416 56.28 0.504 0.493 847 887 0.118151 0.066516

ENSMUST00000025818 2292 0.2136 0.3981 0.2179 0.3513 0.215 0.107 0.465 47.74 0.632 0.612 750 763 -0.403408 0.049803

ENSMUST00000029838 1050 0.3806 0.2687 0.3162 0.3254 0.201 -0.018 0.415 54.43 0.450 0.469 342 349 -0.805158 0.085960

ENSMUST00000024042 1053 0.2193 0.4349 0.2387 0.4534 0.315 0.096 0.499 45.56 0.657 0.562 341 350 -0.540857 0.068571

ENSMUST00000028935 414 0.3704 0.3426 0.2268 0.3407 0.230 0.031 0.431 41.39 0.523 0.448 130 137 -0.117518 0.124088

ENSMUST00000029744 3504 0.3040 0.3404 0.2242 0.3406 0.209 0.053 0.440 53.87 0.557 0.547 1137 1167 -0.108655 0.079692

ENSMUST00000027933 2190 0.3810 0.3044 0.3217 0.2123 0.219 -0.019 0.416 54.37 0.420 0.476 704 729 -0.501783 0.069959

ENSMUST00000025946 1494 0.2375 0.4050 0.1549 0.4579 0.217 0.087 0.455 46.92 0.678 0.564 479 497 -0.105433 0.088531

ENSMUST00000023055 318 0.1667 0.4861 0.1216 0.5775 0.299 0.006 0.443 37.65 0.784 0.552 97 105 -0.380952 0.104762

ENSMUST00000025631 648 0.2308 0.3669 0.3101 0.3775 0.254 0.125 0.498 52.78 0.575 0.518 207 215 -0.575814 0.074419

ENSMUST00000027736 2379 0.3506 0.2712 0.3417 0.2835 0.226 0.015 0.430 55.70 0.441 0.489 777 792 -0.754545 0.025253

ENSMUST00000024596 1671 0.2111 0.4371 0.1544 0.4000 0.249 0.156 0.491 46.76 0.689 0.564 527 556 0.443165 0.111511

ENSMUST00000022464 342 0.3778 0.3667 0.2985 0.2698 0.312 0.053 0.471 61.00 0.481 0.457 104 113 -0.444248 0.088496

ENSMUST00000024928 975 0.2602 0.4238 0.1897 0.3548 0.248 0.168 0.508 53.98 0.626 0.555 305 324 -0.232408 0.101852

ENSMUST00000027153 1293 0.2909 0.3606 0.3185 0.2857 0.234 0.076 0.468 59.00 0.505 0.496 410 430 -0.240465 0.083721

ENSMUST00000024107 636 0.2216 0.3593 0.2679 0.3795 0.230 0.098 0.468 51.58 0.600 0.618 205 211 -0.663507 0.047393

ENSMUST00000025997 717 0.3290 0.2839 0.3770 0.3388 0.258 0.057 0.472 54.10 0.463 0.454 229 238 -0.896639 0.058824

ENSMUST00000022091 429 0.2743 0.3717 0.2718 0.3333 0.208 0.127 0.496 51.44 0.563 0.545 135 142 -0.923240 0.084507

ENSMUST00000029069 1008 0.3946 0.2414 0.2895 0.3441 0.250 0.098 0.476 57.13 0.451 0.475 328 335 -0.462388 0.044776

ENSMUST00000025826 1371 0.1832 0.5089 0.1072 0.3926 0.268 0.219 0.526 42.01 0.751 0.577 437 456 0.620175 0.125000

ENSMUST00000026032 1062 0.3197 0.3234 0.2908 0.3435 0.230 0.063 0.446 61.00 0.513 0.513 345 353 -0.522663 0.056657

ENSMUST00000029552 570 0.3677 0.2968 0.2482 0.3308 0.198 -0.031 0.390 56.27 0.495 0.483 182 189 -0.193122 0.100529

ENSMUST00000022122 1260 0.3176 0.3559 0.2109 0.3746 0.222 0.027 0.428 52.31 0.569 0.523 404 419 -0.460143 0.078759

ENSMUST00000028909 1215 0.3581 0.3277 0.3079 0.3438 0.246 0.017 0.433 54.60 0.496 0.445 395 404 -0.589604 0.084158

ENSMUST00000023460 471 0.3136 0.2966 0.3304 0.4000 0.234 0.060 0.477 51.20 0.510 0.483 151 156 -0.910897 0.121795

ENSMUST00000026795 2025 0.2133 0.5067 0.1799 0.4167 0.300 0.145 0.503 47.66 0.693 0.536 644 674 -0.280564 0.121662

ENSMUST00000026119 1458 0.2281 0.4662 0.0994 0.4341 0.258 0.143 0.484 44.12 0.724 0.570 457 485 0.222887 0.117526

ENSMUST00000029336 3006 0.4044 0.2354 0.3757 0.2727 0.203 -0.020 0.406 53.20 0.382 0.426 968 1001 -0.471528 0.075924

ENSMUST00000027884 624 0.3030 0.2955 0.3960 0.3972 0.210 -0.076 0.387 59.84 0.490 0.438 194 207 -0.842995 0.062802

ENSMUST00000029658 2838 0.2834 0.3665 0.3066 0.3281 0.232 0.059 0.456 53.17 0.533 0.481 895 945 -0.344444 0.118519

ENSMUST00000022543 1299 0.2656 0.3187 0.3466 0.3578 0.224 -0.008 0.422 57.37 0.519 0.492 412 432 -0.405093 0.113426

ENSMUST00000026076 1407 0.2306 0.4689 0.1994 0.3711 0.276 0.143 0.509 50.44 0.659 0.536 454 468 -0.300855 0.074786

ENSMUST00000022842 1626 0.3186 0.3627 0.2488 0.3547 0.256 0.038 0.440 53.63 0.545 0.499 516 541 -0.173752 0.038817

ENSMUST00000022642 2382 0.3470 0.3207 0.3446 0.3083 0.214 -0.075 0.382 53.56 0.470 0.462 764 793 -0.439344 0.073140

ENSMUST00000023356 810 0.3167 0.3982 0.2525 0.2995 0.256 0.087 0.477 56.22 0.545 0.497 264 269 -0.557249 0.078067

ENSMUST00000023363 1971 0.3790 0.2795 0.2599 0.3663 0.224 0.008 0.413 57.30 0.488 0.456 639 656 -0.113567 0.097561

ENSMUST00000023726 432 0.3394 0.4404 0.2165 0.3488 0.253 -0.018 0.419 45.51 0.574 0.469 136 143 -0.081818 0.111888

ENSMUST00000028727 621 0.3287 0.3916 0.2384 0.3786 0.282 0.117 0.500 49.72 0.568 0.511 192 206 -0.865049 0.019417

ENSMUST00000025374 1017 0.1842 0.5075 0.1407 0.4080 0.269 0.153 0.508 47.63 0.734 0.603 323 338 -0.339053 0.050296

ENSMUST00000025472 1488 0.1610 0.5537 0.1090 0.4045 0.295 0.219 0.544 43.23 0.776 0.596 478 495 0.047273 0.119192

ENSMUST00000024791 684 0.2120 0.4348 0.1658 0.3867 0.212 0.039 0.427 49.25 0.682 0.604 220 227 -0.029956 0.052863

ENSMUST00000027128 3333 0.3092 0.2511 0.3717 0.3937 0.222 0.025 0.439 53.27 0.482 0.446 1070 1110 -0.853874 0.030631

ENSMUST00000027444 453 0.2407 0.4167 0.3571 0.2857 0.209 0.020 0.433 56.33 0.532 0.478 141 150 -0.288667 0.113333

ENSMUST00000023132 1608 0.2743 0.4175 0.1864 0.4146 0.274 0.064 0.457 48.35 0.635 0.526 512 535 -0.385421 0.082243

ENSMUST00000025500 1860 0.2823 0.3488 0.3041 0.3493 0.227 0.068 0.453 53.68 0.537 0.475 594 619 -0.088207 0.122779

ENSMUST00000023007 3750 0.2454 0.4232 0.2105 0.3734 0.232 0.102 0.468 52.18 0.627 0.524 1209 1249 -0.043875 0.088070

ENSMUST00000025363 627 0.2267 0.3430 0.2364 0.4172 0.200 0.084 0.454 53.01 0.620 0.542 205 208 -0.257212 0.062500

ENSMUST00000022328 1986 0.2455 0.4729 0.1802 0.3038 0.266 0.117 0.492 44.36 0.643 0.589 642 661 -0.426626 0.083207

ENSMUST00000029141 1857 0.1996 0.4476 0.1846 0.4316 0.234 0.139 0.497 47.76 0.690 0.587 591 618 -0.600809 0.124595

ENSMUST00000027565 1161 0.3536 0.3179 0.4022 0.2667 0.250 0.013 0.433 52.98 0.428 0.413 367 386 -0.348446 0.106218

ENSMUST00000025585 1254 0.3015 0.4179 0.2305 0.2727 0.256 0.093 0.470 56.41 0.558 0.504 396 417 0.000000 0.079137

ENSMUST00000028279 2019 0.3943 0.1943 0.4190 0.2379 0.194 -0.095 0.362 50.83 0.340 0.435 647 672 -0.422917 0.053571

ENSMUST00000027027 2664 0.4128 0.2332 0.4250 0.2716 0.219 -0.053 0.408 51.00 0.370 0.412 844 887 -1.027.058 0.074408

ENSMUST00000024763 591 0.2105 0.3947 0.2662 0.3537 0.189 0.067 0.438 47.75 0.605 0.560 185 196 -0.502551 0.066327

ENSMUST00000025929 885 0.2043 0.3872 0.2727 0.3616 0.198 0.017 0.423 54.04 0.601 0.550 286 294 -0.434354 0.057823

ENSMUST00000028990 1767 0.3222 0.3305 0.2489 0.3333 0.205 0.001 0.410 55.35 0.531 0.507 573 588 -0.389966 0.062925

ENSMUST00000025364 774 0.3442 0.2930 0.3102 0.2907 0.202 -0.087 0.367 51.26 0.461 0.468 245 257 0.388716 0.136187

ENSMUST00000023329 417 0.3628 0.3982 0.1584 0.2872 0.259 0.136 0.496 50.85 0.558 0.505 129 138 0.253623 0.057971

ENSMUST00000026455 792 0.2479 0.4188 0.1408 0.3452 0.194 0.073 0.447 49.52 0.656 0.586 253 263 0.567681 0.125475

ENSMUST00000026129 1215 0.2075 0.4371 0.1414 0.5141 0.244 0.050 0.454 42.72 0.723 0.578 394 404 -0.522277 0.081683

ENSMUST00000025271 1059 0.2271 0.3919 0.2168 0.3971 0.205 -0.007 0.411 50.30 0.636 0.581 341 352 -0.410511 0.079545

ENSMUST00000029709 1101 0.2143 0.4252 0.2467 0.3253 0.160 0.012 0.421 49.74 0.615 0.586 356 366 -0.544262 0.060109

ENSMUST00000024004 435 0.2909 0.3727 0.2143 0.3958 0.189 0.032 0.439 50.62 0.598 0.509 132 144 -0.525695 0.083333

ENSMUST00000026273 1908 0.2228 0.4588 0.1781 0.3630 0.257 0.113 0.475 48.13 0.664 0.535 602 635 0.369449 0.135433

ENSMUST00000028328 1488 0.2270 0.4610 0.1675 0.3360 0.256 0.173 0.509 49.04 0.666 0.584 479 495 0.033737 0.101010

ENSMUST00000025036 3261 0.1963 0.4630 0.1690 0.4191 0.228 0.063 0.456 45.26 0.701 0.591 1048 1086 -0.563352 0.082873

ENSMUST00000023143 486 0.1986 0.4752 0.2373 0.2807 0.262 0.105 0.471 41.90 0.639 0.590 155 161 0.130435 0.093168

ENSMUST00000025764 450 0.1681 0.5042 0.2018 0.3694 0.286 0.257 0.563 46.73 0.701 0.584 144 149 -0.343624 0.073826

ENSMUST00000022566 3735 0.2199 0.4580 0.1988 0.3723 0.226 0.096 0.476 48.42 0.660 0.578 1210 1244 -0.742765 0.060289

ENSMUST00000022563 1854 0.3477 0.3210 0.2841 0.3493 0.195 -0.047 0.391 57.86 0.505 0.463 598 617 -0.403242 0.098865

ENSMUST00000025288 3936 0.2483 0.5045 0.1723 0.4140 0.303 0.149 0.520 50.15 0.681 0.530 1277 1311 -0.597559 0.067124

ENSMUST00000027061 477 0.2171 0.4419 0.1707 0.3932 0.256 0.149 0.493 51.62 0.678 0.563 152 158 -0.104430 0.050633

ENSMUST00000027475 3876 0.3489 0.2436 0.3462 0.3506 0.201 -0.005 0.426 54.04 0.462 0.506 1239 1291 -1.232.688 0.058869

ENSMUST00000027384 1779 0.3381 0.3158 0.2495 0.3195 0.235 0.038 0.437 55.40 0.511 0.511 577 592 -0.064527 0.069257

ENSMUST00000024857 318 0.2838 0.4324 0.2133 0.4225 0.284 0.089 0.475 51.66 0.626 0.533 99 105 -0.799048 0.076190

ENSMUST00000022293 888 0.3959 0.2816 0.2731 0.3088 0.276 0.027 0.455 51.88 0.458 0.468 288 295 -0.223390 0.105085

ENSMUST00000022429 543 0.3986 0.2536 0.2993 0.3095 0.268 0.072 0.453 56.52 0.435 0.454 170 180 -0.083889 0.088889

ENSMUST00000025357 582 0.3725 0.2941 0.3841 0.2667 0.218 -0.076 0.369 61.00 0.412 0.402 187 193 -0.010363 0.077720

ENSMUST00000029840 939 0.3537 0.3089 0.3607 0.2663 0.254 0.009 0.431 60.52 0.437 0.466 295 312 -0.192628 0.121795

ENSMUST00000023530 639 0.3313 0.3193 0.2289 0.3800 0.249 0.088 0.453 54.63 0.542 0.502 203 212 -0.104717 0.080189

ENSMUST00000022105 1848 0.2302 0.4391 0.1771 0.3846 0.244 0.137 0.484 47.44 0.659 0.529 580 615 0.442114 0.146341

ENSMUST00000025649 3423 0.3133 0.4085 0.1917 0.3526 0.243 0.045 0.441 52.25 0.591 0.508 1105 1140 -0.107895 0.078070

ENSMUST00000028410 11355 0.4016 0.2436 0.4229 0.2424 0.213 -0.040 0.409 51.72 0.362 0.420 3680 3784 -0.845824 0.055497

ENSMUST00000027992 768 0.2931 0.3678 0.3333 0.3873 0.223 -0.061 0.394 54.48 0.533 0.437 246 255 -0.914902 0.070588

ENSMUST00000023568 822 0.3411 0.3084 0.3268 0.2895 0.211 0.050 0.448 58.97 0.464 0.479 261 273 -0.246886 0.084249

ENSMUST00000021991 1239 0.3932 0.2291 0.3585 0.3030 0.205 -0.016 0.395 51.32 0.405 0.413 405 412 -0.234951 0.092233

ENSMUST00000025846 2535 0.3516 0.3016 0.3328 0.3046 0.193 -0.098 0.366 56.04 0.460 0.459 811 844 -0.486374 0.058057

ENSMUST00000029454 1248 0.3397 0.3686 0.2319 0.4570 0.289 -0.004 0.420 57.82 0.577 0.477 402 415 -0.647711 0.103614

ENSMUST00000028527 2661 0.4422 0.2128 0.4292 0.2249 0.229 0.009 0.433 51.24 0.328 0.394 857 886 -0.678555 0.057562

ENSMUST00000025329 873 0.2000 0.4095 0.2457 0.4395 0.203 0.031 0.428 52.34 0.650 0.551 283 290 -0.507586 0.041379

ENSMUST00000027856 2631 0.3933 0.2399 0.3692 0.2358 0.198 -0.006 0.421 54.11 0.378 0.471 850 876 -0.758562 0.054795

ENSMUST00000025547 735 0.3175 0.2593 0.3503 0.3315 0.183 -0.019 0.406 59.88 0.460 0.467 239 244 -0.456967 0.102459

ENSMUST00000025388 714 0.2199 0.4450 0.2294 0.3841 0.286 0.226 0.546 47.19 0.646 0.532 229 237 -0.372152 0.092827

ENSMUST00000027502 1182 0.3239 0.3711 0.2135 0.3550 0.225 0.078 0.463 51.93 0.564 0.519 374 393 -0.206107 0.106870

ENSMUST00000025910 615 0.2422 0.3851 0.2083 0.3497 0.177 -0.002 0.430 54.67 0.617 0.626 193 204 -0.970098 0.063725

ENSMUST00000029814 2640 0.2890 0.4177 0.2194 0.3589 0.271 0.133 0.498 54.22 0.596 0.498 836 879 -0.241638 0.135381

ENSMUST00000027753 3582 0.2941 0.3777 0.2559 0.3376 0.218 0.015 0.439 53.06 0.561 0.546 1167 1193 -0.568818 0.054484

ENSMUST00000025003 1395 0.2072 0.4987 0.1541 0.3390 0.254 0.141 0.511 44.63 0.698 0.625 450 464 -0.796121 0.073276

ENSMUST00000028859 1899 0.2634 0.3505 0.2581 0.3972 0.223 0.027 0.432 49.71 0.578 0.509 600 632 -0.031646 0.083861

ENSMUST00000024897 750 0.3472 0.2591 0.3946 0.2686 0.206 -0.058 0.376 55.88 0.409 0.447 237 249 -0.395181 0.060241

ENSMUST00000027082 525 0.1329 0.6573 0.0693 0.4421 0.319 0.378 0.648 36.97 0.840 0.590 162 174 -0.853448 0.178161

ENSMUST00000026890 609 0.0933 0.6000 0.1133 0.4931 0.321 0.138 0.510 39.74 0.839 0.601 192 202 -0.187129 0.094059

ENSMUST00000025568 489 0.1533 0.5547 0.1947 0.3366 0.279 0.282 0.562 48.18 0.719 0.502 153 162 0.704321 0.179012

ENSMUST00000028663 1563 0.1966 0.4939 0.1756 0.3652 0.260 0.135 0.490 46.41 0.695 0.588 498 520 -0.487500 0.057692

ENSMUST00000024854 2115 0.3063 0.3316 0.2559 0.3276 0.220 0.029 0.443 55.22 0.532 0.509 688 704 -0.334659 0.071023

ENSMUST00000028583 594 0.3129 0.2381 0.3929 0.2893 0.176 -0.024 0.399 61.00 0.420 0.484 193 197 -0.446700 0.040609

ENSMUST00000026723 657 0.4560 0.2473 0.2857 0.2806 0.198 -0.064 0.374 49.35 0.398 0.416 211 218 -0.134862 0.091743

ENSMUST00000029469 474 0.2787 0.3607 0.3148 0.3365 0.184 -0.025 0.408 56.72 0.537 0.488 147 157 -0.666879 0.133758

ENSMUST00000029684 1050 0.2448 0.4724 0.1718 0.3241 0.258 0.139 0.496 48.84 0.654 0.580 335 349 0.037822 0.131805

ENSMUST00000022438 2574 0.4134 0.2528 0.3804 0.2442 0.201 -0.100 0.359 49.06 0.373 0.391 816 857 0.155893 0.123687

ENSMUST00000027362 2424 0.3167 0.3733 0.2378 0.3819 0.255 0.077 0.462 53.57 0.570 0.497 770 807 -0.502230 0.085502

ENSMUST00000028997 1254 0.2848 0.3437 0.2538 0.3766 0.210 0.066 0.453 54.09 0.567 0.521 406 417 -0.627818 0.071942

ENSMUST00000027559 1878 0.2320 0.3977 0.2025 0.3941 0.225 0.065 0.462 51.26 0.643 0.591 610 625 -0.439840 0.054400

ENSMUST00000024179 2706 0.2827 0.3704 0.1810 0.3379 0.190 0.035 0.428 49.21 0.601 0.612 884 901 -0.551609 0.076582

ENSMUST00000028205 870 0.2564 0.3487 0.2115 0.5207 0.218 -0.063 0.394 47.63 0.649 0.531 279 289 -1.234.602 0.044983

ENSMUST00000027409 1410 0.1647 0.5260 0.1293 0.4667 0.280 0.178 0.526 42.72 0.768 0.596 456 469 -0.721109 0.057569

ENSMUST00000026671 4008 0.2323 0.4325 0.1951 0.3784 0.230 0.087 0.461 48.60 0.648 0.549 1267 1335 -0.177978 0.083146

ENSMUST00000028362 5172 0.3466 0.3473 0.2916 0.3114 0.238 0.029 0.444 56.39 0.501 0.480 1626 1723 -0.465003 0.121300

ENSMUST00000026672 2472 0.2890 0.3716 0.2975 0.3432 0.220 0.020 0.434 57.19 0.540 0.485 795 823 -0.424787 0.070474

ENSMUST00000022704 801 0.2850 0.4299 0.1390 0.4819 0.326 0.155 0.506 51.56 0.664 0.518 259 266 -0.083083 0.105263

ENSMUST00000025850 822 0.2100 0.4338 0.2128 0.3421 0.233 0.213 0.532 45.35 0.643 0.620 269 273 -0.850549 0.047619

ENSMUST00000029777 831 0.3291 0.3932 0.2410 0.2805 0.226 0.041 0.439 60.15 0.527 0.438 262 276 0.641305 0.159420

ENSMUST00000025745 651 0.2398 0.3450 0.2768 0.3631 0.191 -0.007 0.405 56.66 0.571 0.526 210 216 -0.204630 0.074074

ENSMUST00000022853 741 0.2750 0.4050 0.3373 0.2515 0.234 0.057 0.466 58.43 0.521 0.520 234 246 -0.464634 0.117886

ENSMUST00000025292 3135 0.2324 0.4023 0.1688 0.4589 0.232 0.056 0.448 47.86 0.679 0.582 1014 1044 -0.572414 0.073755

ENSMUST00000027257 750 0.3387 0.2688 0.3892 0.3393 0.216 0.023 0.442 56.18 0.442 0.427 242 249 -0.597992 0.096386

ENSMUST00000023734 1113 0.1149 0.5621 0.1250 0.3916 0.266 0.306 0.594 36.60 0.800 0.654 355 370 -0.360541 0.072973

ENSMUST00000022744 723 0.2759 0.3645 0.2849 0.3099 0.178 0.051 0.444 61.00 0.543 0.531 234 240 -0.437500 0.062500

ENSMUST00000028106 3189 0.3178 0.3348 0.3078 0.3045 0.213 0.014 0.424 54.26 0.496 0.485 1034 1062 -0.180038 0.091337

ENSMUST00000027232 522 0.4526 0.2482 0.3680 0.2087 0.223 -0.056 0.380 54.93 0.349 0.432 166 173 -0.300578 0.086705

ENSMUST00000029082 747 0.2895 0.3895 0.2576 0.3536 0.243 0.020 0.434 61.00 0.566 0.505 244 248 -0.433065 0.068548

ENSMUST00000027657 1410 0.3911 0.3045 0.3266 0.2322 0.228 0.008 0.430 51.63 0.422 0.468 453 469 -0.266525 0.089552

ENSMUST00000025724 801 0.3237 0.3768 0.2980 0.2941 0.225 0.033 0.448 56.77 0.514 0.462 259 266 -0.618045 0.086466

ENSMUST00000029598 1635 0.4100 0.2506 0.3916 0.2054 0.197 -0.030 0.392 48.87 0.354 0.411 525 544 -0.163603 0.053309

ENSMUST00000026555 765 0.2133 0.4929 0.1393 0.3454 0.245 0.092 0.467 47.05 0.701 0.596 244 254 -0.457874 0.090551

ENSMUST00000026220 1080 0.1913 0.5101 0.1554 0.3991 0.260 0.179 0.519 42.44 0.718 0.559 341 359 -0.224234 0.136490

ENSMUST00000023213 1182 0.2058 0.3344 0.1574 0.5063 0.228 0.141 0.476 49.96 0.697 0.634 380 393 -0.132316 0.040712

ENSMUST00000029448 2982 0.4521 0.1617 0.5194 0.2358 0.231 -0.014 0.425 45.23 0.286 0.345 965 993 -1.025.478 0.044310

ENSMUST00000025936 990 0.2103 0.5277 0.1897 0.3425 0.278 0.179 0.524 46.77 0.683 0.524 319 329 -0.002432 0.121581

ENSMUST00000025383 897 0.2929 0.3347 0.2408 0.3565 0.212 0.096 0.457 56.76 0.557 0.538 291 298 -0.311745 0.050336

ENSMUST00000023920 591 0.1646 0.4390 0.1582 0.4183 0.214 0.110 0.463 48.12 0.723 0.631 188 196 -0.138265 0.056122

ENSMUST00000028963 1209 0.1944 0.4290 0.2013 0.4231 0.223 0.086 0.451 47.35 0.677 0.568 384 402 -0.156468 0.082090

ENSMUST00000022245 1329 0.2667 0.3639 0.2222 0.3932 0.225 0.096 0.465 56.69 0.600 0.551 430 442 -0.336425 0.088235

ENSMUST00000025963 2424 0.3514 0.2605 0.3297 0.3657 0.227 -0.008 0.406 54.22 0.472 0.443 786 807 -0.525279 0.060719

ENSMUST00000023405 561 0.3681 0.2917 0.2857 0.3520 0.236 0.010 0.419 56.97 0.480 0.439 179 186 -0.193548 0.069892

ENSMUST00000025659 642 0.4390 0.2134 0.4024 0.2013 0.231 0.013 0.444 48.97 0.327 0.444 205 213 -0.734272 0.042254

ENSMUST00000022136 2067 0.3778 0.2481 0.3809 0.2687 0.192 -0.015 0.408 53.63 0.396 0.436 669 688 -0.448546 0.074128

ENSMUST00000025488 348 0.3605 0.2907 0.3023 0.3415 0.217 0.041 0.427 56.41 0.482 0.446 110 115 -0.257391 0.104348

ENSMUST00000025061 309 0.2209 0.4884 0.2361 0.2941 0.193 0.066 0.459 44.02 0.633 0.552 98 102 -0.225490 0.019608

ENSMUST00000029135 2106 0.2791 0.3971 0.2212 0.3607 0.263 0.069 0.459 49.88 0.595 0.543 669 701 -0.318117 0.105563

ENSMUST00000027853 384 0.2745 0.2549 0.3608 0.3077 0.163 -0.036 0.385 57.31 0.462 0.491 117 127 0.124409 0.125984

ENSMUST00000027643 357 0.1429 0.4286 0.3118 0.4773 0.252 0.118 0.505 49.77 0.649 0.492 111 118 -1.034.746 0.033898

ENSMUST00000027040 4767 0.4273 0.1966 0.4399 0.2563 0.212 -0.043 0.394 49.16 0.336 0.396 1534 1588 -0.559761 0.049118

ENSMUST00000024733 2943 0.2037 0.3950 0.1965 0.4089 0.219 0.066 0.447 48.34 0.664 0.609 949 980 -0.168571 0.061224

ENSMUST00000022548 657 0.3799 0.3073 0.2761 0.2628 0.208 0.079 0.450 49.27 0.459 0.472 209 218 -0.544495 0.082569

ENSMUST00000029367 1812 0.4013 0.2824 0.3837 0.2035 0.199 -0.034 0.402 55.67 0.378 0.434 569 603 -0.291376 0.129353

ENSMUST00000029186 2040 0.2685 0.3537 0.2457 0.3826 0.220 0.021 0.424 54.05 0.580 0.521 655 679 -0.111929 0.082474

ENSMUST00000028595 1536 0.3561 0.3081 0.3053 0.3343 0.214 -0.019 0.406 54.38 0.484 0.454 498 511 -0.493933 0.084149

ENSMUST00000028175 1080 0.2931 0.3552 0.2437 0.3496 0.213 0.097 0.479 52.29 0.562 0.510 349 359 -0.553482 0.064067

ENSMUST00000029696 1839 0.3659 0.2500 0.3301 0.2577 0.190 -0.065 0.381 53.38 0.416 0.520 598 612 -0.446569 0.062092

ENSMUST00000024870 1383 0.3000 0.3150 0.2735 0.3056 0.174 -0.013 0.390 58.14 0.510 0.490 441 460 0.276087 0.106522

ENSMUST00000022901 1056 0.3623 0.2717 0.3651 0.3261 0.211 -0.076 0.379 53.69 0.439 0.434 335 351 -0.223931 0.133903

ENSMUST00000029719 2271 0.2552 0.4205 0.2301 0.3351 0.241 0.115 0.487 53.32 0.603 0.534 743 756 -0.431217 0.071429

ENSMUST00000028981 807 0.4031 0.2347 0.3645 0.3158 0.226 -0.060 0.390 51.76 0.409 0.444 259 268 -0.425373 0.089552

ENSMUST00000026354 1530 0.2525 0.4343 0.1600 0.4270 0.270 0.147 0.506 47.26 0.668 0.574 494 509 -0.444008 0.037328

ENSMUST00000027422 1539 0.2471 0.4266 0.2080 0.3472 0.214 0.035 0.430 53.80 0.620 0.524 484 512 0.463672 0.117188

ENSMUST00000027843 1620 0.2972 0.3710 0.2704 0.3418 0.223 -0.007 0.410 54.28 0.545 0.484 517 539 -0.187570 0.109462

ENSMUST00000023615 894 0.2716 0.4033 0.1818 0.4076 0.254 0.090 0.462 49.42 0.630 0.512 292 297 0.036364 0.070707

ENSMUST00000022038 2178 0.3593 0.3315 0.2931 0.3258 0.255 0.064 0.454 56.13 0.493 0.463 685 725 -0.256828 0.089655

ENSMUST00000027959 570 0.3113 0.4305 0.2568 0.2302 0.260 0.097 0.478 54.35 0.533 0.527 182 189 -0.850265 0.058201

ENSMUST00000023849 1290 0.1775 0.5042 0.1774 0.3826 0.235 0.159 0.504 46.52 0.711 0.590 419 429 -0.375058 0.083916

ENSMUST00000022690 2235 0.2261 0.4204 0.1909 0.4107 0.255 0.108 0.468 48.51 0.660 0.569 729 744 -0.127823 0.072581

ENSMUST00000028866 918 0.4171 0.2613 0.4286 0.2857 0.268 -0.018 0.440 60.21 0.389 0.425 298 305 -1.261.312 0.072131

ENSMUST00000029080 360 0.3407 0.2527 0.3398 0.2828 0.207 0.011 0.427 56.71 0.436 0.504 117 119 -1.018.487 0.008403

ENSMUST00000029568 1047 0.2484 0.3987 0.2773 0.2578 0.239 0.106 0.471 52.15 0.550 0.498 327 348 0.648276 0.123563

ENSMUST00000028214 1188 0.2065 0.4419 0.1536 0.4355 0.233 0.054 0.447 44.25 0.706 0.592 385 395 -0.409873 0.075949

ENSMUST00000022976 3480 0.3022 0.3867 0.2682 0.3372 0.236 0.095 0.464 54.20 0.548 0.471 1114 1159 -0.157895 0.098361

ENSMUST00000023497 2046 0.3013 0.3648 0.2129 0.3811 0.236 0.104 0.472 53.75 0.584 0.542 654 681 -0.318062 0.092511

ENSMUST00000025542 1113 0.2625 0.3887 0.2255 0.3843 0.232 0.033 0.435 55.93 0.604 0.506 356 370 -0.011622 0.089189

ENSMUST00000026993 813 0.1822 0.5600 0.1135 0.4201 0.266 0.161 0.502 48.79 0.761 0.567 259 270 0.422592 0.137037

ENSMUST00000023790 1914 0.3112 0.4579 0.1793 0.2746 0.286 0.212 0.567 46.99 0.595 0.553 619 637 -0.593564 0.070644

ENSMUST00000025101 1056 0.2959 0.3109 0.3150 0.3589 0.220 0.015 0.427 55.05 0.510 0.472 337 351 -0.414815 0.074074

ENSMUST00000027299 1104 0.2152 0.4768 0.2174 0.3268 0.242 0.137 0.494 51.85 0.645 0.541 352 367 -0.114169 0.098093

ENSMUST00000022232 1758 0.3660 0.2638 0.3550 0.2929 0.195 -0.066 0.377 55.13 0.425 0.448 565 585 -0.224615 0.082051

ENSMUST00000022102 1620 0.2952 0.3867 0.1937 0.3933 0.257 0.053 0.439 50.97 0.604 0.491 512 539 0.057699 0.142857

ENSMUST00000023352 12387 0.3875 0.2614 0.3426 0.2913 0.212 -0.023 0.399 54.34 0.421 0.433 3957 4128 -0.198547 0.089632

ENSMUST00000026548 1737 0.2168 0.4847 0.1489 0.3713 0.257 0.143 0.502 45.69 0.696 0.603 556 578 -0.156401 0.089965

ENSMUST00000022499 927 0.3584 0.2920 0.3263 0.3362 0.229 -0.041 0.399 60.81 0.475 0.443 301 308 -0.563312 0.087662

ENSMUST00000024817 1467 0.2722 0.3402 0.2211 0.4770 0.222 0.036 0.439 52.75 0.620 0.519 469 488 -0.619877 0.040984

ENSMUST00000022977 1719 0.3418 0.3121 0.2803 0.3022 0.231 0.072 0.449 57.37 0.488 0.481 559 572 0.057867 0.101399

ENSMUST00000028950 879 0.2809 0.3574 0.2043 0.3889 0.274 0.118 0.475 52.04 0.596 0.529 282 292 -0.076370 0.034247

ENSMUST00000023836 645 0.4036 0.2108 0.3353 0.3187 0.183 -0.124 0.329 56.85 0.410 0.439 210 214 -0.001869 0.074766

ENSMUST00000029633 1146 0.2421 0.4434 0.1696 0.3822 0.282 0.154 0.492 52.80 0.656 0.520 366 381 0.361155 0.097113

ENSMUST00000025403 873 0.2134 0.4351 0.2294 0.3505 0.229 0.054 0.449 50.46 0.633 0.567 283 290 -0.052414 0.062069

ENSMUST00000023775 801 0.2456 0.4825 0.1354 0.3279 0.273 0.223 0.552 39.82 0.675 0.565 252 266 -0.119173 0.090226

ENSMUST00000023075 795 0.2098 0.4821 0.1538 0.3804 0.274 0.180 0.522 48.61 0.698 0.574 255 264 -0.240530 0.098485

ENSMUST00000027947 1569 0.3668 0.3037 0.3147 0.2906 0.221 0.044 0.440 57.34 0.458 0.464 507 522 -0.510728 0.093870

ENSMUST00000023159 1602 0.2760 0.4253 0.1634 0.3932 0.272 0.106 0.475 47.46 0.642 0.561 528 533 -0.375797 0.071295

ENSMUST00000023165 7326 0.3200 0.3330 0.2959 0.3029 0.221 0.022 0.433 54.31 0.503 0.529 2329 2441 -0.697460 0.043425

ENSMUST00000029371 1293 0.1662 0.5272 0.1438 0.4300 0.258 0.158 0.507 44.67 0.752 0.584 416 430 -0.490465 0.095349

ENSMUST00000028900 2520 0.2939 0.3485 0.2212 0.4020 0.232 0.032 0.430 51.54 0.585 0.537 810 839 -0.221812 0.085816

ENSMUST00000028187 4746 0.2197 0.4613 0.1843 0.3847 0.252 0.119 0.494 45.77 0.674 0.598 1548 1581 -0.451233 0.062619

ENSMUST00000024748 1809 0.1984 0.4254 0.1996 0.4105 0.234 0.074 0.455 47.55 0.672 0.575 589 602 -0.162292 0.058140

ENSMUST00000025680 1188 0.4044 0.2790 0.3407 0.2540 0.232 -0.020 0.408 60.31 0.408 0.435 373 395 -0.108101 0.136709

ENSMUST00000023828 6681 0.3842 0.2460 0.3126 0.2955 0.207 0.005 0.401 52.45 0.428 0.450 2156 2226 0.105571 0.069632

ENSMUST00000023689 1773 0.3134 0.3539 0.3018 0.2926 0.236 0.015 0.431 56.48 0.506 0.484 569 590 -0.277288 0.100000

ENSMUST00000023502 1353 0.3242 0.2909 0.2933 0.3945 0.218 -0.010 0.417 56.01 0.521 0.490 432 450 -0.616000 0.091111

ENSMUST00000025263 708 0.1604 0.5455 0.2044 0.3371 0.201 0.018 0.417 48.82 0.706 0.567 228 235 -0.002553 0.089362

ENSMUST00000023830 894 0.2146 0.4049 0.2072 0.4057 0.211 0.075 0.446 54.07 0.653 0.572 285 297 -0.226263 0.084175

ENSMUST00000022013 3288 0.2387 0.4310 0.2387 0.3678 0.251 0.110 0.478 53.27 0.616 0.503 1045 1095 0.139635 0.103196

ENSMUST00000027112 2721 0.2257 0.4665 0.2018 0.3766 0.239 0.084 0.471 49.12 0.657 0.553 868 906 -0.447792 0.105960

ENSMUST00000028389 972 0.2607 0.4669 0.1967 0.3289 0.262 0.155 0.503 50.50 0.630 0.552 308 323 -0.234365 0.061920

ENSMUST00000027172 1296 0.3056 0.3457 0.3600 0.2776 0.225 0.000 0.430 52.49 0.481 0.484 416 431 -0.563573 0.069606

ENSMUST00000027529 645 0.2125 0.4375 0.1512 0.4643 0.327 0.258 0.572 46.54 0.712 0.575 208 214 -0.292523 0.084112

ENSMUST00000028351 1434 0.3010 0.3743 0.2558 0.3112 0.246 0.112 0.483 56.79 0.548 0.526 449 477 -0.313837 0.073375

ENSMUST00000022964 642 0.3537 0.2109 0.3964 0.3333 0.200 -0.077 0.389 55.92 0.414 0.443 203 213 -0.798122 0.070423

ENSMUST00000026994 2697 0.1629 0.5607 0.1171 0.3761 0.255 0.175 0.511 39.97 0.766 0.616 869 898 -0.115033 0.080178

ENSMUST00000026625 1017 0.2863 0.3168 0.3569 0.2976 0.216 0.039 0.435 56.22 0.480 0.483 329 338 -0.152367 0.068047

ENSMUST00000027366 2484 0.2342 0.4491 0.1789 0.4266 0.273 0.118 0.492 48.22 0.674 0.551 788 827 -0.445586 0.089480

ENSMUST00000029482 2040 0.3059 0.3388 0.3373 0.2934 0.221 0.064 0.461 54.27 0.492 0.486 664 679 -0.514139 0.070692

ENSMUST00000028207 1881 0.2004 0.4826 0.1935 0.4056 0.273 0.129 0.489 46.39 0.687 0.545 597 626 -0.247923 0.092652

ENSMUST00000025397 483 0.2857 0.3061 0.3884 0.4000 0.242 -0.065 0.411 57.89 0.503 0.465 151 160 -1.028.750 0.050000

ENSMUST00000022592 951 0.2462 0.4077 0.2511 0.3410 0.259 0.185 0.530 53.37 0.596 0.541 302 316 -0.374051 0.098101

ENSMUST00000027467 1194 0.2799 0.3679 0.2459 0.3574 0.241 0.036 0.434 49.69 0.568 0.481 380 397 -0.054156 0.088161

ENSMUST00000028852 1299 0.2456 0.3817 0.2515 0.3653 0.198 0.037 0.442 57.05 0.594 0.539 416 432 -0.535185 0.069444

ENSMUST00000027769 1191 0.3281 0.2271 0.3931 0.3346 0.175 0.003 0.409 57.84 0.425 0.455 379 396 -0.394192 0.098485

ENSMUST00000027560 1167 0.2813 0.4343 0.1642 0.3522 0.218 0.069 0.445 50.06 0.626 0.510 366 388 0.407732 0.115979
[truncated: 2,707,568 more chars]
