# Supplementary material for: Systematic Analysis of Mouse Genome Reveals Distinct Evolutionary and Functional Properties Among Circadian and Ultradian Genes
Source: Front Physiol. 2018 Aug 23;9:1178. doi: 10.3389/fphys.2018.01178 (PMC6115496; doi:10.3389/fphys.2018.01178)
Supplement: FILE S2 — Original output of JCat. [file Data_Sheet_2.doc]

Transcript_ID Transcript_length Gene CAI_value

ENSMUST00000000161 2217 Itgb2l 0.3160215274055636

ENSMUST00000000001 1065 Gnai3 0.20397550054358377

ENSMUST00000000096 1659 Tbx4 0.37335555384910063

ENSMUST00000000137 1185 Actr2 0.19505866645978803

ENSMUST00000000122 1284 Ngfr 0.3538708344103882

ENSMUST00000000087 1995 Scmh1 0.25844940093621943

ENSMUST00000000175 480 Sdhd 0.23020001277760455

ENSMUST00000000028 1701 Cdc45 0.27781626333560666

ENSMUST00000000109 3063 Cntn1 0.22772035561361859

ENSMUST00000000095 2136 Tbx2 0.32254810023820446

ENSMUST00000000090 441 Cox5a 0.20248518695794362

ENSMUST00000000058 489 Cav2 0.31566433320263987

ENSMUST00000000127 1068 Wnt3 0.37829483292900923

ENSMUST00000000080 957 Klf6 0.23322589656093767

ENSMUST00000000153 1140 Gna12 0.4413195406642146

ENSMUST00000000049 1038 Apoh 0.23793902390144683

ENSMUST00000000003 525 Pbsn 0.19320724787232968

ENSMUST00000000129 2472 Fer 0.1980861971190314

ENSMUST00000000033 543 Igf2 0.3075049919046991

ENSMUST00000000163 1113 Igsf5 0.235899950246909

ENSMUST00000000186 756 Fgf23 0.3041235512635795

ENSMUST00000000010 753 Hoxb9 0.2918995702767186

ENSMUST00000000171 948 Pih1d2 0.16511325549963585

ENSMUST00000000275 1443 Glra3 0.2009545411281503

ENSMUST00000000260 1038 Gmpr 0.3114861318780843

ENSMUST00000000201 5220 Nalcn 0.20636543168491903

ENSMUST00000000193 447 Ccl2 0.25886689765290793

ENSMUST00000000299 2313 Itgb2 0.31806957816564874

ENSMUST00000000285 1695 Dgke 0.22818078021410443

ENSMUST00000000304 600 Hddc2 0.22511381938926373

ENSMUST00000000287 1359 Scpep1 0.26166444174111364

ENSMUST00000000206 1437 Btbd17 0.3304136761576877

ENSMUST00000000266 1338 Ifi202b 0.17222686536840157

ENSMUST00000000291 1776 Mnt 0.32576091850629607

ENSMUST00000000221 1968 Scnn1g 0.3008775227533285

ENSMUST00000000188 870 Ccnd2 0.3491078584213302

ENSMUST00000000194 315 Ccl12 0.24147290769480978

ENSMUST00000000253 1221 Lhx2 0.317078780358627

ENSMUST00000000208 1809 Slfn4 0.18784018202202446

ENSMUST00000000199 573 Ncs1 0.37692430763572066

ENSMUST00000000254 810 Clec2g 0.24800415176054905

ENSMUST00000000187 627 Fgf6 0.30572408020959263

ENSMUST00000000305 615 Tpd52l1 0.22799996996592606

ENSMUST00000000219 1497 Th 0.30818249522203134

ENSMUST00000000544 1518 Acvr1b 0.2816129571943484

ENSMUST00000000365 546 Mcts1 0.13320745458543448

ENSMUST00000000395 1473 Tmprss2 0.24296305301032575

ENSMUST00000000356 507 Dazap2 0.21416152683763795

ENSMUST00000000574 960 Adora3 0.2591433973871783

ENSMUST00000000421 807 Tssk3 0.2919034021654611

ENSMUST00000000449 1251 Mkrn2 0.26113777570308605

ENSMUST00000000430 1680 Galnt1 0.18736371656280063

ENSMUST00000000412 1653 Egfl6 0.22448178024451995

ENSMUST00000000329 1992 Alox12 0.2736742946783161

ENSMUST00000000326 1425 Bcl6b 0.2428218893998855

ENSMUST00000000542 1509 Acvrl1 0.31504570528797005

ENSMUST00000000310 600 Pemt 0.3404998513004287

ENSMUST00000000514 1125 Serpinb8 0.2256599348860428

ENSMUST00000000348 1101 Rtca 0.21509130377734204

ENSMUST00000000327 918 Clec10a 0.31007193602262145

ENSMUST00000000573 2166 Ovgp1 0.21727990057093774

ENSMUST00000000335 798 Comt 0.32795546204498094

ENSMUST00000000369 894 Rem1 0.3249522625618602

ENSMUST00000000342 294 Ccl11 0.32746972438306

ENSMUST00000000312 2655 Cdh1 0.2873091920299752

ENSMUST00000000349 1449 Dbt 0.20748620751440666

ENSMUST00000000641 2334 Sema4f 0.2664488923390876

ENSMUST00000000619 2244 Clcn4-2 0.2796954256482985

ENSMUST00000000445 768 Myf5 0.3484592591841241

ENSMUST00000000590 1752 Rdx 0.1904933435915322

ENSMUST00000000388 1362 Ccm2 0.2905148756298323

ENSMUST00000000476 3270 Pdgfra 0.2751103294025408

ENSMUST00000000314 2742 Cdh4 0.3246262107514628

ENSMUST00000000466 1278 Plin2 0.29135144970519805

ENSMUST00000000594 426 C1d 0.19212190911113655

ENSMUST00000000543 1179 Grasp 0.3018178955870399

ENSMUST00000000451 1947 Raf1 0.26146648779629633

ENSMUST00000000608 582 Gm2a 0.29314802399294915

ENSMUST00000000317 1257 Ckmt1 0.27064724839878296

ENSMUST00000000450 1518 Pparg 0.26771941956124484

ENSMUST00000000500 726 Pdgfb 0.3331078601964364

ENSMUST00000000384 3777 Trappc10 0.2811220526282175

ENSMUST00000000505 2160 Mcm7 0.2561453771564318

ENSMUST00000000579 1524 Sox9 0.3752281235497891

ENSMUST00000000704 675 Hoxb6 0.34932016839153435

ENSMUST00000000769 1254 Serpinf1 0.30823406316641505

ENSMUST00000000707 2265 Loxl3 0.29927958362906276

ENSMUST00000001081 1182 Rmnd5b 0.3389083468265093

ENSMUST00000000717 366 Tcl1b5 0.22353692152561527

ENSMUST00000000746 1266 Dnmt3l 0.30746838054492764

ENSMUST00000000958 1782 Top1mt 0.3264701149948943

ENSMUST00000001043 675 Chtop 0.18330762087568309

ENSMUST00000001040 789 Icam4 0.26289991034568755

ENSMUST00000000985 1302 Oxa1l 0.2619880730540474

ENSMUST00000000804 1989 Ddx3x 0.17509688208691068

ENSMUST00000001027 4002 Aox1 0.22593395461075833

ENSMUST00000000767 1935 Rpa1 0.2571519653245276

ENSMUST00000000727 648 Rab5b 0.23289009248635886

ENSMUST00000000793 1197 Polr3d 0.29436023220484275

ENSMUST00000000674 1527 St7 0.1887236294661274

ENSMUST00000001130 573 Sebox 0.25318942730214283

ENSMUST00000000895 1062 Necab3 0.2855820738418666

ENSMUST00000000828 1848 Txnrd3 0.22113872718952032

ENSMUST00000000924 1479 Mmp11 0.2824472435215164

ENSMUST00000000896 639 Pxmp4 0.34431231183155

ENSMUST00000000808 2217 Il12rb1 0.30976252657342845

ENSMUST00000001042 1173 Ilf2 0.21114060498700185

ENSMUST00000001148 1116 Pcbp3 0.2991348013951375

ENSMUST00000000925 1158 Smarcb1 0.35180412945886014

ENSMUST00000000964 1011 Hoxa1 0.2637497018161434

ENSMUST00000000844 621 Tpd52l2 0.2510503832581536

ENSMUST00000000811 2199 Kcnn3 0.31285002821987273

ENSMUST00000000809 1857 Slc5a5 0.316342070660137

ENSMUST00000000646 2934 Sez6 0.302083890921496

ENSMUST00000001036 3624 Tyk2 0.332012656099373

ENSMUST00000001008 279 Ccl3 0.26254479969349687

ENSMUST00000000718 363 Tcl1b4 0.23933792189162326

ENSMUST00000001080 1614 N4bp3 0.3272513393707853

ENSMUST00000001002 3555 Heatr6 0.22533301141423687

ENSMUST00000001092 1845 Zfp276 0.3203386525002041

ENSMUST00000001125 951 Kctd10 0.37216482214411134

ENSMUST00000001127 1107 Poldip2 0.2391976701999108

ENSMUST00000001147 3078 Col6a1 0.2795814109928869

ENSMUST00000000901 2454 Dlg3 0.27576569205331086

ENSMUST00000000755 876 Sult5a1 0.33797564984469586

ENSMUST00000001112 1725 Grk4 0.18076810526214346

ENSMUST00000001051 270 S100a6 0.37125328130255314

ENSMUST00000000984 1533 Slc7a7 0.27775827300626443

ENSMUST00000001122 1761 Slc13a2 0.3040231111063327

ENSMUST00000000642 2754 Hk2 0.3430501186837865

ENSMUST00000000756 636 Rpl13 0.3432854816922492

ENSMUST00000001009 225 Wfdc18 0.2420551510205776

ENSMUST00000000940 1398 Nsun5 0.2773597020843164

ENSMUST00000000939 3207 Hip1r 0.36425316938035934

ENSMUST00000001047 306 S100a3 0.3583555295015979

ENSMUST00000001115 1770 Grk6 0.3193697074616459

ENSMUST00000000724 2442 Kat2b 0.24741668245834128

ENSMUST00000001046 306 S100a4 0.3777688553889505

ENSMUST00000001055 834 Icam2 0.2738436429599336

ENSMUST00000000687 861 Haao 0.3064105783871252

ENSMUST00000000776 2718 Tubgcp3 0.23935324494676757

ENSMUST00000000889 423 Il4 0.2399031586174329

ENSMUST00000000910 1869 Dbh 0.33191642011268585

ENSMUST00000001109 1371 Mfsd10 0.28829476198654264

ENSMUST00000000881 2322 Bcl11a 0.263846332710839

ENSMUST00000000759 591 Chmp1a 0.3954321560603359

ENSMUST00000000926 372 Vpreb3 0.3132427619570965

ENSMUST00000001126 1380 Slc46a1 0.30414507926345113

ENSMUST00000001063 1788 Epn2 0.26043702707287925

ENSMUST00000000894 1158 E2f1 0.29085005636388084

ENSMUST00000001108 1992 Add1 0.23880337289121192

ENSMUST00000001059 2934 Ern1 0.2996437107723363

ENSMUST00000000834 840 Fasl 0.2213770766103141

ENSMUST00000001079 3756 Sec24b 0.20426121046480064

ENSMUST00000001155 1815 Araf 0.30764008323870423

ENSMUST00000000696 225 Cd52 0.2042968345319086

ENSMUST00000001701 915 Hoxc11 0.3250557380608619

ENSMUST00000001836 1506 Pwp1 0.21250754594563007

ENSMUST00000001675 1395 Stk38l 0.21541631477658538

ENSMUST00000001451 3054 Smg5 0.2919977376183897

ENSMUST00000001920 453 Aif1l 0.33943448885549243

ENSMUST00000001834 1518 Rtcb 0.23001094699246336

ENSMUST00000001561 690 Nrip2 0.3180140406000042

ENSMUST00000001326 2346 Sp1 0.21781412918597934

ENSMUST00000001802 2220 Naglu 0.3302832054952852

ENSMUST00000001716 357 Ddt 0.30272530295407474

ENSMUST00000001520 2370 Afg3l1 0.25828242240444715

ENSMUST00000001592 2238 Jup 0.38657838768919184

ENSMUST00000001507 1512 Cyp51 0.20353970567513996

ENSMUST00000002084 2211 Abcd1 0.2906454651813919

ENSMUST00000002090 519 Ssr4 0.2613322924934609

ENSMUST00000001319 1011 Efnb2 0.22713182318043124

ENSMUST00000001900 1032 Zdhhc4 0.2809723614139139

ENSMUST00000001927 5139 Ltbp1 0.26428307333816703

ENSMUST00000001964 2199 Ace 0.3253019380941284

ENSMUST00000001652 1179 Bdkrb2 0.41263269196752517

ENSMUST00000001166 2142 Cnnm3 0.32899297833475566

ENSMUST00000002080 2319 Pdzd4 0.3214388155423593

ENSMUST00000001975 1545 Nacc1 0.31806633716470656

ENSMUST00000002048 885 Taco1 0.2534285934353037

ENSMUST00000001878 807 Hoxd12 0.3035921691481283

ENSMUST00000001186 468 Snrnp27 0.21735289879788777

ENSMUST00000001722 1158 Marveld3 0.28038182706975584

ENSMUST00000001565 1392 Gtf2h4 0.317769791346074

ENSMUST00000001812 2382 Smo 0.3277727975746202

ENSMUST00000001497 660 Cideb 0.2771958996610564

ENSMUST00000001183 1626 Ftcd 0.30895523659809515

ENSMUST00000001456 1176 Tmem79 0.2901926616451608

ENSMUST00000001672 1350 Ifrd1 0.175585178223948

ENSMUST00000001485 789 Mrpl10 0.2658126789715642

ENSMUST00000001620 2034 Fxr1 0.1508360032584281

ENSMUST00000001699 1029 Hoxc10 0.27904188955477705

ENSMUST00000001569 1287 Flot1 0.331658063820116

ENSMUST00000001242 801 D10Jhu81e 0.296247907766895

ENSMUST00000001202 2703 Ocrl 0.19731963025952765

ENSMUST00000001984 705 Ceacam9 0.244129053266105

ENSMUST00000001825 996 Chordc1 0.1556212546263175

ENSMUST00000001416 1530 Hars 0.25782437573328715

ENSMUST00000002008 987 Vsig2 0.2839001895268591

ENSMUST00000001806 1692 Coasy 0.2922381420226854

ENSMUST00000001700 987 Hoxc13 0.33100371736922873

ENSMUST00000001921 1254 Cpa3 0.19568342422434798

ENSMUST00000001599 1827 Klhl10 0.2754704523949023

ENSMUST00000001882 768 Folr1 0.33006031703991795

ENSMUST00000001412 2895 Ccdc132 0.18349736030190203

ENSMUST00000001544 2382 Vwa5a 0.22222061262893222

ENSMUST00000001460 2733 Snd1 0.2983910763437343

ENSMUST00000001179 8751 Pcnt 0.2757206605157031

ENSMUST00000001713 723 Gstt1 0.3123830205444101

ENSMUST00000001703 729 Hoxc8 0.29763118289717283

ENSMUST00000001172 552 Ankrd39 0.3200455964246925

ENSMUST00000001963 3939 Ace 0.32004363637869593

ENSMUST00000001631 2223 Acap1 0.2657371705358072

ENSMUST00000001562 1383 Tulp3 0.30102788700255895

ENSMUST00000001187 960 Anxa4 0.294070339250171

ENSMUST00000001720 1365 Tat 0.2856305854579667

ENSMUST00000002044 1881 Map3k3 0.2655086484074632

ENSMUST00000001402 1314 Fbxo9 0.24923626656451847

ENSMUST00000001790 684 Cldn15 0.33963134003995415

ENSMUST00000001254 2274 Slc26a3 0.19112274960715392

ENSMUST00000001181 3105 Col6a2 0.31501411662937034

ENSMUST00000001480 2763 Npepps 0.1915439554273337

ENSMUST00000001339 846 Rrp15 0.26533367889349035

ENSMUST00000002043 1452 Ccdc47 0.22797929961032226

ENSMUST00000001566 1335 Tubb5 0.3442279057463324

ENSMUST00000002081 1698 Srpk3 0.28898668803570693

ENSMUST00000001279 1785 Lsr 0.27898235466009685

ENSMUST00000001455 1524 Mef2d 0.31063539097023973

ENSMUST00000002079 5709 Plxnb3 0.27753755987608736

ENSMUST00000001253 2343 Slc26a4 0.2196620217842738

ENSMUST00000002029 780 Emd 0.24725737360354996

ENSMUST00000001845 807 Capns1 0.29543840715550085

ENSMUST00000001513 1344 Tubb6 0.4151858174070709

ENSMUST00000001626 2001 Tnk1 0.2581978745955123

ENSMUST00000001327 2421 Itgb7 0.28256677384201717

ENSMUST00000002087 1032 Pnck 0.31611941713197816

ENSMUST00000001715 726 Gstt3 0.3302395851433707

ENSMUST00000001559 1332 Itfg2 0.3010030263827481

ENSMUST00000001792 3969 Il16 0.26177216302204676

ENSMUST00000001711 708 Hoxc6 0.28237876716576427

ENSMUST00000001809 1911 Pabpc1 0.19626715602179196

ENSMUST00000001965 2853 Kcnh6 0.32725832718781067

ENSMUST00000002011 1185 Esam 0.24446240195896704

ENSMUST00000001611 2565 Nom1 0.23976896308696477

ENSMUST00000001280 2169 Gramd1a 0.318656501860457

ENSMUST00000002007 1626 Siae 0.22549654549085135

ENSMUST00000001384 894 Cnn1 0.34394715521939623

ENSMUST00000001724 1983 Ddx18 0.23972760337406676

ENSMUST00000002091 738 Bcap31 0.25294897767653624

ENSMUST00000001185 1575 Gmcl1 0.21683174534815963

ENSMUST00000001548 3162 Itga3 0.31068487929620375

ENSMUST00000001950 825 Tollip 0.315189189246324

ENSMUST00000001989 3177 Uba1 0.25680067765132586

ENSMUST00000002063 3369 Ap4e1 0.15803176076850556

ENSMUST00000002064 888 Blvra 0.2289815679617755

ENSMUST00000002053 1785 Npas1 0.2877225455853952

ENSMUST00000001258 2349 Uhrf1 0.33336694347952733

ENSMUST00000001484 1593 Tbx21 0.3096403892277488

ENSMUST00000001479 2631 Kpnb1 0.22216494559044506

ENSMUST00000001534 636 Sost 0.3629301940012023

ENSMUST00000001256 2661 Sema6b 0.30516646476876347

ENSMUST00000002025 1884 Tktl2 0.22155975928073973

ENSMUST00000001536 1119 Nkx2-1 0.31930543232468006

ENSMUST00000002013 450 Spa17 0.20155139541881076

ENSMUST00000001547 4362 Col1a1 0.23227966094021854

ENSMUST00000001583 1920 Ell2 0.19948810381745125

ENSMUST00000001184 684 Mxd1 0.3216404404527412

ENSMUST00000001757 525 Eef1e1 0.22234821644693495

ENSMUST00000001415 1461 Sra1 0.2602623553231407

ENSMUST00000001712 6564 Cabin1 0.30350289708333034

ENSMUST00000001884 2034 Clpb 0.29468574771059236

ENSMUST00000001304 1146 Ckb 0.38312138371773435

ENSMUST00000001709 669 Hoxc5 0.3099143097074227

ENSMUST00000001801 2505 Tcirg1 0.3532817841261572

ENSMUST00000001872 1020 Hoxd13 0.26324880628779185

ENSMUST00000001824 2259 Folh1 0.1722770948345893

ENSMUST00000001419 600 Zmat2 0.3193060264598492

ENSMUST00000001595 1746 Fkbp10 0.33421408913384143

ENSMUST00000001818 2073 Crnkl1 0.22309474675880903

ENSMUST00000001854 1593 Slc7a10 0.3181162585833715

ENSMUST00000001347 684 Rnd2 0.3027413155511864

ENSMUST00000001522 1383 Def8 0.37379632630558385

ENSMUST00000001538 1029 Pax9 0.3267754752799342

ENSMUST00000001706 783 Hoxc9 0.3224715150821545

ENSMUST00000001247 1107 Rnf32 0.18374656683141916

ENSMUST00000001780 1443 Akt1 0.39983777115006525

ENSMUST00000001452 1638 Cct3 0.2353154451523176

ENSMUST00000001156 1395 Cfp 0.26757804117003803

ENSMUST00000003717 3831 Abcb4 0.22557568809227765

ENSMUST00000003569 795 Inmt 0.34828818935199957

ENSMUST00000003444 1482 Ccdc65 0.32112638480070527

ENSMUST00000003529 1608 Paf1 0.2793167242605739

ENSMUST00000002289 693 Uchl3 0.16167214301914354

ENSMUST00000002099 906 Ift46 0.2852489262924054

ENSMUST00000002599 1644 Puf60 0.2859215674396469

ENSMUST00000002625 852 Uck1 0.33333897165073817

ENSMUST00000004428 2241 Clcn5 0.22342781595766778

ENSMUST00000002436 1788 Snx9 0.26091678762863774

ENSMUST00000003369 1500 Plag1 0.21523434779063452

ENSMUST00000003659 2268 Comp 0.3258419024977422

ENSMUST00000003575 747 Tpm4 0.3017847222197793

ENSMUST00000002837 690 Tmed5 0.19103751463682853

ENSMUST00000003599 2793 Pcdhgb6 0.2303732558408318

ENSMUST00000002400 495 Mdp1 0.2036850400220842

ENSMUST00000002350 1431 Narfl 0.284684656901381

ENSMUST00000004094 1086 Ssbp2 0.1923551165923649

ENSMUST00000003156 507 Atp5d 0.2793462278007173

ENSMUST00000002640 2148 Scin 0.23834446972629172

ENSMUST00000002379 783 Cd320 0.2540382074934132

ENSMUST00000003912 1251 Calr 0.30338019565977487

ENSMUST00000003416 1575 Cyp4f16 0.3008769940576784

ENSMUST00000003029 1359 Timm44 0.27041173756410064

ENSMUST00000004145 2382 Stat5a 0.4024377479354072

ENSMUST00000003238 1698 Foxj2 0.32612274877293296

ENSMUST00000003705 1029 Aven 0.2317208386999336

ENSMUST00000002360 1233 Angptl4 0.28317922840367926

ENSMUST00000003370 1575 Hck 0.3455515716808127

ENSMUST00000002292 1176 Rmnd5a 0.1809242158478327

ENSMUST00000002198 2376 Sf3a1 0.29556619704647163

ENSMUST00000002176 1455 Celf2 0.2360077747654778

ENSMUST00000002765 2757 Prkd1 0.22831290526521333

ENSMUST00000003620 1800 Prodh 0.3285003618732302

ENSMUST00000004137 657 Gstm7 0.3681723397090561

ENSMUST00000004057 468 Fam162a 0.26802975446061883

ENSMUST00000003310 9162 Ranbp2 0.14946728296973263

ENSMUST00000004281 1800 Dyrk2 0.2872739140268355

ENSMUST00000003183 1500 Ppp5c 0.35563676089005647

ENSMUST00000003554 1218 Casq1 0.3010805855901146

ENSMUST00000002757 321 Cox16 0.14483295796085777

ENSMUST00000002677 2667 Axl 0.28924681332337276

ENSMUST00000002825 4440 Baz1b 0.23999850043014437

ENSMUST00000002655 348 Mien1 0.3070616225610322

ENSMUST00000002172 1272 Acp2 0.25588469684419396

ENSMUST00000003961 3585 Ppfia3 0.30465770009396276

ENSMUST00000003049 2238 Med25 0.31035957505744705

ENSMUST00000002964 2175 Adgre5 0.30872862733049794

ENSMUST00000002275 1362 Vrk3 0.3272071977418366

ENSMUST00000004072 774 Rpl8 0.2962785710647749

ENSMUST00000003574 1575 Cyp4f18 0.3026685648770252

ENSMUST00000003754 816 Calb2 0.3353139138835103

ENSMUST00000003038 2817 Ap2a2 0.28008678524346287

ENSMUST00000004208 1482 Angptl2 0.35926701459876276

ENSMUST00000002529 2826 Prpf6 0.24950164952703316

ENSMUST00000003720 1839 Crot 0.16080290369796135

ENSMUST00000003451 699 Rnd1 0.2782112085680453

ENSMUST00000003642 1860 Klc4 0.3478866451104478

ENSMUST00000002881 669 Nudt14 0.30450122332500734

ENSMUST00000003284 1260 Irf3 0.31152360256496686

ENSMUST00000002855 639 Kdelr1 0.30929084028021536

ENSMUST00000003345 2061 Amph 0.24141725089453195

ENSMUST00000002790 2916 Cse1l 0.1723476984830724

ENSMUST00000002808 942 Prkra 0.20578684497910676

ENSMUST00000002284 984 Plaur 0.33597573967629

ENSMUST00000004156 2553 Map3k11 0.29219833331409145

ENSMUST00000003971 624 Lin7b 0.36538309419262044

ENSMUST00000003964 2217 Gys1 0.31575878859888556

ENSMUST00000002101 549 Cd3g 0.20808795541931027

ENSMUST00000004140 657 Gstm1 0.3924941818552922

ENSMUST00000002457 1200 Bmp8b 0.2853105167046547

ENSMUST00000003501 1104 Elavl3 0.38100610101170845

ENSMUST00000002310 1035 Prr27 0.1769402023791838

ENSMUST00000002678 1173 Tgfb1 0.30946727845794514

ENSMUST00000003115 594 Cdkn1b 0.28275944794311464

ENSMUST00000002397 1047 Gmpr2 0.2320423947366953

ENSMUST00000002925 858 Timmdc1 0.2133877012952241

ENSMUST00000002518 594 Aes 0.3662413223540789

ENSMUST00000002976 2595 Il17ra 0.3136796561245618

ENSMUST00000004143 2361 Stat5b 0.382124405924284

ENSMUST00000003207 2409 Lipe 0.28737497281518304

ENSMUST00000003312 1347 Edar 0.3154565771950624

ENSMUST00000004326 5619 Plxna3 0.29010776594186216

ENSMUST00000003628 2736 Ddr1 0.32924536180816627

ENSMUST00000004379 735 Emg1 0.24980897058686433

ENSMUST00000003137 1473 Cyp2c29 0.21539925713363836

ENSMUST00000003527 3249 Supt5 0.3169714297028044

ENSMUST00000002809 657 Fkbp7 0.18815913638104367

ENSMUST00000002883 1056 Sfrp4 0.28669942449780317

ENSMUST00000003438 654 Mob3a 0.3881275368410399

ENSMUST00000003843 1968 Man1a 0.23039033158615166

ENSMUST00000002805 567 Naa20 0.18643310834168633

ENSMUST00000002735 819 Clpp 0.31349134033269205

ENSMUST00000003268 1107 Sh3gl1 0.3690011305118792

ENSMUST00000002845 525 Mea1 0.2361064472091939

ENSMUST00000002502 3012 Hltf 0.14879125462345422

ENSMUST00000003274 687 Ebi3 0.3114608804164841

ENSMUST00000004378 1305 Eno2 0.3142216765830325

ENSMUST00000002737 666 Alkbh7 0.2772475213758839

ENSMUST00000004343 1083 Wars2 0.24319844403006566

ENSMUST00000003621 1443 Dgcr14 0.2811203895482604

ENSMUST00000003319 2130 Abcf3 0.2509014507873346

ENSMUST00000003413 1599 Cyp4f39 0.31613124539082993

ENSMUST00000004396 360 Atp6v1f 0.32309629587626726

ENSMUST00000002127 723 Unc119 0.33220380246210784

ENSMUST00000003450 2460 Ddx23 0.307781451745991

ENSMUST00000002469 660 Ocel1 0.34067159052046403

ENSMUST00000002926 1371 Pla1a 0.24864858639056386

ENSMUST00000003313 1773 Grk5 0.27172390121201434

ENSMUST00000003035 4566 Disp1 0.31229593872387706

ENSMUST00000003017 1602 Tbxas1 0.2715869370750586

ENSMUST00000003121 624 Rab8a 0.3239168124252982

ENSMUST00000002495 1137 Meis3 0.31628218409721803

ENSMUST00000003469 663 Cd79a 0.29242613589823024

ENSMUST00000003645 897 Ercc1 0.32205714599871427

ENSMUST00000003910 1062 Dnase2a 0.2703520598882389

ENSMUST00000003152 1311 Stk11 0.3677559001730645

ENSMUST00000002839 1785 Ppp2r5d 0.31702512324973087

ENSMUST00000002663 1068 Pon1 0.17026360185433784

ENSMUST00000003459 3075 Myo1g 0.31973945825337513

ENSMUST00000003493 1566 Prkcsh 0.31542382531101915

ENSMUST00000003857 2115 Shkbp1 0.27510314675989517

ENSMUST00000003826 1470 Htr3a 0.3183632661748172

ENSMUST00000002923 1089 Adprh 0.2899251940095351

ENSMUST00000002413 1443 Tmem161a 0.3524642061155101

ENSMUST00000003714 3258 Cp 0.18068233979468706

ENSMUST00000002327 1893 Def6 0.39847132098189125

ENSMUST00000003643 1146 Ckm 0.4484456389745236

ENSMUST00000003850 2037 Itpkc 0.29124952928360154

ENSMUST00000003290 768 Bcl2l12 0.26407894989593034

ENSMUST00000003860 1602 Adck4 0.2979197935947097

ENSMUST00000002280 1563 Smg9 0.3405404720822339

ENSMUST00000003434 1572 Btbd2 0.3718150956238153

ENSMUST00000002710 1095 Pdcd2l 0.2411147511316583

ENSMUST00000002708 1314 Shh 0.33103533121382506

ENSMUST00000003513 906 Nosip 0.348748987436074

ENSMUST00000002145 1017 Hsf2bp 0.21691333297540916

ENSMUST00000003726 4206 Brd4 0.2983491373256109

ENSMUST00000002880 1620 Btbd6 0.3049991778911409

ENSMUST00000004054 1617 Kpna1 0.1908010832406571

ENSMUST00000003710 2637 Aak1 0.22993661845509317

ENSMUST00000002588 4776 Tiam1 0.2981846614621278

ENSMUST00000002318 1707 Zfp523 0.32873522369682806

ENSMUST00000002551 360 Snrpd1 0.15636111472850184

ENSMUST00000003027 1407 Map2k7 0.32543964805722453

ENSMUST00000003681 1212 Sec14l2 0.33457026627279

ENSMUST00000002914 2736 Chaf1a 0.29670617247145786

ENSMUST00000004294 2379 Kifc2 0.2598718822612303

ENSMUST00000003071 375 Apoc4 0.36048740732457646

ENSMUST00000003677 1140 Rnf215 0.27023000879919107

ENSMUST00000003117 1272 Ap1m1 0.34385772744420234

ENSMUST00000002572 1014 Slc9a3r2 0.3227329275707662

ENSMUST00000002699 2064 Akap8 0.25133351109070745

ENSMUST00000004375 900 Phb2 0.3251577887599673

ENSMUST00000002403 942 Dhrs1 0.2505661031982928

ENSMUST00000003386 885 Mrpl4 0.27504259349528243

ENSMUST00000003907 1344 Gcdh 0.27028120902927444

ENSMUST00000004232 1128 Adh1 0.23685281030231367

ENSMUST00000003318 2151 Dvl3 0.3283274497697275

ENSMUST00000003818 678 Insig2 0.18314756752651828

ENSMUST00000003509 1197 St8sia6 0.2513807074880575

ENSMUST00000003597 1431 Psg18 0.19098830742382067

ENSMUST00000003759 1020 Ciao1 0.2664232546613278

ENSMUST00000002274 1260 Napsa 0.2607001034510381

ENSMUST00000002445 1476 Ranbp3 0.2991187937566229

ENSMUST00000004050 717 Mmd 0.2389002218356825

ENSMUST00000003219 1518 Cnot11 0.24307585345658994

ENSMUST00000002889 3816 Flii 0.3344969438910288

ENSMUST00000004076 2640 Grm3 0.2401427638488735

ENSMUST00000003442 1455 Cacnb3 0.3240410555851117

ENSMUST00000003360 987 Car11 0.3099249900821483

ENSMUST00000003461 3072 Ogdh 0.2914134338263291

ENSMUST00000002456 1722 Nt5c1b 0.2136599368439572

ENSMUST00000003741 2175 Rps6ka1 0.3311877772958147

ENSMUST00000003536 600 Med29 0.29450143469611006

ENSMUST00000003687 1239 Tgfb3 0.3233203835082888

ENSMUST00000004206 963 Eif3g 0.33628175902301993

ENSMUST00000003044 1569 Pnkp 0.2737137986900195

ENSMUST00000002846 882 Gnmt 0.33088621014413216

ENSMUST00000002395 1776 Rec8 0.261588445591487

ENSMUST00000002532 651 Rgs19 0.2893321177504648

ENSMUST00000004222 3048 Hira 0.2403701198265447

ENSMUST00000003550 2127 Ncstn 0.25756308548055734

ENSMUST00000003154 630 Efna2 0.38260707726397186

ENSMUST00000003572 2190 Gars 0.229435564724529

ENSMUST00000003640 1017 Fosb 0.28951743074229475

ENSMUST00000003521 477 Rps11 0.3049272985497205

ENSMUST00000002979 5505 Lamb1 0.25967524468069136

ENSMUST00000004201 5220 Col5a3 0.2431208038931805

ENSMUST00000003635 483 Ier3 0.26724420745056165

ENSMUST00000002298 1524 Ppm1j 0.29580530787233694

ENSMUST00000002473 1002 Babam1 0.33718485223765043

ENSMUST00000003397 1272 Ap1m2 0.32634785044211745

ENSMUST00000003445 606 Fkbp11 0.2503123405536426

ENSMUST00000004173 627 Cdip1 0.31648328574710005

ENSMUST00000002850 4497 Abcc6 0.3378075283572817

ENSMUST00000003906 1527 Farsa 0.3341665967704077

ENSMUST00000002128 780 Rab34 0.2995782268557616

ENSMUST00000004036 1023 Efnb3 0.26989274515348677

ENSMUST00000002902 1212 Qtrt1 0.3099570696741667

ENSMUST00000004134 675 Gstm5 0.31404058849453653

ENSMUST00000002740 471 Pspn 0.2677524936118916

ENSMUST00000004327 1548 G6pdx 0.31040532666131776

ENSMUST00000002487 2415 Braf 0.1954750808546809

ENSMUST00000003203 3777 Cdk12 0.1981573797165525

ENSMUST00000004203 1413 Ppan 0.3382717253042131

ENSMUST00000003946 1212 Nob1 0.3084050790700015

ENSMUST00000002121 5181 Supt6 0.28810206817625933

ENSMUST00000002444 2154 Rfx2 0.3654649962376864

ENSMUST00000004381 1464 Lpcat3 0.27509797746993386

ENSMUST00000003568 1293 Crhr2 0.34833512522034477

ENSMUST00000002603 4998 Scrib 0.2909058217292994

ENSMUST00000002924 1461 Tmem39a 0.20766433922523606

ENSMUST00000002368 1989 Dbf4 0.1637773920273543

ENSMUST00000002095 11892 Kmt2a 0.2319593387026033

ENSMUST00000002152 582 Bbc3 0.32632395633126005

ENSMUST00000003512 1098 Fcgrt 0.27274730015827453

ENSMUST00000003911 1089 Rad23a 0.29500601738672017

ENSMUST00000002100 1098 Tmem25 0.2471257450838637

ENSMUST00000004097 4281 Cux1 0.28124510876059133

ENSMUST00000004172 948 Hmox2 0.26690998260588894

ENSMUST00000002533 720 Rgs20 0.2530531146244054

ENSMUST00000004133 2352 Brinp2 0.2973371169936028

ENSMUST00000003468 2940 Grik5 0.3703292352937122

ENSMUST00000003870 888 Mxi1 0.27441087102263306

ENSMUST00000004330 1293 Ikbkg 0.327519537337816

ENSMUST00000002389 2448 Tgm1 0.2748545118622086

ENSMUST00000002840 2946 Pex6 0.27312506957356847

ENSMUST00000002180 819 Spi1 0.3694761346582131

ENSMUST00000002885 675 Epdr1 0.3344416659924115

ENSMUST00000004392 1494 Irf5 0.3161245006825543

ENSMUST00000004051 888 Hlf 0.2969657358158741

ENSMUST00000002418 363 2310045N01Rik 0.3763560702471115

ENSMUST00000003561 993 Phyhip 0.4416604020739416

ENSMUST00000002177 1338 Nr1h3 0.32119976317699733

ENSMUST00000002305 2823 Kdm7a 0.1836300102367736

ENSMUST00000003612 558 Dusp3 0.34681362037200636

ENSMUST00000004136 657 Gstm3 0.3307922251708593

ENSMUST00000002112 480 Trappc6a 0.41096149946254423

ENSMUST00000002398 3234 Adcy4 0.2983177181317263

ENSMUST00000003061 1869 Bcam 0.2993239757775391

ENSMUST00000002412 3807 Ncan 0.28381536853989664

ENSMUST00000002133 660 Sdf2 0.24913599157886523

ENSMUST00000002452 432 Ndufa11 0.3156480582085629

ENSMUST00000002291 3171 Paxip1 0.2655439905063104

ENSMUST00000003395 1833 Pde4a 0.3260571245062175

ENSMUST00000002844 921 Mrpl2 0.262542196129485

ENSMUST00000002818 597 Ykt6 0.2490376559190885

ENSMUST00000003981 1380 Il7r 0.16828628511939533

ENSMUST00000002683 1023 Ccdc97 0.3501013033759113

ENSMUST00000004389 381 Grcc10 0.30170173146673845

ENSMUST00000002911 2010 Hdgfrp2 0.3075824766066922

ENSMUST00000003947 825 Nqo1 0.27253904875931473

ENSMUST00000002466 1173 Nr2f6 0.36012270672274443

ENSMUST00000002756 588 Med6 0.1781672576419479

ENSMUST00000003622 936 Slc25a1 0.28944637521355016

ENSMUST00000002891 3012 Top3a 0.2644811008520103

ENSMUST00000003655 1131 As3mt 0.21776688278244913

ENSMUST00000002320 1323 Ppard 0.4267813603189775

ENSMUST00000003808 912 Gdf15 0.2971702568146826

ENSMUST00000002336 1740 Zim1 0.27047047328507046

ENSMUST00000003876 2856 Brd8 0.20459408523013634

ENSMUST00000004202 4863 Dnmt1 0.30214717707993716

ENSMUST00000003436 933 Abhd17a 0.3614827663810133

ENSMUST00000002549 1236 Abhd3 0.24370732710656398

ENSMUST00000004120 1128 Fancl 0.18787866261517158

ENSMUST00000003123 339 Fam32a 0.40972825284419184

ENSMUST00000004200 690 Cwc15 0.22001158393278733

ENSMUST00000002848 3972 Grin2d 0.32580566165567915

ENSMUST00000002733 1527 Gtf2f1 0.35449106961763116

ENSMUST00000003762 1752 Has1 0.2868689793064896

ENSMUST00000003100 1476 Cyp2f2 0.3307600745649214

ENSMUST00000002989 1224 Arrdc2 0.3115448653346742

ENSMUST00000003135 1176 Elf3 0.3409387171479089

ENSMUST00000003898 2292 Ece2 0.2879712816303906

ENSMUST00000003320 2154 Eif2b5 0.2396722679000465

ENSMUST00000007803 702 Bcl2l1 0.2865327571873223

ENSMUST00000009143 1293 Bmp7 0.36838106293508804

ENSMUST00000009877 1509 Tfcp2 0.26249808402209657

ENSMUST00000011934 80661 Ttn 0.19597269964263123

ENSMUST00000010239 1581 Slc3a2 0.2632134763624798

ENSMUST00000009220 513 Zmat5 0.3865925980742195

ENSMUST00000012580 3009 Hps3 0.17222060743857462

ENSMUST00000006462 1308 Aamp 0.2935633065341379

ENSMUST00000005651 2037 Por 0.3497624503644274

ENSMUST00000007318 1251 Krt31 0.4413617639952223

ENSMUST00000006217 777 Snf8 0.30095227086923565

ENSMUST00000005238 1251 Slc2a9 0.2999763755393851

ENSMUST00000005509 867 Stx1a 0.42606941147371635

ENSMUST00000005057 2064 Thop1 0.40010279077994226

ENSMUST00000010941 1083 Wnt2 0.31249306073657457

ENSMUST00000010502 861 Ifi35 0.2763830464694067

ENSMUST00000012440 1620 Tjap1 0.30620388109424335

ENSMUST00000004920 3114 Ulk2 0.20059940677277582

ENSMUST00000008052 1032 Hmgcll1 0.17272542478299663

ENSMUST00000011733 1491 Fsd1 0.36317949495095525

ENSMUST00000004673 1116 Ndrg2 0.2533286408528185

ENSMUST00000008094 1320 Abhd8 0.34528392286515586

ENSMUST00000012587 3159 Kif11 0.16152720854484365

ENSMUST00000008684 468 Mgst1 0.27021648136487536

ENSMUST00000006254 735 Tbcb 0.2716128782952136

ENSMUST00000004576 1659 Tbccd1 0.22689160011075976

ENSMUST00000005103 1983 Nfe2l3 0.1863843511438901

ENSMUST00000013807 1212 Pten 0.1835396727917331

ENSMUST00000006392 1134 Serpinb9b 0.22913217181476891

ENSMUST00000008605 1134 Fut1 0.27669928064790994

ENSMUST00000013842 393 Pea15a 0.3498264734143745

ENSMUST00000011315 1314 Vipr2 0.2617393713226135

ENSMUST00000005678 996 Fcer2a 0.2631633695649443

ENSMUST00000010211 813 Rassf1 0.270159679356625

ENSMUST00000005950 1422 Mmp12 0.1964531310022043

ENSMUST00000007130 2346 Ctnnb1 0.2179461943624708

ENSMUST00000006557 840 Elovl1 0.27289106187686685

ENSMUST00000007754 1479 Gtpbp3 0.3052215444388559

ENSMUST00000010974 645 Kdelr3 0.3282390624274822

ENSMUST00000010248 600 Nxf1 0.23804791947929804

ENSMUST00000009790 588 Pla2g12b 0.36899447268337837

ENSMUST00000011178 1998 Slc5a1 0.2934811117560942

ENSMUST00000009344 3375 Xpo6 0.25404704804270806

ENSMUST00000005487 501 Txn2 0.3047441825541122

ENSMUST00000008582 2190 Adam21 0.1744906776531203

ENSMUST00000006854 4083 Usp19 0.27296878237099376

ENSMUST00000012921 1278 Acpt 0.3145364442534079

ENSMUST00000004990 1083 Mapk14 0.28910906620140564

ENSMUST00000004829 1194 Cd244 0.2350346073531267

ENSMUST00000012679 2772 Tnpo3 0.17392763265966363

ENSMUST00000007042 1674 Ik 0.24664968180607552

ENSMUST00000007272 1455 Krt14 0.3973173791987897

ENSMUST00000011776 639 Pinlyp 0.3421167055495543

ENSMUST00000010795 1275 Acaa1b 0.302157465287805

ENSMUST00000004774 810 Aqp1 0.3393179640011965

ENSMUST00000009885 1515 Ubp1 0.21005761854564187

ENSMUST00000009774 930 Ppp2cb 0.2025703494776488

ENSMUST00000006301 1947 Lrwd1 0.33121603788899195

ENSMUST00000013294 1158 Gfod2 0.34165969853519285

ENSMUST00000009058 3831 Abcb1b 0.20705130363365395

ENSMUST00000009036 852 Vdac3 0.1891361834335523

ENSMUST00000006587 816 Tmem189 0.35103987977069406

ENSMUST00000007738 1203 Hapln4 0.29580029641009875

ENSMUST00000005643 1542 Celf1 0.22514943769691523

ENSMUST00000010682 375 Tsacc 0.21026657118186093

ENSMUST00000009705 1962 Eng 0.31113583524442

ENSMUST00000005019 417 Crabp2 0.3449361674490231

ENSMUST00000012279 645 Gsc2 0.2745274726180919

ENSMUST00000008088 720 Ttc9b 0.31648486210577303

ENSMUST00000007977 2409 Aldh16a1 0.267931302898747

ENSMUST00000012734 786 Dnajb6 0.22232231258303595

ENSMUST00000010804 2271 Plcd1 0.3401757287912654

ENSMUST00000012355 570 Tex22 0.3237449865461211

ENSMUST00000005751 2571 Mrvi1 0.27982074200862816

ENSMUST00000013773 6678 Cad 0.2865541342611831

ENSMUST00000007533 447 Vps25 0.26044603612806094

ENSMUST00000008004 1443 Ddx49 0.32863395698605863

ENSMUST00000004827 1635 Ly9 0.24605378946729758

ENSMUST00000011152 1701 Mcoln2 0.2636361336563263

ENSMUST00000005829 2301 Ampd3 0.3296194208637597

ENSMUST00000006667 7284 Gvin1 0.1744969656106637

ENSMUST00000005532 3738 Nid1 0.2936089556833266

ENSMUST00000009219 648 Cabp7 0.4086784888169597

ENSMUST00000010969 3075 Grid2ip 0.3297853755044526

ENSMUST00000009174 906 Pdcl 0.2525304961720263

ENSMUST00000007949 483 Twist2 0.4335833316647332

ENSMUST00000006476 774 Upk1a 0.3312456997532942

ENSMUST00000005493 1632 Slc1a3 0.28391843813085355

ENSMUST00000007800 339 Arpp19 0.19953219722467289

ENSMUST00000009727 705 Syngr1 0.4085221299567176

ENSMUST00000012540 918 Nanog 0.28626833885643777

ENSMUST00000008445 1158 Phax 0.22826005893231288

ENSMUST00000011492 1878 Acad9 0.24578505950672075

ENSMUST00000008594 384 Nutf2 0.2520389403506448

ENSMUST00000005279 1341 Klf5 0.2660978919473935

ENSMUST00000006893 3246 D130043K22Rik 0.25701674757951803

ENSMUST00000004968 2226 Plod3 0.31966841636219867

ENSMUST00000011055 726 Apip 0.24057893106310552

ENSMUST00000005220 1677 Slc1a2 0.2885725636174734

ENSMUST00000006716 1095 Wnt6 0.3175805867440194

ENSMUST00000010249 660 Taf6l 0.2286492579110184

ENSMUST00000006525 1680 Cbfa2t3 0.3440149294313482

ENSMUST00000011029 474 Dnajc19 0.17072602126528005

ENSMUST00000006293 912 Crkl 0.22038843152983084

ENSMUST00000005820 1077 Nr1i3 0.2685063280606206

ENSMUST00000013458 1134 Adh4 0.2131937928097991

ENSMUST00000006614 2934 Epha2 0.3455165982495348

ENSMUST00000005064 1524 Pias4 0.35273226534019125

ENSMUST00000007482 501 Mrpl49 0.2695352635659575

ENSMUST00000007171 2847 Chrd 0.274800741374155

ENSMUST00000006646 1593 Nsmf 0.3474576457678719

ENSMUST00000008991 7167 Sptbn2 0.3153685641964351

ENSMUST00000006760 1674 Cdt1 0.30975492533962284

ENSMUST00000007884 1758 Zfp54 0.18198970755267105

ENSMUST00000007747 1914 Dus3l 0.3347976128470333

ENSMUST00000004910 1056 Eif2b2 0.32093037111291045

ENSMUST00000010597 2634 Kansl3 0.2544183645563492

ENSMUST00000008451 633 1700109H08Rik 0.1887597047680802

ENSMUST00000010044 1059 Wnt3a 0.33840064798419583

ENSMUST00000004686 726 Pex11g 0.31592423489017846

ENSMUST00000008036 345 Rplp1 0.2864271849273003

ENSMUST00000012104 2175 Ccnt1 0.21501532334105233

ENSMUST00000005488 774 Casp14 0.25714214185547496

ENSMUST00000006911 912 Cdk4 0.2437626499247303

ENSMUST00000013227 333 2200002J24Rik 0.26361321533733373

ENSMUST00000006718 1254 Wnt10a 0.3677773347804569

ENSMUST00000008016 360 Id3 0.2687424611229243

ENSMUST00000007257 726 Clic1 0.3175825791247083

ENSMUST00000006444 7890 Tep1 0.24189074693110135

ENSMUST00000006626 2703 Actn3 0.3410330561717004

ENSMUST00000004480 351 Sst 0.3658828075454868

ENSMUST00000012426 1065 Wnt8a 0.3519108940509099

ENSMUST00000010451 726 Tmem86a 0.32846408303710517

ENSMUST00000009102 1107 Vps72 0.2652239782799927

ENSMUST00000006178 1293 Kptn 0.29908845461360634

ENSMUST00000005503 4077 Msh6 0.2021448885859361

ENSMUST00000009679 1398 Rnmt 0.181026422581568

ENSMUST00000010673 603 Gm266 0.4085570931118663

ENSMUST00000010736 897 Dazl 0.179418386800217

ENSMUST00000009522 1461 Slc16a12 0.21207471218008317

ENSMUST00000006380 822 Fam131c 0.3389414758604765

ENSMUST00000006470 8166 Kmt2b 0.2605223931275076

ENSMUST00000004507 1641 Ddx56 0.2499446799745962

ENSMUST00000006181 888 Napa 0.35758948934395257

ENSMUST00000004913 477 Pgf 0.2924214275735713

ENSMUST00000010520 246 Nedd8 0.23876634228628202

ENSMUST00000009798 1641 Oit3 0.32556679256497023

ENSMUST00000006659 798 Prl7a1 0.1557582387202895

ENSMUST00000004784 918 Cnn2 0.3826247373435843

ENSMUST00000007139 342 Eif1b 0.25458862796273896

ENSMUST00000007266 396 Lsm2 0.2680436252544055

ENSMUST00000008297 2871 Clstn3 0.3178012029486058

ENSMUST00000012281 1365 Bmp5 0.20689180008540725

ENSMUST00000007959 582 Rhoa 0.23397361500572741

ENSMUST00000006792 978 Ncr1 0.22191759698327385

ENSMUST00000013130 2391 Strn3 0.1514916279058562

ENSMUST00000006397 1524 Epor 0.2962794121712162

ENSMUST00000006391 1125 Serpinb9 0.2252038822464796

ENSMUST00000006136 855 Dnase1 0.2607747285163993

ENSMUST00000008573 1215 Herpud2 0.18894375816227532

ENSMUST00000006853 1512 P4htm 0.321364534265947

ENSMUST00000005051 1086 Ldha 0.27078821128223224

ENSMUST00000007245 2676 Vwa7 0.3002773194840409

ENSMUST00000013886 2349 Ppp1r12c 0.29835903079478276

ENSMUST00000010085 2076 Nxf2 0.2117020895940859

ENSMUST00000009396 771 Tspan32 0.3281601360836483

ENSMUST00000006435 1536 Atp6v1b2 0.24021376129717373

ENSMUST00000007131 3276 Acly 0.3068325660776086

ENSMUST00000006128 978 Rcn1 0.3364619968776861

ENSMUST00000009234 2832 Ap1b1 0.3233139821160706

ENSMUST00000006353 1737 Cdkal1 0.2129643912941568

ENSMUST00000010506 846 Rdm1 0.27139040541686926

ENSMUST00000010195 1389 Hyal1 0.2671399343009768

ENSMUST00000011450 1932 Sugp1 0.32611478628463114

ENSMUST00000008745 642 Rab25 0.34372863955863125

ENSMUST00000010421 6705 2610507B11Rik 0.23268566925684764

ENSMUST00000005889 2538 Vav1 0.32366902802772873

ENSMUST00000005841 2211 Ctcf 0.23526341769104447

ENSMUST00000006037 4389 Ncoa2 0.2636187478599766

ENSMUST00000005173 1509 Sgsh 0.33400631844160616

ENSMUST00000006235 1020 Ctsb 0.25912903599439074

ENSMUST00000006838 2328 Qars 0.2510302122589189

ENSMUST00000007207 2727 Clcn2 0.2823800111868768

ENSMUST00000008633 1083 Elavl2 0.2151378536218817

ENSMUST00000009693 333 Srp14 0.3657678899394097

ENSMUST00000013949 1809 Birc3 0.22345116671212759

ENSMUST00000010536 753 Gosr1 0.20961812347567244

ENSMUST00000005504 2793 Fbxo11 0.15756198030169077

ENSMUST00000006762 864 Snai3 0.3424689257660342

ENSMUST00000005352 3342 Corin 0.2682394692050447

ENSMUST00000011981 1080 Snapc2 0.27957636322414436

ENSMUST00000009390 3477 Trpm5 0.3258704921455193

ENSMUST00000007865 654 Ccdc124 0.37868621723539875

ENSMUST00000007275 1314 Krt13 0.3476724746140988

ENSMUST00000006053 2472 Smpd4 0.2872842535142715

ENSMUST00000004728 1212 Cd33 0.26573688655457467

ENSMUST00000004456 666 Gpx5 0.23187599662949207

ENSMUST00000013497 1104 4931406B18Rik 0.221145919731598

ENSMUST00000010049 999 Kdsr 0.23649879498304138

ENSMUST00000007156 786 Klk1b11 0.25155365918483136

ENSMUST00000005592 2067 Siglecg 0.28746189780499176

ENSMUST00000010251 1614 Slc22a8 0.2770766070099717

ENSMUST00000006112 2982 Ephb3 0.29653424085487384

ENSMUST00000005364 1068 G6pc2 0.22143286987188987

ENSMUST00000006005 1776 Pth1r 0.3113869454327136

ENSMUST00000005791 522 Cabp5 0.2757773434693509

ENSMUST00000010280 741 Pifo 0.2061393382899677

ENSMUST00000006704 2736 Itih1 0.29059632307270916

ENSMUST00000005671 4110 Igf1r 0.3174527246321546

ENSMUST00000006703 2829 Itih4 0.26508659325803025

ENSMUST00000005188 1866 Sh2b2 0.3132134684183725

ENSMUST00000005003 1881 Lbr 0.23146147187070817

ENSMUST00000006478 675 Tmem147 0.28769195118458785

ENSMUST00000004868 501 Mtfp1 0.35665592700402965

ENSMUST00000012849 345 Retn 0.26664663730848365

ENSMUST00000009831 540 Fxyd5 0.2634336760578075

ENSMUST00000006764 543 Aprt 0.3241592822695971

ENSMUST00000006669 1305 Pdk1 0.21682510178704462

ENSMUST00000009329 294 Ccl8 0.3069699132912981

ENSMUST00000013759 3234 Fnbp4 0.18863056532750969

ENSMUST00000005815 2940 Kit 0.2447323892425447

ENSMUST00000006027 570 Reep5 0.36717188649165866

ENSMUST00000009789 1605 P4ha1 0.19949894996577705

ENSMUST00000005077 630 Hspb1 0.30954327171757384

ENSMUST00000006523 234 Crip1 0.319916806689347

ENSMUST00000013299 1041 Enkd1 0.2814466033830439

ENSMUST00000007255 858 Ddah2 0.2533732098969393

ENSMUST00000012314 2922 Zfp729a 0.18041962869946715

ENSMUST00000006625 2010 Rbm14 0.24516247572191813

ENSMUST00000013766 672 Atraid 0.21834528546921922

ENSMUST00000009777 312 G0s2 0.37097673812619936

ENSMUST00000008517 1500 Prpf31 0.3240880266319267

ENSMUST00000006749 2790 Slc4a1 0.30833962239300916

ENSMUST00000005907 495 Cd247 0.3287185509515867

ENSMUST00000006660 762 Prl7a2 0.15117718584617226

ENSMUST00000013559 1734 Igf2bp1 0.2879091380622572

ENSMUST00000007340 3108 Atp12a 0.25544713416760934

ENSMUST00000008542 1230 Elk3 0.3112717127637116

ENSMUST00000006286 1407 Inpp5k 0.277774895290485

ENSMUST00000006137 2121 Trap1 0.28490281403393597

ENSMUST00000005041 1428 U2af2 0.2992177749168503

ENSMUST00000004681 3984 Pnpla6 0.2606814347907883

ENSMUST00000010899 2496 Cars 0.27736131446130613

ENSMUST00000013220 2907 Amotl1 0.309725308463799

ENSMUST00000006559 1281 Tpbg 0.26629061073131177

ENSMUST00000005749 3522 Ctr9 0.22888208192445575

ENSMUST00000013771 1101 Trim54 0.3833907847445402

ENSMUST00000004565 621 Ralb 0.3422891060991172

ENSMUST00000004473 729 Spic 0.22140254469170695

ENSMUST00000011493 1113 Dmrtc2 0.2703943731368443

ENSMUST00000005705 2505 Trim28 0.26200392494773517

ENSMUST00000008999 3366 Hdac5 0.3416331702920935

ENSMUST00000007993 2253 Rbm28 0.23991037056591752

ENSMUST00000009667 2733 Ush1c 0.3300508498943156

ENSMUST00000004959 654 Grap 0.35374244668766713

ENSMUST00000005336 1707 Senp3 0.2521543148999336

ENSMUST00000012028 630 Gltp 0.42624657785994774

ENSMUST00000007449 951 Akr1b7 0.3078733560700966

ENSMUST00000009728 576 Syngr1 0.401231184017873

ENSMUST00000009713 3276 Mkl2 0.2606117932272492

ENSMUST00000006750 1341 Rundc3a 0.37220929151346144

ENSMUST00000011058 1506 Pdhx 0.1947546460862736

ENSMUST00000010250 1638 Slc22a6 0.33168657891779424

ENSMUST00000006544 1251 Gcat 0.32030823383742235

ENSMUST00000012873 3138 Zfp729b 0.16827243882692672

ENSMUST00000012262 1002 Dhdds 0.3035970750354753

ENSMUST00000009772 8358 Tex15 0.1355424761046519

ENSMUST00000007046 1485 Tmco6 0.248383567637222

ENSMUST00000012664 945 Phox2b 0.27837999972738886

ENSMUST00000004786 633 Polr2e 0.41646069676205355

ENSMUST00000005365 681 Spc25 0.19225689774191787

ENSMUST00000004936 360 Ccl24 0.28244027334006044

ENSMUST00000006035 1152 Ergic3 0.35348690602078514

ENSMUST00000006742 4389 Atp7b 0.2765638386984078

ENSMUST00000010201 1143 Nprl2 0.3422589024106913

ENSMUST00000010007 849 Sdhb 0.3109404583947367

ENSMUST00000009321 2322 Dgcr8 0.22614385400014558

ENSMUST00000008579 1005 Rdh13 0.27166076905336545

ENSMUST00000004729 768 Etfb 0.34707180768564483

ENSMUST00000009538 1761 Syn2 0.29566861739974376

ENSMUST00000007216 1308 Ap2m1 0.3397627639371058

ENSMUST00000009039 348 Rpl30 0.22506725418604837

ENSMUST00000010348 525 Fdx1l 0.3280863781549257

ENSMUST00000005234 1821 Wdr1 0.28495937732658727

ENSMUST00000005256 1185 Ndrg1 0.33313507332158176

ENSMUST00000005548 870 Hmox1 0.34924384553729604

ENSMUST00000007584 2895 Pcdhac1 0.2157505224102995

ENSMUST00000006508 1707 Ggt1 0.30031584705228753

ENSMUST00000006915 807 Mettl1 0.2836407992753781

ENSMUST00000007280 1410 Krt16 0.34835003781170554

ENSMUST00000007248 1926 Hspa1l 0.3173385517754083

ENSMUST00000006991 2733 Hcn1 0.2598241509847363

ENSMUST00000009340 1278 Mnda 0.1897116407314505

ENSMUST00000009018 591 Clec3a 0.2650541284910558

ENSMUST00000010807 1344 Fads1 0.3282889996420832

ENSMUST00000013787 768 Lypd8 0.258438849043558

ENSMUST00000011302 2031 Brf1 0.2733011565547188

ENSMUST00000011607 1248 Cpb1 0.2328837924128092

ENSMUST00000007814 2247 Khsrp 0.2929243334748172

ENSMUST00000004554 615 Rps5 0.3364389900131389

ENSMUST00000007251 1677 Abhd16a 0.3496480321124023

ENSMUST00000006900 423 Acot13 0.19018548173367375

ENSMUST00000009236 687 Derl3 0.35256486222232125

ENSMUST00000013845 630 Timm23 0.17781358541675255

ENSMUST00000006105 1431 Shpk 0.28162070122874533

ENSMUST00000008090 843 Phox2a 0.30704744812308427

ENSMUST00000005053 423 Tmem242 0.1784133926909447

ENSMUST00000005218 2343 Cd44 0.22619224141402533

ENSMUST00000005452 2400 Fgfr4 0.29835641064827545

ENSMUST00000007733 1245 Tinf2 0.2230405191789506

ENSMUST00000006956 369 Saa3 0.2731108134707839

ENSMUST00000004494 3297 Sin3b 0.34896796002827063

ENSMUST00000006496 585 Rps9 0.2986913058313047

ENSMUST00000006851 2334 Qrich1 0.23166425591140402

ENSMUST00000008830 1638 Zfp955a 0.19062946878778983

ENSMUST00000004684 3066 Arhgef18 0.3121495703595543

ENSMUST00000006403 1212 Ccdc159 0.32741983974494193

ENSMUST00000005108 5073 Kdm5a 0.1777717927415093

ENSMUST00000013302 924 4933405L10Rik 0.2610998481188109

ENSMUST00000008748 1791 Ubqln4 0.32660075566669106

ENSMUST00000008528 711 Sertad1 0.30638848341659264

ENSMUST00000004749 300 Pcp2 0.35006960850551777

ENSMUST00000004646 1425 Coro1c 0.3034789626987464

ENSMUST00000010208 1518 Slc38a3 0.32565894799772205

ENSMUST00000013970 1266 Pip4k2c 0.3010934626733377

ENSMUST00000010267 1704 Slc47a1 0.24481141468961473

ENSMUST00000013737 1392 Ndufs2 0.27337083828916175

ENSMUST00000011526 1002 Dhdh 0.24674704482499

ENSMUST00000005017 714 Hdgf 0.3383857273306967

ENSMUST00000013235 501 Tmem190 0.24829675074785168

ENSMUST00000008893 1455 Coro1b 0.30713664263603213

ENSMUST00000005976 720 Tnfsf14 0.27719718761876705

ENSMUST00000009120 1365 Gabpa 0.168208854139861

ENSMUST00000004965 642 Chmp2b 0.2144937945756438

ENSMUST00000004986 1101 Mapk13 0.3674108861862636

ENSMUST00000010904 435 Phlda2 0.4172478215863252

ENSMUST00000006687 621 Orm3 0.24126558126271203

ENSMUST00000013882 1335 Crbn 0.18550767410276064

ENSMUST00000005952 1293 Slc30a4 0.17994312716177496

ENSMUST00000006825 3771 Nphs1 0.29629567721531874

ENSMUST00000013755 1317 Snx31 0.29564645950761165

ENSMUST00000005606 1056 Prkaca 0.36089903191130324

ENSMUST00000005714 552 Ube2m 0.3467754883401064

ENSMUST00000008734 1314 Htr3b 0.2592619496955995

ENSMUST00000006632 855 Zdhhc24 0.2977690090045411

ENSMUST00000006786 1344 Slc17a2 0.21684365973339598

ENSMUST00000006774 1644 Gtf2h1 0.19139034771408217

ENSMUST00000007790 1155 Rad51c 0.141127734694599

ENSMUST00000013931 3792 Ehmt2 0.330201035575807

ENSMUST00000008812 459 Rps18 0.3218821476964833

ENSMUST00000004949 1593 Traf6 0.2372264420993594

ENSMUST00000008477 678 Snrpb2 0.18854900154922116

ENSMUST00000006828 1965 Aplp1 0.3100804924365374

ENSMUST00000007735 879 Tssk4 0.298308837378267

ENSMUST00000004756 1245 Wwox 0.3122826663114341

ENSMUST00000006061 3735 Pex1 0.1911603881556011

ENSMUST00000006754 2259 Ubtf 0.33876910184804315

ENSMUST00000007250 2502 Msh5 0.2808583048939137

ENSMUST00000005954 519 Bloc1s6 0.2474992627777055

ENSMUST00000004683 1743 Mcoln1 0.33479433796605435

ENSMUST00000006969 1269 Krt23 0.2851194453779721

ENSMUST00000011398 897 Thg1l 0.2839002199418599

ENSMUST00000005830 678 Bcas2 0.2210105258458017

ENSMUST00000005620 1023 Dnajb1 0.3037534470323025

ENSMUST00000012798 1710 Siglecf 0.27468001048515145

ENSMUST00000007444 1296 Strada 0.2729190215491583

ENSMUST00000004732 522 Lim2 0.34808727612832874

ENSMUST00000007259 408 Ly6g6d 0.2957898176096482

ENSMUST00000006618 2409 Arhgef19 0.3347591507349038

ENSMUST00000006123 1173 Tuft1 0.28944849468734707

ENSMUST00000004505 4002 Npc1l1 0.2961211897428005

ENSMUST00000006377 2385 Zbtb17 0.3371168744551487

ENSMUST00000013667 1902 Bcas1 0.2585121342725314

ENSMUST00000007012 669 Sod2 0.2801406262402394

ENSMUST00000011298 912 1700026D08Rik 0.25175015935708445

ENSMUST00000006611 909 Srm 0.3914572506832244

ENSMUST00000008273 198 Prok2 0.22929698759793243

ENSMUST00000006701 867 Tmem110 0.27024140983417216

ENSMUST00000005810 1053 Mthfd2 0.22760414595565243

ENSMUST00000004943 648 Tmed11 0.11435965628338587

ENSMUST00000009732 1581 St6galnac1 0.27425060057929274

ENSMUST00000005185 297 Cstb 0.26192125812085276

ENSMUST00000008021 504 Tcap 0.37909232566167805

ENSMUST00000011391 954 Prss45 0.27495824578983874

ENSMUST00000012161 2502 Scarf2 0.3080159652937855

ENSMUST00000005891 756 Klk9 0.3085676668850543

ENSMUST00000008987 636 Cldn13 0.24503138844538783

ENSMUST00000004715 1557 Mospd2 0.1849535141309714

ENSMUST00000005923 795 Psmb4 0.26178744396133374

ENSMUST00000006662 726 Prl8a9 0.17331668096040045

ENSMUST00000008032 1278 Crlf1 0.32118192985518756

ENSMUST00000005607 609 Asf1b 0.3321380673792662

ENSMUST00000006046 771 Trh 0.2615442062513079

ENSMUST00000004470 8370 Utp20 0.19636833124093622

ENSMUST00000009875 1956 Kcnd1 0.289180992972863

ENSMUST00000005825 3603 Pan2 0.27967239094642554

ENSMUST00000012627 366 Rpa3 0.18526004512052194

ENSMUST00000006664 723 Prl8a1 0.17945340655825703

ENSMUST00000011895 7686 Sptbn4 0.3290516120091443

ENSMUST00000005616 2841 Pkn1 0.31332591981760155

ENSMUST00000010298 2157 Spire2 0.35666780641102264

ENSMUST00000006963 1389 Krt28 0.2967327069857647

ENSMUST00000008826 645 Rpl10 0.2880408574615118

ENSMUST00000009259 1029 Spatc1l 0.30887957908969566

ENSMUST00000013693 552 Commd8 0.19311419299926483

ENSMUST00000007797 1425 Gabrb2 0.22913322636308295

ENSMUST00000008995 3231 Hdac4 0.30826566989139603

ENSMUST00000009435 525 Pttg1ip 0.3100563432787879

ENSMUST00000006451 1323 Ttc5 0.2904430322223497

ENSMUST00000006378 2064 Clcnkb 0.3289378761633918

ENSMUST00000005014 1026 Hapln2 0.3399412421878618

ENSMUST00000009699 1119 Cdk9 0.34801397267529055

ENSMUST00000009550 1290 Elk1 0.259478880601311

ENSMUST00000004574 1077 Dnajb11 0.244915075361081

ENSMUST00000005431 2217 Letm1 0.3201547191911431

ENSMUST00000004770 1602 Tyr 0.203396693807009

ENSMUST00000005072 1857 Dtx2 0.33247456812561793

ENSMUST00000006103 1104 Ctns 0.25817297938370015

ENSMUST00000009138 1398 Stk38 0.23094990446923416

ENSMUST00000007253 1230 Neu1 0.34369246578378143

ENSMUST00000007980 918 Hnrnpa0 0.42618476780697145

ENSMUST00000010198 333 Tusc2 0.3362144962355664

ENSMUST00000007161 768 Klk4 0.2657945268553707

ENSMUST00000012847 717 Cd209a 0.26651516559267996

ENSMUST00000005507 2595 Mlxipl 0.28984092938804157

ENSMUST00000005975 1710 Gpr108 0.334799030891613

ENSMUST00000010985 495 1700006E09Rik 0.1879743311079216

ENSMUST00000006949 1467 Tph2 0.2418561655727293

ENSMUST00000009358 666 Tmem8c 0.36339669957404713

ENSMUST00000005769 1038 Tmod4 0.26618426337746115

ENSMUST00000007602 837 M6pr 0.22623838046778072

ENSMUST00000005798 1221 Snx6 0.16061139556181125

ENSMUST00000009157 270 Dynll1 0.33782881251635644

ENSMUST00000009411 1479 Zfp212 0.3126425650860916

ENSMUST00000006638 1806 Slc34a3 0.2848873463715062

ENSMUST00000005647 792 Ndufs3 0.2409196718792816

ENSMUST00000013851 555 Tnfaip8l2 0.3348121808700583

ENSMUST00000012259 2370 Med15 0.30686972584150973

ENSMUST00000012152 1650 Dgcr2 0.30206711346655357

ENSMUST00000007007 1191 Wtap 0.18377466466675035

ENSMUST00000005334 1212 Shbg 0.25398478809708597

ENSMUST00000009740 1416 Taf7l 0.20008693264726318

ENSMUST00000005015 1476 Prcc 0.26201015352568674

ENSMUST00000007317 1212 Krt19 0.3575984823795146

ENSMUST00000007559 801 Gatad1 0.2265382217702427

ENSMUST00000011400 2763 Adam19 0.2959624195528899

ENSMUST00000010038 1242 Snap47 0.2933530422494103

ENSMUST00000006692 1206 Mvd 0.32797899653128365

ENSMUST00000004622 2001 Gab2 0.29918776218347476

ENSMUST00000006303 753 Upk3bl 0.314516835885497

ENSMUST00000005601 1872 Il27ra 0.26836355854816624

ENSMUST00000006818 630 Tcf23 0.28080358891107726

ENSMUST00000007212 2727 Psmd2 0.2411049739797665

ENSMUST00000006341 1530 Prss16 0.23840839296255853

ENSMUST00000006029 1278 Arhgap8 0.32512020492555904

ENSMUST00000013910 405 Ly6g6e 0.3006584796001697

ENSMUST00000005233 4947 Eif2ak4 0.250387529966382

ENSMUST00000006697 2670 Itih3 0.30109984519605065

ENSMUST00000006745 216 Defb2 0.23166035768801255

ENSMUST00000011285 594 Fgf11 0.3399843198760321

ENSMUST00000008284 807 Emc10 0.31653553716071275

ENSMUST00000010020 519 Cox4i2 0.35228772942260833

ENSMUST00000005067 948 Sgta 0.3625090145706031

ENSMUST00000005262 948 4930550C14Rik 0.20719948187846746

ENSMUST00000005669 1476 Cyp2b13 0.23987006856796528

ENSMUST00000005849 396 Agrp 0.27728470086425916

ENSMUST00000006467 903 Arpc2 0.23198839476699518

ENSMUST00000012186 978 4930579F01Rik 0.23085040476476856

ENSMUST00000006094 1503 Cyp2d26 0.30627711491350734

ENSMUST00000007296 6852 Pole 0.2721772446230574

ENSMUST00000012348 657 Gstm2 0.33438229895099486

ENSMUST00000006565 1500 Cdc20 0.25783925355531007

ENSMUST00000010434 699 AI597479 0.2181791665336752

ENSMUST00000004587 987 Clec11a 0.28975072425559356

ENSMUST00000006311 1284 Tead4 0.32772123351150784

ENSMUST00000006221 2934 Vps54 0.14290482049111955

ENSMUST00000005839 381 Sh2d1a 0.23329302930092247

ENSMUST00000005394 924 Ufd1l 0.24158021725983073

ENSMUST00000009617 1887 Kcnc4 0.3127046253687189

ENSMUST00000005711 669 Chmp2a 0.2701036525292174

ENSMUST00000008051 1176 Cabs1 0.16650062665269366

ENSMUST00000004657 1428 Psg19 0.20555427060626472

ENSMUST00000005413 2640 Zfp112 0.24875642799496814

ENSMUST00000005826 1395 Cs 0.2764357636116719

ENSMUST00000009392 792 Ascl2 0.26636984227131266

ENSMUST00000004560 588 Bid 0.31944396547225373

ENSMUST00000007249 2124 Slc44a4 0.3049173654196444

ENSMUST00000013338 1479 Arih2 0.2552088721079096

ENSMUST00000007236 690 Syngr3 0.29739196153721564

ENSMUST00000005073 1275 Zp3 0.27385490524369

ENSMUST00000005583 699 Pafah1b3 0.2613104789664537

ENSMUST00000009695 489 6330409D20Rik 0.1649370983297483

ENSMUST00000006973 2490 Kat2a 0.32606412485695846

ENSMUST00000011262 1179 Panx3 0.26208663564985396

ENSMUST00000010753 1347 Psen2 0.3694906892890245

ENSMUST00000007981 597 Prrg2 0.2656202111746976

ENSMUST00000010192 1326 Ifrd2 0.2884579291281944

ENSMUST00000006761 1755 Runx1t1 0.2512827016106319

ENSMUST00000005692 3105 Atp4a 0.32556789816159687

ENSMUST00000006362 684 Rhox6 0.251255466402737

ENSMUST00000006020 1995 Cnga2 0.2596358956498203

ENSMUST00000010940 1428 Asz1 0.15934949171806623

ENSMUST00000005817 927 Tomm40l 0.26433229073515496

ENSMUST00000006104 1368 P2rx5 0.31050284722796995

ENSMUST00000008626 720 Rnf151 0.3136220023902606

ENSMUST00000009003 621 Rala 0.21522806873673694

ENSMUST00000010241 1857 Nxf1 0.2010277209728938

ENSMUST00000006856 4398 Pola1 0.1753940725960016

ENSMUST00000006912 1218 Pip4k2a 0.2444065155044019

ENSMUST00000006424 651 Mob1b 0.19514984411415678

ENSMUST00000007921 423 0610009B22Rik 0.2046833670163948

ENSMUST00000010205 1053 Gnat1 0.34155656937196865

ENSMUST00000011896 765 Pgam1 0.352440913529863

ENSMUST00000005292 597 Prdx2 0.2991378692261017

ENSMUST00000011877 7092 Sptbn1 0.27409225281744576

ENSMUST00000006914 1602 B4galnt1 0.26176307888303174

ENSMUST00000006952 393 Saa4 0.24693608271843173

ENSMUST00000008907 1926 Man1a2 0.19618662598074652

ENSMUST00000009814 2844 Tex11 0.14729698195018134

ENSMUST00000005016 1428 Rrnad1 0.2891576029562777

ENSMUST00000004750 1356 Sgce 0.19078536547380598

ENSMUST00000010189 1053 Tmem115 0.2714836970901996

ENSMUST00000004985 3615 Brpf3 0.31948937075057254

ENSMUST00000005862 1017 Tfap4 0.36286956529552195

ENSMUST00000011623 2361 Dennd1c 0.26277415710374397

ENSMUST00000005069 588 Nmrk2 0.31241869935505134

ENSMUST00000005964 1125 Adh5 0.2293527722244949

ENSMUST00000011445 714 Cd209d 0.2576591442868442

ENSMUST00000012331 1008 Mogat1 0.22112777395901606

ENSMUST00000006785 1398 Slc17a1 0.19860036914913803

ENSMUST00000008280 840 Fhl2 0.37023225549516126

ENSMUST00000005066 1182 Map2k1 0.29631459344051025

ENSMUST00000006101 3504 Itgae 0.27067758938340586

ENSMUST00000006367 1443 Htra1 0.2703533053617727

ENSMUST00000006071 1068 Otx1 0.28571574293525814

ENSMUST00000006431 1542 Atp6v1b1 0.3272837807136639

ENSMUST00000004955 1110 Prpsap2 0.2426946747807489

ENSMUST00000004430 2601 Clcn3 0.178021448716526

ENSMUST00000010279 630 Tmigd3 0.2477925392836856

ENSMUST00000009256 1305 Bcl2l13 0.2217485468194372

ENSMUST00000005406 2088 App 0.32279577027095535

ENSMUST00000005120 597 Ccdc130 0.3503991442938814

ENSMUST00000006721 594 Cryba2 0.3287560713337819

ENSMUST00000007799 537 Cav1 0.3258478162546579

ENSMUST00000013633 1590 Fgfrl1 0.29136759365327525

ENSMUST00000013304 1056 Atp6v0d1 0.30656183397812864

ENSMUST00000010210 3447 Cacna2d2 0.3217521204120991

ENSMUST00000004614 2499 Zfp110 0.21109691671313519

ENSMUST00000010127 1788 Tktl1 0.20685013080793907

ENSMUST00000010278 1029 Wdr77 0.22600504238826588

ENSMUST00000004453 666 Gpx6 0.2520700962346791

ENSMUST00000005164 1197 Prkacb 0.2822618007936425

ENSMUST00000004508 684 Tmed4 0.307600967713554

ENSMUST00000007005 1194 Acat2 0.2237896472688515

ENSMUST00000008966 300 Acyp1 0.25730999456492853

ENSMUST00000009689 2007 Kcnq1 0.304636843975411

ENSMUST00000013797 708 1810065E05Rik 0.19140815406441847

ENSMUST00000008878 1233 Gprc5b 0.34877317546471387

ENSMUST00000005933 786 Klk1b16 0.249490101972689

ENSMUST00000005630 2877 Msh4 0.1822272573595532

ENSMUST00000013262 1758 Zkscan17 0.33396280716949805

ENSMUST00000004994 1455 Pax3 0.293144593556538

ENSMUST00000007757 1512 Tgfbr1 0.16329591710940158

ENSMUST00000005255 1104 Wisp1 0.326288696650665

ENSMUST00000005611 444 Myl10 0.3325672766271381

ENSMUST00000008179 549 Mid1ip1 0.37728000751123975

ENSMUST00000006679 765 Prtn3 0.37018298396190236

ENSMUST00000006578 1527 Pvrl4 0.29768209228163856

ENSMUST00000004497 2271 Large 0.2929697194803007

ENSMUST00000007708 1770 Ppp2r1a 0.28345571207496056

ENSMUST00000010188 1323 Zmynd10 0.30553052283204357

ENSMUST00000005685 1485 Cyp2a5 0.3277761205149771

ENSMUST00000005600 2892 Rfx1 0.3591763942030729

ENSMUST00000005953 1353 Sqrdl 0.2553851746790996

ENSMUST00000008537 447 Carhsp1 0.3094853514617005

ENSMUST00000009241 1467 Tbx1 0.30421506508320645

ENSMUST00000008733 1098 Dnajb6 0.20633589599041005

ENSMUST00000009356 1248 Serpinb2 0.2159559619921677

ENSMUST00000010191 1422 Hyal2 0.31317558916084975

ENSMUST00000006151 1614 Tyrp1 0.21093951418179704

ENSMUST00000008462 1311 Relt 0.2794234500791922

ENSMUST00000009707 966 Tor2a 0.3555421332938003

ENSMUST00000010579 549 Spaca7 0.17458902451774586

ENSMUST00000004655 1428 Psg17 0.18650772644174649

ENSMUST00000010550 366 Mrpl52 0.2057370498912099

ENSMUST00000005490 1686 Slc1a6 0.2883313815262948

ENSMUST00000018918 981 Cd68 0.22931565525247652

ENSMUST00000016143 1506 Wasf3 0.26809095019049456

ENSMUST00000021853 954 Eci3 0.21108963647829426

ENSMUST00000020546 891 Stc2 0.3141155463300888

ENSMUST00000020721 2766 Smtn 0.29179296002780847

ENSMUST00000015749 1515 Srf 0.3456988363781429

ENSMUST00000020308 690 Ddit4 0.32899173400205334

ENSMUST00000018005 2115 Mybl2 0.33011115785415357

ENSMUST00000018549 963 Mrm1 0.29476443935368285

ENSMUST00000021843 696 Nqo2 0.2139548888073209

ENSMUST00000021670 6420 Ylpm1 0.19347145687265954

ENSMUST00000019169 813 Use1 0.3139612065368376

ENSMUST00000018805 2943 Cog1 0.3086514484970507

ENSMUST00000018066 639 Prl3c1 0.17507911964375408

ENSMUST00000018544 1437 Sept4 0.3161808365450491

ENSMUST00000014476 801 Klra8 0.18130896403537478

ENSMUST00000016106 438 1700016C15Rik 0.15632787651170768

ENSMUST00000021595 1323 Psmc1 0.22957390401392386

ENSMUST00000020964 327 Fkbp1b 0.3052797548875005

ENSMUST00000020930 1134 Gna13 0.27838879712248676

ENSMUST00000015645 7293 Hivep2 0.21820944792292

ENSMUST00000019386 2361 Ripk4 0.3114383128080902

ENSMUST00000020158 1911 Myb 0.24444181057098105

ENSMUST00000018431 684 Spag7 0.29363192197420446

ENSMUST00000021817 1470 Ippk 0.23169574884225558

ENSMUST00000017572 1269 Psmd11 0.2254240286010916

ENSMUST00000016668 531 Il19 0.2789132857984357

ENSMUST00000015017 774 Surf2 0.3137194426726019

ENSMUST00000014981 1404 4931428F04Rik 0.3218957107798183

ENSMUST00000017799 870 Cd40 0.2667637102212867

ENSMUST00000014080 501 Myl2 0.32438813163984326

ENSMUST00000021790 345 Tmem14c 0.20043274917755366

ENSMUST00000020756 2352 Pnpt1 0.13407570244460856

ENSMUST00000021659 1770 Fam161b 0.29211065956214055

ENSMUST00000020238 2409 Hsp90b1 0.1975791527632331

ENSMUST00000015620 921 Prrt1 0.273734869180992

ENSMUST00000018610 3435 Nos2 0.3263251944265286

ENSMUST00000018389 723 Prl8a8 0.1607457426839543

ENSMUST00000021181 471 0610010K14Rik 0.2785569899327094

ENSMUST00000021420 456 Ngb 0.3679224953325728

ENSMUST00000020118 1146 Dusp6 0.3281730844631116

ENSMUST00000021065 672 Cacng1 0.34119159960006196

ENSMUST00000015107 2157 Tlk2 0.16709536077250423

ENSMUST00000018710 1530 Slc2a4 0.2573387055873823

ENSMUST00000016094 1215 Ncf1 0.3392134792332361

ENSMUST00000021062 1848 Ddx5 0.17916741404704548

ENSMUST00000020681 1758 Slu7 0.2509209657464075

ENSMUST00000021933 1005 Ctsl 0.2657874027087598

ENSMUST00000021005 2745 Tpo 0.275286705739677

ENSMUST00000020350 2724 Lgr5 0.2000823914065354

ENSMUST00000019911 1467 Hdac2 0.17479309638132237

ENSMUST00000018143 2283 Ddx27 0.34902626411716575

ENSMUST00000020223 1554 Tcp11l2 0.22539265557419472

ENSMUST00000020775 270 Dynll2 0.3204587894135095

ENSMUST00000020692 477 Btg2 0.3785732782941299

ENSMUST00000017945 897 Mlx 0.28218067461089474

ENSMUST00000017590 846 C1qtnf1 0.38742801604666677

ENSMUST00000020573 855 Prss57 0.2701193789726191

ENSMUST00000019422 1233 Dpep1 0.3169704673036736

ENSMUST00000020153 2049 Hbs1l 0.1909141167760537

ENSMUST00000021681 1128 Vash1 0.35526117984534944

ENSMUST00000020754 1032 Cfap36 0.17355529784076845

ENSMUST00000021691 972 Degs2 0.3206176293346945

ENSMUST00000019354 681 Atp6v1e1 0.2328112178026421

ENSMUST00000020315 3693 Cand1 0.18175830592238268

ENSMUST00000016511 1356 Ptk6 0.335957256027896

ENSMUST00000020650 396 Il13 0.28609005889110434

ENSMUST00000020366 1323 Gabrp 0.25172775218873567

ENSMUST00000020947 1005 Rdh14 0.22074172493793298

ENSMUST00000020513 2220 Papolg 0.1397521372690466

ENSMUST00000020984 3438 Adcy3 0.3579066747932159

ENSMUST00000021514 1149 Ppm1a 0.19733070040304657

ENSMUST00000020434 999 Glipr1l2 0.1792174514228836

ENSMUST00000015346 1668 Cnksr3 0.2908309372676661

ENSMUST00000015841 3927 Setdb1 0.2287254766101096

ENSMUST00000015481 885 Endog 0.32133733230045775

ENSMUST00000020792 828 Btnl10 0.2785499731794252

ENSMUST00000021550 1065 Arg2 0.19169003204123736

ENSMUST00000021438 1524 Nova1 0.1608079110227266

ENSMUST00000020920 2028 Rgs9 0.3015421995484237

ENSMUST00000021793 879 Elovl2 0.2788055038636033

ENSMUST00000020878 399 Efcab10 0.2119038974225688

ENSMUST00000020253 1197 Chpt1 0.15348673228176835

ENSMUST00000019896 858 Iyd 0.2771783729696398

ENSMUST00000021077 1068 Slc9a3r1 0.34705831163175543

ENSMUST00000020958 2628 Klhl29 0.29410175815695433

ENSMUST00000020501 333 Sumo3 0.3423099868086469

ENSMUST00000020049 723 Ccdc59 0.22257037673311708

ENSMUST00000021938 681 Aaed1 0.20518064881882103

ENSMUST00000021447 1404 Ppp2r5e 0.17336709963688438

ENSMUST00000018744 1437 Shmt1 0.2694075158471401

ENSMUST00000017151 1548 Rbpjl 0.337621095130513

ENSMUST00000021164 696 Fam64a 0.2854774832653146

ENSMUST00000019135 2157 Gga3 0.27754059063081565

ENSMUST00000021806 723 Tpmt 0.16642192525665256

ENSMUST00000021611 3111 Pitrm1 0.2483675501955015

ENSMUST00000021197 1368 Blmh 0.24111480488615453

ENSMUST00000020377 1959 Tcf3 0.31804699982838647

ENSMUST00000020586 1662 Slc22a4 0.3031970750011378

ENSMUST00000018851 13935 Dync1h1 0.2971925096364144

ENSMUST00000018186 906 Cyb5r3 0.34171371738356837

ENSMUST00000021787 1296 Tfap2a 0.2527145051316318

ENSMUST00000021287 1863 Cfap52 0.2623079641943039

ENSMUST00000019362 2211 Dvl2 0.29973705354643254

ENSMUST00000021297 588 Lsm12 0.197719872241782

ENSMUST00000018184 843 Rrp7a 0.33307392187619284

ENSMUST00000015267 825 Prss28 0.3133078414340503

ENSMUST00000020298 315 Pcbd1 0.3301095847511121

ENSMUST00000021757 1725 Aoah 0.21842326263507778

ENSMUST00000020381 1527 Frs2 0.18780032231768887

ENSMUST00000020411 2697 Osbpl5 0.33392452058674743

ENSMUST00000019118 2889 Sart3 0.3309369998934258

ENSMUST00000015664 990 Ctsk 0.2787982681540447

ENSMUST00000015903 435 Cnih1 0.27750034375097254

ENSMUST00000021243 969 Slc35b1 0.2840266651865608

ENSMUST00000020768 762 Pgam2 0.32682514362732085

ENSMUST00000019907 1266 Fbxo5 0.2131855165565514

ENSMUST00000015611 882 Egfl8 0.30283319070509956

ENSMUST00000020102 1806 Slc17a8 0.24805000028927854

ENSMUST00000021471 837 Tmx1 0.17763321502291804

ENSMUST00000020440 288 Timm13 0.4099834146687012

ENSMUST00000018311 1341 Stard3 0.3080541892235861

ENSMUST00000021864 861 Ssr1 0.18647000190128554

ENSMUST00000020851 828 Cox11 0.2403463343884311

ENSMUST00000018449 7008 Prpf8 0.28636386573420547

ENSMUST00000020504 381 Hint1 0.250323232452707

ENSMUST00000020484 1500 Txnrd1 0.22664767329279123

ENSMUST00000020145 1296 Sgk1 0.26693532356631244

ENSMUST00000021959 1035 Txndc15 0.2599136643072613

ENSMUST00000020980 1173 Rrm2 0.24829735923759755

ENSMUST00000019649 918 Ubb 0.48429991501641895

ENSMUST00000015049 669 Dnajb9 0.182750883428317

ENSMUST00000020382 684 Yeats4 0.21468093841670444

ENSMUST00000014673 909 Tmbim7 0.19171111463980348

ENSMUST00000014447 1566 Glis2 0.30475109457161637

ENSMUST00000019726 1314 Plin3 0.339756865807894

ENSMUST00000020948 4863 Abca8b 0.19138118213387104

ENSMUST00000020941 792 1700012B07Rik 0.2482992391676141

ENSMUST00000020317 747 Pno1 0.18211015512308587

ENSMUST00000019128 921 Hnrnpd 0.1894308781006697

ENSMUST00000019517 1272 Cops3 0.18666033152665962

ENSMUST00000021715 1050 Xrcc3 0.32604229482921226

ENSMUST00000021040 1596 Cct6b 0.17348497307830288

ENSMUST00000019701 1359 Dusp9 0.29355791074438525

ENSMUST00000014830 1281 Ceacam16 0.3094951745801385

ENSMUST00000021416 1026 Mbip 0.1694334543902384

ENSMUST00000020485 1056 Glt8d2 0.24483634998123532

ENSMUST00000021141 1200 P2rx1 0.3062160511916138

ENSMUST00000015889 1227 Plekho1 0.32766381781283765

ENSMUST00000019257 1656 Aire 0.30733037539257324

ENSMUST00000020608 930 Ppp2ca 0.21082241420239714

ENSMUST00000019876 1143 Calr3 0.2978620890673793

ENSMUST00000019611 1857 Arhgef25 0.2737801041329241

ENSMUST00000019043 990 Irf1 0.3121095201623453

ENSMUST00000018630 1080 Wnt9b 0.38587636050061663

ENSMUST00000021259 3327 Gucy2e 0.2820140008632299

ENSMUST00000016086 2811 Gtf2ird2 0.32057909300058013

ENSMUST00000021313 696 Dcakd 0.3588010847248934

ENSMUST00000019050 1614 P4ha2 0.2814708378204981

ENSMUST00000020990 708 Pomc 0.3690484331550157

ENSMUST00000020008 1368 Gopc 0.2054626816708691

ENSMUST00000019999 6513 Arfgef3 0.2790798951806317

ENSMUST00000021866 1704 Riok1 0.21033851998339492

ENSMUST00000015797 1470 Steap2 0.21420813953245993

ENSMUST00000021950 2121 Dbn1 0.30299263408389837

ENSMUST00000018482 1944 Tnip1 0.33700169048976847

ENSMUST00000020886 1476 Nampt 0.16756131857282577

ENSMUST00000017454 480 Spint4 0.22223933165927914

ENSMUST00000021028 2364 Itgb3 0.3331709991981059

ENSMUST00000020630 2529 Hspa4 0.19077808348342928

ENSMUST00000020478 2169 Hcfc2 0.1767163660092266

ENSMUST00000018755 993 Pdlim4 0.29832552966986425

ENSMUST00000014747 1032 Alx3 0.3416495766767757

ENSMUST00000014777 315 Cpne8 0.25638540332766885

ENSMUST00000016338 879 Hsd11b1 0.24580444079379468

ENSMUST00000021333 1446 Foxg1 0.34830191624653134

ENSMUST00000014927 3546 Plekhg4 0.2654320251648784

ENSMUST00000020997 1023 Sh3yl1 0.15730877356163164

ENSMUST00000021362 1221 Klhdc2 0.18281306258813135

ENSMUST00000015486 1341 Xk 0.2622528834401885

ENSMUST00000019859 2100 Gle1 0.28828036022614345

ENSMUST00000015950 726 Qdpr 0.29674503374524663

ENSMUST00000017144 300 Svs6 0.1957950986977976

ENSMUST00000016491 2280 Kcnq2 0.30966237538868163

ENSMUST00000021888 1032 Ctsq 0.173443903507913

ENSMUST00000015605 2121 Atf6b 0.322513122292381

ENSMUST00000021851 1533 Fam217a 0.18988562875819465

ENSMUST00000019721 1239 Pdk4 0.24077527846528174

ENSMUST00000020208 4200 Fgd6 0.2105488121511989

ENSMUST00000020699 996 Gatsl3 0.30766519445220897

ENSMUST00000021331 1716 Klhl28 0.16912749430816512

ENSMUST00000020843 7038 Acaca 0.22451828513724892

ENSMUST00000020375 1287 Rab3ip 0.22870870989955172

ENSMUST00000019986 9369 Rev3l 0.15566461994451197

ENSMUST00000015578 747 Gzmg 0.26666321181359315

ENSMUST00000019697 1860 Haus5 0.27713115274627204

ENSMUST00000015394 1419 Mmp13 0.2202158001813011

ENSMUST00000021046 2790 Ddx42 0.20047713150587654

ENSMUST00000020531 429 Hba-x 0.39647753735774827

ENSMUST00000016400 921 Ctsz 0.34193783151977686

ENSMUST00000018353 1464 Stk4 0.23418799487438782

ENSMUST00000018739 1371 Glra4 0.22945003812089773

ENSMUST00000018871 573 Cpsf4l 0.2709566836312805

ENSMUST00000014698 834 Dguok 0.2763557806330888

ENSMUST00000021653 1299 Acot3 0.3194364147273606

ENSMUST00000020970 1089 Rsad2 0.2920051237492658

ENSMUST00000014445 378 Pam16 0.2981207328727345

ENSMUST00000017488 1437 Vtn 0.28444485608959114

ENSMUST00000021285 711 Stx8 0.21715319494272659

ENSMUST00000020263 1041 Hnrnph3 0.1744737414986916

ENSMUST00000019068 1992 Alox15 0.2892113118465211

ENSMUST00000021116 2433 Unk 0.32275479356504605

ENSMUST00000020982 1509 Klf11 0.2703119634910317

ENSMUST00000018429 2802 Pld2 0.28109632964731385

ENSMUST00000018988 966 Fndc8 0.2937282018112523

ENSMUST00000018156 579 Rac3 0.35069946017810855

ENSMUST00000016897 1272 Ttll1 0.3348927995916133

ENSMUST00000021506 1257 Serpina3n 0.30963760786269057

ENSMUST00000016977 432 Mrps18c 0.20197094759729084

ENSMUST00000021060 1380 Polg2 0.19803670657297207

ENSMUST00000019226 972 Slc25a22 0.341546752203947

ENSMUST00000021609 2190 Golga5 0.2101417772344123

ENSMUST00000017086 2436 Tmprss6 0.35046440707618437

ENSMUST00000020986 822 Dnajc27 0.24725816742184092

ENSMUST00000021166 573 Cygb 0.39385435248058637

ENSMUST00000020449 3225 Helb 0.22483206170353495

ENSMUST00000020258 3150 Herc4 0.15399195143140237

ENSMUST00000017841 1059 Ada 0.3380790907993898

ENSMUST00000020034 969 Echdc1 0.14605010811309535

ENSMUST00000020012 1578 Qrsl1 0.1934055169682315

ENSMUST00000015435 1344 Gdi1 0.2827317192307829

ENSMUST00000020702 879 Igfbp3 0.3218021998255272

ENSMUST00000021784 1353 Irf4 0.296365628332035

ENSMUST00000019992 5451 Lama4 0.23696130630634465

ENSMUST00000019638 975 Cops6 0.2805700294320948

ENSMUST00000016569 4341 Pds5b 0.17892032937445954

ENSMUST00000016680 2280 Cul4a 0.2784912031444373

ENSMUST00000016463 897 Slc25a5 0.27662740419558096

ENSMUST00000019290 972 Cacng2 0.34651436129212765

ENSMUST00000014063 744 Klk12 0.2671836664016126

ENSMUST00000021346 2295 Tshr 0.2577124264237199

ENSMUST00000021467 1161 Sav1 0.21755215176062165

ENSMUST00000020322 3189 Srgap1 0.291562665197002

ENSMUST00000021016 1677 Hnf1b 0.32351635989595945

ENSMUST00000020749 2184 Mtif2 0.1370304770339167

ENSMUST00000020002 246 Abracl 0.13912479666724642

ENSMUST00000019917 732 Rwdd1 0.1948239666161692

ENSMUST00000019281 333 Bcl2l11 0.21355927061167732

ENSMUST00000013995 6933 Abca4 0.26471303714337846

ENSMUST00000020255 1836 Slc5a8 0.18659374512216412

ENSMUST00000019183 1617 Dalrd3 0.24090385065128625

ENSMUST00000020899 1446 Matn3 0.284090604138259

ENSMUST00000017867 525 Wfdc2 0.25733176932993057

ENSMUST00000016781 561 Ift27 0.3085876549947505

ENSMUST00000019924 1020 Hey2 0.21945708604655007

ENSMUST00000018470 741 Ywhab 0.27265065653326503

ENSMUST00000015866 3336 Nlrp5 0.22579370048368066

ENSMUST00000018698 1080 Ybx2 0.260748375290961

ENSMUST00000020085 444 Ube2d1 0.1924435128255265

ENSMUST00000019684 681 Slc9a3r2 0.30818674736100427

ENSMUST00000020340 1968 Pcsk4 0.35709141787551313

ENSMUST00000020185 1641 Il20ra 0.21429728032752066

ENSMUST00000016309 930 Tmbim1 0.28965059870613036

ENSMUST00000020361 675 Ndufs7 0.3109157285972935

ENSMUST00000020999 2391 Kif3c 0.3364580264195326

ENSMUST00000020113 1431 Poc1b 0.2090182157910707

ENSMUST00000018466 930 Tomm34 0.2790861415386168

ENSMUST00000021273 351 Vamp2 0.37010019669647487

ENSMUST00000016088 990 Gatsl2 0.311352738650983

ENSMUST00000016081 1119 H2afy 0.2779733263158575

ENSMUST00000015893 747 Anp32e 0.23981436403828418

ENSMUST00000021425 1017 Ahsa1 0.2969694316670051

ENSMUST00000021329 639 Gosr2 0.3017486774073347

ENSMUST00000021082 603 Nt5c 0.37199994553257526

ENSMUST00000021937 885 Zfp346 0.29483786206016654

ENSMUST00000021283 2616 Pik3r5 0.3228521628178679

ENSMUST00000020071 2298 Sim1 0.251665695635087

ENSMUST00000020004 615 Asf1a 0.1605000748059064

ENSMUST00000021227 975 Ankrd40 0.2354162193697248

ENSMUST00000015234 570 Ptgds 0.3478141466437126

ENSMUST00000020885 732 Sypl 0.2039159327530214

ENSMUST00000021201 4134 Cpd 0.20793000269134898

ENSMUST00000020578 474 Nudcd2 0.25405631843600657

ENSMUST00000021168 1719 Wscd1 0.34240216952498337

ENSMUST00000020190 1503 Vnn3 0.23021235386363065

ENSMUST00000021641 3246 Tshz3 0.31279846594019206

ENSMUST00000019229 807 Med8 0.21708608044312053

ENSMUST00000017604 579 Cyba 0.3268819131000743

ENSMUST00000021284 1815 Ntn1 0.37441344374073365

ENSMUST00000021519 741 Six6 0.28345048116638394

ENSMUST00000021684 1503 Cyp46a1 0.33719607282201575

ENSMUST00000020640 954 Gnb2l1 0.2827818743986609

ENSMUST00000020552 912 Tpgs1 0.33557713827434443

ENSMUST00000021666 1821 Abcd4 0.3092706412020917

ENSMUST00000021134 1578 Tsen54 0.30394323990037475

ENSMUST00000020577 489 Fgf22 0.2933279183196805

ENSMUST00000018653 759 Cenpv 0.3179321469867521

ENSMUST00000017920 915 Crk 0.2481311012347596

ENSMUST00000021605 5931 Trip11 0.16758485231833356

ENSMUST00000020341 330 2310011J03Rik 0.33450961472732765

ENSMUST00000018614 1215 Kcnab3 0.23468789569254533

ENSMUST00000020182 957 Pex7 0.21310635487087534

ENSMUST00000020444 393 Llph 0.30580374931871096

ENSMUST00000020064 474 Fam162b 0.21836845184850404

ENSMUST00000015509 3564 Mov10l1 0.23161946366653777

ENSMUST00000021607 1308 Lgmn 0.3257571473739737

ENSMUST00000021009 3558 Myt1l 0.26790095737594205

ENSMUST00000019063 591 Tm4sf5 0.25833811007105456

ENSMUST00000018767 804 Mmp7 0.24435597719126478

ENSMUST00000020450 1971 Slc5a4a 0.24669980677630252

ENSMUST00000019266 369 Ccl9 0.22846995387142682

ENSMUST00000018699 855 Asgr1 0.2967078043601241

ENSMUST00000014499 5835 Anapc1 0.1856759569093222

ENSMUST00000021226 1476 Luc7l3 0.16170782581104345

ENSMUST00000015000 522 Tmem208 0.2776909613216865

ENSMUST00000019660 1686 Zkscan1 0.25189249757360005

ENSMUST00000017783 1908 Rab11fip4 0.3813046197130532

ENSMUST00000021290 609 Rcvrn 0.3583263813011446

ENSMUST00000020957 540 Adi1 0.3558770112519266

ENSMUST00000015498 1245 Pcolce2 0.24017785777966763

ENSMUST00000021405 453 Polr2h 0.2544928023123846

ENSMUST00000017908 1368 Zswim1 0.3569983606582371

ENSMUST00000017561 1503 Plxdc1 0.3214521052637713

ENSMUST00000021661 1431 Coq6 0.2568301049804739

ENSMUST00000015011 810 Surf4 0.3201075803030208

ENSMUST00000017430 897 Glod4 0.20185345737034968

ENSMUST00000021794 2502 Nedd9 0.26361790014581393

ENSMUST00000019443 3201 Rnf31 0.28325913944350195

ENSMUST00000015920 603 Med22 0.3196308049152853

ENSMUST00000020094 969 Epyc 0.19246978473324897

ENSMUST00000020759 1482 Efemp1 0.21377873984133924

ENSMUST00000020801 2976 Smg8 0.21130441826787777

ENSMUST00000021231 4569 Abcc3 0.29471365636852986

ENSMUST00000021548 951 Rdh12 0.24903714918663405

ENSMUST00000020523 1218 Pex13 0.15489926503038723

ENSMUST00000015433 447 Lage3 0.20578028280359262

ENSMUST00000019012 1299 Pnpla5 0.31957485042948847

ENSMUST00000021161 1719 Slc13a5 0.2797345179447718

ENSMUST00000015171 1563 Galns 0.304107781768206

ENSMUST00000021770 831 Scgn 0.23507090937836708

ENSMUST00000020243 696 Ascl1 0.32075555952676826

ENSMUST00000016498 1524 Srms 0.29383147425149486

ENSMUST00000016033 1836 Lta4h 0.1997960813170937

ENSMUST00000021797 882 Tbc1d7 0.22837983141855192

ENSMUST00000020463 1692 Ncln 0.35803191577488214

ENSMUST00000018748 2226 Tbx3 0.27575772605468923

ENSMUST00000018896 723 Tnfsf13 0.21158762262074382

ENSMUST00000020311 1452 Micu1 0.28046985470023517

ENSMUST00000020016 618 Gje1 0.20977232429582596

ENSMUST00000021453 1728 Tex21 0.20411170285815314

ENSMUST00000019439 1089 Tmem129 0.29028592859645136

ENSMUST00000019994 876 Popdc3 0.21222205298290425

ENSMUST00000019722 1329 Ubxn6 0.31698778968860386

ENSMUST00000020677 936 Upp1 0.30692491371862124

ENSMUST00000018993 3315 Wwc1 0.32124912877358364

ENSMUST00000016530 885 Mpped2 0.23888663902158905

ENSMUST00000017881 2193 Mmp9 0.3102434864937349

ENSMUST00000018315 1221 Vmp1 0.18582615303437985

ENSMUST00000020665 702 Med7 0.15416402217256509

ENSMUST00000019908 1122 Mtrf1l 0.23423331248141552

ENSMUST00000021885 375 Tpbpa 0.1805259182169086

ENSMUST00000017961 2091 Jph2 0.3411626150945587

ENSMUST00000021004 1620 Sntg2 0.24576572175364872

ENSMUST00000020461 1320 Nfic 0.362175540085209

ENSMUST00000017288 735 Rnd3 0.22083824035058505

ENSMUST00000021802 1431 Cap2 0.25654227054483436

ENSMUST00000018795 1332 Tada2a 0.22568652152332966

ENSMUST00000018561 3411 Myo1b 0.2281815111291287

ENSMUST00000018965 429 Avpi1 0.3427078820164129

ENSMUST00000021706 1704 Traf3 0.3157370172820747

ENSMUST00000015800 1941 Hspa8 0.25161023887142825

ENSMUST00000017458 1659 Mpp2 0.32082551428468503

ENSMUST00000021246 816 Snx11 0.27780347777214764

ENSMUST00000020508 411 Smim23 0.2718030237753322

ENSMUST00000015858 1143 Cers2 0.26956767868034354

ENSMUST00000021208 426 Rph3al 0.38304153266817725

ENSMUST00000021961 1188 Catsper3 0.27448173205194537

ENSMUST00000019037 465 Mb 0.35434049698471637

ENSMUST00000018521 1557 Vezf1 0.23108012739197714

ENSMUST00000017188 1200 Serpinb6a 0.25184054945161594

ENSMUST00000021631 2628 Ppp4r4 0.1730085958093225

ENSMUST00000021413 945 Nfkbia 0.33383153291096795

ENSMUST00000020649 3939 Rad50 0.19084134408420064

ENSMUST00000015511 5778 Plxnd1 0.3485013655042274

ENSMUST00000021011 294 Ccl7 0.2812825821964617

ENSMUST00000021494 2469 Ccdc175 0.171790702063101

ENSMUST00000017851 1419 Serinc3 0.23500982617032476

ENSMUST00000021495 1218 Serpina5 0.3035129548712743

ENSMUST00000020149 1122 Ikbip 0.20804453575301673

ENSMUST00000021179 606 Vmo1 0.27607100849544514

ENSMUST00000021728 528 Siva1 0.27816991509797434

ENSMUST00000021063 1371 Psmd12 0.21885841289898017

ENSMUST00000016294 8196 Tenm1 0.19900198101709715

ENSMUST00000020979 723 Bcap29 0.15504275409767865

ENSMUST00000015829 1299 Acadsb 0.20853736060075911

ENSMUST00000015622 543 Rnf5 0.289244824024844

ENSMUST00000020078 1014 Lims1 0.2720860480419791

ENSMUST00000018623 711 1700125H20Rik 0.25795560024607855

ENSMUST00000014891 1494 Ankrd53 0.2614107008304994

ENSMUST00000019975 1680 Wasf1 0.20754898400824395

ENSMUST00000021676 423 0610007P14Rik 0.2583607098517496

ENSMUST00000021048 2517 Ftsj3 0.24009853166992096

ENSMUST00000014686 1647 Clec4f 0.28055000770565086

ENSMUST00000021523 930 Mnat1 0.20190900812394771

ENSMUST00000018304 4683 Med1 0.21242619462213644

ENSMUST00000020045 7023 Ros1 0.18701877741148415

ENSMUST00000020022 1338 Smpdl3a 0.20692802026018126

ENSMUST00000021512 1017 Dhrs7 0.22699565324876467

ENSMUST00000020741 1104 Drg1 0.19643034615090213

ENSMUST00000017348 1341 Gsdma 0.2714840320354511

ENSMUST00000018572 2574 Akap1 0.2574744061626022

ENSMUST00000020528 1002 Mpg 0.2377635699654981

ENSMUST00000021963 885 Caml 0.23935921963924567

ENSMUST00000021941 621 Mxd3 0.36907784939759253

ENSMUST00000021903 480 Gadd45g 0.31729115821738524

ENSMUST00000019931 1452 Lrp11 0.26483092838396444

ENSMUST00000021335 1920 Scfd1 0.1664843282542646

ENSMUST00000015846 1038 Anxa9 0.23863620854154075

ENSMUST00000020161 972 Arg1 0.23472177971809383

ENSMUST00000018571 360 Ypel2 0.2303309455732698

ENSMUST00000021033 828 Rad51d 0.24799051837807007

ENSMUST00000017339 981 Zpbp2 0.16817438451858768

ENSMUST00000020339 2016 Tbc1d15 0.17469799654407916

ENSMUST00000021177 2160 Sec14l1 0.2918347690349704

ENSMUST00000020420 3600 Ap3d1 0.36150438357074477

ENSMUST00000020288 363 Eif4ebp2 0.25997885390151043

ENSMUST00000020550 708 Cdc34 0.40241608248772387

ENSMUST00000017148 369 Svs5 0.21658612315033351

ENSMUST00000019512 1212 Sec14l4 0.33442599032354664

ENSMUST00000020938 1626 Fam20a 0.3016077743035464

ENSMUST00000021076 672 Rab37 0.3120377890980645

ENSMUST00000021614 2355 Pfkp 0.2635578925748186

ENSMUST00000015227 507 C8g 0.2569427418588955

ENSMUST00000015449 3693 Sash1 0.27821325288840293

ENSMUST00000020106 3567 Arid5b 0.25589317544192075

ENSMUST00000019456 1608 Grb7 0.31659963514647027

ENSMUST00000015138 2583 Eln 0.1556104842012022

ENSMUST00000021837 1164 Serpinb9c 0.22116553177189172

ENSMUST00000021407 1515 Srp54a 0.15963995989451832

ENSMUST00000020101 2088 Rhobtb1 0.3052876254013405

ENSMUST00000020057 702 Lin7a 0.2261460364417308

ENSMUST00000017544 1227 Stac2 0.3238037223203755

ENSMUST00000021824 3498 Nol8 0.17714748873534109

ENSMUST00000019117 894 Hoxb1 0.2794449124011587

ENSMUST00000020927 1188 Hs1bp3 0.32565623476598865

ENSMUST00000020241 1362 Pah 0.2467739356289223

ENSMUST00000020977 975 Dus4l 0.1563356193805505

ENSMUST00000020399 1332 Cpm 0.25303218715710046

ENSMUST00000020679 1221 Nipal4 0.2899767316637283

ENSMUST00000018568 1095 Drg2 0.31555345674939406

ENSMUST00000021372 603 Atp5s 0.1656027103743958

ENSMUST00000020776 834 Ccdc117 0.23188353002455217

ENSMUST00000019662 1350 Ap4m1 0.23338020707160703

ENSMUST00000020040 510 Nts 0.2396070929849379

ENSMUST00000021707 1377 Amn 0.3111267522430702

ENSMUST00000021854 1077 Eci2 0.2248699123595506

ENSMUST00000019470 765 Psme3 0.22857647163555903

ENSMUST00000017276 1194 Cyth1 0.33899866494919356

ENSMUST00000021323 1149 1700023F06Rik 0.298072990022975

ENSMUST00000019962 594 Cd164 0.21900435178287295

ENSMUST00000016907 3057 Scube1 0.3442033464845194

ENSMUST00000015712 1425 Lpl 0.27531964711171336

ENSMUST00000021559 315 Erh 0.28934650490481545

ENSMUST00000021779 684 Prl4a1 0.2193370264778646

ENSMUST00000016406 1134 Sertad4 0.2536477531561848

ENSMUST00000017741 1464 Krt12 0.25783538233769815

ENSMUST00000015236 447 Edf1 0.34117627523606836

ENSMUST00000021813 765 Barx1 0.308360429092248

ENSMUST00000020488 558 D10Wsu102e 0.22669557326459408

ENSMUST00000018816 618 Copz2 0.2777917237922036

ENSMUST00000019991 1428 Tube1 0.2240646265776335

ENSMUST00000020856 1260 Bzw2 0.24487918153854038

ENSMUST00000019220 2283 Strn4 0.2968544784022788

ENSMUST00000018798 1287 Ikzf1 0.33055432899879017

ENSMUST00000015796 1020 Steap1 0.19782942409887264

ENSMUST00000015124 507 Tsen15 0.18155775582665332

ENSMUST00000019939 489 Snx3 0.21537160629859575

ENSMUST00000021347 2373 Sel1l 0.23603087407328183

ENSMUST00000019441 1911 Nop9 0.21045786810577302

ENSMUST00000020617 4092 Flt4 0.3366107650109042

ENSMUST00000020286 870 Ppa1 0.24729004154498924

ENSMUST00000015576 735 Mcpt2 0.1802749121669058

ENSMUST00000016231 1359 Fli1 0.32468962112928074

ENSMUST00000021544 1062 Plek2 0.27947996879504994

ENSMUST00000019944 576 Adat2 0.24246444229163494

ENSMUST00000021148 513 Ube2g1 0.16136232281983198

ENSMUST00000017732 1347 Krt27 0.32216648260046743

ENSMUST00000021552 1017 Zfp36l1 0.36406932560416966

ENSMUST00000021087 669 Mif4gd 0.36067743900543725

ENSMUST00000019576 1284 Ddx39 0.31192483030541307

ENSMUST00000018246 381 Hist1h2bc 0.4794734753936948

ENSMUST00000016703 411 H3f3b 0.326598415418792

ENSMUST00000014221 588 Chp1 0.2525934119928064

ENSMUST00000019268 1245 Scrn1 0.23455957188588397

ENSMUST00000018382 1326 Gdf9 0.2786965719932365

ENSMUST00000021411 1755 1110008L16Rik 0.21119675450961545

ENSMUST00000015771 1215 Gata5 0.3036267647882347

ENSMUST00000019977 1026 Ddo 0.2502774007170385

ENSMUST00000020537 516 Nsg2 0.3329564732749808

ENSMUST00000021241 723 Dlx4 0.2911677574730258

ENSMUST00000019927 1383 Trmt11 0.1658459253993065

ENSMUST00000020922 1032 Trib2 0.2790308910612685

ENSMUST00000018430 717 Psmb6 0.2501738464383311

ENSMUST00000019503 1620 Gdpd2 0.2694807947391946

ENSMUST00000019074 279 Ccl4 0.310783647254543

ENSMUST00000020705 1755 Pes1 0.31124469337194716

ENSMUST00000021091 1233 Pafah1b1 0.1696040516696444

ENSMUST00000019920 984 Clvs2 0.2218194009213611

ENSMUST00000019861 3171 Zfp451 0.1913266220837088

ENSMUST00000017549 2097 Nek8 0.32777416272054144

ENSMUST00000020706 3357 Adcy1 0.30847732527390376

ENSMUST00000020252 765 Sycp3 0.15265645391518917

ENSMUST00000018485 2625 Il12rb2 0.2016818766469568

ENSMUST00000015587 747 Gzmn 0.2268794123351983

ENSMUST00000021204 1308 Nxn 0.31847098654173406

ENSMUST00000019749 870 Hoxd8 0.25071617215419634

ENSMUST00000018337 1596 Cdc73 0.1604544730995008

ENSMUST00000019426 3126 Dsg4 0.2101200289723504

ENSMUST00000017751 2091 Tns4 0.3047317402337052

ENSMUST00000021620 705 Otub2 0.2882659575229685

ENSMUST00000021097 2949 Recql5 0.2821640367112223

ENSMUST00000021898 1425 Shc3 0.29221653179813134

ENSMUST00000016678 1248 Lamp2 0.18643776420878566

ENSMUST00000020827 1188 Rnft1 0.14188238425321012

ENSMUST00000014691 1203 Wdfy2 0.2739552941147227

ENSMUST00000020794 363 Ska2 0.14436346388349325

ENSMUST00000021458 6990 Sptb 0.341843679175827

ENSMUST00000018437 423 Pfn1 0.26687189087554236

ENSMUST00000020379 1950 Tcf3 0.31996800662665414

ENSMUST00000020804 945 Gdpd1 0.22230355613561587

ENSMUST00000019374 993 Lhx9 0.31156176866433216

ENSMUST00000015333 2394 Casd1 0.18309972100830887

ENSMUST00000020197 1251 Eya3 0.22603248001757706

ENSMUST00000017743 1296 Krt20 0.2596608324668431

ENSMUST00000021726 1374 Adssl1 0.33602526430045876

ENSMUST00000018313 966 Mfng 0.32824136788408403

ENSMUST00000020543 2190 Cpeb4 0.19723831312640866

ENSMUST00000017090 705 Kctd5 0.2753452211815684

ENSMUST00000016279 717 N4bp2l1 0.2599339958021983

ENSMUST00000021564 1359 Smoc1 0.29865521708445525

ENSMUST00000015581 744 Gzmb 0.22653769034471824

ENSMUST00000021490 1236 Serpina1f 0.24645322192778096

ENSMUST00000021482 459 Tomm20l 0.2209386134964018

ENSMUST00000018841 1581 Aatf 0.21125382889572406

ENSMUST00000019026 921 Mrpl45 0.20683624726636005

ENSMUST00000020392 456 9530003J23Rik 0.21775109423702937

ENSMUST00000018990 1113 Pank3 0.15079727814251745

ENSMUST00000017975 648 Rab5a 0.14261720837227163

ENSMUST00000021957 2337 Fam193b 0.29996467586539133

ENSMUST00000020413 1227 Zpbp 0.17765839604037448

ENSMUST00000020270 2205 Ddx50 0.15227510305689093

ENSMUST00000017864 717 Trp53tg5 0.2264135791200486

ENSMUST00000021891 1002 Cts8 0.20670920842458174

ENSMUST00000020062 2787 Gprc6a 0.21732409004480327

ENSMUST00000020974 405 Id2 0.34711294091201467

ENSMUST00000019405 2922 Map1s 0.29568332504752187

ENSMUST00000014578 2439 Plg 0.26829913868322297

ENSMUST00000021921 4305 Ptch1 0.2605173554064042

ENSMUST00000020123 2082 Tmpo 0.19387079582799294

ENSMUST00000021807 1143 Dek 0.18950873957295128

ENSMUST00000021050 1146 Adap2 0.29532451470244764

ENSMUST00000021497 627 Rtn1 0.32707634232569843

ENSMUST00000021918 2835 Ror2 0.3552232493352302

ENSMUST00000019231 1392 Atp6ap1 0.2643453778679636

ENSMUST00000016471 1071 Atp1b4 0.24450014262351932

ENSMUST00000021424 1683 Sptlc2 0.2179424644529656

ENSMUST00000015197 1443 Gata2 0.30251541872172316

ENSMUST00000021056 5526 Scn4a 0.38341300977378423

ENSMUST00000017208 693 Prl5a1 0.21084589612996038

ENSMUST00000020490 942 Wdr82 0.2581325202858582

ENSMUST00000021634 972 Akr1c13 0.20820236510388837

ENSMUST00000017759 951 Tnfaip1 0.3314143350944747

ENSMUST00000021262 2034 Alox8 0.28641159026987084

ENSMUST00000016771 5883 Myh9 0.4095234384257747

ENSMUST00000019067 354 Med11 0.3299505002831717

ENSMUST00000021090 654 Grb2 0.29685507865414373

ENSMUST00000020668 846 Havcr2 0.20328550066606316

ENSMUST00000014321 618 Tvp23b 0.24553231762119818

ENSMUST00000016452 459 Ube2a 0.1825575720217365

ENSMUST00000015239 1722 Fbxw5 0.326257908226436

ENSMUST00000015003 1233 E2f4 0.29389836233279676

ENSMUST00000021785 2775 Exoc2 0.18100061855192265

ENSMUST00000017904 1479 Ctsa 0.3150744708687761

ENSMUST00000014917 2169 Dll1 0.3454229485064682

ENSMUST00000014290 2013 Apbb1ip 0.19337660543593152

ENSMUST00000015723 957 Nkx2-5 0.3652087955905449

ENSMUST00000020039 1437 Mgat4c 0.1382679789650277

ENSMUST00000021306 2919 Eftud2 0.30781114280044836

ENSMUST00000020985 1611 Grhl1 0.27919652009851004

ENSMUST00000016553 1248 Nkap 0.20703709602601944

ENSMUST00000020898 528 Agr2 0.22453914920699608

ENSMUST00000021572 1491 Wdr37 0.18235664743060817

ENSMUST00000014220 435 Tctex1d2 0.22709020381498343

ENSMUST00000021078 1485 Fdxr 0.30095030371836695

ENSMUST00000020248 585 Ccdc53 0.19902348441195725

ENSMUST00000020928 1578 Arsg 0.26125719346607174

ENSMUST00000021822 897 Ogn 0.14026109194723055

ENSMUST00000020309 1131 Dnajb12 0.3417202716425074

ENSMUST00000021800 1023 Mcur1 0.23922560055845798

ENSMUST00000014614 714 Rnf166 0.413728188274659

ENSMUST00000018651 2271 Trpv2 0.29368971320201787

ENSMUST00000021119 939 Aspa 0.15987216832720336

ENSMUST00000020767 1491 Polm 0.2640742147131982

ENSMUST00000021277 1038 Aurkb 0.3096202112252344

ENSMUST00000020103 927 1700040L02Rik 0.2230362549308456

ENSMUST00000020374 330 Cnot2 0.17830231299931865

ENSMUST00000018727 1398 G3bp1 0.25753551189060403

ENSMUST00000021083 465 Hn1 0.2669633948339767

ENSMUST00000020502 1434 Slc36a3 0.35264603107076903

ENSMUST00000020568 2169 Wdpcp 0.20193510547179833

ENSMUST00000016110 1500 Il17rb 0.2588412620629558

ENSMUST00000020551 1578 Asb3 0.14800831282597646

ENSMUST00000014743 1659 Csf1 0.2807490583156598

ENSMUST00000018094 1425 Hnf4a 0.3620113892278102

ENSMUST00000018966 945 Sfrp5 0.3966180150711133

ENSMUST00000019232 945 Dnase1l1 0.238705714882011

ENSMUST00000020163 1983 Nedd1 0.18505065341799865

ENSMUST00000020846 3405 Srebf1 0.30201768891511793

ENSMUST00000019514 450 Calm3 0.33795169766445515

ENSMUST00000019982 684 Gtf3c6 0.22747854656352667

ENSMUST00000020703 1332 Gabra6 0.18620820551898593

ENSMUST00000021410 1362 Ppp2r3c 0.1771039454908103

ENSMUST00000019942 789 Aig1 0.2512501917033487

ENSMUST00000020203 261 Snrpf 0.24844424941498927

ENSMUST00000016172 9105 Celsr1 0.3176257221907667

ENSMUST00000020991 2727 Dnmt3a 0.3444421560622861

ENSMUST00000017981 576 Nkiras2 0.40124051636374264

ENSMUST00000018295 1251 Pick1 0.31529915980653983

ENSMUST00000021816 774 Susd3 0.3056679660421634

ENSMUST00000016168 1446 Lbp 0.29143848725078536

ENSMUST00000015157 420 Trappc2l 0.388809767905771

ENSMUST00000020454 954 Hmg20b 0.33221310944439

ENSMUST00000015719 246 Atp6v0e 0.2994630615127831

ENSMUST00000016427 915 H2-M2 0.29811567875765105

ENSMUST00000020530 1710 Nprl3 0.2936283690827335

ENSMUST00000016115 1875 Actr8 0.23522720207444864

ENSMUST00000016401 588 Slmo2 0.24014542567606198

ENSMUST00000014339 1485 Dnajc7 0.23771914459886134

ENSMUST00000018875 2856 Ap2b1 0.22903150570510186

ENSMUST00000015594 744 Mcpt8 0.22755551743058075

ENSMUST00000021377 1059 Cdkl1 0.2490265520747224

ENSMUST00000018700 2175 Dlg4 0.30801548490865516

ENSMUST00000020365 2049 Mum1 0.2926932581359566

ENSMUST00000018880 1038 Ndel1 0.16120008089938584

ENSMUST00000020864 645 Pctp 0.2911212191680272

ENSMUST00000019906 516 Vip 0.23643677476505

ENSMUST00000014058 837 Klk10 0.29697150795105204

ENSMUST00000020448 1830 Irak3 0.20605856924660498

ENSMUST00000015663 1014 2310057J18Rik 0.16779790532422204

ENSMUST00000020717 543 Arf5 0.35448309352996404

ENSMUST00000021617 1905 Asb2 0.35509799104898404

ENSMUST00000020967 390 Pfn4 0.2101676937760971

ENSMUST00000018333 987 Uchl5 0.14811846403743165

ENSMUST00000019060 426 Csf2 0.2513992776665062

ENSMUST00000019109 741 Ywhah 0.27015582947839184

ENSMUST00000021468 6108 Nin 0.24995651715304454

ENSMUST00000015585 747 Gzmc 0.23415239215966782

ENSMUST00000020285 597 Sar1a 0.1871445007202637

ENSMUST00000018478 2622 Ksr1 0.35016324114800745

ENSMUST00000014848 1119 Hoxa2 0.2562469549113131

ENSMUST00000020830 1050 Mfap3 0.24087754419450935

ENSMUST00000021190 1416 Coro6 0.3016015320426537

ENSMUST00000018392 720 Prl8a6 0.14698889370235446

ENSMUST00000019317 705 Rab5c 0.288420649391232

ENSMUST00000021596 3519 Nrde2 0.2889954498628906

ENSMUST00000019464 1050 Noxo1 0.23691670147563298

ENSMUST00000019379 1635 Rps6kl1 0.2973215393513858

ENSMUST00000021930 969 Sfxn1 0.230193654426529

ENSMUST00000020277 2748 Hkdc1 0.3575101561851146

ENSMUST00000021370 1395 L2hgdh 0.19158712985045895

ENSMUST00000018765 1398 Mmp8 0.19715827267126615

ENSMUST00000020822 879 Cnot8 0.2811219957174753

ENSMUST00000020971 879 Rnf144a 0.3212411702164726

ENSMUST00000020024 399 Fabp7 0.26866454479116714

ENSMUST00000020527 591 1700093K21Rik 0.21867646478642466

ENSMUST00000015146 2460 Efr3a 0.1809833811769532

ENSMUST00000017552 1794 Cacnb1 0.31374025358623503

ENSMUST00000021773 2529 Gpld1 0.26811763106140585

ENSMUST00000021922 804 Msx2 0.28085590347938694

ENSMUST00000014647 1866 Pkd2l2 0.21404064202082057

ENSMUST00000015628 507 Fam163a 0.31925355323131926

ENSMUST00000019051 1989 Alox12e 0.2862253409400856

ENSMUST00000015583 786 Ctsg 0.2757928763228037

ENSMUST00000019199 2187 Plod1 0.34208233993888787

ENSMUST00000019075 333 Natd1 0.403457847193753

ENSMUST00000021486 270 Timm9 0.21157463304481205

ENSMUST00000021889 1005 Ctsr 0.20862541243003924

ENSMUST00000021155 1257 Tekt1 0.3316344689391868

ENSMUST00000018877 1080 Pex12 0.19811990850727929

ENSMUST00000019044 1674 Slc22a5 0.27812826444151173

ENSMUST00000018531 2283 Shroom1 0.25569669582073246

ENSMUST00000020212 2079 Cep83 0.14989922286164725

ENSMUST00000020575 771 Fstl3 0.3416154032527111

ENSMUST00000021603 1347 Fbln5 0.2820336613783062

ENSMUST00000019058 501 Il3 0.23678794818481608

ENSMUST00000020343 669 Rab21 0.20182543067281314

ENSMUST00000018985 990 Rad51d 0.2570130887587341

ENSMUST00000017530 1413 Traf4 0.3573684389632791

ENSMUST00000021234 6819 Cacna1g 0.3350433397797458

ENSMUST00000015271 720 Unkl 0.26155301928370905

ENSMUST00000019677 1248 Mknk1 0.2789537500937368

ENSMUST00000015277 6045 Lrrk1 0.3136406566791025

ENSMUST00000020535 438 Hbq1a 0.2986912170607988

ENSMUST00000016901 1920 Ttll12 0.3851280715690894

ENSMUST00000019143 996 Slc35b4 0.25859098971185684

ENSMUST00000021900 2586 Sema4d 0.29062183575327527

ENSMUST00000017147 798 Svs3a 0.20570371520662598

ENSMUST00000021324 2829 Map3k14 0.31161915170940596

ENSMUST00000019291 1419 Psg28 0.1921613623997847

ENSMUST00000017453 864 Cd300lg 0.30816834329165377

ENSMUST00000019615 1140 Cdc37 0.3937317221501849

ENSMUST00000020023 765 Reep3 0.23317603421717958

ENSMUST00000020188 1434 Ifngr1 0.20473938769974334

ENSMUST00000021527 2052 Prkch 0.27687021996102734

ENSMUST00000020931 3294 Smc6 0.14444767086114194

ENSMUST00000020278 1155 Tacr2 0.36639918722388476

ENSMUST00000021036 1008 Rffl 0.33745536243411783

ENSMUST00000021682 2004 Angel1 0.2914241082951408

ENSMUST00000019633 588 Cd70 0.30951283507527066

ENSMUST00000017946 1401 Fam134c 0.26420178770380814

ENSMUST00000019878 786 Leng1 0.2857684515063595

ENSMUST00000021052 1596 Smarcd2 0.29472143716382626

ENSMUST00000020734 2880 Eif4enif1 0.18868680892515305

ENSMUST00000020497 2772 Aldh1l2 0.2657045581531111

ENSMUST00000020755 2463 Smek2 0.14531280022554466

ENSMUST00000020174 786 Stx7 0.20361851828072242

ENSMUST00000021170 537 Mxra7 0.3073656694820187

ENSMUST00000018122 3915 Cadps2 0.2122190587185737

ENSMUST00000021147 2094 Exoc7 0.3664171196994979

ENSMUST00000018681 1029 Pcgf2 0.30967716450200594

ENSMUST00000019950 1413 Ltv1 0.2194215601229956

ENSMUST00000016125 1467 Stk32c 0.3415932041400831

ENSMUST00000020877 993 Twistnb 0.2012530328668399

ENSMUST00000021332 675 Fkbp3 0.17987884183897881

ENSMUST00000015545 669 Tmem27 0.18627553356183266

ENSMUST00000015725 687 Bnip1 0.259165511334957

ENSMUST00000018711 354 Gabarap 0.23979754477129217

ENSMUST00000019987 1668 Traf3ip2 0.2580636532657841

ENSMUST00000021832 1983 Wrnip1 0.2817064266708547

ENSMUST00000018821 1080 Slc25a39 0.3304844133398397

ENSMUST00000021380 477 Trappc6b 0.17742147539456707

ENSMUST00000015934 921 Surf1 0.2213215249766871

ENSMUST00000016124 1572 Lrrc27 0.24830579660677546

ENSMUST00000020753 1791 Clhc1 0.1527382068977266

ENSMUST00000016396 159 Atp5e 0.2863148872347764

ENSMUST00000014694 2352 Rbsn 0.25559762333285463

ENSMUST00000020629 2049 Gfpt2 0.2609643384764035

ENSMUST00000021045 1692 Asic2 0.3107820491400364

ENSMUST00000021479 1254 Actr10 0.16455225521536876

ENSMUST00000015667 1026 Ctss 0.2550920574799683

ENSMUST00000021761 1542 Trim27 0.29684206350615244

ENSMUST00000021466 1677 Atl1 0.21735068931397006

ENSMUST00000017839 1254 Rnf135 0.2732097683513977

ENSMUST00000019192 591 Cryaa 0.3595008936973126

ENSMUST00000016571 213 Ndufa1 0.28187979209414815

ENSMUST00000020257 2214 Sirt1 0.15756244458041593

ENSMUST00000021939 1041 Cdk20 0.32450142203376

ENSMUST00000015100 984 Ppp1cb 0.16424496207629583

ENSMUST00000020397 381 Snrpd3 0.23529752181862573

ENSMUST00000014457 231 Cox6c 0.2480421786881847

ENSMUST00000020372 171 Uqcr11 0.4355501162171235

ENSMUST00000020262 867 Pbld2 0.26493586745514547

ENSMUST00000020853 321 Lrrc72 0.20620341719360993

ENSMUST00000021738 1158 Gpr137b 0.2804713899489892

ENSMUST00000017891 1599 Ghdc 0.28163851432775877

ENSMUST00000021714 705 Zfyve21 0.3132631817652196

ENSMUST00000018743 1365 Mief2 0.3070036768732827

ENSMUST00000021606 1068 Atxn3 0.1651823238892309

ENSMUST00000018989 2790 Unc45b 0.3523754065731974

ENSMUST00000020283 1119 H2afy2 0.29926067445460863

ENSMUST00000017694 5481 Atad5 0.15986233622890766

ENSMUST00000018491 1671 Zkscan6 0.2540383760337314

ENSMUST00000019065 3372 Pelp1 0.22772399679359925

ENSMUST00000019862 1065 L3hypdh 0.2629773997846574

ENSMUST00000020820 621 Mrpl22 0.21586493712405522

ENSMUST00000017622 2919 Zc3h18 0.2618678441354999

ENSMUST00000020323 1272 Avpr1a 0.3107848781999213

ENSMUST00000017332 327 Coa3 0.24818540609500062

ENSMUST00000017153 597 Sdc4 0.3268810402663378

ENSMUST00000017821 1266 Wsb1 0.15552923036884167

ENSMUST00000021130 486 Ten1 0.2675594138738909

ENSMUST00000018403 723 Prl8a2 0.15841128235304106

ENSMUST00000014118 552 Mcemp1 0.22945975609356448

ENSMUST00000014996 3114 Adamts13 0.2902680606987446

ENSMUST00000020687 600 Pttg1 0.22043587654136707

ENSMUST00000020835 1797 Ppm1d 0.22082041833290755

ENSMUST00000019683 987 Rcn3 0.35025861621413606

ENSMUST00000021345 1137 Gtf2a1 0.1688741422502036

ENSMUST00000020383 4008 Atp8b3 0.33418582791429335

ENSMUST00000020017 5076 Aim1 0.23183711830228115

ENSMUST00000020165 1341 Pde7b 0.24112861798970453

ENSMUST00000021792 1869 Mak 0.22469329080684547

ENSMUST00000014545 999 Ldhc 0.19963370562635344

ENSMUST00000020965 1245 Allc 0.2085751721264858

ENSMUST00000021645 1560 Dcaf4 0.307625151564797

ENSMUST00000020547 2598 Vmn2r81 0.16439300005403054

ENSMUST00000020769 1311 Dbnl 0.3457401062257859

ENSMUST00000019974 672 Rab32 0.2616037837281503

ENSMUST00000015358 2010 Mtmr1 0.18535993839660372

ENSMUST00000019038 1731 Sept9 0.36512633151433577

ENSMUST00000019224 1239 Mfsd3 0.23926722386509572

ENSMUST00000021030 1170 Mettl2 0.2292904762780257

ENSMUST00000020439 1140 Wif1 0.2553913012722124

ENSMUST00000021554 2679 Actn1 0.34957701702203536

ENSMUST00000018909 2025 Fxr2 0.2568485855504923

ENSMUST00000018963 993 Lsp1 0.26687429702736404

ENSMUST00000020099 894 Cdk1 0.22774592388614753

ENSMUST00000021803 4389 Nup153 0.173678693569745

ENSMUST00000021828 471 Nxnl2 0.3606455611093786

ENSMUST00000020456 621 4930404N11Rik 0.3290830468143703

ENSMUST00000020634 1272 Mapk9 0.18299618841794466

ENSMUST00000020346 654 Thap2 0.15961303608566277

ENSMUST00000017548 591 Rpl19 0.3838709539829266

ENSMUST00000020647 1008 3010026O09Rik 0.25191648872621464

ENSMUST00000016023 2829 Fam184b 0.2953514352527762

ENSMUST00000019516 777 Nrbp2 0.3011801392436611

ENSMUST00000020316 2190 Tbk1 0.20189287393027747

ENSMUST00000021428 1611 Snw1 0.2097420329667984

ENSMUST00000016673 537 Il10 0.2970744059254248

ENSMUST00000021574 1905 Gtpbp4 0.18839257035498375

ENSMUST00000017443 987 Dnttip1 0.27772625946813345

ENSMUST00000020159 4113 Med23 0.23059902907543425

ENSMUST00000020000 618 Hebp2 0.2538375967898689

ENSMUST00000015891 1713 Vps45 0.21361116998012294

ENSMUST00000016344 1725 Syt14 0.197039790465544

ENSMUST00000019854 651 Mrpl24 0.2154791692313529

ENSMUST00000020403 582 Csrp2 0.2715450483160204

ENSMUST00000021314 1491 Nmt1 0.3209434495852357

ENSMUST00000021734 228 Gng4 0.4937187599016073

ENSMUST00000021639 1341 Tubal3 0.21777893546145335

ENSMUST00000014421 7812 Ankrd17 0.16511855591898494

ENSMUST00000015460 1032 Slamf1 0.23882009681797134

ENSMUST00000019198 459 Fis1 0.3310154546323647

ENSMUST00000021089 957 Slc25a19 0.29469467333037763

ENSMUST00000018685 1251 Cwc25 0.3162608671527614

ENSMUST00000017365 1593 Psmd3 0.33214327486792733

ENSMUST00000018361 483 Pmp22 0.3135425739340484

ENSMUST00000021249 1278 Scrn2 0.3247090083940142

ENSMUST00000018593 660 Rpain 0.2573880795118701

ENSMUST00000015791 11157 Lama5 0.31509952635373667

ENSMUST00000019276 690 BC005537 0.23197105538757581

ENSMUST00000018522 1167 Cuedc1 0.3147358796943972

ENSMUST00000021674 1143 Fos 0.318088751071069

ENSMUST00000014892 591 Tex261 0.3169438065580361

ENSMUST00000016323 1434 Camk1g 0.3188034395459299

ENSMUST00000019998 582 Perp 0.3621465580877392

ENSMUST00000021880 414 Ctla2a 0.2550705543682604

ENSMUST00000021630 972 Akr1c6 0.1858668910051079

ENSMUST00000020268 3441 Ccar1 0.15229897114080312

ENSMUST00000016138 1134 Fnta 0.19297024082567862

ENSMUST00000020150 1038 Ikbip 0.1754609883870824

ENSMUST00000020959 858 Rnaseh1 0.28695667166043604

ENSMUST00000019378 4236 Mlh3 0.16493638304286115

ENSMUST00000020446 717 Tmbim4 0.2272631834094253

ENSMUST00000021907 1020 Fbp2 0.2823036170273968

ENSMUST00000018625 1758 Appbp2 0.19490175853494823

ENSMUST00000016087 414 Bola1 0.24562323584290455

ENSMUST00000021692 1245 Yy1 0.3388121273752961

ENSMUST00000021142 3117 Atp2a3 0.3245657400839085

ENSMUST00000021443 2808 Mthfd1 0.2476298703626802

ENSMUST00000018637 5829 Myh1 0.35557297636611795

ENSMUST00000016640 873 Cd274 0.2385114633517597

ENSMUST00000020287 1299 Npffr1 0.37529562782057957

ENSMUST00000021610 1392 Chga 0.33795037968321084

ENSMUST00000020653 597 Sar1b 0.186542737641234

ENSMUST00000019572 1344 Zfp874b 0.18560227764194168

ENSMUST00000021217 459 Nme2 0.3641733930307359

ENSMUST00000014684 720 U2af1 0.26223889521093924

ENSMUST00000021390 2055 Galc 0.1993953498236858

ENSMUST00000020909 927 Laptm4a 0.24834608549015177

ENSMUST00000021646 3843 Papln 0.3077890521476484

ENSMUST00000014389 759 Pigl 0.27035421013211375

ENSMUST00000015540 591 Cd83 0.2689831896331713

ENSMUST00000017142 342 Svs4 0.19518518971283633

ENSMUST00000015612 5895 Notch4 0.3071501690021771

ENSMUST00000014913 723 Psmb1 0.2458527466837841

ENSMUST00000017309 306 Gast 0.3004281198168803

ENSMUST00000017455 297 Pyy 0.3772308229400259

ENSMUST00000015467 975 Slc39a1 0.2740193384756847

ENSMUST00000017637 765 Igfbp4 0.40992963078544836

ENSMUST00000014505 2985 Mertk 0.23266127172877266

ENSMUST00000021187 1131 Dhrs13 0.27434968164010415

ENSMUST00000021942 654 Prelid1 0.33039659977295494

ENSMUST00000019882 378 Polr2i 0.3906813362342231

ENSMUST00000021368 3195 Nemf 0.16180303387906078

ENSMUST00000016654 831 Mtif3 0.23972010928049503

ENSMUST00000020549 795 Gzmm 0.2969067502407602

ENSMUST00000021530 2511 Hif1a 0.16831886022897816

ENSMUST00000018577 2172 Nol11 0.15694032108422676

ENSMUST00000020015 1173 Nmbr 0.2493268798903308

ENSMUST00000018713 636 Cldn7 0.27796427230505166

ENSMUST00000021343 738 Tshr 0.2074411900483476

ENSMUST00000018506 1590 Kpna2 0.17412489473491533

ENSMUST00000016026 1803 Lcorl 0.14764085710067518

ENSMUST00000016638 1149 Cd34 0.22253003655928097

ENSMUST00000020707 1368 Gabra1 0.19179116928980483

ENSMUST00000021652 1266 Acot4 0.2792331002188778

ENSMUST00000015941 1092 Bhmt2 0.25975789038974767

ENSMUST00000014640 3162 Ankrd28 0.1559683491649757

ENSMUST00000021669 597 Fcf1 0.24575285780910605

ENSMUST00000021860 489 Ly86 0.23577811095152568

ENSMUST00000020672 387 Fabp6 0.38163178926942903

ENSMUST00000020779 2157 Mpo 0.333887677876335

ENSMUST00000019248 2607 Cd22 0.2781499325726116

ENSMUST00000018800 582 Myl4 0.3569219636610604

ENSMUST00000020580 3624 Polrmt 0.3455767055874727

ENSMUST00000020312 1053 Mcu 0.24276892094607114

ENSMUST00000020157 3750 Apaf1 0.18765672211363935

ENSMUST00000021207 651 Fam101b 0.3063758832700245

ENSMUST00000019071 351 Ccl6 0.22831741663385433

ENSMUST00000020538 3216 Xpo1 0.1313864202240172

ENSMUST00000021567 7035 Pcnx 0.24292691615539222

ENSMUST00000015137 1944 Limk1 0.3376968591319884

ENSMUST00000016951 1149 Serpinb1b 0.23948985911043233

ENSMUST00000019006 1791 Otop3 0.344433898028565

ENSMUST00000020566 1128 Theg 0.29150070209252493

ENSMUST00000021001 603 Rab10 0.22463262129610198

ENSMUST00000021693 921 Slc25a29 0.32095193469736344

ENSMUST00000020220 1977 Nuak1 0.3011480400432181

ENSMUST00000021857 1356 Fars2 0.27851722762910053

ENSMUST00000021956 1869 Ddx41 0.323634589780969

ENSMUST00000021522 459 Glrx5 0.33781964512753676

ENSMUST00000021970 300 Cxcl14 0.46983087366954546

ENSMUST00000021632 972 Akr1c12 0.20118239791818973

ENSMUST00000019482 3714 Zfp687 0.2691100829142407

ENSMUST00000020234 636 Timp3 0.3713264525051276

ENSMUST00000019723 501 Mydgf 0.3211611562907

ENSMUST00000020378 2010 Best3 0.2625095215486467

ENSMUST00000021647 1815 Numb 0.23830171934332148

ENSMUST00000021929 1239 Habp4 0.2952324688605606

ENSMUST00000019932 723 1700021F05Rik 0.23488495586253746

ENSMUST00000021157 396 Med31 0.2192462294886147

ENSMUST00000021307 714 Ccdc103 0.2690022695874609

ENSMUST00000014370 690 Cacybp 0.1968259323768512

ENSMUST00000019734 753 Cyb561 0.3129005089456

ENSMUST00000021158 372 Txndc17 0.28905101813809475

ENSMUST00000020437 2127 Mdm1 0.21409659217911023

ENSMUST00000014920 663 Nol3 0.2967052793222794

ENSMUST00000020664 1860 Itk 0.2760570593717519

ENSMUST00000015484 1713 Cybb 0.24999112864258421

ENSMUST00000020273 2340 Supv3l1 0.2312673163288513

ENSMUST00000020217 540 Nudt4 0.23524125593204692

ENSMUST00000021920 1422 Sptlc1 0.2199735968306155

ENSMUST00000019913 1539 Frk 0.19126524804416628

ENSMUST00000019708 1806 Arid3a 0.3276317170428093

ENSMUST00000017576 1125 Rbfox3 0.26860627453538094

ENSMUST00000018914 426 Atp5g3 0.1974179059446428

ENSMUST00000017629 4839 Top2b 0.1551597780359598

ENSMUST00000018287 471 Mafk 0.3538079385111377

ENSMUST00000021668 450 Npc2 0.3306262069248141

ENSMUST00000018113 1506 Ptgis 0.3027690591623362

ENSMUST00000020077 1191 Lims1 0.26607744221082047

ENSMUST00000021818 861 Cenpp 0.1761788404361494

ENSMUST00000021628 972 Akr1c21 0.1770122119645588

ENSMUST00000015456 483 Gadd45b 0.31745465075484663

ENSMUST00000021328 447 Lyzl6 0.26182343902257366

ENSMUST00000021892 996 Cts7 0.19920328789844274

ENSMUST00000018691 1251 Pip4k2b 0.35521531181262456

ENSMUST00000018644 999 Adora2b 0.3040702667865431

ENSMUST00000020507 624 Fgf18 0.34127350239585885

ENSMUST00000020969 1344 Cmpk2 0.28226296208226337

ENSMUST00000020334 2946 Usp15 0.19633323009010425

ENSMUST00000021173 1350 Mfsd11 0.24217752454700212

ENSMUST00000021311 2505 Kif18b 0.3053753692918871

ENSMUST00000017911 681 Spata25 0.29488210145449834

ENSMUST00000014174 1176 Pax5 0.27042346298031783

ENSMUST00000021423 1101 Noxred1 0.21682604225388216

ENSMUST00000020329 3633 Egfr 0.2540980827448716

ENSMUST00000020493 1290 Pofut2 0.3112829282904136

ENSMUST00000021940 1077 Lman2 0.3235217361469503

ENSMUST00000019333 1992 Rnf145 0.2594881184225654

ENSMUST00000021834 1128 Serpinb1c 0.23915053751668594

ENSMUST00000016672 1161 Mapkapk2 0.2921723480752252

ENSMUST00000021381 2181 Pnn 0.2007914877397038

ENSMUST00000021359 1584 Pole2 0.18718393092334268

ENSMUST00000020496 2106 Adarb1 0.3026524981765042

ENSMUST00000021426 1419 Vipas39 0.24092000833629004

ENSMUST00000021071 1323 Gprc5c 0.33493359566764225

ENSMUST00000020112 4374 Uhrf1bp1l 0.18959772445236064

ENSMUST00000014248 435 Sval2 0.14676651665886803

ENSMUST00000021356 2445 Dnaaf2 0.255768695465494

ENSMUST00000020524 2571 Rhbdf1 0.3410670402465716

ENSMUST00000019302 567 Tmem160 0.28302108722844

ENSMUST00000017126 2919 Pom121l2 0.2195577983293454

ENSMUST00000020662 1422 Kremen1 0.300443036465243

ENSMUST00000017270 1359 Krt42 0.3827369508725906

ENSMUST00000021338 435 Ap4s1 0.18872309716672073

ENSMUST00000018569 2340 Dhx40 0.15222517478959127

ENSMUST00000019101 2718 Scml2 0.16742308362396421

ENSMUST00000019447 654 Psmc3ip 0.30728379864201166

ENSMUST00000021685 2376 Hhipl1 0.33192240464021056

ENSMUST00000021302 297 Higd1b 0.32321122758540294

ENSMUST00000020655 2490 Jade2 0.2974451567472515

ENSMUST00000021667 465 Isca2 0.22493515288334207

ENSMUST00000019614 2568 Xab2 0.32819049384488885

ENSMUST00000021271 3876 Per1 0.29222593863181057

ENSMUST00000019469 1074 G6pc 0.2635914492135677

ENSMUST00000020027 1362 Serinc1 0.1855347596355154

ENSMUST00000020908 819 E2f6 0.20136230706928715

ENSMUST00000020554 1218 Madcam1 0.3121769597479888

ENSMUST00000021018 1674 Taf15 0.16901488738532972

ENSMUST00000021719 177 2010107E04Rik 0.1713499741960311

ENSMUST00000016399 1356 Tubb1 0.31296211285321773

ENSMUST00000019901 2151 Ccdc170 0.24871234717070528

ENSMUST00000020576 885 Ccng1 0.16675482965971358

ENSMUST00000020884 1314 Atp6v1c2 0.2716630857091211

ENSMUST00000021135 1848 1200014J11Rik 0.21808508248505426

ENSMUST00000021375 2298 Sec23a 0.1796374455324871

ENSMUST00000021459 639 Rab15 0.31970955056631845

ENSMUST00000021558 1677 Galnt16 0.3015207453064318

ENSMUST00000019382 927 Tecr 0.3545801767930358

ENSMUST00000021239 924 Lrrc59 0.3308462079147886

ENSMUST00000016664 2064 Lnx2 0.3160865251191061

ENSMUST00000020821 1461 Tubd1 0.19802782938617255

ENSMUST00000021820 1122 Aspn 0.1497249931442304

ENSMUST00000019937 2283 Sec63 0.16758620408222055

ENSMUST00000018737 909 Sparc 0.40809896074960395

ENSMUST00000019323 1017 Mdh2 0.3036779618251651

ENSMUST00000020289 2580 Pald1 0.3606254453786511

ENSMUST00000016072 4395 Rrbp1 0.30875545735214394

ENSMUST00000021114 1179 Galk1 0.29290336540691225

ENSMUST00000019954 519 Zc2hc1b 0.19420354499194986

ENSMUST00000021539 1323 Vrk1 0.20446386501526717

ENSMUST00000021513 771 Gsc 0.3566802914748396

ENSMUST00000021547 7590 Zfyve26 0.27327390436859983

ENSMUST00000021776 735 Prl7d1 0.17202467506213992

ENSMUST00000017193 672 Ccdc70 0.28012125515801034

ENSMUST00000018992 1983 Rars 0.22641412203089725

ENSMUST00000014065 1644 Clip3 0.27700819744525845

ENSMUST00000015998 1059 Cd5l 0.25152399407390535

ENSMUST00000014750 945 Slc25a11 0.27986807669796426

ENSMUST00000020227 1821 Cry1 0.23063937796242667

ENSMUST00000021379 810 Gemin2 0.1550317489315334

ENSMUST00000019608 1218 Ptger1 0.2653418388659838

ENSMUST00000019938 1158 Nr2e1 0.22586653393837197

ENSMUST00000020362 576 Kcnmb1 0.310539369843906

ENSMUST00000016383 2262 Lonrf3 0.21375825987168223

ENSMUST00000018212 3597 Ints2 0.17875394395774205

ENSMUST00000021592 1317 Cdca7l 0.25353849428352254

ENSMUST00000021282 4014 Pfas 0.2695759011660058

ENSMUST00000020107 3663 Atp2b1 0.18007606180271019

ENSMUST00000021913 945 Auh 0.19947549619324462

ENSMUST00000019283 1674 Isyna1 0.33353593722854685

ENSMUST00000021536 744 Atp6v1d 0.21364939072579187

ENSMUST00000014990 531 Tppp3 0.3474527300696611

ENSMUST00000020896 615 Tspan13 0.24803354658330315

ENSMUST00000014438 300 Ndufa2 0.2795493406525323

ENSMUST00000016696 1998 Foxred2 0.33973461130097193

ENSMUST00000018012 1104 Sgk2 0.30200398513707916

ENSMUST00000021029 1299 Efcab3 0.1889030654970442

ENSMUST00000021242 387 Tac4 0.2406762774322209

ENSMUST00000021958 411 Pcbd2 0.22488216269305608

ENSMUST00000015160 1752 Acsf3 0.2918834551119062

ENSMUST00000015981 1215 Sqstm1 0.30682948018165

ENSMUST00000020704 819 Igfbp1 0.2848966418634334

ENSMUST00000020391 804 Rab36 0.32179024632081366

ENSMUST00000021948 1794 F12 0.30926627210294066

ENSMUST00000017836 1215 Rhbdl3 0.3568571981953226

ENSMUST00000021209 1239 Doc2b 0.37416999159055914

ENSMUST00000017567 1395 Zfp207 0.174765199929626

ENSMUST00000019631 1644 Trip10 0.3136672743937457

ENSMUST00000019808 1392 Plin5 0.3547122248576376

ENSMUST00000021251 972 Lrrc46 0.3006952878912369

ENSMUST00000014683 801 Klra5 0.17470074427852753

ENSMUST00000021020 1533 Mmp28 0.3068411580709042

ENSMUST00000021412 741 Psma6 0.18834321755811007

ENSMUST00000021796 609 Edn1 0.24908726771864678

ENSMUST00000019076 1044 Map2k3 0.3635971339897014

ENSMUST00000020408 1470 Mdm2 0.22464902862656186

ENSMUST00000020831 1473 Fam114a2 0.20022481910122555

ENSMUST00000014022 861 Rnf170 0.15427793228023037

ENSMUST00000019577 1002 Gipc1 0.34664411990184096

ENSMUST00000018803 786 Pnpo 0.3272855407668049

ENSMUST00000021772 1305 Mrs2 0.24546717036579668

ENSMUST00000020962 777 Ubxn2a 0.16352956195835572

ENSMUST00000015278 1539 Aldh1a3 0.3029687371816915

ENSMUST00000021203 585 Timm22 0.2651834486500785

ENSMUST00000021450 1293 Sgpp1 0.25138085852941044

ENSMUST00000019803 501 Ccdc12 0.3365725866299587

ENSMUST00000014812 1992 Chfr 0.23330939165067915

ENSMUST00000016144 855 Ppap2a 0.2004660885717462

ENSMUST00000020911 936 Sdc1 0.28282165254622027

ENSMUST00000019246 1362 Aldh3a1 0.31195686199291656

ENSMUST00000021890 1005 Cts6 0.20915182312608313

ENSMUST00000018383 537 Zcchc10 0.20027441934498885

ENSMUST00000015987 1392 Rxrg 0.2948075930449845

ENSMUST00000021296 774 Tmem101 0.3062077848715767

ENSMUST00000019506 285 D8Ertd738e 0.2924649634541054

ENSMUST00000021278 3636 Ctc1 0.23182452173181736

ENSMUST00000014562 3381 Hps5 0.19694541636339324

ENSMUST00000020271 459 Srgn 0.22021455386618627

ENSMUST00000020949 1005 Map2k6 0.22335786929933973

ENSMUST00000020624 1659 Cnot6 0.1636902674317028

ENSMUST00000021049 1221 Psmc5 0.27369913996547907

ENSMUST00000018274 1248 Csnk1d 0.27868947298696006

ENSMUST00000020171 1047 Ctgf 0.3891481819266922

ENSMUST00000020553 501 Chac2 0.16264568258991807

ENSMUST00000021968 948 Pitx1 0.35803299926552484

ENSMUST00000016034 1281 Amdhd1 0.2800299850458605

ENSMUST00000017900 3252 Slc12a7 0.3222356483597943

ENSMUST00000021750 14901 Ryr2 0.24911329589277217

ENSMUST00000019833 342 1110001J03Rik 0.22513515316790864

ENSMUST00000020657 459 Ube2b 0.16588049743856506

ENSMUST00000021635 972 Akr1c18 0.21742907630637515

ENSMUST00000018842 1221 Lhx1 0.27715314392644685

ENSMUST00000017610 663 Timp2 0.33406550216280495

ENSMUST00000021532 1170 Snapc1 0.2136478833791737

ENSMUST00000021689 1245 Evl 0.290254451556083

ENSMUST00000020169 2625 Enpp3 0.20111100131921475

ENSMUST00000015391 744 Nipsnap3b 0.20090880710505138

ENSMUST00000019069 1710 Heatr9 0.23545336473812303

ENSMUST00000014957 744 Stc1 0.27745681650685317

ENSMUST00000017290 5439 Brca1 0.1879317966799934

ENSMUST00000014922 3594 Fhod1 0.28847233739116684

ENSMUST00000020251 3708 Gnptab 0.22790982799832302

ENSMUST00000018632 5820 Myh4 0.35978492932176764

ENSMUST00000021810 486 Id4 0.30377918081033506

ENSMUST00000015855 1365 Prune 0.2762437268873168

ENSMUST00000020500 1989 Appl2 0.2318206736018768

ENSMUST00000021649 1362 Acot2 0.3199381858107556

ENSMUST00000017974 2037 Dhx58 0.29303981336081303

ENSMUST00000020215 597 Socs2 0.19140614053880925

ENSMUST00000015894 744 Aph1a 0.23093948082854499

ENSMUST00000020683 846 Hus1 0.2098880539662516

ENSMUST00000018716 1206 Phf23 0.243817339103776

ENSMUST00000015596 1209 Ager 0.26359152840285327

ENSMUST00000018002 1281 Ift52 0.2416692191434005

ENSMUST00000016105 1371 Adss 0.14568375006755432

ENSMUST00000020719 783 2310033P09Rik 0.31854099327153146

ENSMUST00000015877 861 Capza2 0.15514541248259697

ENSMUST00000015501 1905 Clpx 0.16380667882971112

ENSMUST00000019130 1554 Slfn3 0.17823627933725533

ENSMUST00000020284 1707 Tysnd1 0.30745751001836674

ENSMUST00000021085 1971 Nup85 0.2629742331155718

ENSMUST00000019616 2754 Icam5 0.26770701452723095

ENSMUST00000020926 879 Fam84a 0.3739209386741779

ENSMUST00000015901 1479 Ppil4 0.17433937722189569

ENSMUST00000016315 3507 Lamb3 0.3046796579114103

ENSMUST00000018407 1557 Tbx5 0.31457059316453334

ENSMUST00000021339 507 Dtd2 0.2529918325677982

ENSMUST00000017831 1992 Rhot1 0.17529019384578728

ENSMUST00000021183 1314 Eral1 0.25321181579544916

ENSMUST00000017408 2664 Exosc10 0.2768916755595889

ENSMUST00000020522 2343 Pfkl 0.3501692388310846

ENSMUST00000020579 2385 Hmmr 0.1729543435350796

ENSMUST00000021120 1929 Trim47 0.335456826522372

ENSMUST00000021791 1515 Gcm2 0.28327853047222773

ENSMUST00000020939 3771 Nrcam 0.1978679389510787

ENSMUST00000014597 1500 Blk 0.32729851007725236

ENSMUST00000020003 3432 Fam184a 0.216635623910039

ENSMUST00000021406 906 2700097O09Rik 0.17111822616380792

ENSMUST00000017597 1173 Pipox 0.23813645610765954

ENSMUST00000020081 759 Zwint 0.26414689455323304

ENSMUST00000019445 1035 Hsd17b1 0.25414037748086105

ENSMUST00000019679 1407 Armc6 0.2970969561404883

ENSMUST00000018971 1962 Brsk2 0.32368900761745834

ENSMUST00000021729 1149 Gpr132 0.31764510937124596

ENSMUST00000017692 2226 Suz12 0.15683215110395454

ENSMUST00000017534 1092 Aldoc 0.27119775809732005

ENSMUST00000019945 1119 Pex3 0.17349543933260145

ENSMUST00000021133 1878 Srp68 0.29930949622884867

ENSMUST00000016902 453 Bik 0.3198220092369928

ENSMUST00000020954 2394 Trappc12 0.28279800272250244

ENSMUST00000021195 1893 Slc6a4 0.2944802952018508

ENSMUST00000019967 3147 Mical1 0.29344992113964585

ENSMUST00000016631 2910 Ppfibp1 0.2247216016075435

ENSMUST00000021778 669 Prl2c5 0.17779555346867662

ENSMUST00000021288 3399 Usp43 0.2718710562418583

ENSMUST00000020043 2445 Lrriq1 0.1620461280662831

ENSMUST00000015368 1515 Cyp2j11 0.21021725610845365

ENSMUST00000020249 717 Dram1 0.24503045439054233

ENSMUST00000021399 930 Zc3h14 0.1760254134520377

ENSMUST00000021066 984 Cacng4 0.3356864391474713

ENSMUST00000021884 342 Ctla2b 0.25211316132091527

ENSMUST00000020904 4167 Rock2 0.16685003747950997

ENSMUST00000020695 4323 Tns3 0.2785375935696111

ENSMUST00000020712 507 4921536K21Rik 0.2753333640448773

ENSMUST00000020643 2139 Rufy1 0.25754333094698295

ENSMUST00000020529 996 Ahsa2 0.2230130299561767

ENSMUST00000017255 1539 Krt24 0.27431069457856655

ENSMUST00000019064 741 Cxcl16 0.22701691830208537

ENSMUST00000017354 2964 Med24 0.3359299971689883

ENSMUST00000020349 6825 Apc2 0.3056155441350684

ENSMUST00000018476 1494 Stk3 0.17473963567900283

ENSMUST00000014546 1176 Tsg101 0.20267703708501614

ENSMUST00000019965 1260 Smpd2 0.29472854336554577

ENSMUST00000020109 1191 Actr6 0.17643031382202554

ENSMUST00000021932 1341 Drd1 0.2647691195566071

ENSMUST00000018516 2484 Cep95 0.18476842904817747

ENSMUST00000020204 1887 Ntn4 0.2824967908900951

ENSMUST00000016670 1761 Dyrk3 0.25594061237660953

ENSMUST00000018399 1215 Krt33a 0.4529581487384327

ENSMUST00000018905 744 Mpdu1 0.26214167137301836

ENSMUST00000015816 564 Mrpl32 0.19117501444531695

ENSMUST00000019625 5814 Myh8 0.3567127737669195

ENSMUST00000021665 1086 Vsx2 0.30833356784895155

ENSMUST00000020826 549 Sap30l 0.3692922255366577

ENSMUST00000020301 930 4632428N05Rik 0.30201979100224036

ENSMUST00000021870 1242 Slc35b3 0.2173218917800133

ENSMUST00000019997 2328 Tnfaip3 0.25231259086520225

ENSMUST00000021384 1554 Mia2 0.16121860364244292

ENSMUST00000021268 2136 Aloxe3 0.3157241280666863

ENSMUST00000014642 3231 Ankrd52 0.2793418547168285

ENSMUST00000019791 3096 Sema6a 0.28133116438737926

ENSMUST00000025853 618 Drap1 0.3097936063137548

ENSMUST00000027579 1257 Actr3 0.17823034588165818

ENSMUST00000027337 4521 Fam135a 0.1412889018517907

ENSMUST00000026474 3336 Gli1 0.2737253496266375

ENSMUST00000026973 1146 Prkar1b 0.3086382226084002

ENSMUST00000029268 390 1810062G17Rik 0.2117200791466082

ENSMUST00000029208 1803 Slc13a3 0.3588013436435616

ENSMUST00000027507 867 Pdcd1 0.2951550602181904

ENSMUST00000024839 2340 Sik1 0.31116062234864605

ENSMUST00000028118 2124 Prkcq 0.2615777483871091

ENSMUST00000023958 2199 P3h3 0.2953864846055711

ENSMUST00000025786 2886 Pacs1 0.27665192373724673

ENSMUST00000025236 675 Stard4 0.186346984067178

ENSMUST00000028593 681 Prrg4 0.176900872112098

ENSMUST00000027690 1266 Avpr1b 0.33809292516356004

ENSMUST00000025506 1263 Rbm22 0.27245552466800965

ENSMUST00000026550 1269 Inpp5a 0.288943250837712

ENSMUST00000025705 3399 Jak2 0.1813469668404246

ENSMUST00000025636 873 Ms4a8a 0.24447230563995498

ENSMUST00000026520 1203 Paip1 0.1622011788887775

ENSMUST00000022051 1386 Nkd2 0.30412576375625433

ENSMUST00000024783 1311 Bysl 0.3338645806554286

ENSMUST00000024742 1095 Nfkbie 0.26324530561495685

ENSMUST00000024721 1317 Rhag 0.18162013255352125

ENSMUST00000024826 390 Tff2 0.3029856474940254

ENSMUST00000026577 2190 Eps8l2 0.34545049437948483

ENSMUST00000029877 1008 Decr1 0.18841506271897102

ENSMUST00000028584 597 Commd9 0.27589845495013204

ENSMUST00000025192 753 H2-Oa 0.3410859838384249

ENSMUST00000028360 471 Il1f5 0.2905695178663106

ENSMUST00000027286 225 Coa5 0.2666292841475461

ENSMUST00000025993 4596 Slit1 0.34971862182340613

ENSMUST00000028105 1335 Fam188a 0.15242912761172475

ENSMUST00000028898 396 1700020A23Rik 0.26726495700105024

ENSMUST00000025864 501 Rnaseh2c 0.25274644841614075

ENSMUST00000025058 3570 Anks1 0.3278582502664382

ENSMUST00000025749 1458 Rps6kb2 0.2914039055136464

ENSMUST00000022646 714 Nkx3-1 0.2847106936617005

ENSMUST00000022834 783 Cma1 0.2449846751568717

ENSMUST00000025053 1644 Mllt1 0.38708194734991974

ENSMUST00000027826 453 Dusp23 0.3190099598356293

ENSMUST00000025617 468 Rfk 0.21576999439125513

ENSMUST00000022163 615 Btf3 0.24249085955320043

ENSMUST00000028137 801 Coq4 0.3126956864208417

ENSMUST00000027237 1845 Il18rap 0.20858806251320747

ENSMUST00000023083 1503 Cyp2d22 0.31137749945915516

ENSMUST00000022587 432 Tsc22d1 0.24260322578527577

ENSMUST00000027682 1446 Gpr37l1 0.34171018839694817

ENSMUST00000027921 3039 Iars2 0.22485534006959496

ENSMUST00000027429 861 2810459M11Rik 0.2798520933339555

ENSMUST00000029521 309 Crct1 0.3200477668931692

ENSMUST00000026318 516 Sat1 0.22515508914488216

ENSMUST00000023994 1515 Serping1 0.29258716727929057

ENSMUST00000023805 1482 Csad 0.35281662098178335

ENSMUST00000026710 2478 Usp16 0.17280716975260413

ENSMUST00000029850 996 Cryz 0.20858488731595748

ENSMUST00000025477 1143 St8sia3 0.18847539202184838

ENSMUST00000028689 5718 Lrp4 0.3087154080070454

ENSMUST00000027554 867 Cln8 0.3210844984688876

ENSMUST00000028355 1374 Pax8 0.2931499906080758

ENSMUST00000027269 1131 Mstn 0.1955702579438826

ENSMUST00000025511 456 Rps14 0.27890511382577343

ENSMUST00000028972 402 Pdrg1 0.2627397598379856

ENSMUST00000023265 372 Psca 0.351311461545977

ENSMUST00000028300 1761 Nacc2 0.3337038149979456

ENSMUST00000025064 3717 Pdzph1 0.18544902442382785

ENSMUST00000029567 1554 Fam198b 0.2355847937121662

ENSMUST00000022344 1926 Ecd 0.22385346699654016

ENSMUST00000028408 1251 Hat1 0.1977769977993999

ENSMUST00000025729 3501 Tnks2 0.16177988412480931

ENSMUST00000029386 1851 Etfdh 0.17113142773785175

ENSMUST00000028209 717 Dolpp1 0.2800504052971889

ENSMUST00000029311 2925 Trpc4 0.22782901569207456

ENSMUST00000029508 1401 Dennd2d 0.23100944531932993

ENSMUST00000028597 1530 Tcp11l1 0.24174977304689502

ENSMUST00000023147 3237 Ciita 0.3033412317466042

ENSMUST00000029422 996 Shox2 0.2587786867343699

ENSMUST00000023219 1608 Fbxl6 0.2724828795564186

ENSMUST00000028761 1620 Ubox5 0.2593418015248283

ENSMUST00000025718 960 Ankrd1 0.2263378312489858

ENSMUST00000022856 843 Rad1 0.19605736761317477

ENSMUST00000025406 1326 Srfbp1 0.22226100924936268

ENSMUST00000029123 396 a 0.34908321508655904

ENSMUST00000022616 1347 Clu 0.34950315313027946

ENSMUST00000023814 345 Npff 0.3239847361627005

ENSMUST00000025713 1257 Tm7sf2 0.29002588632072

ENSMUST00000029911 2445 Pnisr 0.20359975238025468

ENSMUST00000022115 981 Xrcc4 0.19430085458977087

ENSMUST00000029419 2502 Veph1 0.22855177185371614

ENSMUST00000026560 1131 Psmd13 0.2576759381899419

ENSMUST00000022629 1719 Dpysl2 0.31359158701677503

ENSMUST00000025641 1872 Zp1 0.27260114388375006

ENSMUST00000022450 4497 Fam208a 0.14178297605148638

ENSMUST00000024884 1548 Eif2ak2 0.15851971545759694

ENSMUST00000027533 1746 Klhl30 0.33086006673651414

ENSMUST00000024761 2106 Fbxl17 0.2805777668712497

ENSMUST00000025229 2292 Cfb 0.2720317490235517

ENSMUST00000029643 696 Gar1 0.21016200543302

ENSMUST00000024049 1179 Bmp15 0.19749813519164547

ENSMUST00000022980 540 Ndufb9 0.31731850174651993

ENSMUST00000025762 270 Banf1 0.324316848628213

ENSMUST00000029625 888 Sfrp2 0.4324451459154948

ENSMUST00000022916 2577 Lrp12 0.21525527514117376

ENSMUST00000025377 1341 Ppp2r2b 0.2535454535400225

ENSMUST00000022573 849 Esd 0.21332862530366295

ENSMUST00000022696 3264 Xpo7 0.2293992394238389

ENSMUST00000027440 1218 Nmur1 0.3294258517414192

ENSMUST00000027602 1506 Dars 0.1673995218313096

ENSMUST00000028987 1425 Bpifb1 0.3431802838317588

ENSMUST00000024956 618 Rhoq 0.19698187076674628

ENSMUST00000027434 2454 Armc9 0.2751302954621543

ENSMUST00000023617 1614 Ildr1 0.29560002576898486

ENSMUST00000029909 1113 Coq3 0.19608008009506137

ENSMUST00000026659 2433 Tmc6 0.3189586690307988

ENSMUST00000022528 999 Pinx1 0.23903562974845158

ENSMUST00000026128 255 Anapc11 0.393911190655864

ENSMUST00000023670 1791 Clic6 0.2546649646231074

ENSMUST00000027064 438 Tmem14a 0.17467698104484308

ENSMUST00000026297 1734 Gnl3l 0.24011752632913777

ENSMUST00000028475 1278 Clp1 0.23417069656443695

ENSMUST00000029046 408 Fabp5 0.3079360703965839

ENSMUST00000022767 1743 Mettl3 0.21041071752837848

ENSMUST00000022518 675 N6amt2 0.25587092402563993

ENSMUST00000025497 8724 Fbn2 0.3025521367223302

ENSMUST00000022986 1068 Fbxo32 0.2853092698607038

ENSMUST00000028252 1617 Grb14 0.23047123487251897

ENSMUST00000025739 2412 Uhrf2 0.18932234155834304

ENSMUST00000029451 666 Tspan2 0.2100356905256212

ENSMUST00000023150 828 1810013L24Rik 0.23619422698139467

ENSMUST00000028834 912 Tmco5 0.20436739636488352

ENSMUST00000026045 4413 Col17a1 0.25492730079669995

ENSMUST00000023509 1803 Klhl24 0.19022812896594624

ENSMUST00000027830 858 Slamf9 0.24983424796967893

ENSMUST00000023061 609 Josd1 0.25575675390207164

ENSMUST00000029674 621 Efna4 0.2810538360961795

ENSMUST00000028024 597 Tnfsf4 0.2203887757987009

ENSMUST00000027137 1713 Slc40a1 0.21482358828799586

ENSMUST00000029149 1575 Rbm39 0.14779749707471218

ENSMUST00000025004 2070 Adgre4 0.18659782324044422

ENSMUST00000022226 1941 Ppwd1 0.17352179525504455

ENSMUST00000024756 1434 Enpp5 0.17927496123072809

ENSMUST00000025570 495 Sdhaf2 0.25743135113854915

ENSMUST00000026539 462 Fuom 0.26505155822919746

ENSMUST00000024930 1311 1600002H07Rik 0.24077864398306492

ENSMUST00000026381 2001 Padi4 0.3655031333706922

ENSMUST00000025823 990 Rce1 0.28562930595181457

ENSMUST00000022757 747 Gzmf 0.258190133859802

ENSMUST00000022954 1041 Khdrbs3 0.2252725304570798

ENSMUST00000025856 4845 Lrp5 0.35483155818158657

ENSMUST00000028314 945 Nmi 0.21331114444311425

ENSMUST00000026188 2328 R3hcc1l 0.1688688416378097

ENSMUST00000022150 390 Cartpt 0.3208566736549788

ENSMUST00000022218 564 Dhfr 0.18609096491356722

ENSMUST00000023370 3333 Boc 0.27463719259199987

ENSMUST00000022330 984 Ldb3 0.32552826387498374

ENSMUST00000028129 1434 Slc2a8 0.34201020073765737

ENSMUST00000023686 477 Tmem50b 0.22968089038570194

ENSMUST00000026062 912 Casp7 0.31934027070869664

ENSMUST00000024118 630 Clec4n 0.22394124809954655

ENSMUST00000029331 1122 P2ry1 0.2541129662079926

ENSMUST00000025484 1269 Fech 0.2599367507916216

ENSMUST00000023494 1116 Popdc2 0.3059313297903215

ENSMUST00000028841 3243 Usp8 0.2043658082374994

ENSMUST00000029666 1875 Papss1 0.27067149673141117

ENSMUST00000026661 702 Tk1 0.36336299862467547

ENSMUST00000023212 777 Maf1 0.3007143326089357

ENSMUST00000028238 648 Rab14 0.16599079920808596

ENSMUST00000027931 1332 Nek2 0.2812464833673086

ENSMUST00000025931 1410 Pdcd4 0.16707062579871507

ENSMUST00000029795 1551 Rorc 0.27921416328809073

ENSMUST00000025295 903 Spry4 0.3513104030596486

ENSMUST00000022097 351 Ndufs6 0.24538570576097413

ENSMUST00000023104 1983 Rpap3 0.2124976238415873

ENSMUST00000028970 1842 Mylk2 0.3250272247349496

ENSMUST00000024963 438 Mcfd2 0.3160372114729596

ENSMUST00000024984 681 Tmem204 0.30525037689683204

ENSMUST00000022925 1059 Eif3h 0.25721407424795656

ENSMUST00000025212 1818 Slc23a1 0.28761585660415534

ENSMUST00000029866 1212 Ccne2 0.15212851138181588

ENSMUST00000027303 876 Imp4 0.2927131547858419

ENSMUST00000023489 954 Fyttd1 0.1492258481075329

ENSMUST00000023758 1581 Asic1 0.3547629120690861

ENSMUST00000024104 1311 Gcm1 0.27695938995270014

ENSMUST00000026036 1419 Nr0b1 0.33443230012656383

ENSMUST00000023555 1452 Hspbap1 0.23138094451021315

ENSMUST00000027876 2208 Scyl3 0.21017800398283612

ENSMUST00000022272 714 Kctd6 0.2477394389433137

ENSMUST00000022688 1470 Slc39a14 0.3227765207764266

ENSMUST00000029034 399 Pmp2 0.27519491875246077

ENSMUST00000022650 2271 Pibf1 0.16580776966341443

ENSMUST00000023361 2370 Pdxdc1 0.1938061703414839

ENSMUST00000029043 399 Fabp12 0.2678413463231695

ENSMUST00000028672 423 Mdk 0.3656488922777109

ENSMUST00000025968 1473 Cyp2c39 0.19795950622944436

ENSMUST00000023220 1353 Slc52a2 0.2800424912795183

ENSMUST00000026743 1443 Uqcrc1 0.2957995609516413

ENSMUST00000023477 2100 Dnm1l 0.15873065245052942

ENSMUST00000025314 1533 0610009O20Rik 0.27584525184903685

ENSMUST00000029405 2082 Gmps 0.15455179477452163

ENSMUST00000027886 1695 Ralgps2 0.17408217514530175

ENSMUST00000026519 1920 4833420G17Rik 0.17595689570609716

ENSMUST00000026096 2973 Bnc1 0.27830421943112066

ENSMUST00000025806 1164 Doc2g 0.31531290865724

ENSMUST00000025413 2898 Sncaip 0.22553983874244207

ENSMUST00000026541 681 Caly 0.34021560357241853

ENSMUST00000027394 774 Zfand2b 0.2554929147022018

ENSMUST00000023846 1677 Lrrc71 0.29113570697663005

ENSMUST00000023222 3867 Oplah 0.27614725740286084

ENSMUST00000024999 936 Tpsg1 0.2960079910844475

ENSMUST00000022962 894 Emc2 0.17624358339626597

ENSMUST00000028167 2184 Gpd2 0.22557591604716934

ENSMUST00000023737 1191 Dhh 0.25172784698584344

ENSMUST00000023217 2199 Bop1 0.31782697634849044

ENSMUST00000028977 2244 Kif3b 0.2552150789630396

ENSMUST00000029761 795 Myoz2 0.21174067324908938

ENSMUST00000025580 735 Ms4a6b 0.21034736155175152

ENSMUST00000025246 648 Csnk2b 0.32342749086593575

ENSMUST00000023070 864 Upk3a 0.33780543564386556

ENSMUST00000025322 996 H2-M10.1 0.2366975446508186

ENSMUST00000026912 1146 Ccdc7b 0.13152045867859585

ENSMUST00000027491 1245 Agxt 0.32032421738001143

ENSMUST00000022960 1338 Eif3e 0.17632858957264247

ENSMUST00000026859 1560 Mfsd8 0.16281526577834007

ENSMUST00000023359 1035 Nde1 0.2417282421153181

ENSMUST00000025562 2631 Incenp 0.28769370651923115

ENSMUST00000029183 1758 Fam83d 0.2889524749212245

ENSMUST00000027380 894 Tmem169 0.3374825068257921

ENSMUST00000027726 918 Cyb5r1 0.2698311169535431

ENSMUST00000026538 873 Echs1 0.24235212419848862

ENSMUST00000024802 501 Ppil1 0.31631950664109476

ENSMUST00000028759 2667 Ltk 0.2909407158904137

ENSMUST00000027634 264 Dbi 0.28749010786783075

ENSMUST00000028554 1575 Lpcat4 0.27081269736474034

ENSMUST00000029369 1404 Sohlh2 0.22946996875758086

ENSMUST00000027988 822 Ccdc3 0.34737674694496085

ENSMUST00000025663 4152 Tmem2 0.24019733606573382

ENSMUST00000029677 1635 Zbtb7b 0.3097048611613538

ENSMUST00000026495 1662 Atp5a1 0.2400585616900612

ENSMUST00000026818 540 Sec11a 0.17817603918421387

ENSMUST00000022597 2595 Naa16 0.16787544718839917

ENSMUST00000026507 2694 Usp33 0.1965650326374975

ENSMUST00000025844 1116 Ctsw 0.31570193631987026

ENSMUST00000023525 1323 Gtf2e1 0.21868166741486217

ENSMUST00000024786 1605 Tfeb 0.3774320332942156

ENSMUST00000025755 1125 Dmrt1 0.32721834043772896

ENSMUST00000025106 429 Polr2d 0.2628082434102509

ENSMUST00000026823 1746 Pigq 0.30942655397631735

ENSMUST00000025349 1491 Myot 0.2023079296507166

ENSMUST00000022678 729 Pebp4 0.21710685037908853

ENSMUST00000026217 2238 Chuk 0.16263492166797677

ENSMUST00000029641 1488 Asic5 0.19854752705649084

ENSMUST00000023276 2883 Trappc9 0.3038882470771312

ENSMUST00000025684 1605 Ehd1 0.4176453212090415

ENSMUST00000029822 1359 Tacr3 0.28052427382967743

ENSMUST00000028619 939 Hsd17b12 0.2203618015202177

ENSMUST00000027975 1017 Phyh 0.23416522274793883

ENSMUST00000027267 2754 Pms1 0.1550997354228038

ENSMUST00000026122 1530 P4hb 0.29612773496605704

ENSMUST00000023068 3747 Smc1b 0.1535072402737087

ENSMUST00000023048 1392 Mief1 0.26706798495169415

ENSMUST00000029771 885 F3 0.19937671320860803

ENSMUST00000027768 6732 Ahctf1 0.14732249279422205

ENSMUST00000024749 2085 Polh 0.20803954191228488

ENSMUST00000023179 2058 Zfp7 0.27633103174900386

ENSMUST00000025598 888 Keg1 0.22085322849160094

ENSMUST00000029444 3423 Trim33 0.1744162480877885

ENSMUST00000025305 765 Mrps18b 0.2317310477437058

ENSMUST00000029438 3633 Hipk1 0.2263725552886226

ENSMUST00000026470 1515 Shmt2 0.31067333200644837

ENSMUST00000023044 1488 Fam83f 0.32798924616593794

ENSMUST00000025254 1026 Lims2 0.39876279648271107

ENSMUST00000027071 867 Lactb2 0.18477950233473228

ENSMUST00000024810 1968 Fgd2 0.3666060828526019

ENSMUST00000024470 1050 Ogfod2 0.3555119030220941

ENSMUST00000022813 1683 Efs 0.27415375954481286

ENSMUST00000028995 573 Fam210b 0.273575432281603

ENSMUST00000026416 1041 Cdk2 0.2501090458669424

ENSMUST00000028636 1344 Galk2 0.234404046790544

ENSMUST00000026997 2184 Uimc1 0.20749256773664657

ENSMUST00000025393 1656 Smad4 0.19508851840919517

ENSMUST00000025761 816 Cabp4 0.29348287349160246

ENSMUST00000029573 1014 Lrrc39 0.19450910302060542

ENSMUST00000024005 639 Scg5 0.23680310337249283

ENSMUST00000026988 732 Arl10 0.3288294515009287

ENSMUST00000028223 1398 Kynu 0.1747547218883103

ENSMUST00000025645 3057 Tmem132a 0.3061984554331569

ENSMUST00000022189 2136 Aggf1 0.22886236988099834

ENSMUST00000029416 1599 Ccnl1 0.1964875749875349

ENSMUST00000022082 324 Glrx 0.24789297710152228

ENSMUST00000027476 366 3110079O15Rik 0.31669348940933223

ENSMUST00000022172 2559 Polk 0.1746623921871256

ENSMUST00000028304 564 Lcn3 0.2685830825720894

ENSMUST00000025706 1236 Tnfrsf25 0.3040525217044447

ENSMUST00000026735 1221 Ccdc51 0.2525471798890929

ENSMUST00000026499 726 Crisp3 0.14382324076356529

ENSMUST00000029060 3144 Atp9a 0.3445149570016716

ENSMUST00000025751 2982 Ighmbp2 0.3171734605537511

ENSMUST00000028162 1155 Ptges2 0.3506385278318225

ENSMUST00000026891 876 Exosc7 0.26308776150983565

ENSMUST00000024725 2562 Efhb 0.17028907024651088

ENSMUST00000022913 1413 Dcstamp 0.21538717832445947

ENSMUST00000029078 783 Car2 0.2454304726464691

ENSMUST00000025356 528 Mal2 0.223973664637242

ENSMUST00000026144 735 Dcxr 0.324499215119883

ENSMUST00000027792 261 Srp9 0.2152375139842725

ENSMUST00000025646 1737 Slc15a3 0.31961256432014096

ENSMUST00000022078 1836 Rhobtb3 0.2220430431337651

ENSMUST00000025025 1104 Dusp1 0.3594391785818889

ENSMUST00000025760 1362 Chka 0.21459686176376194

ENSMUST00000023113 615 Polr3h 0.3260512490118103

ENSMUST00000023407 1398 Mina 0.22470830213499374

ENSMUST00000028951 1587 Snph 0.3779383687782128

ENSMUST00000025137 1974 Thoc1 0.15373714285548642

ENSMUST00000029170 3192 Rbl1 0.17039685553428363

ENSMUST00000027754 1578 Ncf2 0.2914875644796886

ENSMUST00000024171 3057 Stk31 0.1498537936916961

ENSMUST00000027425 810 Itm2c 0.3360184466222275

ENSMUST00000027438 2124 Ncl 0.1807608062403682

ENSMUST00000023112 789 Pmm1 0.3502796672827389

ENSMUST00000029131 1989 Ggt7 0.2981315862556869

ENSMUST00000029433 2409 Ptpn22 0.19615080995662734

ENSMUST00000027518 612 Spp2 0.2667472921026483

ENSMUST00000028515 1374 Chrna1 0.2995598532703612

ENSMUST00000025833 1866 Papss2 0.2381552295568836

ENSMUST00000025075 783 Bambi 0.3001832873460675

ENSMUST00000028098 1302 Orc4 0.12037826249134351

ENSMUST00000025656 1506 Aldh1a7 0.21275044882172225

ENSMUST00000027997 546 Rgs5 0.32945709109490257

ENSMUST00000027564 1170 Serpinb13 0.2369192956804793

ENSMUST00000025427 1233 Rnmt 0.1900778430948528

ENSMUST00000026449 591 Il23a 0.23284934712560248

ENSMUST00000028858 3180 Bub1 0.15874170044882896

ENSMUST00000029504 1140 Cym 0.3292043211988458

ENSMUST00000027052 1617 Stau2 0.16400102643589384

ENSMUST00000026009 549 Arl3 0.2555972127078637

ENSMUST00000022494 621 Ebpl 0.23044647565058898

ENSMUST00000029629 579 Pla2g12a 0.3210523143432663

ENSMUST00000022721 1026 Cln5 0.20101648684621792

ENSMUST00000027860 345 Xcl1 0.21691436322056357

ENSMUST00000024570 1875 Serac1 0.21469273990983442

ENSMUST00000025546 1428 Cndp2 0.31876986193399914

ENSMUST00000022348 3294 Cfap70 0.21853426921575447

ENSMUST00000027849 621 Cd247 0.3043506128666994

ENSMUST00000027173 1272 Wdr12 0.20185411860549693

ENSMUST00000023754 882 Aqp6 0.2821545214668819

ENSMUST00000025666 1656 Slc22a19 0.1847688303766568

ENSMUST00000027991 618 Rgs4 0.2878016764102329

ENSMUST00000022119 636 Atg10 0.17933054199632692

ENSMUST00000023344 1314 Slc35a5 0.2003943054604587

ENSMUST00000029199 873 Zmat3 0.29841205255944975

ENSMUST00000022665 2187 Rhobtb2 0.32216293854743583

ENSMUST00000022064 1533 Lrrc14b 0.256189244222347

ENSMUST00000023693 1542 Ifnar2 0.20837203078528235

ENSMUST00000022705 813 Med4 0.18767082873814872

ENSMUST00000023247 405 Ly6f 0.21370411496120262

ENSMUST00000027233 2394 Slc9a4 0.25119949081196674

ENSMUST00000024697 363 Hcfc1r1 0.3192204414882678

ENSMUST00000022419 621 Ppif 0.30034279184592483

ENSMUST00000026420 348 Rps26 0.2949840080885358

ENSMUST00000023897 783 Gzma 0.16834935165909967

ENSMUST00000027302 1362 Ptpn18 0.29055008337542204

ENSMUST00000023396 729 Pmm2 0.22479475581122196

ENSMUST00000026303 1551 Apex2 0.2649139699694581

ENSMUST00000028071 975 Bmi1 0.1893154864997248

ENSMUST00000025956 2586 Pde6c 0.23617801648135892

ENSMUST00000025569 264 Tmem216 0.2968552145199852

ENSMUST00000024782 1179 Pgc 0.3548801983132172

ENSMUST00000027178 4956 Als2 0.22307496225961584

ENSMUST00000022593 5685 Akap11 0.1549255497145423

ENSMUST00000027379 2199 Xrcc5 0.20266478220924253

ENSMUST00000025957 1074 Fam45a 0.19414538072712975

ENSMUST00000028332 1521 Dpp7 0.28943604462029227

ENSMUST00000028306 579 Lcn5 0.24416605655196189

ENSMUST00000022906 2130 Fzd6 0.2100441545196247

ENSMUST00000027941 546 Atf3 0.27049383385967335

ENSMUST00000025875 1572 Slc1a1 0.2630549966618734

ENSMUST00000023283 2109 Lmf2 0.2888912002464007

ENSMUST00000026841 1428 Hadhb 0.19468424594942352

ENSMUST00000027012 1122 Casp4 0.20879112969124833

ENSMUST00000023065 1023 Dmc1 0.18782762825730204

ENSMUST00000025060 1023 Armc12 0.2822906050350596

ENSMUST00000023934 444 Hbb-bs 0.3656001257493603

ENSMUST00000028062 1401 Vim 0.3507077481760704

ENSMUST00000022831 2016 Khnyn 0.29528790585422093

ENSMUST00000028161 1800 Cel 0.32280118051425594

ENSMUST00000028005 462 Mgst3 0.2937703152801918

ENSMUST00000024233 1689 Tulp2 0.262966766543365

ENSMUST00000026243 2751 Mgea5 0.1997215831650396

ENSMUST00000027956 1434 Suv39h2 0.20264920712119489

ENSMUST00000023610 2907 Adamts1 0.2657081177758735

ENSMUST00000028256 513 Morn5 0.3773103533748662

ENSMUST00000028241 855 Stom 0.2543837119044541

ENSMUST00000022634 657 Bnip3l 0.2381101080986267

ENSMUST00000028737 1032 Slx4ip 0.180038297789944

ENSMUST00000027370 1158 Pnkd 0.3164701247770907

ENSMUST00000022327 2172 Ldb3 0.3384412495364873

ENSMUST00000029325 1197 Aadac 0.13799367975317345

ENSMUST00000028831 2502 Mcm8 0.1696264015655138

ENSMUST00000025867 1650 Rela 0.2941754489713826

ENSMUST00000025181 1110 H2-K1 0.3259905057512063

ENSMUST00000023237 1617 Naprt 0.2706538001290166

ENSMUST00000026698 1512 Podxl 0.27288602013714935

ENSMUST00000027414 3219 Stk11ip 0.27181645280834726

ENSMUST00000026480 1161 Ttc4 0.2647195742417417

ENSMUST00000022725 1554 Dct 0.25712865242197935

ENSMUST00000022881 1443 Fam134b 0.2136862859212365

ENSMUST00000029891 990 Tmem68 0.1416114702209747

ENSMUST00000028329 1278 Sapcd2 0.31135758283359727

ENSMUST00000029663 960 Aimp1 0.21939545190921836

ENSMUST00000028838 1989 Hdc 0.2905282548676163

ENSMUST00000022508 1281 Ptpn20 0.17859881243316206

ENSMUST00000028826 2034 Chgb 0.2519326695504775

ENSMUST00000028342 360 Ssna1 0.3454908051583288

ENSMUST00000027760 2307 Rgl1 0.2956502650899807

ENSMUST00000026434 603 Chmp6 0.4042791137297954

ENSMUST00000023810 414 Prr13 0.25576971180557573

ENSMUST00000025983 1140 Ccnj 0.2518978154382022

ENSMUST00000029553 3318 Ubap2l 0.21118137384082042

ENSMUST00000022197 1017 Scamp1 0.2506317332626702

ENSMUST00000024764 615 Crip3 0.2935882544361182

ENSMUST00000025057 636 Taf11 0.2786327752776533

ENSMUST00000024757 1371 Enpp4 0.1906841042333338

ENSMUST00000026156 999 Rfng 0.29932824949646686

ENSMUST00000027503 639 Dtymk 0.22665561093716724

ENSMUST00000027824 675 Apcs 0.19164179998610176

ENSMUST00000025409 1236 Lox 0.2687380625771999

ENSMUST00000023086 324 Smdt1 0.2302558338573459

ENSMUST00000024774 606 Guca1b 0.4099639916113496

ENSMUST00000026274 900 Lztfl1 0.1563896770604101

ENSMUST00000029140 729 Procr 0.33664297047390757

ENSMUST00000027626 954 Nifk 0.2019368692416809

ENSMUST00000022567 3276 Cacna2d3 0.24620417743895956

ENSMUST00000024620 1644 Riok2 0.1932152870764571

ENSMUST00000028157 1545 Gtf3c5 0.3013176628375814

ENSMUST00000028934 420 Cst11 0.26781581530663523

ENSMUST00000027979 1260 Uhmk1 0.22232238271495977

ENSMUST00000025396 1029 Rax 0.2962120250677417

ENSMUST00000028855 2508 Prom2 0.32077213464443893

ENSMUST00000028293 1149 Sdccag3 0.22130644634896918

ENSMUST00000026322 4173 Polr3a 0.2797259230284183

ENSMUST00000025740 1170 Rad9a 0.300170251375781

ENSMUST00000022998 2685 Mtbp 0.18118848528752482

ENSMUST00000029297 1509 Slc7a11 0.20139168585286696

ENSMUST00000022791 1158 Fbxo4 0.20075720329427943

ENSMUST00000027639 1557 Marco 0.21672946203544846

ENSMUST00000024981 573 Hn1l 0.2009029137131676

ENSMUST00000024709 1914 Cd2ap 0.1547517559312115

ENSMUST00000026607 1989 Chm 0.14657673541761776

ENSMUST00000025802 633 Nudt8 0.27693452565126203

ENSMUST00000029061 3204 Sall4 0.2941952121102151

ENSMUST00000029385 201 Serp1 0.23698702869817534

ENSMUST00000025595 1842 Fam111a 0.1820788894198784

ENSMUST00000023339 480 Gcsam 0.22508519724435036

ENSMUST00000029545 2079 Crtc2 0.24928793854655382

ENSMUST00000028049 2448 Rabgap1l 0.18342140840447632

ENSMUST00000027797 2568 Nvl 0.158646634543885

ENSMUST00000022345 780 Dnajc9 0.2607227950057386

ENSMUST00000022287 1032 Fst 0.31434262595509355

ENSMUST00000025092 894 Tmem178 0.2828836145235507

ENSMUST00000029803 654 Eif4e 0.1916347897686619

ENSMUST00000024704 420 Flywch2 0.346397441635843

ENSMUST00000022641 1404 Adamdec1 0.20233423973570616

ENSMUST00000029201 3207 Pik3ca 0.1998650839570451

ENSMUST00000026658 5703 Tnrc6c 0.25804456259726255

ENSMUST00000025110 1278 Syt4 0.23400326048027428

ENSMUST00000024792 969 Treml1 0.265140740876263

ENSMUST00000026487 249 Ier3ip1 0.21278057739919154

ENSMUST00000028259 3078 Ifih1 0.19673567025951658

ENSMUST00000024627 5136 Chd1 0.18575508106808153

ENSMUST00000022784 1092 Haus4 0.3023291529268619

ENSMUST00000029030 645 Edn3 0.27100749754149295

ENSMUST00000028111 807 Il2ra 0.2433632349422764

ENSMUST00000027103 2070 Fastkd2 0.17252412843427767

ENSMUST00000027706 2193 Lrrn2 0.3120447888475775

ENSMUST00000023154 636 Cldn1 0.2632689089222322

ENSMUST00000027202 3495 Sgol2a 0.15884016767285455

ENSMUST00000026677 873 Nat1 0.18330905076614265

ENSMUST00000026259 909 Pitx3 0.3364835746963691

ENSMUST00000022461 249 Dph3 0.24033521589053722

ENSMUST00000028612 2163 Pamr1 0.262515295842684

ENSMUST00000028177 1458 Olfm1 0.3363400265573495

ENSMUST00000025791 1944 Adrbk1 0.3493996985635857

ENSMUST00000022316 420 Dydc2 0.2570940529843221

ENSMUST00000029805 2685 Mttp 0.23794185701777668

ENSMUST00000029275 510 Il2 0.24702381616745056

ENSMUST00000027241 1731 Il1r1 0.18721417225879552

ENSMUST00000023714 1617 4732456N10Rik 0.32415090192059565

ENSMUST00000026398 735 Mettl7b 0.33248580719512594

ENSMUST00000026387 753 Sbds 0.3183522321742696

ENSMUST00000028667 2790 Dgkz 0.33806345316830644

ENSMUST00000027499 642 Bok 0.30711612314880266

ENSMUST00000022820 849 Dhrs2 0.2942983954527096

ENSMUST00000024011 1509 Kcnk5 0.31431776036747866

ENSMUST00000022121 816 Zcchc9 0.22945608659511865

ENSMUST00000022311 1380 Oxsm 0.1812309551036618

ENSMUST00000029164 780 Sla2 0.2822423649960293

ENSMUST00000027057 1179 Terf1 0.1820879911000471

ENSMUST00000026360 2304 Itgb8 0.18136065668232812

ENSMUST00000029038 399 Fabp9 0.21566919151884262

ENSMUST00000023226 13632 Plec 0.3666443257657801

ENSMUST00000027198 1731 Orc2 0.15203815339633509

ENSMUST00000029172 312 Ghrh 0.40827945262454335

ENSMUST00000025253 6477 Prrc2a 0.24520946682742087

ENSMUST00000029477 1428 Slc25a24 0.22845081858074623

ENSMUST00000029213 1497 Ocstamp 0.28547379445709875

ENSMUST00000026479 1209 Dctn2 0.2850747193196187

ENSMUST00000024967 2808 Msh2 0.1947491217228214

ENSMUST00000022380 1170 Psmc6 0.14668775695372455

ENSMUST00000026011 969 Sfxn2 0.3182709682649053

ENSMUST00000028804 1731 Cdc25b 0.29893971484093407

ENSMUST00000022437 1746 Hacl1 0.21988477509674303

ENSMUST00000022620 1539 Chrna2 0.3526146579665107

ENSMUST00000027405 1836 Slc23a3 0.24458536616797466

ENSMUST00000025430 4749 Setbp1 0.2730504532154378

ENSMUST00000026601 2235 Satl1 0.1674276768974936

ENSMUST00000025250 3465 Bag6 0.2720347164238812

ENSMUST00000026411 1584 Mmp19 0.2570924632510739

ENSMUST00000025932 1749 Shoc2 0.17598395175499168

ENSMUST00000022007 549 1700001L19Rik 0.30255445897282934

ENSMUST00000024206 1023 Gnb3 0.31964127381991

ENSMUST00000025208 1074 Dnajc18 0.24069624548833968

ENSMUST00000029651 1905 Gstcd 0.20737353155423793

ENSMUST00000027601 2466 Mcm6 0.21509412068092587

ENSMUST00000028125 1404 Zbtb43 0.2604555179668396

ENSMUST00000028248 2184 Ttll11 0.28403718920687865

ENSMUST00000029007 513 Fam209 0.257929353227708

ENSMUST00000023614 2292 Dyrk1a 0.2026615928578279

ENSMUST00000023452 1626 Ccdc116 0.28109901241740937

ENSMUST00000025681 4656 Cdc42bpg 0.32379100434344504

ENSMUST00000023710 1575 Krt71 0.3945741460476712

ENSMUST00000027032 6288 Rp1 0.15582096462703227

ENSMUST00000026292 13137 Huwe1 0.19331279243132263

ENSMUST00000028580 1104 Ccdc34 0.19437507315549063

ENSMUST00000022135 519 Ak6 0.18575444634164723

ENSMUST00000022185 1200 F2rl1 0.2826978090404019

ENSMUST00000025045 411 Uqcc2 0.3664720949024376

ENSMUST00000023752 816 Aqp2 0.3372566539018938

ENSMUST00000027185 1257 Stradb 0.19181194077533506

ENSMUST00000025840 1428 Mtl5 0.2766917799989831

ENSMUST00000029785 360 Riiad1 0.24288847023469654

ENSMUST00000024734 438 Mrpl14 0.3406208009623748

ENSMUST00000024976 1170 Spsb3 0.33564655317750347

ENSMUST00000022921 1497 Angpt1 0.19079248098467483

ENSMUST00000027157 687 Rpe 0.21771164867055967

ENSMUST00000029445 570 Nras 0.26123734375230256

ENSMUST00000025675 714 Naa40 0.2979360411908575

ENSMUST00000024988 4992 C3 0.31729253892898185

ENSMUST00000023365 1353 Bfar 0.24490509339682653

ENSMUST00000022682 2202 Sorbs3 0.2862801018261502

ENSMUST00000029516 1917 Tchhl1 0.19190049071225662

ENSMUST00000027783 585 Desi2 0.1751689078946249

ENSMUST00000023140 558 Tnfrsf17 0.28210521945137446

ENSMUST00000023348 657 Gtpbp8 0.146869850587367

ENSMUST00000023206 2754 Ercc4 0.25078881970883055

ENSMUST00000029815 408 Cisd2 0.28095308442742983

ENSMUST00000029753 1677 Ecm1 0.3026661656998721

ENSMUST00000027817 7248 Spta1 0.2358472097796559

ENSMUST00000027298 609 Cfc1 0.2652979032677427

ENSMUST00000025196 831 Psmb8 0.29429109328281733

ENSMUST00000023672 597 Rcan1 0.2672328683136907

ENSMUST00000023343 945 Atg3 0.1780179319681768

ENSMUST00000026256 903 Fbxl15 0.2907283772952424

ENSMUST00000029450 417 Tshb 0.2588532947395538

ENSMUST00000023362 933 Ntan1 0.17986438153922368

ENSMUST00000023144 156 Prm1 0.28660925534627524

ENSMUST00000023203 1491 Gpt 0.3122453752827606

ENSMUST00000024916 2103 Lhcgr 0.2226746826225304

ENSMUST00000024099 1845 Ache 0.3085500996574258

ENSMUST00000023353 2589 Mcm4 0.20360340405286018

ENSMUST00000029848 5202 Col24a1 0.1743647327842946

ENSMUST00000023328 318 Retnlb 0.23708448288976774

ENSMUST00000029421 1146 Ptx3 0.2768220761928847

ENSMUST00000026573 1995 Lmntd2 0.27297901234132016

ENSMUST00000025914 624 Vegfb 0.3140676497995599

ENSMUST00000027637 366 3110009E18Rik 0.1498877816524899

ENSMUST00000027215 678 Tex30 0.15029761256461507

ENSMUST00000022358 5499 Zswim8 0.2657479637393092

ENSMUST00000028783 1524 Spint1 0.31366708008235156

ENSMUST00000022718 1329 Ednrb 0.2381514590881081

ENSMUST00000028280 5517 Col5a1 0.253658489482624

ENSMUST00000027263 1119 Stk17b 0.1530072688851322

ENSMUST00000025541 1080 Gnaq 0.28389255316258866

ENSMUST00000022766 1860 Tox4 0.22485504985965532

ENSMUST00000025686 576 Ankrd22 0.21716072895824035

ENSMUST00000022204 2118 Kif2a 0.15658380435276484

ENSMUST00000029485 507 1700013F07Rik 0.23547557025615154

ENSMUST00000028132 2184 Lrsam1 0.33771404381527736

ENSMUST00000029483 1620 Clcc1 0.21207227739688964

ENSMUST00000027650 1173 Cd55 0.16199492044546923

ENSMUST00000029465 1122 Hsd3b1 0.25394107108232916

ENSMUST00000022519 984 Anxa8 0.30629354981435863

ENSMUST00000029277 2679 Spata5 0.15956422406971893

ENSMUST00000024270 801 Cdca3 0.21732198932768618

ENSMUST00000028148 1764 Fpgs 0.3185531801877137

ENSMUST00000022228 1410 Cwc27 0.19834156883056234

ENSMUST00000027532 1299 Scly 0.31067190960418845

ENSMUST00000022331 1566 Opn4 0.3145027403368231

ENSMUST00000028309 1605 Ccdc183 0.29727369522511843

ENSMUST00000025278 447 Mrpl27 0.2540272227191073

ENSMUST00000022369 3201 Vcl 0.2371349339861436

ENSMUST00000024866 4008 Xdh 0.28034513847564146

ENSMUST00000028467 672 Prg2 0.21991483324419497

ENSMUST00000028045 4371 Mrc1 0.2031748145715319

ENSMUST00000026879 1077 Gdap1 0.194071745586185

ENSMUST00000029654 1491 Glrb 0.20377198891298262

ENSMUST00000029910 2763 Nsmaf 0.20951356498426046

ENSMUST00000027065 300 Tmem14a 0.17339246502231218

ENSMUST00000023036 327 Rbx1 0.28166740527628037

ENSMUST00000025421 1083 Seh1l 0.2074865242041644

ENSMUST00000026537 1515 Paox 0.2672692316115295

ENSMUST00000029632 696 Lrat 0.26454903320180917

ENSMUST00000027569 2121 Slco6c1 0.1315241090306908

ENSMUST00000024518 555 Rhebl1 0.23610181407207628

ENSMUST00000028694 1275 Pacsin3 0.3415683050669265

ENSMUST00000026125 768 Alyref 0.2910888143112653

ENSMUST00000026414 2193 Dgka 0.2663057204652961

ENSMUST00000028836 1185 Bmp2 0.2602301193997793

ENSMUST00000025836 579 Mrpl11 0.2734494820662346

ENSMUST00000024660 1344 Smoc2 0.2979839781893321

ENSMUST00000024957 660 Pigf 0.1579185846334006

ENSMUST00000028361 483 Il1f6 0.224347987393139

ENSMUST00000023561 1767 Senp2 0.20756692931055956

ENSMUST00000029748 1215 Fcgr1 0.22152140195615885

ENSMUST00000024575 2202 Rps6ka2 0.31234024089637275

ENSMUST00000024944 2058 Slc3a1 0.23319945758077165

ENSMUST00000023906 522 Reg2 0.22960235437559356

ENSMUST00000027066 1776 Eya1 0.20542184383555426

ENSMUST00000023965 1032 Cfhr1 0.14604925355991397

ENSMUST00000027451 2961 Epha4 0.25669624537691893

ENSMUST00000023448 1797 Aifm3 0.32036468568215876

ENSMUST00000029796 1677 Rap1gds1 0.16107591802510254

ENSMUST00000023062 429 Tomm22 0.2507413308863083

ENSMUST00000029908 1230 Faxc 0.27496661843046816

ENSMUST00000028905 1914 Pcsk2 0.2904852348026132

ENSMUST00000028984 720 Bpifa3 0.26968065601834723

ENSMUST00000022612 993 Pbk 0.1643085946208109

ENSMUST00000029527 420 Lce1g 0.3411300466114297

ENSMUST00000029740 918 Rnf115 0.19327817061502395

ENSMUST00000028683 1518 Pdia3 0.20640080829981253

ENSMUST00000029240 1572 Slc2a2 0.229597876373752

ENSMUST00000028769 2382 Ptpra 0.26606887162472126

ENSMUST00000024755 756 Clic5 0.35671191442572997

ENSMUST00000025992 798 Kctd1 0.23095998547804855

ENSMUST00000029559 1383 Il6ra 0.2725210074469902

ENSMUST00000028128 1917 Odf2 0.2693697733788507

ENSMUST00000023854 843 Fhl1 0.3877639825484883

ENSMUST00000022137 1668 Marveld2 0.24948963459575219

ENSMUST00000022707 1719 Gpc5 0.22442317156948072

ENSMUST00000023351 480 Mzt2 0.2634415084487554

ENSMUST00000022865 1740 Mtdh 0.19288161672247411

ENSMUST00000027897 1302 Smyd2 0.35144432420117283

ENSMUST00000029257 3528 Atp11b 0.1420325682329713

ENSMUST00000024885 3159 Cebpz 0.20857635406435243

ENSMUST00000026693 1926 Zfp202 0.2312763519852965

ENSMUST00000027290 5634 Tmem131 0.19797411085538913

ENSMUST00000027981 1566 Uap1 0.2183087483107769

ENSMUST00000026236 1002 Tlx1 0.3302018973953586

ENSMUST00000023959 2619 Grm2 0.35284710068429764

ENSMUST00000025237 423 Tslp 0.203247766433807

ENSMUST00000023861 1164 Serpinb3d 0.1952509254528392

ENSMUST00000028883 747 Pdyn 0.29392918408365076

ENSMUST00000026262 1587 Hexa 0.27958521755840443

ENSMUST00000026552 1482 Cyp2e1 0.25260082295142816

ENSMUST00000022603 1005 Lect1 0.2814960804653197

ENSMUST00000022469 4782 Nisch 0.29499611617077676

ENSMUST00000027916 927 Bpnt1 0.2708540079456436

ENSMUST00000028117 2148 Yme1l1 0.1302675747658621

ENSMUST00000022720 1287 Fbxl3 0.19687622535172628

ENSMUST00000029888 918 Rmdn1 0.16971407753603102

ENSMUST00000027863 915 Atp1b1 0.3245842505510345

ENSMUST00000028348 2364 Itgb6 0.23837871401329128

ENSMUST00000023074 996 Parvg 0.32908645904045775

ENSMUST00000028730 1713 Mkks 0.14553851614596544

ENSMUST00000027743 768 Stx6 0.28399156605189485

ENSMUST00000026551 1719 Dpysl4 0.3042883103674186

ENSMUST00000024572 1170 Rsph3b 0.24126308476408306

ENSMUST00000029005 924 Rtfdc1 0.31473956829433913

ENSMUST00000028592 1125 Eif3m 0.18967906353741887

ENSMUST00000022550 2760 Extl3 0.3248256351048338

ENSMUST00000025505 1383 Dctn4 0.2449372061392077

ENSMUST00000023554 1437 Dirc2 0.1956847060106458

ENSMUST00000028282 2181 Brd3 0.3021511130125386

ENSMUST00000023486 2292 Tfrc 0.16347947109745045

ENSMUST00000027266 462 Ormdl1 0.15764123627101995

ENSMUST00000023911 1554 Nagpa 0.32957818023270574

ENSMUST00000022538 309 Mrpl57 0.3530839940148797

ENSMUST00000025020 1401 Rgs11 0.34970188585977974

ENSMUST00000025704 795 Cdca5 0.20743411252582492

ENSMUST00000029380 1188 Wwtr1 0.2915059748641174

ENSMUST00000024832 906 Rsph1 0.27371767559838844

ENSMUST00000022694 1152 Dmtn 0.31714213858135704

ENSMUST00000029490 1593 Ahcyl1 0.2438338035876819

ENSMUST00000025007 561 Nme4 0.3136393113918288

ENSMUST00000022586 1455 Nufip1 0.19829872933194162

ENSMUST00000029865 720 Trp53inp1 0.21274115593175297

ENSMUST00000024778 639 Med20 0.33603480234071453

ENSMUST00000027285 780 Unc50 0.22344964438503784

ENSMUST00000026839 957 Prps2 0.24148242708520262

ENSMUST00000023918 1929 Ivns1abp 0.1871830942130495

ENSMUST00000024849 3225 Emilin2 0.2848989274251618

ENSMUST00000025891 2142 Capn1 0.33951433769123657

ENSMUST00000024905 1947 Ralbp1 0.26850941509655035

ENSMUST00000024816 2514 Cmtr1 0.3095223416684963

ENSMUST00000022343 1059 Nudt13 0.24340230167126825

ENSMUST00000027139 2493 Wdr75 0.18410280819897185

ENSMUST00000022857 1377 Ttc23l 0.20988518192455866

ENSMUST00000027421 6078 Trip12 0.15863003152629634

ENSMUST00000025402 1347 Gnal 0.29295902901916826

ENSMUST00000025065 1389 Nudt12 0.18218383469425928

ENSMUST00000029769 825 Gclm 0.20667404589814808

ENSMUST00000025243 2301 Iws1 0.213160062387723

ENSMUST00000022849 2169 Tars 0.24518328783770657

ENSMUST00000029565 666 Slc50a1 0.2949815898067004

ENSMUST00000026014 2253 Efhc2 0.17887376718071335

ENSMUST00000028102 2871 Kif5c 0.298718716816037

ENSMUST00000026506 726 Clns1a 0.18992317584303994

ENSMUST00000022186 300 S100z 0.3310368574474094

ENSMUST00000022638 2547 Nefm 0.33078656927261013

ENSMUST00000028121 1419 Acbd5 0.17137616366586625

ENSMUST00000028336 681 Arl6ip6 0.22036245397967696

ENSMUST00000023146 963 Nubp1 0.29616799221192786

ENSMUST00000025719 342 Mlana 0.24318160595861102

ENSMUST00000029128 366 Map1lc3a 0.44074170888973035

ENSMUST00000027393 2298 Bard1 0.19648141106772077

ENSMUST00000025490 1332 Prrc1 0.2529110119814857

ENSMUST00000026018 570 Dusp21 0.23251501321619117

ENSMUST00000028702 882 Adal 0.19315656849582244

ENSMUST00000027492 1041 Mterf4 0.22292586789968794

ENSMUST00000027775 495 Efcab2 0.17908379162969765

ENSMUST00000024939 699 Sbpl 0.2599083695228001

ENSMUST00000026740 8835 Col7a1 0.22666662481652047

ENSMUST00000026218 1614 Cwf19l1 0.22937100030204186

ENSMUST00000024705 969 Slc25a27 0.20496437027407857

ENSMUST00000022019 435 Il9 0.2546500793192004

ENSMUST00000023694 873 1110004E09Rik 0.2704850531234403

ENSMUST00000029139 1638 Trim55 0.24992696175058268

ENSMUST00000028780 672 Chac1 0.2866132532872552

ENSMUST00000025069 1038 Crem 0.15402247156150656

ENSMUST00000023231 966 Tsta3 0.3258864025633525

ENSMUST00000027015 1209 Casp1 0.18249374678256508

ENSMUST00000027472 723 Efhd1 0.3730098989210538

ENSMUST00000025476 870 Txnl1 0.18046927429753468

ENSMUST00000026826 846 Rab40c 0.3668314088887086

ENSMUST00000025563 549 Fth1 0.28940618014388003

ENSMUST00000027367 786 Ctdsp1 0.3185055688128037

ENSMUST00000029692 660 Rit1 0.2791425594159034

ENSMUST00000029400 2253 Mme 0.17701354628939256

ENSMUST00000022124 1986 Cd180 0.1946212554604116

ENSMUST00000028059 834 Rsu1 0.2741700518055613

ENSMUST00000028910 1017 Mgme1 0.21909037811579749

ENSMUST00000023813 1098 Tarbp2 0.27753909454533776

ENSMUST00000026750 3099 Cnksr2 0.18894989528442158

ENSMUST00000025698 387 Gpha2 0.3459287547947541

ENSMUST00000023750 954 Faim2 0.31532342335607116

ENSMUST00000029092 1245 Arfgap1 0.29093389893952665

ENSMUST00000028243 960 4930568D16Rik 0.17624385087471803

ENSMUST00000026865 2505 Jade1 0.285014116671883

ENSMUST00000025027 534 Cuta 0.24491683829565686

ENSMUST00000025582 744 Ms4a6d 0.2074767845703703

ENSMUST00000022871 609 Sdc2 0.22164409089374731

ENSMUST00000026175 930 Fn3k 0.31685425464476624

ENSMUST00000023180 2304 Mefv 0.2426132196741646

ENSMUST00000023718 1440 5430421N21Rik 0.41096239476204377

ENSMUST00000022609 1701 Elp3 0.23733097431692643

ENSMUST00000029772 1977 Rfx5 0.2085846320718403

ENSMUST00000028758 1380 Itpka 0.32269467629309484

ENSMUST00000028251 573 Rbm18 0.22475238974716594

ENSMUST00000028807 1275 Ivd 0.27132157999692

ENSMUST00000026922 1065 Homer2 0.3291576242261787

ENSMUST00000023043 1455 Adsl 0.2821395342715843

ENSMUST00000023760 1050 Gpd1 0.3374020201295506

ENSMUST00000026270 1764 Sacm1l 0.15705619585393668

ENSMUST00000023390 1341 Drd3 0.2842873308100968

ENSMUST00000023269 474 Rpl24 0.2462608115857413

ENSMUST00000023387 1038 Qtrtd1 0.2226924739183237

ENSMUST00000027943 357 Batf3 0.38978439325843256

ENSMUST00000026378 1989 Padi1 0.36235396657758395

ENSMUST00000025391 1719 Mtm1 0.1645204135146769

ENSMUST00000023769 810 Atf1 0.19983186698971256

ENSMUST00000022915 1560 Dpys 0.2614750081500435

ENSMUST00000023432 831 Nit2 0.20678642548976833

ENSMUST00000023602 687 Prl2a1 0.20267831658566027

ENSMUST00000026148 735 Cbr2 0.3078294844407348

ENSMUST00000023781 516 1700011A15Rik 0.33730447594859747

ENSMUST00000027338 315 Sdhaf4 0.20460386281102155

ENSMUST00000026162 579 Sectm1a 0.3056967997536303

ENSMUST00000029626 831 Casp6 0.3107793005777114

ENSMUST00000026241 807 Fgf8 0.38074642099399747

ENSMUST00000025048 2118 1700061G19Rik 0.2999361244128396

ENSMUST00000029549 747 Tpm3 0.28103391322887383

ENSMUST00000023799 1596 Krt79 0.34818153580971445

ENSMUST00000026558 1593 Ric8 0.296013843467532

ENSMUST00000029142 738 Eif6 0.32365939333466204

ENSMUST00000024119 1443 Trip6 0.2723428270683385

ENSMUST00000022386 2136 Samd4 0.27303162861446884

ENSMUST00000025266 609 Lta 0.2877245434608669

ENSMUST00000025774 2637 Sf3b2 0.27316751409545736

ENSMUST00000023593 744 Adipoq 0.24658267490178137

ENSMUST00000023572 1098 Cxadr 0.21855828794097454

ENSMUST00000027725 1707 Klhl12 0.2158746466570434

ENSMUST00000029459 1497 Gdap2 0.21616713274011184

ENSMUST00000025354 1134 Pggt1b 0.24445363125022299

ENSMUST00000026196 1242 Got1 0.27318162147579456

ENSMUST00000028880 2046 Slc20a1 0.21416983008110965

ENSMUST00000027356 1602 Cyp27a1 0.28042111287851934

ENSMUST00000025215 1398 Sil1 0.31112195881932836

ENSMUST00000024015 351 Guca2a 0.27905841675549903

ENSMUST00000027131 2502 Slc39a10 0.18435394844166716

ENSMUST00000028340 444 Tmem210 0.2468419517586681

ENSMUST00000022511 4131 Zmym2 0.16381887980702015

ENSMUST00000028239 2343 Gsn 0.33548226853518115

ENSMUST00000022147 867 Smn1 0.20216947718236228

ENSMUST00000022553 1014 Cab39l 0.22994190682223262

ENSMUST00000029382 1113 Ppid 0.15063829162923925

ENSMUST00000027251 3750 Rev1 0.17889967454516872

ENSMUST00000023924 369 Rpp14 0.19285079287708662

ENSMUST00000023687 999 Ifngr2 0.22805286307189052

ENSMUST00000027090 528 Crygb 0.34427879895889635

ENSMUST00000026989 912 4833439L19Rik 0.2588666392819815

ENSMUST00000026409 462 Ormdl2 0.2642367075260737

ENSMUST00000023759 1548 Smarcd1 0.2859309543324732

ENSMUST00000026172 987 Ankrd2 0.3039069903154023

ENSMUST00000029667 2802 Kcnq5 0.22592272646068978

ENSMUST00000028748 2130 Capn3 0.3164587013311463

ENSMUST00000023467 1575 Pak2 0.19737065087163386

ENSMUST00000022945 2007 Shcbp1 0.15961609913630406

ENSMUST00000028608 3075 Nat10 0.25451312007551413

ENSMUST00000027121 1503 Rftn2 0.22645410979115269

ENSMUST00000026681 666 Tma16 0.18413999271973097

ENSMUST00000028633 8622 Fbn1 0.27387573977254914

ENSMUST00000028915 561 Rbbp9 0.2878391360988728

ENSMUST00000025202 1536 Kcnc1 0.358690283824329

ENSMUST00000025319 453 Rpp21 0.3411919459279772

ENSMUST00000023335 1533 Pvrl3 0.15765302336929785

ENSMUST00000025079 6513 Svil 0.25114113258453974

ENSMUST00000023845 990 H2-M10.2 0.27836787031509663

ENSMUST00000027165 657 Cd28 0.2489717357838844

ENSMUST00000029783 1581 Snx27 0.2285062065379199

ENSMUST00000028814 981 Rassf2 0.32910676298649183

ENSMUST00000023024 906 Tef 0.36785602490414016

ENSMUST00000028386 3387 Nckap1 0.17413826509230104

ENSMUST00000023869 597 Denr 0.17353336410429812

ENSMUST00000029727 1890 Fbxw7 0.1838383202330936

ENSMUST00000029271 2733 Trpc3 0.2640369072165342

ENSMUST00000028856 465 Mall 0.33169681647109317

ENSMUST00000028764 378 Oxt 0.42235040597510626

ENSMUST00000027036 693 Lypla1 0.17777977188572383

ENSMUST00000028842 1173 Usp50 0.28122891197014327

ENSMUST00000022271 2046 Acox2 0.25899499902639656

ENSMUST00000025904 633 Prdx5 0.2442002509761373

ENSMUST00000023221 1866 Gpaa1 0.2939527909097394

ENSMUST00000023087 1053 Twf1 0.1981559957352615

ENSMUST00000028926 897 Napb 0.2609326684663411

ENSMUST00000022803 795 Psmb5 0.26164715569607444

ENSMUST00000029406 2739 Vmn2r1 0.18072556149512503

ENSMUST00000027885 1473 Angptl1 0.23591356053853976

ENSMUST00000022895 1878 Grhl2 0.3003604055219171

ENSMUST00000028966 540 Defb22 0.2902988965107154

ENSMUST00000023285 1416 Tymp 0.2596221552992109

ENSMUST00000025521 807 Cdx1 0.318120697012379

ENSMUST00000025217 2040 Hspa9 0.18864928657734759

ENSMUST00000023270 2571 Cep97 0.1993211790454187

ENSMUST00000026540 450 Prap1 0.312333044470121

ENSMUST00000025679 816 Otub1 0.35796911584567803

ENSMUST00000029116 1080 Pcmtd2 0.23170040062798516

ENSMUST00000026667 1236 Eif4a3 0.3098137865415195

ENSMUST00000023453 666 Sdf2l1 0.2891041854013112

ENSMUST00000023123 4464 Col2a1 0.23952103404402647

ENSMUST00000022576 1269 Cpb2 0.21442984707216026

ENSMUST00000028114 1581 Pfkfb3 0.2802417658604985

ENSMUST00000022176 2664 Hmgcr 0.20836780555565607

ENSMUST00000025818 2292 Rin1 0.2920510939516999

ENSMUST00000029838 1050 Rpf1 0.190256983410781

ENSMUST00000024042 1053 Creld2 0.33202588399984595

ENSMUST00000028935 414 Cst9 0.2662971037139878

ENSMUST00000029744 3504 Itga10 0.2312481936022102

ENSMUST00000027933 2190 Dtl 0.1938776163481576

ENSMUST00000025946 1494 Cyp26a1 0.2839010734911558

ENSMUST00000023055 318 Dnal4 0.4594478150731963

ENSMUST00000025631 648 Ostf1 0.2550974536489011

ENSMUST00000027736 2379 Zc3h11a 0.1966659846108064

ENSMUST00000024596 1671 Slc22a1 0.30259125240050055

ENSMUST00000022464 342 Msmb 0.23364230817187095

ENSMUST00000024928 975 Prss21 0.2642215732889278

ENSMUST00000027153 1293 Acadl 0.20467262643977074

ENSMUST00000024107 636 Wfdc1 0.27615186891783516

ENSMUST00000025997 717 Smndc1 0.19263260720154235

ENSMUST00000022091 429 8030423J24Rik 0.28846897153612855

ENSMUST00000029069 1008 E2f5 0.1765779739951298

ENSMUST00000025826 1371 Slc29a2 0.3489961879492362

ENSMUST00000026032 1062 Pcgf6 0.19576034514184898

ENSMUST00000029552 570 4933434E20Rik 0.20372561560642607

ENSMUST00000022122 1260 Ckmt2 0.2666872114309465

ENSMUST00000028909 1215 Snx5 0.21379616586770794

ENSMUST00000023460 471 Ncbp2 0.21344998024827894

ENSMUST00000026795 2025 Alox5 0.3348346908110951

ENSMUST00000026119 1458 Gcgr 0.34286622860269295

ENSMUST00000029336 3006 Dhx36 0.16042530875561536

ENSMUST00000027884 624 Tex35 0.22038116924828996

ENSMUST00000029658 2838 Enpep 0.2336339679436173

ENSMUST00000022543 1299 Micu2 0.2167263603871163

ENSMUST00000026076 1407 Gfra1 0.3019780863721006

ENSMUST00000022842 1626 Cct5 0.2521555319737699

ENSMUST00000022642 2382 Adam28 0.21111758590526503

ENSMUST00000023356 810 Snai2 0.24064872176256935

ENSMUST00000023363 1971 Rrn3 0.19191486606470065

ENSMUST00000023726 432 Lalba 0.23618233044836265

ENSMUST00000028727 621 Snap25 0.2963295791586953

ENSMUST00000025374 1017 Pou4f3 0.29158290853672325

ENSMUST00000025472 1488 Pcyox1l 0.3496571889894911

ENSMUST00000024791 684 Trem2 0.3111990576725598

ENSMUST00000027128 3333 Ccdc150 0.1962128922385911

ENSMUST00000027444 453 Pde6d 0.2292920394973172

ENSMUST00000023132 1608 Pde1b 0.3172327153796917

ENSMUST00000025500 1860 Slc27a6 0.21906281119225976

ENSMUST00000023007 3750 Adcy8 0.26522316857464595

ENSMUST00000025363 627 Hbegf 0.2446926634899172

ENSMUST00000022328 1986 Ldb3 0.34266610249449325

ENSMUST00000029141 1857 Mmp24 0.32034048265072246

ENSMUST00000027565 1161 Serpinb3c 0.18981510102910576

ENSMUST00000025585 1254 Gif 0.24229168366206424

ENSMUST00000028279 2019 Strbp 0.15827859139710268

ENSMUST00000027027 2664 Cwf19l2 0.1796613855238029

ENSMUST00000024763 591 Mrps18a 0.2619633645858129

ENSMUST00000025929 885 Hrasls5 0.253883309584934

ENSMUST00000028990 1767 Cdk5rap1 0.22415151374143916

ENSMUST00000025364 774 Yipf5 0.18588400945901581

ENSMUST00000023329 417 Retnla 0.2604807039814864

ENSMUST00000026455 792 Mip 0.26376442016203006

ENSMUST00000026129 1215 Pcyt2 0.3571037460995876

ENSMUST00000025271 1059 Pou5f1 0.2973370824486898

ENSMUST00000029709 1101 Sh2d2a 0.27139831662261393

ENSMUST00000024004 435 Ccl25 0.28511691695709634

ENSMUST00000026273 1908 Slc6a20b 0.3081572804732226

ENSMUST00000028328 1488 Entpd2 0.295992436490793

ENSMUST00000025036 3261 Kdm4b 0.34460858344642914

ENSMUST00000023143 486 Litaf 0.30812656178532216

ENSMUST00000025764 450 Cst6 0.326898965460115

ENSMUST00000022566 3735 Spata13 0.30986718252114226

ENSMUST00000022563 1854 Mtmr6 0.20122210731018314

ENSMUST00000025288 3936 Zfp521 0.31007333481348975

ENSMUST00000027061 477 Il17a 0.3150292785873734

ENSMUST00000027475 3876 Gigyf2 0.21124891565465137

ENSMUST00000027384 1779 Atic 0.2047013487481773

ENSMUST00000024857 318 Lbh 0.321478943603281

ENSMUST00000022293 888 Vdac2 0.21608105551500828

ENSMUST00000022429 543 Arf4 0.21064312430660878

ENSMUST00000025357 582 Ap3s1 0.16117979740371466

ENSMUST00000029840 939 Ctbs 0.19140998425570596

ENSMUST00000023530 639 Ropn1 0.2415018909612612

ENSMUST00000022105 1848 Slc6a18 0.3032226647961668

ENSMUST00000025649 3423 Ddb1 0.2604851771860783

ENSMUST00000028410 11355 Xirp2 0.16353245740246733

ENSMUST00000027992 768 Enkur 0.2400843155413866

ENSMUST00000023568 822 Chodl 0.19959335905890366

ENSMUST00000021991 1239 Mterf3 0.16739829907963144

ENSMUST00000025846 2535 Ppp6r3 0.19132875323859957

ENSMUST00000029454 1248 Casq2 0.2734681467463325

ENSMUST00000028527 2661 Kif18a 0.15038587933013256

ENSMUST00000025329 873 Trim15 0.2921309663203169

ENSMUST00000027856 2631 Dcaf6 0.18040550385825566

ENSMUST00000025547 735 Timm21 0.1748149915349845

ENSMUST00000025388 714 Ftmt 0.26390161078262087

ENSMUST00000027502 1182 Atg4b 0.252807564191765

ENSMUST00000025910 615 Bad 0.28564122546606385

ENSMUST00000029814 2640 Manba 0.2573702341059429

ENSMUST00000027753 3582 Lamc2 0.261802196535607

ENSMUST00000025003 1395 Sox8 0.3304968312885409

ENSMUST00000028859 1899 Acoxl 0.2687893820634497

ENSMUST00000024897 750 Vapa 0.19002981392151466

ENSMUST00000027082 525 Crygf 0.4888160342886735

ENSMUST00000026890 609 Clec3b 0.3903322935786261

ENSMUST00000025568 489 Tmem138 0.3195089386207521

ENSMUST00000028663 1563 Creb3l1 0.3496314841744201

ENSMUST00000024854 2115 Clip4 0.21567633470050432

ENSMUST00000028583 594 Lin7c 0.15280038643432034

ENSMUST00000026723 657 Hprt 0.17212621839504644

ENSMUST00000029469 474 Reg4 0.2424064032117989

ENSMUST00000029684 1050 Scamp3 0.3190583894729649

ENSMUST00000022438 2574 Vmn2r88 0.16301428861170486

ENSMUST00000027362 2424 Plcd4 0.2565573331358908

ENSMUST00000028997 1254 Aurka 0.24625737913053242

ENSMUST00000027559 1878 Tnfrsf11a 0.27606533555673796

ENSMUST00000024179 2706 Nfatc4 0.27921693220677923

ENSMUST00000028205 870 BC005624 0.32308602921794544

ENSMUST00000027409 1410 Des 0.3537898615667149

ENSMUST00000026671 4008 Rptor 0.30752852069417275

ENSMUST00000028362 5172 Ly75 0.21540062809708962

ENSMUST00000026672 2472 Pde8a 0.2237665610024882

ENSMUST00000022704 801 Itm2b 0.31549344862300516

ENSMUST00000025850 822 Fosl1 0.3088346353988518

ENSMUST00000029777 831 Tmem56 0.1779279425479373

ENSMUST00000025745 651 Mrpl21 0.2241054425961134

ENSMUST00000022853 741 C1qtnf3 0.2612272558649208

ENSMUST00000025292 3135 Dhx16 0.31508073003117876

ENSMUST00000027257 750 Mitd1 0.1745875916234428

ENSMUST00000023734 1113 Wnt1 0.3490168637592666

ENSMUST00000022744 723 Gdnf 0.2026801187281968

ENSMUST00000028106 3189 Itga8 0.20599572527497792

ENSMUST00000027232 522 Ptp4a1 0.1586643256026943

ENSMUST00000029082 747 Psma7 0.2351028410775953

ENSMUST00000027657 1410 C4bp 0.19778776873969842

ENSMUST00000025724 801 Il33 0.20515158323162788

ENSMUST00000029598 1635 1700006A11Rik 0.14981503981181024

ENSMUST00000026555 765 Odf3 0.3496634981051697

ENSMUST00000026220 1080 Scd3 0.34416074911821287

ENSMUST00000023213 1182 Hgh1 0.2604877485197704

ENSMUST00000029448 2982 Sycp1 0.1252487124717165

ENSMUST00000025936 990 Tectb 0.29243694950853727

ENSMUST00000025383 897 Dtwd2 0.19736545545754644

ENSMUST00000023920 591 Tmem52 0.3245205948872099

ENSMUST00000028963 1209 Tbc1d20 0.3321371701922051

ENSMUST00000022245 1329 Mrps30 0.23410851906678068

ENSMUST00000025963 2424 Noc3l 0.1951844664135593

ENSMUST00000023405 561 Arl6 0.19863720598466036

ENSMUST00000025659 642 Zfand5 0.19131924412440815

ENSMUST00000022136 2067 Rad17 0.15485387446516719

ENSMUST00000025488 348 C330018D20Rik 0.19820306990126074

ENSMUST00000025061 309 Clpsl2 0.30567353369047856

ENSMUST00000029135 2106 Acss2 0.2894418660345587

ENSMUST00000027853 384 Mpc2 0.19315582688780336

ENSMUST00000027643 357 Atp6v1g3 0.30897442731536

ENSMUST00000027040 4767 Rb1cc1 0.14089826129181798

ENSMUST00000024733 2943 Aars2 0.29584693043351995

ENSMUST00000022548 657 1700129C05Rik 0.21491714598831238

ENSMUST00000029367 1812 Bche 0.16500192725743923

ENSMUST00000029186 2040 Dhx35 0.25635632454438806

ENSMUST00000028595 1536 Depdc7 0.18700749185074284

ENSMUST00000028175 1080 Cytip 0.23515597578873873

ENSMUST00000029696 1839 2810403A07Rik 0.18781710997624995

ENSMUST00000024870 1383 Slc30a6 0.18877719701687173

ENSMUST00000022901 1056 Rrm2b 0.1896192646531604

ENSMUST00000029719 2271 Dclk2 0.25336763408704993

ENSMUST00000028981 807 Mapre1 0.19258556968850188

ENSMUST00000026354 1530 Banp 0.31982913274586944

ENSMUST00000027422 1539 Slc16a14 0.2600544930931234

ENSMUST00000027843 1620 Fmo9 0.25229092976932566

ENSMUST00000023615 894 Dscr3 0.28639331421358794

ENSMUST00000022038 2178 Naa35 0.2205811612316918

ENSMUST00000027959 570 Cfap126 0.2418230194023645

ENSMUST00000023849 1290 Ccrn4l 0.3133392189710946

ENSMUST00000022690 2235 Fam160b2 0.3044492173103082

ENSMUST00000028866 918 Zc3h8 0.18220920917782954

ENSMUST00000029080 360 Cypt12 0.1773447664776508

ENSMUST00000029568 1047 Tmem144 0.21242503581438701

ENSMUST00000028214 1188 Sh3glb2 0.33412783054355183

ENSMUST00000022976 3480 E430025E21Rik 0.23560916272253576

ENSMUST00000023497 2046 Lmln 0.24734340534167046

ENSMUST00000025542 1113 Psat1 0.24801475145923915

ENSMUST00000026993 813 Tspan17 0.34030584472602904

ENSMUST00000023790 1914 Krt1 0.28128979281475985

ENSMUST00000025101 1056 Dync2li1 0.20877811640675686

ENSMUST00000027299 1104 Prss39 0.2660919802670425

ENSMUST00000022232 1758 4933425L06Rik 0.17019932937386814

ENSMUST00000022102 1620 Clptm1l 0.277830595652557

ENSMUST00000023352 12387 Prkdc 0.17221618396626429

ENSMUST00000026548 1737 Adgra1 0.34953908598392897

ENSMUST00000022499 927 Rnaseh2b 0.186750814978131

ENSMUST00000024817 1467 Rnf8 0.27292916421053476

ENSMUST00000022977 1719 Sqle 0.1823837982589396

ENSMUST00000028950 879 Sdcbp2 0.2646102162416424

ENSMUST00000023836 645 Mospd1 0.15340383392916093

ENSMUST00000029633 1146 Npy2r 0.2773325919833501

ENSMUST00000025403 873 Impa2 0.28068708078145593

ENSMUST00000023775 801 Cela1 0.33418420016155725

ENSMUST00000023075 795 C1qtnf6 0.3354003652274561

ENSMUST00000027947 1569 Angel2 0.18808758256371721

ENSMUST00000023159 1602 Mgrn1 0.30349956612956325

ENSMUST00000023165 7326 Crebbp 0.24425915859487066

ENSMUST00000029371 1293 Smad9 0.33690124044682834

ENSMUST00000028900 2520 Vps16 0.26886394176243417

ENSMUST00000028187 4746 Lamc3 0.3336259947599771

ENSMUST00000024748 1809 Gtpbp2 0.3020756493121241

ENSMUST00000025680 1188 Lipf 0.18067763895074604

ENSMUST00000023828 6681 Rttn 0.16351975600769414

ENSMUST00000023689 1773 Ifnar1 0.21347525520945795

ENSMUST00000023502 1353 Snx4 0.22670519173519937

ENSMUST00000025263 708 Tnf 0.2969648889204177

ENSMUST00000023830 894 Nus1 0.2394087367896094

ENSMUST00000022013 3288 Adcy2 0.25782770170280694

ENSMUST00000027112 2721 Nrp2 0.3019570518502743

ENSMUST00000028389 972 Frzb 0.2839914096742163

ENSMUST00000027172 1296 Ica1l 0.21091857425316293

ENSMUST00000027529 645 Rab17 0.3595252750841116

ENSMUST00000028351 1434 Dph7 0.22992089639293756

ENSMUST00000022964 642 Ebag9 0.1765583726791638

ENSMUST00000026994 2697 Unc5a 0.3697777828046994

ENSMUST00000026625 1017 Clybl 0.19197875077121015

ENSMUST00000027366 2484 Vil1 0.31572426069592924

ENSMUST00000029482 2040 Gpsm2 0.19648027527017597

ENSMUST00000028207 1881 Crat 0.3308395291464202

ENSMUST00000025397 483 Cplx4 0.22942064405448012

ENSMUST00000022592 951 Tnfsf11 0.2514461240752519

ENSMUST00000027467 1194 Serpine2 0.25910071562226294

ENSMUST00000028852 1299 Mrps5 0.2486722234991317

ENSMUST00000027769 1191 Tfb2m 0.14407399454444714

ENSMUST00000027560 1167 Htr4 0.2730733091070443

ENSMUST00000023812 2667 Map3k12 0.23887017403905297

ENSMUST00000028035 990 Cenpl 0.15198734882519194

ENSMUST00000027800 2415 Tmem63a 0.3263293082322224

ENSMUST00000027431 1440 Htr2b 0.2001644444490072

ENSMUST00000025803 819 Dkk1 0.264916941026303

ENSMUST00000025337 3795 Diap1 0.2424022057892508

ENSMUST00000023225 1038 Grina 0.33512205721934696

ENSMUST00000027629 1440 Tfcp2l1 0.28781876658257605

ENSMUST00000023442 1080 Lrrc74b 0.2032831982554248

ENSMUST00000022816 384 Sub1 0.1888748861723344

ENSMUST00000022460 1917 Galnt15 0.33817718697353427

ENSMUST00000022787 1596 Slc7a8 0.2962099962180015

ENSMUST00000028794 5106 Siglec1 0.2839248358083247

ENSMUST00000027780 1578 Acbd3 0.21787851359512264

ENSMUST00000022749 1686 C9 0.17525142868385485

ENSMUST00000028752 2628 Vps39 0.2712734692443646

ENSMUST00000027970 1020 Dusp12 0.21533783159741782

ENSMUST00000025211 567 Mzb1 0.28443799788187923

ENSMUST00000027062 606 Msc 0.33366372621891993

ENSMUST00000023652 2601 Grik1 0.2387774606588772

ENSMUST00000029907 996 Ubxn2b 0.15400935412749253

ENSMUST00000027089 525 Crygc 0.3716309193407354

ENSMUST00000026957 1440 Pramel7 0.20756010823534568

ENSMUST00000022782 2142 Lrp10 0.2693740326619935

ENSMUST00000028931 429 Cst8 0.22222613649699013

ENSMUST00000026208 4632 Abcc2 0.22712558020810739

ENSMUST00000023832 900 Rgn 0.19245312311133464

ENSMUST00000022048 1905 Slc6a19 0.3348260405403681

ENSMUST00000023630 870 Psmg1 0.23530190023477196

ENSMUST00000027053 1026 Rdh10 0.27088454467680023

ENSMUST00000022537 966 Prss52 0.21631543005501572

ENSMUST00000026227 2058 Peo1 0.22706347720283596

ENSMUST00000023069 1074 Fam118a 0.27957809530184974

ENSMUST00000027009 1260 Casp12 0.20146003628038003

ENSMUST00000029564 579 Pmvk 0.3372000842795384

ENSMUST00000022239 2676 Parp8 0.2183196161515665

ENSMUST00000027214 3513 Ercc5 0.21076178562052897

ENSMUST00000028052 1113 Rabgap1l 0.24410783040894402

ENSMUST00000024736 1554 Sgol1 0.18190597329852565

ENSMUST00000023289 1185 Chkb 0.2587474233441136

ENSMUST00000025483 1680 Nars 0.25249240321752464

ENSMUST00000022589 1932 Enox1 0.25340449949418886

ENSMUST00000026599 798 Apool 0.16228202826379343

ENSMUST00000022501 2475 Gucy1b2 0.25064550907291516

ENSMUST00000026427 3279 Esyt1 0.26631387600741063

ENSMUST00000022821 840 Dhrs4 0.23687479595335267

ENSMUST00000022310 1956 Ngly1 0.17165798535504537

ENSMUST00000026240 735 Fgf8 0.3724783682590783

ENSMUST00000029147 1380 Nfs1 0.2508749181364346

ENSMUST00000023029 2112 L3mbtl2 0.3145760163565946

ENSMUST00000027404 2946 Ptprn 0.28211324998412857

ENSMUST00000025093 1587 Thumpd2 0.19164039171189926

ENSMUST00000029476 648 Sec22b 0.24321772273189704

ENSMUST00000029165 390 1110008F13Rik 0.2673813133774996

ENSMUST00000026021 1377 Msr1 0.17388789395578047

ENSMUST00000025779 384 Cdk2ap2 0.2898400936703799

ENSMUST00000026092 381 3110040N11Rik 0.2421381417344042

ENSMUST00000025186 1431 Slc39a7 0.26691822764447465

ENSMUST00000024059 477 Sva 0.15554761031581732

ENSMUST00000022666 2256 Klhl1 0.19301437973129817

ENSMUST00000028918 471 Scp2d1 0.2469974486962652

ENSMUST00000026985 405 Cplx2 0.46215104907411436

ENSMUST00000029650 1386 Ints12 0.1780049379502592

ENSMUST00000028487 921 Tfpi 0.20471027993669194

ENSMUST00000029023 411 1700021F07Rik 0.2798811298835325

ENSMUST00000027189 1443 Casp8 0.2675474779485698

ENSMUST00000029464 1062 Hao2 0.20826194648360394

ENSMUST00000022377 2460 Txndc16 0.1804241197637207

ENSMUST00000024747 441 Vegfa 0.3567567587820101

ENSMUST00000023535 1797 Iqcb1 0.1351705993488637

ENSMUST00000023851 351 Ndufa5 0.2186645376588307

ENSMUST00000021997 1458 Rsl1 0.18891607084969175

ENSMUST00000028034 1431 Nsun6 0.15376699193125873

ENSMUST00000029800 807 Tspan5 0.22137865598049503

ENSMUST00000028312 582 Lcn12 0.2937937394462012

ENSMUST00000022819 1887 Jph4 0.3116098157055231

ENSMUST00000028917 630 Dtd1 0.34084790483437255

ENSMUST00000023820 399 Fabp2 0.22136151540203042

ENSMUST00000027766 1938 Adck3 0.34092518915108194

ENSMUST00000023550 1554 Pdia5 0.3062156373755168

ENSMUST00000022693 2976 Bmp1 0.3459474663019088

ENSMUST00000029773 993 Cnn3 0.25144467315673336

ENSMUST00000026072 744 Trub1 0.23249681036164976

ENSMUST00000027906 1377 Esrrg 0.26348643270204375

ENSMUST00000028228 336 Cutal 0.2477399624511636

ENSMUST00000026663 1089 Cbx8 0.284716197437077

ENSMUST00000026858 2778 Plk4 0.16076537625692097

ENSMUST00000023514 390 Ndufb4 0.23716908612364257

ENSMUST00000025078 1404 Map3k8 0.21897138725967916

ENSMUST00000025912 3705 Plcb3 0.33478308785114164

ENSMUST00000022746 2916 Osmr 0.19124255637570545

ENSMUST00000028846 957 Dusp2 0.31800765023336564

ENSMUST00000028914 951 Polr3f 0.19083213695301962

ENSMUST00000022095 1548 Irx4 0.30508143253994724

ENSMUST00000026649 675 Syngr2 0.3759730243257764

ENSMUST00000023076 642 Nfam1 0.2663068449786138

ENSMUST00000028600 3579 Hipk3 0.19168096255185024

ENSMUST00000029125 849 Armc1 0.16866863113947075

ENSMUST00000023334 1650 Pvrl3 0.150154517273672

ENSMUST00000024959 306 Cript 0.2214086280290953

ENSMUST00000026816 1116 Wdr73 0.2215131158433928

ENSMUST00000023598 1095 Rfc4 0.15429583701723393

ENSMUST00000025804 606 Rab1b 0.3409068711172125

ENSMUST00000023629 2028 Pros1 0.1959807701603702

ENSMUST00000026448 2640 Faap100 0.33020593728937403

ENSMUST00000029784 1398 Celf3 0.3508228756153261

ENSMUST00000025095 336 Cox7a2l 0.26654478763329087

ENSMUST00000025089 2685 Map4k3 0.18370359708866837

ENSMUST00000025571 1485 Cd5 0.3143830215466684

ENSMUST00000022378 1395 Ero1l 0.21114008571631707

ENSMUST00000023761 174 Cox14 0.30139936873881623

ENSMUST00000027888 3549 Abl2 0.2519182705556651

ENSMUST00000027874 1860 Sele 0.23673644677101216

ENSMUST00000028123 1758 Gad2 0.22488940407457322

ENSMUST00000023673 1398 Runx1 0.3300785687639315

ENSMUST00000028469 1824 Slc43a1 0.2884092521619429

ENSMUST00000025682 1203 Lipn 0.1796842985358148

ENSMUST00000025925 489 Pla2g16 0.2870484386960687

ENSMUST00000027488 2001 Capn10 0.2934782444507082

ENSMUST00000023189 1641 Glyr1 0.24478858295204345

ENSMUST00000025054 978 Spdef 0.32957184139811

ENSMUST00000027252 3651 Eif5b 0.18289974603625117

ENSMUST00000027396 2529 Abcb6 0.26880241077251593

ENSMUST00000028857 2076 Nphp1 0.22443435596991218

ENSMUST00000024708 1968 Tnfrsf21 0.31258273458873026

ENSMUST00000026937 507 Iscu 0.3006601795805775

ENSMUST00000022304 1428 Thrb 0.26955402828367026

ENSMUST00000027961 1530 Hspa14 0.18270644023362198

ENSMUST00000029355 1083 Ppm1l 0.2829069189697081

ENSMUST00000023250 405 Ly6i 0.25276850214904684

ENSMUST00000023464 2217 Mfi2 0.3321467309909227

ENSMUST00000022325 414 9230112D13Rik 0.18348793537489813

ENSMUST00000023260 384 Lypd2 0.2876663525158818

ENSMUST00000023157 1968 Anks3 0.26476442384852394

ENSMUST00000025183 1221 Ring1 0.2998672137240816

ENSMUST00000026013 1581 Maoa 0.19290919645895963

ENSMUST00000027059 1380 Tfap2b 0.2366645262375537

ENSMUST00000028190 3372 Abl1 0.32419883739956534

ENSMUST00000029531 414 Lce1b 0.35470320323166193

ENSMUST00000024906 669 Twsg1 0.35912827545489273

ENSMUST00000025522 3300 Pdgfrb 0.31281343421020463

ENSMUST00000027748 606 Rgs16 0.3056795307407015

ENSMUST00000029423 1233 Serpini1 0.20116404756981018

ENSMUST00000022220 3285 Msh3 0.18463226060652022

ENSMUST00000027493 4152 Pask 0.24557085055503683

ENSMUST00000023684 3033 Gart 0.2126020862298981

ENSMUST00000027164 672 Ctla4 0.2254743830216588

ENSMUST00000022561 1908 Amer2 0.28861503133395283

ENSMUST00000023129 504 Gtsf1 0.2363142722430858

ENSMUST00000024974 783 Hagh 0.3102263038354718

ENSMUST00000029303 2598 Naa15 0.15693559198365806

ENSMUST00000028552 900 Katnbl1 0.12638297272527294

ENSMUST00000024702 822 Paqr4 0.312023026374971

ENSMUST00000029562 1506 Chrnb2 0.3256648236695784

ENSMUST00000027449 381 Nppc 0.3095963273473241

ENSMUST00000028623 2157 Ext2 0.3257806481751944

ENSMUST00000027785 2154 Sdccag8 0.23342544933392925

ENSMUST00000029368 1266 Ccna1 0.23506307487951217

ENSMUST00000029708 849 Apoa1bp 0.26861248537211196

ENSMUST00000029548 5646 Nup210l 0.20908111244925057

ENSMUST00000024159 999 Dlx2 0.3223694242223675

ENSMUST00000024860 1608 Ehd3 0.34987538514716743

ENSMUST00000022663 1146 Tnfrsf10b 0.23089016183731564

ENSMUST00000029502 1503 Slc16a4 0.17126139402483462

ENSMUST00000024044 1374 Cd4 0.29103701035424556

ENSMUST00000029786 798 Mrpl9 0.25461627642884793

ENSMUST00000026439 639 Nabp2 0.2877902103784297

ENSMUST00000028509 1356 Gorasp2 0.2245439319737421

ENSMUST00000025453 1404 Smad2 0.19529645407957835

ENSMUST00000023566 3210 Tmprss15 0.15928845881884554

ENSMUST00000026266 1491 Adpgk 0.2481177535634892

ENSMUST00000027581 1371 Gpr39 0.3142272530179326

ENSMUST00000028787 909 Gfra4 0.292826626322598

ENSMUST00000029540 3174 Npr1 0.2847939675793623

ENSMUST00000023244 1932 Rhpn1 0.3595039918631761

ENSMUST00000028426 1155 Cers6 0.22795466092494412

ENSMUST00000025842 375 Gal 0.2748153349801113

ENSMUST00000028536 831 Arl14ep 0.20009405840896183

ENSMUST00000026211 1485 Cyp2c44 0.24934897459005234

ENSMUST00000026565 414 Ifitm3 0.2908348513403765

ENSMUST00000026139 2013 Lrrc45 0.34208899674702237

ENSMUST00000027852 4845 Adcy10 0.23869363104010485

ENSMUST00000029524 402 Lce1d 0.3360617344250435

ENSMUST00000026559 774 Sirt3 0.2722699867107189

ENSMUST00000029698 378 Lamtor2 0.2765826149556777

ENSMUST00000026866 2067 Sclt1 0.1529636062748795

ENSMUST00000022317 657 Fam213a 0.2439397638692328

ENSMUST00000025515 1371 Tmx3 0.14460387060703814

ENSMUST00000024882 2544 Ttc27 0.19359389119255144

ENSMUST00000023059 1200 Dscc1 0.21628611081592186

ENSMUST00000023835 1047 Slc10a2 0.19856155668438374

ENSMUST00000025019 678 Arhgdig 0.35320073162250587

ENSMUST00000022337 2580 Cdhr1 0.2814049550897028

ENSMUST00000023867 1050 Rfc2 0.3311552793983838

ENSMUST00000025395 441 Grp 0.2397538814709594

ENSMUST00000025695 1494 Ppp2r5b 0.3231556732030529

ENSMUST00000025411 519 Slmo1 0.3124308762268681

ENSMUST00000023391 621 Mrpl40 0.29007385371418504

ENSMUST00000022894 738 Ywhaz 0.2137289478477371

ENSMUST00000027989 1005 Hsd17b7 0.22946950911246047

ENSMUST00000029446 2397 Csde1 0.16639814769165595

ENSMUST00000024260 1212 Pcbp4 0.3020353276040695

ENSMUST00000025635 630 1700017D01Rik 0.1398305473717604

ENSMUST00000029575 993 Extl2 0.2526245594976921

ENSMUST00000026986 321 Higd2a 0.24815898190246016

ENSMUST00000023720 1812 Krt84 0.3004310668629858

ENSMUST00000025381 243 Spink1 0.18444614428312106

ENSMUST00000027149 1200 Lancl1 0.27369938819163775

ENSMUST00000025581 678 Ms4a4d 0.14280957293592667

ENSMUST00000025691 984 Fas 0.1940078235523595

ENSMUST00000025773 324 Pold4 0.3622791050066224

ENSMUST00000023468 1524 Spag6 0.2162834737730489

ENSMUST00000025797 735 Mbl2 0.21771894663138786

ENSMUST00000023050 1509 Tab1 0.3197170707127715

ENSMUST00000028081 1593 Plxdc2 0.20374444372916514

ENSMUST00000029885 1602 Cpne3 0.19637414430792302

ENSMUST00000023210 978 Cyc1 0.25783879483991673

ENSMUST00000025390 486 Dynap 0.20543010048734348

ENSMUST00000026039 1575 Gk 0.16374955958040055

ENSMUST00000024946 870 Eci1 0.2998086694669119

ENSMUST00000029090 687 Gid8 0.22805381447105566

ENSMUST00000026120 1068 Bhlhe22 0.3255477761572303

ENSMUST00000026896 1128 St8sia2 0.29651580490012985

ENSMUST00000029024 585 Rab22a 0.2064036464663632

ENSMUST00000026459 1590 Atp5b 0.2179584510770682

ENSMUST00000026221 1077 Scd2 0.3425324784532461

ENSMUST00000023666 1719 Chaf1b 0.2554542387393871

ENSMUST00000022532 897 4930578I06Rik 0.32886902392931266

ENSMUST00000023538 5853 Mylk 0.31779526755181253

ENSMUST00000026990 1056 Thoc3 0.30315885942112836

ENSMUST00000023611 2793 Adamts5 0.25609341847530587

ENSMUST00000022618 2208 Adam2 0.2128585582176046

ENSMUST00000028740 3939 Ttbk2 0.19052897188978765

ENSMUST00000028948 591 Gins1 0.16915726117053212

ENSMUST00000028004 1557 Aldh9a1 0.2584752118614702

ENSMUST00000029453 1581 Vangl1 0.26142343010894603

ENSMUST00000026289 786 Hsd17b10 0.19503315221399897

ENSMUST00000023882 3867 Sympk 0.352449820249348

ENSMUST00000027623 687 Tsn 0.2030738695840277

ENSMUST00000025002 1344 Tekt4 0.33420744621334053

ENSMUST00000026613 1947 Gusb 0.2814428000673871

ENSMUST00000029686 2340 Hcn3 0.31333121901188243

ENSMUST00000027675 2316 Pigr 0.2691309325244714

ENSMUST00000024727 2409 Cdc5l 0.19174406329273233

ENSMUST00000026476 3018 Mbd6 0.258985507726535

ENSMUST00000022952 831 Osr2 0.30016405141080454

ENSMUST00000025224 1194 Gfra3 0.3051637745836367

ENSMUST00000023589 1986 Kng1 0.2052963362048905

ENSMUST00000026553 990 Syce1 0.2758172857473263

ENSMUST00000029569 981 Slc35a3 0.12226299435242731

ENSMUST00000023601 1212 St6gal1 0.3024026207997445

ENSMUST00000023707 465 Sod1 0.20646141131943838

ENSMUST00000028103 576 Lypd6b 0.2669399939568569

ENSMUST00000027373 1359 Ppm1f 0.28073560536202097

ENSMUST00000023105 1365 Endou 0.29593276592871093

ENSMUST00000024833 1362 Tmprss3 0.29900294229447544

ENSMUST00000025567 1335 Fads2 0.35790673569455406

ENSMUST00000025885 600 Sssca1 0.2752269042126463

ENSMUST00000028816 363 Tmem230 0.22135744930429122

ENSMUST00000025338 2883 Gabbr1 0.3019740556238224

ENSMUST00000029742 942 Nudt17 0.25636165507814457

ENSMUST00000027986 1755 Optn 0.3006978666077251

ENSMUST00000028955 1530 Angpt4 0.35413487117016346

ENSMUST00000026377 1995 Padi3 0.3645821874714411

ENSMUST00000028607 2124 Caprin1 0.18551211977412502

ENSMUST00000029515 297 S100a11 0.2542628535803557

ENSMUST00000023788 1662 Krt6a 0.3442718700367643

ENSMUST00000025160 2601 Ccdc178 0.1812517506257761

ENSMUST00000022256 1170 Psmd6 0.21177113537549663

ENSMUST00000026845 636 Il6 0.175419048591027

ENSMUST00000028902 387 Otor 0.21110324981325448

ENSMUST00000025425 7545 Cep192 0.168439946324909

ENSMUST00000022826 879 Fitm1 0.2877596499168015

ENSMUST00000029712 2400 Ntrk1 0.3294947829030896

ENSMUST00000026225 2514 Sema4g 0.2950034912138104

ENSMUST00000026703 2220 Bach1 0.2496270373692272

ENSMUST00000023400 669 Tmem114 0.3053699528367749

ENSMUST00000025590 2418 Osbp 0.26244585233276063

ENSMUST00000028135 2250 Fam129b 0.37588931137034454

ENSMUST00000027277 2247 Stat4 0.19409701434465262

ENSMUST00000029611 1194 Lef1 0.27748897566126474

ENSMUST00000024958 4296 Caskin1 0.29512100921983875

ENSMUST00000027035 1260 Sox17 0.31423317649159205

ENSMUST00000022858 1626 Agxt2 0.20630012035174786

ENSMUST00000022341 735 2700060E02Rik 0.1743288075309732

ENSMUST00000023807 717 Igfbp6 0.25828817727054554

ENSMUST00000028749 2466 Capn3 0.3059214433962923

ENSMUST00000022836 741 Mcpt1 0.20285574511390406

ENSMUST00000026831 1122 Rhbdl1 0.3677807285360001

ENSMUST00000027684 561 Arl8a 0.3100763393229627

ENSMUST00000025166 2721 Cdh2 0.21335037677542337

ENSMUST00000025439 1770 Me2 0.17830261325295993

ENSMUST00000022428 447 Rnase4 0.3131108688714353

ENSMUST00000028933 393 8030411F24Rik 0.19441891376372702

ENSMUST00000029839 1335 Spata1 0.12328133343522767

ENSMUST00000026585 717 Tspan4 0.31877187713634847

ENSMUST00000024873 741 Yipf4 0.13090333527368855

ENSMUST00000026325 1290 4933436I01Rik 0.19738255140376174

ENSMUST00000028084 1389 Nr5a1 0.372571362478804

ENSMUST00000029670 1101 Ptgfr 0.22287623863709677

ENSMUST00000022909 1338 Dcaf13 0.19451164009090027

ENSMUST00000023454 2619 Dlg1 0.17213866706302772

ENSMUST00000027279 597 Nabp1 0.175657670365109

ENSMUST00000025811 882 Yif1a 0.27319743331115465

ENSMUST00000029766 2463 Bcar3 0.3231208735420952

ENSMUST00000026239 1722 Poll 0.3042230507044822

ENSMUST00000026154 1086 Zdhhc16 0.31097654183830503

ENSMUST00000026881 483 Ly96 0.16273536802734262

ENSMUST00000028656 963 Duoxa2 0.2705126876025097

ENSMUST00000028139 936 Med27 0.2756075363609761

ENSMUST00000024035 1473 Gabrr2 0.347930275122248

ENSMUST00000025379 1713 Dpysl3 0.2985837431764101

ENSMUST00000028644 801 Cd82 0.35732621909349715

ENSMUST00000029017 1869 Pck1 0.35159515356858934

ENSMUST00000026562 405 Ifitm5 0.3100382376224075

ENSMUST00000029623 2355 Tlr2 0.21641856653967761

ENSMUST00000023295 1311 Acr 0.25475011671634235

ENSMUST00000022075 2262 Pcsk1 0.19920099536291397

ENSMUST00000022806 582 Bcl2l2 0.2928307310502188

ENSMUST00000029653 3654 Egf 0.21872683918324776

ENSMUST00000023608 327 Atp5j 0.20089401240649754

ENSMUST00000025835 2322 Cpt1a 0.30874570175191346

ENSMUST00000029256 1197 Sec62 0.20369627475625335

ENSMUST00000023779 1806 Nr4a1 0.29742993148431074

ENSMUST00000025270 1560 Riok3 0.18555023066487994

ENSMUST00000024779 2058 Usp49 0.3027197534315811

ENSMUST00000027247 723 Pdcl3 0.2988799838080268

ENSMUST00000025142 2532 Esco1 0.15087752506612398

ENSMUST00000028302 1209 Lhx3 0.3613969633212585

ENSMUST00000028888 2121 Tgm6 0.3287483473000324

ENSMUST00000028850 855 Kcnip3 0.30847803520788336

ENSMUST00000022028 552 1700067P10Rik 0.23897976778273344

ENSMUST00000027615 2010 F13b 0.1738648609470988

ENSMUST00000023259 351 Lynx1 0.40017067189826794

ENSMUST00000027186 2742 Trak2 0.23260222359161467

ENSMUST00000024797 1158 Mocs1 0.35071031038326045

ENSMUST00000025419 639 Ppic 0.2496023034222158

ENSMUST00000025241 2352 Ercc3 0.2862113456856311

ENSMUST00000028466 669 Prg3 0.2856717646007502

ENSMUST00000022708 702 Trim52 0.26039824751477675

ENSMUST00000028335 2817 Grin1 0.3601239046847775

ENSMUST00000023071 1410 Samm50 0.24431079765815775

ENSMUST00000023357 525 Fopnl 0.14688182118368664

ENSMUST00000024636 1140 Fgfr1op 0.17561012081465135

ENSMUST00000023756 1887 Racgap1 0.2597051595910082

ENSMUST00000024121 2727 4921501E09Rik 0.19684394637629196

ENSMUST00000022195 978 Otp 0.37263650543483773

ENSMUST00000025711 2349 Vps51 0.32850140013058293

ENSMUST00000023741 16767 Kmt2d 0.2727415360184832

ENSMUST00000022100 1860 Slc6a3 0.3383586530884946

ENSMUST00000025486 1767 Lmnb1 0.30850324268435564

ENSMUST00000026043 3609 Slk 0.19524757896080625

ENSMUST00000022786 1626 4931414P19Rik 0.28873436712804657

ENSMUST00000026134 462 Myadml2 0.2808696546783505

ENSMUST00000026846 924 Tyms 0.24783785503511693

ENSMUST00000029435 1626 Dclre1b 0.21152488280646647

ENSMUST00000022716 2169 Rnf219 0.21561161098855372

ENSMUST00000023507 1263 Gsk3b 0.20283988446128734

ENSMUST00000029346 1455 Schip1 0.2857719084430629

ENSMUST00000024047 1050 Twf2 0.3314598420406841

ENSMUST00000023056 1194 Deptor 0.2863973679133717

ENSMUST00000029645 1221 Tdo2 0.21107986931141276

ENSMUST00000025290 957 Impact 0.2279585155371662

ENSMUST00000025903 2322 Rps6ka4 0.34858841177776406

ENSMUST00000025463 663 Gm16286 0.3601377131869816

ENSMUST00000024698 390 Tnfrsf12a 0.2684472181632374

ENSMUST00000026295 588 Tsr2 0.2720209837487729

ENSMUST00000027339 1323 Smap1 0.21321298289572635

ENSMUST00000027315 5532 Zfp142 0.27543982204983114

ENSMUST00000027432 2862 Psmd1 0.19043455224212238

ENSMUST00000028821 1308 Fsip1 0.19674407754035048

ENSMUST00000024056 816 Ube2j2 0.20632813140527215

ENSMUST00000026432 2367 Cdh9 0.18769109609783344

ENSMUST00000027067 666 Gsta3 0.27010773629077345

ENSMUST00000027357 1371 Rnf25 0.2347297005090466

ENSMUST00000029143 2331 Fam83c 0.2890808974352945

ENSMUST00000026405 378 Bloc1s1 0.3726586692970166

ENSMUST00000028020 1473 Myoc 0.2896950877230534

ENSMUST00000026008 1656 Trim8 0.3998925875959491

ENSMUST00000027127 3915 Sf3b1 0.16058817530150338

ENSMUST00000022275 1881 Ankrd55 0.2638727045812815

ENSMUST00000024731 810 Spats1 0.23902262220215179

ENSMUST00000025404 654 Cidea 0.28006983053180184

ENSMUST00000028985 837 Bpifa1 0.1870430268260026

ENSMUST00000025989 1764 Tm9sf3 0.17213575529193015

ENSMUST00000026407 717 Cd63 0.2663026709322542

ENSMUST00000029722 795 Rps3a1 0.22782320279676524

ENSMUST00000028767 2148 Rtf1 0.22630552128337234

ENSMUST00000023128 3162 Itga5 0.2867383660365315

ENSMUST00000023619 312 Stfa2 0.1688109815906547

ENSMUST00000023612 1407 Ets2 0.3086392923863471

ENSMUST00000026081 1449 Pnliprp2 0.25524474188399787

ENSMUST00000027478 1068 Ndufa10 0.28386979045974586

ENSMUST00000029041 399 Fabp4 0.2875011438101222

ENSMUST00000028346 960 Fam166a 0.3056852338417858

ENSMUST00000028704 1113 Hao1 0.20767515502565517

ENSMUST00000022574 1917 Lrrc63 0.1504828674084322

ENSMUST00000023677 642 Atp5o 0.26542205806172753

ENSMUST00000025444 1983 Cxxc1 0.3198764261258209

ENSMUST00000026569 1164 Drd4 0.33161400603775054

ENSMUST00000023797 1578 Krt4 0.3194621743257018

ENSMUST00000025921 2232 Mark2 0.30031697847647576

ENSMUST00000024155 879 Kcnk16 0.33211191617542174

ENSMUST00000028853 294 Mal 0.2713720723445461

ENSMUST00000029730 2703 Chd1l 0.25078597600506636

ENSMUST00000029499 2175 Slc6a17 0.3907389286408235

ENSMUST00000024829 2001 Abcg1 0.35398656344617674

ENSMUST00000029307 864 Stoml3 0.25183673274333196

ENSMUST00000026410 2112 Dnajc14 0.23862631468234488

ENSMUST00000024970 1407 Gtf2a1l 0.22316010656988178

ENSMUST00000028755 1626 Ehd4 0.3563091957897841

ENSMUST00000023749 714 Tmbim6 0.2906770764807496

ENSMUST00000023161 2733 Srl 0.2502737418733794

ENSMUST00000028603 1443 Fbxo3 0.22385112751494324

ENSMUST00000029700 2283 Sema4a 0.3131400641201416

ENSMUST00000021993 336 Uqcrb 0.18959231900894435

ENSMUST00000023441 1170 P2rx6 0.3025260233791235

ENSMUST00000026415 897 Cdk2 0.2519676049487199

ENSMUST00000029900 1053 Atp6v0d2 0.21914236764615388

ENSMUST00000025800 1620 Pip5k1b 0.23460321258723743

ENSMUST00000027810 1524 Fh1 0.19475957919334108

ENSMUST00000026267 1893 Parp6 0.26780053850836083

ENSMUST00000028921 2856 Xrn2 0.17182109218312433

ENSMUST00000029741 1602 Polr3c 0.2655854926911567

ENSMUST00000024880 1953 Vit 0.25154996337140995

ENSMUST00000024123 2733 Agap3 0.34750531769723386

ENSMUST00000025077 1758 Mtpap 0.1762080539183272

ENSMUST00000028610 1584 Cat 0.2704460942388191

ENSMUST00000028233 5043 Hc 0.18982943970103014

ENSMUST00000027730 675 Myog 0.356834238052899

ENSMUST00000029387 1746 Eif2a 0.15847840562439913

ENSMUST00000025767 993 Aip 0.3387227092157592

ENSMUST00000026504 606 Xrcc6bp1 0.24808311426281646

ENSMUST00000028295 534 Dnlz 0.25391053449513

ENSMUST00000023531 1461 Hcls1 0.2839735162348645

ENSMUST00000023774 1707 Slc11a2 0.2719163600348338

ENSMUST00000025847 1095 Fibp 0.32585146704508894

ENSMUST00000024724 732 Crisp2 0.16297216163577283

ENSMUST00000025602 1068 Gna14 0.2837170707929855

ENSMUST00000025639 1281 Ccdc86 0.24050899744904927

ENSMUST00000027083 1641 Pth2r 0.257578025946965

ENSMUST00000024794 489 Tspo2 0.2999889233168822

ENSMUST00000027744 1026 Mr1 0.22358874827780598

ENSMUST00000027781 354 Cox20 0.16454881908581534

ENSMUST00000022182 1110 F2rl2 0.24223471658888004

ENSMUST00000022224 1257 Trappc13 0.16522714995839674

ENSMUST00000022089 408 Med10 0.27301370045642004

ENSMUST00000028665 1590 Patl2 0.2422280864057879

ENSMUST00000029630 1674 Fga 0.2178717272265751

ENSMUST00000026547 2718 Tubgcp2 0.30322461958564434

ENSMUST00000028806 1668 Smox 0.3349014669785966

ENSMUST00000023330 2853 Morc1 0.20471312714461623

ENSMUST00000028617 1515 Api5 0.1900203705537298

ENSMUST00000029155 2640 Epb4.1l1 0.34564969512716504

ENSMUST00000024249 1428 Prph 0.33054181441075

ENSMUST00000026135 1653 Aspscr1 0.29344259508975706

ENSMUST00000023712 2124 Krt2 0.2553260988231274

ENSMUST00000029624 1038 Ccdc109b 0.22994353533861356

ENSMUST00000022967 1512 Kcnv1 0.2429223731240912

ENSMUST00000028795 1020 Rad51 0.19010578178794232

ENSMUST00000026428 624 Myl6b 0.3420840328090334

ENSMUST00000029876 786 Calb1 0.2096892944286047

ENSMUST00000023360 585 Mpv17l 0.2932257120818261

ENSMUST00000023088 1248 Naga 0.3160385971079267

ENSMUST00000027965 396 Fam107b 0.23772574274540878

ENSMUST00000027752 4824 Lamc1 0.3025570430689993

ENSMUST00000025482 3756 Atp8b1 0.24822786345247042

ENSMUST00000029780 1599 Ptbp2 0.15933894351300945

ENSMUST00000024486 534 Mrps23 0.2416953080520276

ENSMUST00000023006 1422 Lrrc6 0.1895284551613103

ENSMUST00000027271 1191 Inpp1 0.250224208453734

ENSMUST00000028843 5589 Trpm7 0.14138441763481935

ENSMUST00000027297 666 Plekhb2 0.3031156890342618

ENSMUST00000029551 321 4933434E20Rik 0.1905979867915664

ENSMUST00000023482 1035 B4galt4 0.2837680770763446

ENSMUST00000022235 1266 Htr1a 0.3266565891829936

ENSMUST00000025010 2310 Tmem8 0.3164309461643062

ENSMUST00000028113 1149 4931423N10Rik 0.18989482429294163

ENSMUST00000022585 1041 Gpalpp1 0.19863022197891425

ENSMUST00000027171 1965 Carf 0.16002466477530833

ENSMUST00000022867 684 Laptm4b 0.28131018550707193

ENSMUST00000025618 5634 Pcsk5 0.2925577047183935

ENSMUST00000022040 3657 Agtpbp1 0.18729272249948345

ENSMUST00000026328 825 Prdx4 0.21075028804653406

ENSMUST00000029817 738 Bdh2 0.23005797064947084

ENSMUST00000029588 1713 Larp7 0.21494918308705538

ENSMUST00000025468 2583 Pde6a 0.3351068518213832

ENSMUST00000026446 549 Cnpy2 0.24815198314300996

ENSMUST00000022470 2886 Parg 0.18458235880844248

ENSMUST00000029447 624 Sike1 0.24448038296824254

ENSMUST00000024711 2097 Adgrf4 0.20400595342720798

ENSMUST00000028964 1527 Rbck1 0.36995391231303665

ENSMUST00000026408 1218 Gdf11 0.32866724605119757

ENSMUST00000027455 1590 Alppl2 0.29781202249933647

ENSMUST00000027973 1179 Sephs1 0.21868330834351976

ENSMUST00000029316 831 Exosc8 0.17267118679362392

ENSMUST00000027265 1245 Osgepl1 0.13078206749019167

ENSMUST00000026121 465 Ppp1r27 0.2626440910892583

ENSMUST00000028599 2154 Cstf3 0.17721919338032108

ENSMUST00000025198 1545 Btnl2 0.2806005880388929

ENSMUST00000025966 1473 Cyp2c55 0.22198422949911045

ENSMUST00000024914 1710 Themis3 0.23189951904896894

ENSMUST00000028781 4287 Atrn 0.24345389439321743

ENSMUST00000029711 3903 Insrr 0.293393291273463

ENSMUST00000029105 1536 Zgpat 0.3191783570942343

ENSMUST00000028882 813 Il1a 0.23346398765784196

ENSMUST00000026888 2799 Taf3 0.2577309695231668

ENSMUST00000021971 921 Slc25a48 0.3164797020970402

ENSMUST00000023501 2352 Maats1 0.259346802153584

ENSMUST00000029603 2286 Prss12 0.22327018624547748

ENSMUST00000026999 1281 Smad7 0.30772735301628423

ENSMUST00000027217 447 1500015O10Rik 0.23727332138820026

ENSMUST00000023431 2055 Tbc1d23 0.2022070185181357

ENSMUST00000026324 1320 Acot9 0.1652604589163046

ENSMUST00000027381 912 Pecr 0.2427604240123344

ENSMUST00000029679 240 Cks1b 0.2787400469005006

ENSMUST00000023727 1359 Kansl2 0.21070260146293338

ENSMUST00000029881 1824 Mmp16 0.2041299068564998

ENSMUST00000025177 4314 Trappc8 0.16858791931457207

ENSMUST00000028430 318 Cyct 0.2058246891175906

ENSMUST00000028257 663 Gca 0.2475153537567347

ENSMUST00000028639 1434 Meis2 0.2244272832102259

ENSMUST00000025083 2892 Kif5b 0.1705994116260808

ENSMUST00000029132 600 Dnajc5b 0.22453181040380943

ENSMUST00000023171 849 Hes1 0.2777576441887072

ENSMUST00000026917 2772 Nrp1 0.25370502718959376

ENSMUST00000025262 921 Ltb 0.27773458761690234

ENSMUST00000024657 1494 Phf10 0.20440376952266212

ENSMUST00000022815 948 Ngdn 0.2409897575203208

ENSMUST00000026911 1068 Ccr1 0.2264938319354597

ENSMUST00000023590 1611 Hrg 0.2022262975401538

ENSMUST00000028350 684 Zmynd19 0.26286155804846545

ENSMUST00000028825 1290 Fam98b 0.16986375548667104

ENSMUST00000028817 786 Pcna 0.19370628561533157

ENSMUST00000023728 396 4930415O20Rik 0.23322685467779872

ENSMUST00000024805 1782 Cpne5 0.3344827044910614

ENSMUST00000025908 1197 Kcnk4 0.29755141541310587

ENSMUST00000023119 1269 Vdr 0.3655294942603089

ENSMUST00000028286 837 Agpat2 0.341801217143202

ENSMUST00000022476 1116 Glt8d1 0.17436772719679525

ENSMUST00000028735 3657 Jag1 0.29869316037683386

ENSMUST00000022296 582 Ube2e1 0.23517350710085713

ENSMUST00000023455 1566 Ppil2 0.28537785830462753

ENSMUST00000024766 681 Rrp36 0.3101298784300152

ENSMUST00000029610 945 Hadh 0.2930418317214533

ENSMUST00000024032 408 9530002B09Rik 0.18579927808746108

ENSMUST00000028944 2049 Acss1 0.2782752386416306

ENSMUST00000026744 510 Tmem89 0.2809874278419322

ENSMUST00000027521 2574 Agap1 0.32686826823765497

ENSMUST00000028087 918 Ppp6c 0.16895576413324226

ENSMUST00000026498 735 Crisp1 0.14917153580071113

ENSMUST00000022053 1299 Trip13 0.218389434101683

ENSMUST00000029194 2028 Skil 0.20830796044793035

ENSMUST00000029076 783 Car3 0.3110742857354781

ENSMUST00000023913 525 Wrb 0.2871776951346113

ENSMUST00000025109 3171 Sap130 0.2360543483563774

ENSMUST00000026486 1230 Katnal2 0.20693151952951636

ENSMUST00000025293 666 Ndfip1 0.20811491221937353

ENSMUST00000023133 516 Ppp1r1a 0.28151867119036805

ENSMUST00000024932 468 Atp6v0c 0.323742147439386

ENSMUST00000025668 1626 Atl3 0.17799046113395703

ENSMUST00000026461 1254 Prim1 0.2175401825025327

ENSMUST00000026737 708 Shisa5 0.29391119861865417

ENSMUST00000021990 1422 Ptdss1 0.26244040340790475

ENSMUST00000022164 939 Ankra2 0.20167227288290157

ENSMUST00000026956 1458 Pramel6 0.22603359524971492

ENSMUST00000024983 4395 Ift140 0.30798193132005247

ENSMUST00000029707 1248 Gpatch4 0.2749092557052953

ENSMUST00000028368 1425 Wdsub1 0.23025399353794837

ENSMUST00000026436 1608 Baiap2 0.3609986984213869

ENSMUST00000024936 933 Prss30 0.2724624736489195

ENSMUST00000022595 414 Rgcc 0.31237661805124967

ENSMUST00000025394 579 Sec11c 0.1942786929867858

ENSMUST00000022292 1437 Samd8 0.2177776389435795

ENSMUST00000023478 1287 Igsf11 0.23767836937575895

ENSMUST00000028080 813 Nebl 0.30049460003281026

ENSMUST00000029879 2256 Nbn 0.1621934018037671

ENSMUST00000029463 1122 Hsd3b6 0.24970573491063808

ENSMUST00000026027 2406 Taf5 0.19070512273691323

ENSMUST00000023241 420 Ly6h 0.32139381609650786

ENSMUST00000026893 2013 Tgm4 0.2937852794413351

ENSMUST00000026670 1299 Nptx1 0.36249387253241766

ENSMUST00000029699 1998 Lmna 0.3618885429000178

ENSMUST00000025358 2976 Lvrn 0.20081790368217453

ENSMUST00000025979 2388 Aldh18a1 0.2660186514043005

ENSMUST00000028076 3207 Mllt10 0.17482470737827616

ENSMUST00000025851 2217 Dpp3 0.3178784060016642

ENSMUST00000029429 1170 Wnt2b 0.3112495031244595

ENSMUST00000025273 408 Psors1c2 0.29515431572907536

ENSMUST00000027528 1773 Mlph 0.3087920082927643

ENSMUST00000028937 402 9230104L09Rik 0.22181969057459497

ENSMUST00000022281 3123 Skiv2l2 0.17930466064693124

ENSMUST00000025702 1014 Snx15 0.30143617987461924

ENSMUST00000024858 1653 Galnt14 0.29581493934119274

ENSMUST00000027534 1179 Ilkap 0.24448520498509438

ENSMUST00000026284 10746 Zfhx4 0.20853096828304546

ENSMUST00000027688 1242 Rassf5 0.297636676551691

ENSMUST00000027778 696 Mixl1 0.2739770643082212

ENSMUST00000022148 1692 Mccc2 0.23503947633103775

ENSMUST00000022536 1236 Ska3 0.18018941025904808

ENSMUST00000026142 2328 Maged1 0.2891717002969877

ENSMUST00000022424 738 Rnase10 0.2647980346323267

ENSMUST00000023732 1170 Wnt10b 0.32140621306402445

ENSMUST00000026472 1059 Inhbc 0.29902405773402646

ENSMUST00000028897 2169 Cpxm1 0.27955804877238827

ENSMUST00000022262 1368 Fezf2 0.3186808156459152

ENSMUST00000022728 1326 Gpr180 0.28406623569976175

ENSMUST00000025230 2283 C2 0.29310439582877024

ENSMUST00000024338 927 Fam132a 0.24976954964817524

ENSMUST00000026170 684 Ubtd1 0.35557142825441035

ENSMUST00000027980 2658 Mcm10 0.23436376178924234

ENSMUST00000026357 2235 Jph3 0.37509351817768105

ENSMUST00000028349 1305 Arrdc1 0.29817155749570035

ENSMUST00000028743 633 Snap23 0.21690544262317643

ENSMUST00000022459 924 Phf7 0.22693329379736904

ENSMUST00000022170 2334 Gfm2 0.20260708518211965

ENSMUST00000025778 3078 Gldc 0.2781781772086923

ENSMUST00000024993 840 Prss29 0.3219646905079513

ENSMUST00000025023 978 Luc7l 0.1997882461254898

ENSMUST00000024699 660 Cldn6 0.3211540256830289

ENSMUST00000026975 2562 Dnaaf5 0.3242153158743269

ENSMUST00000024851 1929 Ndc80 0.19623598155168093

ENSMUST00000023214 1497 Dgat1 0.281083958478

ENSMUST00000022613 1779 Esco2 0.15905118332321364

ENSMUST00000027050 1005 Cops5 0.19348108014551763

ENSMUST00000027231 2445 Slc9a2 0.2722334806807362

ENSMUST00000023510 1446 Umps 0.22973415995576435

ENSMUST00000025471 543 Il17b 0.35430855257888955

ENSMUST00000022875 1479 Ank 0.29777004934148155

ENSMUST00000029535 363 Lelp1 0.265214305536776

ENSMUST00000024823 555 Glo1 0.24568523074180496

ENSMUST00000022904 1149 Atp6v1c1 0.19194524441929312

ENSMUST00000029520 549 Lce1m 0.35507809944137625

ENSMUST00000022927 1908 Rad21 0.21346668865875454

ENSMUST00000022734 1515 Dnajc3 0.2165937119662728

ENSMUST00000026073 1017 Trub1 0.24232240574913344

ENSMUST00000027291 1857 Zap70 0.3861015896125079

ENSMUST00000023099 1515 Slc38a2 0.22421293021545513

ENSMUST00000022692 582 Sftpc 0.2890557555865295

ENSMUST00000022908 951 Slc25a32 0.16783436194446533

ENSMUST00000026500 2460 Avil 0.32256960235209636

ENSMUST00000023873 1914 Prmt5 0.26875002024957634

ENSMUST00000022637 1728 Ebf2 0.2474463509681073

ENSMUST00000022062 1995 Sdha 0.214851296343593

ENSMUST00000029649 939 Ctso 0.21944966272283514

ENSMUST00000027695 1662 Slc45a3 0.3159839466612986

ENSMUST00000028549 3453 Slc12a6 0.22453738330156991

ENSMUST00000026586 933 Chid1 0.25573406184694747

ENSMUST00000025127 981 Mapre2 0.250347601256626

ENSMUST00000022601 1131 Wbp4 0.19015286276829216

ENSMUST00000027377 816 Igfbp5 0.38446733315822973

ENSMUST00000023271 2952 Mroh4 0.3073242740994722

ENSMUST00000025827 1446 Minpp1 0.2805548249768934

ENSMUST00000027123 1722 Hspd1 0.1510131996120502

ENSMUST00000025062 342 Clps 0.3625939114238916

ENSMUST00000022099 1605 Lpcat1 0.2718695819649078

ENSMUST00000023487 4278 Arhgap31 0.26028486887621965

ENSMUST00000025998 687 Mxi1 0.2662078184937379

ENSMUST00000029002 540 Stmn2 0.35487472388537117

ENSMUST00000022751 789 Ttc33 0.21289709328018175

ENSMUST00000023803 1272 Krt18 0.3394856996625058

ENSMUST00000027331 681 Tm4sf20 0.1936142530327163

ENSMUST00000022697 651 Fgf17 0.40213778954464235

ENSMUST00000026902 699 Rassf3 0.3114455279190951

ENSMUST00000027667 2187 Pkp1 0.3723820354399877

ENSMUST00000022577 5190 Zc3h13 0.21144221904773589

ENSMUST00000024595 1656 Slc22a3 0.24057121420676564

ENSMUST00000027670 1608 Fcamr 0.2269265774977681

ENSMUST00000023291 2493 Mapk8ip2 0.28595730261577906

ENSMUST00000025375 3303 Tcerg1 0.20263097080397702

ENSMUST00000026578 372 Tmem80 0.25498583535130953

ENSMUST00000027505 723 Ing5 0.3182758746673936

ENSMUST00000027877 2382 Kifap3 0.20004430957939567

ENSMUST00000028511 792 Mtx2 0.1883410096043509

ENSMUST00000028771 1185 Nusap1 0.19717768628860202

ENSMUST00000027111 2184 Ndufs1 0.16349317823589737

ENSMUST00000029547 1113 Creb3l4 0.2525002805316366

ENSMUST00000028471 1380 Smtnl1 0.2658321595457387

ENSMUST00000026210 1374 Cpn1 0.31378241769838877

ENSMUST00000026016 468 Fundc1 0.16474661141053337

ENSMUST00000029563 3459 Adar 0.2603799145265774

ENSMUST00000029794 747 Them5 0.23671971241362583

ENSMUST00000022169 1611 Hexb 0.1857448891351611

ENSMUST00000027230 657 Pih1d3 0.16808170298339697

ENSMUST00000028721 2175 Tgm5 0.28989841791627097

ENSMUST00000027587 2172 Ccnt2 0.20722456446706575

ENSMUST00000022212 2049 Plk2 0.2386617405724568

ENSMUST00000022497 591 Spryd7 0.18960657876803214

ENSMUST00000029570 1473 Hiat1 0.17627841457658608

ENSMUST00000029402 1653 Slc33a1 0.16319269804814598

ENSMUST00000029013 1107 Rae1 0.252704082303166

ENSMUST00000027603 708 Rgs18 0.17099129849921638

ENSMUST00000029676 2595 Adam15 0.2774362814813802

ENSMUST00000028986 813 Bpifa5 0.21399888080795415

ENSMUST00000023709 1743 Krt5 0.34143009330332164

ENSMUST00000025081 3354 Zeb1 0.21184136089753586

ENSMUST00000026173 1035 Wdr45b 0.23334114844565473

ENSMUST00000023089 1080 Wbp2nl 0.2032277178398916

ENSMUST00000029053 1299 Ptpn1 0.3135675570671525

ENSMUST00000027020 2709 Gria4 0.18599308675372464

ENSMUST00000027470 1560 Chrng 0.3362258607674099

ENSMUST00000029214 1290 Actl6a 0.22665914129140388

ENSMUST00000029845 858 Ddah1 0.29775463305015176

ENSMUST00000027871 1119 Sell 0.25993752758842137

ENSMUST00000023504 1296 Nr1i2 0.33237144282725956

ENSMUST00000022793 4017 Acin1 0.23874696699963963

ENSMUST00000028609 762 Elf5 0.31868577210221627

ENSMUST00000023116 2343 Aco2 0.31185558675199926

ENSMUST00000028864 1323 Fbln7 0.3265159886175794

ENSMUST00000027243 1233 Il1r2 0.22175437855309943

ENSMUST00000029180 981 Rprd1b 0.21385606576887253

ENSMUST00000029480 1629 Prpf38b 0.23985835709951664

ENSMUST00000022781 342 Dad1 0.29554789493110745

ENSMUST00000023243 687 Gpihbp1 0.3005864599837665

ENSMUST00000023156 1713 Il1rap 0.2410011416426982

ENSMUST00000028278 933 Psmd14 0.16565497403088103

ENSMUST00000026617 1167 Phkg1 0.31608969228923683

ENSMUST00000028553 195 Nop10 0.2615588827081755

ENSMUST00000028378 1902 Galnt3 0.18831640662205

ENSMUST00000026828 483 Fam195a 0.29033535636834573

ENSMUST00000028648 1281 Syt13 0.35445725522748617

ENSMUST00000022808 909 Pabpn1 0.2531542735760713

ENSMUST00000025752 1803 Pola2 0.2728980154196065

ENSMUST00000026666 2862 Gaa 0.3402383814179186

ENSMUST00000027878 738 Prrx1 0.30812660824127786

ENSMUST00000026700 429 Map3k7cl 0.2765176147669112

ENSMUST00000027374 168 Tnp1 0.29323212165300033

ENSMUST00000028014 1683 Fmo4 0.19693414244966412

ENSMUST00000029694 2958 Arhgef2 0.2789533471558376

ENSMUST00000023312 1752 Alcam 0.21002914257907654

ENSMUST00000022032 1017 2210016F16Rik 0.2789726067834534

ENSMUST00000025696 684 Ak3 0.2640500965156608

ENSMUST00000022531 3129 Lats2 0.3302850765110722

ENSMUST00000026367 2349 Sp4 0.16271743313023565

ENSMUST00000022203 942 Dimt1 0.18465559051194663

ENSMUST00000027571 1290 Gin1 0.17365721970395273

ENSMUST00000027978 351 Ucma 0.3176790368212294

ENSMUST00000025906 1269 Esrra 0.340095727435348

ENSMUST00000028363 552 Il1f8 0.17016637662716697

ENSMUST00000029541 2004 Slc27a3 0.2861530551066034

ENSMUST00000025385 2208 Hsd17b4 0.1936960428056862

ENSMUST00000022765 651 Rab2b 0.24092032413747103

ENSMUST00000029252 1248 Lrrc34 0.15536773088492273

ENSMUST00000028661 657 4933406J08Rik 0.1712894596517936

ENSMUST00000025462 1053 Rbfa 0.2865869623194979

ENSMUST00000023532 2805 Ccdc14 0.1791757804437985

ENSMUST00000028551 552 Emc4 0.2745333131109937

ENSMUST00000029682 2871 Thbs3 0.29482296633528093

ENSMUST00000026624 1989 Tm9sf2 0.18977086004867685

ENSMUST00000029269 1317 Exosc9 0.17313961248780702

ENSMUST00000026029 702 Samt4 0.1545358595949846

ENSMUST00000023067 1134 Ribc2 0.2920571692670899

ENSMUST00000023040 924 Slc25a17 0.22131608584964985

ENSMUST00000029574 2220 Vcam1 0.1938018968515763

ENSMUST00000026296 2883 Fgd1 0.2896441603923825

ENSMUST00000026222 561 Ndufb8 0.31749869212299703

ENSMUST00000023829 480 Cdkn1a 0.2865602246132585

ENSMUST00000027477 1863 Ngef 0.36263566739606595

ENSMUST00000024811 942 Pim1 0.327152148312763

ENSMUST00000024492 984 Acbd4 0.35589546968491387

ENSMUST00000023599 1224 Eif4a2 0.1859627443900125

ENSMUST00000022322 1677 Glud1 0.25703733264357437

ENSMUST00000023444 2514 Lztr1 0.3020203497021234

ENSMUST00000027566 1167 Serpinb11 0.19308861206419936

ENSMUST00000023952 1473 Krt8 0.4064209710947385

ENSMUST00000024827 246 Tff3 0.2781125030571353

ENSMUST00000025930 3654 Smc3 0.1887009319964114

ENSMUST00000022153 1146 Ptcd2 0.20863371881605047

ENSMUST00000028728 5274 Ubr1 0.1885702655957583

ENSMUST00000026576 1014 Taldo1 0.30435130663709636

ENSMUST00000022555 1500 Cdadc1 0.1800380904470733

ENSMUST00000028844 1572 Sppl2a 0.1868889136318359

ENSMUST00000028854 462 Mal 0.28797444997309446

ENSMUST00000023465 2589 Top3b 0.2955455103294217

ENSMUST00000025834 1338 Peli3 0.30318799366396426

ENSMUST00000029057 2022 Nfatc2 0.332090186466946

ENSMUST00000029175 1608 Src 0.3706103421513514

ENSMUST00000022269 672 Oit1 0.2921698491284492

ENSMUST00000029071 789 Car13 0.19096957641856446

ENSMUST00000024078 837 March5 0.1584869616105199

ENSMUST00000027649 1683 Nr5a2 0.2446499170392814

ENSMUST00000024599 7452 Igf2r 0.26685292186734744

ENSMUST00000028829 1335 Spred1 0.17812629574476754

ENSMUST00000029344 1395 Mfsd1 0.2179808824505115

ENSMUST00000022059 2106 Ahrr 0.2650950433836447

ENSMUST00000023211 1143 Sharpin 0.2209894641580342

ENSMUST00000027795 483 Cnih3 0.3268745291816373

ENSMUST00000023600 4839 Robo1 0.21401685198274453

ENSMUST00000028311 1506 Traf2 0.30057269512382206

ENSMUST00000022600 1341 Mtrf1 0.1767331234444696

ENSMUST00000027861 606 Dpt 0.3409766016798246

ENSMUST00000028938 423 Cst3 0.32464141436971033

ENSMUST00000027487 2163 Rnpepl1 0.33585724230925135

ENSMUST00000022120 1671 Acot12 0.2329470337326121

ENSMUST00000026093 1467 Btbd1 0.1988658029767

ENSMUST00000026832 816 Jmjd8 0.26338873903656795

ENSMUST00000027125 729 Coq10b 0.1546431109834311

ENSMUST00000029489 657 Gstm4 0.313902943508444

ENSMUST00000022246 630 Fgf10 0.24546101547944096

ENSMUST00000027114 876 9430016H08Rik 0.21325438814903905

ENSMUST00000025197 2109 Tap2 0.30317708841841595

ENSMUST00000028283 558 Lcn4 0.18382776414470958

ENSMUST00000023268 1191 Arc 0.3727788819455163

ENSMUST00000024831 264 Tff1 0.36924626804794825

ENSMUST00000024840 2190 Arhgap28 0.23673671942272248

ENSMUST00000029542 3126 Ints3 0.2736308710131719

ENSMUST00000026987 537 Nop16 0.29733271092810143

ENSMUST00000029652 1038 Pdgfc 0.2135188987629725

ENSMUST00000027312 1518 Prim2 0.24755639454357595

ENSMUST00000024707 2283 Mep1a 0.2759211452668969

ENSMUST00000027056 4797 Prex2 0.20977209120305207

ENSMUST00000027102 504 4933402D24Rik 0.253083318322491

ENSMUST00000025574 804 Ms4a7 0.19949654448763454

ENSMUST00000022507 1572 Pspc1 0.19791445839692515

ENSMUST00000022230 462 Srek1ip1 0.25431928022576195

ENSMUST00000023580 3168 Usp25 0.18163215191472068

ENSMUST00000028166 1797 Nr4a2 0.2552278044401651

ENSMUST00000029510 1323 Chil6 0.22298926628730525

ENSMUST00000022680 762 Bin3 0.33121236029826956

ENSMUST00000025474 939 Napg 0.26277274890175656

ENSMUST00000029440 1221 Olfml3 0.2663884955983709

ENSMUST00000022416 1512 Anxa11 0.31204026281123265

ENSMUST00000023806 1578 Soat2 0.32894214266587946

ENSMUST00000025831 6303 Dock8 0.290386024173512

ENSMUST00000023450 1437 Serpind1 0.242982232888005

ENSMUST00000029738 1368 Gpr89 0.2758405368316635

ENSMUST00000025924 1146 AI846148 0.28379497190778985

ENSMUST00000026631 744 Fgf14 0.22818059169312938

ENSMUST00000022918 2337 Oxr1 0.17587998156530543

ENSMUST00000029717 1011 Cd1d1 0.25454774017035253

ENSMUST00000024894 1632 Cyp1b1 0.2978750156291096

ENSMUST00000022722 1467 Irg1 0.25063435215266305

ENSMUST00000023101 1644 Slc38a4 0.25739628262131686

ENSMUST00000026078 993 BC051665 0.2669748466694469

ENSMUST00000022496 1566 Kpna3 0.13576198530532638

ENSMUST00000028225 1515 Psmd5 0.18140841476774597

ENSMUST00000028916 2304 Sec23b 0.23219626904964488

ENSMUST00000022446 807 Eaf1 0.29394839158870184

ENSMUST00000022947 2814 Matn2 0.27291939364715834

ENSMUST00000027642 606 Nek7 0.13516459212715282

ENSMUST00000028928 2121 Gzf1 0.266846518711251

ENSMUST00000022418 372 Tmem254b 0.2518656307805045

ENSMUST00000025981 1788 Tctn3 0.23179183353205177

ENSMUST00000028179 945 Fcnb 0.26853979418222274

ENSMUST00000028624 1272 Gatm 0.24844634847760924

ENSMUST00000023015 1050 Wnt7b 0.3848540953439607

ENSMUST00000024972 1413 Meiob 0.15047935927909256

ENSMUST00000022985 1746 Klhl38 0.31608467646163774

ENSMUST00000025728 1401 Frmd8 0.34571016223702

ENSMUST00000022196 3318 Ap3b1 0.215870812684517

ENSMUST00000024762 831 Rsph9 0.3209482726453007

ENSMUST00000029049 699 Chmp4c 0.2845883171100603

ENSMUST00000023931 498 Ssxb2 0.21314257037665194

ENSMUST00000026564 321 Ifitm1 0.356974500303596

ENSMUST00000022009 504 Cetn3 0.17168091135502067

ENSMUST00000023776 3270 Slc4a8 0.3104409275125046

ENSMUST00000027358 1257 Bcs1l 0.23482310185694089

ENSMUST00000023060 1119 Npcd 0.36728450393826867

ENSMUST00000029507 543 Dram2 0.1340024033654066

ENSMUST00000023238 1464 Gsdmd 0.2738624198024871

ENSMUST00000022368 1302 Plau 0.2546797562301922

ENSMUST00000029871 2484 Cdh17 0.20180156756782047

ENSMUST00000029025 7482 Zdbf2 0.16079431689939866

ENSMUST00000022709 948 Spry2 0.2640260764694746

ENSMUST00000028307 1005 Fcna 0.2730851469417721

ENSMUST00000026288 1140 Ribc1 0.24710376015990135

ENSMUST00000029018 1236 Zbp1 0.27426696973320297

ENSMUST00000022353 1341 Mss51 0.1851604007617151

ENSMUST00000028232 1737 Phf19 0.28675836526136317

ENSMUST00000022591 945 Epsti1 0.2521321987303142

ENSMUST00000026265 1563 Bbs4 0.23190208684649924

ENSMUST00000026433 3300 Smarcc2 0.2910135335129185

ENSMUST00000027343 1395 Ogfrl1 0.2266650019133852

ENSMUST00000023583 1038 Ahsg 0.24142321377306636

ENSMUST00000029414 558 Ssr3 0.22574734070357433

ENSMUST00000022458 2187 Bap1 0.2790426619573607

ENSMUST00000026254 5586 Gbf1 0.2534478263065248

ENSMUST00000026760 546 Tmem47 0.24728271087208611

ENSMUST00000023513 843 Cyp2ab1 0.3344650285900711

ENSMUST00000028188 1380 Fibcd1 0.3268348466448532

ENSMUST00000029673 693 Efna3 0.3998657933426967

ENSMUST00000024887 1311 Ndufaf7 0.19610259887694903

ENSMUST00000022057 657 Tppp 0.3443905198337751

ENSMUST00000028465 1194 P2rx3 0.3101514385869901

ENSMUST00000023474 1077 Wdr53 0.20252625746936573

ENSMUST00000027068 1125 Tram1 0.2123964656479274

ENSMUST00000027264 1884 Asnsd1 0.1415724918652124

ENSMUST00000028156 993 Gfi1b 0.3691682499540809

ENSMUST00000026406 957 Rdh5 0.3012259982878283

ENSMUST00000028119 2598 Mastl 0.17634920857499495

ENSMUST00000028650 1011 Pex16 0.30590311795274283

ENSMUST00000028696 1299 Ddb2 0.24884243364568134

ENSMUST00000028982 1047 Sun5 0.2859704326655711

ENSMUST00000024926 969 Prss41 0.31228088742099935

ENSMUST00000022451 2442 Capn7 0.1621202654266145

ENSMUST00000026475 507 Ddit3 0.3139103686883775

ENSMUST00000028525 2604 Rapgef4 0.2174031829727292

ENSMUST00000028347 1212 Rbms1 0.24548105376868093

ENSMUST00000023176 2043 Zfp263 0.24524111514151323

ENSMUST00000025418 795 Psmg2 0.20637827600037262

ENSMUST00000022104 3369 Tert 0.29123577877811313

ENSMUST00000025276 1974 3110002H16Rik 0.25277537551796425

ENSMUST00000025204 369 Pfdn1 0.24327804691577976

ENSMUST00000027697 1356 Cdk18 0.35783484790454173

ENSMUST00000028635 1332 Cops2 0.1642182839522921

ENSMUST00000022030 972 Ccnh 0.17151431425143784

ENSMUST00000023437 2358 Adgrg7 0.166903877689494

ENSMUST00000026571 1374 Irf7 0.30344176451057936

ENSMUST00000027929 2388 Mark1 0.235389398588007

ENSMUST00000028170 2559 Ralgds 0.344656067443485

ENSMUST00000025554 291 Scgb1a1 0.260105692127556

ENSMUST00000026190 2274 Loxl4 0.3160705094424129

ENSMUST00000022656 1578 Bora 0.1830289095524931

ENSMUST00000025163 384 Pfdn6 0.27348750574897496

ENSMUST00000028341 2514 Anapc2 0.2766190797227398

ENSMUST00000027256 483 Mrpl30 0.24285448367924037

ENSMUST00000028017 2097 Mettl13 0.3118230988552439

ENSMUST00000022861 1572 Ugt3a1 0.17263798983944875

ENSMUST00000026554 357 Urah 0.3488542278261529

ENSMUST00000028398 624 Ube2e3 0.19789268954259592

ENSMUST00000025399 312 Pmaip1 0.20304800675540075

ENSMUST00000025234 2061 Epb4.1l4a 0.2184302418675241

ENSMUST00000023117 333 Phf5a 0.2967688397150639

ENSMUST00000026886 2859 Itih5 0.28338302833185786

ENSMUST00000022039 1182 Golm1 0.27416282143615217

ENSMUST00000028356 660 Cd302 0.2002349283513443

ENSMUST00000023736 1470 Lmbr1l 0.31745193660190774

ENSMUST00000028003 1149 Lmx1a 0.32750410127808877

ENSMUST00000028815 1947 Slc23a2 0.2580998112315248

ENSMUST00000025239 1767 Bin1 0.32522963234573044

ENSMUST00000022257 2604 Atxn7 0.21928391706839281

ENSMUST00000028100 2442 Epc1 0.21033628440306731

ENSMUST00000023840 330 Cxcl13 0.15381971689645402

ENSMUST00000028083 834 Psmb7 0.20155497316229717

ENSMUST00000026897 2133 Slco3a1 0.32785401904559486

ENSMUST00000022639 1632 Nefl 0.3869797950559046

ENSMUST00000029842 702 Bcl10 0.2055561578817652

ENSMUST00000026662 1560 Cbx2 0.32052000929425245

ENSMUST00000027673 531 Il20 0.19395342892784806

ENSMUST00000024717 2448 Tbc1d5 0.2256864448400975

ENSMUST00000027517 3663 Dgkd 0.2745349327892351

ENSMUST00000023786 1665 Krt6b 0.3431709101575636

ENSMUST00000023072 1098 Parvb 0.3277513599976912

ENSMUST00000023138 1227 Shisa9 0.31893703034266857

ENSMUST00000029270 1269 Ccna2 0.19349447274540127

ENSMUST00000023884 1278 Gm853 0.31918990702585187

ENSMUST00000026234 942 Kazald1 0.280618771458788

ENSMUST00000025120 2496 Elp2 0.211596308816068

ENSMUST00000029837 912 Uox 0.2862489047305618

ENSMUST00000026209 4743 Dnmbp 0.2833777876219813

ENSMUST00000029566 618 Efna1 0.3149873998111795

ENSMUST00000027867 1530 Ccdc181 0.21931175417223187

ENSMUST00000027940 1053 Tmem206 0.32493942967305117

ENSMUST00000025264 3993 Wdr33 0.21365745893108837

ENSMUST00000025862 4752 Smarca2 0.27347728956225537

ENSMUST00000022063 783 Ccdc127 0.1911691868249629

ENSMUST00000025918 1632 Stip1 0.2876617501026788

ENSMUST00000023713 1551 Krt82 0.380455479443526

ENSMUST00000025893 555 Arl2 0.39443417497320393

ENSMUST00000027741 2088 Xpr1 0.2234301606920122

ENSMUST00000022349 3375 Cfap70 0.21492106306500605

ENSMUST00000025805 483 Cnih2 0.36025271419594074

ENSMUST00000027740 1173 Lhx4 0.30299780686740235

ENSMUST00000025707 933 Zfpl1 0.2985778531675492

ENSMUST00000023095 1014 Sept3 0.3380603905435931

ENSMUST00000028949 1113 Nsfl1c 0.24661196687504375

ENSMUST00000024696 1848 Mmp25 0.26845064756598325

ENSMUST00000027494 1086 Ppp1r7 0.23105282255163825

ENSMUST00000029671 1269 Ifi44 0.2087499979988992

ENSMUST00000029084 1275 Ntsr1 0.3503658990746168

ENSMUST00000023151 2124 Bcl6 0.3157046316117618

ENSMUST00000022727 1068 Tgds 0.20263529665399393

ENSMUST00000027512 1824 Atg16l1 0.2290848400005767

ENSMUST00000022207 846 Elovl7 0.20367073917183537

ENSMUST00000028382 978 Nup35 0.15807362298047625

ENSMUST00000025170 1869 Wdr46 0.3026445065199863

ENSMUST00000028691 1605 Arfgap2 0.24768303109479795

ENSMUST00000025408 2409 Afg3l2 0.23349235749196753

ENSMUST00000023100 3393 Srebf2 0.30247570518280975

ENSMUST00000026557 336 Bet1l 0.2881758099798592

ENSMUST00000022036 2112 Slc28a3 0.22825204882919758

ENSMUST00000027974 1971 Atf6 0.20659742690904656

ENSMUST00000026572 570 Hras 0.3305845573166894

ENSMUST00000025632 1203 2410127L17Rik 0.19715621103906686

ENSMUST00000029126 2595 Itch 0.1687572964420738

ENSMUST00000024026 522 Cypt1 0.19427478355778263

ENSMUST00000025193 2397 Brd2 0.23105071240397843

ENSMUST00000025520 1914 Slc6a7 0.3372998677763874

ENSMUST00000022535 1809 Dcp1a 0.22706419765081381

ENSMUST00000025685 1269 Lipm 0.1971629725691054

ENSMUST00000022946 408 Hrsp12 0.19129358094272955

ENSMUST00000029462 1809 Tbx15 0.2585534251806761

ENSMUST00000023747 3972 Nckap5l 0.2993825085478779

ENSMUST00000028151 255 Dpm2 0.28790007149141267

ENSMUST00000026169 948 Ogfod3 0.2528233838334201

ENSMUST00000029587 792 Neurog2 0.3132965587941394

ENSMUST00000029759 1371 Mettl14 0.18400887852468434

ENSMUST00000029377 609 Tm4sf4 0.25986807090379843

ENSMUST00000029635 1863 Gucy1b3 0.24507492292686772

ENSMUST00000029754 1179 Hormad1 0.14493198975779675

ENSMUST00000022060 576 Pdcd6 0.26872766171869406

ENSMUST00000028403 873 Cybrd1 0.2756919290470064

ENSMUST00000023587 1167 Fetub 0.2720085633083331

ENSMUST00000027677 582 Csrp1 0.32727046375905317

ENSMUST00000024543 678 Hes7 0.2999538453084096

ENSMUST00000029602 2622 Ndst3 0.22592385004543655

ENSMUST00000025714 807 Rpp30 0.14629104131086818

ENSMUST00000027464 1002 Mrpl44 0.21218078889579436

ENSMUST00000023491 2337 Lrch3 0.19416185067829814

ENSMUST00000028911 2340 Csrp2bp 0.247173476155091

ENSMUST00000022145 189 Serf1 0.24657994240101355

ENSMUST00000022828 621 Emc9 0.27042407830535925

ENSMUST00000027146 1581 Ikzf2 0.26438866616673323

ENSMUST00000025050 507 Nudt3 0.3566001405061279

ENSMUST00000029752 2172 Tars2 0.24017088047262433

ENSMUST00000026900 2331 Hgs 0.3576134295321269

ENSMUST00000022897 399 4930447A16Rik 0.14977331729115304

ENSMUST00000025561 1041 Anxa1 0.21316081166470655

ENSMUST00000026833 2373 Wdr24 0.30421159717149415

ENSMUST00000028384 663 Dusp19 0.20342729988404804

ENSMUST00000028533 393 Fshb 0.2977814470405673

ENSMUST00000025117 1455 Celf4 0.3637826322245966

ENSMUST00000027249 1125 Chst10 0.27123263206274434

ENSMUST00000027727 1128 Adipor1 0.31664274935648246

ENSMUST00000025965 2466 Hells 0.13777595421481642

ENSMUST00000023826 372 Sncg 0.3206954710286994

ENSMUST00000027837 1566 Vangl2 0.33496149011910165

ENSMUST00000022614 627 Ccdc25 0.22497654085846294

ENSMUST00000029846 1140 Cyr61 0.29977210331991305

ENSMUST00000023762 1245 Cers5 0.2665119551734598

ENSMUST00000024773 1041 Prph2 0.38711865433249204

ENSMUST00000024987 2523 Telo2 0.30687114920950276

ENSMUST00000027655 1860 Ddx59 0.22116387283102243

ENSMUST00000027144 4503 Cps1 0.24398998235387442

ENSMUST00000028068 975 Ptf1a 0.273665409588564

ENSMUST00000025350 1269 Dcp2 0.2164837343203588

ENSMUST00000029075 921 Dok5 0.2470141001702098

ENSMUST00000028681 1857 F2 0.31128789850755373

ENSMUST00000025294 1743 Ttc39c 0.23908892736112283

ENSMUST00000026976 984 Get4 0.31871373622626903

ENSMUST00000027764 483 A530064D06Rik 0.24171606513903718

ENSMUST00000029178 1692 Ctnnbl1 0.3308387467085504

ENSMUST00000024738 915 Sult1c1 0.25163826796615024

ENSMUST00000024978 510 Nme3 0.32009817152919856

ENSMUST00000025951 738 Rbp4 0.3409052082020644

ENSMUST00000026820 1947 Slc28a1 0.3106009012281494

ENSMUST00000029259 2154 Mccc1 0.23615517249574206

ENSMUST00000029158 1155 Aar2 0.3684683161754044

ENSMUST00000022623 1551 Trim35 0.3308538607820162

ENSMUST00000026827 615 0610011F06Rik 0.3382258913233779

ENSMUST00000028369 324 Dapl1 0.20850831159677413

ENSMUST00000028668 786 Eif3j1 0.14649012009377105

ENSMUST00000026315 933 Dnase1l3 0.2804732944699479

ENSMUST00000025727 696 Trpd52l3 0.22502108952730326

ENSMUST00000029345 711 Il12a 0.30229145883723496

ENSMUST00000023449 783 Snap29 0.2575531916476292

ENSMUST00000027816 660 Mptx1 0.21126855324984614

ENSMUST00000025601 1161 Lpxn 0.29182868865397205

ENSMUST00000027966 1023 Fcgr2b 0.2506988290395377

ENSMUST00000022517 960 Cryl1 0.2937120286815799

ENSMUST00000023809 1707 Amhr2 0.27856584714577837

ENSMUST00000027866 1233 Blzf1 0.17314003707327152

ENSMUST00000029388 336 4930579G24Rik 0.18786408492819218

ENSMUST00000022098 309 Mrpl36 0.29424393876600263

ENSMUST00000022338 876 Rgr 0.3003246792370272

ENSMUST00000029665 780 Dkk2 0.2699557707660938

ENSMUST00000026068 2376 Vwa2 0.30237398098306034

ENSMUST00000022462 936 Oxnad1 0.20460676450254234

ENSMUST00000022081 759 Spata9 0.18706101144906315

ENSMUST00000029478 1422 Slc25a54 0.1822588250219847

ENSMUST00000024739 2175 Hsp90ab1 0.33130071370656144

ENSMUST00000024909 459 Ndufv2 0.15461109652192273

ENSMUST00000026546 2478 Adam8 0.28293949124203666

ENSMUST00000022340 4212 Nid2 0.2830416387105736

ENSMUST00000022264 4329 Ptprg 0.2602890352591705

ENSMUST00000026269 2007 Limd1 0.2760417029252881

ENSMUST00000027777 3045 Parp1 0.31917171685044865

ENSMUST00000027687 615 Ube2t 0.1931287080911059

ENSMUST00000026602 810 2010106E10Rik 0.17569887993691613

ENSMUST00000029441 1572 Syt10 0.21618892187249023

ENSMUST00000028517 1191 Ola1 0.1783943947183102

ENSMUST00000023562 795 Tmem41a 0.2952872212673664

ENSMUST00000028983 1389 Bpifb2 0.26357751267503154

ENSMUST00000023457 2250 Senp5 0.21758298334841752

ENSMUST00000024706 1323 Pla2g7 0.18397379458362265

ENSMUST00000029833 1902 Lrriq3 0.17700118093825307

ENSMUST00000022268 1080 Pdhb 0.19206726658033163

ENSMUST00000023978 537 Lcn9 0.2682901549952868

ENSMUST00000025861 804 Ovol1 0.37903250692992657

ENSMUST00000022142 4212 Naip1 0.2051386476312356

ENSMUST00000026466 351 Tac2 0.31131123183757964

ENSMUST00000027401 918 Stk16 0.2609412339458123

ENSMUST00000026635 3147 Farp1 0.31420684149816447

ENSMUST00000026608 447 Crcp 0.28493311019218776

ENSMUST00000022480 3090 Ogdhl 0.35344420966776663

ENSMUST00000029404 3774 Ppfia2 0.17345115719846918

ENSMUST00000025046 1191 Ip6k3 0.34009258362507255

ENSMUST00000022992 2991 Tbc1d31 0.19422911077714383

ENSMUST00000029530 450 Lce1a2 0.3414426394074273

ENSMUST00000029188 756 Wisp2 0.32727650789760404

ENSMUST00000022993 756 Derl1 0.24430768611459433

ENSMUST00000025209 618 Spata24 0.27226429630742144

ENSMUST00000022217 4587 Zfyve16 0.15797831644802832

ENSMUST00000027446 795 Cops7b 0.2622233019759084

ENSMUST00000027498 1281 Stk25 0.3460275204989496

ENSMUST00000023856 528 Msrb2 0.2669032911164609

ENSMUST00000029905 1512 Cyp7a1 0.19922708564737263

ENSMUST00000028796 1662 Rpusd2 0.27230394337546826

ENSMUST00000023818 2076 Calcoco1 0.3375122030655728

ENSMUST00000027580 1575 Slc35f5 0.17253699561115618

ENSMUST00000026568 1422 Ptdss2 0.35363019988372424

ENSMUST00000026452 264 Pde6g 0.3463523028812052

ENSMUST00000029273 441 Il21 0.20584503343570668

ENSMUST00000027592 1521 Ubxn4 0.18842720643895083

ENSMUST00000026383 1218 Gpr143 0.23964044285657493

ENSMUST00000023616 2223 Slc15a2 0.21243524051483226

ENSMUST00000024701 1473 Pkmyt1 0.30859306825789906

ENSMUST00000025311 3543 Pcdh12 0.23997857273783466

ENSMUST00000028848 942 Fahd2a 0.28163763031781486

ENSMUST00000029875 774 Tmem55a 0.1905285700023895

ENSMUST00000026425 1185 Pa2g4 0.22281174895163336

ENSMUST00000026704 1647 Cct8 0.16821910914054428

ENSMUST00000025218 1314 Etf1 0.1441785262935592

ENSMUST00000028010 1605 Fmo3 0.2507865431841183

ENSMUST00000029326 954 Sucnr1 0.24018737650176378

ENSMUST00000022610 1476 Scara5 0.3268913360357992

ENSMUST00000023085 396 Ndufa6 0.2791483933422984

ENSMUST00000027952 5685 Plxna2 0.29834431880087525

ENSMUST00000025523 2934 Csf1r 0.3073964453623926

ENSMUST00000026150 1485 Arhgap19 0.22056501467884512

ENSMUST00000026050 723 Gsto1 0.2787839884331602

ENSMUST00000028881 810 Il1b 0.2774060289429171

ENSMUST00000022242 993 Emb 0.18732723972696588

ENSMUST00000025503 894 Isoc1 0.24421296846604076

ENSMUST00000029644 993 Ppa2 0.23257155015742353

ENSMUST00000026445 1479 Fscn2 0.32658453859897585

ENSMUST00000029639 1338 Snx7 0.24101540231172042

ENSMUST00000025161 1401 Tapbp 0.3078475810592757

ENSMUST00000029196 1611 Slc2a10 0.27893532269338767

ENSMUST00000022691 3549 Hr 0.27518029809459493

ENSMUST00000023282 858 Miox 0.3593254332926746

ENSMUST00000027846 1008 Tada1 0.2572187839942324

ENSMUST00000028160 1545 Slc25a25 0.32134031466926205

ENSMUST00000022699 1395 Gfra2 0.3486789192676668

ENSMUST00000023364 456 Pla2g10 0.2952074218252122

ENSMUST00000029266 960 Anxa5 0.250239719031129

ENSMUST00000028763 2643 Tyro3 0.3028142915755365

ENSMUST00000022698 1239 Dok2 0.29353945138893556

ENSMUST00000022545 627 Fgf9 0.20736149635674353

ENSMUST00000022681 1050 Pdlim2 0.3305531994731808

ENSMUST00000025014 774 Mrpl28 0.35696637645911117

ENSMUST00000029374 8811 Nbea 0.18907894240951736

ENSMUST00000023559 2157 Ehhadh 0.20418279565345157

ENSMUST00000027226 1050 Khdrbs2 0.19845449220292807

ENSMUST00000029222 2814 Ccdc39 0.17932300470845292

ENSMUST00000028199 1011 Tor1b 0.33639850838798296

ENSMUST00000028907 1992 Bfsp1 0.2892515636397709

ENSMUST00000029047 1035 Snx16 0.15643302582994417

ENSMUST00000022953 4212 Fam135b 0.20839082311725665

ENSMUST00000026899 864 Slc25a10 0.33221444276146966

ENSMUST00000026199 789 Cutc 0.16107949865629503

ENSMUST00000027802 963 Pycr2 0.35449328565070237

ENSMUST00000023294 672 Rabl2 0.30452804293976954

ENSMUST00000022830 1461 Ripk3 0.24620516071642498

ENSMUST00000026699 2208 Mkln1 0.19195417993670877

ENSMUST00000028999 231 Pkia 0.15646441488562401

ENSMUST00000022108 1071 Hapln1 0.24090592330079783

ENSMUST00000025830 2529 Apba1 0.3428187669204919

ENSMUST00000025986 3039 Tll2 0.3134279726355272

ENSMUST00000028178 1482 Acvr1c 0.23092690562879697

ENSMUST00000027368 1647 Slc11a1 0.30672035168620587

ENSMUST00000027693 615 Rab29 0.3137164936164194

ENSMUST00000027193 315 Ndufb3 0.18028590514923107

ENSMUST00000028630 3273 Slc12a1 0.23010541553139563

ENSMUST00000027809 1203 Opn3 0.2944885572644573

ENSMUST00000022227 921 Cenpk 0.1195162583702624

ENSMUST00000025178 2172 Vps52 0.3084034689298357

ENSMUST00000022023 2589 Trpc7 0.31080616017581836

ENSMUST00000029087 1902 Ogfr 0.32861840706857903

ENSMUST00000026469 1578 Nab2 0.2824689103602895

ENSMUST00000028835 912 Crls1 0.18052165332045209

ENSMUST00000027151 567 Myl1 0.29459318988565114

ENSMUST00000028200 1002 Tor1a 0.34959208386510554

ENSMUST00000029804 1161 Metap1 0.20601610073793197

ENSMUST00000025890 2421 Scyl1 0.31305528487580897

ENSMUST00000027049 1074 Ppp1r42 0.14404756555561304

ENSMUST00000022580 876 Slc25a30 0.2289354761051639

ENSMUST00000025759 513 Eif1ad 0.31063506570538507

ENSMUST00000028499 3135 Itgav 0.2158424828558622

ENSMUST00000028337 996 Lrrc26 0.2205051116404253

ENSMUST00000024594 1137 Agpat4 0.33086244158564304

ENSMUST00000027738 1563 Tor1aip1 0.2352878695014829

ENSMUST00000023435 822 Tmem45a 0.18082463399631948

ENSMUST00000023064 384 Cby1 0.26259014895178107

ENSMUST00000026595 801 Tmem192 0.21827036900121188

ENSMUST00000023524 711 Rabl3 0.17256252493898536

ENSMUST00000023953 792 Ceacam14 0.15479438655813502

ENSMUST00000026927 657 Nudt5 0.225133217219466

ENSMUST00000025434 2793 Slc14a2 0.30395839909218103

ENSMUST00000024954 2625 Epas1 0.34572204524368266

ENSMUST00000022579 663 Erich6b 0.2054350082133956

ENSMUST00000029358 1512 Nmd3 0.1735749770795636

ENSMUST00000027859 1341 Tbx19 0.27192274269287187

ENSMUST00000026817 366 Nmb 0.3200015912020681

ENSMUST00000029770 1980 Abcd3 0.1808440620657448

ENSMUST00000026972 1740 Fam20c 0.32654991377410275

ENSMUST00000023570 759 Btg3 0.21326017771942757

ENSMUST00000028155 3483 Tsc1 0.24634518170760228

ENSMUST00000025961 774 Prdx3 0.19536847660951748

ENSMUST00000022286 528 Ndufs4 0.21263387140523515

ENSMUST00000029812 2916 Nfkb1 0.2514170706078682

ENSMUST00000022640 2370 Adam7 0.18412593869402188

ENSMUST00000025583 597 Ms4a2 0.1584146316060456

ENSMUST00000028470 273 Timm10 0.3982610878903768

ENSMUST00000023336 1809 Cd96 0.19333479657023098

ENSMUST00000022522 1122 Tdh 0.2648051193695384

ENSMUST00000022590 450 Dnajc15 0.16958408397228644

ENSMUST00000025076 447 Lyzl1 0.26222298883611633

ENSMUST00000025279 3834 Npc1 0.2673694193135388

ENSMUST00000024238 9906 Celsr3 0.2720363706112777

ENSMUST00000022855 1062 Brix1 0.17084473689615862

ENSMUST00000025223 1464 Cyp21a1 0.33396451907713876

ENSMUST00000023682 1683 Donson 0.20627362895584125

ENSMUST00000025420 1149 Ptpn2 0.15752997163033075

ENSMUST00000028577 1458 4930430A15Rik 0.1573486030551028

ENSMUST00000025642 1515 Prpf19 0.2765674842015626

ENSMUST00000026126 2895 Ints4 0.1890357076570288

ENSMUST00000027288 1896 Cnga3 0.31192307496150856

ENSMUST00000028288 7596 Notch1 0.3475066807505692

ENSMUST00000024869 1845 Spast 0.1791776452567457

ENSMUST00000026494 1044 Rnf165 0.32804237825859883

ENSMUST00000022391 3984 Ktn1 0.17292253736058016

ENSMUST00000026159 633 Cd7 0.28513078254920937

ENSMUST00000027287 2820 Inpp4a 0.2929389433042877

ENSMUST00000022890 2523 Rnf19a 0.18520830622150042

ENSMUST00000024931 1743 Tbc1d24 0.2985284167329179

ENSMUST00000029662 3696 Alpk1 0.21565688092372753

ENSMUST00000023691 1056 Il10rb 0.2536085555649228

ENSMUST00000026462 954 Hsd17b6 0.23768208570358015

ENSMUST00000026485 780 Hdhd2 0.21405561163821682

ENSMUST00000023134 456 Glycam1 0.23513779066478355

ENSMUST00000025897 2466 Map4k2 0.31864469583114025

ENSMUST00000028250 789 Mrrf 0.2167131818692849

ENSMUST00000025872 1044 4430402I18Rik 0.18347370475726682

ENSMUST00000025647 1164 Pga5 0.2825297442450489

ENSMUST00000022213 2892 Thbs4 0.297158129741328

ENSMUST00000022398 885 1700011H14Rik 0.2114547552078622

ENSMUST00000027322 948 Rhbdd1 0.2645892683053948

ENSMUST00000028522 3222 Itga6 0.20216120114048144

ENSMUST00000029456 1035 Cd2 0.24407932412827776

ENSMUST00000028392 2382 Dnajc10 0.16584943931372503

ENSMUST00000028892 1155 Idh3b 0.29072016238511417

ENSMUST00000025732 867 Slc25a45 0.27629617532811634

ENSMUST00000028299 714 1700019N19Rik 0.3131366736659395

ENSMUST00000025940 1002 C1qtnf9 0.23203238558570488

ENSMUST00000025944 816 Hhex 0.2977678574431087

ENSMUST00000026390 1476 Rabgef1 0.30463088902597274

ENSMUST00000028768 993 Ndufaf1 0.17843989800285887

ENSMUST00000027896 1158 Nphs2 0.2310346348815078

ENSMUST00000029806 843 Dapp1 0.20856474412411186

ENSMUST00000025955 4035 Eif3a 0.17981133915270403

ENSMUST00000023660 459 Ripply3 0.22668313570313023

ENSMUST00000027575 2052 Slco6d1 0.12415298035630075

ENSMUST00000023886 891 Sult1c2 0.25784125904650423

ENSMUST00000026084 1554 Slc18a2 0.26501051219341065

ENSMUST00000029546 441 Jtb 0.19997678464080046

ENSMUST00000025667 714 Rtn3 0.23559879689045432

ENSMUST00000022529 1872 Tkt 0.33854969743413804

ENSMUST00000024031 1227 Acy1 0.314744971187165

ENSMUST00000027908 1140 Spata17 0.2204900039708731

ENSMUST00000025999 2121 Add3 0.24801863931287785

ENSMUST00000026012 1524 Cyp17a1 0.2613831330488936

ENSMUST00000025815 1182 Cbwd1 0.1415919302417812

ENSMUST00000026561 213 Cox8b 0.2362260715263996

ENSMUST00000022701 2766 Rb1 0.15991302596638207

ENSMUST00000028494 726 Phospho2 0.14423500600680528

ENSMUST00000023019 1254 Trmu 0.27913147742301475

ENSMUST00000024200 852 Cela3a 0.30647778346632043

ENSMUST00000029825 2415 Depdc1a 0.11725308670198065

ENSMUST00000022282 630 Gpx8 0.21517356161605192

ENSMUST00000029014 714 Rbm38 0.3677903102794624

ENSMUST00000026665 1656 Cbx4 0.34348390651557703

ENSMUST00000025249 573 Apom 0.26619587768034764

ENSMUST00000035201 2961 Rbm6 0.19111799762389406

ENSMUST00000036111 963 Mrps35 0.21895419773526667

ENSMUST00000037012 1965 Kcna4 0.25760543861029767

ENSMUST00000031422 1698 Anapc7 0.2754985201345944

ENSMUST00000033602 954 Tnmd 0.20569521809812444

ENSMUST00000030432 978 Hmgcl 0.27067004426391267

ENSMUST00000048824 354 Gm5617 0.23076924003334784

ENSMUST00000034443 2355 Cdh15 0.32018787044536295

ENSMUST00000035773 3999 Sos2 0.1794215733723746

ENSMUST00000042092 2292 Cdh26 0.2340725849162717

ENSMUST00000039784 1275 Acaa1a 0.303633253393978

ENSMUST00000031852 1767 4921507P07Rik 0.2012679228581419

ENSMUST00000050092 4437 Zfp518a 0.13732020849303128

ENSMUST00000041272 3225 Plekhm1 0.30100013855106816

ENSMUST00000047119 552 Crygn 0.39269619403936457

ENSMUST00000041723 1899 Zdhhc17 0.19241810977880267

ENSMUST00000045912 3357 Rptn 0.21149069218873273

ENSMUST00000045503 1707 Ppp1r16b 0.3427728601587509

ENSMUST00000031601 2241 Zkscan5 0.28582104727303126

ENSMUST00000033127 1395 Sergef 0.24009725554805872

ENSMUST00000037418 981 Tmed8 0.26135102719588726

ENSMUST00000033056 582 Pycard 0.27014586976563415

ENSMUST00000040860 753 Psma8 0.1803153896276042

ENSMUST00000038463 2022 Raver2 0.21575140312519867

ENSMUST00000042296 978 Qk 0.19783364122969652

ENSMUST00000041375 2796 Sik2 0.2284720363832846

ENSMUST00000039994 7305 Cep250 0.29569952501947516

ENSMUST00000035625 1524 Cyp7b1 0.16197437483191937

ENSMUST00000047510 2577 Usp5 0.3063615152893396

ENSMUST00000037165 837 Lyl1 0.33152055073206643

ENSMUST00000034992 1731 Nt5e 0.23052498093361745

ENSMUST00000035548 6435 Alpk2 0.22689630949074563

ENSMUST00000045743 816 Prkab2 0.2573416350137424

ENSMUST00000032315 918 Tas2r116 0.17498119295221526

ENSMUST00000030280 1368 Angptl3 0.17687226705876047

ENSMUST00000031530 1155 Sppl3 0.23220456672449852

ENSMUST00000031354 2289 Abcb9 0.3923911638497425

ENSMUST00000041314 375 Paip2 0.2117198768485756

ENSMUST00000035077 2124 Ltf 0.2538550435930847

ENSMUST00000033824 1221 Lamp1 0.27947706825260266

ENSMUST00000045713 4515 Nacad 0.272805004279481

ENSMUST00000035532 1365 Pik3r1 0.2108116343758544

ENSMUST00000042471 2877 Dis3 0.18157862206215258

ENSMUST00000042405 984 Fbl 0.2638598909494638

ENSMUST00000034820 2295 Tle3 0.30775556507424723

ENSMUST00000033585 1254 Pgk2 0.1948402415514724

ENSMUST00000046687 2424 Spon1 0.3186093515523727

ENSMUST00000041133 1266 Fam110c 0.24762151597203283

ENSMUST00000040806 264 Dbil5 0.37780519238118426

ENSMUST00000035170 6732 Dnajc13 0.18177595363200547

ENSMUST00000031320 318 Pf4 0.26629869530424966

ENSMUST00000049793 1053 Duxbl1 0.1937451213320642

ENSMUST00000034766 1572 Rora 0.2624645091325294

ENSMUST00000045694 1089 Mettl18 0.1597352319903231

ENSMUST00000042658 903 Echdc3 0.2641436803255326

ENSMUST00000049040 762 Bri3bp 0.32060574550226345

ENSMUST00000033923 3279 Dlc1 0.3048463381202322

ENSMUST00000049819 972 Vmn1r179 0.18250203902328974

ENSMUST00000031741 1512 Cyp3a13 0.209678058426409

ENSMUST00000045810 1572 Zfp446 0.2573875186654026

ENSMUST00000039161 681 Thyn1 0.24083640554733007

ENSMUST00000047812 2283 Dpp4 0.19777230771909396

ENSMUST00000041859 1119 Gmds 0.22059029223853252

ENSMUST00000049483 3252 Fmnl2 0.20749956591057617

ENSMUST00000037007 6108 Evpl 0.379797061891743

ENSMUST00000032152 387 Prok2 0.2567311956455539

ENSMUST00000030090 993 Alad 0.2920224149618482

ENSMUST00000050038 936 Olfr1184 0.20176590876341063

ENSMUST00000030898 2142 Arhgef16 0.34226805975009794

ENSMUST00000038424 2847 Txndc11 0.2566271104825671

ENSMUST00000030660 543 Trappc3 0.3023189912999802

ENSMUST00000048485 96 Sln 0.2834931373879962

ENSMUST00000030585 1113 A3galt2 0.3035089516721745

ENSMUST00000030385 936 Ppcs 0.2388188328029316

ENSMUST00000049032 1593 Gmeb2 0.2671774927110319

ENSMUST00000044675 951 Jmjd7 0.30660529413486154

ENSMUST00000030456 2322 Nasp 0.16973249466018217

ENSMUST00000039229 2502 Cdcp1 0.29161703926899857

ENSMUST00000038661 957 Slc25a34 0.31580366111766056

ENSMUST00000041477 1287 Islr 0.2987520078956233

ENSMUST00000040531 2064 Pak4 0.33072811660707185

ENSMUST00000048187 444 Ppp1r14a 0.35130192835775564

ENSMUST00000033300 978 Art1 0.3053288047958333

ENSMUST00000032073 684 Nat8 0.24352286101922588

ENSMUST00000038404 1059 Ccno 0.33265393931159515

ENSMUST00000039537 1380 Fam63a 0.23757700393528527

ENSMUST00000043419 465 Eif5a 0.26393316215633456

ENSMUST00000041779 717 Clec4a2 0.19243636596537717

ENSMUST00000030610 1305 Zbtb8a 0.2774722178446807

ENSMUST00000038770 2022 Vasn 0.26795919794792417

ENSMUST00000041388 1209 Serpine1 0.31942415438935423

ENSMUST00000034960 2679 Dpp8 0.19216028381220995

ENSMUST00000041047 3078 Lnpep 0.17661218456382155

ENSMUST00000033800 834 Plp1 0.25369882148788303

ENSMUST00000030158 561 Dctn3 0.3120962409652764

ENSMUST00000048391 3948 Clspn 0.22519473786108588

ENSMUST00000039913 810 Tigar 0.21822749906380215

ENSMUST00000030427 2322 Tceb3 0.23551827590112157

ENSMUST00000032425 786 Emc3 0.26659125517969146

ENSMUST00000034879 1041 Hmg20a 0.24142563692673552

ENSMUST00000031017 981 Fosl2 0.3593952332924198

ENSMUST00000041138 1089 Elac1 0.2283022533247425

ENSMUST00000038580 1011 H2-M3 0.2753426410688582

ENSMUST00000030010 6786 Abca1 0.28129819814051166

ENSMUST00000035938 276 Ccl5 0.3043865338805246

ENSMUST00000037210 1008 Metap1d 0.1995064433515115

ENSMUST00000040519 579 Apopt1 0.23624145158171642

ENSMUST00000043138 3399 Inpp5f 0.20570919388767395

ENSMUST00000046433 1026 Rnf113a1 0.2744160506939915

ENSMUST00000041392 1269 Cylc2 0.18004404872061813

ENSMUST00000042614 13257 Gm15800 0.27434497427064086

ENSMUST00000039605 1005 Fam50b 0.47105537516649126

ENSMUST00000047311 1479 Mboat1 0.21781134387874715

ENSMUST00000047247 2325 Hectd2 0.15733767731174653

ENSMUST00000031653 4002 Flt1 0.24585114934230645

ENSMUST00000042373 1920 Shcbp1l 0.22215141195876079

ENSMUST00000033497 792 Pqbp1 0.2722821924404066

ENSMUST00000032440 969 Sec13 0.30158367460359536

ENSMUST00000034969 1701 Lctl 0.2696871274791623

ENSMUST00000041984 945 Akap7 0.21715039394984317

ENSMUST00000049389 1101 Zdhhc2 0.2081975905950021

ENSMUST00000035842 969 Rassf4 0.2619941149226747

ENSMUST00000041162 1248 1700017N19Rik 0.18367399229315928

ENSMUST00000034581 1254 Cadm1 0.255801189362874

ENSMUST00000033549 927 Asb12 0.1835139292447383

ENSMUST00000048782 693 Trem1 0.22296238376274535

ENSMUST00000030575 558 Tmem54 0.2781256537913268

ENSMUST00000036188 1791 Zc3h12a 0.30216124400803784

ENSMUST00000043577 657 Cldn5 0.30425024622200153

ENSMUST00000038207 2817 Dnajc6 0.2393333182017509

ENSMUST00000045986 789 Spr 0.33566903189929836

ENSMUST00000044125 1098 Nkx6-1 0.303221633522839

ENSMUST00000041331 1068 Scd1 0.3543311472231752

ENSMUST00000043584 1338 Tubb4b 0.35460492793125054

ENSMUST00000038765 1236 Inhbb 0.38517531652278364

ENSMUST00000047947 1083 Gmppb 0.27170327303803404

ENSMUST00000034299 747 Ifi30 0.3170176936907806

ENSMUST00000050149 2883 Mical2 0.3418700189062949

ENSMUST00000030134 1251 Rad23b 0.1981490248048629

ENSMUST00000044081 1629 Papd7 0.2536432290622326

ENSMUST00000041606 1059 Necab1 0.22441920935097243

ENSMUST00000049743 936 Olfr462 0.3209366299944409

ENSMUST00000041055 6228 Atg2b 0.21549347003537062

ENSMUST00000040560 975 Tsfm 0.28688554843495045

ENSMUST00000034339 2355 Cdh5 0.3284464765180103

ENSMUST00000031625 1113 Arpc1a 0.31055832458927163

ENSMUST00000040231 1761 Trp63 0.310217832719609

ENSMUST00000030797 312 Vamp3 0.23628967191194017

ENSMUST00000041674 1575 Btn1a1 0.2453587162770553

ENSMUST00000049178 1473 Cyp2c37 0.2032140414444754

ENSMUST00000040789 1089 Qpct 0.18627378105246303

ENSMUST00000048502 2007 Eml2 0.32985194270427326

ENSMUST00000035732 1473 Tekt3 0.26342007986541777

ENSMUST00000032887 654 Coq7 0.2505557045359056

ENSMUST00000030531 429 Pla2g2e 0.3759349126233442

ENSMUST00000038377 516 Btg1 0.3007768561056559

ENSMUST00000049382 1785 Gatad2b 0.2018062584364554

ENSMUST00000047873 2763 Arhgef1 0.3193194309904605

ENSMUST00000046587 708 Scamp5 0.3378540269472267

ENSMUST00000033965 8148 Tenm3 0.2519121067481201

ENSMUST00000048128 1710 Zbtb7a 0.37081900907698895

ENSMUST00000047334 1119 Kcnj1 0.20730204878885225

ENSMUST00000039925 1182 Upb1 0.2952341827425654

ENSMUST00000033012 2862 Copb1 0.16721779790886998

ENSMUST00000032895 1263 Nucb2 0.2294505275339709

ENSMUST00000040655 771 H2-Aa 0.27290404170314103

ENSMUST00000031051 978 Cgref1 0.24341894615434698

ENSMUST00000030627 1257 Rhd 0.30603673973731277

ENSMUST00000029938 951 Gtf2b 0.21426105068256734

ENSMUST00000035715 1008 Prss42 0.2479462020371961

ENSMUST00000030400 312 Mycbp 0.1773782157361171

ENSMUST00000032931 2295 Fchsd2 0.18562826422342732

ENSMUST00000049052 1632 9130019O22Rik 0.27578019940075393

ENSMUST00000041890 1113 Tm6sf1 0.1955800694307472

ENSMUST00000040667 1938 Zfp300 0.15840810346325016

ENSMUST00000034774 3567 Itga11 0.3438791608980433

ENSMUST00000048044 996 Trub2 0.2884792793568158

ENSMUST00000038053 1842 Lpp 0.2572619475261982

ENSMUST00000048209 999 Ldha 0.2793348881468238

ENSMUST00000034204 684 Nudt21 0.20602892774792403

ENSMUST00000042512 1176 Kin 0.21899574556040977

ENSMUST00000048914 279 Mrpl34 0.3499232009250011

ENSMUST00000042975 1062 Tmem30b 0.4185039695198999

ENSMUST00000041385 2013 Arhgap27 0.3299084344903902

ENSMUST00000038142 546 Isg20 0.3852448569882629

ENSMUST00000032491 1365 Tnfrsf1a 0.3081395560198077

ENSMUST00000049152 606 Snx10 0.22993938992828442

ENSMUST00000040188 765 Ska1 0.16760847232293494

ENSMUST00000046371 1359 Lppr2 0.32310941140811233

ENSMUST00000036865 3993 Fanci 0.21400277930883524

ENSMUST00000049997 1146 Prokr2 0.3065520237804395

ENSMUST00000032571 1479 Nova2 0.3490464419977666

ENSMUST00000041769 1479 Dync1li2 0.22253515369611865

ENSMUST00000033489 537 Praf2 0.2747090559082277

ENSMUST00000034409 735 Izumo1r 0.34549921321332866

ENSMUST00000033198 942 Crym 0.25609924699835623

ENSMUST00000032568 1896 Dmpk 0.3247885758430403

ENSMUST00000030897 4719 Megf6 0.33650680006773204

ENSMUST00000033662 1173 Pdha1 0.23855855961803685

ENSMUST00000037955 1185 Sdr42e1 0.2839988570949642

ENSMUST00000032949 1386 Coro1a 0.3256804682870406

ENSMUST00000032512 702 Klrb1a 0.16334606163396015

ENSMUST00000034065 3612 Nek1 0.16204416441584615

ENSMUST00000034230 1188 Cx3cl1 0.26253842047188763

ENSMUST00000040368 1233 Rmdn2 0.19649680644467446

ENSMUST00000030968 747 Pacrgl 0.18600995191796463

ENSMUST00000034277 624 Emc8 0.37314525757319805

ENSMUST00000039146 954 Tex19.2 0.27313335660512894

ENSMUST00000040159 1092 C1galt1 0.14498106419568402

ENSMUST00000035295 972 Degs1 0.26612313567309204

ENSMUST00000036999 3039 Clip2 0.3507257493995769

ENSMUST00000047045 1743 Acsm4 0.24086245160672246

ENSMUST00000030003 825 Exosc3 0.20860917423277167

ENSMUST00000046306 1260 Ikzf5 0.21627524318984237

ENSMUST00000030088 1461 Bspry 0.29156222868252785

ENSMUST00000039836 1800 Plk5 0.33679186867928274

ENSMUST00000048139 1599 Wrap53 0.2515173080064257

ENSMUST00000031985 1446 Mkrn1 0.263748797759112

ENSMUST00000034406 717 Ankrd49 0.1990428782473369

ENSMUST00000045450 2901 Ints7 0.2557731433912408

ENSMUST00000049572 1194 Lipa 0.21077153040261665

ENSMUST00000036247 657 Lrrc24 0.27128434541395774

ENSMUST00000034796 939 Elovl4 0.26962629421264445

ENSMUST00000044823 3567 Zswim5 0.26629067174033005

ENSMUST00000033054 555 Adm 0.27260627150542327

ENSMUST00000035899 558 Bloc1s5 0.2704936680958676

ENSMUST00000031709 1428 Fezf1 0.25484628784948626

ENSMUST00000036737 834 Colec10 0.2358501919857675

ENSMUST00000048289 1413 Egr2 0.2839327491253104

ENSMUST00000036937 1341 Trmt12 0.21557703690169105

ENSMUST00000034148 489 Il15 0.17252621217124517

ENSMUST00000031598 2625 Ddx54 0.34562367822839696

ENSMUST00000049956 1566 Lrrc24 0.2628608240217119

ENSMUST00000041097 1362 Ppp2r2d 0.21659902836093683

ENSMUST00000046497 522 Dnph1 0.2617532586138531

ENSMUST00000032373 1005 Ldhb 0.3266978071798097

ENSMUST00000033465 924 Vgll1 0.24562232527621863

ENSMUST00000042185 2121 Zfyve9 0.21112580361706543

ENSMUST00000031478 1920 Ddx51 0.2711173453936962

ENSMUST00000049009 1212 Rad9b 0.20142454070789081

ENSMUST00000032322 4842 Lrp6 0.20851010818048768

ENSMUST00000039419 7785 Igsf10 0.18517011553369514

ENSMUST00000034398 1503 Cep57 0.16484379800530072

ENSMUST00000030081 1440 Slc46a2 0.25112651294449095

ENSMUST00000048162 1284 Bsdc1 0.2786817118371596

ENSMUST00000030439 1701 Rnf220 0.2881869454937369

ENSMUST00000030261 336 2610528J11Rik 0.2923871497634454

ENSMUST00000043200 1287 Smap2 0.2432240138591613

ENSMUST00000046603 3918 Gak 0.25375078972660126

ENSMUST00000038275 1338 Mylip 0.3389588838521796

ENSMUST00000033100 1194 Izumo1 0.2096932038162245

ENSMUST00000042290 2853 Itih2 0.23844493812190798

ENSMUST00000033506 1458 Wdr13 0.30312290989574087

ENSMUST00000037875 2106 Rrbp1 0.32116804859512305

ENSMUST00000047206 4476 Plekhh2 0.22300796326676653

ENSMUST00000044384 1560 Aldh1b1 0.33517684795821645

ENSMUST00000040506 2556 Fam13b 0.15981506499202683

ENSMUST00000049289 1602 Ugt1a2 0.23333509661317664

ENSMUST00000040217 1011 Tbc1d21 0.3304115556525776

ENSMUST00000049642 684 Fahd1 0.2888168245366369

ENSMUST00000030696 924 Fam76a 0.30488822074207367

ENSMUST00000048192 837 Haus1 0.16099295341312145

ENSMUST00000040772 1998 Fermt3 0.361945192990274

ENSMUST00000033915 837 Gpm6a 0.27368297550057086

ENSMUST00000045402 3249 Slc38a10 0.30497770323999696

ENSMUST00000043675 312 Atp5l 0.22431665788555386

ENSMUST00000032898 3294 Ipo5 0.2083090125597751

ENSMUST00000035055 3378 Map4 0.21571081768085718

ENSMUST00000048572 1431 Hlx 0.2885560236630302

ENSMUST00000038217 1044 Dtx3 0.35438869522449096

ENSMUST00000045540 1740 Socs7 0.2597163371258101

ENSMUST00000032198 1107 Usp18 0.2759589694997185

ENSMUST00000037807 879 Vax2 0.3092375583163735

ENSMUST00000048121 2571 Myrip 0.3256824686074055

ENSMUST00000041654 1029 Fut7 0.30509063141427484

ENSMUST00000035092 891 Myd88 0.26763638206755713

ENSMUST00000043484 765 Reep2 0.3063412024419493

ENSMUST00000034983 837 Atp1b3 0.1796172594282793

ENSMUST00000033936 666 Dkk4 0.22444668967563203

ENSMUST00000031273 1386 Cds1 0.27123847076479896

ENSMUST00000032138 537 Cnbp 0.2647816021076681

ENSMUST00000034478 2568 St14 0.3448172725075262

ENSMUST00000043227 1596 Rcbtb1 0.21895631874249666

ENSMUST00000035177 1047 Mrpl3 0.20614730859640093

ENSMUST00000034831 1188 Nr2e3 0.3136562228488644

ENSMUST00000044664 930 Cysltr2 0.21230112483457325

ENSMUST00000044598 3120 Rpgr 0.1692626036303172

ENSMUST00000044462 1647 Tcam1 0.2690819397513417

ENSMUST00000034650 1947 Mcam 0.27326069495868344

ENSMUST00000030645 2103 Cnksr1 0.3016284783594549

ENSMUST00000036450 7293 Spg11 0.22489399953486539

ENSMUST00000030103 336 Tmem261 0.23040316987202394

ENSMUST00000035701 2079 Fshr 0.23239663789699075

ENSMUST00000049910 1065 Otc 0.18378493243517402

ENSMUST00000039540 828 March1 0.25630857566098

ENSMUST00000049298 1344 Tph1 0.22735602009548583

ENSMUST00000042070 1014 Zdhhc15 0.22968881031411623

ENSMUST00000033429 1968 Elf4 0.27044855288274877

ENSMUST00000038252 1269 B3galt2 0.16376296537877372

ENSMUST00000044798 1032 Kcnk15 0.3703534339326082

ENSMUST00000038867 2937 Opa1 0.18668825433017744

ENSMUST00000048002 3105 B4galnt4 0.30501237017456206

ENSMUST00000041783 2337 Iqce 0.29573147380579007

ENSMUST00000037391 714 Cd99l2 0.21853725207379734

ENSMUST00000030381 1776 Ctps 0.20577013696334495

ENSMUST00000034431 435 Tmem170 0.2737104537474292

ENSMUST00000037746 876 Tlx3 0.3199272220492028

ENSMUST00000040715 249 Mustn1 0.4031173521002129

ENSMUST00000036700 1398 Adra2a 0.3241643123383785

ENSMUST00000041543 1854 Vgf 0.2756263254251613

ENSMUST00000036355 3750 Phldb2 0.23620383176175408

ENSMUST00000047055 444 Lce1c 0.34724990181340193

ENSMUST00000038699 2424 Ap5z1 0.34944783476313274

ENSMUST00000039213 4059 Ibtk 0.18581014431184278

ENSMUST00000038488 2499 Sidt2 0.33016867166783836

ENSMUST00000045281 4257 Smg6 0.23790861882645634

ENSMUST00000046983 1884 Smpd1 0.26047932004841673

ENSMUST00000038720 1977 Ccdc114 0.317203677309167

ENSMUST00000037824 1206 Foxh1 0.29528062937110333

ENSMUST00000045776 1071 AI182371 0.17213302694109714

ENSMUST00000034543 1134 Rpusd4 0.24082502705821324

ENSMUST00000042391 1869 Fdxacb1 0.20095569592822543

ENSMUST00000031367 1725 Slc15a4 0.29633533765569

ENSMUST00000047951 894 Fbxo2 0.4055146579149047

ENSMUST00000045644 1827 Actr5 0.3269724944423529

ENSMUST00000040357 1239 Rcsd1 0.2758066678117856

ENSMUST00000030806 3453 Kif1b 0.21236212646912525

ENSMUST00000050129 249 Cox7b2 0.2149238808622848

ENSMUST00000050010 426 Vamp4 0.17482366044188102

ENSMUST00000030460 1422 Gpbp1l1 0.2043817052500207

ENSMUST00000040440 450 Calm2 0.24004598207227826

ENSMUST00000048678 2202 Lss 0.29060054373373423

ENSMUST00000048309 4437 Camsap2 0.20038161948636468

ENSMUST00000030170 5139 Unc13a 0.36887302705918085

ENSMUST00000030526 633 Pla2g2f 0.38392037021096737

ENSMUST00000038644 558 Rangrf 0.2630100863025181

ENSMUST00000044252 828 Nubp2 0.2904337734664054

ENSMUST00000039892 2229 Tbc1d25 0.24147988068455004

ENSMUST00000031249 1953 Sparcl1 0.2454034322961959

ENSMUST00000030816 996 Dffa 0.2938182642308383

ENSMUST00000043765 2580 Hnrnpul1 0.2894438226161362

ENSMUST00000034703 1827 Carm1 0.3068981732259428

ENSMUST00000034554 771 Pou2af1 0.289569726350297

ENSMUST00000042985 1080 Cebpa 0.3326780745385788

ENSMUST00000042452 816 Psmf1 0.2657505158968582

ENSMUST00000033643 498 Glt28d2 0.18235034168968262

ENSMUST00000043108 5592 Zgrf1 0.1691921222907581

ENSMUST00000042993 435 Grifin 0.3494053282729638

ENSMUST00000035276 513 Dctpp1 0.2556871783828114

ENSMUST00000032762 1554 Plin1 0.35457564382909335

ENSMUST00000044505 2121 Pde1c 0.2311865658330146

ENSMUST00000040844 3147 Ankrd27 0.2698518454866499

ENSMUST00000033283 2379 Rrm1 0.2281125362250335

ENSMUST00000039922 1455 Sufu 0.27356328898709625

ENSMUST00000033877 1263 Brf2 0.2700821247788658

ENSMUST00000034178 1686 Ces1f 0.2276515091869543

ENSMUST00000046897 1323 Trim14 0.31484611695208575

ENSMUST00000033769 2253 Irak1 0.2692934897025493

ENSMUST00000040987 978 Akr1d1 0.21293601154391045

ENSMUST00000033419 6222 Dock11 0.1679790182746791

ENSMUST00000030763 1410 Cdk14 0.22212199839662486

ENSMUST00000032141 1062 Hmces 0.28192549107722475

ENSMUST00000031160 1278 Paics 0.19241389288374855

ENSMUST00000037923 711 Rbm24 0.32258593000947294

ENSMUST00000043774 921 4933433C11Rik 0.3006103973108049

ENSMUST00000040381 1740 Ncoa5 0.2605716599030789

ENSMUST00000034197 1053 St3gal2 0.3591940191950248

ENSMUST00000046515 1227 Nceh1 0.280461407442817

ENSMUST00000037478 1566 Slc18a1 0.22416711050158258

ENSMUST00000037048 744 Mmd2 0.3617884753033921

ENSMUST00000031691 1446 Hyal4 0.1597433575088323

ENSMUST00000046663 2310 Dcbld2 0.2173321332710266

ENSMUST00000035371 2229 Sv2a 0.3056714627293255

ENSMUST00000032997 729 Lat 0.2938764944548259

ENSMUST00000041265 588 Lbx2 0.2533657941756896

ENSMUST00000048250 2151 Tab3 0.16363515197702308

ENSMUST00000046864 2544 Vav3 0.19104563968366436

ENSMUST00000034749 1095 Fam81a 0.2721366568575969

ENSMUST00000047040 783 Prkcdbp 0.29691702492326555

ENSMUST00000030606 720 Rcan3 0.3226216062566706

ENSMUST00000045096 3270 Ppp1r3a 0.1551453208464259

ENSMUST00000037286 864 C1ql2 0.32805357504537513

ENSMUST00000038676 1965 Cpz 0.34369783058306635

ENSMUST00000043380 2061 Catsper1 0.24806361622669634

ENSMUST00000031766 1686 Asns 0.23003786575208043

ENSMUST00000047768 1821 Neil3 0.2084984794261804

ENSMUST00000041048 753 Orai2 0.43289586465623175

ENSMUST00000040336 2070 Slc22a23 0.3021423318190986

ENSMUST00000031726 1023 Gnb2 0.355617907340552

ENSMUST00000041944 1590 Kcnj12 0.36528019316536126

ENSMUST00000030578 504 Ptp4a2 0.15700952316991715

ENSMUST00000033437 1797 Enox2 0.212207646851349

ENSMUST00000049999 774 Spin2c 0.23487457142776755

ENSMUST00000031227 1743 Zfp326 0.21085267539978897

ENSMUST00000039374 2586 Ddx26b 0.15754726056775326

ENSMUST00000033480 3351 Atp11c 0.14034047820089246

ENSMUST00000050273 192 Cox17 0.3034005499521842

ENSMUST00000049503 2631 Eml4 0.18408598312285068

ENSMUST00000032238 2568 Vmn2r26 0.16628868932791213

ENSMUST00000030905 585 Ssu72 0.2787696252229137

ENSMUST00000034000 1185 Asah1 0.1597170784908209

ENSMUST00000031931 744 2210010C04Rik 0.26283747454133954

ENSMUST00000037941 711 Cd81 0.3346482239117403

ENSMUST00000033096 1380 Nucb1 0.37311607989551787

ENSMUST00000034226 765 Fam192a 0.26164636872254415

ENSMUST00000034240 2475 Kifc3 0.3696280014092793

ENSMUST00000034285 429 Cotl1 0.37180738196065577

ENSMUST00000033341 1518 Tub 0.3210566876442649

ENSMUST00000043377 963 Sun3 0.1685118230668814

ENSMUST00000040274 399 Tnfrsf18 0.2804014786795269

ENSMUST00000049357 894 Pnrc1 0.23850402593346032

ENSMUST00000038830 468 1110059E24Rik 0.1996701788643743

ENSMUST00000043654 1356 Tubg2 0.37300514868740264

ENSMUST00000042666 1608 Slc39a5 0.25425675696616906

ENSMUST00000030865 486 Agtrap 0.30636956040209795

ENSMUST00000049972 1854 Scg2 0.22783922333159953

ENSMUST00000042412 900 Hey1 0.2561232692529047

ENSMUST00000031108 1440 Chrna9 0.2875032262008317

ENSMUST00000029999 1449 Polr1e 0.2662443079998905

ENSMUST00000040411 648 Rab42 0.2923128314087334

ENSMUST00000033430 714 Rab33a 0.3199014438715115

ENSMUST00000048966 5898 Tcf20 0.21541474870246016

ENSMUST00000032701 1224 Tdrd12 0.20676978617121394

ENSMUST00000036852 3651 Recql4 0.2635222558357053

ENSMUST00000031175 1254 Tmprss11d 0.16117262176028518

ENSMUST00000047405 3405 Nckap1l 0.2626320994222298

ENSMUST00000041550 999 Mgl2 0.2726590033270937

ENSMUST00000039796 720 Gucd1 0.4001376375152536

ENSMUST00000048603 12753 Dnah1 0.3413257791218818

ENSMUST00000043560 3441 Smg7 0.2042324229009719

ENSMUST00000034163 1242 Zfp821 0.22531103388035706

ENSMUST00000045245 1683 Tdrkh 0.18143983323383617

ENSMUST00000034488 1431 Mmp10 0.21431824235099345

ENSMUST00000033505 1563 Was 0.22858505625501002

ENSMUST00000032800 345 Tyrobp 0.223672347209491

ENSMUST00000038099 639 Socs1 0.33742559175275305

ENSMUST00000033263 1929 Umod 0.2954902247186501

ENSMUST00000029970 1011 Slc35a1 0.1477298367138558

ENSMUST00000034458 1713 Galnt2 0.3398815709453996

ENSMUST00000035667 1428 Trim62 0.3892387151529847

ENSMUST00000045972 1872 Slc5a12 0.18069989938414907

ENSMUST00000044524 309 Dap 0.27920812453086996

ENSMUST00000042312 1743 Trafd1 0.28946228554955855

ENSMUST00000047526 2715 Asap3 0.3198622393777084

ENSMUST00000036045 231 Leap2 0.22478384173853222

ENSMUST00000031355 2880 Uso1 0.1767373024467997

ENSMUST00000033973 567 Rwdd4a 0.21335418401904555

ENSMUST00000041768 972 Akr1c14 0.1926614549847843

ENSMUST00000039810 2892 Xpot 0.2159589011104111

ENSMUST00000041240 594 Shisa4 0.30082642079914135

ENSMUST00000030742 1122 Mecr 0.26206285855427197

ENSMUST00000029968 1737 Rars2 0.1534946293417619

ENSMUST00000045196 1494 Kcns1 0.3402996669163376

ENSMUST00000034610 300 Pate4 0.1907820670099598

ENSMUST00000033674 1053 Itgb1bp2 0.2245766238434996

ENSMUST00000048183 1131 Fmod 0.34506374798925465

ENSMUST00000039978 1044 Gabpb1 0.15416036668597247

ENSMUST00000030029 3189 Invs 0.23452086221102578

ENSMUST00000044620 9990 Brca2 0.1557773880527511

ENSMUST00000031127 1314 Slc10a4 0.302645122225836

ENSMUST00000036252 1389 Lmna 0.35958147995872747

ENSMUST00000037534 1044 Rnf167 0.22698585522710546

ENSMUST00000035069 687 Nradd 0.2719026336441342

ENSMUST00000040131 1839 Elf1 0.18169737914541506

ENSMUST00000035785 552 Ssr2 0.2581373336554279

ENSMUST00000033721 2964 Arhgap6 0.23807293931664963

ENSMUST00000038403 1437 Ica1 0.21007306180288088

ENSMUST00000030738 762 Utp11l 0.23699750592575816

ENSMUST00000038432 930 Olfr16 0.20612131220460375

ENSMUST00000037722 273 Banf2 0.35047956713997985

ENSMUST00000042744 1686 Npnt 0.24440341726063064

ENSMUST00000048656 1875 Ubash3a 0.3074441381623036

ENSMUST00000037718 1437 Fbxo15 0.192692607261467

ENSMUST00000049706 753 Fcer1a 0.19506187555420929

ENSMUST00000047737 1275 Irf8 0.36891185408291727

ENSMUST00000034591 1506 Bace1 0.3145680120291957

ENSMUST00000050104 1071 Gprc5a 0.2966552037197696

ENSMUST00000034066 1230 Mfap3l 0.2543790921397696

ENSMUST00000049537 1983 Picalm 0.15078025703157483

ENSMUST00000045243 3357 Pde4dip 0.2340162378499645

ENSMUST00000035120 348 Cck 0.2922303470581144

ENSMUST00000044857 567 Trappc5 0.34683018694800005

ENSMUST00000039627 5541 Bzrap1 0.29298058507637953

ENSMUST00000030122 261 Spink4 0.3101938740367641

ENSMUST00000033158 1107 Ubfd1 0.2676388927305528

ENSMUST00000038564 738 Asph 0.25210563540033265

ENSMUST00000049091 1332 Cox10 0.2790695492486819

ENSMUST00000031617 891 Rpl6 0.29313460839062483

ENSMUST00000042601 3687 Dhx38 0.3387464185437647

ENSMUST00000036221 1125 Fbxl8 0.26889604914577586

ENSMUST00000050157 954 Olfr686 0.22097114793185998

ENSMUST00000042710 183 Smim3 0.2922603073477918

ENSMUST00000050207 1095 Tefm 0.1923386166455798

ENSMUST00000033776 1530 Dkc1 0.18344981287708137

ENSMUST00000041105 2928 Sfmbt2 0.23573889835253697

ENSMUST00000041733 3597 Taf2 0.19730849373412834

ENSMUST00000029927 918 Spaca1 0.1833856242703823

ENSMUST00000049260 2631 Garem 0.2745932293955823

ENSMUST00000046908 876 Oser1 0.21636412570190028

ENSMUST00000039074 5193 Shank3 0.29880122957726274

ENSMUST00000030332 561 Gpx7 0.32255364727586433

ENSMUST00000036954 1047 Gbx2 0.30988742804672015

ENSMUST00000034079 2040 Heatr3 0.2118796119287129

ENSMUST00000042964 1827 Zfp189 0.20339121286672124

ENSMUST00000036380 618 Atp6v0b 0.2752572544914608

ENSMUST00000036387 279 S100b 0.4106370810494457

ENSMUST00000040971 1923 Capn5 0.3627919412799975

ENSMUST00000031633 1515 Cyp3a16 0.19749049509554478

ENSMUST00000037646 2412 Prdm4 0.22542389526720152

ENSMUST00000050248 3153 Zbtb11 0.1902420082780102

ENSMUST00000035325 2247 Qsox1 0.2816409752447225

ENSMUST00000041023 3123 Ipo9 0.25954427328862667

ENSMUST00000035266 1272 Igtp 0.27646537773795415

ENSMUST00000043923 1194 Acat3 0.22446058968272561

ENSMUST00000042026 2283 Pkd2l1 0.31396374946831646

ENSMUST00000047560 783 Dhrs11 0.31179092145951437

ENSMUST00000045702 1494 Slc2a6 0.30994792969910734

ENSMUST00000044711 579 Snx22 0.32293732608842257

ENSMUST00000033161 1917 Scnn1b 0.35865526796834235

ENSMUST00000030028 1221 Erp44 0.14933857193092182

ENSMUST00000045152 1032 Taar3 0.2082664618345297

ENSMUST00000036320 1128 Rhbdd3 0.28652300044328655

ENSMUST00000042054 1341 Foxf2 0.2884030193651342

ENSMUST00000040254 4221 Edc4 0.26588875617291746

ENSMUST00000044369 1452 Atp6v1h 0.18124525451100593

ENSMUST00000040437 483 Spaca5 0.26661175684639477

ENSMUST00000031654 426 Pomp 0.23273710361691488

ENSMUST00000034171 2469 Ap1g1 0.17933151353982513

ENSMUST00000030915 1434 Morn1 0.2756461700994495

ENSMUST00000033444 1251 Stk26 0.16953017615532504

ENSMUST00000037115 537 Med30 0.2661406282601408

ENSMUST00000034911 1428 Tinag 0.19802798395777416

ENSMUST00000046769 6036 Ckap5 0.17150575826569406

ENSMUST00000030731 486 Taf12 0.24276905227132647

ENSMUST00000042042 1023 Slc51a 0.28999279057174776

ENSMUST00000038039 1113 Tdp2 0.16332201865476692

ENSMUST00000039904 570 Camk2a 0.3583308711439838

ENSMUST00000030922 1779 Prkcz 0.3133633021113153

ENSMUST00000042699 1464 Aldh8a1 0.23296828041360923

ENSMUST00000032747 540 Hddc3 0.26974742935660795

ENSMUST00000034944 1113 Dapk2 0.3165442500222637

ENSMUST00000050226 1371 Smtnl2 0.31164608224579016

ENSMUST00000037964 2058 Txlnb 0.24794282328141018

ENSMUST00000034625 1431 Chek1 0.17181177979011847

ENSMUST00000031040 2100 Cwh43 0.1999201239384246

ENSMUST00000046948 1707 Cables1 0.3086898633865989

ENSMUST00000031868 1878 Slc13a4 0.34188077635947595

ENSMUST00000034198 1065 Gnao1 0.32708858896556653

ENSMUST00000040418 1077 Chad 0.32263360795409957

ENSMUST00000032261 828 Clec12b 0.24945722679203794

ENSMUST00000047629 2061 Cirh1a 0.20464737909918962

ENSMUST00000037218 612 Rasl10a 0.2750120303891424

ENSMUST00000041703 315 Dmkn 0.26780269777100946

ENSMUST00000032926 723 Tmem219 0.23403139591767347

ENSMUST00000036872 1338 Wipi2 0.2769209423355829

ENSMUST00000043169 2472 Arel1 0.2521583026490486

ENSMUST00000036578 2319 Bahd1 0.29982976021542035

ENSMUST00000044922 1029 Hs3st6 0.3133203754485419

ENSMUST00000039742 1131 Hykk 0.18037559750034923

ENSMUST00000044475 3141 Ogt 0.19269634134020347

ENSMUST00000038973 924 Gnptg 0.29521680559584157

ENSMUST00000036331 1257 Neu3 0.2894166555280165

ENSMUST00000035604 4509 Gemin5 0.23813146441167746

ENSMUST00000031536 741 Ftsj2 0.286612059760586

ENSMUST00000038475 1119 Fa2h 0.3594039906578576

ENSMUST00000046975 726 Pcgf3 0.2972452334734227

ENSMUST00000030254 396 Leprot 0.21484761421357618

ENSMUST00000032330 483 Emp1 0.32666832162810727

ENSMUST00000036606 3060 Stard8 0.2762932947557194

ENSMUST00000036653 1416 Htr2a 0.24442004872127995

ENSMUST00000044271 1827 Nploc4 0.2585346219662643

ENSMUST00000049874 1344 Agmo 0.17187524548476102

ENSMUST00000047421 3405 Tie1 0.2857498828910443

ENSMUST00000042470 2883 Zkscan2 0.22822697778648934

ENSMUST00000030181 729 Ccdc107 0.26048796028186977

ENSMUST00000030455 978 Akr1a1 0.2525346367232112

ENSMUST00000036807 633 Thap1 0.3445809412436935

ENSMUST00000030044 624 Orm1 0.23432373444888122

ENSMUST00000037399 621 Blvrb 0.29966551213676734

ENSMUST00000033991 4206 Wrn 0.17498970160039823

ENSMUST00000033754 1458 Piga 0.18021221221860456

ENSMUST00000032962 1317 Cln3 0.26563756343232103

ENSMUST00000049305 762 Espn 0.3239161797917439

ENSMUST00000032262 690 Clec1b 0.20338479455445543

ENSMUST00000044007 606 Oxld1 0.2815616333486065

ENSMUST00000034692 1347 Olfm2 0.355912655738105

ENSMUST00000031199 900 Sult1b1 0.19192882626261767

ENSMUST00000036572 750 Tmem51 0.3097977767317137

ENSMUST00000042724 3129 Usp38 0.18729588300471686

ENSMUST00000043596 3549 Phka1 0.1941712675152964

ENSMUST00000045186 723 Rpl3l 0.3347136534615263

ENSMUST00000034369 822 Psmb10 0.26930537448634323

ENSMUST00000041524 1446 Trmt13 0.15864391438694014

ENSMUST00000035565 12882 Pkd1 0.2727934424240381

ENSMUST00000045366 780 Dcun1d2 0.23920008452958322

ENSMUST00000034840 1383 Celf6 0.2934197020978127

ENSMUST00000038237 2052 Thoc5 0.2924356677498315

ENSMUST00000036206 945 Ccdc92 0.3872056591481142

ENSMUST00000034109 627 1700011L22Rik 0.2620720580289213

ENSMUST00000045291 14715 Kmt2c 0.18415152247478636

ENSMUST00000030724 1443 Sesn2 0.3389910943573065

ENSMUST00000045041 1731 Galnt12 0.2972937040405354

ENSMUST00000041377 1539 Lyn 0.2548732906942453

ENSMUST00000031565 1482 Fscn1 0.3868665393296891

ENSMUST00000034206 2166 Bbs2 0.23092530294400257

ENSMUST00000035721 1029 Prpf18 0.21646354249774874

ENSMUST00000033166 1365 4933427G17Rik 0.21769616614130352

ENSMUST00000040307 1047 March9 0.3386741772110491

ENSMUST00000034343 555 Cmtm3 0.2938190524685532

ENSMUST00000030661 933 Gpn2 0.38621115675722156

ENSMUST00000034560 1977 Ppp2r1b 0.19639499148905398

ENSMUST00000031405 990 Tmem116 0.26294031883011465

ENSMUST00000049896 1467 Gpkow 0.25088891284495324

ENSMUST00000031221 1695 Cdc7 0.18340865095210082

ENSMUST00000034054 915 Anxa10 0.2150592676396257

ENSMUST00000040404 384 Ly6d 0.3215605847038522

ENSMUST00000049239 2544 Map4k5 0.18945785533087414

ENSMUST00000039559 3516 Thbs1 0.31840481759753797

ENSMUST00000031018 972 Rbks 0.20787299666396447

ENSMUST00000030384 528 Edn2 0.3258827722254735

ENSMUST00000032566 1152 Qpctl 0.2993656237843053

ENSMUST00000046073 3930 Kdm2b 0.315725218181336

ENSMUST00000047793 987 Hoxd1 0.314599754228388

ENSMUST00000030586 603 Ccdc28b 0.3124514883658598

ENSMUST00000050214 1926 Akap8l 0.28735181619885763

ENSMUST00000046095 1092 Vsx1 0.2766392399418036

ENSMUST00000041364 2583 Nop14 0.22651097050043817

ENSMUST00000036236 1620 Mpp6 0.17301828799160981

ENSMUST00000040583 6117 Heatr5a 0.19144654251626922

ENSMUST00000035462 3624 Dsel 0.1330466263257854

ENSMUST00000039551 327 Polr3k 0.2780300464980988

ENSMUST00000032441 1329 Ccdc91 0.254657575130276

ENSMUST00000034448 1764 Glb1l3 0.2083436907180853

ENSMUST00000046383 876 Tnfsf10 0.26783783165697167

ENSMUST00000046916 1995 Ckap2 0.1645023456542631

ENSMUST00000043152 1794 Utp6 0.22081590910634258

ENSMUST00000046719 1044 Nfya 0.23553364000668384

ENSMUST00000032992 2736 Eif3c 0.2798739203914448

ENSMUST00000047368 618 Mnd1 0.18624378462013738

ENSMUST00000032910 510 Mylpf 0.3568406583884145

ENSMUST00000049721 708 Fam3b 0.2433629815670495

ENSMUST00000047111 1725 Pklr 0.28674500888371196

ENSMUST00000036615 1089 Hacd3 0.22685389943637818

ENSMUST00000034722 666 Rab27a 0.27773634956576526

ENSMUST00000047978 372 Rabif 0.341509305836709

ENSMUST00000048216 1014 Abhd13 0.20539200300850274

ENSMUST00000040481 1392 Slc38a7 0.328625084688797

ENSMUST00000043757 2514 Abcf1 0.31648328830158456

ENSMUST00000031554 2094 Tmem168 0.1626826003676989

ENSMUST00000034775 1884 Fem1b 0.23103799885186277

ENSMUST00000036540 1755 Fam117b 0.2585034124080818

ENSMUST00000048781 2004 Pck2 0.29106538072601496

ENSMUST00000033035 2022 Slc5a11 0.29490215608895776

ENSMUST00000034878 1095 Tmem30a 0.19257289988413426

ENSMUST00000043616 2235 Zyg11b 0.20312560317281406

ENSMUST00000034091 3408 Rbl2 0.1979672232586889

ENSMUST00000049031 1806 Fam63b 0.22218586974787102

ENSMUST00000032779 1389 Ctsc 0.2250669907867078

ENSMUST00000048527 633 Abhd14b 0.31000593437247165

ENSMUST00000030535 441 Cda 0.3467170462015786

ENSMUST00000031327 291 Cxcl1 0.2776207114355376

ENSMUST00000034349 1605 Nae1 0.1414704456003936

ENSMUST00000031423 3135 Atp2a2 0.24555338669841806

ENSMUST00000045224 828 Acer2 0.2729179234846124

ENSMUST00000045477 1197 Stk32a 0.2796770710102545

ENSMUST00000047305 630 Wbscr28 0.2978414063091436

ENSMUST00000034787 5466 Phip 0.15670683138828073

ENSMUST00000033057 693 Dkkl1 0.31343205590591655

ENSMUST00000031198 1287 Fam69a 0.17066737330397186

ENSMUST00000049719 945 Olfr478 0.2189910742664271

ENSMUST00000030530 453 Pla2g2c 0.3869015973699659

ENSMUST00000049628 216 Atp5k 0.2624321155759897

ENSMUST00000048377 3765 Suco 0.14710582956558074

ENSMUST00000032183 1203 Tmem43 0.28565990371266503

ENSMUST00000049968 717 Odf3b 0.2862244700399482

ENSMUST00000046390 3324 Myo1b 0.22810310808689907

ENSMUST00000035202 1671 Mon1a 0.31217945793529217

ENSMUST00000030950 651 Cptp 0.31532505858884713

ENSMUST00000048915 522 Rbm8a 0.21270974259560768

ENSMUST00000036113 2301 Tbc1d16 0.36420071795361675

ENSMUST00000049810 1056 Cxcr6 0.24904318419287189

ENSMUST00000035100 3945 Ttc21a 0.32707984627460335

ENSMUST00000032486 753 Cd27 0.3099504495091724

ENSMUST00000033255 1605 Gp2 0.2590754099668856

ENSMUST00000037811 13518 Apob 0.189447312351964

ENSMUST00000034851 1515 Chrna3 0.30163003676025996

ENSMUST00000034830 414 Crabp1 0.33692451345145397

ENSMUST00000030465 723 Tspan1 0.25479545768687095

ENSMUST00000043962 1863 Cdc16 0.21413201848124686

ENSMUST00000030163 3552 1700022I11Rik 0.19828877503871978

ENSMUST00000040112 1491 Papl 0.3107054308174486

ENSMUST00000034164 1089 Ist1 0.2159410433915411

ENSMUST00000032560 1215 Ppm1n 0.2912773301138231

ENSMUST00000032774 1467 Ttc23 0.2516651952971221

ENSMUST00000036328 7689 Zfhx2 0.2881748212760062

ENSMUST00000032177 1389 Slc41a3 0.32428535217605164

ENSMUST00000031268 774 Enoph1 0.30506227565213495

ENSMUST00000037615 1572 Aldh5a1 0.24993760436969475

ENSMUST00000045807 2412 Tsr1 0.2072143402356534

ENSMUST00000041010 1635 Ccdc9 0.31736944342768425

ENSMUST00000042147 1551 Noc4l 0.3190573321572916

ENSMUST00000030948 2088 Dvl1 0.3302366004054632

ENSMUST00000034497 1440 Mmp3 0.21595904462970125

ENSMUST00000037666 3147 Mfhas1 0.30438014437755473

ENSMUST00000033846 1491 Angpt2 0.3104190485618993

ENSMUST00000049248 2466 Malt1 0.1757388106342472

ENSMUST00000033933 1092 Saraf 0.2464000784793171

ENSMUST00000047857 2073 Fbxl5 0.18672153057061255

ENSMUST00000049146 1080 Ephx4 0.21312381374244344

ENSMUST00000049257 1797 Ddx52 0.17681011981873784

ENSMUST00000031531 369 Psmg3 0.3067335374367503

ENSMUST00000032356 1944 Plcz1 0.16583731878845442

ENSMUST00000048043 1443 Coro2b 0.3375869984279503

ENSMUST00000045301 666 Hist1h1d 0.3789266958385356

ENSMUST00000047655 1026 Slc25a43 0.23235954698959715

ENSMUST00000041413 1056 E330034G19Rik 0.29344641220690054

ENSMUST00000033763 708 Naa10 0.3361881896195195

ENSMUST00000043739 816 Elovl3 0.27873116222285665

ENSMUST00000049648 1824 Kbtbd3 0.15489177686935615

ENSMUST00000040992 2955 Nek9 0.23773755277122158

ENSMUST00000039840 1323 Enpp6 0.28362641702795904

ENSMUST00000036987 1731 Ncaph2 0.3238634995982945

ENSMUST00000049184 2043 Ushbp1 0.30535538648278726

ENSMUST00000048065 552 Trem3 0.2724928856948625

ENSMUST00000046659 2934 Dlgap3 0.34413909979212204

ENSMUST00000041353 1149 Slc35b2 0.28181446106888547

ENSMUST00000039568 3213 Pcdh8 0.30463730249974613

ENSMUST00000034734 1263 Dyx1c1 0.17421556475248468

ENSMUST00000041093 1566 Creb3l2 0.2968516339609358

ENSMUST00000041142 1896 Muc1 0.23987704638453128

ENSMUST00000031599 762 Rita1 0.2987372758669891

ENSMUST00000041589 1089 Tob1 0.2674624766980754

ENSMUST00000032551 1392 Zik1 0.1983156087529083

ENSMUST00000038359 3795 2210018M11Rik 0.18529861969674957

ENSMUST00000047436 1026 Thoc6 0.2833695203418406

ENSMUST00000049294 357 Snrpd2 0.41118765243906297

ENSMUST00000033466 783 Cd40lg 0.15472069146892498

ENSMUST00000034815 2862 Kif23 0.19381559770118723

ENSMUST00000035340 4767 Usp54 0.22353282456090803

ENSMUST00000032477 828 Necap1 0.22153855031338193

ENSMUST00000039490 930 Tnfsf9 0.23589866276219865

ENSMUST00000035521 2127 Rsph6a 0.37778839483323684

ENSMUST00000041115 2142 Erich6 0.19870469752250855

ENSMUST00000040636 1170 Sec1 0.3389616025430868

ENSMUST00000037620 708 Mospd3 0.26586299053523416

ENSMUST00000041096 777 Pcsk1n 0.2647566975808568

ENSMUST00000046030 2538 Esf1 0.1738991700875335

ENSMUST00000046001 507 Avp 0.4449167256658126

ENSMUST00000037728 2949 Nlrp4c 0.21556267122940737

ENSMUST00000033609 1743 Cstf2 0.19925698699702413

ENSMUST00000041231 5532 Psme4 0.14911357411883386

ENSMUST00000043610 4269 Ric1 0.21110824604493086

ENSMUST00000035673 546 Vhl 0.2872152870382688

ENSMUST00000037014 966 BC022687 0.26321574782861673

ENSMUST00000040498 1059 Rnf39 0.2980873520784511

ENSMUST00000045105 1932 Spire1 0.2510213946156701

ENSMUST00000049166 357 Bet1 0.14145842564398184

ENSMUST00000033468 2388 Arhgef6 0.18763772431599635

ENSMUST00000040915 2259 Ppig 0.18372290345159292

ENSMUST00000032307 447 Magohb 0.19139935000767835

ENSMUST00000039672 1401 Mfsd9 0.24804181956096777

ENSMUST00000034984 2544 Rasa2 0.18032812033438483

ENSMUST00000031590 2859 Rbm19 0.33531536133961

ENSMUST00000039049 702 Syngr4 0.31642969116633723

ENSMUST00000037261 1149 Ptgdr2 0.3254207153326565

ENSMUST00000045127 381 Wfdc5 0.2703336109641484

ENSMUST00000039021 1845 Ssx2ip 0.22710700729145802

ENSMUST00000034239 1977 Katnb1 0.32453955247331945

ENSMUST00000037299 1680 Ythdf1 0.25676602563687345

ENSMUST00000044116 2016 Spg20 0.2185600283282411

ENSMUST00000039652 327 Ins1 0.33495953435344494

ENSMUST00000042818 981 Pim3 0.3773435448314319

ENSMUST00000049705 1944 Zfp457 0.1621734304289301

ENSMUST00000045555 4059 Etl4 0.24745293155214493

ENSMUST00000040383 2832 Cc2d1a 0.3082744312866439

ENSMUST00000048208 873 Ccdc122 0.12489562203969662

ENSMUST00000038949 1401 Adrb1 0.36386910344274664

ENSMUST00000040961 1146 Pabpc5 0.16962520789751848

ENSMUST00000032839 1653 Det1 0.3013840681679942

ENSMUST00000034429 948 Tmem231 0.3185045027968621

ENSMUST00000041007 1095 Gjd4 0.2675175457860102

ENSMUST00000031091 4191 D5Ertd579e 0.18975631303138912

ENSMUST00000045697 384 Mrpl55 0.2812910101220441

ENSMUST00000034215 186 Mt1 0.4796721810020953

ENSMUST00000030849 1566 Fam126a 0.18708058814882458

ENSMUST00000042497 1395 Ndufv1 0.28573744894093206

ENSMUST00000037762 1989 Hif3a 0.3045298385468639

ENSMUST00000036934 21459 Neb 0.2733319086045143

ENSMUST00000047281 522 2310036O22Rik 0.27725742258713565

ENSMUST00000049729 1065 Olfr735 0.20895405300505843

ENSMUST00000048568 849 Fam212a 0.22902795545583726

ENSMUST00000049053 708 Fam168a 0.31053581944001907

ENSMUST00000034846 3072 1700017B05Rik 0.2578501414791895

ENSMUST00000032633 2502 Oca2 0.2041349298291416

ENSMUST00000039234 1866 Fbxl4 0.19642894853379156

ENSMUST00000044155 1917 Ubash3b 0.24793443050106642

ENSMUST00000042717 1467 Trim39 0.2704324104853977

ENSMUST00000045846 1029 Sfxn5 0.27507502403379963

ENSMUST00000047477 258 Sprr2d 0.3810260324408

ENSMUST00000044331 6012 Kat6a 0.23700115317337542

ENSMUST00000042410 981 Cdk6 0.27489383983715826

ENSMUST00000034856 1272 Mpi 0.30588996632252785

ENSMUST00000046974 1401 Fbxw17 0.2899693462447599

ENSMUST00000044355 267 Sepw1 0.3065562725308126

ENSMUST00000048129 2916 Piwil2 0.2551175014285914

ENSMUST00000032888 612 Arl6ip1 0.2139325561720853

ENSMUST00000048946 552 1110007C09Rik 0.35129087096948075

ENSMUST00000042834 825 Uqcrfs1 0.21498741507397043

ENSMUST00000035214 1302 Ip6k1 0.32995802675150326

ENSMUST00000034278 558 Gins2 0.38849835062037263

ENSMUST00000033929 3963 Tnks 0.20391198849750325

ENSMUST00000033543 207 Cmc4 0.1597781661834872

ENSMUST00000047511 1896 Shtn1 0.20846435404833644

ENSMUST00000042217 687 Rspo4 0.32603075024273437

ENSMUST00000048762 3753 Cilp 0.29761964077610237

ENSMUST00000035889 3789 Myo6 0.21452480095386842

ENSMUST00000049079 738 Gm5771 0.3158522803550422

ENSMUST00000043707 1086 Rhbdd2 0.33600248956292406

ENSMUST00000038384 2211 Ythdc1 0.16142867173284708

ENSMUST00000031622 330 Ocm 0.2962164164013758

ENSMUST00000030852 2379 Rint1 0.23402661161152694

ENSMUST00000036229 2883 Ctdp1 0.28781132561506073

ENSMUST00000047799 2340 Acad11 0.23356109707667003

ENSMUST00000042824 14580 Herc1 0.16725223005638618

ENSMUST00000032157 1335 Gxylt2 0.30021381715575934

ENSMUST00000044773 4650 Frmpd1 0.27284305554907085

ENSMUST00000038824 1545 Cyp24a1 0.28152879908970785

ENSMUST00000034552 567 Fdx1 0.22507600525878518

ENSMUST00000034033 1470 1700007B14Rik 0.1535390021545158

ENSMUST00000036074 2916 Gmip 0.3031940503730411

ENSMUST00000031144 972 Tmem165 0.18265522473625587

ENSMUST00000036462 1617 Fam214b 0.2814708757530121

ENSMUST00000030191 3144 Npr2 0.2662546174265423

ENSMUST00000037275 2472 Ccdc15 0.16412991461389173

ENSMUST00000037489 858 Agpat1 0.3433046905227801

ENSMUST00000034014 636 Fgf20 0.24039627619778497

ENSMUST00000030628 1995 Tmem57 0.2302455305687339

ENSMUST00000050160 1158 AU021092 0.300056842068868

ENSMUST00000034316 3009 BC021891 0.2813099624841363

ENSMUST00000041920 1008 Cd1d2 0.24743242881248745

ENSMUST00000039160 1548 Gpr176 0.29165277444086135

ENSMUST00000047906 1974 Tiparp 0.1620686055845058

ENSMUST00000033966 555 Dctd 0.27234062616617505

ENSMUST00000036561 2031 Wdr48 0.20918475904177766

ENSMUST00000032419 1299 Cmas 0.21076244807613925

ENSMUST00000030305 1512 Cyp2j13 0.22871528311608572

ENSMUST00000035854 540 Cdrt4 0.2403664472939508

ENSMUST00000033686 606 Dmrtc1a 0.2638441985431094

ENSMUST00000048138 483 S100a13 0.2683028118781983

ENSMUST00000045102 894 Gipc3 0.3465278575923948

ENSMUST00000042581 495 6430571L13Rik 0.3634336031814794

ENSMUST00000038627 1719 Zfp91 0.21708645641573057

ENSMUST00000037341 4764 Nhsl1 0.24705371473555507

ENSMUST00000033956 1224 Ido1 0.24011854213712314

ENSMUST00000033473 738 Fgf13 0.25039690300322115

ENSMUST00000032286 843 Klra7 0.1796954304501433

ENSMUST00000030577 618 Tmem54 0.26479050024549483

ENSMUST00000030896 801 Tprgl 0.36958827812889555

ENSMUST00000047630 1110 Actrt3 0.23351094124769428

ENSMUST00000034413 600 Vstm5 0.29794916363241897

ENSMUST00000047885 1404 Nt5dc1 0.22589231928332174

ENSMUST00000031663 1770 C8b 0.23225058947603344

ENSMUST00000034405 2121 Mre11a 0.23050338872896264

ENSMUST00000033560 2409 Ophn1 0.22208069665294303

ENSMUST00000037900 1674 Cpne7 0.3589402493847956

ENSMUST00000033994 576 Rbpms 0.25711458740458687

ENSMUST00000042747 1116 Npy6r 0.2098658404048676

ENSMUST00000034385 1665 Has3 0.30542578240959467

ENSMUST00000034203 2358 Cog4 0.2710201815820242

ENSMUST00000049920 4680 Ino80 0.20207314835628917

ENSMUST00000039191 288 1810011H11Rik 0.21799893810290477

ENSMUST00000031984 3066 Smarcad1 0.1521093384846018

ENSMUST00000035216 2934 Uba7 0.27356199942529974

ENSMUST00000047028 519 Lgalsl 0.21604729406392925

ENSMUST00000038677 3888 Rrp12 0.30506381509041175

ENSMUST00000037609 1971 Hgsnat 0.23597598628646504

ENSMUST00000037285 2313 Git1 0.3087549921861241

ENSMUST00000042986 3291 Plcl1 0.21836627171107026

ENSMUST00000032335 3921 Atf7ip 0.20412616273197276

ENSMUST00000044790 1566 Foxn4 0.34516685299614414

ENSMUST00000034946 1566 Snx1 0.2620016669022223

ENSMUST00000046373 3588 Iqsec3 0.3272726772351873

ENSMUST00000040787 1626 Ankrd13c 0.20402148013484003

ENSMUST00000044642 3030 Micall2 0.27444154171752744

ENSMUST00000043378 1032 Tmem120a 0.37236151799875206

ENSMUST00000034945 483 Fam96a 0.1908253043698485

ENSMUST00000047689 2052 Fignl1 0.17285708340010394

ENSMUST00000043914 2064 Ints9 0.2853873176689226

ENSMUST00000048702 1455 Papd4 0.15487504760631354

ENSMUST00000035167 3978 Nphp3 0.23953693063593484

ENSMUST00000032239 645 Clec4e 0.2283160286916276

ENSMUST00000035983 483 Rpl21 0.31962340693886765

ENSMUST00000035433 558 Hesx1 0.20418317764093785

ENSMUST00000030143 3399 Ubap2 0.23723806364136718

ENSMUST00000031779 948 Calu 0.252823958306371

ENSMUST00000038893 1563 Rcc2 0.32463559045056994

ENSMUST00000034382 1911 Zfp90 0.22450124928347961

ENSMUST00000042790 549 Hspb2 0.30230717638466037

ENSMUST00000040275 2595 Sobp 0.2809642365966985

ENSMUST00000032827 1080 Hapln3 0.29830960181355864

ENSMUST00000033519 351 Dynlt3 0.2089828436641873

ENSMUST00000034629 1512 Hinfp 0.29242793810289947

ENSMUST00000047275 1545 Bace2 0.25151518783004606

ENSMUST00000046662 1614 Gm4847 0.22213105474200445

ENSMUST00000034355 1680 Ces2e 0.26830304762060003

ENSMUST00000049937 750 Chtop 0.1818456499960146

ENSMUST00000033095 1839 Prr14 0.23251369817451328

ENSMUST00000048184 1455 Pdcd7 0.2520013413201337

ENSMUST00000045097 1917 Gbp7 0.2445468050790365

ENSMUST00000040962 1074 Nudt19 0.26748891905712496

ENSMUST00000049230 1206 Kcnab1 0.25069529066881885

ENSMUST00000036019 12033 Fras1 0.2637459699265863

ENSMUST00000035645 4572 Rusc2 0.2980620104882798

ENSMUST00000031181 1599 Ugt2b34 0.21007957433672922

ENSMUST00000047194 714 Igsf6 0.22486714596198715

ENSMUST00000047168 1605 Pde9a 0.3300819849834416

ENSMUST00000045374 444 Ramp3 0.37473142578698404

ENSMUST00000034487 1449 Mmp20 0.24046767522771612

ENSMUST00000048462 1023 Marc1 0.28712506673898514

ENSMUST00000034218 3009 Slc12a3 0.34141311524968165

ENSMUST00000034457 4575 Urb2 0.20884010077536083

ENSMUST00000034272 816 Mvb12a 0.3149580299350585

ENSMUST00000040065 2976 Tlr13 0.19186677825781326

ENSMUST00000042665 5454 Parp14 0.2069105875810093

ENSMUST00000034307 1959 Pde4c 0.31553278109771055

ENSMUST00000039113 1584 Pdia2 0.3066358540986293

ENSMUST00000037378 570 1700020L24Rik 0.2978136751608995

ENSMUST00000030533 3648 Vwa5b1 0.3214703098268523

ENSMUST00000033755 885 Asb11 0.2040817508032104

ENSMUST00000037408 4197 Scaper 0.16906873480203558

ENSMUST00000040284 219 BC031181 0.2153026220771772

ENSMUST00000048229 2715 Myrfl 0.23563681342876186

ENSMUST00000041973 633 Cmtm2b 0.22357280156147194

ENSMUST00000034742 1197 Ccnb2 0.266489702147045

ENSMUST00000049463 684 Prl3a1 0.19746060788755132

ENSMUST00000044694 561 Ttc36 0.2972631599822995

ENSMUST00000047502 1749 Mynn 0.17835659170390794

ENSMUST00000045674 879 Ppapdc2 0.3362437546840643

ENSMUST00000031899 2142 Kel 0.2293114186069437

ENSMUST00000047443 1245 Mansc1 0.2107885695656968

ENSMUST00000039506 1836 Igsf8 0.28363174418465575

ENSMUST00000040881 1242 Cluap1 0.2341412325939866

ENSMUST00000037071 933 Olfr796 0.2853393547660035

ENSMUST00000045971 1506 Chrnb1 0.26361730101677133

ENSMUST00000049424 1155 Wdr74 0.22594575010106324

ENSMUST00000035230 1212 Amt 0.22485814004334617

ENSMUST00000032386 1233 Bhlhe41 0.23487336209193319

ENSMUST00000037330 1101 Inha 0.2615722977289889

ENSMUST00000040340 2430 Fcho2 0.15985687474934196

ENSMUST00000041759 1785 Hmgxb4 0.24820437322859823

ENSMUST00000043087 1158 Alg12 0.23860531167121427

ENSMUST00000043797 900 Capza3 0.19830086558895632

ENSMUST00000035007 552 Cmtm6 0.3204792522214858

ENSMUST00000031646 729 Rasl11a 0.2979267513259378

ENSMUST00000030775 5859 Chd5 0.3728970656837547

ENSMUST00000033333 600 Tmem9b 0.23477470748671286

ENSMUST00000032216 306 Ptms 0.3068992439688283

ENSMUST00000044338 3918 Arhgap33 0.2788898406722551

ENSMUST00000041815 2094 Tsga10 0.20246662834180754

ENSMUST00000034432 888 Cfdp1 0.20095644086409611

ENSMUST00000046254 2367 Lrfn2 0.29819983270519224

ENSMUST00000040442 4011 Aox4 0.23820806053785923

ENSMUST00000048001 2610 Dmgdh 0.2419677047277702

ENSMUST00000047950 8097 Zfp292 0.16171967612491697

ENSMUST00000030817 954 Car6 0.30038416127689715

ENSMUST00000045562 1242 Cox15 0.24474848202003402

ENSMUST00000047860 2082 Noa1 0.2439429239237417

ENSMUST00000047373 696 Sox15 0.2875066004921036

ENSMUST00000029991 540 Ppp3r2 0.3499708741151367

ENSMUST00000034140 1833 Itfg1 0.1714002670071658

ENSMUST00000032844 591 Tmem126a 0.11823397856615596

ENSMUST00000040576 891 Parm1 0.2754203610103184

ENSMUST00000035931 3405 Pcdh18 0.21351061846658143

ENSMUST00000030121 1200 B4galt1 0.24326095750060794

ENSMUST00000034934 774 Aph1b 0.22993750517771092

ENSMUST00000047672 1608 Cct2 0.17921909021651367

ENSMUST00000045736 4746 Mroh2b 0.22741683021737516

ENSMUST00000041956 3924 Spag9 0.19798170148516708

ENSMUST00000044638 984 Stx16 0.2720495776393077

ENSMUST00000039212 3006 Slc4a5 0.3252961845152685

ENSMUST00000044970 1617 Mgat3 0.358263541238525

ENSMUST00000044850 4872 Abca9 0.19471240781216753

ENSMUST00000030076 1572 Ptbp3 0.1825214052873218

ENSMUST00000047762 1770 Gata6 0.2739902574809573

ENSMUST00000031778 393 1700012A03Rik 0.2676841861035065

ENSMUST00000034808 795 Nnmt 0.2743911867777118

ENSMUST00000031843 294 Npy 0.2922475790608188

ENSMUST00000033282 951 Bccip 0.2504805115871413

ENSMUST00000045756 294 S100a10 0.2805674419587134

ENSMUST00000040241 1440 Ddx19b 0.2899431656756379

ENSMUST00000048207 987 Aipl1 0.4019572166464269

ENSMUST00000048670 1128 Slc35f2 0.22443687948587318

ENSMUST00000044953 1128 Svs2 0.13599995504545376

ENSMUST00000032627 3075 Tubgcp5 0.1920574623270761

ENSMUST00000031680 1266 Ing3 0.20256347047329934

ENSMUST00000030245 1341 4921539E11Rik 0.15658042246882645

ENSMUST00000030644 405 Zfp593 0.3093007773269973

ENSMUST00000035540 3291 Phf2 0.33052101939461415

ENSMUST00000043208 2328 Six4 0.2374162928594901

ENSMUST00000032165 1371 Ruvbl1 0.30337775926862254

ENSMUST00000041284 5505 Inadl 0.2020365618459575

ENSMUST00000043069 1182 Cyth4 0.38770758836574504

ENSMUST00000033218 1440 2610020H08Rik 0.18525209392050832

ENSMUST00000049388 609 Commd10 0.2423408952242327

ENSMUST00000047621 2475 Ppp1r13l 0.31103217735338684

ENSMUST00000041264 1740 Zfp938 0.14798729122870546

ENSMUST00000037049 1800 Lzts1 0.343854839178937

ENSMUST00000044426 321 Guca2b 0.3179485371247455

ENSMUST00000037618 1581 Rbpj 0.1988164348182225

ENSMUST00000030299 1506 Cyp2j5 0.21741558701144043

ENSMUST00000041874 963 Npl 0.2526241990255529

ENSMUST00000035086 2610 Pdcd6ip 0.2197301979401578

ENSMUST00000036493 3072 Atp1a1 0.29224931001870064

ENSMUST00000045714 2022 Abcg8 0.30954937075397154

ENSMUST00000047660 1044 Pglyrp3 0.23038479892628858

ENSMUST00000041616 939 Pdxk 0.36813619322004776

ENSMUST00000047531 768 Mettl20 0.1922272620848585

ENSMUST00000046514 4539 Eprs 0.21077092804730363

ENSMUST00000039887 1764 Pof1b 0.16706481031339304

ENSMUST00000036647 1398 Ctdspl2 0.15340992961569752

ENSMUST00000035651 1332 Lrrc17 0.24398286384166126

ENSMUST00000035014 1704 Tgfbr2 0.3667360436747137

ENSMUST00000039534 528 Resp18 0.2082327248092987

ENSMUST00000044053 5025 Shprh 0.19623758550383275

ENSMUST00000037280 2922 Vps18 0.3284432080974892

ENSMUST00000047558 1812 Prc1 0.20637709933298443

ENSMUST00000049792 2265 Kcnq2 0.3099820618528586

ENSMUST00000035342 5721 Adamts20 0.22731131402880805

ENSMUST00000032074 684 Cml5 0.2302288564372285

ENSMUST00000032327 1035 Gprc5d 0.2892719726725632

ENSMUST00000049055 918 Lysmd3 0.1472068868720043

ENSMUST00000033871 906 Slc25a15 0.24381213042824681

ENSMUST00000034709 576 Bcl2l10 0.27744395023343305

ENSMUST00000031251 897 Hsd17b11 0.2360921084871504

ENSMUST00000043335 1164 Fggy 0.20531276433656956

ENSMUST00000033806 1224 Vsig1 0.19151164946571383

ENSMUST00000035493 1248 Hnrnpf 0.2509297475939681

ENSMUST00000031967 714 Prss37 0.2147719226906944

ENSMUST00000044923 2574 Ddx24 0.25627438298393407

ENSMUST00000034702 648 Lysmd2 0.2620682971551658

ENSMUST00000033180 3447 Vwa3a 0.26867583417687796

ENSMUST00000032260 624 Clec2d 0.23935360931293378

ENSMUST00000034568 372 Tex12 0.17920556787250097

ENSMUST00000043437 2067 Fchsd1 0.2713594450331509

ENSMUST00000047740 555 Upk2 0.25210992425532935

ENSMUST00000049484 1119 Gxylt1 0.21911380788713133

ENSMUST00000032487 357 Vamp1 0.286251271808321

ENSMUST00000029947 1443 Gabrr1 0.3300905519195491

ENSMUST00000047835 4371 Scaf11 0.1791196586337363

ENSMUST00000038080 1362 Fer 0.22801508488127192

ENSMUST00000036299 3627 Camta2 0.28218360236180445

ENSMUST00000035155 627 Rab6b 0.3118505550983924

ENSMUST00000040413 918 Ccdc83 0.19921376913926633

ENSMUST00000036172 1065 Sgpp2 0.27227045196958627

ENSMUST00000044165 3111 Itga9 0.29485902979678985

ENSMUST00000030164 2421 Vcp 0.20885799753717102

ENSMUST00000045277 1728 Epn1 0.3051340545173798

ENSMUST00000042399 4326 Rsf1 0.19105835081837566

ENSMUST00000038474 882 Exosc2 0.25217602601595257

ENSMUST00000049811 2967 Cep120 0.21922024996896813

ENSMUST00000030102 3165 Kdm4c 0.19551768794051577

ENSMUST00000036273 2187 Nfkbiz 0.2762114152194484

ENSMUST00000043283 2040 Git2 0.2549266154096007

ENSMUST00000032269 1260 Rad52 0.24100631501129774

ENSMUST00000038539 429 1700123O20Rik 0.24700955179139886

ENSMUST00000038956 789 Lrrc18 0.3150518460247287

ENSMUST00000038161 1077 Agmat 0.2909153585796425

ENSMUST00000041662 1035 H2-M1 0.236067567864857

ENSMUST00000037035 1620 Ripk2 0.17015746611640034

ENSMUST00000042203 987 Wdr5b 0.16866009323056116

ENSMUST00000043414 3825 Dennd3 0.3217989770724528

ENSMUST00000030741 4341 Ptpru 0.35735449578620726

ENSMUST00000034713 2589 Ldlr 0.34420895228029647

ENSMUST00000041168 3498 Adgrg6 0.21550155346529695

ENSMUST00000048054 2910 Chtf18 0.295230193803157

ENSMUST00000034761 2967 Ice2 0.15294677123330158

ENSMUST00000033999 777 Frg1 0.18592847342644703

ENSMUST00000033554 1368 Gpr165 0.25396149018266456

ENSMUST00000035061 504 Ngp 0.26213465840246714

ENSMUST00000045011 2358 Atrip 0.2152557383324013

ENSMUST00000048686 891 Prpmp5 0.233631763959024

ENSMUST00000036643 945 Lrrc52 0.2590758152476415

ENSMUST00000030730 864 Trnau1ap 0.32563303207697325

ENSMUST00000042281 972 Dhrs7b 0.2807250979865609

ENSMUST00000042035 4749 Adgrb1 0.3467991313969787

ENSMUST00000038477 2019 Fam65b 0.27074792789730145

ENSMUST00000029964 2997 Epha7 0.18515317012482146

ENSMUST00000037715 318 Dusp15 0.2696099687147034

ENSMUST00000030263 1125 St3gal3 0.304145116035585

ENSMUST00000031172 984 Gnrhr 0.25298501761282693

ENSMUST00000033030 1119 Parva 0.2796973698973717

ENSMUST00000045004 6888 Dopey2 0.2885476000578882

ENSMUST00000036579 13173 Vps13d 0.23582970569221967

ENSMUST00000039132 1605 Zfp472 0.19839062973646943

ENSMUST00000045847 1656 Erf 0.3060770724134517

ENSMUST00000034469 1203 Egln1 0.31208780445234824

ENSMUST00000048655 1452 Dusp10 0.28225914445528594

ENSMUST00000043794 1881 Jakmip1 0.3621271590078993

ENSMUST00000034561 1836 Alg9 0.24094669230371424

ENSMUST00000038368 447 Id1 0.3461390519648977

ENSMUST00000050178 1638 Ccdc60 0.366095574069293

ENSMUST00000043881 2760 Bclaf1 0.18506767198774038

ENSMUST00000033051 3609 Itgad 0.2788641363065215

ENSMUST00000045288 1245 Tgfb2 0.27160129367397057

ENSMUST00000042889 4326 Setd5 0.19104758003901418

ENSMUST00000033775 1401 Mpp1 0.233488919627061

ENSMUST00000030723 2028 Mtf1 0.22997131360950845

ENSMUST00000048934 2046 Tbr1 0.2876866069223185

ENSMUST00000048665 8610 Chd9 0.18302631509048092

ENSMUST00000048444 882 Klk5 0.30739646639601886

ENSMUST00000044250 1188 Alkbh5 0.37056967614790204

ENSMUST00000030136 912 Aqp7 0.24472910834542583

ENSMUST00000034296 2169 Pik3r2 0.3397738522003261

ENSMUST00000031366 6624 Kntc1 0.21232804731802887

ENSMUST00000045296 849 Siah1a 0.19532600247492216

ENSMUST00000044858 1575 Rxrb 0.2963233325718749

ENSMUST00000035748 1371 Armcx1 0.1945859605297833

ENSMUST00000041767 2898 Tbc1d2b 0.3012851506116107

ENSMUST00000030036 2283 Brinp1 0.30382140293045357

ENSMUST00000031891 1047 Fam131b 0.27930301764634347

ENSMUST00000031186 1590 Ugt2b35 0.1769835039740646

ENSMUST00000031008 927 Stx18 0.25392608225118823

ENSMUST00000031410 1422 Mapkapk5 0.2503072066770788

ENSMUST00000032748 2835 Unc45a 0.33805536324514684

ENSMUST00000048432 1137 Prelp 0.37731272085378237

ENSMUST00000049038 2349 Sox30 0.2466769659945947

ENSMUST00000038876 492 Emp3 0.33730906998953925

ENSMUST00000046288 1407 Ndufv3 0.24471513342173024

ENSMUST00000034133 2388 Mylk3 0.24685149223842748

ENSMUST00000033372 1044 Rp2h 0.1909929359117323

ENSMUST00000040972 1587 Utp15 0.1834377046579043

ENSMUST00000032362 2148 Slco1c1 0.1778311317462891

ENSMUST00000033930 1197 Dusp4 0.39224312769266395

ENSMUST00000034597 1647 Tmprss13 0.3215554798704273

ENSMUST00000032207 567 Klrg1 0.23947127726285047

ENSMUST00000043111 1413 Edil3 0.193556993762599

ENSMUST00000038730 792 Rtp1 0.33793734680353116

ENSMUST00000047749 465 4921524J17Rik 0.17049486168195996

ENSMUST00000033159 1572 Ears2 0.3158937826116213

ENSMUST00000034910 810 Mlip 0.1803356980881583

ENSMUST00000049694 1020 Vmn1r26 0.18653748977815302

ENSMUST00000033152 591 Chp2 0.2812954577699308

ENSMUST00000040167 1005 Mat2b 0.22627726223149294

ENSMUST00000035657 1161 Tssc1 0.3013358319321338

ENSMUST00000040321 993 Trmt10a 0.23062506559580798

ENSMUST00000043266 651 4930596D02Rik 0.2605251075378199

ENSMUST00000048016 1470 Fut11 0.26993349821140816

ENSMUST00000049074 5697 Ptprf 0.32900531908885233

ENSMUST00000033136 1734 Bag3 0.28025379250343274

ENSMUST00000032459 864 Vgll4 0.3321893258984546

ENSMUST00000047331 1647 Lgi3 0.30967505185133676

ENSMUST00000037977 1917 Ccpg1 0.22974798502193094

ENSMUST00000033876 4011 Adgra2 0.2939234568831312

ENSMUST00000032559 1416 Rtn2 0.26991435555472115

ENSMUST00000048188 3042 Phf20l1 0.1749704305831129

ENSMUST00000034723 1557 Aldh1a2 0.2531634504059098

ENSMUST00000034303 486 Mphosph6 0.23836117578867164

ENSMUST00000033093 579 Bax 0.3605134798396842

ENSMUST00000035043 2022 Armc8 0.16518659439614675

ENSMUST00000042345 2721 Ctnna1 0.24362694372650343

ENSMUST00000047490 2652 Ndst2 0.2658215570728855

ENSMUST00000037260 1500 Zfp623 0.25269059792472104

ENSMUST00000048807 621 Mis12 0.22929030301148226

ENSMUST00000039152 3735 Dhx8 0.2862295605067729

ENSMUST00000040638 1305 Pbx3 0.24687487961857013

ENSMUST00000031121 1659 Gabra4 0.18991897560218027

ENSMUST00000034214 186 Mt2 0.39031391335905946

ENSMUST00000044123 1149 Trhr2 0.3493605078160837

ENSMUST00000037877 1470 Tcfl5 0.2406942716816633

ENSMUST00000046533 810 Prr7 0.3090915754696741

ENSMUST00000039144 2940 Clstn1 0.3275124205610442

ENSMUST00000039318 1458 Cdc14b 0.21702490610009179

ENSMUST00000043937 450 Ostc 0.203148655982388

ENSMUST00000034359 933 Tradd 0.2851809885323029

ENSMUST00000049681 1485 Itgbl1 0.2614292284847557

ENSMUST00000032704 666 Faap24 0.22620856063730496

ENSMUST00000034232 312 Ccl17 0.32201801549968057

ENSMUST00000033544 876 Brcc3 0.17563231687631303

ENSMUST00000047997 1131 Sp6 0.28088530643047355

ENSMUST00000043160 4446 Aqr 0.2240694171231493

ENSMUST00000037246 825 Ccs 0.2601798354305718

ENSMUST00000035471 9252 Lama1 0.27139547494238414

ENSMUST00000043839 903 F11r 0.25638139687188216

ENSMUST00000037998 1113 Tram2 0.2828386555185141

ENSMUST00000034615 1446 Pus3 0.16526400470642189

ENSMUST00000035300 288 Scgb1c1 0.30025645018707625

ENSMUST00000038695 1500 Kcna2 0.25906549061385836

ENSMUST00000036288 3408 R3hdm1 0.19723215085395904

ENSMUST00000040790 4698 Slx4 0.2544234480004495

ENSMUST00000039666 1425 Plekhs1 0.2134702342710056

ENSMUST00000035288 7680 Stab2 0.2584141856692251

ENSMUST00000033063 912 Cd37 0.3153804192704595

ENSMUST00000030768 1062 Kcnab2 0.30823235676077826

ENSMUST00000032648 645 4933421I07Rik 0.25978990898686405

ENSMUST00000046322 1062 Fcrla 0.24361051094592626

ENSMUST00000033483 1884 Ccdc22 0.2874512316277136

ENSMUST00000034249 582 Cfap20 0.2850664427884292

ENSMUST00000031446 3237 Tmem132b 0.30911839493246757

ENSMUST00000045593 2010 Daglb 0.2971850085389869

ENSMUST00000032427 2199 Asun 0.18408983920717303

ENSMUST00000037906 936 Tmem177 0.238776111590869

ENSMUST00000048112 3282 Sgsm1 0.3662745269417007

ENSMUST00000044622 7719 Epg5 0.2416469217274467

ENSMUST00000030243 1659 Prkaa2 0.2274389818743623

ENSMUST00000043911 873 A230050P20Rik 0.356885555299628

ENSMUST00000048073 1668 Pigs 0.300767760063404

ENSMUST00000039562 1224 Trim13 0.2023145993140482

ENSMUST00000035672 5265 Ppl 0.33245389065890074

ENSMUST00000037901 4014 Trpm3 0.26394909620734014

ENSMUST00000030947 1329 Mxra8 0.30545942722393593

ENSMUST00000040562 2367 Cdh10 0.19398234359471955

ENSMUST00000033715 1089 Nsdhl 0.24707961607516268

ENSMUST00000043269 1392 Hnrnpk 0.18229942374980115

ENSMUST00000047846 3297 Catsperg1 0.2531446089654602

ENSMUST00000031521 1482 Cyp2w1 0.326062626735634

ENSMUST00000043815 6027 Nup205 0.19829886598818075

ENSMUST00000034428 354 Gabarapl2 0.29680613613115014

ENSMUST00000032667 1404 Siglece 0.26463841274640726

ENSMUST00000033886 414 Ggn 0.25678860827648625

ENSMUST00000043970 3381 Nutm1 0.1968638007118542

ENSMUST00000036952 780 9530068E07Rik 0.30088707728260966

ENSMUST00000031099 654 Grpel1 0.29411851741698725

ENSMUST00000045174 366 Ypel5 0.2079840084408471

ENSMUST00000035797 783 Rab26 0.305879428300555

ENSMUST00000047257 1467 Katnal1 0.2591510807872827

ENSMUST00000046656 1263 Tasp1 0.2015380825633172

ENSMUST00000045918 1317 Alg3 0.27861935120884157

ENSMUST00000034929 1656 Lactb 0.2180221696507692

ENSMUST00000033070 1377 Kat8 0.30018348608133755

ENSMUST00000032726 693 Tm2d3 0.29461802066484505

ENSMUST00000043148 219 Gng12 0.3002366523099679

ENSMUST00000038690 1266 AW209491 0.2152535054296409

ENSMUST00000047362 1134 Rccd1 0.26277330271449123

ENSMUST00000049449 1080 Ptpn7 0.3244081953254329

ENSMUST00000030893 1035 Dffb 0.33635146595285675

ENSMUST00000044415 1575 Npepl1 0.3334493186696476

ENSMUST00000030125 1068 Bag1 0.23141718807189343

ENSMUST00000032840 576 Mrps11 0.2677458562363741

ENSMUST00000030408 1605 Mfsd2a 0.29228604685346843

ENSMUST00000035608 972 Olig2 0.32380953483594715

ENSMUST00000034534 1323 Ets1 0.3016980952553564

ENSMUST00000048010 2877 Dse 0.23215747320570726

ENSMUST00000039680 996 Parp11 0.2639511652253783

ENSMUST00000033664 1110 Il2rg 0.21866795390220506

ENSMUST00000042227 1677 D11Wsu47e 0.20570124781186816

ENSMUST00000043058 1257 Serpina3k 0.2625858793881179

ENSMUST00000034512 849 Oaf 0.3149377786732908

ENSMUST00000040924 1257 Ss18 0.2656968624640382

ENSMUST00000037843 531 Ubald1 0.34489195544044565

ENSMUST00000038932 873 Odf4 0.282515466699744

ENSMUST00000035250 951 Olfr720 0.2408279148001949

ENSMUST00000036862 2490 Cog5 0.16567443682361127

ENSMUST00000036467 1362 Asb16 0.2971518961982648

ENSMUST00000047903 1854 Colgalt1 0.33169687063262926

ENSMUST00000030714 2319 Sema3a 0.20554181334993457

ENSMUST00000038336 12741 Pkhd1l1 0.16037107605774858

ENSMUST00000033720 1278 Rbbp7 0.18793452119923393

ENSMUST00000037687 504 Tmem35 0.29393094388555796

ENSMUST00000045909 687 Zbed3 0.37122038577046157

ENSMUST00000037915 1851 Msl1 0.23690400118431187

ENSMUST00000032371 2115 Gys2 0.23886939837246202

ENSMUST00000036928 1368 Ephx1 0.33573946506255653

ENSMUST00000037763 4338 Ythdc2 0.1545936610481353

ENSMUST00000031094 2085 Tbc1d14 0.23429728519185997

ENSMUST00000042917 4719 Rims2 0.18771226848279476

ENSMUST00000042190 6468 Tecta 0.3035803757055606

ENSMUST00000048945 786 Klk1b26 0.2625368167531536

ENSMUST00000039156 375 Phpt1 0.2634728620931424

ENSMUST00000039205 1029 Galm 0.2522418476497471

ENSMUST00000037145 3927 Cdhr2 0.3096252071358648

ENSMUST00000034511 1764 Trim29 0.37251381179214027

ENSMUST00000030192 492 Hint2 0.23941455079580654

ENSMUST00000040090 960 Nubpl 0.15117536049750202

ENSMUST00000037942 2739 Ttll7 0.2047961883474617

ENSMUST00000044332 1596 Cerk 0.30423715165864995

ENSMUST00000039048 1179 1810055G02Rik 0.26127480284296345

ENSMUST00000036541 540 Arl5a 0.17866919901721603

ENSMUST00000039733 2274 Osbpl11 0.2338788582455759

ENSMUST00000038281 288 Dexi 0.3273258176306238

ENSMUST00000036497 1251 Prkar2b 0.23648047648491557

ENSMUST00000031591 1209 Lhx5 0.30029391569488545

ENSMUST00000034279 3672 Gse1 0.33314680964340104

ENSMUST00000044478 1158 Hibch 0.16393004516801088

ENSMUST00000031429 1167 P2rx4 0.3112279055596476

ENSMUST00000030851 168 Tomm7 0.2506646256183056

ENSMUST00000042503 2529 Usp6nl 0.1993849235531698

ENSMUST00000040421 984 Coq5 0.2613944095970464

ENSMUST00000048482 375 2010003K11Rik 0.3203193676943752

ENSMUST00000033731 1500 Zfp275 0.30291478012370865

ENSMUST00000030795 1887 Abcf2 0.2970011017172789

ENSMUST00000044705 858 Vstm2b 0.2687431651155316

ENSMUST00000032416 720 Cidec 0.3932657580154118

ENSMUST00000033553 3474 Heph 0.2178183635465087

ENSMUST00000044804 1686 Cdsn 0.2711709318616377

ENSMUST00000041197 1068 Abhd4 0.28638318030535714

ENSMUST00000040872 2547 Lpin3 0.3079476984881748

ENSMUST00000033913 573 Dctn6 0.25507596885251943

ENSMUST00000031640 1395 Cdk8 0.1860971323057079

ENSMUST00000034588 795 Apoa1 0.35909471976530544

ENSMUST00000034527 1677 Nxpe2 0.23110492663708304

ENSMUST00000031540 1536 Oasl1 0.35665093117240315

ENSMUST00000030011 1866 Stra6l 0.3194303914783855

ENSMUST00000036570 2124 Appl1 0.14743318909053013

ENSMUST00000033463 1935 Slc9a9 0.240374612935202

ENSMUST00000032252 699 Klrk1 0.17583191590511899

ENSMUST00000044009 1158 Camk1d 0.24673079746144075

ENSMUST00000042771 4050 Sbno2 0.34029672172422765

ENSMUST00000031420 855 Gpn3 0.35296387797820067

ENSMUST00000030872 1308 Orc5 0.1615977964165078

ENSMUST00000037472 2358 Leng8 0.3216794021818258

ENSMUST00000030637 2190 Ncdn 0.31063614933761907

ENSMUST00000035538 6198 Wnk2 0.31374809954867583

ENSMUST00000033058 5619 Sbf2 0.18946789632035355

ENSMUST00000030903 1776 Atad3a 0.2721367348336517

ENSMUST00000049614 870 B430306N03Rik 0.2902218433329368

ENSMUST00000046461 978 Dok4 0.35916621313367747

ENSMUST00000032710 1749 Slc17a6 0.22250267743945978

ENSMUST00000046351 1881 Lrfn3 0.2683783586606033

ENSMUST00000045521 1851 Dtx4 0.29054686243625977

ENSMUST00000043680 1356 Tubg1 0.3696825300916349

ENSMUST00000047037 4785 Thoc2 0.14685131603052984

ENSMUST00000034141 2559 Lonp2 0.21192568131633388

ENSMUST00000041012 621 Ptcra 0.31096582888434965

ENSMUST00000040434 1026 Tbcc 0.3606206266720198

ENSMUST00000044684 3210 Helq 0.20929581589068685

ENSMUST00000041123 1971 Muc20 0.22233495915913506

ENSMUST00000030784 1701 Prkag2 0.24878463146528168

ENSMUST00000046191 555 Gnpnat1 0.15450613998229254

ENSMUST00000045738 1578 Slc32a1 0.3969841158759161

ENSMUST00000036181 1014 Car14 0.26727027026206607

ENSMUST00000032946 627 Rab6a 0.19342091713806392

ENSMUST00000037376 1032 Nagk 0.27963275238584134

ENSMUST00000043338 480 Sft2d2 0.2722610039631469

ENSMUST00000048605 402 Il5 0.22986215590676445

ENSMUST00000044608 3705 Jarid2 0.27364207622624054

ENSMUST00000033610 1692 Nox1 0.213248991215705

ENSMUST00000045898 1110 Pcyt1b 0.22925430169804473

ENSMUST00000032182 2793 Xpc 0.2912088077088176

ENSMUST00000046506 678 Clcf1 0.31064254860604257

ENSMUST00000049185 495 Ybey 0.2697779876749988

ENSMUST00000039909 279 Fxyd1 0.32317874682555575

ENSMUST00000034900 495 Ooep 0.22458167165181886

ENSMUST00000046206 939 Rprd1a 0.16873367130526568

ENSMUST00000040700 6342 Dock9 0.2778037380321569

ENSMUST00000050004 423 Rnf125 0.19476052764639754

ENSMUST00000033825 1062 Adprhl1 0.28755500208924495

ENSMUST00000034375 1482 Dus2 0.24445819559020418

ENSMUST00000047753 3831 Abcb1a 0.21287882200683644

ENSMUST00000034547 1275 Acat1 0.20133077945370673

ENSMUST00000043550 1542 Traf3ip3 0.2749541197481215

ENSMUST00000033184 1689 Tpp1 0.23598397547959524

ENSMUST00000050044 999 Olfr1324 0.1932955195722473

ENSMUST00000048374 2724 C330027C09Rik 0.13471108054396877

ENSMUST00000031262 1125 Coq2 0.22649467682990712

ENSMUST00000044000 1725 4930447C04Rik 0.1236564296194918

ENSMUST00000038498 1374 Bag4 0.22100950128357405

ENSMUST00000031011 666 Rab28 0.17622497733917297

ENSMUST00000048493 4230 Rpap1 0.2755693167375044

ENSMUST00000033695 2259 Abcb7 0.15758806028404895

ENSMUST00000043045 1083 Wdr45 0.316302066390146

ENSMUST00000046470 1182 Mettl22 0.31361836691902706

ENSMUST00000043374 495 Ppp1r14c 0.2582461498777392

ENSMUST00000036060 1050 Isl1 0.25410653667052496

ENSMUST00000035346 2040 Nol4l 0.3663344541907546

ENSMUST00000030138 3459 Nol6 0.29543589315919366

ENSMUST00000037481 810 Clec1a 0.2752255529840702

ENSMUST00000038844 489 Ubd 0.2631901196668517

ENSMUST00000033539 6960 F8 0.15628389984846733

ENSMUST00000034057 3342 Palld 0.25238273029815333

ENSMUST00000033583 1107 Magt1 0.18201664303876344

ENSMUST00000030841 1761 Klhl7 0.19642099283934275

ENSMUST00000031562 1620 Zfp513 0.29078262417957923

ENSMUST00000041901 564 Cib2 0.34104625320929305

ENSMUST00000034205 1014 Cenpn 0.2151026490971543

ENSMUST00000048718 1248 Mmaa 0.20061287385353357

ENSMUST00000044083 4293 Dapk1 0.2828136746474762

ENSMUST00000033310 9534 Mki67 0.1805645385240164

ENSMUST00000035237 2889 Usp4 0.2644302551291746

ENSMUST00000046614 945 Gipc2 0.22579133425262438

ENSMUST00000049130 390 Bex2 0.31381998854708515

ENSMUST00000043458 765 Srd5a2 0.26791773784078887

ENSMUST00000031787 1251 Evx1 0.320998857605046

ENSMUST00000030834 3609 Nos3 0.31999370495967056

ENSMUST00000047315 1380 Foxa2 0.3734698813659876

ENSMUST00000035724 4215 Akna 0.2787992725012824

ENSMUST00000034392 543 Nip7 0.2895541506530007

ENSMUST00000030971 3933 Adgra3 0.2324611278028884

ENSMUST00000049378 2049 Ablim3 0.313576435562018

ENSMUST00000048976 2076 Gucy1a3 0.26857462115865116

ENSMUST00000044976 891 Glyat 0.19659912377420036

ENSMUST00000038648 1887 Nek11 0.2203867704128116

ENSMUST00000041804 4416 Lmtk2 0.2419870668728329

ENSMUST00000042166 360 Fam103a1 0.22964641609838965

ENSMUST00000038101 1194 Hrh2 0.3055556888066247

ENSMUST00000031081 1410 Pi4k2b 0.21287152208701668

ENSMUST00000042641 2115 Zfp60 0.20034846199716397

ENSMUST00000034717 2532 Kank2 0.33202283778401265

ENSMUST00000049931 1188 Spn 0.22393410462162194

ENSMUST00000045042 834 Batf2 0.2956358963779642

ENSMUST00000045529 1191 Kiss1r 0.32159727725668596

ENSMUST00000031859 3156 Trim24 0.19023005551438724

ENSMUST00000031736 1440 Agfg2 0.2768086811231805

ENSMUST00000033608 2022 Sytl4 0.24194358735389634

ENSMUST00000037828 927 Ldlrap1 0.3320077835269351

ENSMUST00000041659 744 Prrx2 0.28822895751176425

ENSMUST00000048953 3501 Atp2b4 0.26282994303185764

ENSMUST00000045931 1437 Zfp410 0.20431843476601935

ENSMUST00000030939 1320 Nadk 0.30471922538922475

ENSMUST00000033156 549 Dctn5 0.21150702597731738

ENSMUST00000038422 2328 Irf2bpl 0.2684947876369295

ENSMUST00000031378 870 Stx2 0.3045963930768763

ENSMUST00000039271 2010 2610008E11Rik 0.19156638771649376

ENSMUST00000047945 702 Samt3 0.18435694705582478

ENSMUST00000030489 990 Tal1 0.29884411164553826

ENSMUST00000032912 900 Qprt 0.28926787617300953

ENSMUST00000034342 459 Cklf 0.1801000539945579

ENSMUST00000038614 360 Ypel3 0.3423736888329978

ENSMUST00000033187 1728 Cnga4 0.3111767133887029

ENSMUST00000033006 843 Nsmce1 0.2905180854511725

ENSMUST00000032561 1128 Vasp 0.3038982727797774

ENSMUST00000043094 942 Itln1 0.2956873049720916

ENSMUST00000034822 2166 Acsbg1 0.3325520240973499

ENSMUST00000036991 591 Hspb8 0.2792314197308506

ENSMUST00000038163 1293 Pnmal1 0.2554410397958043

ENSMUST00000031419 756 Fam216a 0.23418463216004023

ENSMUST00000032909 3300 Pde3b 0.19276366086597918

ENSMUST00000033741 1110 Bgn 0.32876522292772126

ENSMUST00000032336 1653 Plbd1 0.28288057834638136

ENSMUST00000032539 2070 Slc27a5 0.23862932343060586

ENSMUST00000030313 1071 Caap1 0.18842697415729304

ENSMUST00000047923 3099 Sec24d 0.25640522729698145

ENSMUST00000040833 1734 Edem2 0.30214190195872764

ENSMUST00000038383 951 Akr1b10 0.3246212307109179

ENSMUST00000034955 927 Spg21 0.22226747574820835

ENSMUST00000030212 1059 Sh3gl2 0.26916743287879036

ENSMUST00000041160 207 Gng10 0.3808481962096676

ENSMUST00000031913 741 Try4 0.34800117226882005

ENSMUST00000044423 3525 Brip1 0.17324137187457722

ENSMUST00000046422 1266 Acot5 0.2958760190674036

ENSMUST00000039340 1413 Nme8 0.15675937323067696

ENSMUST00000030879 2613 Clcn6 0.2919266917373641

ENSMUST00000039620 834 Cbr3 0.27917494844282

ENSMUST00000032217 1566 Lag3 0.2600770558388543

ENSMUST00000032673 1443 Zfp94 0.2764179971055725

ENSMUST00000031224 2553 Tgfbr3 0.2697443553436367

ENSMUST00000030848 405 Rbp7 0.3041262715382855

ENSMUST00000038409 1620 Podxl2 0.3250489406512547

ENSMUST00000032234 3366 Cd163 0.2114896897493409

ENSMUST00000030477 651 Mob3c 0.36880343987509684

ENSMUST00000044616 2988 Ints8 0.1565929013511977

ENSMUST00000045550 1206 Xkr8 0.3215151061587951

ENSMUST00000042065 2880 Map3k13 0.27761034045554284

ENSMUST00000036300 5538 Col27a1 0.25615789109554254

ENSMUST00000034132 789 Orc6 0.16857224123758882

ENSMUST00000033692 513 Zcchc13 0.2908331266852238

ENSMUST00000031167 3525 Polr2b 0.19489999030053892

ENSMUST00000032865 1260 Fah 0.26211015915445157

ENSMUST00000033044 1401 BC017158 0.2667547255115502

ENSMUST00000048687 315 Wbp5 0.27032436505776003

ENSMUST00000034866 1002 Etfa 0.14304458497425618

ENSMUST00000034594 1728 Il10ra 0.2618229427168402

ENSMUST00000031243 885 Spp1 0.2497005354378646

ENSMUST00000032321 987 Bcl2l14 0.2861488769931505

ENSMUST00000037375 1803 Eps8l3 0.29442914823326194

ENSMUST00000034173 1689 Ces1e 0.2297200897592709

ENSMUST00000044567 975 Alg5 0.19538849146857798

ENSMUST00000040234 1383 Tsen2 0.29522081562276886

ENSMUST00000041591 2589 Enpp2 0.20804771597256114

ENSMUST00000047875 2175 Eef2k 0.31712562124710575

ENSMUST00000039431 2478 Ntrk3 0.30882399857381826

ENSMUST00000045295 4059 Pnpla7 0.2700897754601245

ENSMUST00000034264 774 Pgls 0.3246975973927944

ENSMUST00000042954 2601 Poln 0.17743434058991484

ENSMUST00000033898 5010 Col4a1 0.2066744056521815

ENSMUST00000033394 618 Fadd 0.3484138518445684

ENSMUST00000049095 1740 Faah 0.3158705722775831

ENSMUST00000045247 1296 Wdr18 0.35935191339414607

ENSMUST00000030289 2355 Usp1 0.17029199780639284

ENSMUST00000035158 2094 Trf 0.27898131567671325

ENSMUST00000032902 393 Calcb 0.3713993843719812

ENSMUST00000030345 1977 Cpt2 0.26415092510672566

ENSMUST00000040465 2829 Clca2 0.21209388268268914

ENSMUST00000046638 1803 Mettl25 0.20733318598944683

ENSMUST00000035606 6597 Ascc3 0.15413128613200514

ENSMUST00000031082 1341 Pi4k2b 0.2239941729960305

ENSMUST00000033000 1590 Il21r 0.28503482658444024

ENSMUST00000049093 1191 Txnip 0.296325081645691

ENSMUST00000037499 501 Immp1l 0.14251359641970543

ENSMUST00000049285 2151 Lrrn1 0.228721048839139

ENSMUST00000030269 1902 Slc6a9 0.34554250398333564

ENSMUST00000042135 2409 Rasa4 0.3202779902180774

ENSMUST00000033139 1551 Ate1 0.20454709026703435

ENSMUST00000040496 1239 Bmp8a 0.3127273356383275

ENSMUST00000050073 1014 Dnajb4 0.17037121059353294

ENSMUST00000034623 660 Trappc4 0.2819820681185152

ENSMUST00000034414 948 4931406C07Rik 0.19480622784004395

ENSMUST00000046627 1776 Osbpl10 0.31968083562795946

ENSMUST00000032555 1086 Tomm40 0.30918349050132976

ENSMUST00000035812 1134 Ttl 0.27909123052097995

ENSMUST00000045110 2784 Dstyk 0.265382233796511

ENSMUST00000034881 252 Cox7a2 0.2443432416164835

ENSMUST00000030687 798 Rspo1 0.37166750894918205

ENSMUST00000035323 804 Spib 0.32826920491312994

ENSMUST00000030221 699 Hacd4 0.19935155057629395

ENSMUST00000045884 1755 Klhdc4 0.30347394249113147

ENSMUST00000035026 2172 Trim42 0.37006321377241996

ENSMUST00000037349 1839 Aifm1 0.19468695318885745

ENSMUST00000031145 723 Pdcl2 0.14916029407437675

ENSMUST00000047099 1953 Pif1 0.28586712994662195

ENSMUST00000031377 1437 Scarb2 0.22811765747511903

ENSMUST00000040416 1437 Ddx19a 0.30950917857216964

ENSMUST00000031810 1122 Cep41 0.24875850905837063

ENSMUST00000040324 3492 Ppp1r26 0.29453024764426133

ENSMUST00000038280 2034 Fermt1 0.2261988959419793

ENSMUST00000037796 1263 Gmppa 0.28269095847721293

ENSMUST00000034185 1320 Irx6 0.30464448058500915

ENSMUST00000031866 357 Mtpn 0.22655377079756653

ENSMUST00000042220 4212 Naip6 0.20273804938597292

ENSMUST00000033335 639 Akip1 0.24050056585404142

ENSMUST00000045993 1221 Cops4 0.19431376319308868

ENSMUST00000036805 495 Plekhj1 0.3878117193874934

ENSMUST00000037788 2463 Pomt2 0.2657564884347971

ENSMUST00000049412 1458 Stau1 0.23402476978375392

ENSMUST00000037397 3312 Cep126 0.14576941828861753

ENSMUST00000032114 2505 Mogs 0.25476624410783427

ENSMUST00000043062 2004 Acsbg2 0.308505029401376

ENSMUST00000035661 6984 Cspg4 0.29842502776005836

ENSMUST00000030142 1584 Epb4.1l4b 0.2569090780111424

ENSMUST00000030453 840 Mmachc 0.22982914251614056

ENSMUST00000050120 780 Kctd4 0.18518662829702726

ENSMUST00000048455 1770 Ntng2 0.3755010616494746

ENSMUST00000033826 885 Atp4b 0.3569402855595902

ENSMUST00000033325 1758 Swap70 0.26372151143906525

ENSMUST00000041761 1149 Prss23 0.28914235712671105

ENSMUST00000038537 1197 Wtip 0.3164861263812323

ENSMUST00000033805 696 Psmd10 0.1940638332866611

ENSMUST00000043214 579 Rac2 0.3627582400854799

ENSMUST00000033241 813 Lhpp 0.30995741968307866

ENSMUST00000035094 1107 Exog 0.2453793387413718

ENSMUST00000037739 1617 Gnl3 0.16378985840632515

ENSMUST00000034076 582 Cbln1 0.37846724255088715

ENSMUST00000042235 1389 Eef1a1 0.25531548965716727

ENSMUST00000040177 879 Znrd1as 0.18707688285696378

ENSMUST00000036917 651 Hand1 0.31490414145188766

ENSMUST00000033185 1383 Hpx 0.2513227087134723

ENSMUST00000037865 2571 Atp6v0a2 0.31096422080586666

ENSMUST00000030446 1104 Urod 0.22527439034788263

ENSMUST00000032071 966 Dusp11 0.17993100942647364

ENSMUST00000042390 1977 Man1b1 0.2879951008671462

ENSMUST00000035048 795 Cldn18 0.34075481804467495

ENSMUST00000048892 855 Icmt 0.3067783010110714

ENSMUST00000047760 795 Spsb2 0.31569631129213116

ENSMUST00000033723 1098 Syap1 0.1989326502540121

ENSMUST00000048988 5730 Znfx1 0.3179554709261161

ENSMUST00000049245 1167 Rbmxl1 0.17529944999756883

ENSMUST00000034533 1260 Kcnj5 0.3016555751524663

ENSMUST00000049004 2901 Anpep 0.3223617744247981

ENSMUST00000049312 834 Tmem55b 0.22948077171021047

ENSMUST00000038229 990 Neil2 0.26034411302810945

ENSMUST00000031750 4746 Arhgef5 0.22026409764223848

ENSMUST00000044392 1263 Ccbl2 0.20114016164460646

ENSMUST00000033388 456 Oraov1 0.27352080661321043

ENSMUST00000036116 660 Ttc9 0.2671836567014367

ENSMUST00000031311 783 Dck 0.20646140472720617

ENSMUST00000046746 849 Igfbp7 0.3073888557129408

ENSMUST00000038185 1953 Exd2 0.24200254465914492

ENSMUST00000045068 477 Cplx3 0.3632464808673662

ENSMUST00000033770 1506 Mecp2 0.2796891434919278

ENSMUST00000035444 1383 Chrm1 0.3436976555755614

ENSMUST00000033149 2295 Cpxm2 0.2907654623468353

ENSMUST00000031069 1515 Sepsecs 0.19940752910087242

ENSMUST00000043814 2724 Fig4 0.2248593241547596

ENSMUST00000034064 1875 F11 0.21319090199583227

ENSMUST00000037709 1698 Tm7sf3 0.25673219312408174

ENSMUST00000049567 1875 Keap1 0.36744561361497097

ENSMUST00000048061 576 1700031F05Rik 0.318673645861233

ENSMUST00000030561 1065 Gnat3 0.1969054004666557

ENSMUST00000033983 891 Mak16 0.2175359840373278

ENSMUST00000032129 555 Gkn1 0.3062079044290174

ENSMUST00000046835 3498 Fnip1 0.16427111912799974

ENSMUST00000049271 1263 4930427A07Rik 0.28469905311394195

ENSMUST00000047399 2727 Adgrf1 0.21222032514224767

ENSMUST00000034562 528 Cryab 0.35580801676239193

ENSMUST00000048675 948 Magea4 0.21252485585963954

ENSMUST00000041614 2280 Ttc13 0.2583593097216423

ENSMUST00000038874 1821 Scai 0.18911635582393313

ENSMUST00000044691 633 Bag2 0.27861940676064584

ENSMUST00000037146 1035 Gas2l1 0.31755512189703605

ENSMUST00000030973 2430 Prom1 0.2304209468023657

ENSMUST00000031850 2370 Zc3hav1 0.251457180894551

ENSMUST00000030340 1644 Scp2 0.2269638892838059

ENSMUST00000030757 2154 Fbxo42 0.2339744504589892

ENSMUST00000042942 2997 Sec23ip 0.19742610003415312

ENSMUST00000039769 1614 Sntb1 0.26095018723116237

ENSMUST00000031897 681 Gstk1 0.23707019130081947

ENSMUST00000029922 855 Fhl5 0.26303760297933176

ENSMUST00000045085 3012 Grin3b 0.3179943357875995

ENSMUST00000047929 1722 Acsm1 0.27142715875379225

ENSMUST00000041758 543 Cypt2 0.19329222793259612

ENSMUST00000034909 537 Rasgrf1 0.2701724560765277

ENSMUST00000030069 990 Ptgr1 0.22283169546036655

ENSMUST00000031117 831 Gnpda2 0.1671256630455402

ENSMUST00000046233 1164 Bbox1 0.2117546284069122

ENSMUST00000030858 888 Fbxo6 0.30330137063109747

ENSMUST00000042450 1851 Rmi1 0.1362170690053774

ENSMUST00000036691 4461 Prrc2b 0.29525647231832075

ENSMUST00000033289 2058 Stim1 0.31075175263550114

ENSMUST00000040428 297 Rnasek 0.2658946445917933

ENSMUST00000036357 960 Olfr1347 0.27514296874027827

ENSMUST00000044503 1665 Rftn1 0.29053694264072

ENSMUST00000034980 7926 Atr 0.14260726383085753

ENSMUST00000031314 1827 Alb 0.24779566254748356

ENSMUST00000039798 2643 Bbs9 0.20357715495588563

ENSMUST00000037696 2463 Svs1 0.290807202977063

ENSMUST00000034961 2382 Igdcc3 0.28811620049361464

ENSMUST00000046549 675 Apobec2 0.3307062437247958

ENSMUST00000041826 1146 Rnf13 0.18856754645823284

ENSMUST00000040489 6087 Trpm6 0.22082430235942468

ENSMUST00000031341 345 Cdk2ap1 0.25475125955842826

ENSMUST00000049784 3564 Myt1l 0.2673738386022862

ENSMUST00000040504 2154 Klhl4 0.16611828805400347

ENSMUST00000037096 2022 Cnbd2 0.26255724934127045

ENSMUST00000036439 2373 Cdh6 0.258676683748493

ENSMUST00000040717 4164 Kif15 0.17978145092257364

ENSMUST00000033386 1032 Mrgprf 0.35358406157384104

ENSMUST00000045617 1608 Hpse 0.22488271757347636

ENSMUST00000033730 1155 Grpr 0.305234940965294

ENSMUST00000031411 1560 Aldh2 0.346463375357123

ENSMUST00000039303 1149 Npy1r 0.2685160301145551

ENSMUST00000047611 903 Nthl1 0.29707708593749066

ENSMUST00000030726 1305 Rcc1 0.2739176777080114

ENSMUST00000032994 1587 Spns1 0.2836585511619497

ENSMUST00000034058 711 Cbr4 0.1841609416155589

ENSMUST00000035563 708 Tspan8 0.20612858818227447

ENSMUST00000037814 471 Cmtm5 0.3450789202203507

ENSMUST00000035258 681 Ms4a4b 0.14512486074508785

ENSMUST00000044766 3540 4930402H24Rik 0.23280261131494773

ENSMUST00000044297 813 Igfbpl1 0.2901717446552233

ENSMUST00000035010 2472 Stt3b 0.2178790772685194

ENSMUST00000035079 2283 Mlh1 0.22155595616656615

ENSMUST00000032143 1827 Rpn1 0.28932896499302374

ENSMUST00000043379 429 Cysrt1 0.2694549680656317

ENSMUST00000046893 1767 Gpr162 0.28125409713490157

ENSMUST00000045802 333 2810417H13Rik 0.20888877929935584

ENSMUST00000040455 1050 Hif1an 0.2552947485742219

ENSMUST00000042456 981 B3galt1 0.26488592417635315

ENSMUST00000039412 2469 Mcph1 0.21774224437036283

ENSMUST00000036734 669 Gadd45gip1 0.2774311901583554

ENSMUST00000048103 708 Bpifa2 0.1551201950672245

ENSMUST00000038445 1452 Mybph 0.31426545051535637

ENSMUST00000030808 771 Tnfrsf9 0.2814041742269683

ENSMUST00000033098 1182 Bcat2 0.29500141098980687

ENSMUST00000047321 1881 Arntl 0.20104877078666042

ENSMUST00000046174 624 Cldn11 0.3263350898700323

ENSMUST00000034111 1023 Slc10a7 0.23238003161364035

ENSMUST00000049355 2163 Mapk6 0.25180047793459015

ENSMUST00000031901 2172 Trpv5 0.25089724979783473

ENSMUST00000031627 546 Pdap1 0.23946640948598838

ENSMUST00000035689 693 4932438H23Rik 0.25175944346506396

ENSMUST00000033905 1248 Ankrd10 0.2642417651850212

ENSMUST00000029919 2742 Clca1 0.22513916228149916

ENSMUST00000046633 885 AW549877 0.18445106848072912

ENSMUST00000037324 492 Skp1a 0.17619571996127578

ENSMUST00000033269 1338 Ctbp2 0.2931648727814635

ENSMUST00000031817 3012 Herc6 0.16924768653975114

ENSMUST00000040056 2649 Ppfibp2 0.24121400910380592

ENSMUST00000032501 762 Tspan11 0.3772043976504482

ENSMUST00000044220 321 Cmc1 0.20997413667000764

ENSMUST00000036753 2043 Mid1 0.2973687341275207

ENSMUST00000033941 1680 Plat 0.2877469485007326

ENSMUST00000032760 732 Mesp1 0.302919458745446

ENSMUST00000031029 1413 Snx17 0.2674278395539615

ENSMUST00000041860 1899 Gdpd4 0.1808546602286717

ENSMUST00000036426 1230 Prss35 0.2555997735447193

ENSMUST00000039449 5304 Ltn1 0.17419177518505743

ENSMUST00000038791 996 Gde1 0.2977111061796798

ENSMUST00000048691 2706 Ptchd4 0.254308517972795

ENSMUST00000031229 1410 Rufy3 0.21728936144787458

ENSMUST00000038890 5610 Dennd4a 0.17329297572400112

ENSMUST00000030765 2022 Padi2 0.37935998926951137

ENSMUST00000046745 1248 Tgtp2 0.2072011742587189

ENSMUST00000035279 2016 Hps4 0.3175870103085767

ENSMUST00000035800 2112 Nfatc1 0.3125000890299643

ENSMUST00000035112 4362 Nktr 0.20503365698535517

ENSMUST00000043074 402 Fau 0.30593903035840453

ENSMUST00000050211 813 Tk2 0.25230654026632554

ENSMUST00000044216 1035 Shd 0.34114395812635007

ENSMUST00000042438 4173 Plekhg1 0.24066197648546736

ENSMUST00000047404 1572 Dync1li1 0.21377482404153889

ENSMUST00000039507 816 Oscar 0.2578021497265984

ENSMUST00000031093 1311 Cckar 0.3123414360647838

ENSMUST00000037540 1122 P2ry2 0.32288197176350153

ENSMUST00000041638 2649 Gtf3c3 0.16865401001672542

ENSMUST00000049863 1182 Pofut1 0.3381598374003914

ENSMUST00000044911 915 Stub1 0.36086896017700165

ENSMUST00000046589 426 Cst13 0.26201349426439896

ENSMUST00000033625 486 4930513O06Rik 0.18626330486009626

ENSMUST00000034228 492 Arl2bp 0.26991247455399164

ENSMUST00000042702 744 Fam173b 0.24855916653849924

ENSMUST00000040359 1722 Arsi 0.3145972699214754

ENSMUST00000046255 1248 Gpr19 0.3334859762375785

ENSMUST00000038188 2706 Limch1 0.27372217374456403

ENSMUST00000037235 1743 Xkr7 0.3363424257233004

ENSMUST00000034740 2664 Nedd4 0.21315947764621895

ENSMUST00000039601 372 Snrnp25 0.28326878498355984

ENSMUST00000049454 2160 Six5 0.2834086947196868

ENSMUST00000041965 5142 Cdc42bpb 0.30521114910222685

ENSMUST00000034159 450 Txnl4b 0.232023793967321

ENSMUST00000030124 327 Tal2 0.28961086519464263

ENSMUST00000036503 1845 Zfand4 0.18345446164700863

ENSMUST00000032815 1080 Nfkbib 0.258627019878363

ENSMUST00000032978 2049 Sh2b1 0.24663973051333138

ENSMUST00000047309 621 Nat14 0.3178199564870195

ENSMUST00000037268 459 1700106J16Rik 0.34155849107818065

ENSMUST00000032958 927 Ucp3 0.32082796138775227

ENSMUST00000042055 972 Ppp2r4 0.3304193546670946

ENSMUST00000032469 1665 Mbd4 0.18518435257446605

ENSMUST00000033866 1161 Vps36 0.2274719206087733

ENSMUST00000050148 1008 Cdc37l1 0.19218455943223461

ENSMUST00000047282 1062 Mthfsd 0.27166944475211907

ENSMUST00000049138 618 2410131K14Rik 0.3303746807322981

ENSMUST00000032172 822 Chchd6 0.29799571559181126

ENSMUST00000048150 4902 Cc2d2a 0.20955092694263833

ENSMUST00000037372 2814 Nlrp4f 0.20230947404750976

ENSMUST00000035775 312 Lsm7 0.4049203980518534

ENSMUST00000044382 675 Zc4h2 0.2614173608985175

ENSMUST00000039047 1218 Serpini2 0.15172445839096427

ENSMUST00000039442 654 Alg14 0.23452420477418148

ENSMUST00000038896 1317 Lcat 0.31338245838668727

ENSMUST00000034393 720 Tmed6 0.2497750502226458

ENSMUST00000045560 1467 Slc19a3 0.21744466845437999

ENSMUST00000037983 354 Ensa 0.31316079339713976

ENSMUST00000037154 1209 Kcnj15 0.28420400944085805

ENSMUST00000040005 861 Alkbh3 0.254989196894593

ENSMUST00000044825 1032 Ndufaf5 0.18268382519615176

ENSMUST00000034986 1107 Ube2cbp 0.21602232268128913

ENSMUST00000049930 540 Tcf21 0.3712744426588666

ENSMUST00000041398 993 H2-M10.6 0.2643728127365825

ENSMUST00000038131 1071 Rfc3 0.2168808483107346

ENSMUST00000049624 945 Olfr1445 0.22654181501241075

ENSMUST00000037912 4272 Ssh2 0.20840367241994445

ENSMUST00000047008 447 Cst10 0.2706035416553788

ENSMUST00000031588 1554 Usp30 0.3116963765175179

ENSMUST00000034510 1548 Pvrl1 0.35178355882051654

ENSMUST00000034022 663 Sap30 0.21448924138254472

ENSMUST00000049519 1230 Irgm1 0.22996585824991336

ENSMUST00000049197 1131 Tm6sf2 0.2989261023907479

ENSMUST00000048194 1329 Tfap2e 0.3004916168420314

ENSMUST00000035301 357 Atp6v1g1 0.3817632400420992

ENSMUST00000035495 4320 Fanca 0.25333509658281317

ENSMUST00000046122 1560 Lap3 0.22431682498115907

ENSMUST00000040873 2628 Srrt 0.3060233900905855

ENSMUST00000044767 1014 Neurod6 0.2103732604038398

ENSMUST00000032908 1506 Cyp2r1 0.14261523944173946

ENSMUST00000043050 1260 Chst12 0.39650336830569866

ENSMUST00000037820 756 Hdhd3 0.2742513349273605

ENSMUST00000040261 972 Macrod1 0.3526694562974064

ENSMUST00000034472 933 Jam3 0.22970894823224022

ENSMUST00000037297 513 Ssxb1 0.21258301827916867

ENSMUST00000034287 1842 Klhl36 0.42658812476604185

ENSMUST00000048994 1146 Nfkbil1 0.33248479943599024

ENSMUST00000036267 1593 Chst2 0.29982800722790165

ENSMUST00000031574 711 Spdye4b 0.340460523854056

ENSMUST00000038287 1155 Dusp5 0.3367939755762047

ENSMUST00000038546 3420 Tex16 0.16108429557291473

ENSMUST00000031364 2064 Sdad1 0.2686729937121515

ENSMUST00000039153 1251 Cept1 0.14866299996949744

ENSMUST00000049621 504 Hes5 0.4081779063387152

ENSMUST00000031399 678 Psph 0.2612314327503321

ENSMUST00000033464 1200 Brs3 0.2219553919003583

ENSMUST00000032065 1518 Pcyox1 0.2609117707160319

ENSMUST00000041627 6531 Sdk2 0.34693119823137686

ENSMUST00000034231 279 Ccl22 0.24881924649983544

ENSMUST00000033992 1503 Gsr 0.27601631258674053

ENSMUST00000031624 945 1700018F24Rik 0.22896398990663694

ENSMUST00000037380 3495 Atp8a1 0.2173231558466395

ENSMUST00000030524 414 Pla2g5 0.3435831862144697

ENSMUST00000042509 3390 Ppip5k2 0.16874207059949056

ENSMUST00000048657 3291 Sec24c 0.23977712095704148

ENSMUST00000034699 1416 Scg3 0.23148013938007506

ENSMUST00000038719 1749 Nudcd1 0.17857901555427833

ENSMUST00000035918 1515 Cyp3a11 0.20309763264974948

ENSMUST00000030886 1164 Miip 0.28356709452265266

ENSMUST00000044749 4866 Myo3a 0.1861958433848472

ENSMUST00000034150 2088 Gab1 0.2488159549940311

ENSMUST00000050125 927 2900026A02Rik 0.3684220107298176

ENSMUST00000049680 2001 Zfp710 0.40321511423460044

ENSMUST00000032492 681 Cd9 0.2954654273970759

ENSMUST00000030165 1872 Fancg 0.23316206804241024

ENSMUST00000047425 1563 Sntb2 0.25287052203528004

ENSMUST00000041110 2577 Ttc7 0.34974518883303124

ENSMUST00000047936 1116 Cmklr1 0.40052648728740864

ENSMUST00000034280 927 Zdhhc7 0.3294509005575273

ENSMUST00000047057 612 Dpcd 0.26334901012320744

ENSMUST00000036153 630 Cops8 0.23140832950680112

ENSMUST00000030626 474 Tmem50a 0.2452750728915051

ENSMUST00000035929 864 Aspdh 0.28389847218505576

ENSMUST00000048402 1749 Spata7 0.19987929219678036

ENSMUST00000040429 2298 Usp45 0.18799510315083287

ENSMUST00000042546 1980 Ano10 0.2432785351048572

ENSMUST00000046941 465 Rnf122 0.3199544429164636

ENSMUST00000036072 2028 5031414D18Rik 0.2347000017750872

ENSMUST00000048026 942 Hoxa11 0.3016311721727422

ENSMUST00000039317 3393 Tmc3 0.26929772908271604

ENSMUST00000032705 2061 Rhpn2 0.29675784109791425

ENSMUST00000038374 2175 Pcca 0.20099711124607952

ENSMUST00000037419 1377 Pramel1 0.24164590751032983

ENSMUST00000039075 342 Spag11b 0.17032779532028358

ENSMUST00000043709 1125 Gna15 0.37663155694370865

ENSMUST00000049064 552 Rap2b 0.3935716505325752

ENSMUST00000031740 2001 Mepce 0.2671877087195375

ENSMUST00000044987 354 Hypm 0.2679885269630867

ENSMUST00000038942 2184 Pbxip1 0.3226479713705839

ENSMUST00000043149 969 Grap2 0.29473015898731075

ENSMUST00000034377 1239 Pla2g15 0.2868819621369361

ENSMUST00000043141 15465 Hydin 0.27018686038213674

ENSMUST00000047379 1542 Ptger4 0.3069892892040718

ENSMUST00000034817 993 Paqr5 0.32425883414159967

ENSMUST00000047207 1920 Ccdc27 0.3376384300628441

ENSMUST00000034427 1500 Adat1 0.25715702801991425

ENSMUST00000046177 984 Nfkbid 0.2779320951279318

ENSMUST00000033431 969 Slc25a14 0.16793011879897032

ENSMUST00000031280 795 Fgf5 0.2364249199427485

ENSMUST00000046704 1263 Ifi47 0.21120006971245206

ENSMUST00000031788 1008 Hibadh 0.19973241414749557

ENSMUST00000047463 2295 Arhgap44 0.30079937885890745

ENSMUST00000032818 861 Fbxo17 0.2972557925290618

ENSMUST00000033582 243 Cox7b 0.17355708425278918

ENSMUST00000031391 633 Bcl7a 0.2743427029857786

ENSMUST00000035519 2523 Otud7b 0.2587998473649863

ENSMUST00000040402 396 Ppp1r11 0.25989996546867733

ENSMUST00000033257 735 Mettl10 0.15854488567380579

ENSMUST00000048486 1311 Fgg 0.24402903599532938

ENSMUST00000035110 1512 Hhatl 0.3137381932804664

ENSMUST00000031971 459 Ssbp1 0.1534554837837933

ENSMUST00000033053 3510 Itgax 0.26431941334869685

ENSMUST00000037585 1818 Dennd6a 0.1690676532075396

ENSMUST00000039064 1611 Fam124a 0.3138654482555547

ENSMUST00000044451 2238 Naaladl1 0.2819244834323976

ENSMUST00000036211 1005 Gpr12 0.29240520258573877

ENSMUST00000033642 1101 Dcx 0.24395502974263747

ENSMUST00000033157 471 Ndufab1 0.1938021189395693

ENSMUST00000033342 1086 Eif3f 0.2171774277364405

ENSMUST00000032736 2316 Mtmr10 0.22228950793004715

ENSMUST00000031650 936 Cdx2 0.29797425233065744

ENSMUST00000044019 1467 Slc41a3 0.312263173609258

ENSMUST00000033749 873 Pir 0.17737903861079612

ENSMUST00000031235 1578 Gbp8 0.2764073395335665

ENSMUST00000046751 3750 Zmym6 0.17210568156187667

ENSMUST00000045602 531 Ndufb10 0.36472886807449983

ENSMUST00000036992 471 Lmo1 0.40785074331436844

ENSMUST00000038423 750 Rtp4 0.22705104802508935

ENSMUST00000033123 4767 Abcc8 0.3456540082255702

ENSMUST00000038671 2118 Kri1 0.3419840617102887

ENSMUST00000035076 1116 Lrrc2 0.23577197936849117

ENSMUST00000048214 609 Oosp1 0.20424960707239162

ENSMUST00000039309 639 Sectm1b 0.25755753889646144

ENSMUST00000034056 2718 Tlr3 0.16733971956682328

ENSMUST00000031597 1785 Plbd2 0.3730488892114789

ENSMUST00000043650 1362 Fam222a 0.3634127799377245

ENSMUST00000034225 3933 Cntnap4 0.22155962059303244

ENSMUST00000034097 1293 Got2 0.30592409908980833

ENSMUST00000033490 1890 Ccdc120 0.26106269162642826

ENSMUST00000035240 597 Armc7 0.3148084957483923

ENSMUST00000046485 489 Efcab11 0.19837178669827316

ENSMUST00000033935 324 Smim19 0.1872470580703516

ENSMUST00000040398 1959 Glb1l2 0.25721072823921587

ENSMUST00000032211 1101 Gdf3 0.2463645971245875

ENSMUST00000042167 1413 Cpq 0.18664725961810627

ENSMUST00000041362 1512 Mavs 0.23486150633435465

ENSMUST00000047265 1503 1700019B03Rik 0.2560498343267969

ENSMUST00000045737 1827 Galnt11 0.23518263845005197

ENSMUST00000036576 2043 Ccdc138 0.1830704636383883

ENSMUST00000043150 2052 Acsl5 0.21260676132094258

ENSMUST00000048801 663 2210407C18Rik 0.16755508848338463

ENSMUST00000038782 1305 Mael 0.1956809011851007

ENSMUST00000042594 1149 Mlc1 0.26255009792690653

ENSMUST00000030583 720 Ak2 0.2970875398540783

ENSMUST00000039846 489 2410137M14Rik 0.24968410790945889

ENSMUST00000030952 819 Tnfrsf4 0.28260261554081834

ENSMUST00000047922 1707 Ttc22 0.33909067647116525

ENSMUST00000047527 3603 Zfp804a 0.17467553117753581

ENSMUST00000030840 1014 Angptl7 0.36286825240974674

ENSMUST00000037813 1068 Gnaz 0.3901016260411895

ENSMUST00000033173 2133 Polr3e 0.2865951296330826

ENSMUST00000043944 2715 Ano5 0.2270794713046651

ENSMUST00000040523 3642 Adamts2 0.3356040063806071

ENSMUST00000038841 1065 Clvs1 0.23013049696988985

ENSMUST00000032151 624 Eif4e3 0.22585820520316985

ENSMUST00000034090 3972 Sall1 0.27217840566506746

ENSMUST00000033611 1350 Xkrx 0.23147911703773066

ENSMUST00000043637 1581 Mitf 0.25892794895478816

ENSMUST00000048375 1179 Fam102a 0.31319378185312

ENSMUST00000032196 561 Arl8b 0.19343277988280122

ENSMUST00000030281 1752 Eps15 0.18495277205378544

ENSMUST00000049545 1377 Adra2c 0.3053480460711722

ENSMUST00000031276 1422 Rasgef1b 0.26147504601201665

ENSMUST00000043612 1794 Pla2g4c 0.23347177379287595

ENSMUST00000035481 333 Chchd5 0.32397808026283603

ENSMUST00000046647 498 4930549C01Rik 0.22247885065806638

ENSMUST00000035484 846 Cdv3 0.2225628424063796

ENSMUST00000037473 1473 Ap5m1 0.14735587068966957

ENSMUST00000032179 5661 Nup210 0.28593305441903083

ENSMUST00000039173 3651 2310035C23Rik 0.16256030214927922

ENSMUST00000048079 2928 Fam160a2 0.2331548174807212

ENSMUST00000038107 810 Cited2 0.3793037999031272

ENSMUST00000039926 1992 Dusp8 0.3509030388860953

ENSMUST00000038489 1254 Pygo1 0.23199043796884755

ENSMUST00000042586 1377 Tespa1 0.23008879517477931

ENSMUST00000040835 420 Chchd4 0.31260459306697336

ENSMUST00000037863 510 Il25 0.34873632065976906

ENSMUST00000033131 486 Lamtor1 0.3065103584871184

ENSMUST00000036333 1068 Prkx 0.24363220650194958

ENSMUST00000033378 1656 Ebf3 0.29272101860606653

ENSMUST00000031723 579 Epo 0.23251029065395376

ENSMUST00000037541 1014 Dgat2l6 0.20165136884093945

ENSMUST00000034074 2682 N4bp1 0.20181650669047962

ENSMUST00000041558 9108 Ep400 0.22990824584242028

ENSMUST00000043718 1359 2310007B03Rik 0.32147238764598035

ENSMUST00000033761 6138 Hcfc1 0.2606342783722021

ENSMUST00000043961 4929 Abca5 0.16513803810846472

ENSMUST00000046071 1053 Klhdc8a 0.31811014078282634

ENSMUST00000039487 216 Gtf2h5 0.30366582747746007

ENSMUST00000040555 2412 Rnf10 0.28192212997037186

ENSMUST00000048050 837 Tmem45b 0.2911303140609071

ENSMUST00000030039 4737 Fmn2 0.21873762808361286

ENSMUST00000049126 2214 Dner 0.3019064733977754

ENSMUST00000042729 1737 Npnt 0.24109666780465921

ENSMUST00000046963 1194 Map2k4 0.1836902734743789

ENSMUST00000042750 1212 Tmem246 0.3082826094809722

ENSMUST00000034499 714 Dcun1d5 0.17043146604969847

ENSMUST00000033575 1572 Magee2 0.233372825478748

ENSMUST00000035227 642 Nicn1 0.2946868746558081

ENSMUST00000030518 906 Capzb 0.2907101617700514

ENSMUST00000040863 231 Ppp1r1c 0.204061621201121

ENSMUST00000032447 909 Ergic2 0.16289490315088126

ENSMUST00000033326 1941 Wee1 0.19255314835561133

ENSMUST00000040084 663 Lhfpl1 0.23177307347815557

ENSMUST00000044729 3105 Ccdc129 0.21365438654828986

ENSMUST00000045907 4299 2700049A03Rik 0.16563844896119598

ENSMUST00000032719 7101 Nav3 0.21857012163140865

ENSMUST00000042534 1383 Ccdc85a 0.2913374025523278

ENSMUST00000045215 864 Rfpl4 0.21685440556542807

ENSMUST00000034785 1857 Glce 0.2021550948865769

ENSMUST00000032967 696 Lipt2 0.25147376264222693

ENSMUST00000035844 567 Josd2 0.335952885345199

ENSMUST00000030636 450 Stmn1 0.32041383045468813

ENSMUST00000038760 1587 Lad1 0.2813703592373035

ENSMUST00000047883 885 Tspan15 0.35049013094868486

ENSMUST00000033979 855 Star 0.30512486130089744

ENSMUST00000031734 2043 Lrch4 0.2697367195140364

ENSMUST00000046045 1824 Nexn 0.14498291603557387

ENSMUST00000043551 3258 Ankib1 0.19990140309589868

ENSMUST00000030451 1536 Toe1 0.25532667699117195

ENSMUST00000049474 1797 Fbxw8 0.303903489811074

ENSMUST00000047614 2853 Pcdha12 0.2448951178194199

ENSMUST00000049251 1263 Cpa4 0.25899110887009646

ENSMUST00000033906 501 1700016D06Rik 0.17888954301150983

ENSMUST00000031304 645 Tesc 0.39789656978263677

ENSMUST00000038472 1905 Hdx 0.15619195547739867

ENSMUST00000029955 651 1700009N14Rik 0.251828412871498

ENSMUST00000042096 2985 Emc1 0.30103422625224324

ENSMUST00000043061 477 Fam159b 0.226703310547758

ENSMUST00000039695 6384 Frem3 0.23307597765681817

ENSMUST00000044306 3600 Grm1 0.2966993173417265

ENSMUST00000033650 675 Rs1 0.2531404991218979

ENSMUST00000038228 1008 Tmx4 0.24715489861772152

ENSMUST00000049348 1413 Traip 0.28510934666216253

ENSMUST00000048099 1314 Tmem194 0.21997083304389559

ENSMUST00000048685 747 Abhd14a 0.2828838603266826

ENSMUST00000044434 900 Crx 0.2945535625173077

ENSMUST00000047273 921 Rpusd1 0.33958919178358676

ENSMUST00000037526 5571 Tanc1 0.26604865393942384

ENSMUST00000039135 5607 Dock5 0.2653302026652748

ENSMUST00000045726 2130 Rgl3 0.2889722501750274

ENSMUST00000041544 3666 Fbln2 0.31674755716480774

ENSMUST00000049308 8013 Itpr3 0.4065994689572302

ENSMUST00000036092 3489 Kcnh2 0.3287995563452037

ENSMUST00000037219 2589 Agbl2 0.24241086305995194

ENSMUST00000048565 1401 Trim60 0.2075765547197665

ENSMUST00000049395 1416 Ttc29 0.26895881276580214

ENSMUST00000046022 3735 Skiv2l 0.30814992697228366

ENSMUST00000031628 2088 Ptcd1 0.32430129416262266

ENSMUST00000034308 1701 Bco1 0.28352521236677514

ENSMUST00000031388 1797 Vps33a 0.2838439351957053

ENSMUST00000040248 2802 Spidr 0.19458245372975258

ENSMUST00000045994 1329 Rbm34 0.18471246472767872

ENSMUST00000047652 813 Tspan14 0.34181229606401464

ENSMUST00000035033 2718 Copb2 0.206931822124086

ENSMUST00000043285 876 Gm11992 0.26398266946544824

ENSMUST00000036248 825 Pmepa1 0.36206974155109845

ENSMUST00000034973 1278 Smad3 0.33392662315963284

ENSMUST00000031513 669 Srsf9 0.280620689039684

ENSMUST00000033827 1695 Grk1 0.33088567713883493

ENSMUST00000045078 987 Grhpr 0.2930396923571943

ENSMUST00000041827 1671 Slc22a20 0.29632508828666043

ENSMUST00000035181 2019 Aste1 0.19271319682145674

ENSMUST00000041195 1965 Ccdc81 0.24998442589013622

ENSMUST00000045970 1674 Gpc1 0.3713049995159783

ENSMUST00000030931 2322 Pank4 0.31737289740570485

ENSMUST00000032359 825 Aebp2 0.1907189615055838

ENSMUST00000043929 1002 Ccdc68 0.19677860221435914

ENSMUST00000038154 852 Mgarp 0.2260855666024881

ENSMUST00000031670 222 Gng11 0.21485390043504352

ENSMUST00000034507 1479 Sesn3 0.24833905592541522

ENSMUST00000047036 1200 Cd3eap 0.27879175952619

ENSMUST00000037059 804 Ctrc 0.34261227743963807

ENSMUST00000034346 1677 Ces2a 0.2858663632903893

ENSMUST00000034138 1239 Dnaja2 0.18736997005302108

ENSMUST00000040853 1584 Oxsr1 0.19478054854997107

ENSMUST00000048688 3585 Fbxo38 0.2121120554571486

ENSMUST00000047196 717 Wbscr27 0.3017748416596786

ENSMUST00000044207 2421 Sart1 0.3309942891500733

ENSMUST00000049005 1599 Bmpr1a 0.18217570419555068

ENSMUST00000030917 2184 Ski 0.3424606169903136

ENSMUST00000034184 1455 Irx5 0.2834325484832163

ENSMUST00000049246 930 Setmar 0.1924827494676609

ENSMUST00000041987 5721 Dicer1 0.20744718661497275

ENSMUST00000035751 2481 L3mbtl1 0.29625706814035524

ENSMUST00000043195 2310 Gcfc2 0.1804664802690122

ENSMUST00000047013 522 Cmtm8 0.3004998722530664

ENSMUST00000045270 1785 Cbfa2t2 0.24179429337892733

ENSMUST00000029975 363 Cga 0.2695024264129439

ENSMUST00000044299 1176 Sstr1 0.3400227814829433

ENSMUST00000033820 1341 F7 0.3057938921125704

ENSMUST00000046856 1407 Chn2 0.27827458526728066

ENSMUST00000039726 1383 3110052M02Rik 0.17981425153503314

ENSMUST00000034131 2391 Vps35 0.15690181853276097

ENSMUST00000048580 822 Tsga13 0.2133264227326167

ENSMUST00000044851 2799 Pcdhga12 0.2485386429463353

ENSMUST00000038166 4008 Dhx57 0.22823986052817757

ENSMUST00000037025 2481 Kdm1b 0.24983549577455044

ENSMUST00000034172 1698 Ces1d 0.2336167325982657

ENSMUST00000030510 2532 Tas1r2 0.3904314329309345

ENSMUST00000046290 1635 Lpcat2 0.20481437451958212

ENSMUST00000030785 2616 Espn 0.3283226971567577

ENSMUST00000034265 642 1700018B08Rik 0.3012839490538622

ENSMUST00000043922 1848 Zfp653 0.3502493999817496

ENSMUST00000046114 408 Mrpl54 0.33635357269219407

ENSMUST00000040134 396 Ndp 0.2829629016407937

ENSMUST00000046611 1824 Cuzd1 0.2221389207443099

ENSMUST00000035725 228 Brk1 0.24615316169669865

ENSMUST00000033189 1362 Cckbr 0.27940244948802895

ENSMUST00000049527 1146 Prkar1a 0.2431411818059896

ENSMUST00000038010 936 Glt6d1 0.2547288465964148

ENSMUST00000042953 7071 Nbas 0.21947075800787239

ENSMUST00000035626 1185 4930480E11Rik 0.16647351548212555

ENSMUST00000047761 690 Cldn10 0.21430276998411119

ENSMUST00000041551 951 Aagab 0.23671327168522205

ENSMUST00000029925 522 Ndufaf4 0.17222608293025451

ENSMUST00000035890 1437 Slc7a13 0.16742139119837107

ENSMUST00000033899 5124 Col4a2 0.22888681582652962

ENSMUST00000032409 1125 Camk1 0.31949071618798014

ENSMUST00000034632 348 Tmem218 0.2365034528724621

ENSMUST00000033524 660 Ctag2 0.18794627573116685

ENSMUST00000040202 3150 Atxn2l 0.2416570699903654

ENSMUST00000034869 1080 Isl2 0.35094326217746985

ENSMUST00000039118 1869 Zic5 0.30626051574143354

ENSMUST00000034388 1314 Vps4a 0.23925920058118194

ENSMUST00000040097 1656 Palmd 0.21494474201577782

ENSMUST00000036719 4953 Prex1 0.3838366301862888

ENSMUST00000049488 1386 Serinc5 0.2941719105382184

ENSMUST00000030739 2577 Epb4.1 0.21808633933130311

ENSMUST00000045441 2532 Pygb 0.3502497035885079

ENSMUST00000033008 792 Psma1 0.177655557410574

ENSMUST00000033207 2142 Zp2 0.21904428352066496

ENSMUST00000031061 2388 Dhx15 0.1698372682670032

ENSMUST00000050140 480 Tmem88 0.2552278308098359

ENSMUST00000036174 1374 Gramd1c 0.20169570199625592

ENSMUST00000046792 2241 Olfml2b 0.28815747598486624

ENSMUST00000049057 2469 Fam171a2 0.30728516497662633

ENSMUST00000048478 996 Olfr750 0.2420550805702308

ENSMUST00000031707 2781 Aass 0.19243480014058867

ENSMUST00000041905 1764 Nupl1 0.15981140748745826

ENSMUST00000034121 3042 Man2b1 0.278437146494077

ENSMUST00000046525 1386 Kremen2 0.3006925704436414

ENSMUST00000041531 993 H2-M10.5 0.2529501575968511

ENSMUST00000031501 888 1700123K08Rik 0.24062433064994504

ENSMUST00000030361 972 Tmem59 0.20243503930090626

ENSMUST00000045246 2637 Pkn3 0.29170407597241094

ENSMUST00000033236 1053 Thumpd1 0.2361473561779909

ENSMUST00000043368 4935 Zcchc11 0.15306925571731536

ENSMUST00000030501 921 Ebna1bp2 0.26247169912323676

ENSMUST00000032480 747 Ing4 0.29349043738092306

ENSMUST00000035695 3705 Rbp3 0.29132440114046615

ENSMUST00000034880 2013 Stra6 0.29158045002130917

ENSMUST00000030169 1062 Stoml2 0.25090314136514247

ENSMUST00000048471 3540 Abi3bp 0.18082659357592015

ENSMUST00000030279 1377 Atg4c 0.17355678606542713

ENSMUST00000031349 735 Snrnp35 0.30034797158634985

ENSMUST00000038223 1896 Zswim2 0.160840202683364

ENSMUST00000036437 1011 Mrpl1 0.13612845652410033

ENSMUST00000047935 1221 Tspyl4 0.27260749332165696

ENSMUST00000039061 3402 Trappc11 0.21772337397298938

ENSMUST00000032927 876 Stard10 0.3430472584946034

ENSMUST00000040025 7629 Tln2 0.2804478562942593

ENSMUST00000045870 999 Rnf225 0.28025422810362804

ENSMUST00000032396 1620 Lrmp 0.27168302262767996

ENSMUST00000042988 1479 Atg14 0.2659584140664148

ENSMUST00000033665 3996 Map3k15 0.21297257685920698

ENSMUST00000032388 1260 Rassf8 0.3013910891172047

ENSMUST00000031841 849 Tra2a 0.1914545656026386

ENSMUST00000030980 2142 Trmt44 0.2670150606799308

ENSMUST00000034860 1542 Cyp1a2 0.34818987221430997

ENSMUST00000041065 3021 Myo1d 0.25809838051331957

ENSMUST00000035985 2124 Fbxl18 0.393577628514536

ENSMUST00000039577 2346 Zfp51 0.1793612313688197

ENSMUST00000031489 840 Ankrd7 0.1524734018047894

ENSMUST00000035419 1665 Prf1 0.3188796836414925

ENSMUST00000046863 1110 Hsd3b7 0.31570682917744103

ENSMUST00000030760 801 Necap2 0.3033983944216576

ENSMUST00000045887 327 Cisd1 0.3428840714605206

ENSMUST00000032080 567 Pradc1 0.28679168288073265

ENSMUST00000035395 426 Anapc15 0.3137454660320884

ENSMUST00000031322 504 Cxcl15 0.19049117867372667

ENSMUST00000030306 2187 Hook1 0.17531174744515168

ENSMUST00000042334 654 Rpl10a 0.370730875158148

ENSMUST00000032749 1854 Vps33b 0.27584264928640634

ENSMUST00000033414 1917 Slc6a14 0.15238219883722406

ENSMUST00000032732 2253 Apba2 0.35465855414019937

ENSMUST00000044051 252 Timm8b 0.23643032679385237

ENSMUST00000034463 801 Arv1 0.26656025499374203

ENSMUST00000040603 4599 Agl 0.19440750349291167

ENSMUST00000046565 1695 Arx 0.3268957186907427

ENSMUST00000040695 795 Sbspon 0.2809025497967912

ENSMUST00000033498 519 Timm17b 0.26049690239198975

ENSMUST00000041569 1134 Ccdc113 0.24890488990282544

ENSMUST00000041640 1323 Ankmy2 0.2484826440827933

ENSMUST00000043624 6516 Med13 0.1535595656892795

ENSMUST00000037996 324 Prm2 0.3116381753464351

ENSMUST00000032500 1185 Prmt8 0.31453328188944085

ENSMUST00000030562 1251 Hcrtr1 0.35283264979506673

ENSMUST00000040783 3441 Usp40 0.18610293697060729

ENSMUST00000030471 2424 Lrrc41 0.22429584724085894

ENSMUST00000040824 279 Dpm3 0.3059157095258365

ENSMUST00000035218 2145 Nckipsd 0.2948324742575736

ENSMUST00000037553 354 Galp 0.243808575541395

ENSMUST00000040967 858 Vps37b 0.28089710270555773

ENSMUST00000042842 3753 Cdon 0.2245456810707978

ENSMUST00000041778 1224 Zfp174 0.25441988340029575

ENSMUST00000041993 282 Iapp 0.22735754761217275

ENSMUST00000030942 450 Mrpl20 0.2591673451880831

ENSMUST00000030198 2916 Reck 0.2511571837998784

ENSMUST00000033626 1281 Serpina7 0.17909989391799477

ENSMUST00000046004 1593 1700019O17Rik 0.25905844730084715

ENSMUST00000036208 2490 Slc9a3 0.303126610536458

ENSMUST00000031729 2397 Tfr2 0.30574450792529656

ENSMUST00000037488 5937 Dock4 0.23947192854956711

ENSMUST00000042775 858 Neurl2 0.28314109438539964

ENSMUST00000033050 957 Lyve1 0.25017892756731874

ENSMUST00000047173 1038 Msantd4 0.2688354494328906

ENSMUST00000039541 2004 Tubgcp4 0.2291860800273069

ENSMUST00000036031 2577 Gsap 0.20955158533883297

ENSMUST00000049453 3252 Ube3c 0.19324674822987567

ENSMUST00000034270 378 Map1lc3b 0.36850005416114795

ENSMUST00000035735 264 Ndufa4l2 0.31734664082249264

ENSMUST00000045372 780 Bpgm 0.2585595316887507

ENSMUST00000035009 504 Cmtm7 0.2878079636184492

ENSMUST00000030311 1803 Ift74 0.15390178997968706

ENSMUST00000042792 5046 Scn7a 0.17637941534210616

ENSMUST00000030862 1032 Draxin 0.3297700068781468

ENSMUST00000049877 567 Msgn1 0.32552429111857495

ENSMUST00000044672 1506 Cdk19 0.22716726447367025

ENSMUST00000037678 1737 Tkfc 0.24988199926023508

ENSMUST00000032237 3855 Bms1 0.22452977807016683

ENSMUST00000048923 1227 Spred3 0.28772915501806023

ENSMUST00000045583 969 Crhbp 0.27275546348718016

ENSMUST00000030078 1473 Hsdl2 0.20164143338517676

ENSMUST00000030420 3015 Epha8 0.35134899551182636

ENSMUST00000049911 477 Ube2i 0.23129211850882425

ENSMUST00000035157 810 Srprb 0.2504441177278392

ENSMUST00000040454 2142 Bbs10 0.16505486947861256

ENSMUST00000034607 1536 Arcn1 0.17881083886866367

ENSMUST00000030180 543 Sit1 0.2631366772751937

ENSMUST00000033163 957 Mettl9 0.20610451024171197

ENSMUST00000041008 1314 Fntb 0.2983001356414819

ENSMUST00000037248 1806 Hirip3 0.28162729447975754

ENSMUST00000031898 456 Sval1 0.14041058691303013

ENSMUST00000034832 1782 Ptpn9 0.24707551839483408

ENSMUST00000032128 555 Gkn2 0.3157899911813239

ENSMUST00000050103 645 Neurog3 0.289623543629791

ENSMUST00000044532 6093 Dock3 0.24521773219254223

ENSMUST00000032877 2700 Ddias 0.1475934958275843

ENSMUST00000034049 897 Slc25a4 0.31423104746994457

ENSMUST00000032629 3762 Cyfip1 0.27213921675129954

ENSMUST00000049559 468 Ear14 0.1805148859674238

ENSMUST00000038598 684 Idi2 0.29494339692324684

ENSMUST00000044792 594 Rcan2 0.27445153752216817

ENSMUST00000048982 2499 Prickle1 0.2924288983642857

ENSMUST00000039803 1038 Ubac2 0.2551502994202386

ENSMUST00000038572 1761 Hnrnpl 0.2946127130084129

ENSMUST00000046234 297 Lce3b 0.3782540093260289

ENSMUST00000044681 567 Arl6ip5 0.31443399731112237

ENSMUST00000045604 408 Mrpl41 0.24836852243370153

ENSMUST00000039121 1002 Fam170a 0.2680502671258111

ENSMUST00000047568 918 Havcr1 0.196994682192047

ENSMUST00000040711 5082 Nrap 0.2996120915963782

ENSMUST00000038522 2466 Slmap 0.16608080708620176

ENSMUST00000042322 3216 Iqch 0.20417089911376102

ENSMUST00000030251 1455 Pde4b 0.25455441406006724

ENSMUST00000035780 1566 Oma1 0.21269935765603828

ENSMUST00000035861 1920 Pex5 0.2869008722678438

ENSMUST00000043359 3156 Smarca5 0.15607258030771512

ENSMUST00000041730 1119 Lmx1b 0.3845408421308042

ENSMUST00000039659 834 Cbr1 0.2872820758074653

ENSMUST00000048993 672 Polr3g 0.22235007067942844

ENSMUST00000043259 3426 Pde3a 0.26834920433247084

ENSMUST00000030800 1638 Fastk 0.2780299531025881

ENSMUST00000032768 1245 Nr2f2 0.33057867065182805

ENSMUST00000037685 417 Spt1 0.1589013520644102

ENSMUST00000046399 1248 Apmap 0.22191992880341083

ENSMUST00000038210 3270 Sec24a 0.16993678533732828

ENSMUST00000031694 1653 Lmod2 0.2353888456284296

ENSMUST00000039323 1821 AA986860 0.28297989671799556

ENSMUST00000041463 726 Pacrg 0.272925419634036

ENSMUST00000030508 1512 Pax7 0.33202937600998306

ENSMUST00000048260 1554 Lman1 0.22739516070286933

ENSMUST00000050026 981 BC051628 0.36716671667989575

ENSMUST00000049027 2373 Slc26a9 0.30939773485066313

ENSMUST00000039775 1614 Lgi4 0.2999119532076598

ENSMUST00000030140 4002 Ikbkap 0.21941873167970366

ENSMUST00000044668 1494 Acss3 0.1926574346647311

ENSMUST00000041830 672 Ntmt1 0.4098689643421459

ENSMUST00000039011 702 Uchl4 0.15965282222764512

ENSMUST00000043896 11172 Zfhx3 0.32036865989864827

ENSMUST00000041229 1005 Bdkrb1 0.3274036743689286

ENSMUST00000037491 2199 F13a1 0.2451000576561995

ENSMUST00000042025 1689 Antxr1 0.2737004071474187

ENSMUST00000031651 2514 Pan3 0.19638109407707338

ENSMUST00000042553 1026 A4gnt 0.294897764258131

ENSMUST00000038574 4098 Dhx29 0.2050502853954667

ENSMUST00000046892 405 Cplx1 0.3929855884408482

ENSMUST00000039674 1425 Pknox2 0.31316186886050507

ENSMUST00000033211 1494 Trim30d 0.20673354838366237

ENSMUST00000044224 1086 Oas1d 0.2951855505789331

ENSMUST00000041024 1443 Lace1 0.21770328204644956

ENSMUST00000049339 1065 Nodal 0.31154274644727925

ENSMUST00000048794 813 Hoxa5 0.3022110534237569

ENSMUST00000037827 2256 Slc45a1 0.3282843128538701

ENSMUST00000034030 1869 Tmem184c 0.15626638479477126

ENSMUST00000038197 384 Mrap 0.32556682686181715

ENSMUST00000030376 2091 Kcnq4 0.3165632025015418

ENSMUST00000049985 1392 Hepacam2 0.19646384561392322

ENSMUST00000033278 1707 Mmp21 0.26794070370623796

ENSMUST00000037551 1575 Ppp1r16a 0.31567366853524603

ENSMUST00000040069 2229 Colec12 0.22893977484768482

ENSMUST00000043760 1188 Mvk 0.2951589774148373

ENSMUST00000033952 945 Sfrp1 0.42103955248232994

ENSMUST00000036584 1647 Pank1 0.2738544283773349

ENSMUST00000031690 1485 Hyal6 0.1640675192537568

ENSMUST00000031793 996 Nt5c3 0.17983424353046942

ENSMUST00000034244 897 Tmem38a 0.3435713699920082

ENSMUST00000044878 1680 Akp3 0.30513255419431984

ENSMUST00000031935 756 1700074P13Rik 0.15381157744252363

ENSMUST00000037649 2946 Rab3gap1 0.19959457447195705

ENSMUST00000034707 4845 Smarca4 0.3312106660846986

ENSMUST00000033783 603 Tceal6 0.28821103821061805

ENSMUST00000049977 1167 Dpf1 0.35867931315080026

ENSMUST00000048417 12921 Dync2h1 0.1668870298877036

ENSMUST00000037853 1512 Kdelc2 0.23687744358906673

ENSMUST00000033009 2277 Prkrir 0.18885103425580782

ENSMUST00000030845 858 Nmnat1 0.3294604016404559

ENSMUST00000044564 1872 Foxj3 0.19051780532356885

ENSMUST00000048180 1071 Plekha1 0.2241947779615053

ENSMUST00000042646 969 Atoh8 0.2740525441419759

ENSMUST00000040468 1614 Primpol 0.17307828492892777

ENSMUST00000030464 1386 Pik3r3 0.2239401763140791

ENSMUST00000033501 3450 Hdac6 0.24378428658821988

ENSMUST00000038514 1533 Nup54 0.17999070362253192

ENSMUST00000040750 477 Lif 0.31386185395022315

ENSMUST00000041317 1035 Ammecr1 0.2529950510811356

ENSMUST00000040718 1017 Ostm1 0.29782960507480843

ENSMUST00000048102 6003 Myh14 0.3584239416216097

ENSMUST00000038523 1683 Ric8b 0.18606231085211836

ENSMUST00000035083 1212 Stac 0.27510943132473475

ENSMUST00000032398 1518 Thumpd3 0.1808706903421732

ENSMUST00000031486 813 Prkab1 0.3122322925833035

ENSMUST00000033699 7944 Flna 0.25653133676452655

ENSMUST00000035163 2103 1300017J02Rik 0.26070725658556965

ENSMUST00000049507 2085 Pcsk9 0.3263513637284514

ENSMUST00000044604 3321 Crybg3 0.20650066198829614

ENSMUST00000031632 1566 Zkscan14 0.2664333452100784

ENSMUST00000033522 912 3830417A13Rik 0.22662350419222688

ENSMUST00000038529 1845 Rbbp8nl 0.29786485202831714

ENSMUST00000039324 5202 Abca17 0.22486109883071959

ENSMUST00000045537 1440 Chrm4 0.3558036099215018

ENSMUST00000045693 1251 Smyd5 0.29100255773400907

ENSMUST00000049509 1254 Vat1l 0.3059289625207694

ENSMUST00000034267 1941 Slc27a1 0.32968877599347607

ENSMUST00000045866 2556 Ddx21 0.22074416811382924

ENSMUST00000038211 2733 Slfn9 0.2148087902775313

ENSMUST00000032944 993 Gdpd3 0.2931271963667

ENSMUST00000042158 2676 Esyt3 0.33488946521942736

ENSMUST00000034004 1128 Pdgfrl 0.27449879821238293

ENSMUST00000035283 2262 Nup88 0.19942029322790134

ENSMUST00000034920 1347 Map2k5 0.20213602352441595

ENSMUST00000047720 4044 Ptchd2 0.33887038926894186

ENSMUST00000036555 5229 Myo5c 0.29648739629841975

ENSMUST00000030117 1542 Smu1 0.21072767929236633

ENSMUST00000040572 1308 Zc3h10 0.31039351942382404

ENSMUST00000049242 555 Lrrc20 0.3441491179939504

ENSMUST00000040227 504 Cldnd2 0.22484887278484766

ENSMUST00000041684 3606 Caskin2 0.29279638624653953

ENSMUST00000041537 300 Gm7257 0.21596456738835274

ENSMUST00000043599 2592 Rps6ka5 0.2113859456202005

ENSMUST00000048557 1254 Npffr2 0.2358162697945132

ENSMUST00000048698 2037 Taf6 0.2755853793769489

ENSMUST00000032402 1362 Bcat1 0.2550576284919529

ENSMUST00000032192 8250 Itpr1 0.28282646135522516

ENSMUST00000040821 981 Heyl 0.2766666470879843

ENSMUST00000032134 471 Rab43 0.360881589119553

ENSMUST00000032259 600 Cd69 0.20644356418170579

ENSMUST00000047264 231 Sprr2i 0.3790211792659894

ENSMUST00000041459 669 Cyb561d2 0.28997412765889

ENSMUST00000036362 612 Lat2 0.3215395392875866

ENSMUST00000034621 2928 Nlrx1 0.30100198547950974

ENSMUST00000038859 3138 Pik3cd 0.3909514652152788

ENSMUST00000030309 1014 Eqtn 0.17812194814204677

ENSMUST00000030813 429 Apitd1 0.2893652435264027

ENSMUST00000036453 2823 Map3k10 0.33222973141932394

ENSMUST00000037374 1788 Gab3 0.18614452185577113

ENSMUST00000039763 984 Ginm1 0.17073123356538458

ENSMUST00000034086 1416 Nkd1 0.3355357799923907

ENSMUST00000034041 1050 Irf2 0.25452641563579687

ENSMUST00000040154 339 Cox6a1 0.31912207563369643

ENSMUST00000049630 279 Cox19 0.25274107588505823

ENSMUST00000036618 7716 Stab1 0.28726530774392

ENSMUST00000040983 951 Olfr6 0.21187662551616426

ENSMUST00000047409 1068 Vash2 0.3502816283972553

ENSMUST00000046518 1260 Itpk1 0.3448431456210346

ENSMUST00000039516 720 Egln3 0.3288350507939051

ENSMUST00000032093 2706 Prickle2 0.31439287165723934

ENSMUST00000035234 759 1700102P08Rik 0.20337069808861408

ENSMUST00000041924 8256 Muc5ac 0.3066913973062745

ENSMUST00000033267 1767 Pdilt 0.22874086615140615

ENSMUST00000036210 1179 Poglut1 0.2212100746219337

ENSMUST00000047620 1827 Fam151a 0.295110817474751

ENSMUST00000030033 1089 Murc 0.2535968110541826

ENSMUST00000031840 1725 Gpnmb 0.2796238392895789

ENSMUST00000039500 2274 Tmc1 0.2527268369161622

ENSMUST00000038750 894 Tas2r108 0.1826149830385309

ENSMUST00000046418 1347 E130309D02Rik 0.32767476475753005

ENSMUST00000036016 1665 Amh 0.2917355316472684

ENSMUST00000047677 4368 Ccdc18 0.12692134611945138

ENSMUST00000031359 2274 Ppef2 0.27033722768174084

ENSMUST00000043864 1011 Cdc123 0.21429176551413168

ENSMUST00000048962 3696 Kif4 0.20727806602549717

ENSMUST00000035113 234 Deb1 0.2835306955422966

ENSMUST00000031255 1317 Agpat9 0.2531332046389724

ENSMUST00000046548 2856 Lgr4 0.1905622451507553

ENSMUST00000031695 1506 Wasl 0.17914752056556826

ENSMUST00000036748 1299 Slc38a8 0.35233334374623193

ENSMUST00000048608 3387 Rtel1 0.26496431557154854

ENSMUST00000035635 3417 Bmp2k 0.2199850185724804

ENSMUST00000046750 852 Tspan33 0.34612744138001467

ENSMUST00000037313 1386 Gdf7 0.31776039552281565

ENSMUST00000029995 2052 Toporsl 0.19681129252059487

ENSMUST00000039338 1389 Kng2 0.21126413985361275

ENSMUST00000030189 2757 Gba2 0.26913375641717385

ENSMUST00000050236 1653 Txndc2 0.26315079559684046

ENSMUST00000049859 924 Olfr202 0.1790584866214059

ENSMUST00000042700 633 Gstp2 0.3377749211875506

ENSMUST00000048715 690 Hoxa7 0.3148024354466965

ENSMUST00000044844 1431 Mfsd12 0.35461285833077255

ENSMUST00000041361 4014 Zfp335 0.31341880593967

ENSMUST00000046929 4035 Usp31 0.23755251671583147

ENSMUST00000049369 1137 Obfc1 0.30016289619327774

ENSMUST00000044734 810 Rims4 0.3671605417479384

ENSMUST00000050258 1005 Ttc24 0.27371387519742446

ENSMUST00000034281 1224 6430548M08Rik 0.36175571178794685

ENSMUST00000033577 597 Pbdc1 0.18179736146732664

ENSMUST00000041623 1770 Enc1 0.28848377730916214

ENSMUST00000034889 3606 Hcn4 0.3204551147823324

ENSMUST00000042740 15105 Abca13 0.18407327648454416

ENSMUST00000038569 942 Spry1 0.3134624164084475

ENSMUST00000039380 786 Gjb6 0.33078334092525224

ENSMUST00000040628 1884 Zfp182 0.18777164089169895

ENSMUST00000036509 1230 Ubac1 0.2746044078042488

ENSMUST00000033004 2433 Il4ra 0.30923907449098414

ENSMUST00000036932 1107 Dcdc2a 0.2264762602918928

ENSMUST00000043429 1008 Nfyc 0.28921744476322353

ENSMUST00000040668 1455 Osbpl2 0.27248561938096605

ENSMUST00000043313 924 Nmnat2 0.34742610333122337

ENSMUST00000033049 294 Cox6a2 0.33382831678663916

ENSMUST00000032907 411 Calca 0.31698258885496955

ENSMUST00000034905 1914 Gclc 0.2628032488022067

ENSMUST00000032557 2196 Mark2 0.30120917538400255

ENSMUST00000036665 1974 Cog6 0.20099677118056983

ENSMUST00000034912 3789 Rasgrf1 0.3213130254890386

ENSMUST00000046384 762 C1qb 0.29003480490788847

ENSMUST00000035148 1932 Slco2a1 0.30598168148443894

ENSMUST00000039928 4071 Plekhh1 0.2913749904203267

ENSMUST00000041964 1006 H2-M11 0.2335506164057932

ENSMUST00000046028 2535 Strip2 0.2566581761229136

ENSMUST00000032891 10977 Smg1 0.1748014157827304

ENSMUST00000034803 2340 Zw10 0.21960662432715577

ENSMUST00000030563 828 Pef1 0.27525744753678955

ENSMUST00000036003 1482 Klhl42 0.3861236025926355

ENSMUST00000034234 942 Coq9 0.34878231311802227

ENSMUST00000037539 1323 Eva1c 0.2608980342630543

ENSMUST00000042286 3285 Fmnl1 0.30154551006446007

ENSMUST00000035497 1140 Oscp1 0.28339437317077787

ENSMUST00000047177 423 Nxt1 0.25712863098172806

ENSMUST00000033752 1923 Slc6a8 0.3301529709284573

ENSMUST00000042299 2967 Kcnh5 0.23088844634361616

ENSMUST00000032159 3087 Cntn3 0.18369549985853031

ENSMUST00000035799 1299 Fgl2 0.2777683301984162

ENSMUST00000043503 2982 Scube3 0.35107048748213676

ENSMUST00000045228 1767 Zkscan8 0.23484531965216376

ENSMUST00000036972 2328 3632451O06Rik 0.21110187036365266

ENSMUST00000041882 327 1600002K03Rik 0.30855516645605696

ENSMUST00000032347 600 Rerg 0.2690062675211203

ENSMUST00000032916 1434 Maz 0.31226727491918016

ENSMUST00000038407 1479 Larp6 0.36309968718261704

ENSMUST00000047632 1068 Surf6 0.36049328062843844

ENSMUST00000039721 1497 Slc17a3 0.18147529387375244

ENSMUST00000037974 4359 Ptprm 0.26577527519692984

ENSMUST00000032107 1800 Kbtbd8 0.17348000990756862

ENSMUST00000040914 639 Hist1h1c 0.35471838663372346

ENSMUST00000045469 2064 Pip5k1c 0.3637539599450852

ENSMUST00000041190 1389 Mcrs1 0.35469364757852595

ENSMUST00000033407 1530 Cttn 0.3111080967621492

ENSMUST00000033671 2223 Rps6ka3 0.1510674246284547

ENSMUST00000032461 1014 Tamm41 0.28027489286564794

ENSMUST00000034021 1974 Galnt7 0.21642691736150063

ENSMUST00000031533 954 Tfec 0.18813783127382583

ENSMUST00000039861 1005 Cd33 0.27729059984198046

ENSMUST00000037960 1095 Zdhhc9 0.28101889674195446

ENSMUST00000039855 9717 Golgb1 0.22495431558737825

ENSMUST00000043850 1038 Igflr1 0.2646037230238108

ENSMUST00000030457 1347 Nasp 0.17255495932515336

ENSMUST00000040912 3366 Anln 0.17757426897653678

ENSMUST00000038073 1767 Catsper2 0.21362231582602317

ENSMUST00000042970 1287 Kcnj2 0.22802404436792711

ENSMUST00000034755 2049 Tcf12 0.1897182651403977

ENSMUST00000040314 1218 Rbm17 0.21703973882153854

ENSMUST00000041447 3378 Trpa1 0.18953951024067917

ENSMUST00000037453 957 Prss34 0.28511857170741334

ENSMUST00000030360 1266 Lrrc42 0.24801435602366917

ENSMUST00000032728 2373 Tarsl2 0.2366702152883158

ENSMUST00000040944 1485 Cyp2g1 0.2650785189710461

ENSMUST00000045840 1014 Gpr26 0.36190242098417336

ENSMUST00000031077 1539 Zcchc4 0.2231496933264204

ENSMUST00000034745 3324 Myo1e 0.27650119590082883

ENSMUST00000042148 561 Mrps28 0.2613330614085097

ENSMUST00000041685 1152 Neurod2 0.3619807703212854

ENSMUST00000042587 975 Pitx2 0.3147086679919394

ENSMUST00000047745 1125 Pglyrp4 0.2327237282320457

ENSMUST00000037181 2871 Mdga2 0.164121603827514

ENSMUST00000039725 2514 Exo1 0.23201702919653555

ENSMUST00000033133 546 Rgs10 0.32762487621526226

ENSMUST00000040941 603 Erv3 0.18242969601803571

ENSMUST00000036340 4353 Fancd2 0.21596737028564716

ENSMUST00000039438 1839 Ntrk3 0.28679941188145475

ENSMUST00000042172 635 Tmem247 0.3550664737038988

ENSMUST00000036215 1266 Foxj1 0.3602274644638055

ENSMUST00000048959 1473 Cyp2c54 0.2002068490274564

ENSMUST00000031291 1032 Asphd2 0.3715991036720294

ENSMUST00000038860 1332 Spag4 0.28526079652825936

ENSMUST00000038149 1293 Pbx2 0.3060423699194769

ENSMUST00000039331 1407 Igsf21 0.34906025664934676

ENSMUST00000044624 1209 Kcnk9 0.3100212936284514

ENSMUST00000043754 996 Ctsll3 0.2670790305546543

ENSMUST00000038257 735 Gstt2 0.2841236507265802

ENSMUST00000031004 1719 Crmp1 0.2912157755887211

ENSMUST00000031401 636 Rhof 0.3456623462812285

ENSMUST00000034612 1455 Ddx25 0.2169183107021159

ENSMUST00000041776 543 Rgs8 0.3166421509927785

ENSMUST00000049460 1809 Grn 0.3074000097495369

ENSMUST00000030541 1665 Hp1bp3 0.24524571710024345

ENSMUST00000031072 2424 Anapc4 0.17577812898616196

ENSMUST00000030041 1050 Ambp 0.295848734728021

ENSMUST00000050067 2586 Hectd3 0.30684568866535694

ENSMUST00000034728 6336 Dock6 0.3399526710121302

ENSMUST00000034949 1380 Csnk1g1 0.17804818311922152

ENSMUST00000033888 627 Cd209e 0.26736004233134947

ENSMUST00000046891 1089 Ptger2 0.25663192307311467

ENSMUST00000046575 1251 Ptov1 0.3704014987358815

ENSMUST00000048792 252 1110008P14Rik 0.3386356172420758

ENSMUST00000036161 6135 Pi4ka 0.2714120161580126

ENSMUST00000040042 609 C8g 0.26003620151592494

ENSMUST00000030702 1056 Ppp1r8 0.2703408072018927

ENSMUST00000030684 2187 Gnl2 0.21785308159527697

ENSMUST00000034737 1323 Khdc3 0.29943485094322425

ENSMUST00000030675 777 Mrps15 0.23714967480131324

ENSMUST00000037057 2391 Zfp40 0.16355501935072592

ENSMUST00000038227 1005 Ackr1 0.23925216892180287

ENSMUST00000030937 1176 Mmp23 0.26745875304018135

ENSMUST00000034190 2349 Vac14 0.3276837693559559

ENSMUST00000041646 1437 Drd5 0.32573322395830256

ENSMUST00000033875 825 Prosc 0.2613933944077157

ENSMUST00000045066 927 Olfr1509 0.223423073365418

ENSMUST00000045706 4431 Cftr 0.1554193459334784

ENSMUST00000032094 1203 Fbxl14 0.39598768297723036

ENSMUST00000040484 513 Gcsh 0.2042931946613075

ENSMUST00000030127 879 Tmem38b 0.20832744923719146

ENSMUST00000037748 2403 Hnrnpu 0.21536765108973477

ENSMUST00000045788 1110 H2afy 0.2746662644845894

ENSMUST00000047726 930 Slc39a2 0.2147104870886377

ENSMUST00000035208 11829 Bsn 0.30109429228607426

ENSMUST00000037270 2514 D15Ertd621e 0.1796845922334743

ENSMUST00000032109 1038 Ino80b 0.2999811388191731

ENSMUST00000040856 924 Tmco5b 0.22279932126220525

ENSMUST00000042529 2775 Inpp4b 0.19659381489907624

ENSMUST00000038332 1263 Ctu1 0.31764832863457576

ENSMUST00000031246 975 Ibsp 0.2372827387354335

ENSMUST00000030025 1884 Nr4a3 0.2722304271285972

ENSMUST00000035196 1023 Hemk1 0.2448699406174038

ENSMUST00000046259 2196 Sun2 0.3200423029695344

ENSMUST00000043314 1506 Cyp2s1 0.2839314388699686

ENSMUST00000034435 792 Ctrb1 0.366346564668366

ENSMUST00000039959 708 1810046K07Rik 0.28414472884863895

ENSMUST00000047734 2682 Zfp281 0.20853223084438982

ENSMUST00000044011 327 Fkbp1a 0.28208404043061436

ENSMUST00000039694 708 Stard3nl 0.1742551776247355

ENSMUST00000036876 1227 Gm13178 0.18931780994363429

ENSMUST00000032988 1020 Prss8 0.2845693679788339

ENSMUST00000045896 1326 Pacsin1 0.3914895901980464

ENSMUST00000040001 1815 Galnt9 0.34890042323899884

ENSMUST00000034187 1989 Mmp2 0.3333863140118473

ENSMUST00000031250 1053 Nudt9 0.29124080252965584

ENSMUST00000040179 3987 Ttll5 0.2181900253375631

ENSMUST00000033230 1203 Tha1 0.298839423963382

ENSMUST00000048012 2736 5330417C22Rik 0.30228883982966587

ENSMUST00000037023 171 Rps29 0.3821787887226393

ENSMUST00000037913 450 Rmi2 0.29227776305638614

ENSMUST00000047098 2058 Mslnl 0.29555313978818

ENSMUST00000044105 999 Tspan10 0.28105177755022337

ENSMUST00000046748 1011 Gpr160 0.20315038070678748

ENSMUST00000032200 1887 Slc6a12 0.3214346620821718

ENSMUST00000032856 1815 Me3 0.3136701128113732

ENSMUST00000042046 1821 Scara3 0.29947164602462656

ENSMUST00000046005 2370 Glis1 0.3392145796370092

ENSMUST00000047153 444 Lce1f 0.3400254845363324

ENSMUST00000033331 723 Nrip3 0.2464976702487455

ENSMUST00000042617 2064 Clcnka 0.3338544410558415

ENSMUST00000044003 4875 Abca6 0.17188194350339417

ENSMUST00000039900 510 Prorsd1 0.14955015842734073

ENSMUST00000040423 372 Cd59a 0.1809795153136537

ENSMUST00000045487 786 Rhou 0.31813250625315564

ENSMUST00000030698 825 Stx12 0.2119678856327595

ENSMUST00000044326 1050 2300002M23Rik 0.2150819829094123

ENSMUST00000049813 1032 Yod1 0.2234125119958268

ENSMUST00000048265 957 Olfr617 0.19921693399824122

ENSMUST00000049149 13638 Lrp1 0.36445473723680716

ENSMUST00000031103 1482 Ugdh 0.2243953832285914

ENSMUST00000036682 1749 Pxk 0.22538541618100175

ENSMUST00000033591 792 Itm2a 0.22049763353052407

ENSMUST00000038684 870 Fhl3 0.33217886036423194

ENSMUST00000038701 1752 Zfp324 0.27510638287840594

ENSMUST00000037547 4038 Disp2 0.27312117497421806

ENSMUST00000040538 1290 Sccpdh 0.19092852336401842

ENSMUST00000031122 1425 Gabrb1 0.23885875367186232

ENSMUST00000032775 465 Lrrc28 0.20023614843463522

ENSMUST00000031534 2154 Mad1l1 0.36561786094545656

ENSMUST00000040008 717 Ube2r2 0.26683667708726794

ENSMUST00000032802 1680 Zfp84 0.29075244282911594

ENSMUST00000039610 1656 Xylb 0.25378651975130484

ENSMUST00000034051 1386 Ufsp2 0.18994036578936796

ENSMUST00000031398 1182 Hpd 0.3207870788126781

ENSMUST00000042944 4617 Arap3 0.2686410846443901

ENSMUST00000031805 1950 Avl9 0.18275492540350247

ENSMUST00000039259 3561 Agap2 0.28012642534842186

ENSMUST00000044547 1563 Ceacam2 0.20195899255364508

ENSMUST00000044385 915 Sgtb 0.20866612589267833

ENSMUST00000030581 1380 Azin2 0.340961044388561

ENSMUST00000049124 1449 C1rl 0.2865580782492892

ENSMUST00000040594 525 Ermard 0.19553194776554522

ENSMUST00000038608 1422 Mboat7 0.3386419252750484

ENSMUST00000044405 1035 Lpar6 0.18733860372057637

ENSMUST00000048068 1248 Arrdc4 0.226846731494511

ENSMUST00000034373 1437 Dpep2 0.2313017314042981

ENSMUST00000033142 1626 Ptpn5 0.3405075395536579

ENSMUST00000037190 1041 2700029M09Rik 0.17666164353928496

ENSMUST00000033179 1374 Rrp8 0.22314226865734116

ENSMUST00000033121 3645 Nomo1 0.29184442208536127

ENSMUST00000030317 519 Pdpn 0.18958785868786796

ENSMUST00000044166 1740 Cdc40 0.19703570286117006

ENSMUST00000047226 2850 Lonp1 0.32189978294121363

ENSMUST00000048613 1380 A830018L16Rik 0.1649711127678947

ENSMUST00000048937 2004 Leo1 0.2633598149603677

ENSMUST00000030436 423 Pnrc2 0.177920469729972

ENSMUST00000031278 1407 Bmp3 0.24600747912908677

ENSMUST00000031324 489 Ereg 0.22484664342795763

ENSMUST00000036819 963 9130409I23Rik 0.17391302906420497

ENSMUST00000046049 1599 Fmo1 0.22694636941915225

ENSMUST00000048941 2112 Capn8 0.29231267040319675

ENSMUST00000033880 354 Eif4ebp1 0.3208928747578573

ENSMUST00000032125 1266 Bmp10 0.2819338351995354

ENSMUST00000041282 2886 Trim37 0.1783069604191307

ENSMUST00000047134 942 Sdr9c7 0.310366089795676

ENSMUST00000038439 912 Dnajc17 0.28414386902074407

ENSMUST00000047877 1335 Dok3 0.3138342736835946

ENSMUST00000031333 930 Gtf2h3 0.29144819928473337

ENSMUST00000034564 426 2310030G06Rik 0.2864758579986384

ENSMUST00000050027 1065 Nov 0.2975398591660825

ENSMUST00000040213 2166 Tyw1 0.3026774114630377

ENSMUST00000030284 465 Rnf11 0.2046359034884632

ENSMUST00000033581 624 Fgf16 0.3008333504466116

ENSMUST00000033010 1245 Kdm8 0.3369015679106986

ENSMUST00000041252 1668 Acad12 0.2838710745795822

ENSMUST00000034834 1596 Pkm 0.3636857014826149

ENSMUST00000036227 1074 Htr5a 0.3044021504902455

ENSMUST00000043249 741 Mcpt4 0.2158531171366854

ENSMUST00000047864 2577 Eef2 0.398416718523704

ENSMUST00000038507 903 Ly6g6f 0.25320791581242186

ENSMUST00000034982 1158 Tfdp2 0.17692180911633065

ENSMUST00000049724 939 Olfr1443 0.21942500675928758

ENSMUST00000034902 669 Gsta2 0.25341691342231687

ENSMUST00000031034 1608 Nrbp1 0.27264541387353763

ENSMUST00000050083 2913 Cul4b 0.15482015294908472

ENSMUST00000044311 1878 Colgalt2 0.3160813748740267

ENSMUST00000030110 798 Zdhhc21 0.19192305772525503

ENSMUST00000032180 1050 Wnt7a 0.37527015107619865

ENSMUST00000038116 6126 Ankrd12 0.16562540551123867

ENSMUST00000039071 828 Cacng5 0.32038443095087893

ENSMUST00000035852 1893 Zcwpw1 0.23892936863692324

ENSMUST00000037918 744 Tmem33 0.18330711520609186

ENSMUST00000035106 981 Slc25a38 0.2868319798833894

ENSMUST00000041175 1089 Ptger3 0.32425847845521966

ENSMUST00000039165 2277 Golga1 0.19359187514337717

ENSMUST00000030290 1749 Inadl 0.22539984954876274

ENSMUST00000033449 855 1700080O16Rik 0.18933679629401934

ENSMUST00000042610 2937 AI607873 0.17040234704655585

ENSMUST00000047243 2184 Rap1gap 0.33576763458744696

ENSMUST00000032770 393 Pgpep1l 0.2523115667220994

ENSMUST00000040514 3966 Irs2 0.29988153650976834

ENSMUST00000041374 1389 Manea 0.17341515067904945

ENSMUST00000043325 1071 Hs2st1 0.29005072222024236

ENSMUST00000038665 2667 Ptchd1 0.19857777717916708

ENSMUST00000030614 1791 CK137956 0.32460149873748007

ENSMUST00000041840 2628 Spef2 0.16370010762597687

ENSMUST00000037232 5265 Rreb1 0.31893852041998655

ENSMUST00000037607 1821 Map3k7 0.20608556248884005

ENSMUST00000033532 3819 Aff2 0.21237596590726632

ENSMUST00000043966 759 Mrpl47 0.1781165603190403

ENSMUST00000038584 2301 Tlk1 0.1645719139591706

ENSMUST00000043873 315 Scgb3a1 0.26922531421081886

ENSMUST00000047480 8475 Piezo2 0.26674943796964534

ENSMUST00000038815 2292 Sox5 0.26553771829018813

ENSMUST00000045262 1689 Ak5 0.25636073855573477

ENSMUST00000032194 1236 Bhlhe40 0.22959582136157036

ENSMUST00000032508 1377 Fkbp4 0.34417922973628884

ENSMUST00000032661 1824 Zfp819 0.23397990858702666

ENSMUST00000037502 1356 Fam117a 0.30148016098111974

ENSMUST00000039554 1494 Trmt6 0.2007786134837307

ENSMUST00000030490 3789 Stil 0.1723753699737366

ENSMUST00000032738 1509 Chrna7 0.30355778187969534

ENSMUST00000034854 1488 Chrnb4 0.3587425921473773

ENSMUST00000041407 621 Sostdc1 0.3223673147862342

ENSMUST00000047218 774 Reep4 0.30738946682003543

ENSMUST00000049385 342 Eif1 0.22244427484141252

ENSMUST00000032421 1068 St8sia1 0.2793289540400283

ENSMUST00000043521 924 Sec22a 0.20719845177689

ENSMUST00000038743 2085 Tmc4 0.2685755912711497

ENSMUST00000030315 3570 Pum1 0.21893097163386546

ENSMUST00000038356 1269 Ube2q1 0.2816925037826183

ENSMUST00000038160 1017 Lum 0.21379138003229362

ENSMUST00000035850 4263 Npat 0.1512548586026489

ENSMUST00000033919 1248 Vegfc 0.2500220506131621

ENSMUST00000031005 3018 Evc 0.3007761192980448

ENSMUST00000034964 837 Tipin 0.15343069568257622

ENSMUST00000047357 2349 Cpsf2 0.20743605031854268

ENSMUST00000047870 939 Olfr1015 0.19665209496883962

ENSMUST00000032462 675 Timp4 0.29280184010492016

ENSMUST00000033023 786 Aqp8 0.25836828965764774

ENSMUST00000030487 1524 Cyp4a14 0.2554902471980497

ENSMUST00000037580 3717 Kcnt1 0.36205689356936305

ENSMUST00000031055 3054 Emilin1 0.26975935423104286

ENSMUST00000033545 642 Rab39b 0.24002831313687195

ENSMUST00000044018 1335 Noxa1 0.2731473635093952

ENSMUST00000040580 2214 Syde1 0.25980496703117106

ENSMUST00000046937 1098 Tssk1 0.31585500653779375

ENSMUST00000043735 2934 Mthfd1l 0.23032367442234336

ENSMUST00000047904 753 Hoxd4 0.3350778870496971

ENSMUST00000042121 786 H2-DMa 0.2983352340145384

ENSMUST00000032270 735 Klrc1 0.21092421594767663

ENSMUST00000039818 1689 Aldh4a1 0.3396789985738924

ENSMUST00000040536 378 Batf 0.38731276460964686

ENSMUST00000030469 720 Lurap1 0.3217603808810889

ENSMUST00000032729 5058 Tjp1 0.18195328332827293

ENSMUST00000033567 1002 Awat2 0.27008471172811277

ENSMUST00000030536 1743 Pink1 0.31591033805448276

ENSMUST00000037992 1950 Ssh3 0.28267906554835676

ENSMUST00000035390 924 Azgp1 0.27496937835317253

ENSMUST00000048246 1446 Fgb 0.23491823420793556

ENSMUST00000031281 1464 Antxr2 0.19597787366146488

ENSMUST00000046951 1149 Pak1ip1 0.2197503238394367

ENSMUST00000033920 1041 Aga 0.18875748358074618

ENSMUST00000048967 1431 Cpe 0.2816209344335389

ENSMUST00000033229 444 Hbb-y 0.2569219873630726

ENSMUST00000038004 1341 Krt25 0.33791140528777286

ENSMUST00000033842 4392 Myom2 0.26773666316057565

ENSMUST00000034592 6336 Dscaml1 0.3380982310424719

ENSMUST00000045730 5055 Akap12 0.2336699686217713

ENSMUST00000037557 5127 Mon2 0.1912564855829242

ENSMUST00000032133 534 Gp9 0.35126569884912323

ENSMUST00000040907 879 Decr2 0.24268130484340034

ENSMUST00000034365 2115 Tsnaxip1 0.2739414746198213

ENSMUST00000044189 1923 Senp1 0.1988817758075319

ENSMUST00000030842 573 Lzic 0.2354165039999953

ENSMUST00000032066 480 Tgfa 0.33848029570319904

ENSMUST00000034868 1452 Clk1 0.16971954644659684

ENSMUST00000042196 1248 Vwa1 0.27501088619468333

ENSMUST00000049393 1320 Zfp113 0.22901952124205288

ENSMUST00000033737 1095 Haus7 0.26675097648381385

ENSMUST00000043273 663 U2af1l4 0.25761393331796567

ENSMUST00000032906 387 Calca 0.3414297774259025

ENSMUST00000042261 1869 Slc2a12 0.20955043379066396

ENSMUST00000034793 1260 Lca5 0.19332702855837047

ENSMUST00000031143 993 Srd5a3 0.2945124863635445

ENSMUST00000037783 1404 Ccdc174 0.1777272772064227

ENSMUST00000038738 1692 Cdkn2aip 0.1926399349169757

ENSMUST00000041059 2394 Trpc4ap 0.26492788354589064

ENSMUST00000031287 1173 Tpst2 0.38374946943569704

ENSMUST00000048935 1431 Dmrt3 0.3274154620094288

ENSMUST00000031798 735 Ssmem1 0.2521783654745621

ENSMUST00000035977 5670 Ticrr 0.22090092900515568

ENSMUST00000031390 1737 Mmp17 0.29943139712743067

ENSMUST00000042971 540 Arl5c 0.2914231894006032

ENSMUST00000039476 4659 Arhgef11 0.2688947204967419

ENSMUST00000034313 573 Ntpcr 0.2881838090881372

ENSMUST00000032220 828 Cops7a 0.29458235357185997

ENSMUST00000047383 372 Kcne2 0.37772834204140376

ENSMUST00000030404 906 Ppie 0.298523112501332

ENSMUST00000033086 1221 Phkg2 0.276585515754586

ENSMUST00000034370 3264 Slc12a4 0.3486742115508162

ENSMUST00000043805 1482 Foxred1 0.2375615921156406

ENSMUST00000037370 3480 Sorcs2 0.32617765622982375

ENSMUST00000030528 435 Pla2g2d 0.35019770448980836

ENSMUST00000031170 2721 Cenpc1 0.1500362579427045

ENSMUST00000049324 3003 Flt3 0.24594700940102873

ENSMUST00000034017 1782 Klhl2 0.21638846371472037

ENSMUST00000034811 1512 Cyp19a1 0.2610780725473498

ENSMUST00000038835 345 Scgb2b2 0.18368905730235985

ENSMUST00000033442 3954 Igsf1 0.22669383741876026

ENSMUST00000038361 2223 Mgat5 0.29963856957111956

ENSMUST00000030964 915 Cd38 0.25339407295166444

ENSMUST00000038757 465 Csdc2 0.3629377042449205

ENSMUST00000042755 1818 Afp 0.22173659467362097

ENSMUST00000049346 1323 Asic3 0.3030916645941889

ENSMUST00000033739 954 Car5b 0.215756375818154

ENSMUST00000037480 1323 Wipf2 0.2549076160473961

ENSMUST00000043112 2241 Fbxo34 0.25236432857365493

ENSMUST00000044138 1455 Chst7 0.3082094382165607

ENSMUST00000034325 1047 Lpar2 0.3144992268091099

ENSMUST00000034219 729 Syce1l 0.23272373212336092

ENSMUST00000048430 819 Cer1 0.2755622455898321

ENSMUST00000038693 663 Cldn22 0.294207871555758

ENSMUST00000045028 525 Crygd 0.4398776889808916

ENSMUST00000033909 561 Tex29 0.24189214210605653

ENSMUST00000031045 765 Yipf7 0.20673930363906037

ENSMUST00000033486 459 Plp2 0.21022576605794732

ENSMUST00000035934 2268 Exoc3 0.25782665263494864

ENSMUST00000034012 858 Cnot7 0.17232699249304645

ENSMUST00000031976 1800 Prdm5 0.2790310719514036

ENSMUST00000033717 819 Hccs 0.20986401854813272

ENSMUST00000045717 6405 Tdrd6 0.2258036837027053

ENSMUST00000046156 420 Sct 0.3227735901903794

ENSMUST00000033450 1674 Gpc4 0.25042791903934036

ENSMUST00000049625 1692 Zfp879 0.24523494263598555

ENSMUST00000036473 2241 Pomt1 0.27346680861356293

ENSMUST00000039088 1056 Tex19.1 0.2577786070106868

ENSMUST00000034426 1788 Kars 0.297599917999909

ENSMUST00000039305 1437 Slc36a2 0.3386729691544369

ENSMUST00000033606 1407 Srpx2 0.24236181319021477

ENSMUST00000049577 2094 Adam26a 0.14849870978320873

ENSMUST00000044106 966 Psmd7 0.2811350500657622

ENSMUST00000037907 2781 Ddx58 0.2210526068866059

ENSMUST00000044551 1890 Ick 0.23515623505353914

ENSMUST00000044373 696 Rfxap 0.2797681112036001

ENSMUST00000034464 969 2310022B05Rik 0.3372264197722519

ENSMUST00000035020 2124 Eomes 0.27881010675476947

ENSMUST00000031583 7347 Acacb 0.35286946061994157

ENSMUST00000034585 1188 Apoa4 0.40727094812525905

ENSMUST00000046838 2574 Adam22 0.21383137756401543

ENSMUST00000039450 1662 Mcoln3 0.24573505767668802

ENSMUST00000038696 2454 Ppp1r9b 0.3371302502908724

ENSMUST00000042097 294 Stfa1 0.18223896930102568

ENSMUST00000031542 1527 Oasl2 0.2845028087589047

ENSMUST00000048453 1569 Btbd16 0.2974254497216819

ENSMUST00000030399 1197 Rragc 0.20818063728759723

ENSMUST00000030395 201 Ccdc23 0.3251425438923731

ENSMUST00000040990 5718 Vwa8 0.22431489071900568

ENSMUST00000050227 930 Olfr868 0.1636688819282752

ENSMUST00000034473 465 Spata19 0.2113399087986851

ENSMUST00000030266 1110 B4galt2 0.3455069785194188

ENSMUST00000032248 789 Clec4a2 0.1882381505954347

ENSMUST00000034842 1170 Neil1 0.27956700520537175

ENSMUST00000044579 1080 Crlf2 0.3291956244050771

ENSMUST00000035164 4548 Topbp1 0.18219591461051574

ENSMUST00000042852 822 Fam210a 0.1835348270289473

ENSMUST00000030013 819 Xpa 0.23228484389654608

ENSMUST00000049333 312 Kcne3 0.26331835763627104

ENSMUST00000033683 792 Rps4x 0.24179638878669504

ENSMUST00000041425 990 Negr1 0.23489416229257098

ENSMUST00000050034 2361 Pcdhb15 0.20843277930065257

ENSMUST00000044660 2781 Armc5 0.23183470247937157

ENSMUST00000043189 3474 Nfasc 0.3293610046026543

ENSMUST00000031835 2271 Aoc1 0.2833244104607705

ENSMUST00000040656 876 Bphl 0.23509800498569308

ENSMUST00000041819 1632 Tulp1 0.3321416047574406

ENSMUST00000043172 1377 Rarg 0.33521044655058485

ENSMUST00000045817 2103 Kirrel2 0.2513224885663105

ENSMUST00000038907 1668 Wee2 0.2043049647686375

ENSMUST00000032191 1119 Sumf1 0.24843626770611385

ENSMUST00000033828 2025 Gas6 0.30791961061657097

ENSMUST00000049156 1368 Tldc1 0.3456426864033226

ENSMUST00000040260 1779 Frrs1 0.20692119960023747

ENSMUST00000050096 2025 Add3 0.25425608226170654

ENSMUST00000039046 486 Il17f 0.2756854908839582

ENSMUST00000045766 1974 Wdr70 0.210635923167767

ENSMUST00000046332 741 C1qc 0.3275411854355229

ENSMUST00000035430 1962 Dars2 0.1838807043671667

ENSMUST00000038446 519 Myl12b 0.4358811460287816

ENSMUST00000031096 3132 Klb 0.2602991765557361

ENSMUST00000031718 1050 Pax4 0.23926879695340028

ENSMUST00000035777 1662 Mon1b 0.2944719775656534

ENSMUST00000032781 1737 Nox4 0.18086256565543665

ENSMUST00000031668 4119 Col1a2 0.18741706070605696

ENSMUST00000046945 1152 Palm 0.33098797903904825

ENSMUST00000034521 2313 4931429I11Rik 0.23023755701998724

ENSMUST00000035426 939 3110001I22Rik 0.1769721561542321

ENSMUST00000043931 486 Atp5h 0.3298779591124164

ENSMUST00000033865 1530 Nek3 0.2172971503809717

ENSMUST00000032414 2784 Ttll3 0.3210326454009059

ENSMUST00000041705 2373 Spata20 0.3075499675718009

ENSMUST00000035822 972 Calhm2 0.3430263484977302

ENSMUST00000039480 3306 Zswim4 0.3183425790634392

ENSMUST00000034408 1272 Gpr83 0.319044045040434

ENSMUST00000047664 1455 Arhgef4 0.3732373142560643

ENSMUST00000030676 2760 Grik3 0.358125690845056

ENSMUST00000039517 3945 Syde2 0.23540036083191795

ENSMUST00000040280 1404 Slc25a23 0.3014003099254476

ENSMUST00000034029 1284 Ednra 0.2849855328609963

ENSMUST00000049255 2610 Armc3 0.22430380582887477

ENSMUST00000048118 915 Hsd17b13 0.22390316196742452

ENSMUST00000040105 3408 Specc1l 0.23301862660043815

ENSMUST00000040271 2286 Cep85 0.24387095898167122

ENSMUST00000036177 975 En2 0.31846529167367704

ENSMUST00000032803 1647 Zfp30 0.295703614875975

ENSMUST00000040250 1440 Kmo 0.22299561712734783

ENSMUST00000044533 1815 Prss56 0.24776544783817814

ENSMUST00000044634 897 Slc25a21 0.19893586184853845

ENSMUST00000047488 2139 Rap1gap2 0.32502293188008646

ENSMUST00000033478 2835 Mcf2 0.16437963100815822

ENSMUST00000047521 1779 Cercam 0.31336575892771107

ENSMUST00000031524 1239 Acads 0.3422198819736823

ENSMUST00000042753 1242 Rbm48 0.17368384674977655

ENSMUST00000040021 5079 Ptpn23 0.2877911896502908

ENSMUST00000048096 2508 Tlr4 0.1656604710093029

ENSMUST00000037360 711 Rhov 0.2832914617537424

ENSMUST00000035372 525 Ascl3 0.36245446009380633

ENSMUST00000035515 3768 Abcb5 0.17790585026050476

ENSMUST00000034351 924 Rrad 0.2917052188766339

ENSMUST00000031119 1398 Gabrg1 0.17412218753818537

ENSMUST00000037633 2913 Zc3h7a 0.16707118069628185

ENSMUST00000032541 555 2900092C05Rik 0.11923303806777895

ENSMUST00000031445 2019 Aacs 0.3553051254552185

ENSMUST00000048642 1134 Parl 0.20262307534492177

ENSMUST00000034620 786 Acrv1 0.1711935565825186

ENSMUST00000041262 339 Churc1 0.28501501718507743

ENSMUST00000049506 1287 Zrsr1 0.25100925300848614

ENSMUST00000033099 633 Fgf21 0.2912919468710063

ENSMUST00000043696 1476 Serpinf2 0.3065726759123496

ENSMUST00000031024 198 Mrpl33 0.24165079144206783

ENSMUST00000035220 1209 Prkar2a 0.22964314127289104

ENSMUST00000040110 1059 Chst11 0.43611334286643516

ENSMUST00000035154 468 1110059G10Rik 0.17154185209083575

ENSMUST00000037302 2097 Tbc1d12 0.2042282035221047

ENSMUST00000038920 1278 Gpr63 0.1978218402698388

ENSMUST00000034647 1224 Zfp558 0.17136345122001095

ENSMUST00000047698 864 Stx3 0.3259555623972802

ENSMUST00000039744 1887 Lnx1 0.26144941757373086

ENSMUST00000043938 3387 Plcl2 0.24718117045971721

ENSMUST00000044111 657 Rras 0.33421155671471453

ENSMUST00000043317 597 Dnajc12 0.27597606453260004

ENSMUST00000029920 1641 Odf2l 0.1600405934473609

ENSMUST00000038570 855 Nipsnap1 0.2880650831568144

ENSMUST00000038863 2709 Lars2 0.2718275147955847

ENSMUST00000044195 2181 Tmc7 0.2993903008886229

ENSMUST00000030443 3549 Ptch2 0.3096469589805285

ENSMUST00000032969 1386 Pold3 0.2216607858774146

ENSMUST00000041180 1047 Taar9 0.21897849893154842

ENSMUST00000038196 1686 Mks1 0.2998358382288645

ENSMUST00000049501 3060 Ofd1 0.16469541808312352

ENSMUST00000030803 372 Uts2 0.2609418315133364

ENSMUST00000032130 1500 Aplf 0.2146648412241453

ENSMUST00000041126 1209 Ss18l1 0.37213167768387356

ENSMUST00000043397 2391 Plekhh3 0.2885306766446111

ENSMUST00000030018 1080 Nans 0.3408854220154227

ENSMUST00000043305 2034 Wdtc1 0.3346990601641488

ENSMUST00000036041 2376 Ap1g2 0.28706982448384066

ENSMUST00000031895 1359 Casp2 0.25400670713947443

ENSMUST00000040687 375 Tax1bp3 0.2512188510688002

ENSMUST00000033210 486 Tmem159 0.24601382489462995

ENSMUST00000048677 1518 Tbc1d22b 0.3096908390045423

ENSMUST00000042615 1494 Ralgps1 0.24771477409710757

ENSMUST00000047419 510 Tspo 0.2967628525980652

ENSMUST00000040729 2412 Clcn7 0.2903334484900676

ENSMUST00000043990 1527 Edc3 0.2558260531970469

ENSMUST00000033821 1482 F10 0.3118523809506647

ENSMUST00000043296 2427 Dlgap5 0.2100188383775411

ENSMUST00000042675 783 Capzb 0.2953776186320616

ENSMUST00000042767 2874 Slitrk5 0.25267475682008816

ENSMUST00000030826 1506 Slc2a5 0.2767549325939668

ENSMUST00000049744 537 Mplkip 0.2323752204286899

ENSMUST00000038455 1161 Skint3 0.13735844923564783

ENSMUST00000042661 1575 Ttyh3 0.3559070749592219

ENSMUST00000038845 660 Cd53 0.21240277401379737

ENSMUST00000045633 4035 Mybbp1a 0.30322635773768286

ENSMUST00000036649 1722 Coil 0.23867497356723874

ENSMUST00000046303 372 Crebl2 0.2588187169577595

ENSMUST00000036880 1083 Cdk10 0.3268787223930196

ENSMUST00000030773 837 Xrcc2 0.19459523369243825

ENSMUST00000041139 624 Rab8b 0.21746008060575614

ENSMUST00000041369 1611 Socs5 0.21581102047593903

ENSMUST00000040344 1635 Gns 0.3082064917695409

ENSMUST00000044583 2538 Vmn2r65 0.1547713259478858

ENSMUST00000040372 240 Tmem258 0.3295281713251833

ENSMUST00000031492 606 Rab35 0.3173807560150826

ENSMUST00000030902 3828 Prdm16 0.3331454890452901

ENSMUST00000048604 1680 Msantd2 0.22784008700272707

ENSMUST00000046999 924 Abhd11 0.2227132550738934

ENSMUST00000037839 807 Zfand1 0.2300323791185986

ENSMUST00000030568 2256 Sema3c 0.2216811895634076

ENSMUST00000031318 399 Cxcl5 0.2023621129914384

ENSMUST00000041316 351 Tcl1 0.3260751264125919

ENSMUST00000041124 1701 Zfp704 0.31025833748412446

ENSMUST00000035648 426 Atg12 0.21339133916604272

ENSMUST00000031104 3999 Pds5a 0.16573574201876098

ENSMUST00000041892 4026 Wdr19 0.23558592298331066

ENSMUST00000040153 1413 Rorb 0.25884605544028355

ENSMUST00000043734 360 Omt2b 0.19067990870007898

ENSMUST00000046128 2031 Uroc1 0.30382461380534453

ENSMUST00000049780 3258 Depdc5 0.2500217997262192

ENSMUST00000034529 2568 Cul5 0.1541826600334155

ENSMUST00000042901 3861 Smc4 0.1420166535212578

ENSMUST00000047721 1428 Rrp9 0.324884503667809

ENSMUST00000032766 1497 Rhcg 0.28424670867784857

ENSMUST00000037958 3801 Arhgap29 0.16927370571449843

ENSMUST00000040865 681 Tmem98 0.31579936144334597

ENSMUST00000034862 1617 AI118078 0.2975793682289469

ENSMUST00000045692 1440 Fbxl16 0.2963842468870813

ENSMUST00000043336 1080 St8sia4 0.18148591269941805

ENSMUST00000032372 417 Golt1b 0.21106968446228083

ENSMUST00000043183 1683 Ces2g 0.2812338647767058

ENSMUST00000031351 690 Arl6ip4 0.29194253153435007

ENSMUST00000038212 792 Gzmk 0.21530023411989174

ENSMUST00000032717 1008 Dbx1 0.27987414664410565

ENSMUST00000045902 1608 Fmo2 0.2508513472660289

ENSMUST00000034465 846 2810004N23Rik 0.2175601280573114

ENSMUST00000043204 1689 Fbxo33 0.2194488228827998

ENSMUST00000030677 3876 Map3k6 0.29119687405772754

ENSMUST00000046558 432 Hspb11 0.1831605351517158

ENSMUST00000031853 567 Npvf 0.21972317981705133

ENSMUST00000049789 4212 Naip5 0.20322479255638984

ENSMUST00000049430 1869 Galnt18 0.36486888193057787

ENSMUST00000034941 981 Plscr4 0.23589309392333144

ENSMUST00000036033 4689 Dip2a 0.2818699276564591

ENSMUST00000039538 3684 Arid4b 0.17126581267174235

ENSMUST00000036554 3978 Abcc4 0.22912520289773264

ENSMUST00000046521 4278 Bcl9 0.2627650652057869

ENSMUST00000047714 4635 Kdm5b 0.21178171519493894

ENSMUST00000030658 1293 Tekt2 0.313478485271535

ENSMUST00000040117 2577 Secisbp2 0.2705679399078732

ENSMUST00000032683 771 Lypd5 0.392150773208498

ENSMUST00000046890 1080 Rassf7 0.2859292985318657

ENSMUST00000041725 1647 Mgat4b 0.3246730629041723

ENSMUST00000034746 1476 Mns1 0.28933087766674026

ENSMUST00000043237 666 Trp53inp2 0.3129223353915733

ENSMUST00000045235 1212 B630005N14Rik 0.18341377654925045

ENSMUST00000031090 3414 Sel1l3 0.24438544381350408

ENSMUST00000040706 2136 Cnst 0.25271606850732564

ENSMUST00000045855 573 Hebp1 0.27333940832303644

ENSMUST00000031256 3681 Aff1 0.2735288818061347

ENSMUST00000033427 1143 Sash3 0.29756639480163893

ENSMUST00000049089 621 Twist1 0.41106709462758956

ENSMUST00000032201 3348 Ret 0.2993310899799755

ENSMUST00000042402 4203 Pogz 0.23814880054927434

ENSMUST00000031472 582 Pxmp2 0.24889394414785848

ENSMUST00000038193 2982 Wdr59 0.27516066288883134

ENSMUST00000030513 717 Mrto4 0.3121387413660504

ENSMUST00000046168 981 Mpped1 0.3620169055670088

ENSMUST00000039697 1422 Gabrb3 0.2417177802612058

ENSMUST00000035129 2955 Ephb1 0.3050341358238904

ENSMUST00000032820 1023 Zscan26 0.22081786006990994

ENSMUST00000037708 1620 Asic4 0.3457964610103927

ENSMUST00000043517 1227 Pvr 0.24540785113040842

ENSMUST00000037119 768 Dlx1 0.28724308714855123

ENSMUST00000030925 1350 Gabrd 0.31632178752987833

ENSMUST00000031732 1770 Fbxo24 0.3174451978366852

ENSMUST00000032573 549 Pglyrp1 0.30570902259650196

ENSMUST00000030032 1119 Tmeff1 0.23116182494112253

ENSMUST00000032920 642 Cdipt 0.30522484931041516

ENSMUST00000040746 576 Tcte3 0.1515704232231782

ENSMUST00000033265 2127 Zranb1 0.16555196935892777

ENSMUST00000035560 849 Acbd6 0.23118551596023895

ENSMUST00000031211 444 Smr3a 0.17217338272438315

ENSMUST00000042221 1971 Slc26a7 0.19017952931777332

ENSMUST00000042923 1005 Sirt6 0.34923204432595756

ENSMUST00000040104 654 Hand2 0.4660309774873454

ENSMUST00000043211 780 Coq10a 0.243784987528775

ENSMUST00000044148 1248 Alg2 0.3096228676394083

ENSMUST00000046285 738 C1qa 0.30386329476306645

ENSMUST00000033839 522 Coprs 0.288524913890886

ENSMUST00000045705 3903 Ambra1 0.23928755213786976

ENSMUST00000031317 1026 Rassf6 0.208469812212092

ENSMUST00000030734 1506 Sf3a3 0.2964701259243362

ENSMUST00000030375 1122 Exo5 0.19911311526658754

ENSMUST00000048197 633 Rhod 0.3097657178459924

ENSMUST00000038558 756 Klf16 0.31887292397197015

ENSMUST00000032899 546 1110004F10Rik 0.23054679267570713

ENSMUST00000041052 630 Hist1h1t 0.28449047589056153

ENSMUST00000035065 1815 Ptgs2 0.2580063077006777

ENSMUST00000032879 612 Rab30 0.20512079280329065

ENSMUST00000036877 3003 Dennd2a 0.29615002688323183

ENSMUST00000045897 336 Ptma 0.30327401424553346

ENSMUST00000050029 855 Six1 0.329681083357513

ENSMUST00000039894 1575 Tbce 0.16156411110051425

ENSMUST00000041342 6984 Cacna1b 0.295615931564324

ENSMUST00000045091 2202 Kirrel3 0.3160430305960457

ENSMUST00000031519 684 Cabp1 0.3091364846692042

ENSMUST00000047393 612 Ctf1 0.3423426385616064

ENSMUST00000046765 1011 Kcnk1 0.4022568091008623

ENSMUST00000048418 3033 Ipo8 0.2232447168115033

ENSMUST00000032203 4425 A2m 0.23814380439451752

ENSMUST00000046735 930 Tex264 0.3030970100530058

ENSMUST00000030152 783 1110017D15Rik 0.3113284712723834

ENSMUST00000044143 558 Rln1 0.20230795657627376

ENSMUST00000031131 672 Uchl1 0.35814964340334654

ENSMUST00000038769 240 S100g 0.27515934693347244

ENSMUST00000038096 930 Fn3krp 0.2468645191253593

ENSMUST00000040577 1257 Rnmtl1 0.24507765179271723

ENSMUST00000040677 1710 Eepd1 0.2781126723683881

ENSMUST00000047349 3156 Usp28 0.21626282207664627

ENSMUST00000033811 2652 Morc4 0.19496699907084294

ENSMUST00000034115 1236 Pou4f2 0.3574429352459547

ENSMUST00000037798 3393 Slc24a1 0.25548515833922875

ENSMUST00000037967 1422 Slc10a3 0.23789017756571618

ENSMUST00000030401 321 Ndufs5 0.29200730693992893

ENSMUST00000036424 2106 Alox12b 0.3118050914721746

ENSMUST00000037182 2157 Hook3 0.15807409185703705

ENSMUST00000031032 1629 Ppm1g 0.273078789771532

ENSMUST00000032399 567 Kras 0.1742875985087865

ENSMUST00000047973 1551 Dhcr24 0.4052607504480508

ENSMUST00000038141 2733 Slfn8 0.2106316360343453

ENSMUST00000040313 858 Bcdin3d 0.2805574506410006

ENSMUST00000041763 702 4930505A04Rik 0.158217995821124

ENSMUST00000047025 3414 Otoa 0.26429002963581266

ENSMUST00000030572 582 Hpca 0.4456230212181811

ENSMUST00000032915 1983 Kif22 0.23839037495962231

ENSMUST00000045153 795 Pik3ip1 0.33397938886091616

ENSMUST00000047034 3927 Ttbk1 0.317649517018295

ENSMUST00000036759 4005 Fam21 0.21236815228090994

ENSMUST00000030201 2262 Gne 0.2669024115114527

ENSMUST00000033500 684 Eras 0.28187470113601565

ENSMUST00000034243 1974 Mmp15 0.34595167521553477

ENSMUST00000037552 3489 Lepr 0.17927730203056308

ENSMUST00000039720 1125 Rragb 0.19569869292696312

ENSMUST00000031195 1605 Ugt2a3 0.1962412368136904

ENSMUST00000032433 1044 Smco2 0.228771845942125

ENSMUST00000032265 1092 Olr1 0.21345738370189263

ENSMUST00000033018 1548 Far1 0.17080017538793418

ENSMUST00000044179 561 Tex13 0.30066623429896827

ENSMUST00000040735 1230 Amdhd2 0.30275580476775255

ENSMUST00000032811 2022 Rasgrp4 0.3119038670734041

ENSMUST00000032127 576 Gkn3 0.25875505363977497

ENSMUST00000037968 2097 Uvrag 0.26250789297167465

ENSMUST00000033433 981 Rbmx2 0.27339132349025164

ENSMUST00000034754 981 Bnip2 0.186004772988237

ENSMUST00000035027 2901 Clstn2 0.29256168738722405

ENSMUST00000036315 2724 Gria1 0.27948564978505247

ENSMUST00000044441 3294 Tmem132d 0.29805195093078046

ENSMUST00000040647 468 Fgf1 0.3052278884106337

ENSMUST00000045229 2037 Tmco3 0.28168750495444145

ENSMUST00000046207 378 Sf3b6 0.19963977092731247

ENSMUST00000039990 2112 P3h2 0.2690347714329316

ENSMUST00000040149 3585 Atp8b4 0.18785243045901925

ENSMUST00000040561 1848 Rundc1 0.2992359389307053

ENSMUST00000048706 2148 Orc3 0.1861907550724998

ENSMUST00000047479 3021 Pcdhac2 0.2532178531013611

ENSMUST00000037448 1935 Zfp109 0.26850213635893466

ENSMUST00000044021 1497 Slc19a2 0.28157716826934404

ENSMUST00000037528 1824 Gdpd5 0.359856615146741

ENSMUST00000035105 888 Rpsa 0.31699491350266995

ENSMUST00000040135 1143 Nup43 0.17242723929270135

ENSMUST00000038014 2319 Dnajc16 0.311712775543551

ENSMUST00000045140 2769 Tcaf1 0.23171321699287886

ENSMUST00000031347 594 Rilpl2 0.38082119116410457

ENSMUST00000047954 1116 Dtna 0.23268710377012944

ENSMUST00000049105 1893 Klhl14 0.31333169473429795

ENSMUST00000036194 693 Rep15 0.2820981921006593

ENSMUST00000038829 1269 Fcmr 0.2391010881746177

ENSMUST00000041400 1947 Ranbp10 0.2940265774947491

ENSMUST00000039718 930 Olfr1353 0.16638274923363913

ENSMUST00000038794 2589 Dpp9 0.3614587159195258

ENSMUST00000041686 927 Nudt22 0.28898721995693044

ENSMUST00000042564 1041 Ghitm 0.1824263346770913

ENSMUST00000029948 1509 Bmpr1b 0.23078379562083703

ENSMUST00000034311 417 Lsm4 0.3852843344648685

ENSMUST00000030992 585 Rnf4 0.2154580769566202

ENSMUST00000045008 717 Cml2 0.21456629954184156

ENSMUST00000038225 2172 Slco4a1 0.3081400609382363

ENSMUST00000031003 1344 Ppp2r2c 0.3798186323279679

ENSMUST00000038406 951 Akr1b8 0.3090794715113225

ENSMUST00000032078 1635 Cct7 0.2818820255848195

ENSMUST00000031078 603 1700001C02Rik 0.29482632981916385

ENSMUST00000040776 1548 Cenpt 0.19949037793821384

ENSMUST00000036155 2361 Xrra1 0.28702685826520313

ENSMUST00000036383 777 Dnali1 0.360714507402176

ENSMUST00000029941 1776 Pdlim5 0.22898212778218677

ENSMUST00000036951 564 Pebp1 0.37280357477725423

ENSMUST00000034611 4116 Phldb1 0.3103708136506984

ENSMUST00000049852 318 Prok1 0.32563535798120313

ENSMUST00000041099 1074 Neurod1 0.30720258046658433

ENSMUST00000031058 846 Mapre3 0.2818664272511405

ENSMUST00000034227 549 Pllp 0.29890525543394614

ENSMUST00000037991 3309 Mical2 0.30896830748260457

ENSMUST00000035038 540 Faim 0.23518049987175146

ENSMUST00000043531 3672 Fam65a 0.26849922650707014

ENSMUST00000047322 2682 Rbbp8 0.16515943300447955

ENSMUST00000042808 2463 Scarf1 0.31307447547800216

ENSMUST00000044352 3534 Pcnxl4 0.1842582942633074

ENSMUST00000030944 1557 Ccnl2 0.23442979749688733

ENSMUST00000030949 2577 Tas1r3 0.27562023891632825

ENSMUST00000048077 810 Lime1 0.297188024391303

ENSMUST00000046875 4656 Uggt1 0.2236423362322724

ENSMUST00000033854 183 Defb8 0.20524493993264872

ENSMUST00000036136 819 Colec11 0.31367003561610685

ENSMUST00000047446 2499 Sidt1 0.29310298944351476

ENSMUST00000031587 921 Ung 0.3051463568797399

ENSMUST00000039016 2016 Lzts2 0.30803174515860426

ENSMUST00000049411 954 Apex1 0.2136855147687778

ENSMUST00000030365 1272 Mrpl37 0.24570022541160635

ENSMUST00000037623 429 Nrn1 0.24175527050163687

ENSMUST00000039178 4683 Tnn 0.32448356598612604

ENSMUST00000037854 3309 Diap2 0.17584382415867905

ENSMUST00000034827 555 Imp3 0.33140359717012746

ENSMUST00000042498 3807 Hdlbp 0.24890186562776062

ENSMUST00000030339 1497 Tnfrsf8 0.28491396446612416

ENSMUST00000038172 2244 Mtmr12 0.2426202559814897

ENSMUST00000033771 1080 Opn1mw 0.2847457646295755

ENSMUST00000043498 1287 Hdac3 0.27989687083935566

ENSMUST00000030901 1803 Cpsf3l 0.30423296682980133

ENSMUST00000034874 1581 Cyp11a1 0.30626499588654

ENSMUST00000038369 1236 Cipc 0.25199603221968153

ENSMUST00000038816 297 Cxcl10 0.23272787214000804

ENSMUST00000039665 2007 Troap 0.24689046841662252

ENSMUST00000034363 1161 Hsd11b2 0.30382176360339236

ENSMUST00000035987 3234 Map3k9 0.29872384051131673

ENSMUST00000041577 2352 Bank1 0.19212090990514702

ENSMUST00000031456 2571 Pde6b 0.30075267621528723

ENSMUST00000047852 1167 Fam199x 0.24934776892935495

ENSMUST00000042575 3102 Topors 0.17628217208390257

ENSMUST00000039373 3162 Uba6 0.14001573549933966

ENSMUST00000034096 1422 Setd6 0.2612849973742051

ENSMUST00000044795 3468 Nup133 0.23770290546524053

ENSMUST00000047005 1716 Spata16 0.243201811150006

ENSMUST00000030206 1224 Snapc3 0.21576104571300422

ENSMUST00000043983 3585 Igsf3 0.35551414230951217

ENSMUST00000036049 993 Hsdl1 0.350892777951881

ENSMUST00000038295 3522 Plekha6 0.3383445989745684

ENSMUST00000046959 1662 Slc22a2 0.2547762295692244

ENSMUST00000039101 873 Rwdd2b 0.22926417245053654

ENSMUST00000030665 999 Nudc 0.39151156386553265

ENSMUST00000039059 2313 Pcsk7 0.2801842106901687

ENSMUST00000046154 2250 Lin54 0.17373382654723704

ENSMUST00000030486 1530 Cyp4a31 0.24787268938021578

ENSMUST00000044977 999 Slc25a16 0.18879256590262564

ENSMUST00000050183 4242 Uaca 0.2847643051246519

ENSMUST00000036370 2145 Tagap 0.26707524700558927
[truncated: 961,264 more chars]
