# Supplementary material for: Systematic Analysis of Mouse Genome Reveals Distinct Evolutionary and Functional Properties Among Circadian and Ultradian Genes
Source: Front Physiol. 2018 Aug 23;9:1178. doi: 10.3389/fphys.2018.01178 (PMC6115496; doi:10.3389/fphys.2018.01178)
Supplement: FILE S3 — Command lines and raw output. [file Data_Sheet_3.doc]

### AME output ###

# Version 4.12.0, implemented at June 2017 #

# Example command line

# ame --o /path/to/FOLDER/out --verbose 1 --control control_1.fasta Mus_GRCm38_ens84_12h_5000bp_flank-coding.fasta /opt/meme_4.11.4/db/motif_databases/MOUSE/chen2008.meme /opt/meme_4.11.4/db/motif_databases/MOUSE/HOCOMOCOv10_MOUSE_mono_meme_format.meme /opt/meme_4.11.4/db/motif_databases/MOUSE/uniprobe_mouse.meme

# Significant enriched TFBS was found for the following comparisons:

# 1. 12h vs. 24h (12h_24h_AME_out)

# 2. 12h vs. set 1 of 500 control sequences (12h_AME_500_1_out)

# 3. 12h vs. set 2 of 500 control sequences (12h_AME_500_2_out)

# 4. 12h vs. set 3 of 500 control sequences (12h_AME_500_3_out)

# 5. 12h vs. set 4 of 500 control sequences (12h_AME_500_4_out)

# 6. 12h vs. set 5 of 500 control sequences (12h_AME_500_5_out)

# 7. 12h vs. set 1 of 1500 control sequences (12h_AME_control_1_out)

# 8. 12h vs. set 2 of 1500 control sequences (12h_AME_control_2_out)

# 9. 12h vs. set 3 of 1500 control sequences (12h_AME_control_3_out)

# 10. 12h vs. set 4 of 1500 control sequences (12h_AME_control_4_out)

# 11. 12h vs. set 5 of 1500 control sequences (12h_AME_control_5_out)

# 12. 24h vs. set 2 of 1500 control sequences (24h_AME_control_2_out)

**Analysis #1**

1. Fisher's exact test p-value of motif XBP1_MOUSE.H10MO.C (GACGTGKCMTWW) top 202 seqs: 4.2e-08 (Corrected p-value: 3.465e-05)

2. Fisher's exact test p-value of motif UP00065_1 Zfp161_primary (KGGCGCGCGCRCHYRD) top 202 seqs: 9.297e-08 (Corrected p-value: 7.67e-05)

3. Fisher's exact test p-value of motif UP00065_2 Zfp161_secondary (GYCGCGCARNGCRN) top 202 seqs: 2.778e-07 (Corrected p-value: 0.0002291)

4. Fisher's exact test p-value of motif UP00013_1 Gabpa_primary (MNWWACCGGAAGTDNNN) top 202 seqs: 2.385e-06 (Corrected p-value: 0.001966)

5. Fisher's exact test p-value of motif MBD2_MOUSE.H10MO.B (SSGKCCGGMGR) top 202 seqs: 2.44e-06 (Corrected p-value: 0.002011)

6. Fisher's exact test p-value of motif EGR4_MOUSE.H10MO.D (GGSGGYRGGGM) top 202 seqs: 7.457e-06 (Corrected p-value: 0.006133)

7. Fisher's exact test p-value of motif UP00001_1 E2F2_primary (NHWARGGCGCGCSAH) top 202 seqs: 4.37e-05 (Corrected p-value: 0.03541)

8. Fisher's exact test p-value of motif UP00241_1 Hoxd3_1742.2 (HTGDDBTAATTAMHBT) top 202 seqs: 4.473e-05 (Corrected p-value: 0.03623)

9. Fisher's exact test p-value of motif FLI1_MOUSE.H10MO.A (SVVRCCGGAAGTGGV) top 202 seqs: 4.554e-05 (Corrected p-value: 0.03687)

AME (Analysis of Motif Enrichment): Compiled on Jun 5 2017 at 14:51:16

------------------------------

**Analysis #2**

1. Fisher's exact test p-value of motif XBP1_MOUSE.H10MO.C (GACGTGKCMTWW) top 202 seqs: 1.157e-06 (Corrected p-value: 0.0009538)

2. Fisher's exact test p-value of motif UP00065_2 Zfp161_secondary (GYCGCGCARNGCRN) top 202 seqs: 2.579e-06 (Corrected p-value: 0.002126)

3. Fisher's exact test p-value of motif MBD2_MOUSE.H10MO.B (SSGKCCGGMGR) top 202 seqs: 4.518e-06 (Corrected p-value: 0.003721)

4. Fisher's exact test p-value of motif UP00065_1 Zfp161_primary (KGGCGCGCGCRCHYRD) top 202 seqs: 4.899e-06 (Corrected p-value: 0.004034)

AME (Analysis of Motif Enrichment): Compiled on Jun 5 2017 at 14:51:16

------------------------------

**Analysis #3**

1. Fisher's exact test p-value of motif UP00065_2 Zfp161_secondary (GYCGCGCARNGCRN) top 202 seqs: 2.079e-08 (Corrected p-value: 1.715e-05)

2. Fisher's exact test p-value of motif UP00065_1 Zfp161_primary (KGGCGCGCGCRCHYRD) top 202 seqs: 1.611e-07 (Corrected p-value: 0.0001329)

3. Fisher's exact test p-value of motif MBD2_MOUSE.H10MO.B (SSGKCCGGMGR) top 202 seqs: 2.529e-07 (Corrected p-value: 0.0002086)

4. Fisher's exact test p-value of motif UP00013_1 Gabpa_primary (MNWWACCGGAAGTDNNN) top 202 seqs: 5.19e-07 (Corrected p-value: 0.0004281)

5. Fisher's exact test p-value of motif XBP1_MOUSE.H10MO.C (GACGTGKCMTWW) top 202 seqs: 7.077e-07 (Corrected p-value: 0.0005836)

6. Fisher's exact test p-value of motif UP00001_1 E2F2_primary (NHWARGGCGCGCSAH) top 202 seqs: 1.298e-06 (Corrected p-value: 0.00107)

7. Fisher's exact test p-value of motif E2F3_MOUSE.H10MO.B (SSCGCSAAAC) top 202 seqs: 1.611e-05 (Corrected p-value: 0.0132)

8. Fisher's exact test p-value of motif GABPA_MOUSE.H10MO.A (SVRCCGGAAGTGV) top 202 seqs: 1.736e-05 (Corrected p-value: 0.01422)

9. Fisher's exact test p-value of motif EGR4_MOUSE.H10MO.D (GGSGGYRGGGM) top 202 seqs: 2.129e-05 (Corrected p-value: 0.01741)

10. Fisher's exact test p-value of motif MECP2_MOUSE.H10MO.C (SCCGGRR) top 202 seqs: 2.826e-05 (Corrected p-value: 0.02304)

11. Fisher's exact test p-value of motif AP2D_MOUSE.H10MO.D (CGCCYGVGGCSCGT) top 202 seqs: 3.231e-05 (Corrected p-value: 0.02631)

12. Fisher's exact test p-value of motif UP00000_2 Smad3_secondary (KHHNCCCCGCCAMYYYB) top 202 seqs: 3.242e-05 (Corrected p-value: 0.02639)

13. Fisher's exact test p-value of motif FLI1_MOUSE.H10MO.A (SVVRCCGGAAGTGGV) top 202 seqs: 3.862e-05 (Corrected p-value: 0.03136)

14. Fisher's exact test p-value of motif UP00003_1 E2F3_primary (VHDADGGCGCGCSHW) top 202 seqs: 6.172e-05 (Corrected p-value: 0.04964)

AME (Analysis of Motif Enrichment): Compiled on Jun 5 2017 at 14:51:16

------------------------------

**Analysis #4**

1. Fisher's exact test p-value of motif UP00065_2 Zfp161_secondary (GYCGCGCARNGCRN) top 202 seqs: 4.553e-09 (Corrected p-value: 3.756e-06)

2. Fisher's exact test p-value of motif UP00065_1 Zfp161_primary (KGGCGCGCGCRCHYRD) top 202 seqs: 2.091e-07 (Corrected p-value: 0.0001725)

3. Fisher's exact test p-value of motif XBP1_MOUSE.H10MO.C (GACGTGKCMTWW) top 202 seqs: 1.157e-06 (Corrected p-value: 0.0009538)

4. Fisher's exact test p-value of motif UP00013_1 Gabpa_primary (MNWWACCGGAAGTDNNN) top 202 seqs: 4.425e-06 (Corrected p-value: 0.003644)

5. Fisher's exact test p-value of motif GABPA_MOUSE.H10MO.A (SVRCCGGAAGTGV) top 202 seqs: 6.121e-06 (Corrected p-value: 0.005037)

6. Fisher's exact test p-value of motif MBD2_MOUSE.H10MO.B (SSGKCCGGMGR) top 202 seqs: 7.058e-06 (Corrected p-value: 0.005806)

7. Fisher's exact test p-value of motif UP00084_2 Gmeb1_secondary (KGRBCRACGTYGTYHW) top 202 seqs: 1.149e-05 (Corrected p-value: 0.009433)

8. Fisher's exact test p-value of motif FLI1_MOUSE.H10MO.A (SVVRCCGGAAGTGGV) top 202 seqs: 2.196e-05 (Corrected p-value: 0.01796)

9. Fisher's exact test p-value of motif UP00241_1 Hoxd3_1742.2 (HTGDDBTAATTAMHBT) top 202 seqs: 3.639e-05 (Corrected p-value: 0.02958)

10. Fisher's exact test p-value of motif HMGA2_MOUSE.H10MO.D (AATWWYSSSSAATAT) top 202 seqs: 3.686e-05 (Corrected p-value: 0.02995)

11. Fisher's exact test p-value of motif EGR4_MOUSE.H10MO.D (GGSGGYRGGGM) top 202 seqs: 5.19e-05 (Corrected p-value: 0.04191)

12. Fisher's exact test p-value of motif CRX_MOUSE.H10MO.S (VRGATTAR) top 202 seqs: 5.629e-05 (Corrected p-value: 0.04538)

AME (Analysis of Motif Enrichment): Compiled on Jun 5 2017 at 14:51:16

------------------------------

**Analysis #5**

1. Fisher's exact test p-value of motif XBP1_MOUSE.H10MO.C (GACGTGKCMTWW) top 202 seqs: 5.196e-09 (Corrected p-value: 4.287e-06)

2. Fisher's exact test p-value of motif UP00065_1 Zfp161_primary (KGGCGCGCGCRCHYRD) top 202 seqs: 4.197e-08 (Corrected p-value: 3.463e-05)

3. Fisher's exact test p-value of motif UP00065_2 Zfp161_secondary (GYCGCGCARNGCRN) top 202 seqs: 1.999e-07 (Corrected p-value: 0.0001649)

4. Fisher's exact test p-value of motif MBD2_MOUSE.H10MO.B (SSGKCCGGMGR) top 202 seqs: 3.261e-07 (Corrected p-value: 0.000269)

5. Fisher's exact test p-value of motif EGR4_MOUSE.H10MO.D (GGSGGYRGGGM) top 202 seqs: 3.415e-06 (Corrected p-value: 0.002814)

6. Fisher's exact test p-value of motif UP00013_1 Gabpa_primary (MNWWACCGGAAGTDNNN) top 202 seqs: 3.514e-06 (Corrected p-value: 0.002895)

7. Fisher's exact test p-value of motif CREB1_MOUSE.H10MO.B (RTGACGTMA) top 202 seqs: 2.046e-05 (Corrected p-value: 0.01674)

AME (Analysis of Motif Enrichment): Compiled on Jun 5 2017 at 14:51:16

--------------------------

**Analysis #6**

1. Fisher's exact test p-value of motif UP00065_1 Zfp161_primary (KGGCGCGCGCRCHYRD) top 202 seqs: 4.227e-09 (Corrected p-value: 3.487e-06)

2. Fisher's exact test p-value of motif UP00065_2 Zfp161_secondary (GYCGCGCARNGCRN) top 202 seqs: 6.609e-08 (Corrected p-value: 5.452e-05)

3. Fisher's exact test p-value of motif XBP1_MOUSE.H10MO.C (GACGTGKCMTWW) top 202 seqs: 2.371e-06 (Corrected p-value: 0.001954)

4. Fisher's exact test p-value of motif UP00002_2 Sp4_secondary (BWWAGGCGTGKCYND) top 202 seqs: 5.051e-06 (Corrected p-value: 0.004158)

5. Fisher's exact test p-value of motif MBD2_MOUSE.H10MO.B (SSGKCCGGMGR) top 202 seqs: 5.654e-06 (Corrected p-value: 0.004654)

6. Fisher's exact test p-value of motif EGR4_MOUSE.H10MO.D (GGSGGYRGGGM) top 202 seqs: 8.593e-06 (Corrected p-value: 0.007064)

7. Fisher's exact test p-value of motif UP00013_1 Gabpa_primary (MNWWACCGGAAGTDNNN) top 202 seqs: 1.694e-05 (Corrected p-value: 0.01388)

8. Fisher's exact test p-value of motif UP00000_2 Smad3_secondary (KHHNCCCCGCCAMYYYB) top 202 seqs: 2.623e-05 (Corrected p-value: 0.0214)

9. Fisher's exact test p-value of motif E4F1_MOUSE.H10MO.D (YGTKACGTC) top 202 seqs: 2.94e-05 (Corrected p-value: 0.02396)

10. Fisher's exact test p-value of motif AP2D_MOUSE.H10MO.D (CGCCYGVGGCSCGT) top 202 seqs: 3.231e-05 (Corrected p-value: 0.02631)

11. Fisher's exact test p-value of motif CREB1_MOUSE.H10MO.B (RTGACGTMA) top 202 seqs: 4.964e-05 (Corrected p-value: 0.04013)

AME (Analysis of Motif Enrichment): Compiled on Jun 5 2017 at 14:51:16

------------------------------

**Analysis #7**

1. Fisher's exact test p-value of motif UP00065_1 Zfp161_primary (KGGCGCGCGCRCHYRD) top 202 seqs: 4.938e-10 (Corrected p-value: 4.074e-07)

2. Fisher's exact test p-value of motif XBP1_MOUSE.H10MO.C (GACGTGKCMTWW) top 202 seqs: 9.453e-09 (Corrected p-value: 7.798e-06)

3. Fisher's exact test p-value of motif UP00065_2 Zfp161_secondary (GYCGCGCARNGCRN) top 202 seqs: 1.745e-08 (Corrected p-value: 1.439e-05)

4. Fisher's exact test p-value of motif MBD2_MOUSE.H10MO.B (SSGKCCGGMGR) top 202 seqs: 1.013e-07 (Corrected p-value: 8.358e-05)

5. Fisher's exact test p-value of motif UP00013_1 Gabpa_primary (MNWWACCGGAAGTDNNN) top 202 seqs: 5.484e-06 (Corrected p-value: 0.004514)

6. Fisher's exact test p-value of motif EGR4_MOUSE.H10MO.D (GGSGGYRGGGM) top 202 seqs: 5.609e-06 (Corrected p-value: 0.004617)

7. Fisher's exact test p-value of motif GABPA_MOUSE.H10MO.A (SVRCCGGAAGTGV) top 202 seqs: 7.164e-06 (Corrected p-value: 0.005893)

8. Fisher's exact test p-value of motif CREB1_MOUSE.H10MO.B (RTGACGTMA) top 202 seqs: 2.88e-05 (Corrected p-value: 0.02348)

9. Fisher's exact test p-value of motif NMYC (CGCACGTGGC) top 202 seqs: 3.353e-05 (Corrected p-value: 0.02728)

10. Fisher's exact test p-value of motif UP00001_1 E2F2_primary (NHWARGGCGCGCSAH) top 202 seqs: 3.376e-05 (Corrected p-value: 0.02747)

11. Fisher's exact test p-value of motif UP00000_2 Smad3_secondary (KHHNCCCCGCCAMYYYB) top 202 seqs: 3.544e-05 (Corrected p-value: 0.02881)

12. Fisher's exact test p-value of motif SP4_MOUSE.H10MO.D (GGGGCCRGGGGSGGGGSGGSSSSG) top 202 seqs: 4.613e-05 (Corrected p-value: 0.03734)

AME (Analysis of Motif Enrichment): Compiled on Jun 5 2017 at 14:51:16

------------------------------

**Analysis #8**

1. Fisher's exact test p-value of motif XBP1_MOUSE.H10MO.C (GACGTGKCMTWW) top 202 seqs: 6.775e-12 (Corrected p-value: 5.589e-09)

2. Fisher's exact test p-value of motif UP00065_1 Zfp161_primary (KGGCGCGCGCRCHYRD) top 202 seqs: 3.859e-09 (Corrected p-value: 3.184e-06)

3. Fisher's exact test p-value of motif UP00065_2 Zfp161_secondary (GYCGCGCARNGCRN) top 202 seqs: 7.021e-08 (Corrected p-value: 5.792e-05)

4. Fisher's exact test p-value of motif MBD2_MOUSE.H10MO.B (SSGKCCGGMGR) top 202 seqs: 1.117e-07 (Corrected p-value: 9.216e-05)

5. Fisher's exact test p-value of motif UP00013_1 Gabpa_primary (MNWWACCGGAAGTDNNN) top 202 seqs: 3.201e-07 (Corrected p-value: 0.0002641)

6. Fisher's exact test p-value of motif E4F1_MOUSE.H10MO.D (YGTKACGTC) top 202 seqs: 3.477e-06 (Corrected p-value: 0.002864)

7. Fisher's exact test p-value of motif FLI1_MOUSE.H10MO.A (SVVRCCGGAAGTGGV) top 202 seqs: 3.49e-06 (Corrected p-value: 0.002875)

8. Fisher's exact test p-value of motif MECP2_MOUSE.H10MO.C (SCCGGRR) top 202 seqs: 4.332e-06 (Corrected p-value: 0.003567)

9. Fisher's exact test p-value of motif GABPA_MOUSE.H10MO.A (SVRCCGGAAGTGV) top 202 seqs: 7.164e-06 (Corrected p-value: 0.005893)

10. Fisher's exact test p-value of motif EGR4_MOUSE.H10MO.D (GGSGGYRGGGM) top 202 seqs: 8.847e-06 (Corrected p-value: 0.007272)

11. Fisher's exact test p-value of motif CREB1_MOUSE.H10MO.B (RTGACGTMA) top 202 seqs: 1.132e-05 (Corrected p-value: 0.009298)

12. Fisher's exact test p-value of motif GABP1_MOUSE.H10MO.C (CCGGAAGTGV) top 202 seqs: 2.07e-05 (Corrected p-value: 0.01693)

13. Fisher's exact test p-value of motif UP00001_1 E2F2_primary (NHWARGGCGCGCSAH) top 202 seqs: 5.639e-05 (Corrected p-value: 0.04546)

AME (Analysis of Motif Enrichment): Compiled on Jun 5 2017 at 14:51:16

------------------------------

**Analysis #9**

1. Fisher's exact test p-value of motif UP00065_1 Zfp161_primary (KGGCGCGCGCRCHYRD) top 202 seqs: 5.989e-09 (Corrected p-value: 4.941e-06)

2. Fisher's exact test p-value of motif UP00065_2 Zfp161_secondary (GYCGCGCARNGCRN) top 202 seqs: 2.172e-08 (Corrected p-value: 1.792e-05)

3. Fisher's exact test p-value of motif MBD2_MOUSE.H10MO.B (SSGKCCGGMGR) top 202 seqs: 6.829e-08 (Corrected p-value: 5.634e-05)

4. Fisher's exact test p-value of motif XBP1_MOUSE.H10MO.C (GACGTGKCMTWW) top 202 seqs: 8.418e-08 (Corrected p-value: 6.945e-05)

5. Fisher's exact test p-value of motif GABPA_MOUSE.H10MO.A (SVRCCGGAAGTGV) top 202 seqs: 8.651e-07 (Corrected p-value: 0.0007135)

6. Fisher's exact test p-value of motif FLI1_MOUSE.H10MO.A (SVVRCCGGAAGTGGV) top 202 seqs: 1.796e-06 (Corrected p-value: 0.00148)

7. Fisher's exact test p-value of motif UP00013_1 Gabpa_primary (MNWWACCGGAAGTDNNN) top 202 seqs: 2.129e-06 (Corrected p-value: 0.001755)

8. Fisher's exact test p-value of motif UP00001_1 E2F2_primary (NHWARGGCGCGCSAH) top 202 seqs: 7.868e-06 (Corrected p-value: 0.00647)

9. Fisher's exact test p-value of motif EGR4_MOUSE.H10MO.D (GGSGGYRGGGM) top 202 seqs: 8.847e-06 (Corrected p-value: 0.007272)

10. Fisher's exact test p-value of motif CRX_MOUSE.H10MO.S (VRGATTAR) top 202 seqs: 4.258e-05 (Corrected p-value: 0.03452)

AME (Analysis of Motif Enrichment): Compiled on Jun 5 2017 at 14:51:16

------------------------------

**Analysis #10**

1. Fisher's exact test p-value of motif MBD2_MOUSE.H10MO.B (SSGKCCGGMGR) top 202 seqs: 5.088e-09 (Corrected p-value: 4.197e-06)

2. Fisher's exact test p-value of motif UP00065_1 Zfp161_primary (KGGCGCGCGCRCHYRD) top 202 seqs: 1.748e-08 (Corrected p-value: 1.442e-05)

3. Fisher's exact test p-value of motif UP00065_2 Zfp161_secondary (GYCGCGCARNGCRN) top 202 seqs: 2.699e-08 (Corrected p-value: 2.226e-05)

4. Fisher's exact test p-value of motif XBP1_MOUSE.H10MO.C (GACGTGKCMTWW) top 202 seqs: 1.525e-07 (Corrected p-value: 0.0001258)

5. Fisher's exact test p-value of motif UP00013_1 Gabpa_primary (MNWWACCGGAAGTDNNN) top 202 seqs: 6.678e-07 (Corrected p-value: 0.0005508)

6. Fisher's exact test p-value of motif EGR4_MOUSE.H10MO.D (GGSGGYRGGGM) top 202 seqs: 2.812e-06 (Corrected p-value: 0.002317)

7. Fisher's exact test p-value of motif NMYC (CGCACGTGGC) top 202 seqs: 1.246e-05 (Corrected p-value: 0.01022)

8. Fisher's exact test p-value of motif UP00001_1 E2F2_primary (NHWARGGCGCGCSAH) top 202 seqs: 1.258e-05 (Corrected p-value: 0.01032)

9. Fisher's exact test p-value of motif MECP2_MOUSE.H10MO.C (SCCGGRR) top 202 seqs: 1.669e-05 (Corrected p-value: 0.01367)

10. Fisher's exact test p-value of motif GABPA_MOUSE.H10MO.A (SVRCCGGAAGTGV) top 202 seqs: 1.711e-05 (Corrected p-value: 0.01402)

11. Fisher's exact test p-value of motif CRX_MOUSE.H10MO.S (VRGATTAR) top 202 seqs: 2.228e-05 (Corrected p-value: 0.01822)

12. Fisher's exact test p-value of motif GABP1_MOUSE.H10MO.C (CCGGAAGTGV) top 202 seqs: 5.786e-05 (Corrected p-value: 0.04661)

13. Fisher's exact test p-value of motif UP00000_2 Smad3_secondary (KHHNCCCCGCCAMYYYB) top 202 seqs: 6.081e-05 (Corrected p-value: 0.04893)

14. Fisher's exact test p-value of motif UP00241_1 Hoxd3_1742.2 (HTGDDBTAATTAMHBT) top 202 seqs: 6.162e-05 (Corrected p-value: 0.04957)

AME (Analysis of Motif Enrichment): Compiled on Jun 5 2017 at 14:51:16

------------------------------

**Analysis #11**

1. Fisher's exact test p-value of motif XBP1_MOUSE.H10MO.C (GACGTGKCMTWW) top 202 seqs: 2.507e-09 (Corrected p-value: 2.068e-06)

2. Fisher's exact test p-value of motif UP00065_1 Zfp161_primary (KGGCGCGCGCRCHYRD) top 202 seqs: 3.265e-08 (Corrected p-value: 2.693e-05)

3. Fisher's exact test p-value of motif UP00065_2 Zfp161_secondary (GYCGCGCARNGCRN) top 202 seqs: 5.694e-08 (Corrected p-value: 4.698e-05)

4. Fisher's exact test p-value of motif MBD2_MOUSE.H10MO.B (SSGKCCGGMGR) top 202 seqs: 3.25e-06 (Corrected p-value: 0.002678)

5. Fisher's exact test p-value of motif UP00013_1 Gabpa_primary (MNWWACCGGAAGTDNNN) top 202 seqs: 3.286e-06 (Corrected p-value: 0.002707)

6. Fisher's exact test p-value of motif EGR4_MOUSE.H10MO.D (GGSGGYRGGGM) top 202 seqs: 7.048e-06 (Corrected p-value: 0.005797)

7. Fisher's exact test p-value of motif MECP2_MOUSE.H10MO.C (SCCGGRR) top 202 seqs: 3.057e-05 (Corrected p-value: 0.02491)

8. Fisher's exact test p-value of motif CRX_MOUSE.H10MO.S (VRGATTAR) top 202 seqs: 3.885e-05 (Corrected p-value: 0.03154)

9. Fisher's exact test p-value of motif UP00001_1 E2F2_primary (NHWARGGCGCGCSAH) top 202 seqs: 5.183e-05 (Corrected p-value: 0.04186)

AME (Analysis of Motif Enrichment): Compiled on Jun 5 2017 at 14:51:16

------------------------------

**Analysis #12**

1. Fisher's exact test p-value of motif ERR3_MOUSE.H10MO.B (TCAAGGTCA) top 2036 seqs: 4.156e-06 (Corrected p-value: 0.003423)

2. Fisher's exact test p-value of motif E4F1_MOUSE.H10MO.D (YGTKACGTC) top 2036 seqs: 3.907e-05 (Corrected p-value: 0.03171)
