# Supplementary material for: Systematic Analysis of Mouse Genome Reveals Distinct Evolutionary and Functional Properties Among Circadian and Ultradian Genes
Source: Front Physiol. 2018 Aug 23;9:1178. doi: 10.3389/fphys.2018.01178 (PMC6115496; doi:10.3389/fphys.2018.01178)
Supplement: FILE S4 — Day length estimation through ages. [file Data_Sheet_4.doc]

**Long-term changes of Earth’s rotation period (length-of-day)**

The assessment of long-term changes of day length on Earth is a matter of investigation since many decades. While it was first noticed that the length-of-day (LOD) increased during Earth’s history , in 1987 Zahnle and Walker came to the conclusion that there are good arguments that in the Precambrian era, when the deceleration of the LOD would have reached about 21 h, the period would have been resonant with the semidiurnal atmospheric tidal torque (10.5 h). At this time, a stabilizing effect on the LOD would have occurred since the atmospheric tidal torque would have had the same magnitude (but opposite sign) as the lunar oceanic torque. This resonance condition would have stabilized the LOD at a value of about 21 h. Recently, Bartlett and Stevenson came to the conclusion that this resonance condition would have broken by sudden atmospheric temperature increases like the deglaciation happened at the end of the Precambrian (i.e. the Marinoan and Sturtian glaciations), causing a further deceleration of the LOD to the current value of 24 hours.
